# Supplementary material for: Efficient Sequencing, Assembly, and Annotation of Human KIR Haplotypes
Source: Front Immunol. 2020 Oct 9;11:582927. doi: 10.3389/fimmu.2020.582927 (PMC7581912; doi:10.3389/fimmu.2020.582927)

Qualimap Analysis Results

BAM QC analysis

Generated by Qualimap v.2.2.1

2020/04/21 17:50:31

1. Input data & parameters

1.1. QualiMap command line

qualimap bamqc -bam ccs999KIR7_18_8.contigs_MN167525_sorted.bam -nw 400 -hm 3

1.2. Alignment

Command line:	bwa mem -k1800 -W9000 -r10 -A1 -B100 -O40 -E40 -L50 -t7 MN167525.fasta ccs999KIR7_18_8.contigs.fasta.gz
Draw chromosome limits:	no
Analyze overlapping paired-end reads:	no
Program:	bwa (0.7.17-r1188)
Analysis date:	Tue Apr 21 17:50:30 GMT 2020
Size of a homopolymer:	3
Skip duplicate alignments:	no
Number of windows:	400
BAM file:	ccs999KIR7_18_8.contigs_MN167525_sorted.bam

2. Summary

2.1. Globals

Reference size	236,345
Number of reads	15
Mapped reads	15 / 100%
Supplementary alignments	1 / 6.67%
Unmapped reads	0 / 0%
Mapped paired reads	0 / 0%
Read min/max/mean length	2,152 / 123,929 / 32,562.13
Duplicated reads (estimated)	1 / 6.67%
Duplication rate	7.14%
Clipped reads	9 / 60%

2.2. ACGT Content

Number/percentage of A's	92,331 / 27.23%
Number/percentage of C's	76,262 / 22.49%
Number/percentage of T's	87,112 / 25.69%
Number/percentage of G's	83,368 / 24.59%
Number/percentage of N's	0 / 0%
GC Percentage	47.08%

2.3. Coverage

Mean	1.4348
Standard Deviation	1.0566

2.4. Mapping Quality

Mean Mapping Quality	47.85

2.5. Mismatches and indels

General error rate	0.02%
Mismatches	60
Insertions	11
Mapped reads with at least one insertion	33.33%
Deletions	24
Mapped reads with at least one deletion	66.67%
Homopolymer indels	91.43%

2.6. Chromosome stats

**Name**	**Length**	**Mapped bases**	**Mean coverage**	**Standard deviation**
MN167525	236345	339101	1.4348	1.0566

3. Results : Coverage across reference

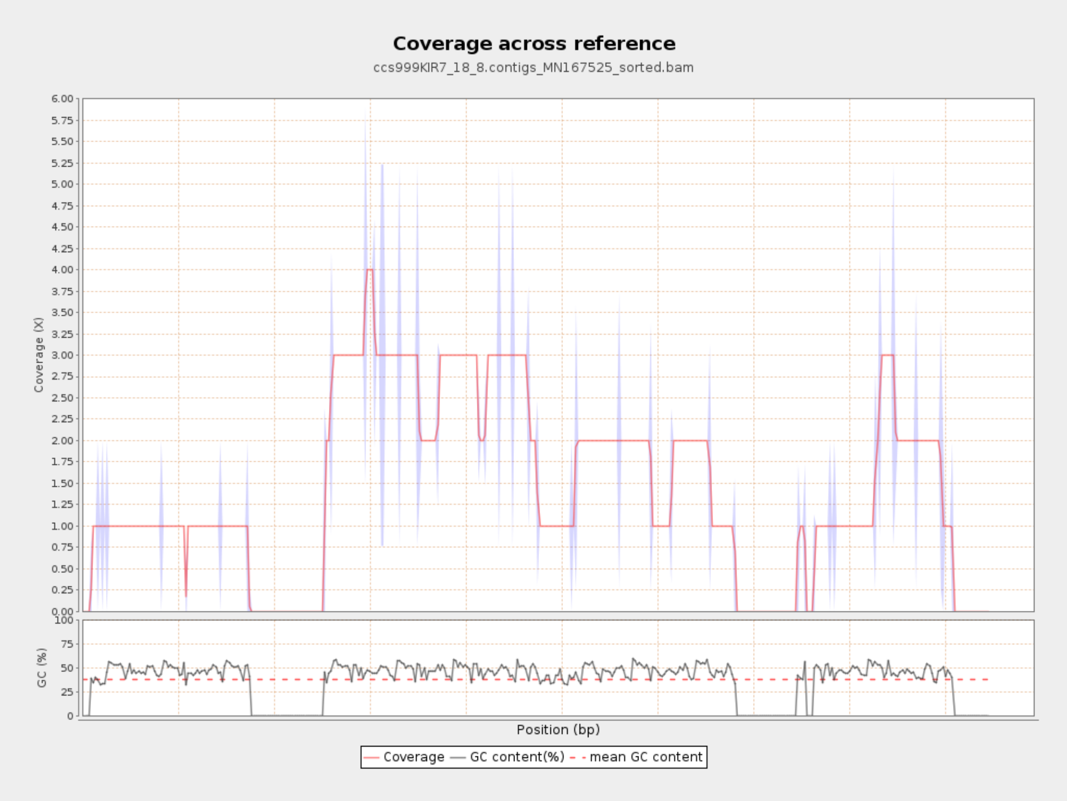

4. Results : Coverage Histogram

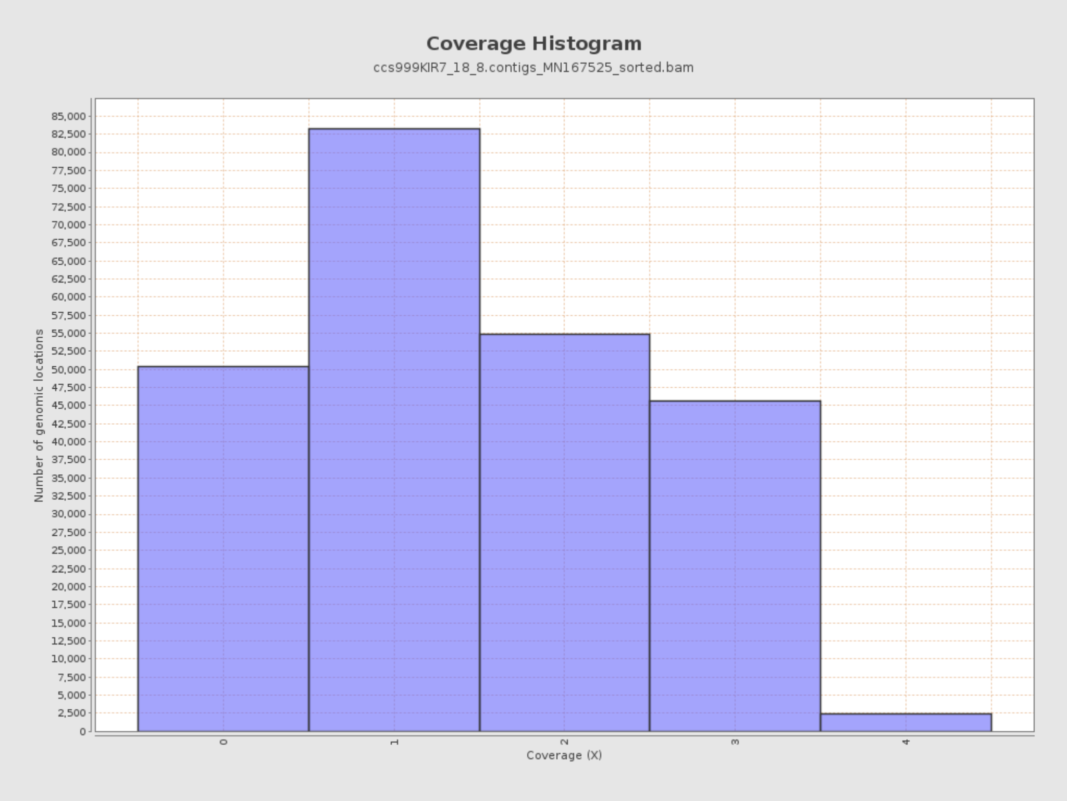

5. Results : Coverage Histogram (0-50X)

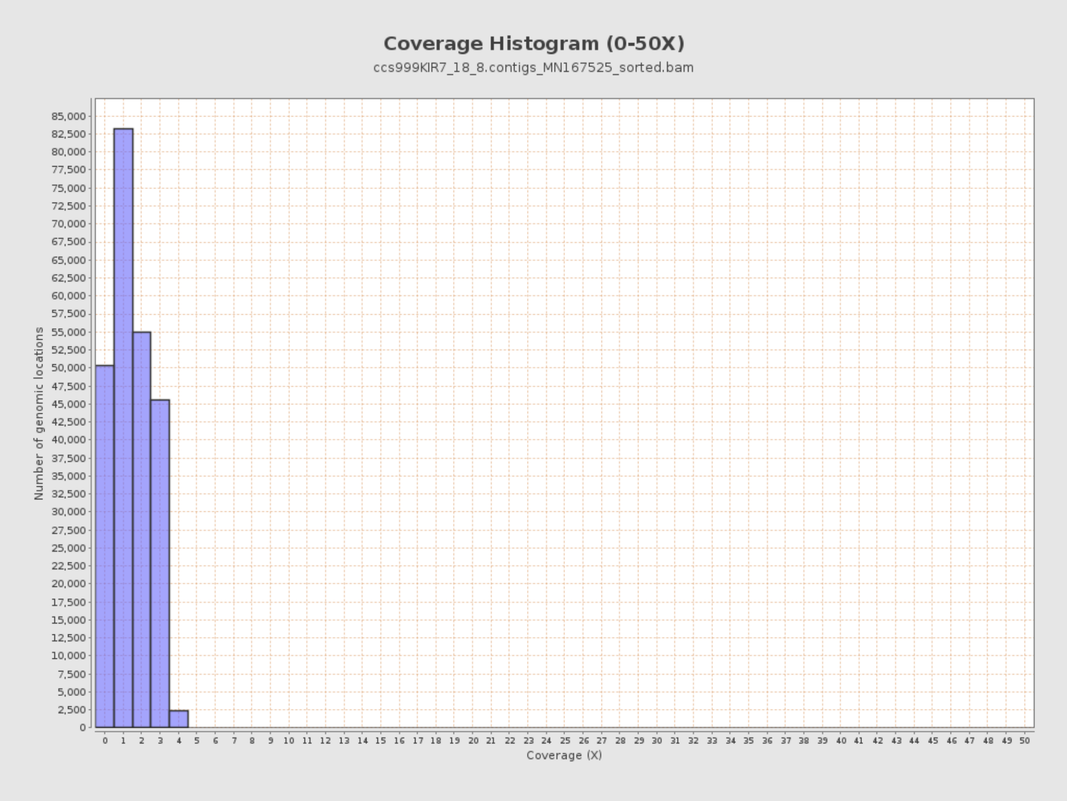

6. Results : Genome Fraction Coverage

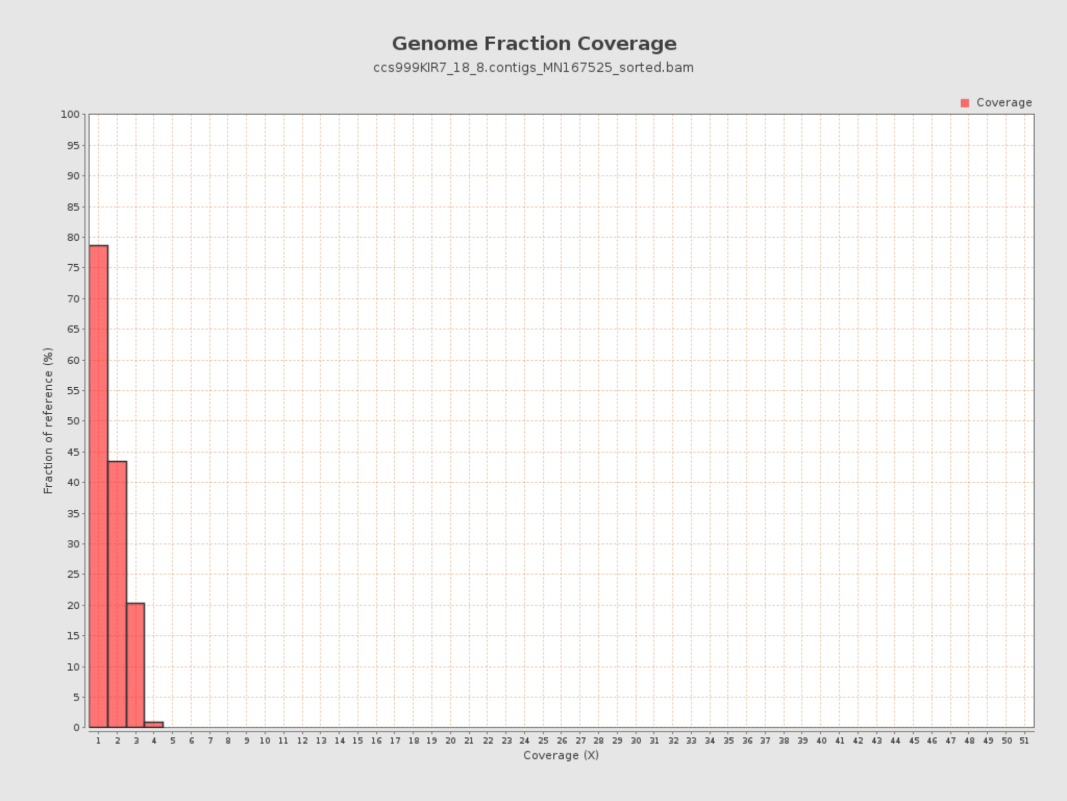

7. Results : Duplication Rate Histogram

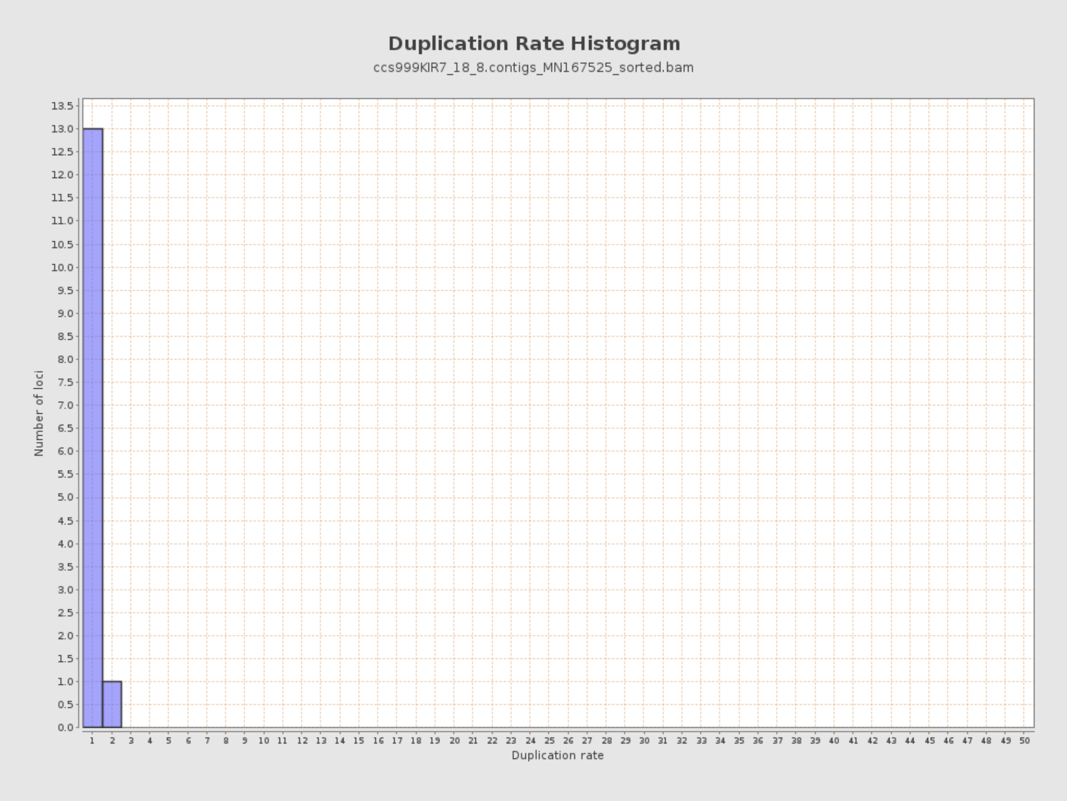

8. Results : Mapped Reads Nucleotide Content

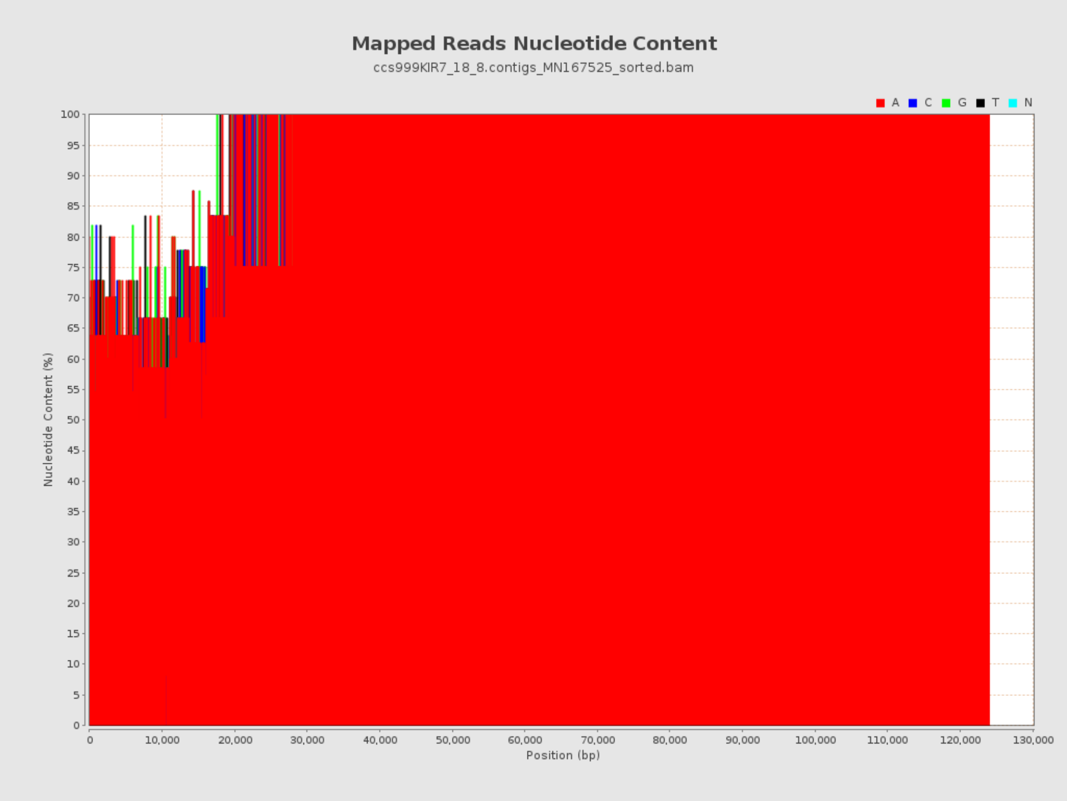

9. Results : Mapped Reads GC-content Distribution

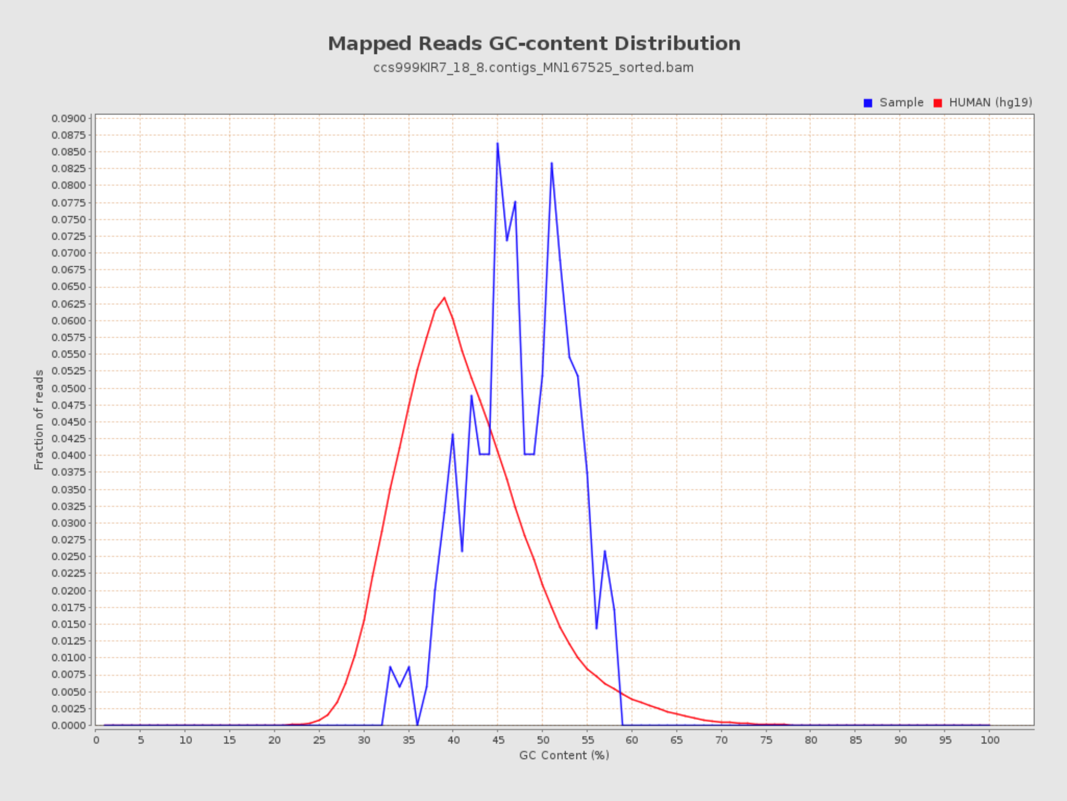

10. Results : Mapped Reads Clipping Profile

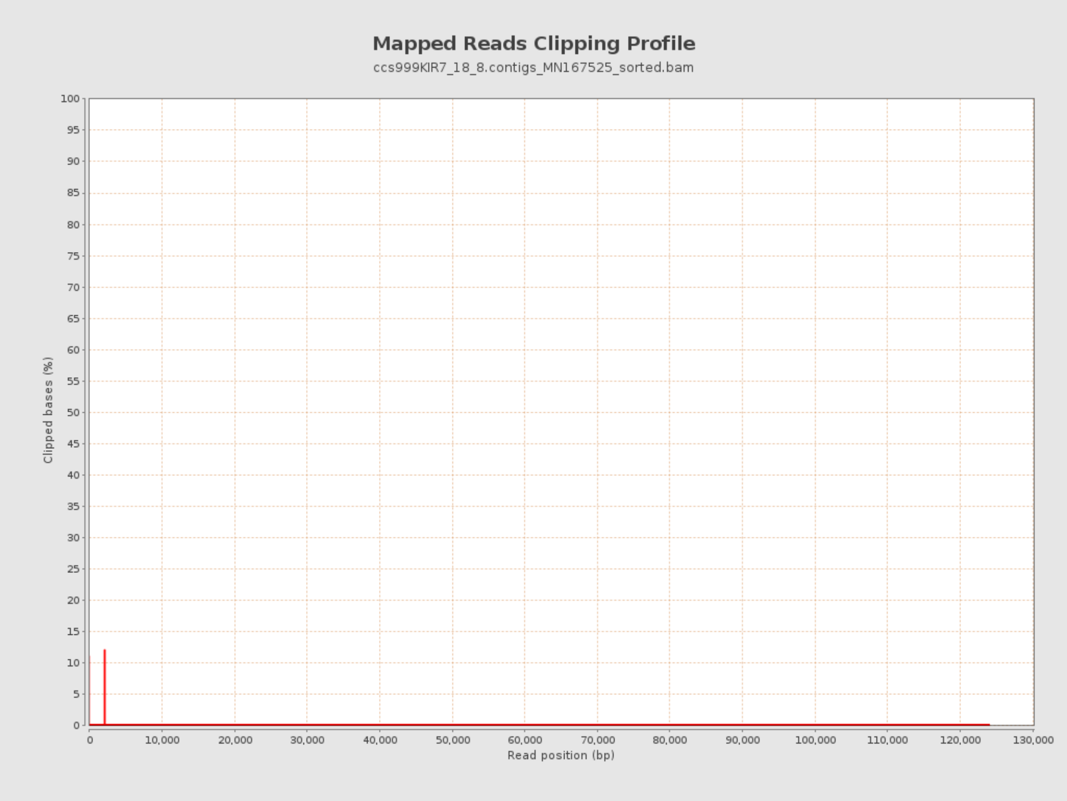

11. Results : Homopolymer Indels

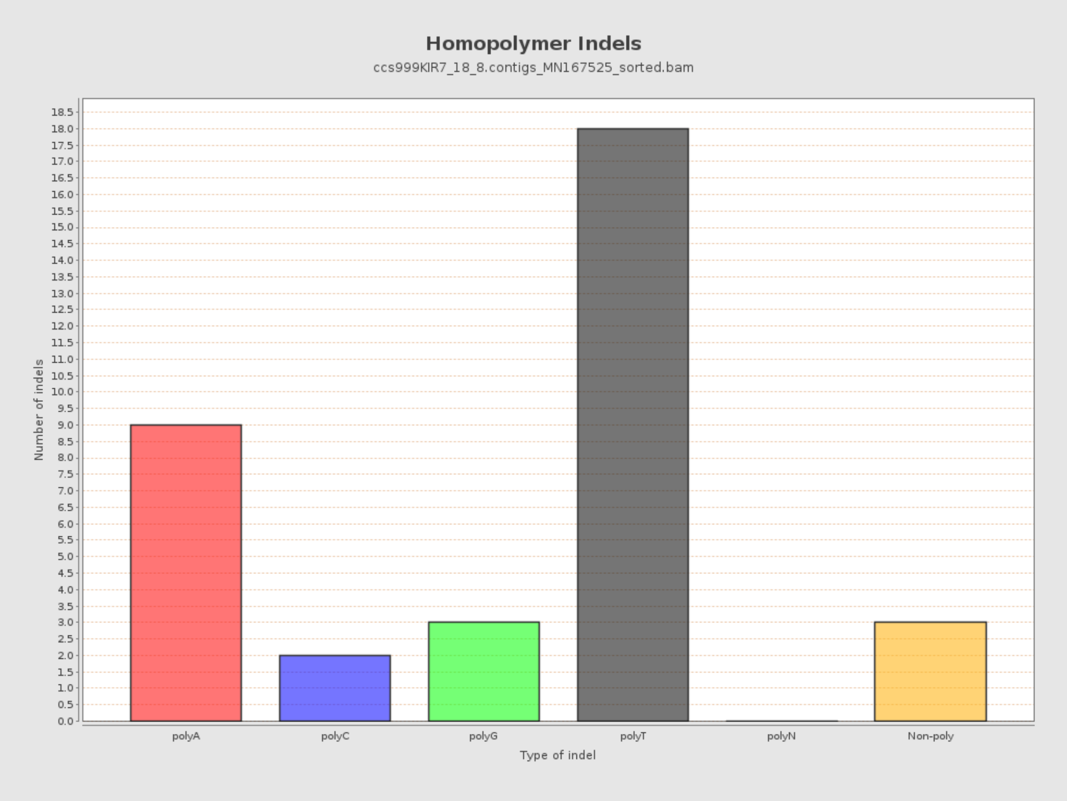

12. Results : Mapping Quality Across Reference

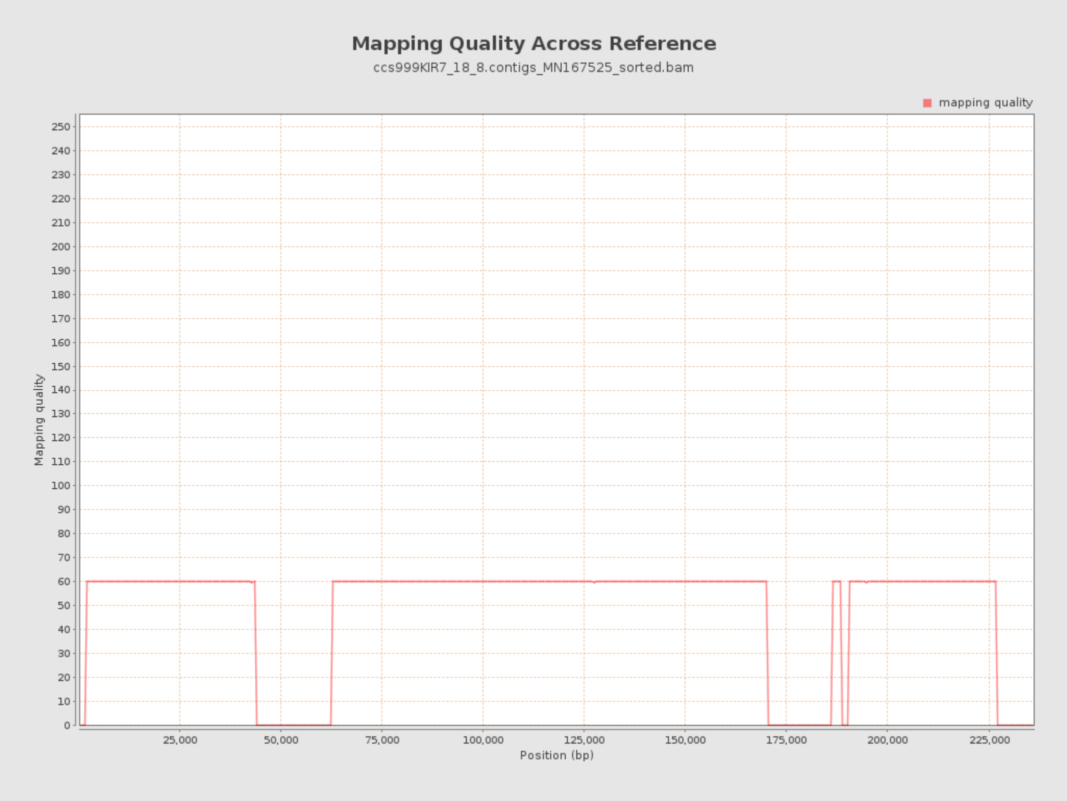

13. Results : Mapping Quality Histogram

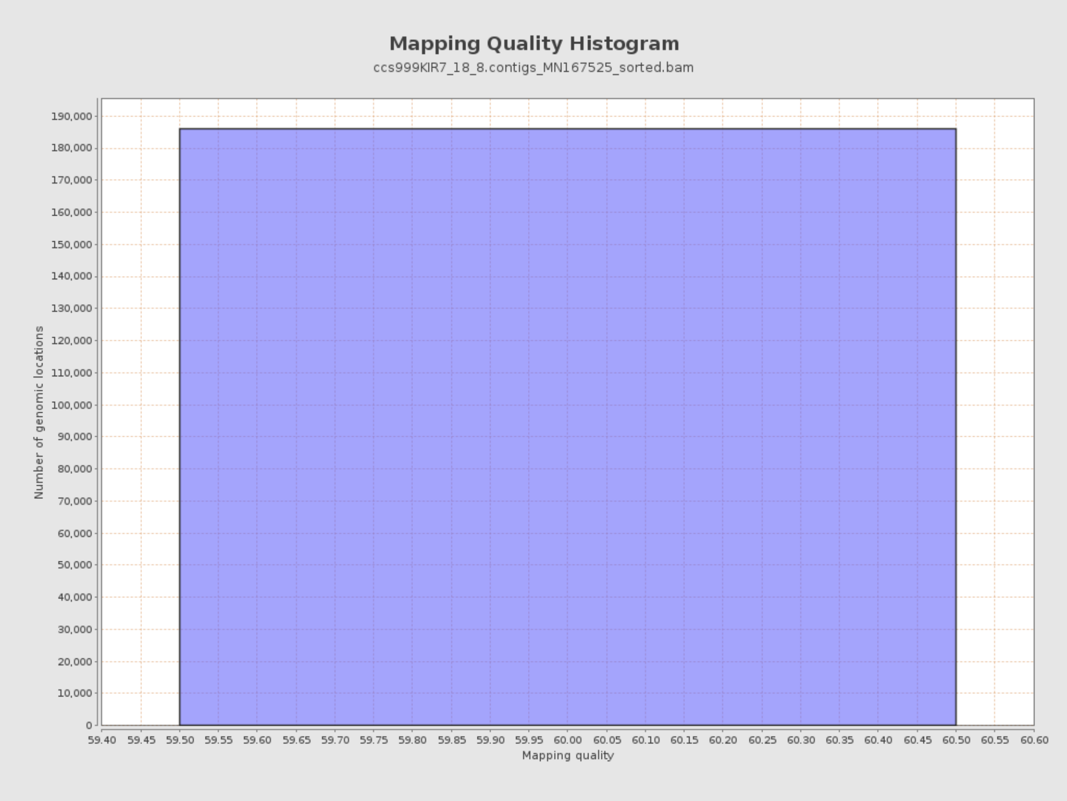

Supplement: Supplementary Figure 1 — AFA and EUR haplotigs. Data Sheets 2-5 are zip files containing the assembled haplotigs for all AFA and EUR assemblies. Also included are Qualimap, NanoPack, and QUAST reports. [file DataSheet_2.zip › SF1d/ccs999KIR7_18_8.contigs_MN167525_reports/ccs999KIR7_18_8.contigs_MN167525_qualimap.pdf]
