# Supplementary material for: Efficient Sequencing, Assembly, and Annotation of Human KIR Haplotypes
Source: Front Immunol. 2020 Oct 9;11:582927. doi: 10.3389/fimmu.2020.582927 (PMC7581912; doi:10.3389/fimmu.2020.582927)
Supplement: Supplementary Figure 1 — AFA and EUR haplotigs. Data Sheets 2-5 are zip files containing the assembled haplotigs for all AFA and EUR assemblies. Also included are Qualimap, NanoPack, and QUAST reports. [file DataSheet_2.zip › SF1d/ccs999KIR7_18_8.contigs_MN167525_reports/quast/icarus.html]

|  |
| --- |
| Icarus **QUAST Contig Browser** by CAB |

**Assemblies:** ccs999KIR7\_18\_8.contigs| Contig size viewer |
| QUAST report |

  

Contig alignment viewer

Aligned to sequences from MN167525.fasta

Fragments: 1, length: 236 345 bp, mean genome fraction: 95.833%,
misassembled blocks: 3
