# Supplementary material for: Efficient Sequencing, Assembly, and Annotation of Human KIR Haplotypes
Source: Front Immunol. 2020 Oct 9;11:582927. doi: 10.3389/fimmu.2020.582927 (PMC7581912; doi:10.3389/fimmu.2020.582927)

Qualimap Analysis Results

BAM QC analysis

Generated by Qualimap v.2.2.1

2020/04/21 17:50:01

1. Input data & parameters

1.1. QualiMap command line

qualimap bamqc -bam ccs999KIR7_18_8.contigs_MN167524_sorted.bam -nw 400 -hm 3

1.2. Alignment

Command line:	bwa mem -k1800 -W9000 -r10 -A1 -B100 -O40 -E40 -L50 -t7 MN167524.fasta ccs999KIR7_18_8.contigs.fasta.gz
Draw chromosome limits:	no
Analyze overlapping paired-end reads:	no
Program:	bwa (0.7.17-r1188)
Analysis date:	Tue Apr 21 17:50:00 GMT 2020
Size of a homopolymer:	3
Skip duplicate alignments:	no
Number of windows:	400
BAM file:	ccs999KIR7_18_8.contigs_MN167524_sorted.bam

2. Summary

2.1. Globals

Reference size	149,229
Number of reads	6
Mapped reads	6 / 100%
Unmapped reads	0 / 0%
Mapped paired reads	0 / 0%
Read min/max/mean length	21,128 / 123,929 / 41,560.33
Duplicated reads (estimated)	0 / 0%
Duplication rate	0%
Clipped reads	3 / 50%

2.2. ACGT Content

Number/percentage of A's	53,490 / 27.28%
Number/percentage of C's	43,814 / 22.34%
Number/percentage of T's	50,778 / 25.89%
Number/percentage of G's	48,027 / 24.49%
Number/percentage of N's	0 / 0%
GC Percentage	46.83%

2.3. Coverage

Mean	1.3142
Standard Deviation	1.0493

2.4. Mapping Quality

Mean Mapping Quality	41.55

2.5. Mismatches and indels

General error rate	0.01%
Mismatches	26
Insertions	1
Mapped reads with at least one insertion	16.67%
Deletions	12
Mapped reads with at least one deletion	83.33%
Homopolymer indels	100%

2.6. Chromosome stats

Name	Length	Mapped bases	Mean coverage	Standard deviation
MN167524	149229	196121	1.3142	1.0493

3. Results : Coverage across reference

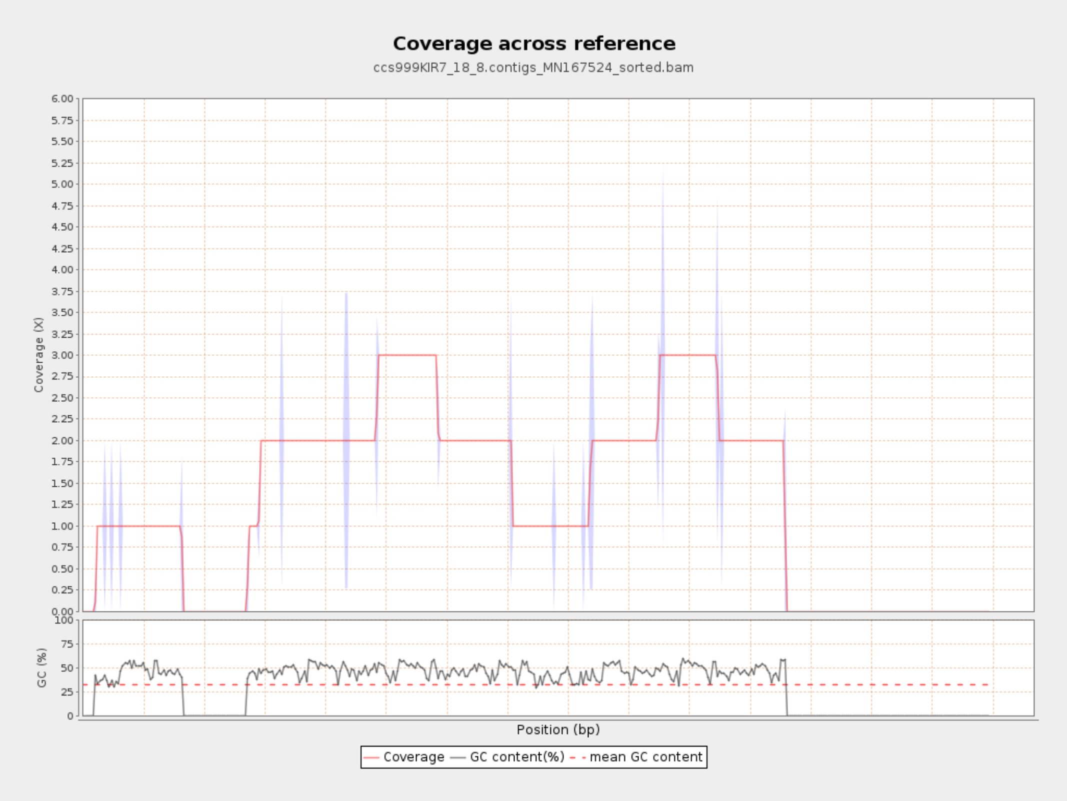

4. Results : Coverage Histogram

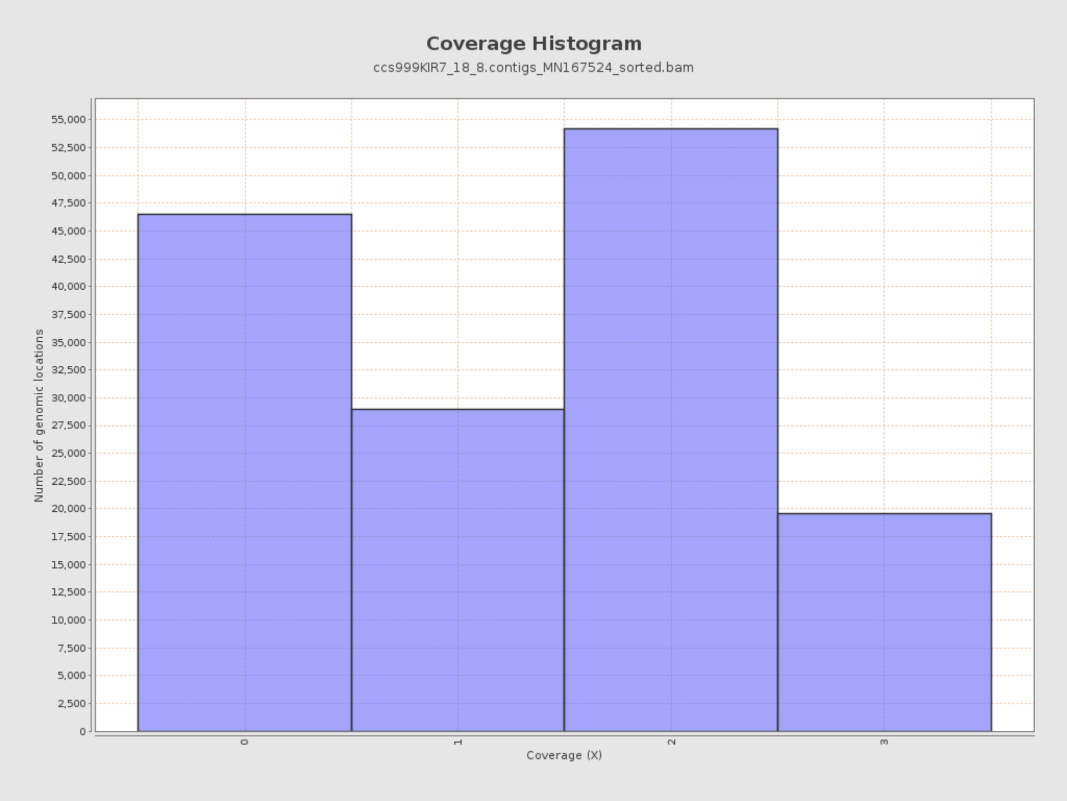

5. Results : Coverage Histogram (0-50X)

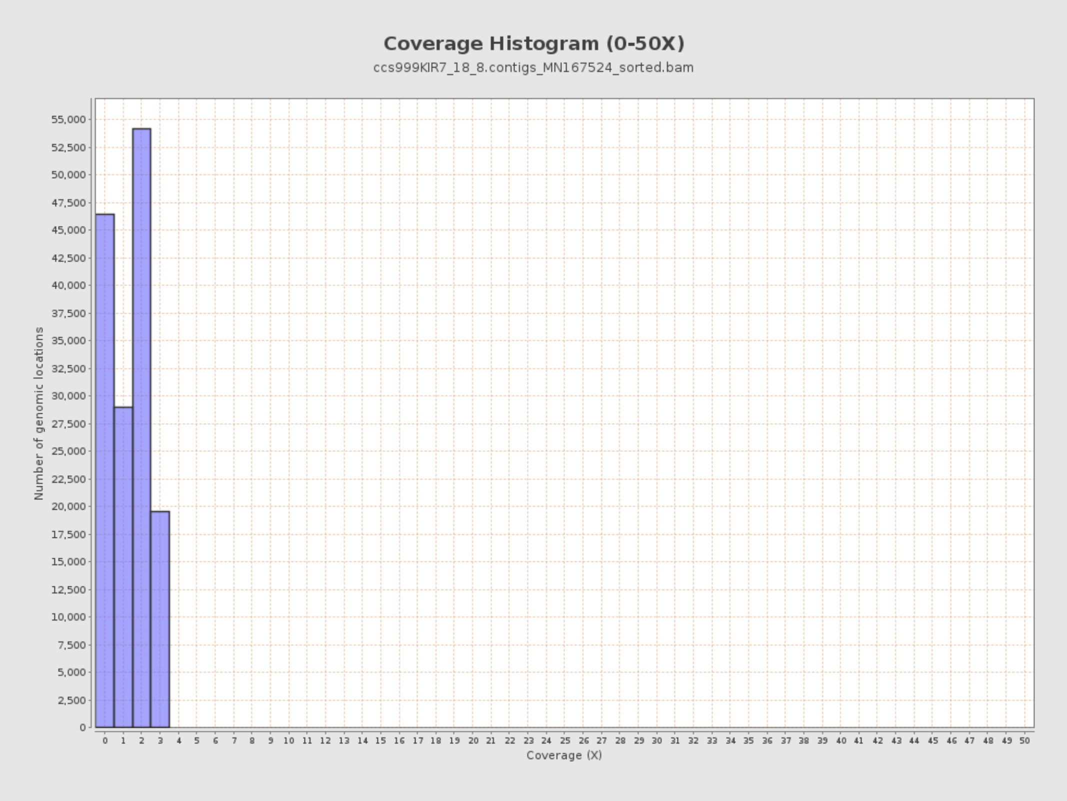

6. Results : Genome Fraction Coverage

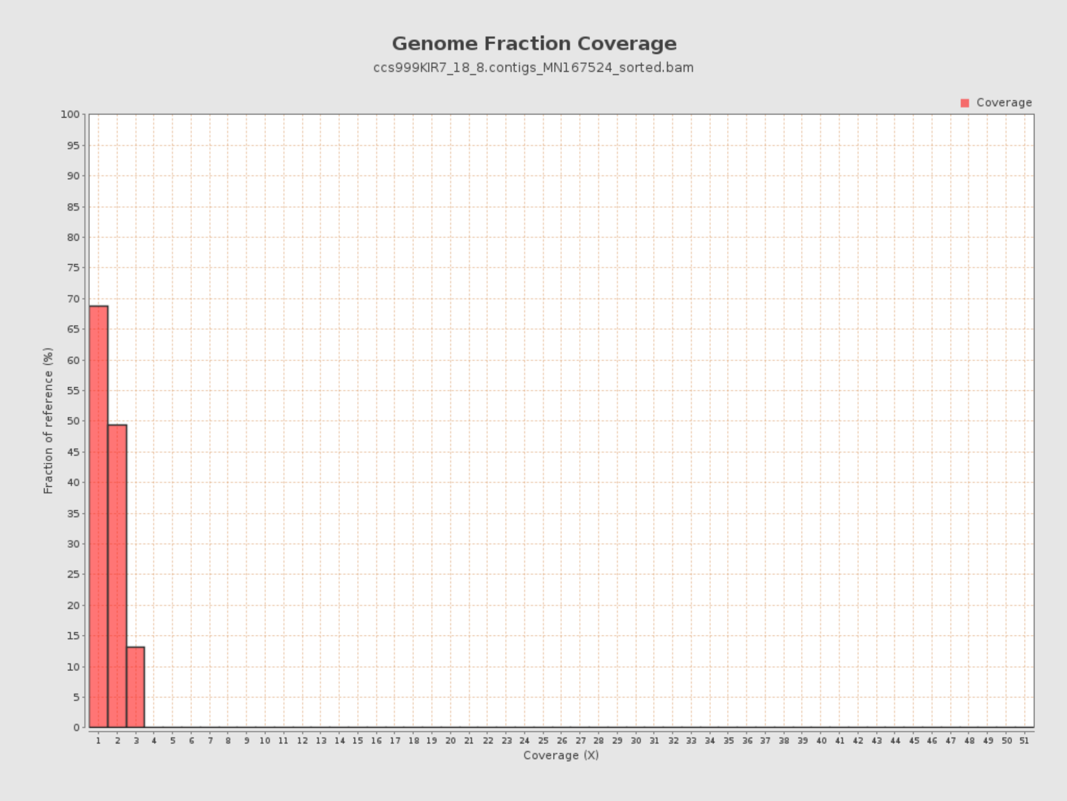

7. Results : Duplication Rate Histogram

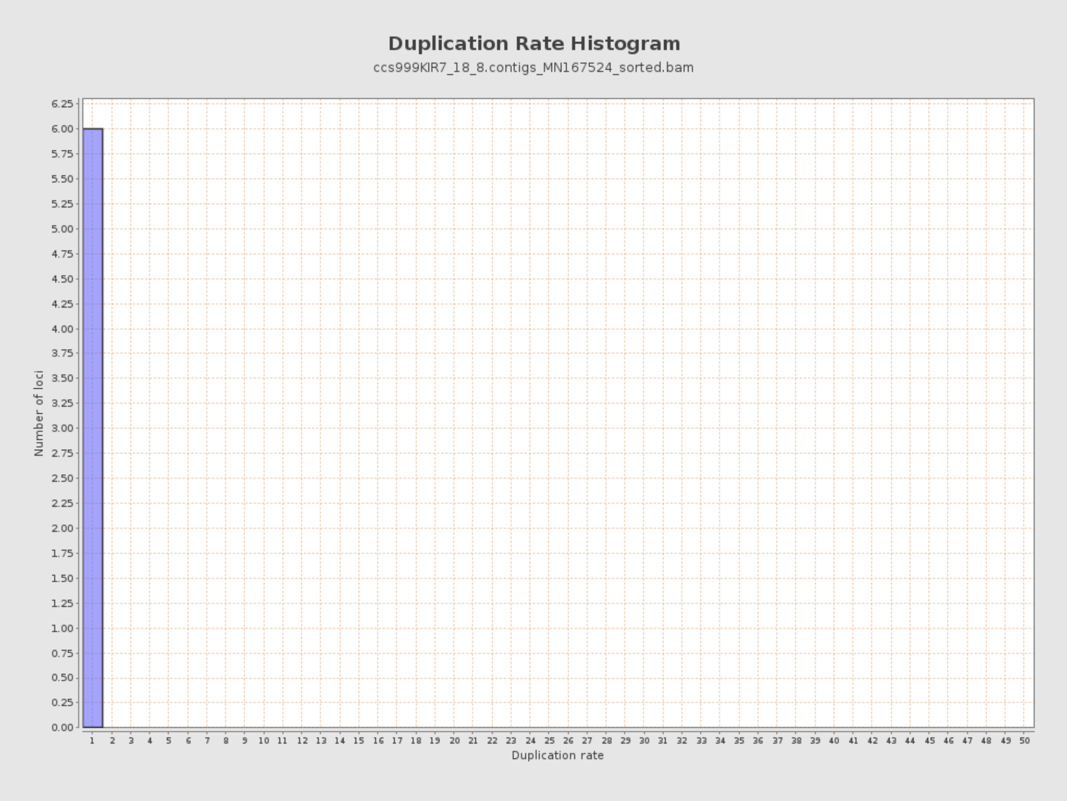

8. Results : Mapped Reads Nucleotide Content

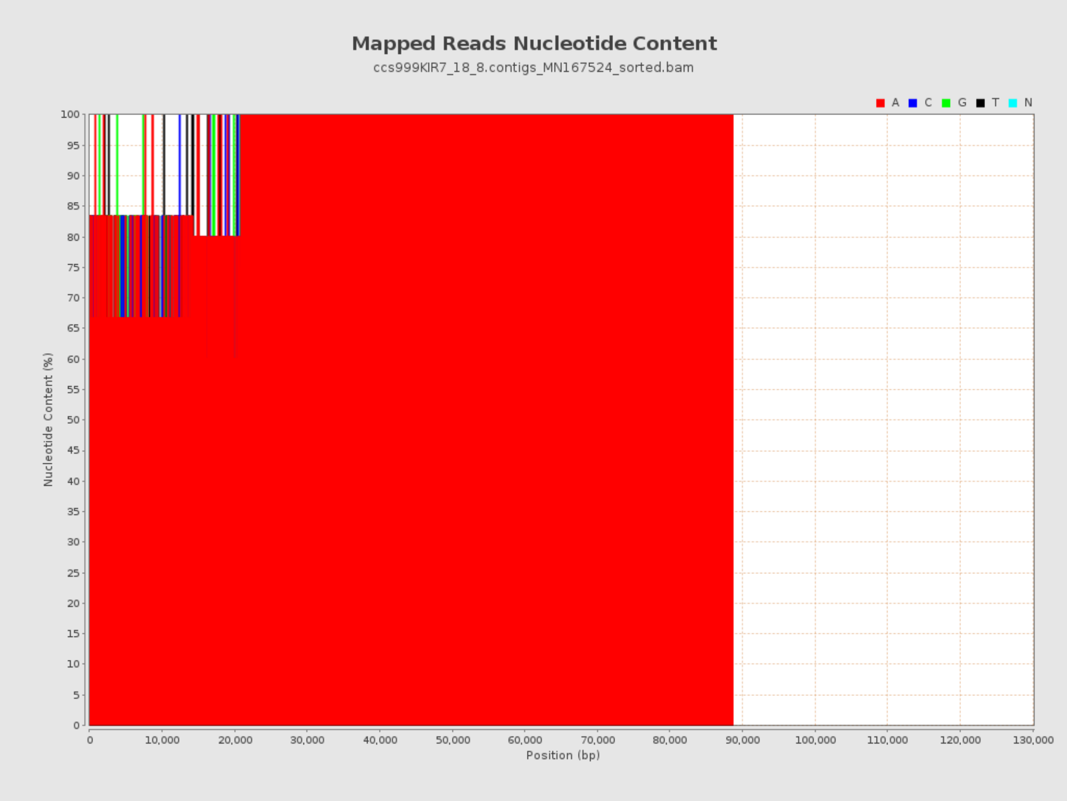

9. Results : Mapped Reads GC-content Distribution

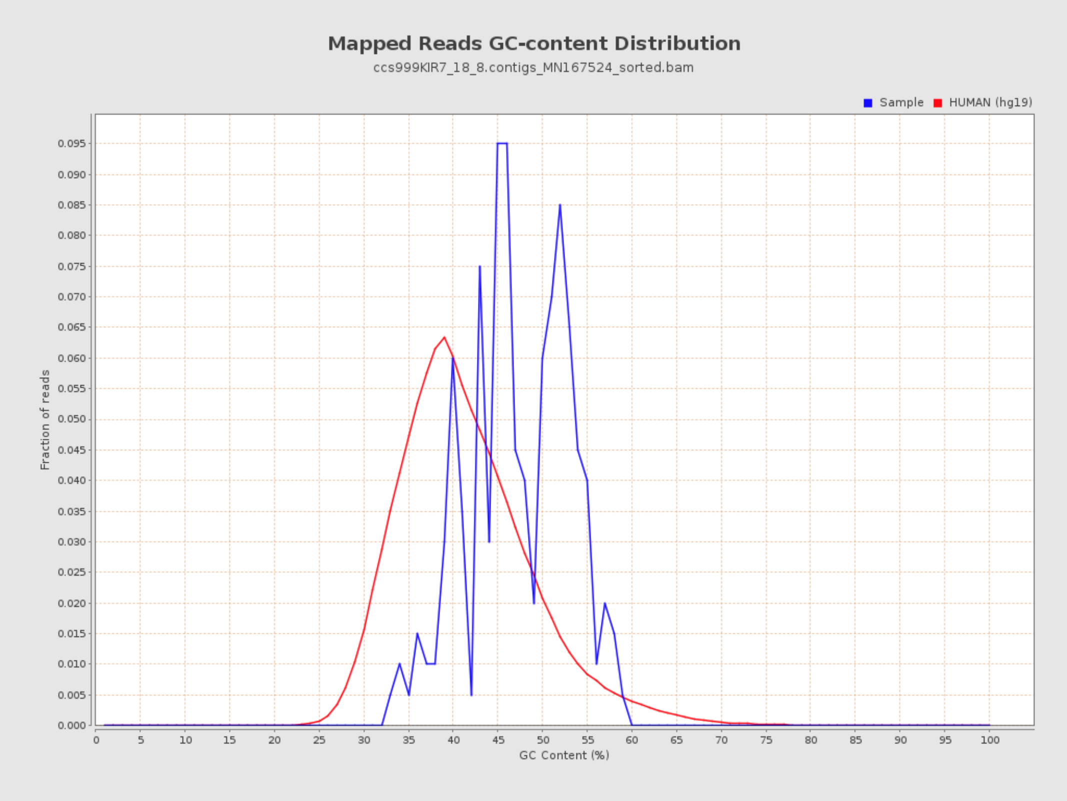

10. Results : Mapped Reads Clipping Profile

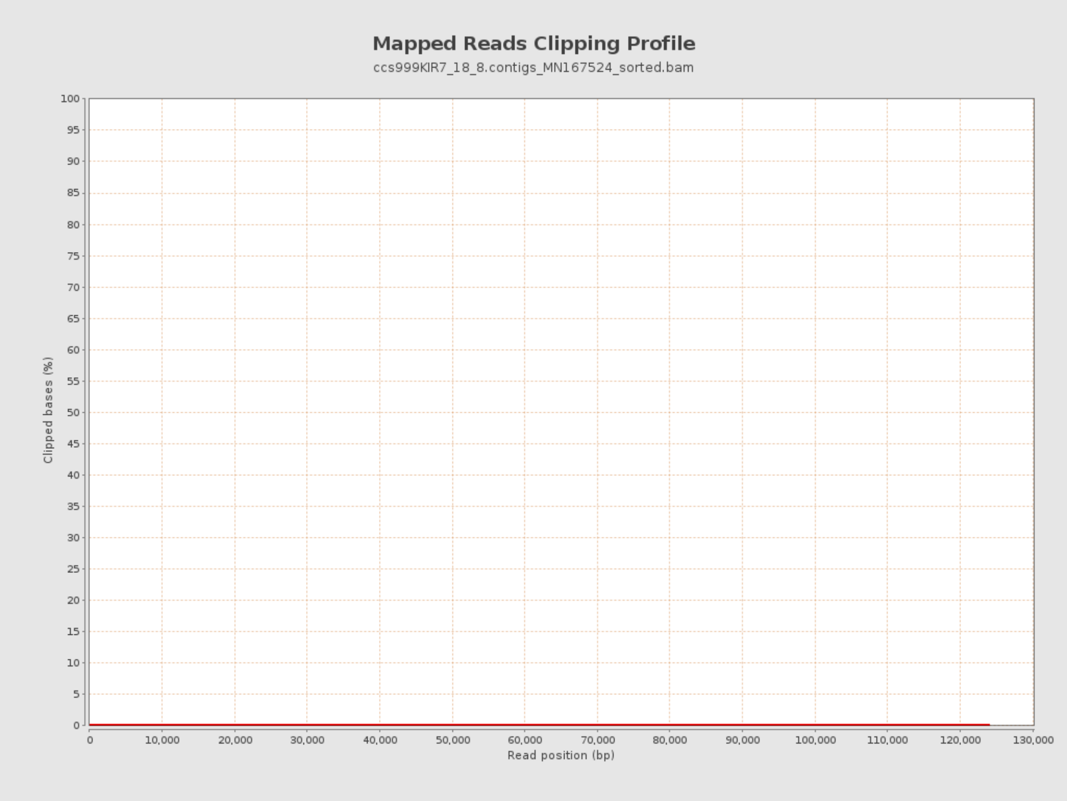

11. Results : Homopolymer Indels

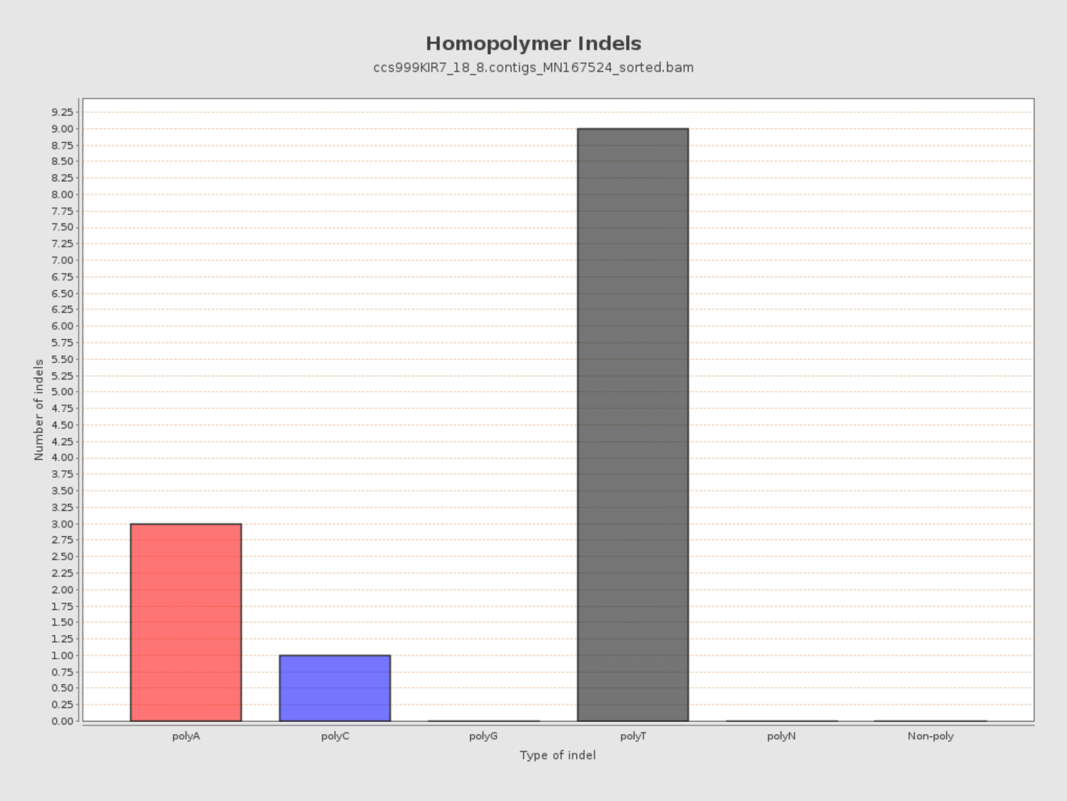

12. Results : Mapping Quality Across Reference

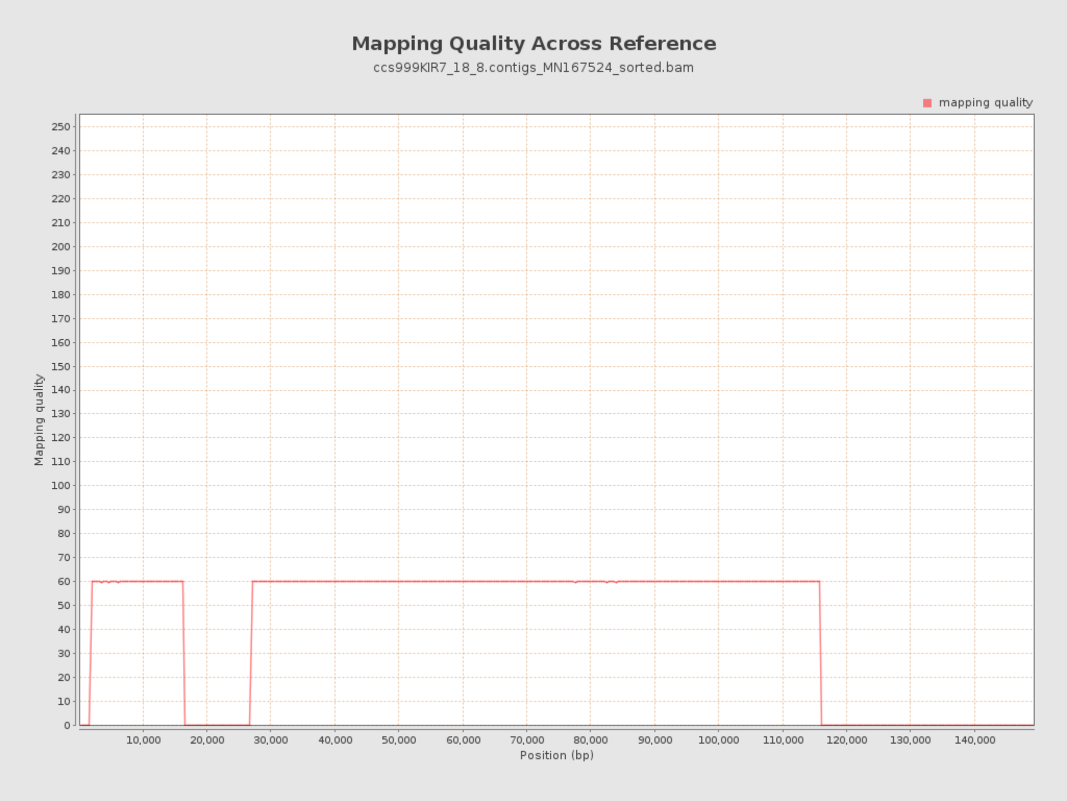

13. Results : Mapping Quality Histogram

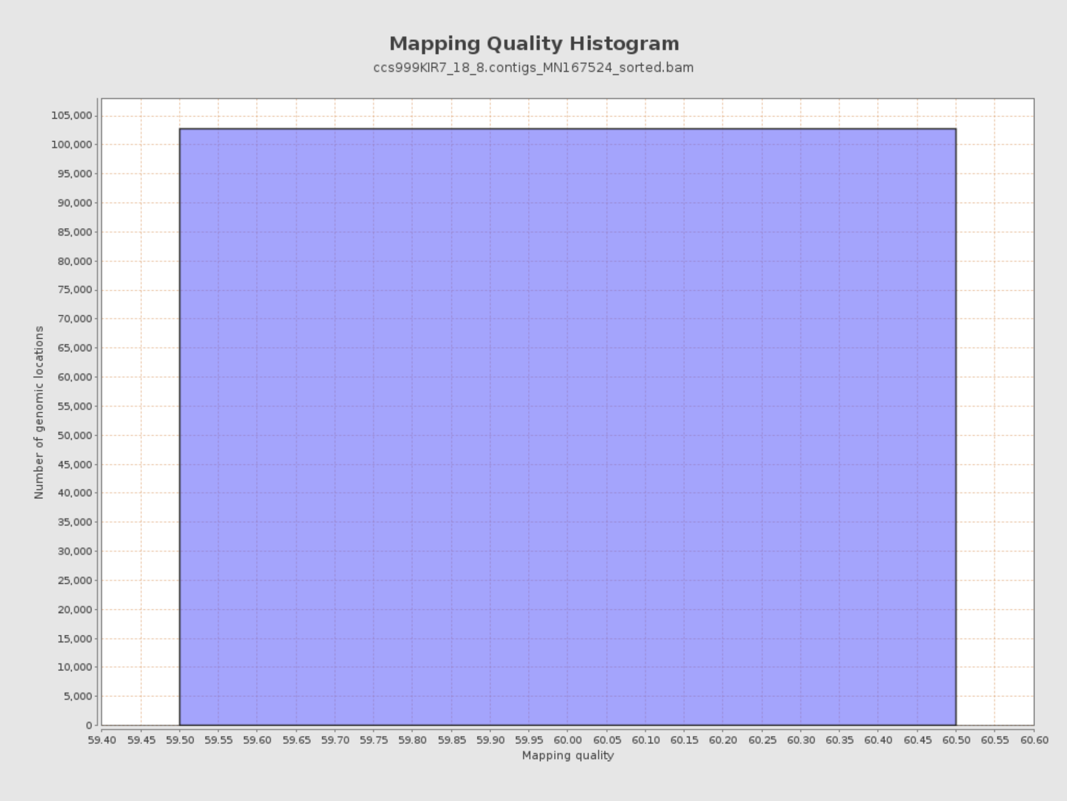

Supplement: Supplementary Figure 1 — AFA and EUR haplotigs. Data Sheets 2-5 are zip files containing the assembled haplotigs for all AFA and EUR assemblies. Also included are Qualimap, NanoPack, and QUAST reports. [file DataSheet_2.zip › SF1d/ccs999KIR7_18_8.contigs_MN167524_reports/ccs999KIR7_18_8.contigs_MN167524_qualimap.pdf]
