# Supplementary figures and images for: Efficient Sequencing, Assembly, and Annotation of Human KIR Haplotypes (part 1 of 2)
Source: Front Immunol. 2020 Oct 9;11:582927. doi: 10.3389/fimmu.2020.582927 (PMC7581912; doi:10.3389/fimmu.2020.582927)

Weighted Histogram of read lengths after log transformation

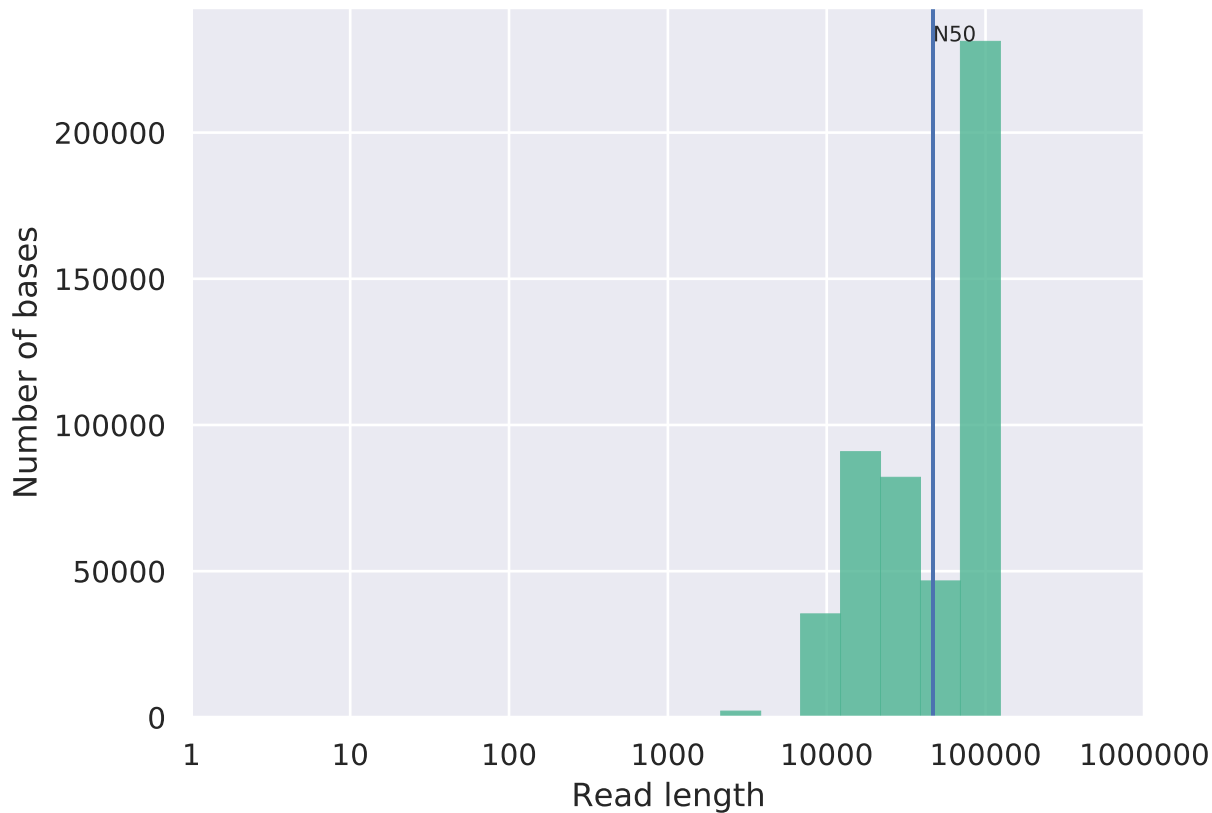

Supplement: Supplementary Figure 1 — AFA and EUR haplotigs. Data Sheets 2-5 are zip files containing the assembled haplotigs for all AFA and EUR assemblies. Also included are Qualimap, NanoPack, and QUAST reports. [file DataSheet_2.zip › SF1d/ccs999KIR7_18_8.contigs_MN167525_reports/ccs999KIR7_18_8.contigs_MN167525Weighted_LogTransformed_HistogramReadlength.pdf]

Weighted Histogram of read lengths

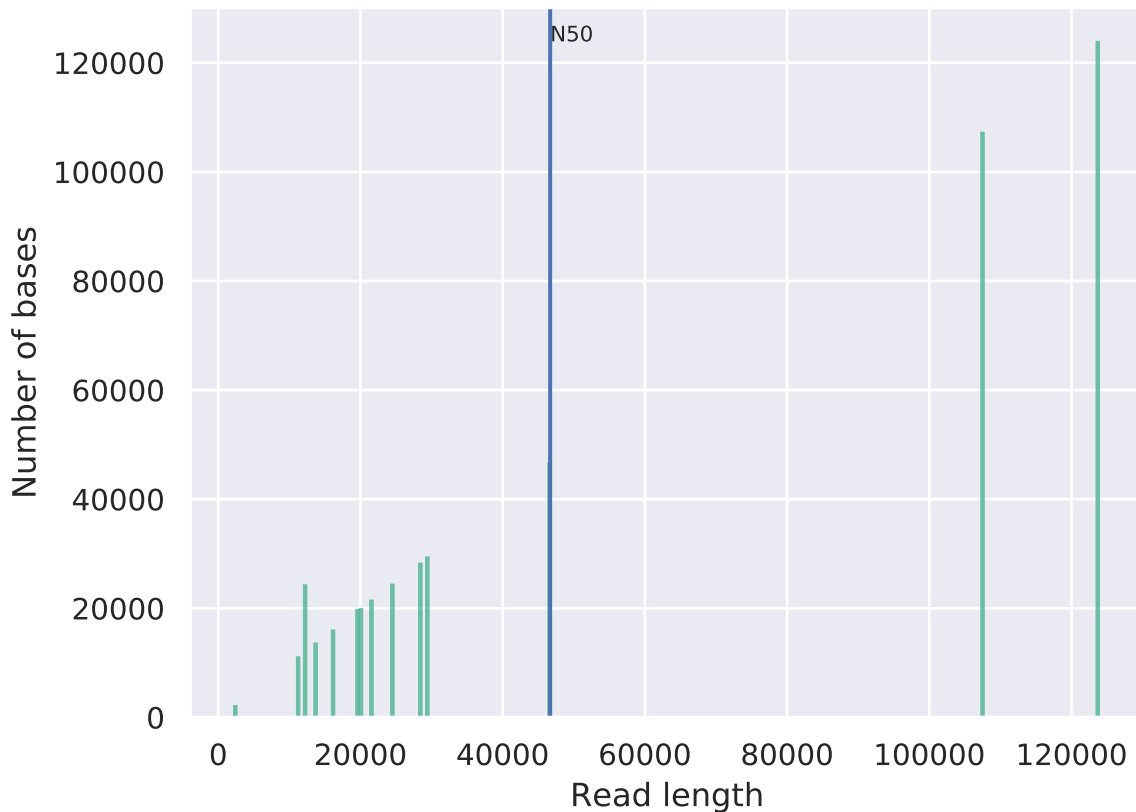

Supplement: Supplementary Figure 1 — AFA and EUR haplotigs. Data Sheets 2-5 are zip files containing the assembled haplotigs for all AFA and EUR assemblies. Also included are Qualimap, NanoPack, and QUAST reports. [file DataSheet_2.zip › SF1d/ccs999KIR7_18_8.contigs_MN167525_reports/ccs999KIR7_18_8.contigs_MN167525Weighted_HistogramReadlength.pdf]

# Aligned read length vs Percent identity plot

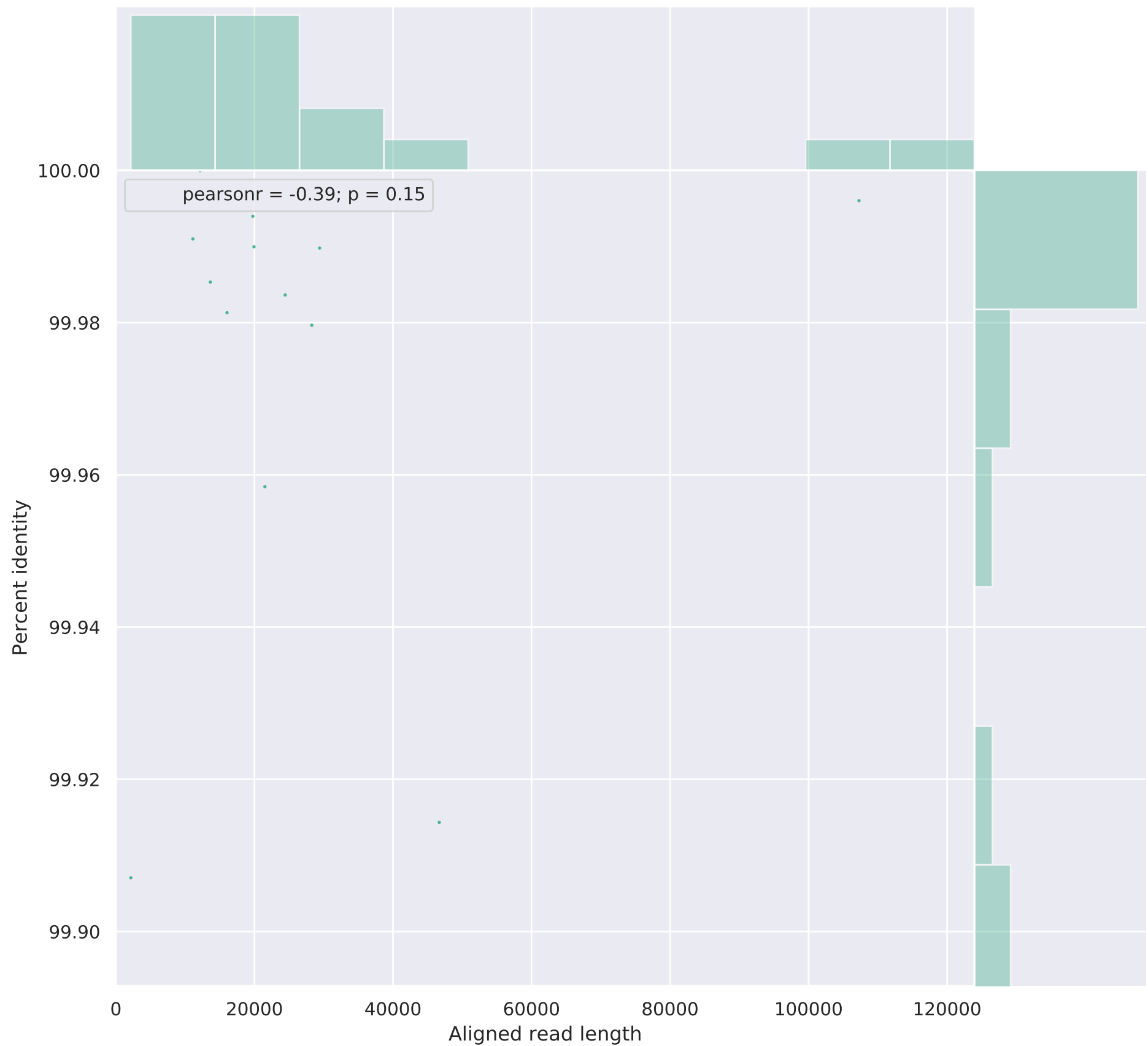

Supplement: Supplementary Figure 1 — AFA and EUR haplotigs. Data Sheets 2-5 are zip files containing the assembled haplotigs for all AFA and EUR assemblies. Also included are Qualimap, NanoPack, and QUAST reports. [file DataSheet_2.zip › SF1d/ccs999KIR7_18_8.contigs_MN167525_reports/ccs999KIR7_18_8.contigs_MN167525PercentIdentityvsAlignedReadLength_dot.pdf]

Yield by length

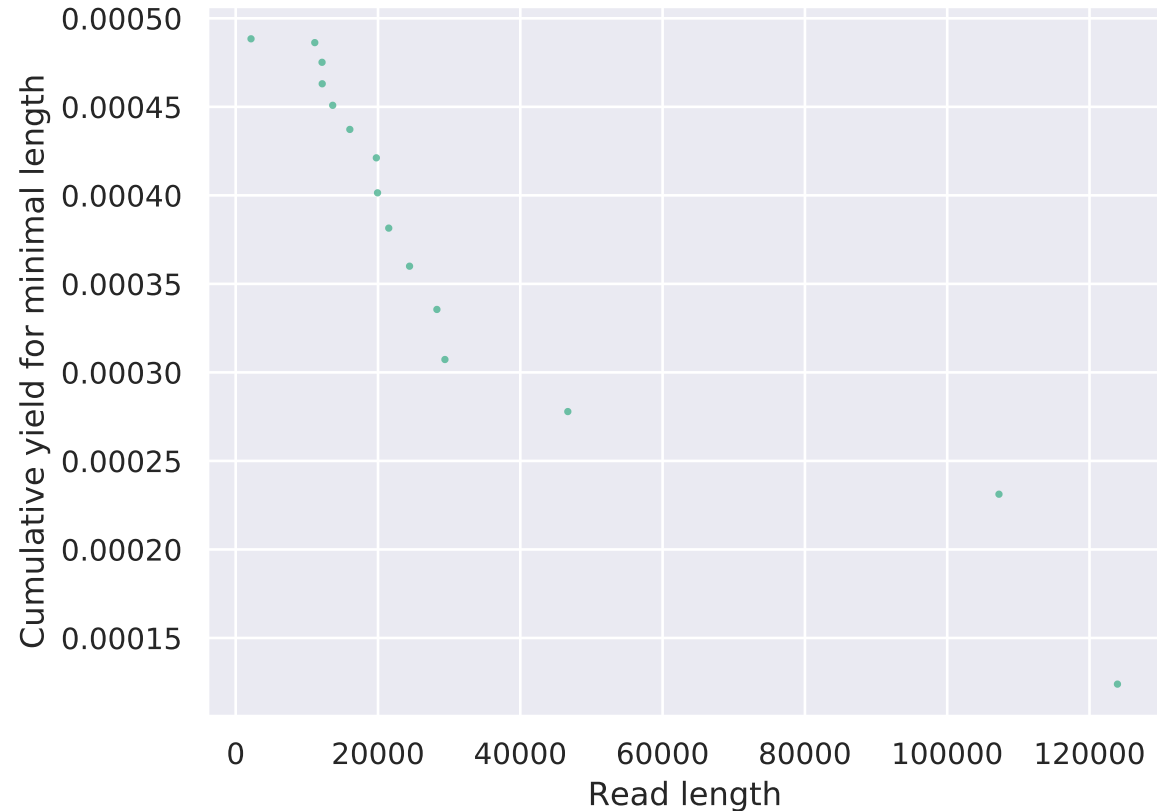

Supplement: Supplementary Figure 1 — AFA and EUR haplotigs. Data Sheets 2-5 are zip files containing the assembled haplotigs for all AFA and EUR assemblies. Also included are Qualimap, NanoPack, and QUAST reports. [file DataSheet_2.zip › SF1d/ccs999KIR7_18_8.contigs_MN167525_reports/ccs999KIR7_18_8.contigs_MN167525Yield_By_Length.pdf]

# Aligned read lengths vs Sequenced read length plot

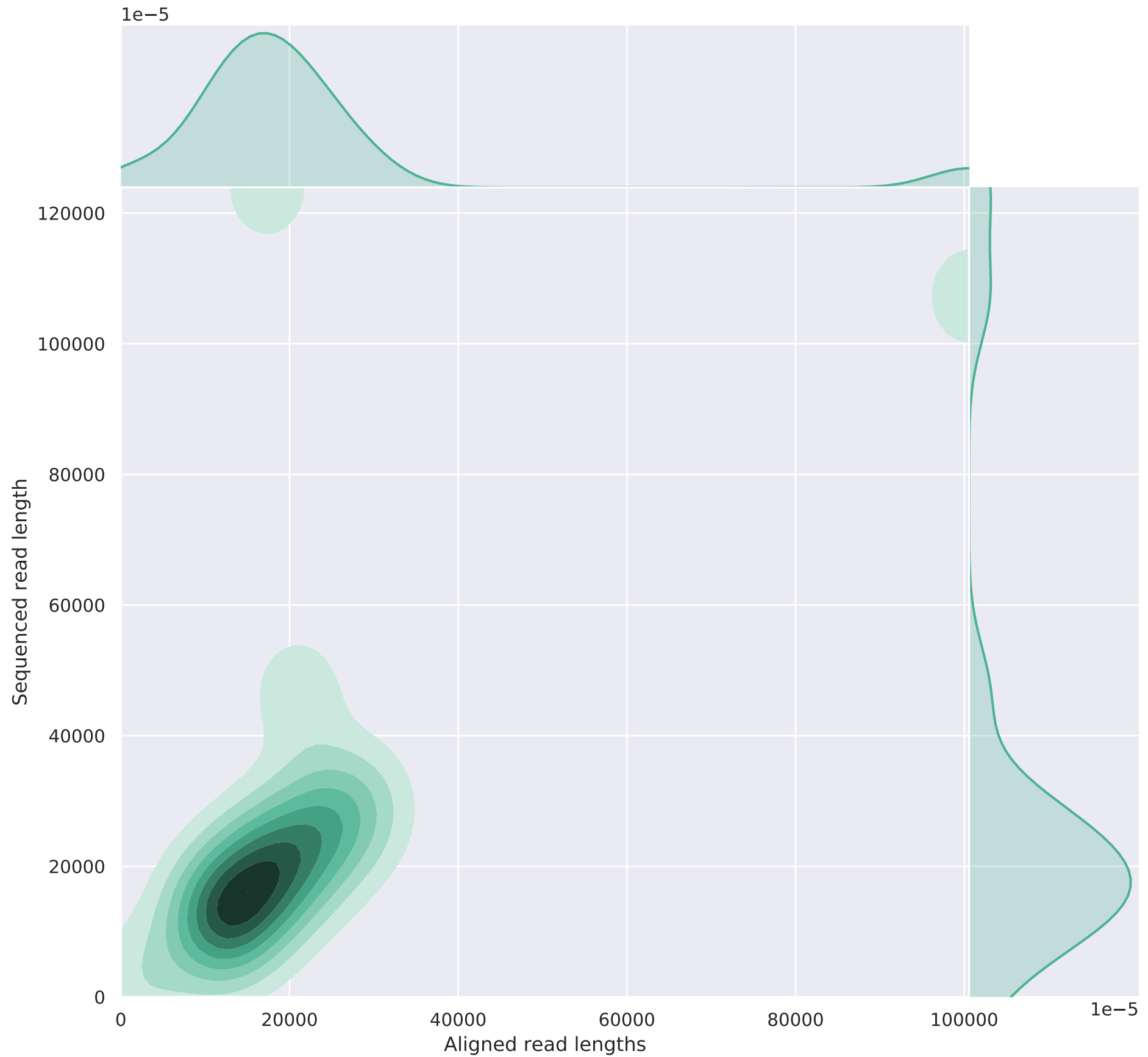

Supplement: Supplementary Figure 1 — AFA and EUR haplotigs. Data Sheets 2-5 are zip files containing the assembled haplotigs for all AFA and EUR assemblies. Also included are Qualimap, NanoPack, and QUAST reports. [file DataSheet_2.zip › SF1d/ccs999KIR7_18_8.contigs_MN167525_reports/ccs999KIR7_18_8.contigs_MN167525AlignedReadlengthvsSequencedReadLength_kde.pdf]

Histogram of read lengths after log transformation

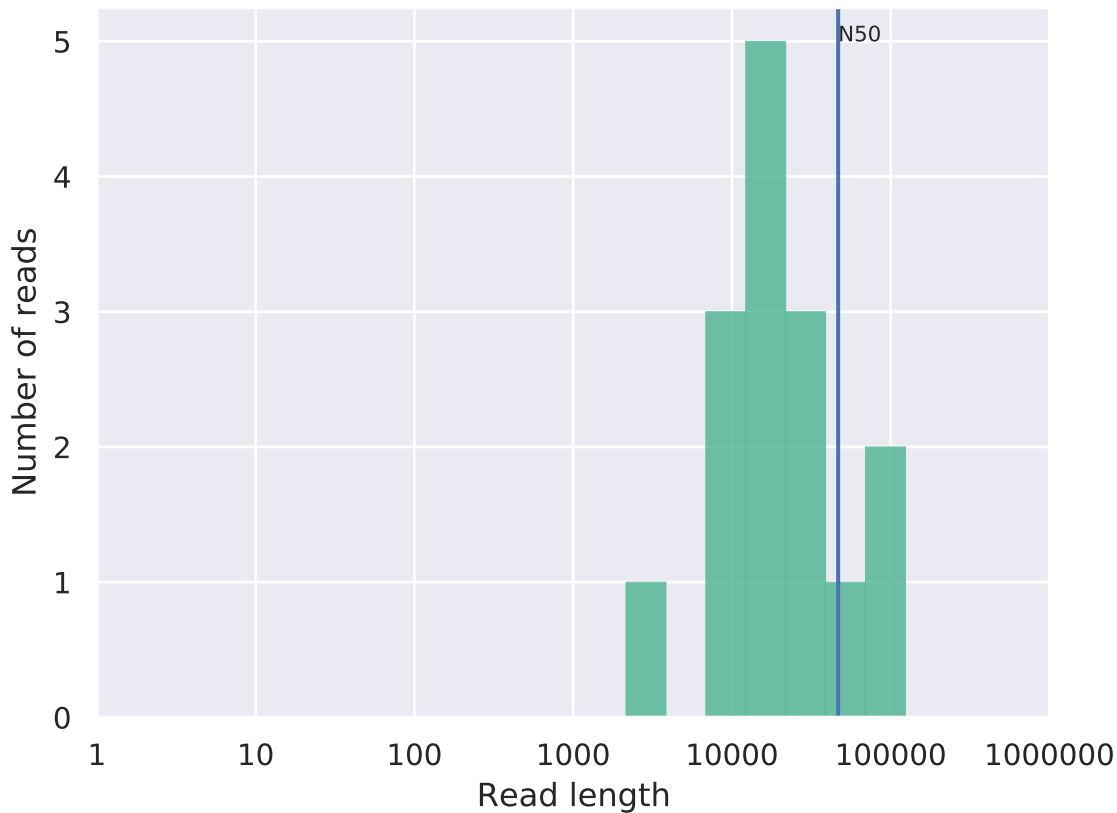

Supplement: Supplementary Figure 1 — AFA and EUR haplotigs. Data Sheets 2-5 are zip files containing the assembled haplotigs for all AFA and EUR assemblies. Also included are Qualimap, NanoPack, and QUAST reports. [file DataSheet_2.zip › SF1d/ccs999KIR7_18_8.contigs_MN167525_reports/ccs999KIR7_18_8.contigs_MN167525LogTransformed_HistogramReadlength.pdf]

# Aligned read length vs Percent identity plot

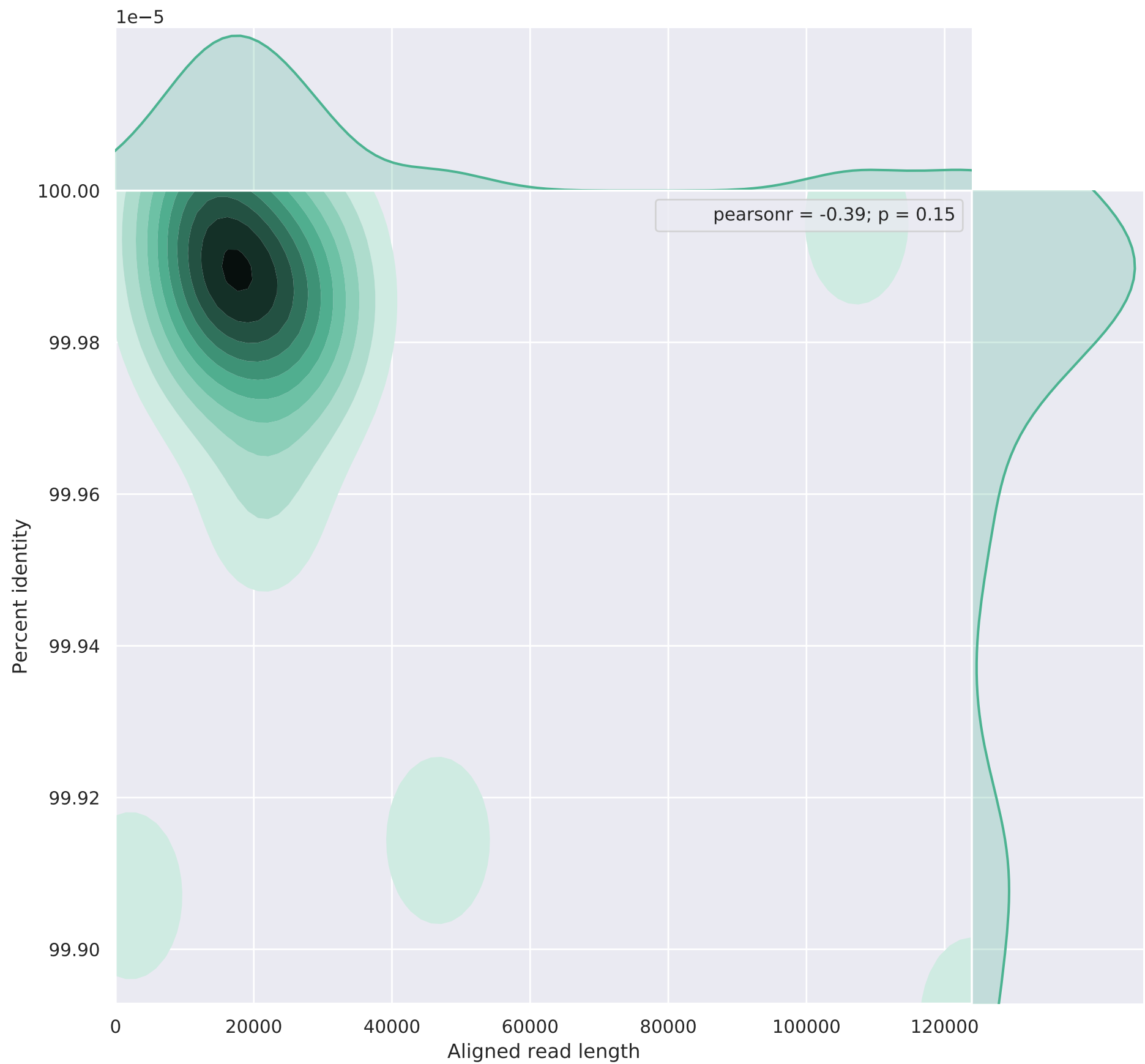

Supplement: Supplementary Figure 1 — AFA and EUR haplotigs. Data Sheets 2-5 are zip files containing the assembled haplotigs for all AFA and EUR assemblies. Also included are Qualimap, NanoPack, and QUAST reports. [file DataSheet_2.zip › SF1d/ccs999KIR7_18_8.contigs_MN167525_reports/ccs999KIR7_18_8.contigs_MN167525PercentIdentityvsAlignedReadLength_kde.pdf]

# Aligned read lengths vs Sequenced read length plot

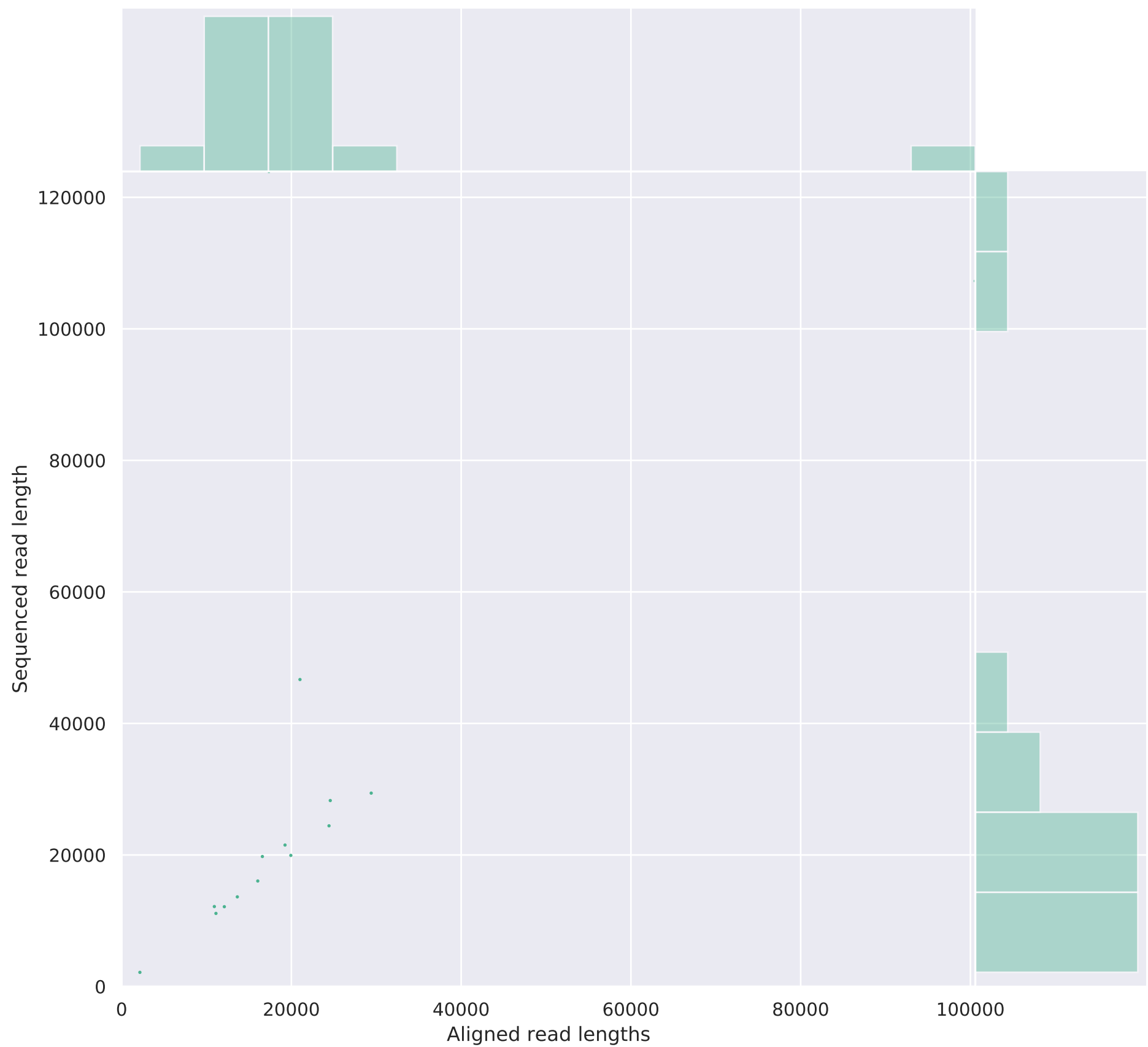

Supplement: Supplementary Figure 1 — AFA and EUR haplotigs. Data Sheets 2-5 are zip files containing the assembled haplotigs for all AFA and EUR assemblies. Also included are Qualimap, NanoPack, and QUAST reports. [file DataSheet_2.zip › SF1d/ccs999KIR7_18_8.contigs_MN167525_reports/ccs999KIR7_18_8.contigs_MN167525AlignedReadlengthvsSequencedReadLength_dot.pdf]

Histogram of read lengths

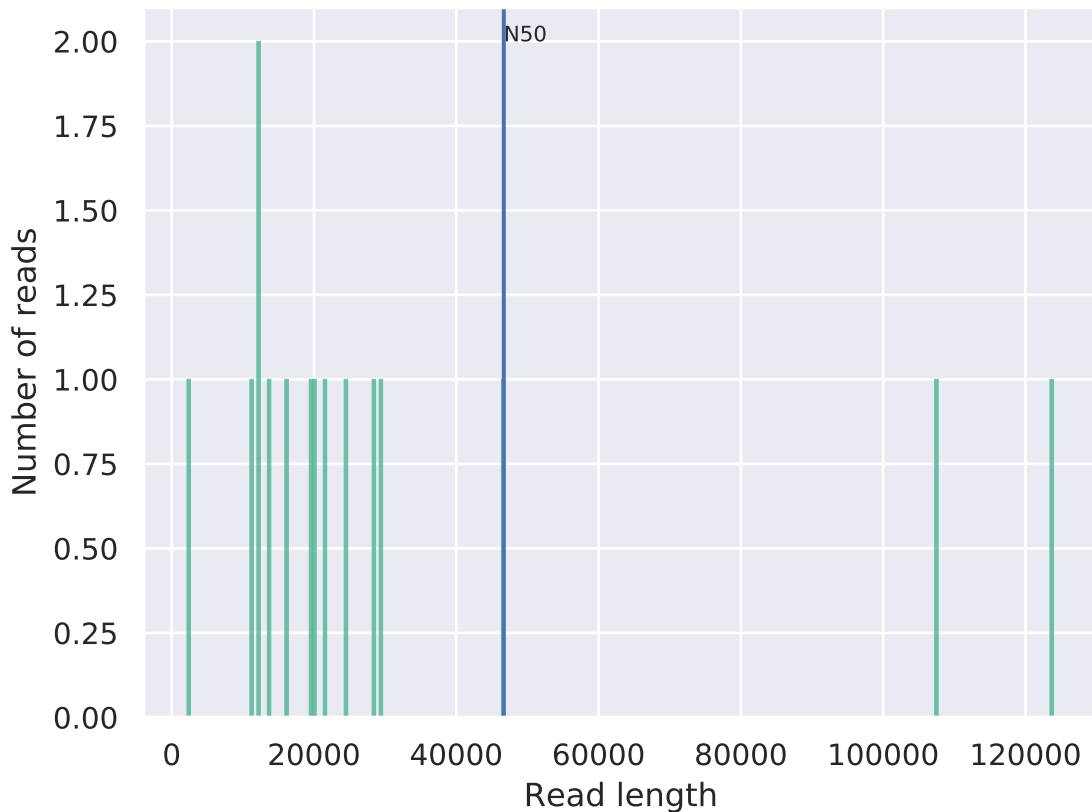

Supplement: Supplementary Figure 1 — AFA and EUR haplotigs. Data Sheets 2-5 are zip files containing the assembled haplotigs for all AFA and EUR assemblies. Also included are Qualimap, NanoPack, and QUAST reports. [file DataSheet_2.zip › SF1d/ccs999KIR7_18_8.contigs_MN167525_reports/ccs999KIR7_18_8.contigs_MN167525HistogramReadlength.pdf]

# Aligned read length vs Percent identity plot

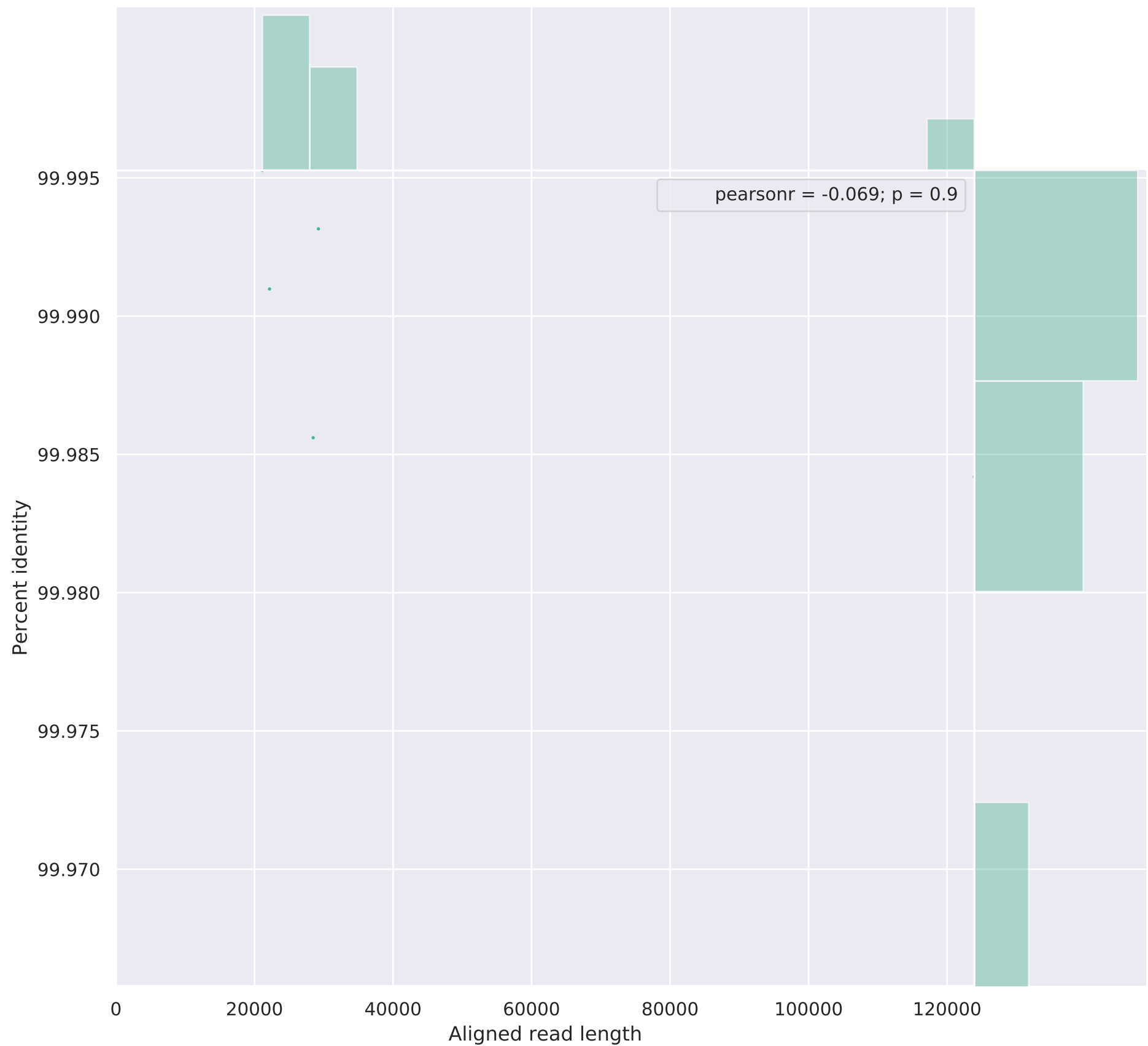

Supplement: Supplementary Figure 1 — AFA and EUR haplotigs. Data Sheets 2-5 are zip files containing the assembled haplotigs for all AFA and EUR assemblies. Also included are Qualimap, NanoPack, and QUAST reports. [file DataSheet_2.zip › SF1d/ccs999KIR7_18_8.contigs_MN167524_reports/ccs999KIR7_18_8.contigs_MN167524PercentIdentityvsAlignedReadLength_dot.pdf]

# Aligned read lengths vs Sequenced read length plot

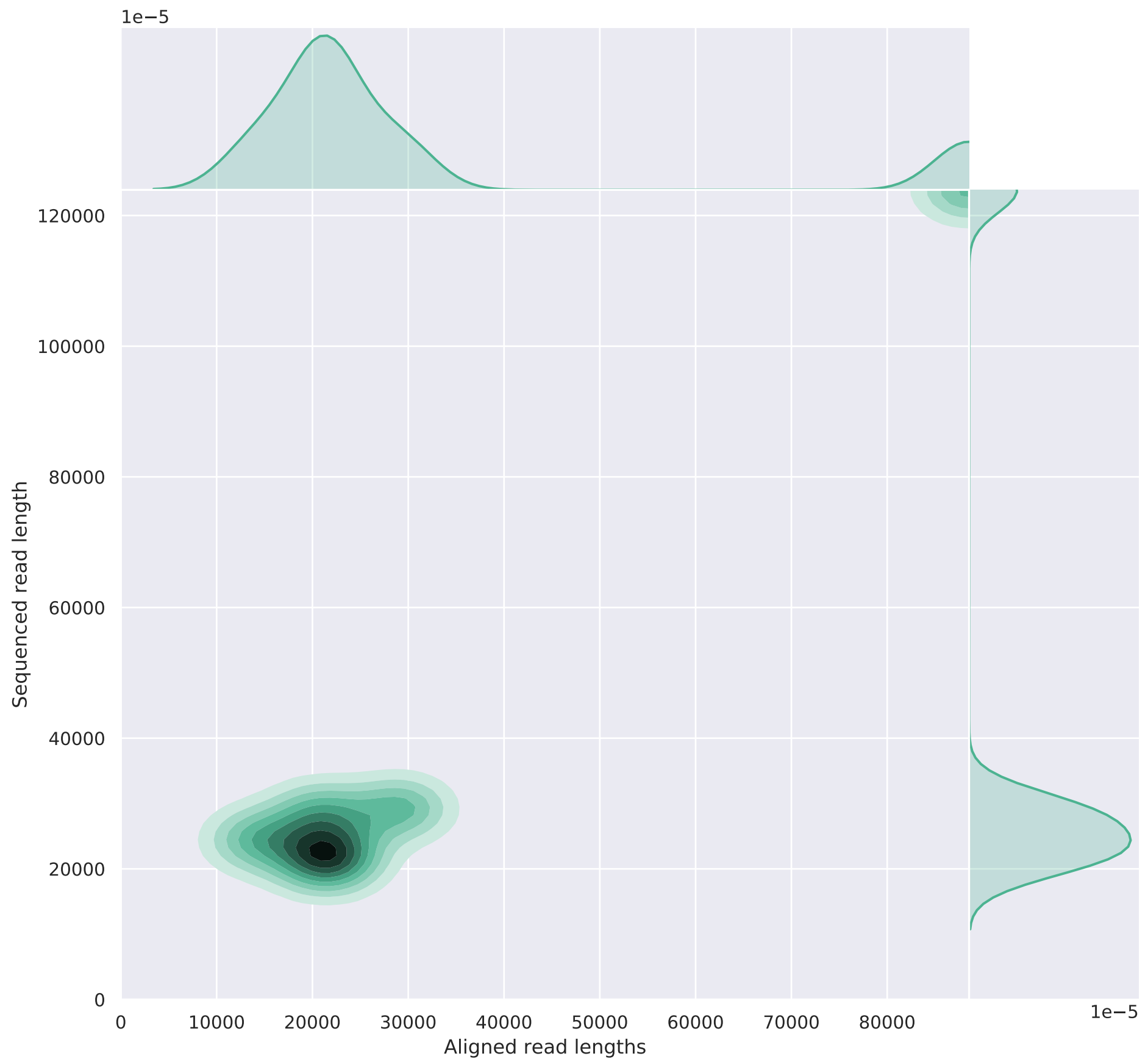

Supplement: Supplementary Figure 1 — AFA and EUR haplotigs. Data Sheets 2-5 are zip files containing the assembled haplotigs for all AFA and EUR assemblies. Also included are Qualimap, NanoPack, and QUAST reports. [file DataSheet_2.zip › SF1d/ccs999KIR7_18_8.contigs_MN167524_reports/ccs999KIR7_18_8.contigs_MN167524AlignedReadlengthvsSequencedReadLength_kde.pdf]

Histogram of read lengths

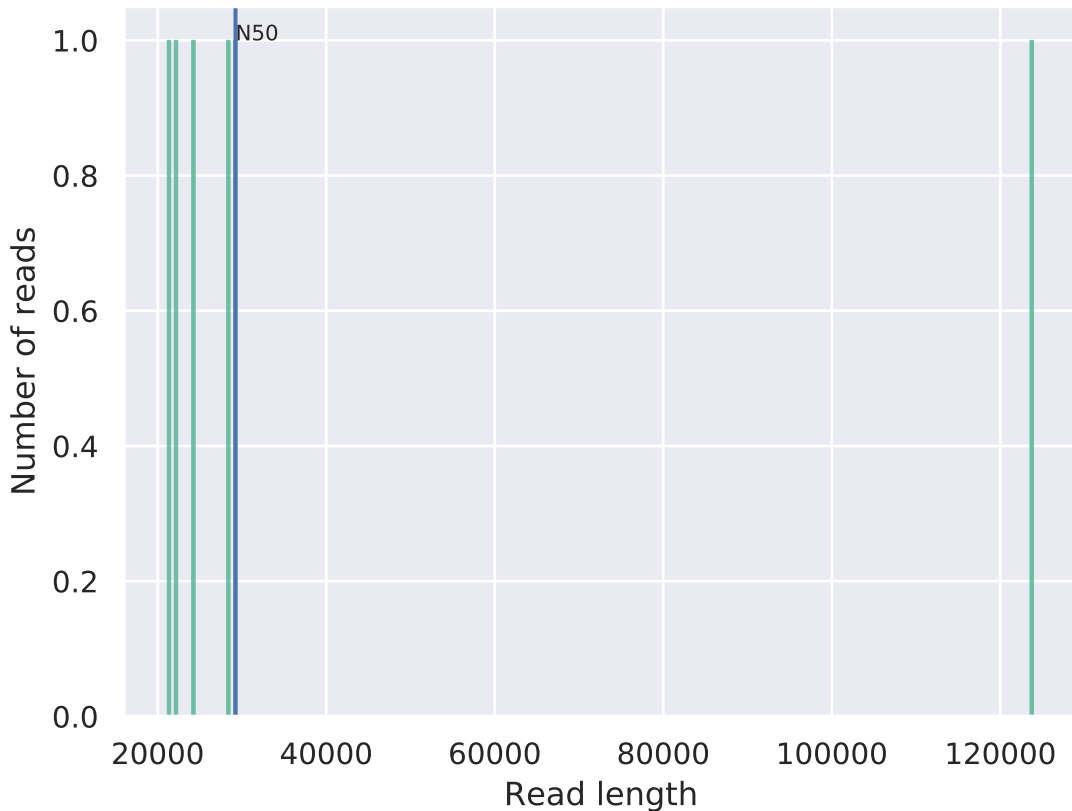

Supplement: Supplementary Figure 1 — AFA and EUR haplotigs. Data Sheets 2-5 are zip files containing the assembled haplotigs for all AFA and EUR assemblies. Also included are Qualimap, NanoPack, and QUAST reports. [file DataSheet_2.zip › SF1d/ccs999KIR7_18_8.contigs_MN167524_reports/ccs999KIR7_18_8.contigs_MN167524HistogramReadlength.pdf]

# Aligned read lengths vs Sequenced read length plot

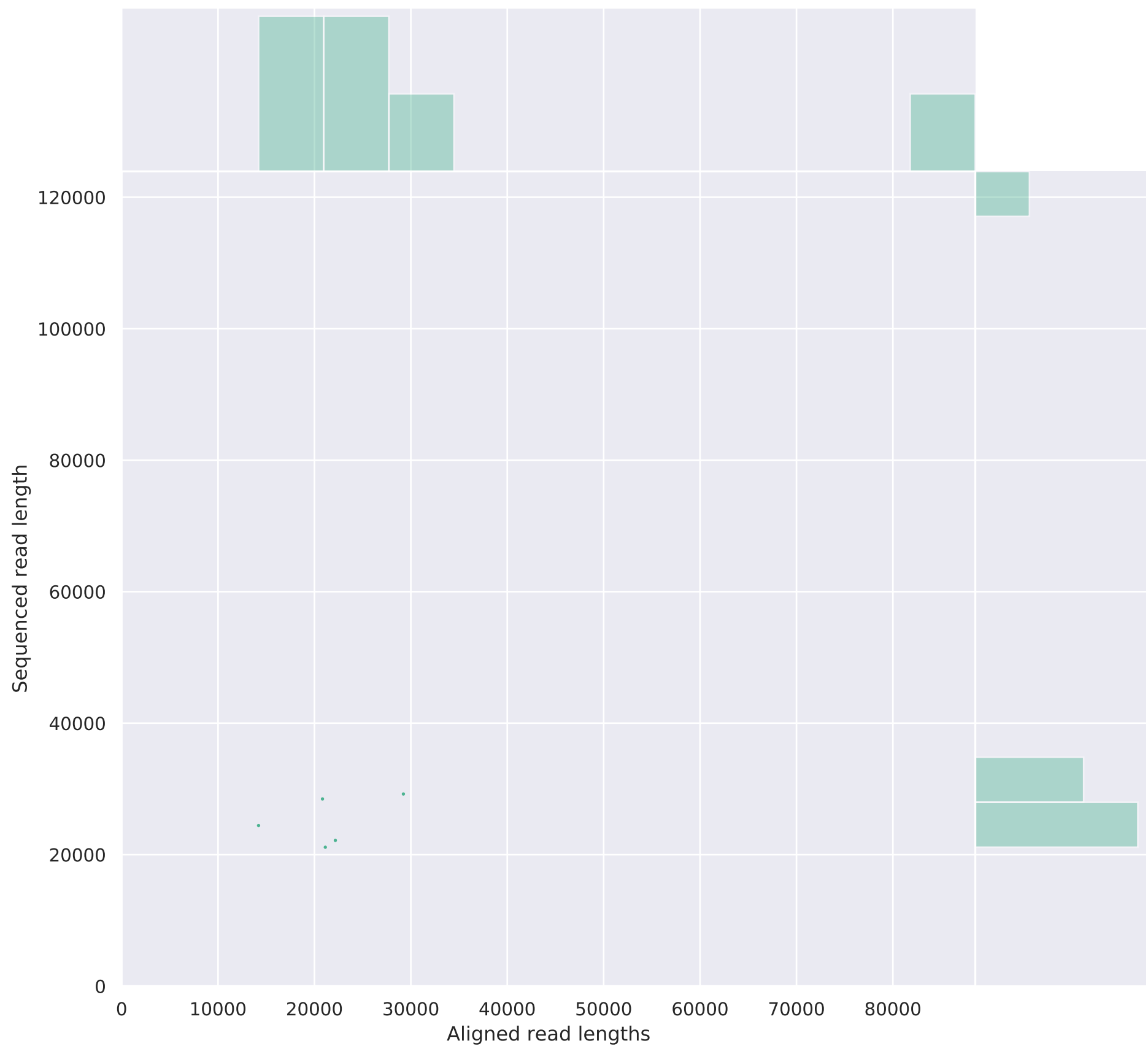

Supplement: Supplementary Figure 1 — AFA and EUR haplotigs. Data Sheets 2-5 are zip files containing the assembled haplotigs for all AFA and EUR assemblies. Also included are Qualimap, NanoPack, and QUAST reports. [file DataSheet_2.zip › SF1d/ccs999KIR7_18_8.contigs_MN167524_reports/ccs999KIR7_18_8.contigs_MN167524AlignedReadlengthvsSequencedReadLength_dot.pdf]

Histogram of read lengths after log transformation

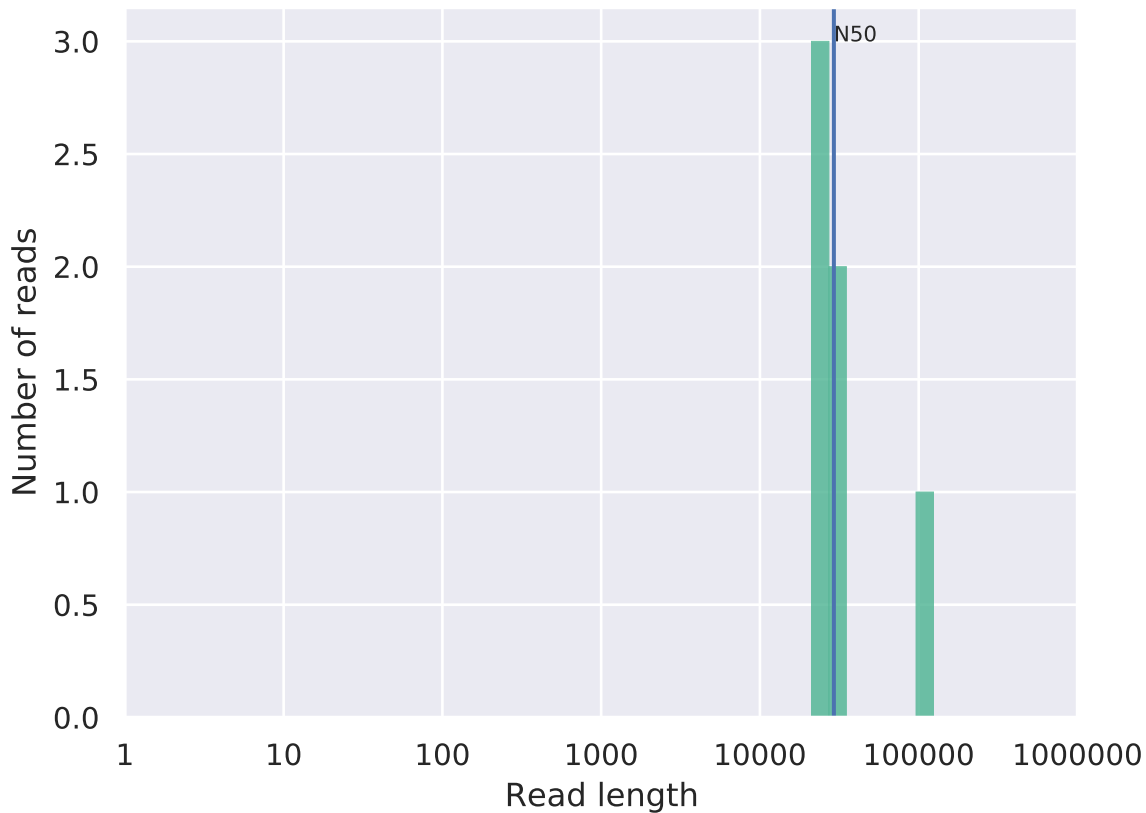

Supplement: Supplementary Figure 1 — AFA and EUR haplotigs. Data Sheets 2-5 are zip files containing the assembled haplotigs for all AFA and EUR assemblies. Also included are Qualimap, NanoPack, and QUAST reports. [file DataSheet_2.zip › SF1d/ccs999KIR7_18_8.contigs_MN167524_reports/ccs999KIR7_18_8.contigs_MN167524LogTransformed_HistogramReadlength.pdf]

Yield by length

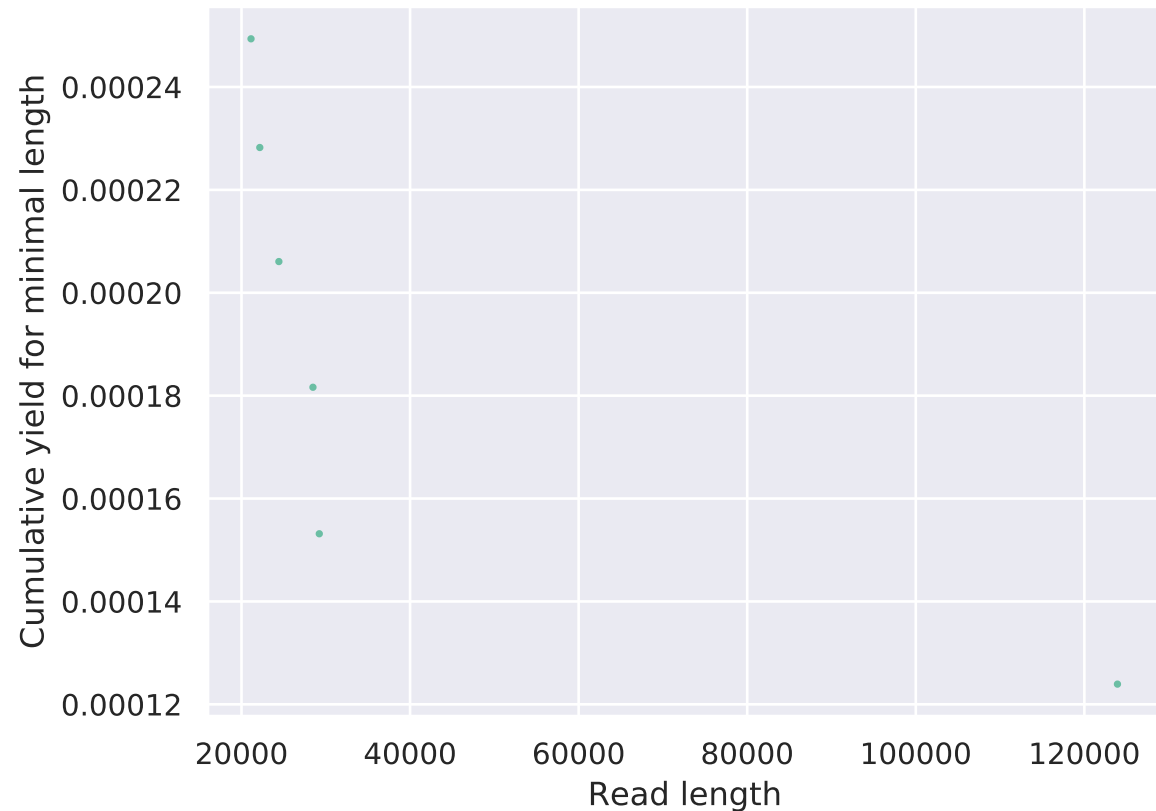

Supplement: Supplementary Figure 1 — AFA and EUR haplotigs. Data Sheets 2-5 are zip files containing the assembled haplotigs for all AFA and EUR assemblies. Also included are Qualimap, NanoPack, and QUAST reports. [file DataSheet_2.zip › SF1d/ccs999KIR7_18_8.contigs_MN167524_reports/ccs999KIR7_18_8.contigs_MN167524Yield_By_Length.pdf]

# Aligned read length vs Percent identity plot

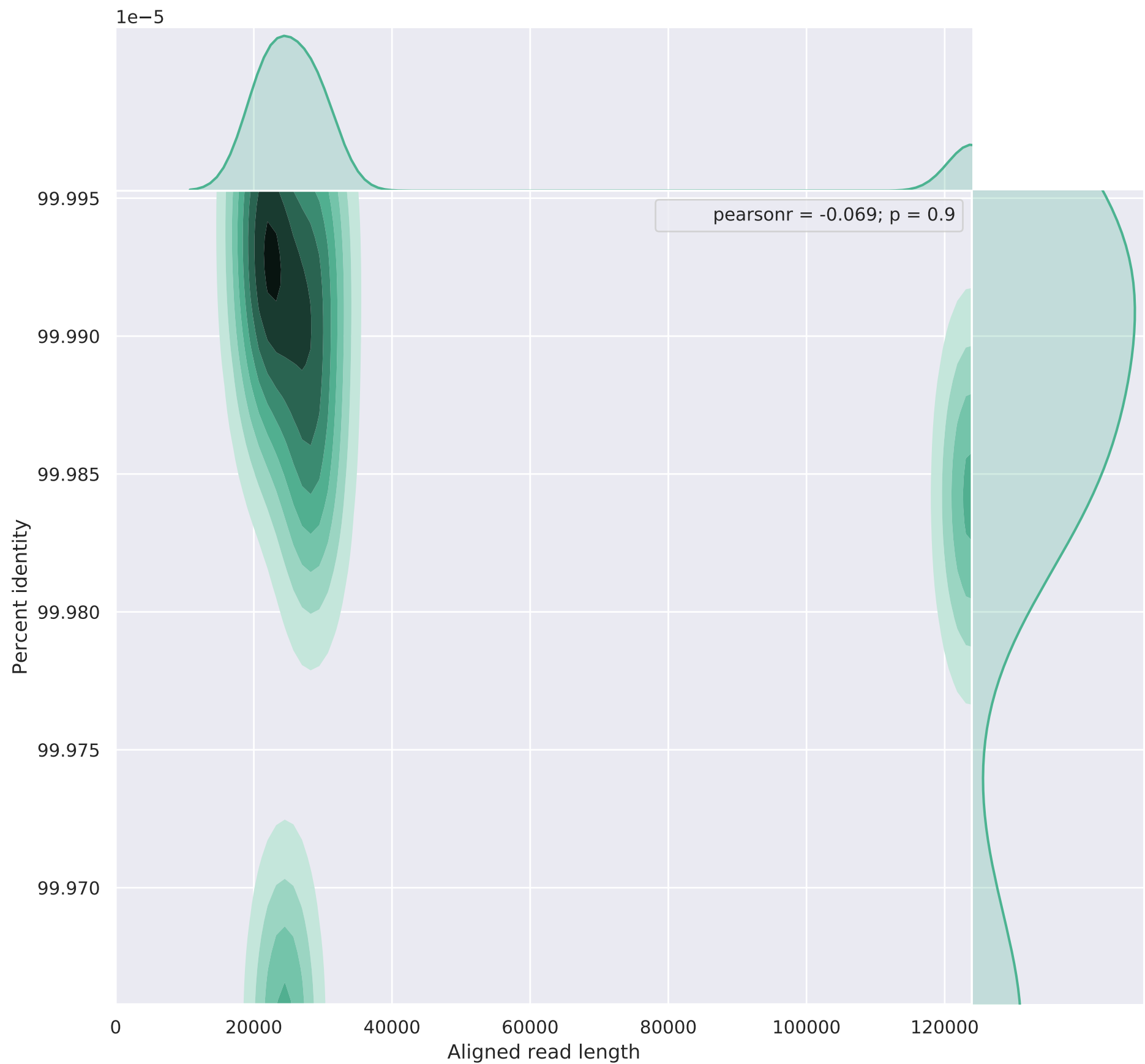

Supplement: Supplementary Figure 1 — AFA and EUR haplotigs. Data Sheets 2-5 are zip files containing the assembled haplotigs for all AFA and EUR assemblies. Also included are Qualimap, NanoPack, and QUAST reports. [file DataSheet_2.zip › SF1d/ccs999KIR7_18_8.contigs_MN167524_reports/ccs999KIR7_18_8.contigs_MN167524PercentIdentityvsAlignedReadLength_kde.pdf]

Weighted Histogram of read lengths after log transformation

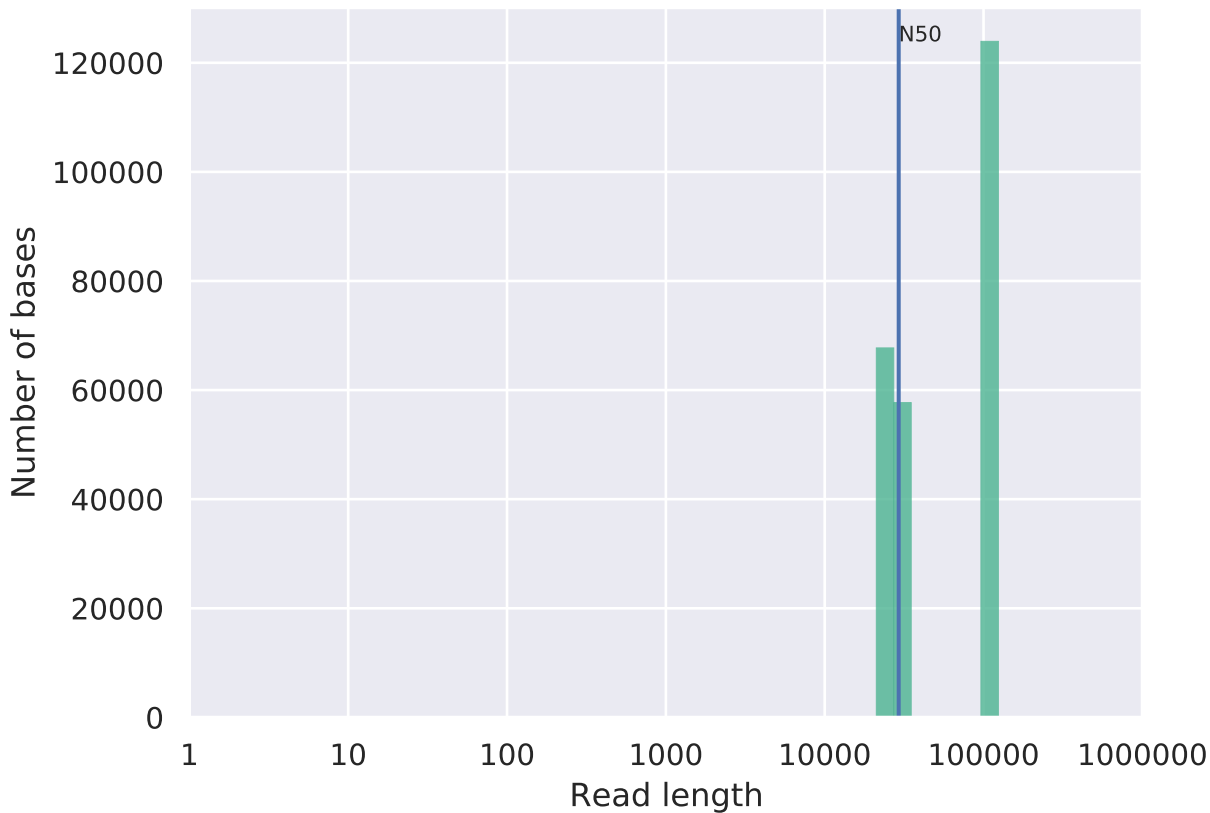

Supplement: Supplementary Figure 1 — AFA and EUR haplotigs. Data Sheets 2-5 are zip files containing the assembled haplotigs for all AFA and EUR assemblies. Also included are Qualimap, NanoPack, and QUAST reports. [file DataSheet_2.zip › SF1d/ccs999KIR7_18_8.contigs_MN167524_reports/ccs999KIR7_18_8.contigs_MN167524Weighted_LogTransformed_HistogramReadlength.pdf]

Weighted Histogram of read lengths

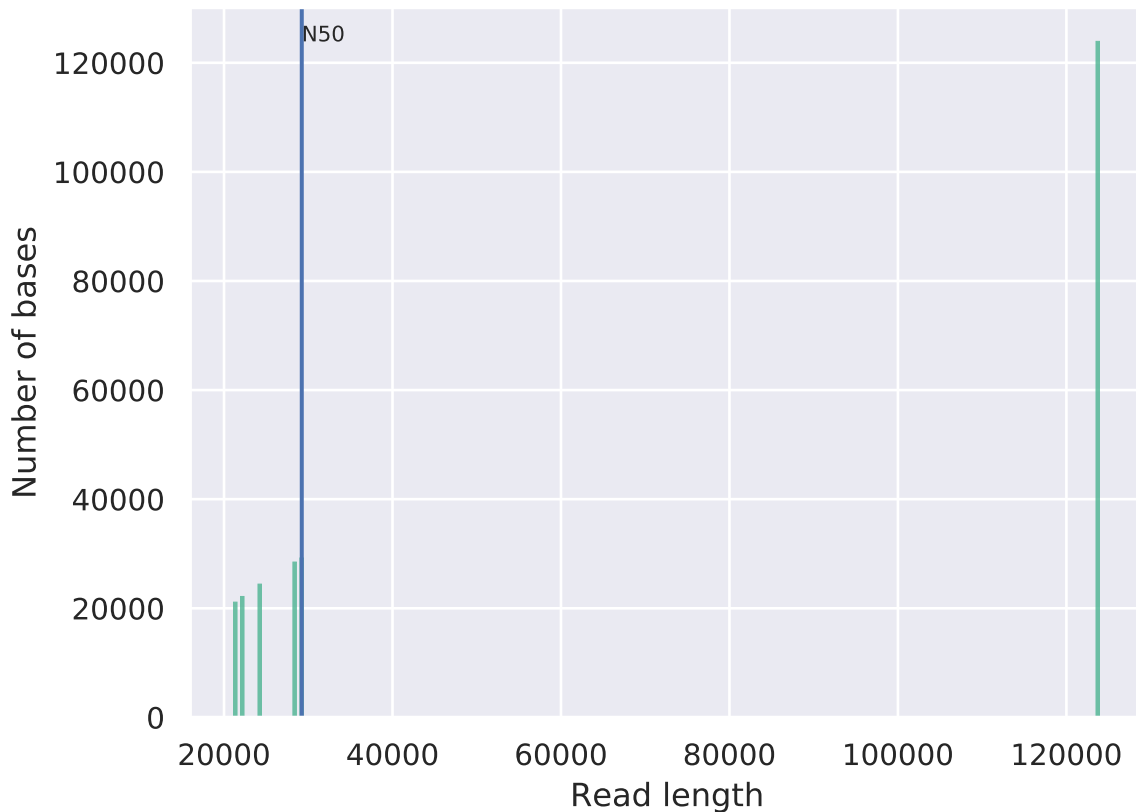

Supplement: Supplementary Figure 1 — AFA and EUR haplotigs. Data Sheets 2-5 are zip files containing the assembled haplotigs for all AFA and EUR assemblies. Also included are Qualimap, NanoPack, and QUAST reports. [file DataSheet_2.zip › SF1d/ccs999KIR7_18_8.contigs_MN167524_reports/ccs999KIR7_18_8.contigs_MN167524Weighted_HistogramReadlength.pdf]

Weighted Histogram of read lengths after log transformation

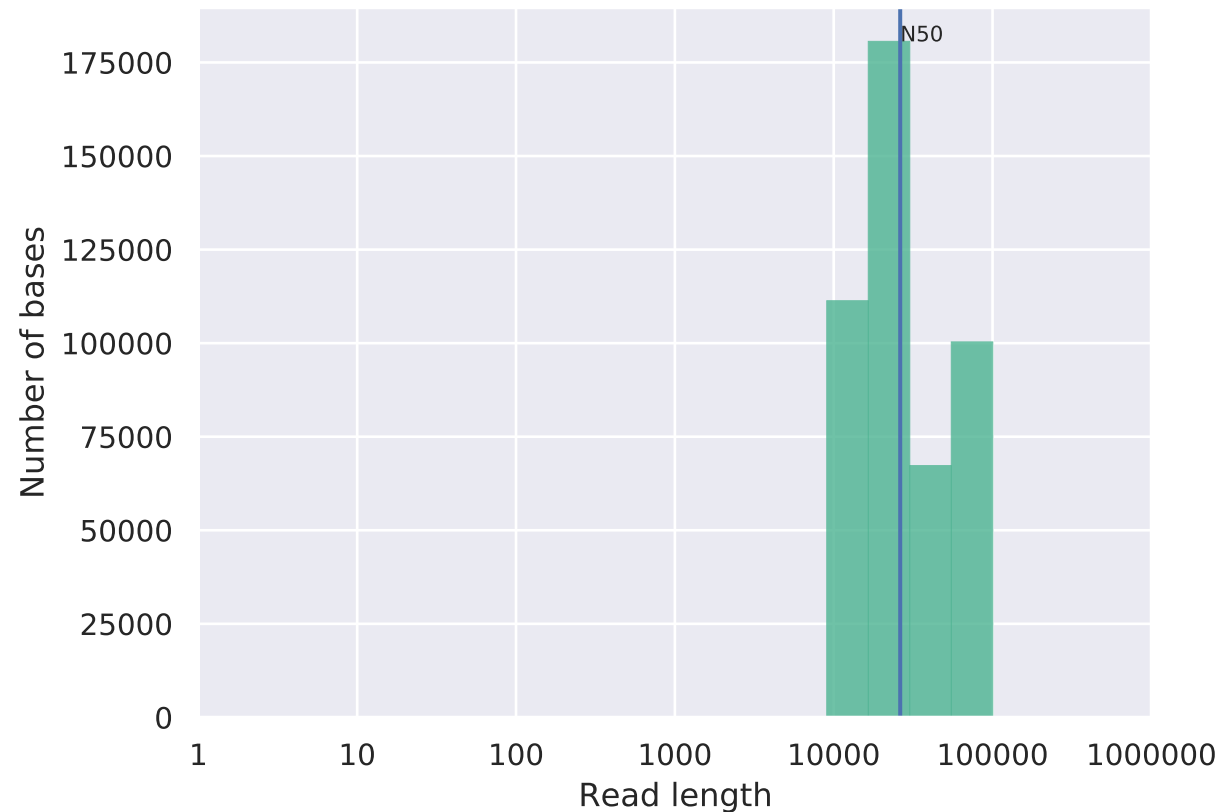

Supplement: Supplementary Figure 1 — AFA and EUR haplotigs. Data Sheets 2-5 are zip files containing the assembled haplotigs for all AFA and EUR assemblies. Also included are Qualimap, NanoPack, and QUAST reports. [file DataSheet_2.zip › SF1d/ccs999KIR7_18_7.contigs_MN167512_reports/ccs999KIR7_18_7.contigs_MN167512Weighted_LogTransformed_HistogramReadlength.pdf]

Histogram of read lengths after log transformation

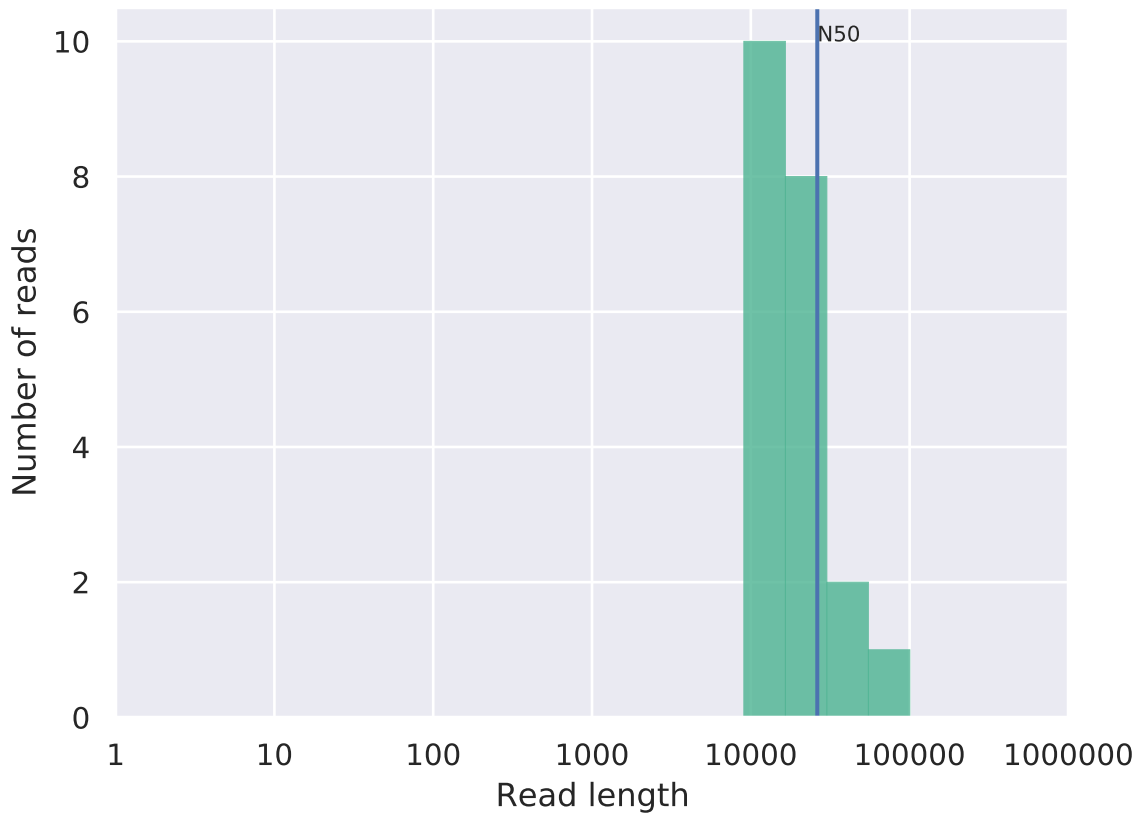

Supplement: Supplementary Figure 1 — AFA and EUR haplotigs. Data Sheets 2-5 are zip files containing the assembled haplotigs for all AFA and EUR assemblies. Also included are Qualimap, NanoPack, and QUAST reports. [file DataSheet_2.zip › SF1d/ccs999KIR7_18_7.contigs_MN167512_reports/ccs999KIR7_18_7.contigs_MN167512LogTransformed_HistogramReadlength.pdf]

# Aligned read length vs Percent identity plot

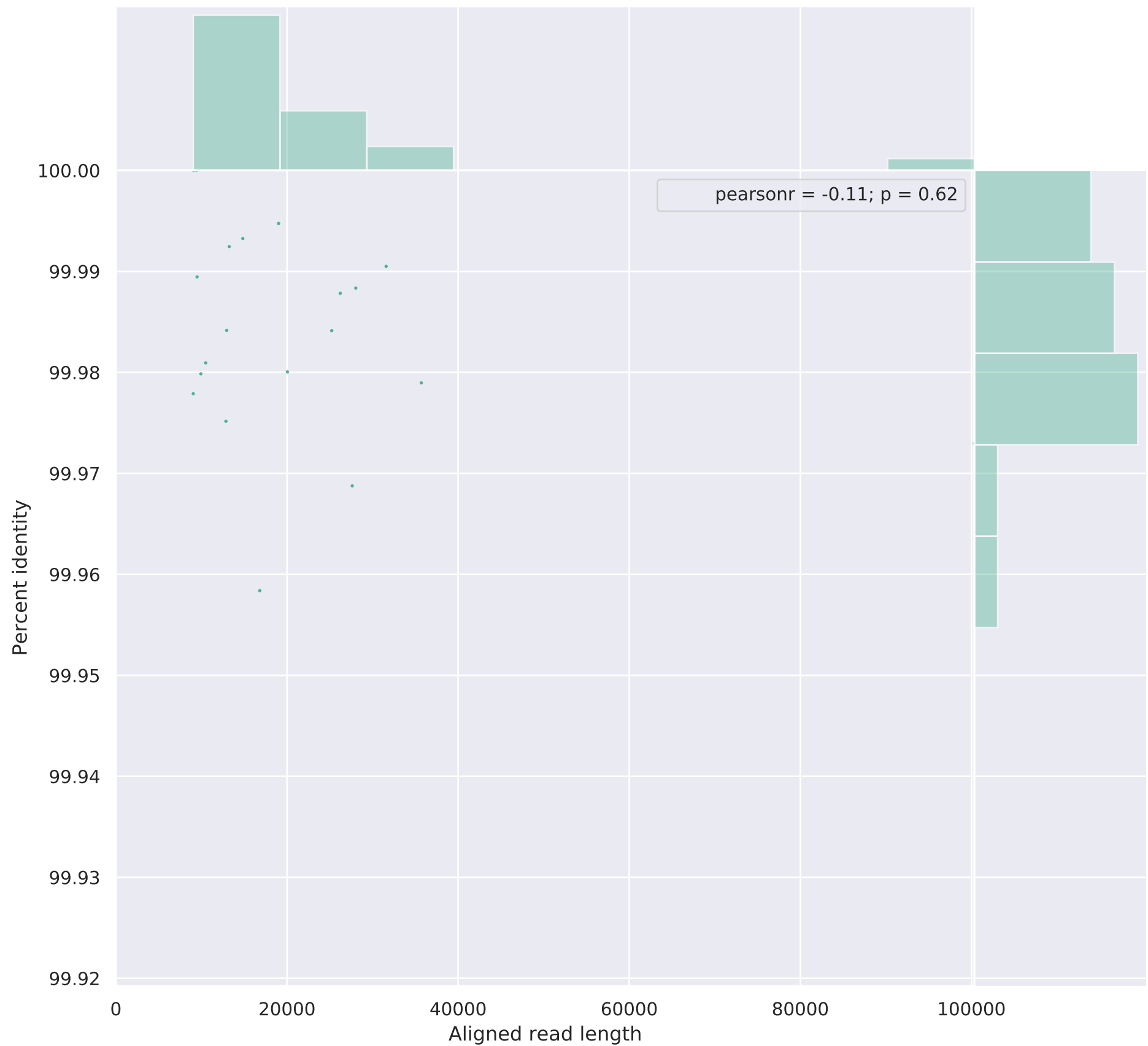

Supplement: Supplementary Figure 1 — AFA and EUR haplotigs. Data Sheets 2-5 are zip files containing the assembled haplotigs for all AFA and EUR assemblies. Also included are Qualimap, NanoPack, and QUAST reports. [file DataSheet_2.zip › SF1d/ccs999KIR7_18_7.contigs_MN167512_reports/ccs999KIR7_18_7.contigs_MN167512PercentIdentityvsAlignedReadLength_dot.pdf]

# Aligned read lengths vs Sequenced read length plot

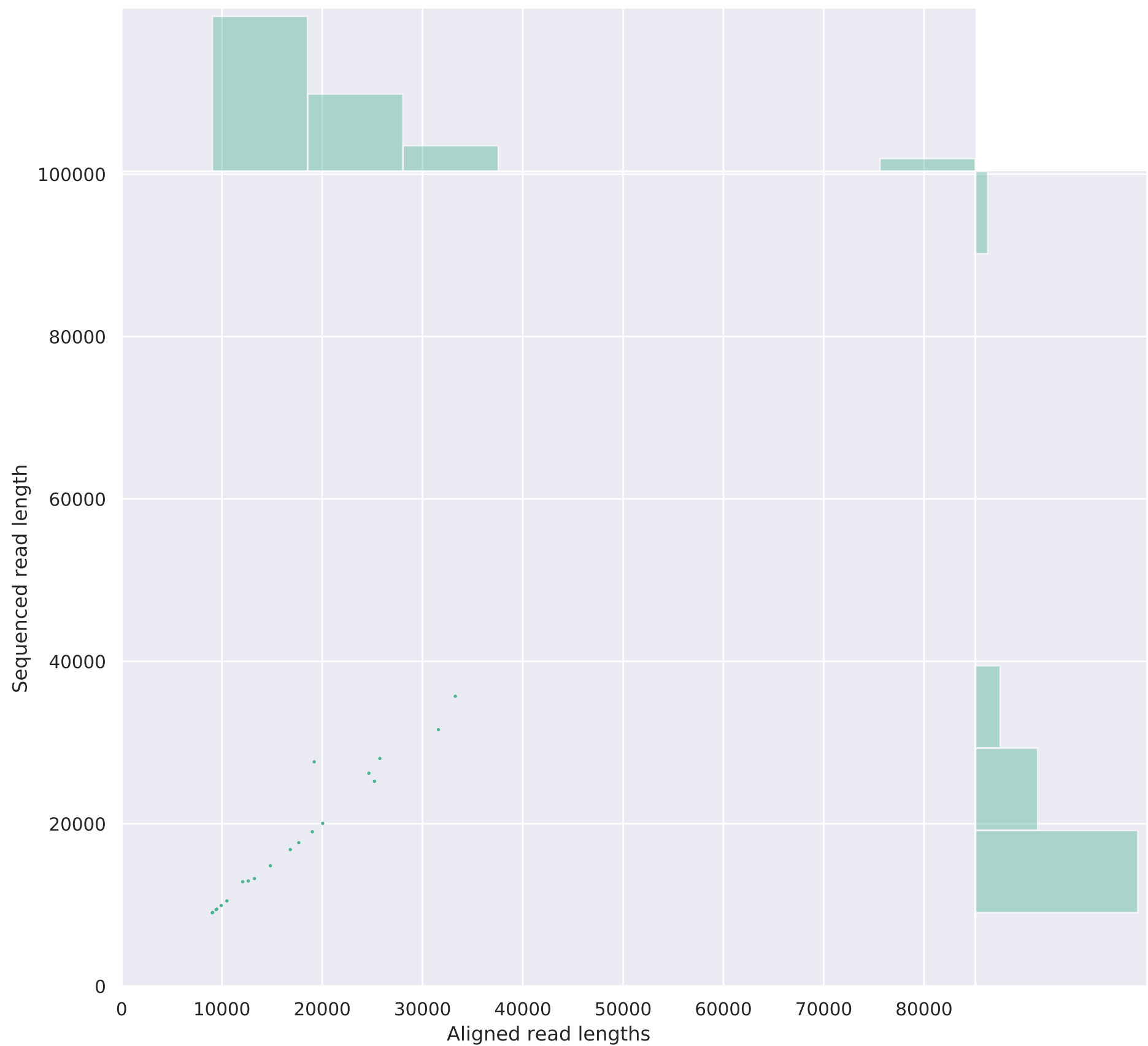

Supplement: Supplementary Figure 1 — AFA and EUR haplotigs. Data Sheets 2-5 are zip files containing the assembled haplotigs for all AFA and EUR assemblies. Also included are Qualimap, NanoPack, and QUAST reports. [file DataSheet_2.zip › SF1d/ccs999KIR7_18_7.contigs_MN167512_reports/ccs999KIR7_18_7.contigs_MN167512AlignedReadlengthvsSequencedReadLength_dot.pdf]

Histogram of read lengths

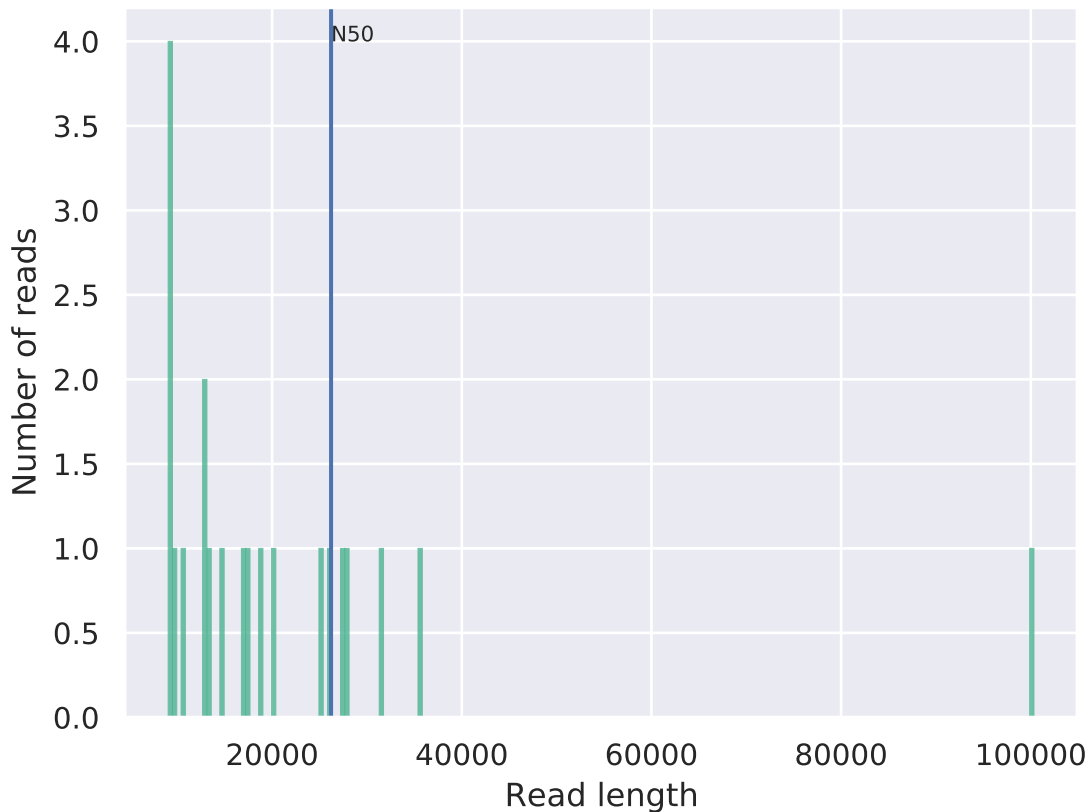

Supplement: Supplementary Figure 1 — AFA and EUR haplotigs. Data Sheets 2-5 are zip files containing the assembled haplotigs for all AFA and EUR assemblies. Also included are Qualimap, NanoPack, and QUAST reports. [file DataSheet_2.zip › SF1d/ccs999KIR7_18_7.contigs_MN167512_reports/ccs999KIR7_18_7.contigs_MN167512HistogramReadlength.pdf]

# Aligned read lengths vs Sequenced read length plot

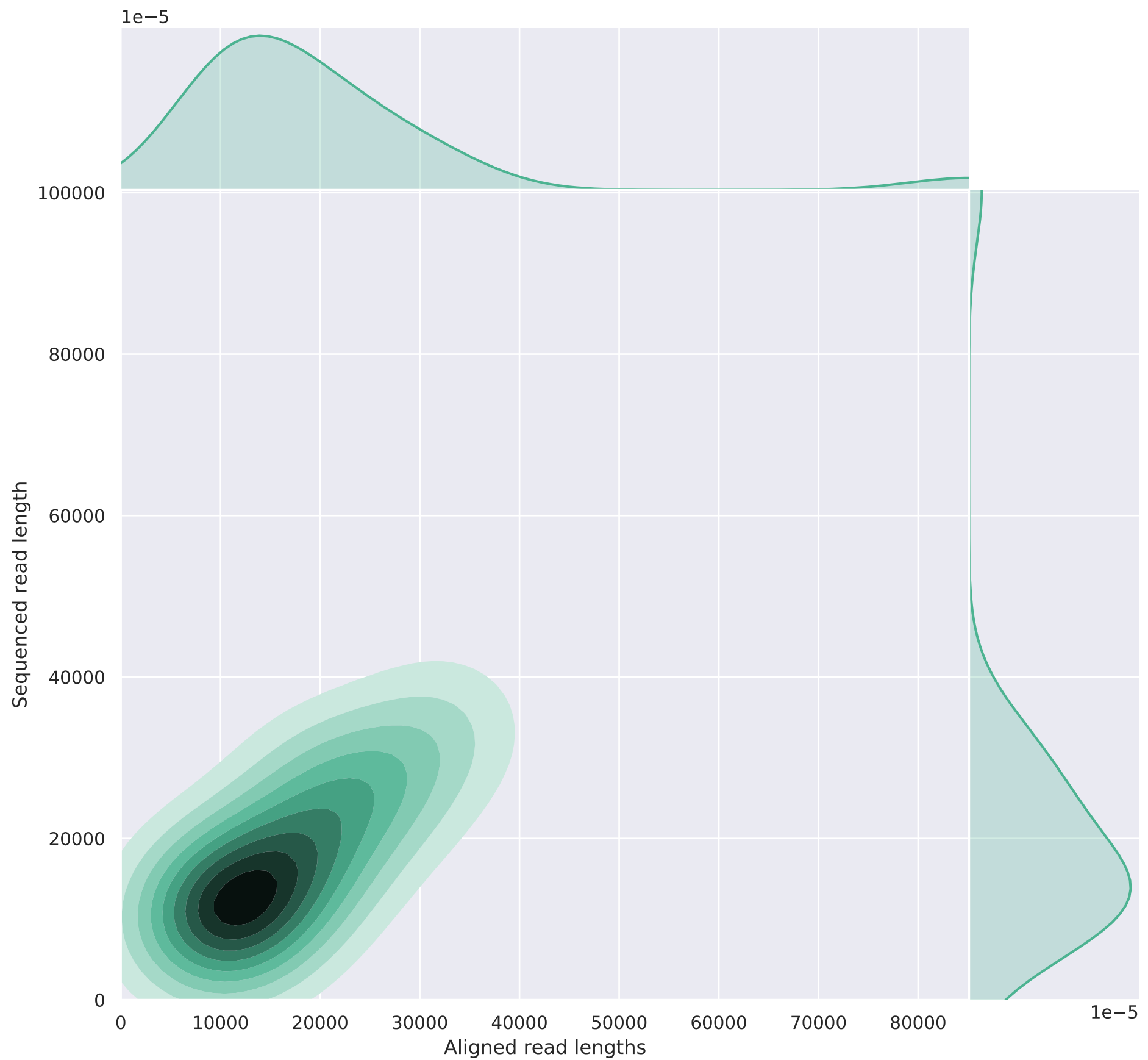

Supplement: Supplementary Figure 1 — AFA and EUR haplotigs. Data Sheets 2-5 are zip files containing the assembled haplotigs for all AFA and EUR assemblies. Also included are Qualimap, NanoPack, and QUAST reports. [file DataSheet_2.zip › SF1d/ccs999KIR7_18_7.contigs_MN167512_reports/ccs999KIR7_18_7.contigs_MN167512AlignedReadlengthvsSequencedReadLength_kde.pdf]

# Aligned read length vs Percent identity plot

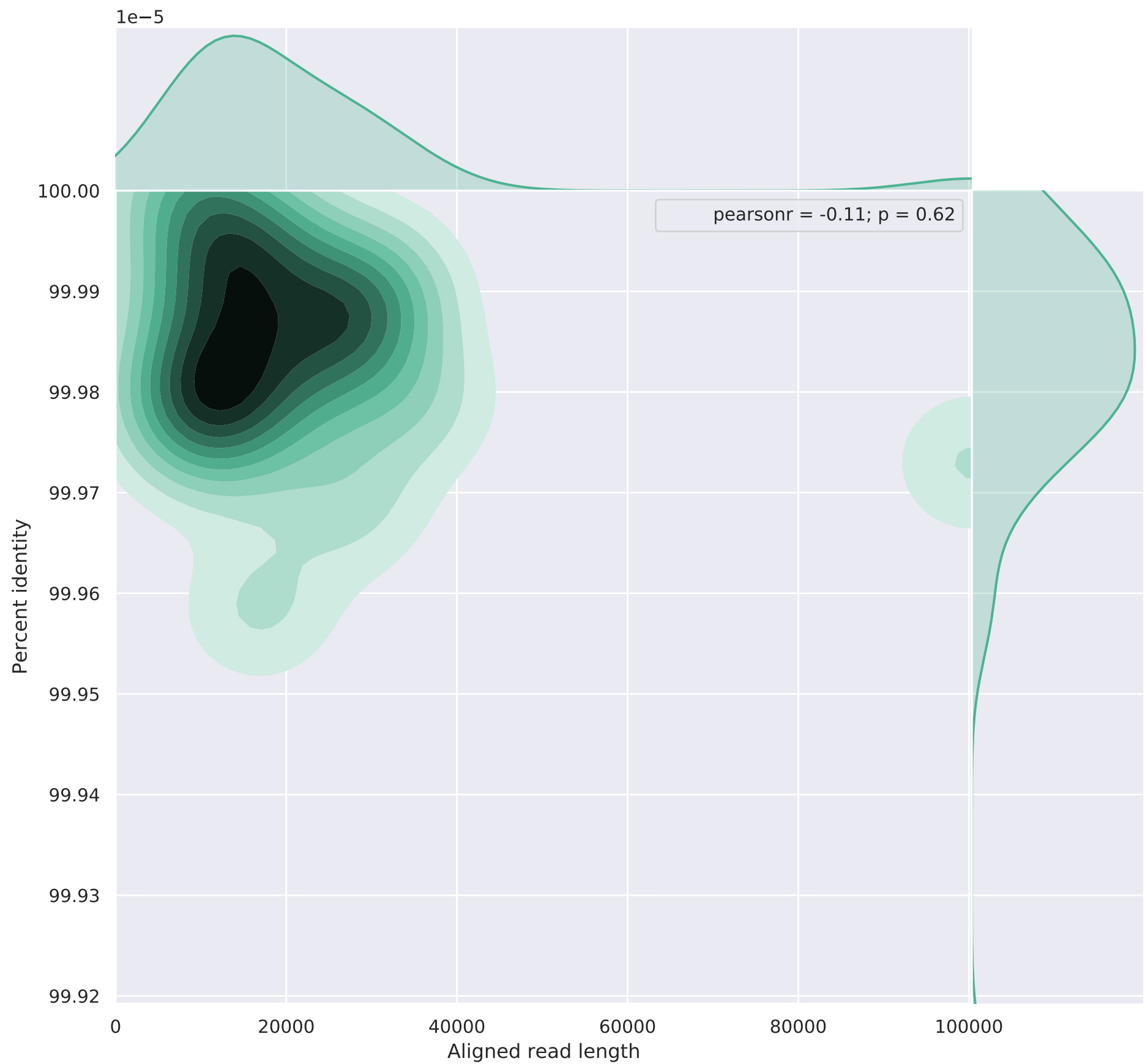

Supplement: Supplementary Figure 1 — AFA and EUR haplotigs. Data Sheets 2-5 are zip files containing the assembled haplotigs for all AFA and EUR assemblies. Also included are Qualimap, NanoPack, and QUAST reports. [file DataSheet_2.zip › SF1d/ccs999KIR7_18_7.contigs_MN167512_reports/ccs999KIR7_18_7.contigs_MN167512PercentIdentityvsAlignedReadLength_kde.pdf]

Weighted Histogram of read lengths

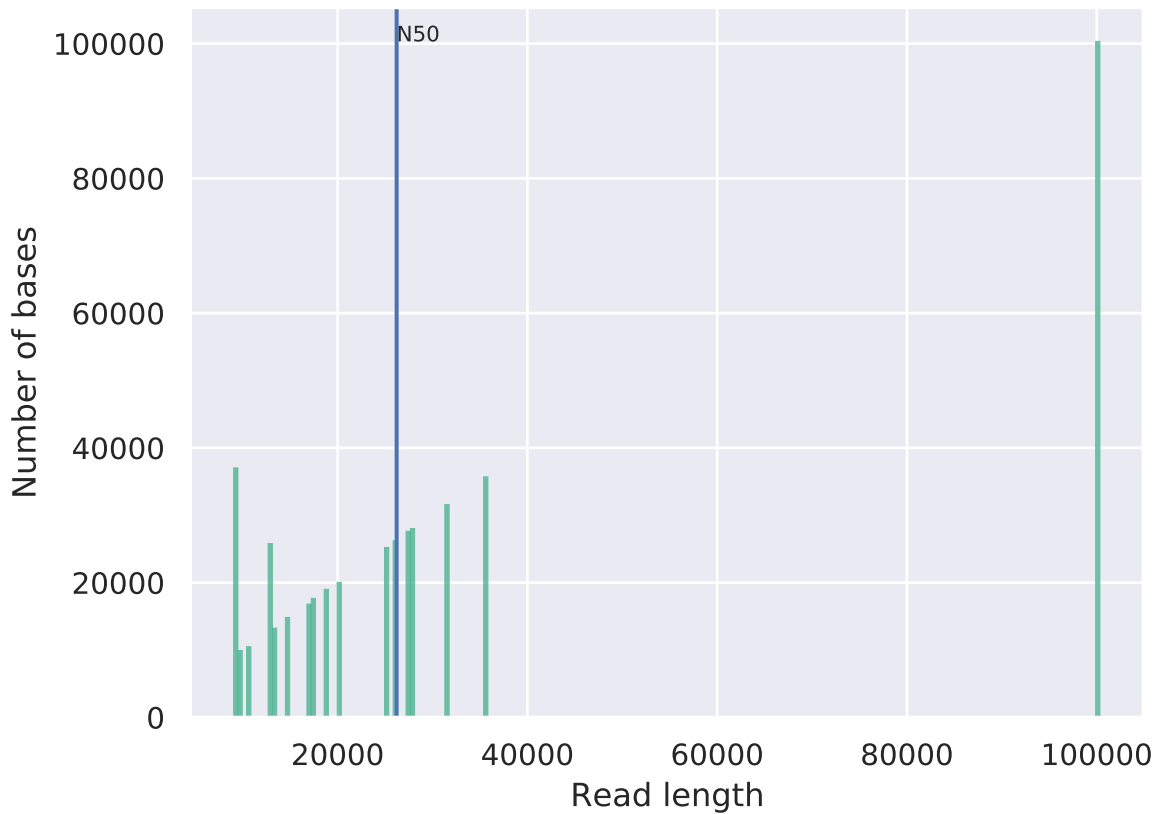

Supplement: Supplementary Figure 1 — AFA and EUR haplotigs. Data Sheets 2-5 are zip files containing the assembled haplotigs for all AFA and EUR assemblies. Also included are Qualimap, NanoPack, and QUAST reports. [file DataSheet_2.zip › SF1d/ccs999KIR7_18_7.contigs_MN167512_reports/ccs999KIR7_18_7.contigs_MN167512Weighted_HistogramReadlength.pdf]

Yield by length

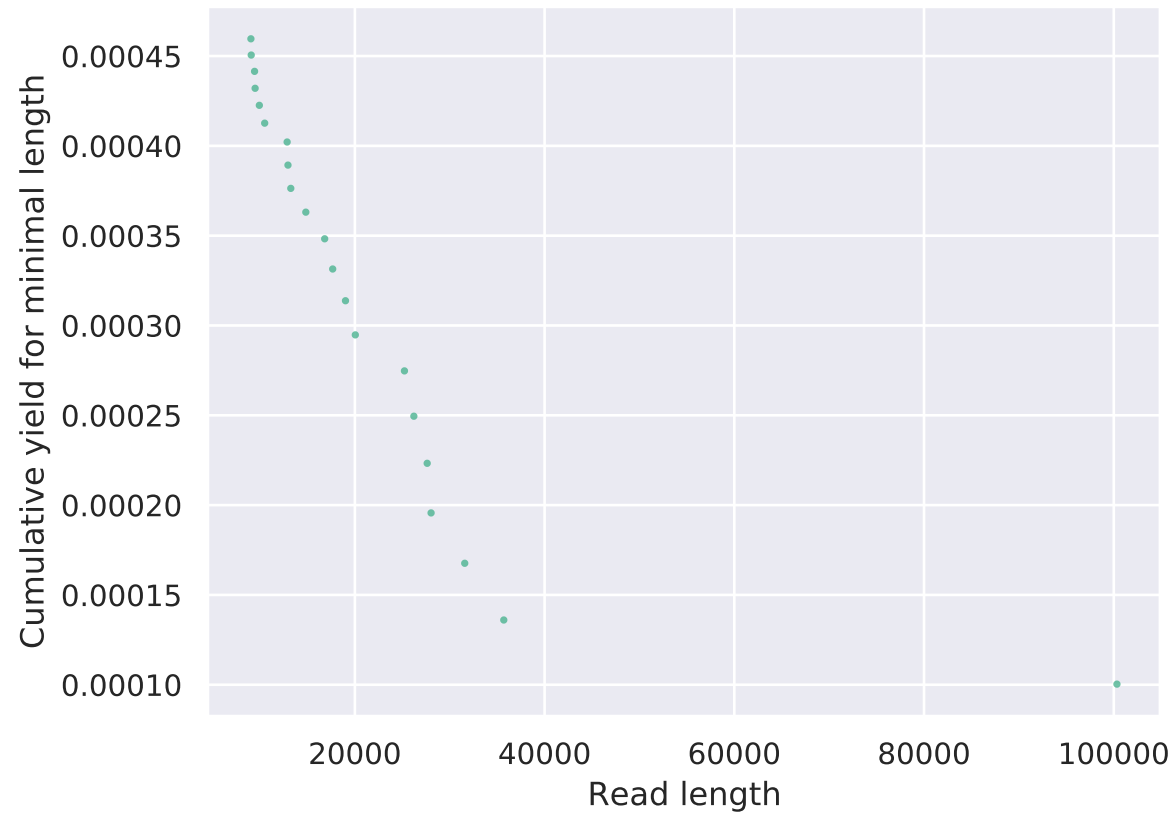

Supplement: Supplementary Figure 1 — AFA and EUR haplotigs. Data Sheets 2-5 are zip files containing the assembled haplotigs for all AFA and EUR assemblies. Also included are Qualimap, NanoPack, and QUAST reports. [file DataSheet_2.zip › SF1d/ccs999KIR7_18_7.contigs_MN167512_reports/ccs999KIR7_18_7.contigs_MN167512Yield_By_Length.pdf]

Yield by length

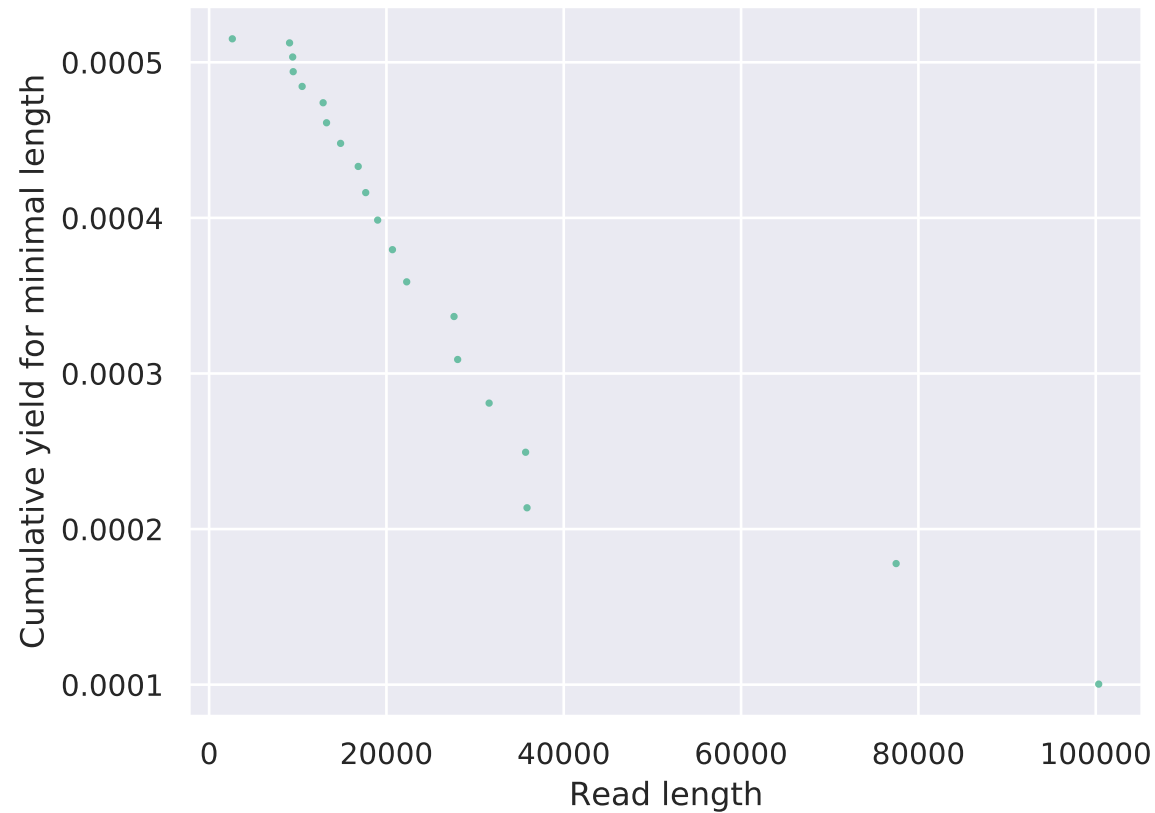

Supplement: Supplementary Figure 1 — AFA and EUR haplotigs. Data Sheets 2-5 are zip files containing the assembled haplotigs for all AFA and EUR assemblies. Also included are Qualimap, NanoPack, and QUAST reports. [file DataSheet_2.zip › SF1d/ccs999KIR7_18_7.contigs_MN167513_reports/ccs999KIR7_18_7.contigs_MN167513Yield_By_Length.pdf]

Histogram of read lengths after log transformation

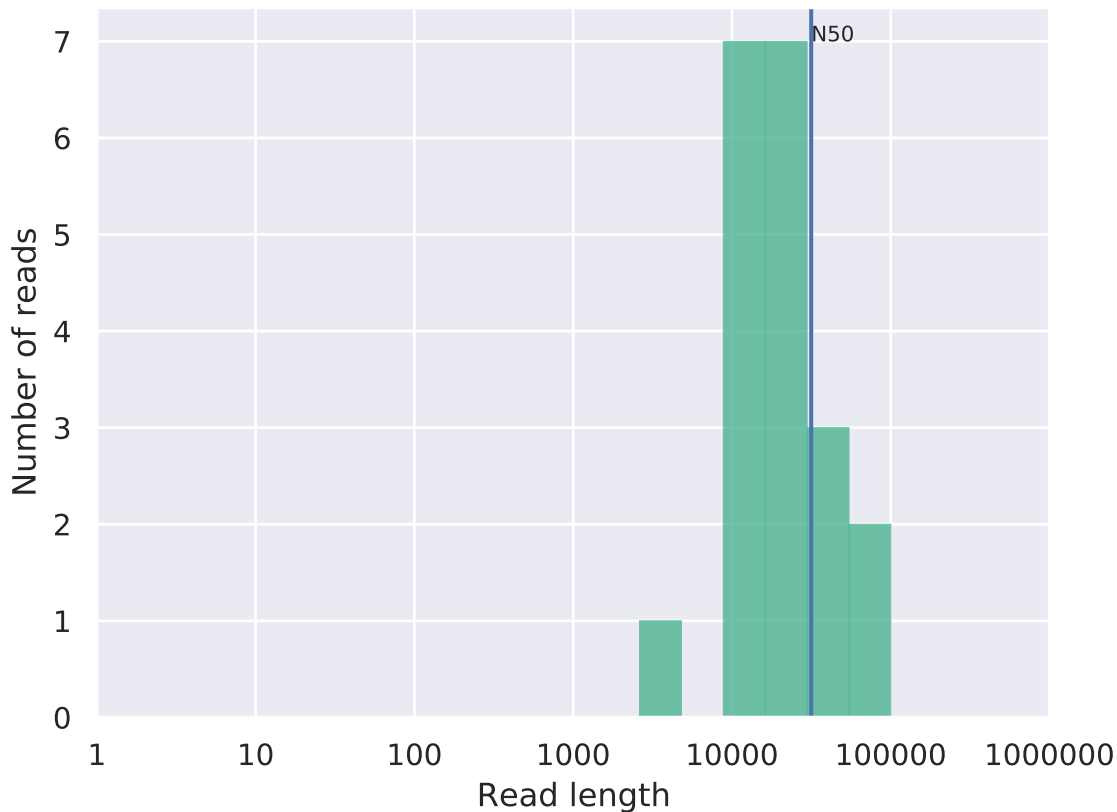

Supplement: Supplementary Figure 1 — AFA and EUR haplotigs. Data Sheets 2-5 are zip files containing the assembled haplotigs for all AFA and EUR assemblies. Also included are Qualimap, NanoPack, and QUAST reports. [file DataSheet_2.zip › SF1d/ccs999KIR7_18_7.contigs_MN167513_reports/ccs999KIR7_18_7.contigs_MN167513LogTransformed_HistogramReadlength.pdf]

# Aligned read length vs Percent identity plot

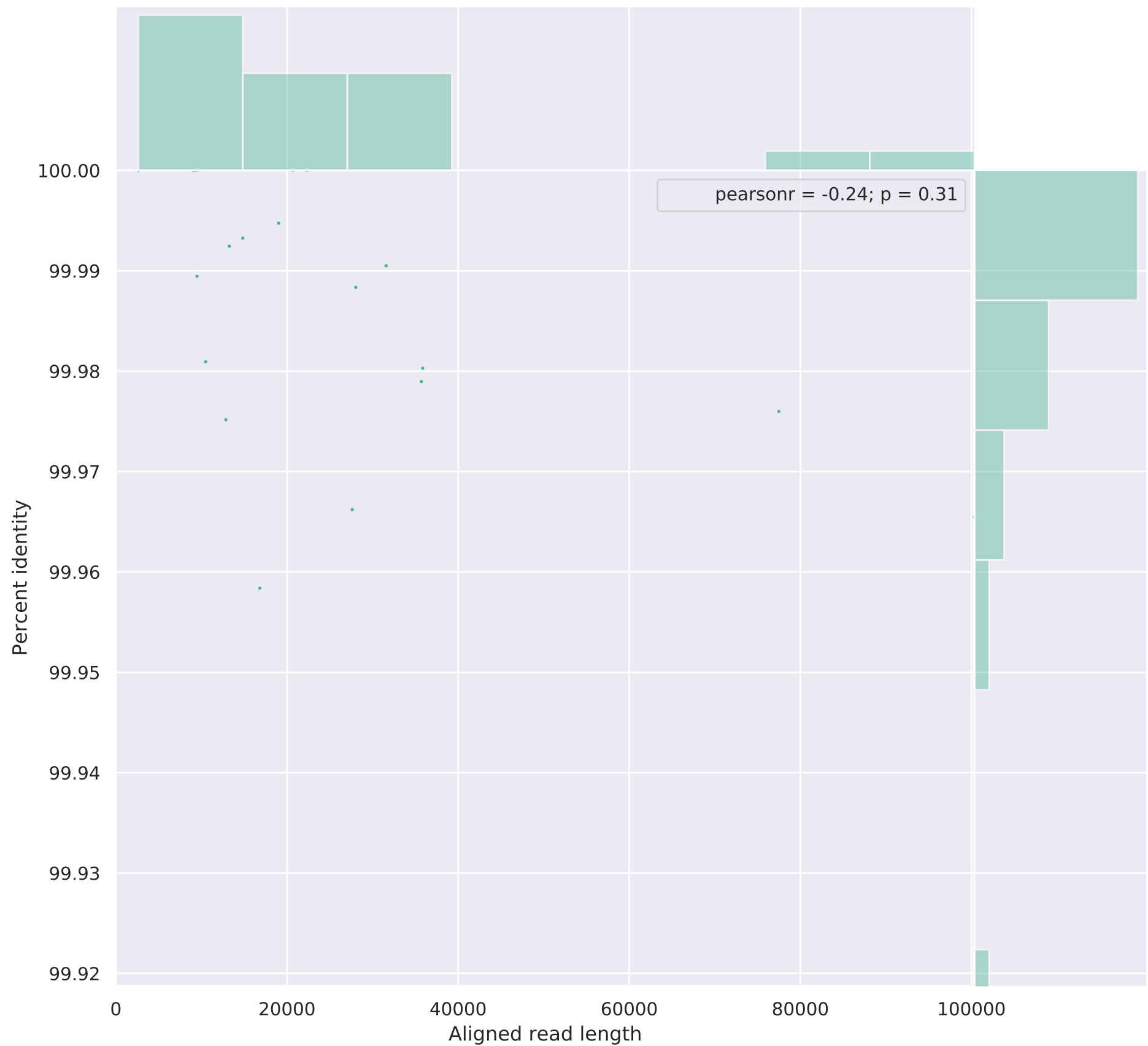

Supplement: Supplementary Figure 1 — AFA and EUR haplotigs. Data Sheets 2-5 are zip files containing the assembled haplotigs for all AFA and EUR assemblies. Also included are Qualimap, NanoPack, and QUAST reports. [file DataSheet_2.zip › SF1d/ccs999KIR7_18_7.contigs_MN167513_reports/ccs999KIR7_18_7.contigs_MN167513PercentIdentityvsAlignedReadLength_dot.pdf]

# Aligned read lengths vs Sequenced read length plot

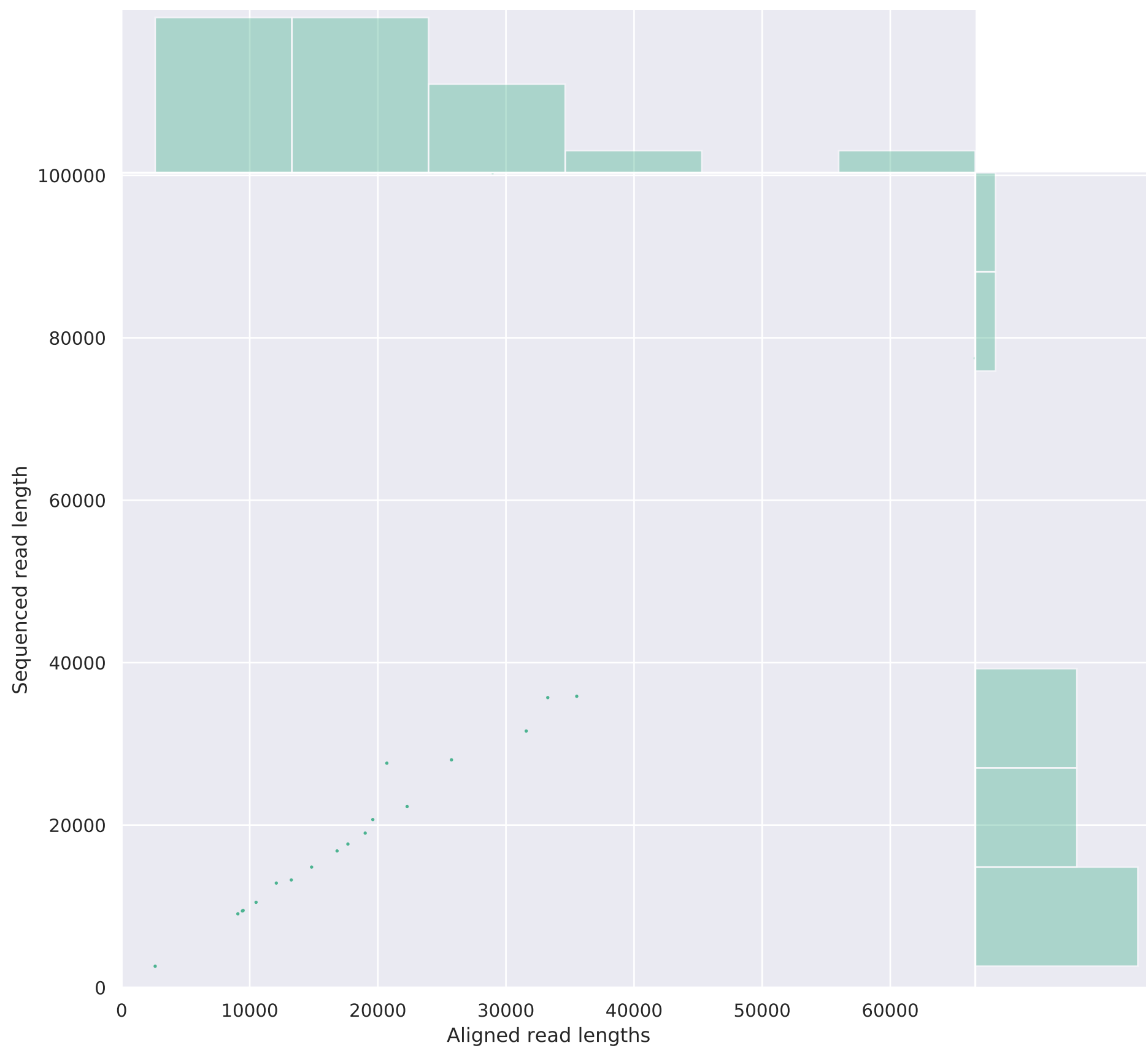

Supplement: Supplementary Figure 1 — AFA and EUR haplotigs. Data Sheets 2-5 are zip files containing the assembled haplotigs for all AFA and EUR assemblies. Also included are Qualimap, NanoPack, and QUAST reports. [file DataSheet_2.zip › SF1d/ccs999KIR7_18_7.contigs_MN167513_reports/ccs999KIR7_18_7.contigs_MN167513AlignedReadlengthvsSequencedReadLength_dot.pdf]

Weighted Histogram of read lengths

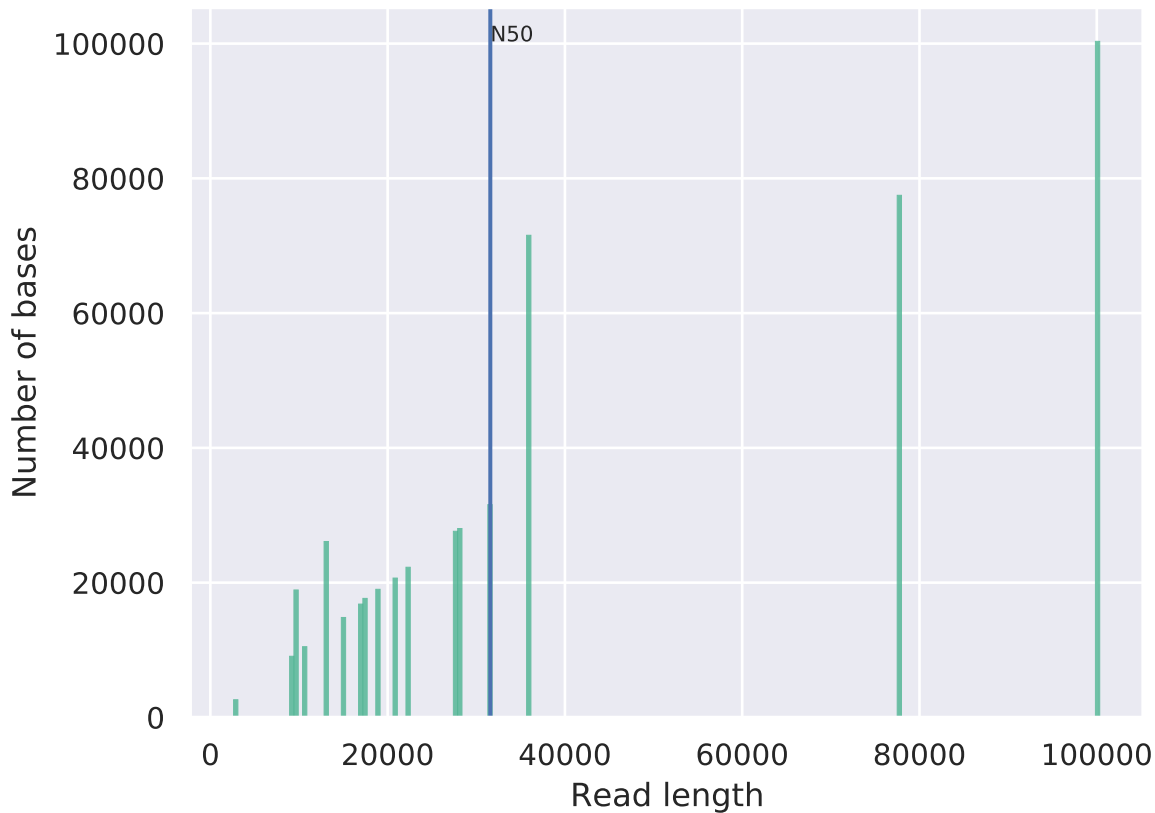

Supplement: Supplementary Figure 1 — AFA and EUR haplotigs. Data Sheets 2-5 are zip files containing the assembled haplotigs for all AFA and EUR assemblies. Also included are Qualimap, NanoPack, and QUAST reports. [file DataSheet_2.zip › SF1d/ccs999KIR7_18_7.contigs_MN167513_reports/ccs999KIR7_18_7.contigs_MN167513Weighted_HistogramReadlength.pdf]

# Aligned read length vs Percent identity plot

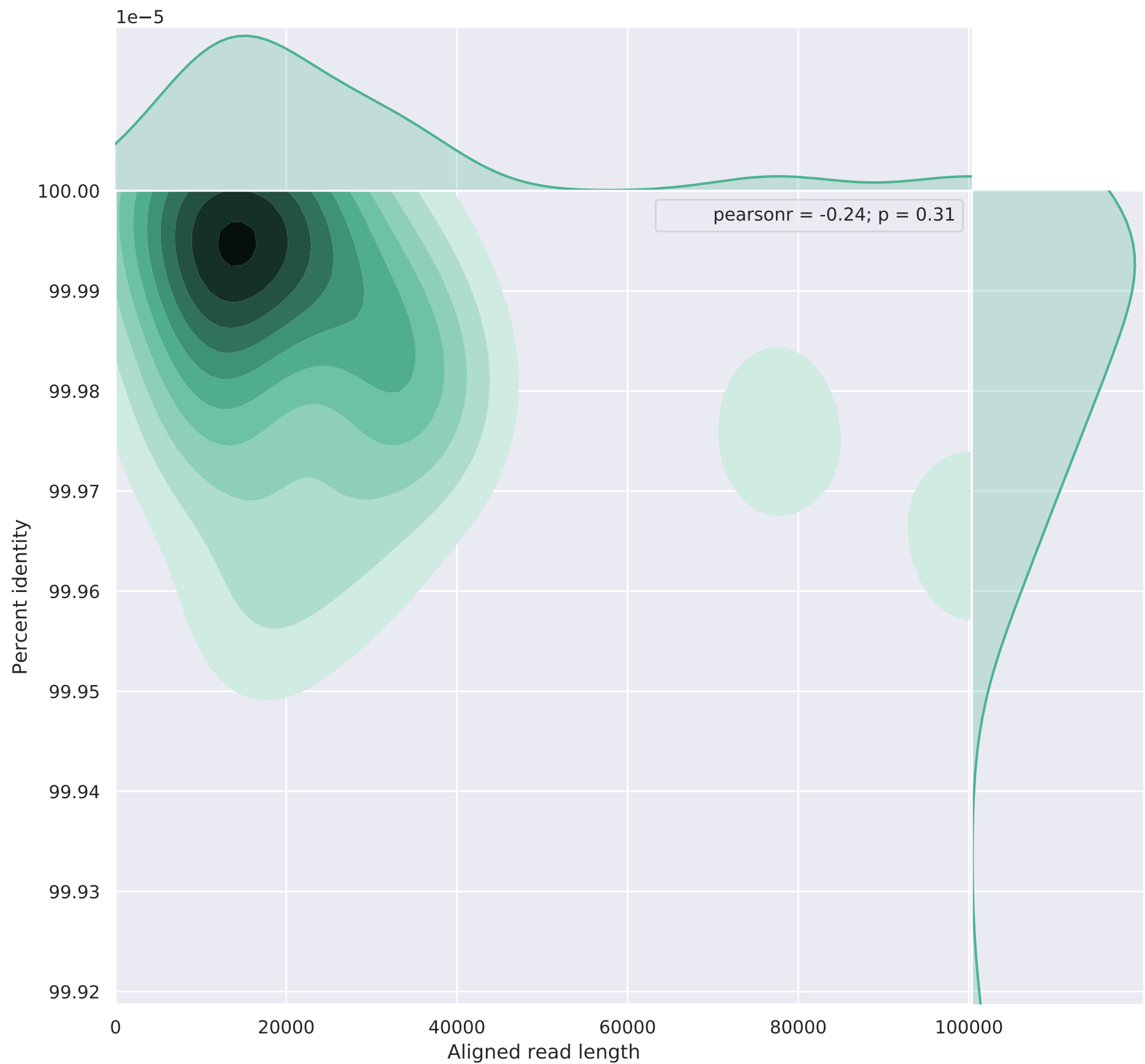

Supplement: Supplementary Figure 1 — AFA and EUR haplotigs. Data Sheets 2-5 are zip files containing the assembled haplotigs for all AFA and EUR assemblies. Also included are Qualimap, NanoPack, and QUAST reports. [file DataSheet_2.zip › SF1d/ccs999KIR7_18_7.contigs_MN167513_reports/ccs999KIR7_18_7.contigs_MN167513PercentIdentityvsAlignedReadLength_kde.pdf]

# Aligned read lengths vs Sequenced read length plot

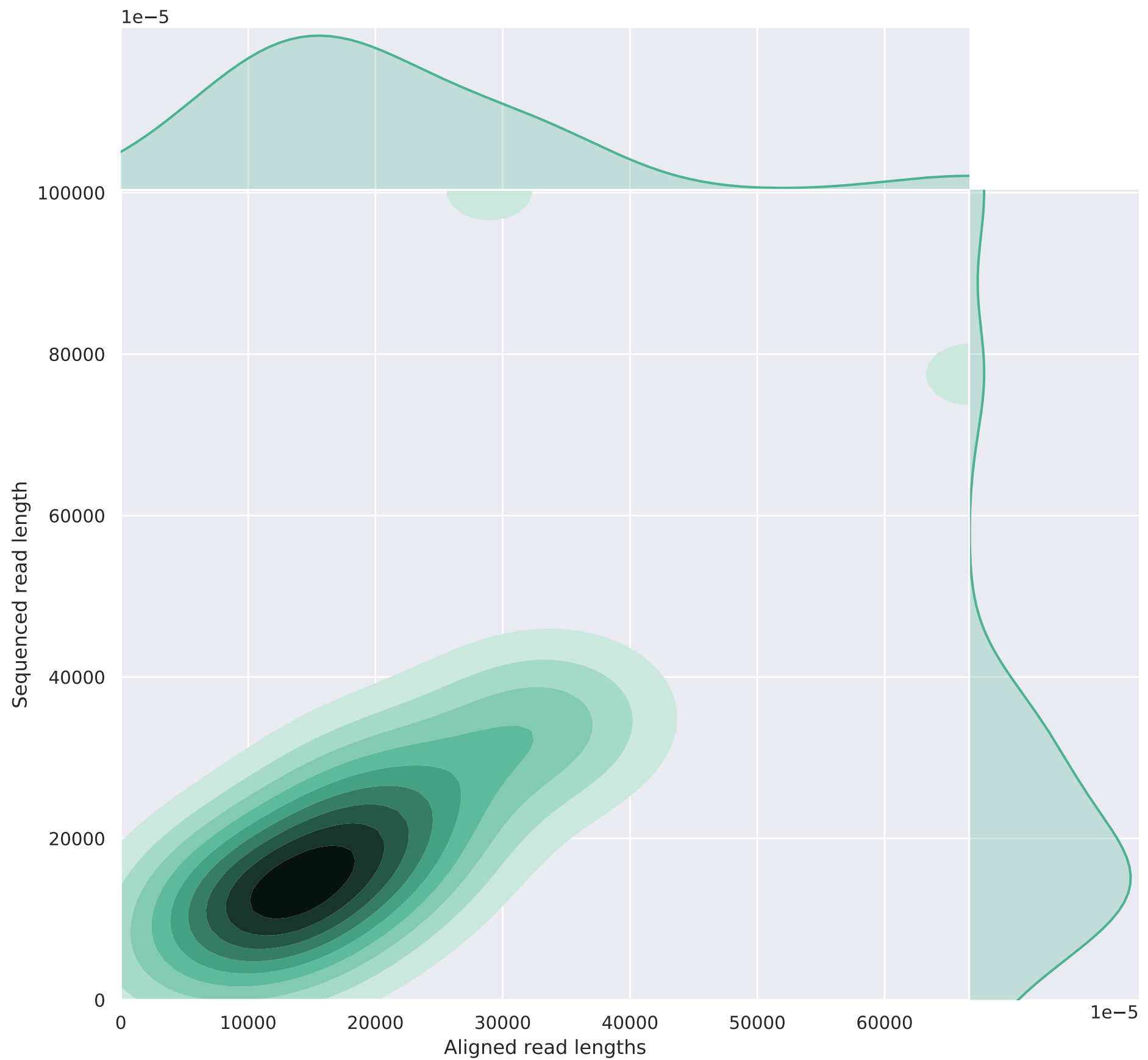

Supplement: Supplementary Figure 1 — AFA and EUR haplotigs. Data Sheets 2-5 are zip files containing the assembled haplotigs for all AFA and EUR assemblies. Also included are Qualimap, NanoPack, and QUAST reports. [file DataSheet_2.zip › SF1d/ccs999KIR7_18_7.contigs_MN167513_reports/ccs999KIR7_18_7.contigs_MN167513AlignedReadlengthvsSequencedReadLength_kde.pdf]

Weighted Histogram of read lengths after log transformation

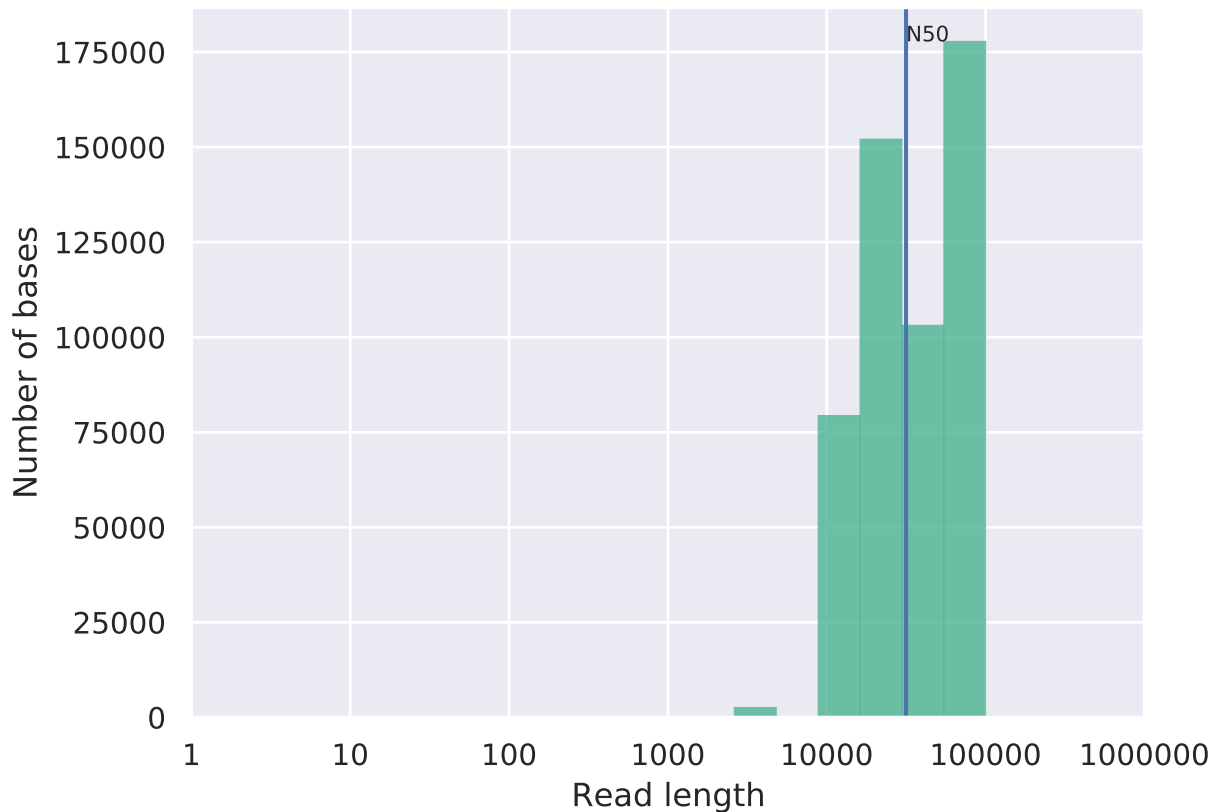

Supplement: Supplementary Figure 1 — AFA and EUR haplotigs. Data Sheets 2-5 are zip files containing the assembled haplotigs for all AFA and EUR assemblies. Also included are Qualimap, NanoPack, and QUAST reports. [file DataSheet_2.zip › SF1d/ccs999KIR7_18_7.contigs_MN167513_reports/ccs999KIR7_18_7.contigs_MN167513Weighted_LogTransformed_HistogramReadlength.pdf]

Histogram of read lengths

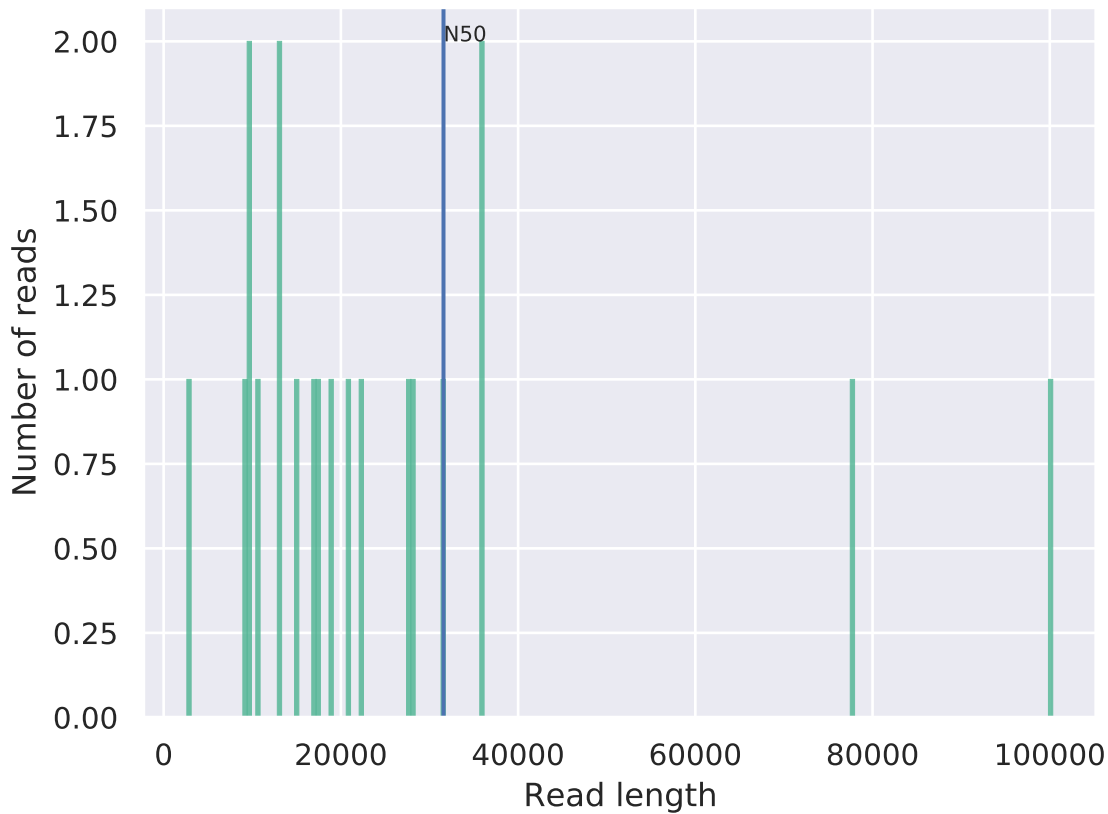

Supplement: Supplementary Figure 1 — AFA and EUR haplotigs. Data Sheets 2-5 are zip files containing the assembled haplotigs for all AFA and EUR assemblies. Also included are Qualimap, NanoPack, and QUAST reports. [file DataSheet_2.zip › SF1d/ccs999KIR7_18_7.contigs_MN167513_reports/ccs999KIR7_18_7.contigs_MN167513HistogramReadlength.pdf]

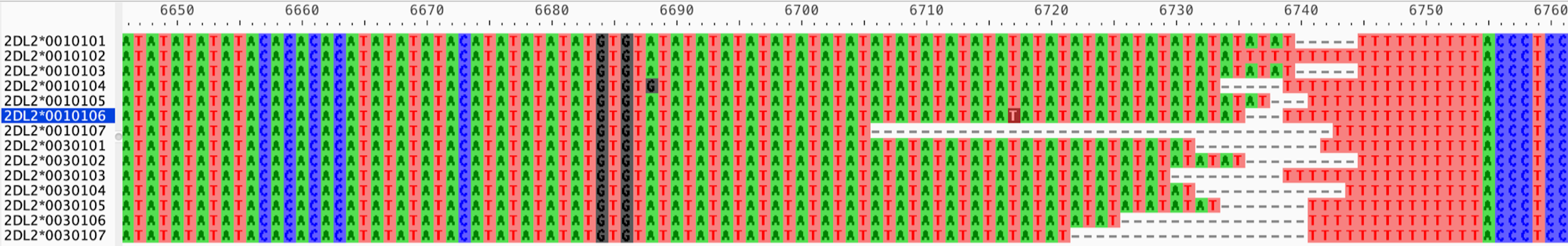

Supplement: Supplementary Figure 3 — Image 3.tif contains a multiple sequence alignment of reference KIR2DL2 alleles in IPD-KIR. The variation between columns 6,706 and 6,744 demonstrates extensive reported variation in a poly-AT region. [file Image_3.tif]

Weighted Histogram of read lengths after log transformation

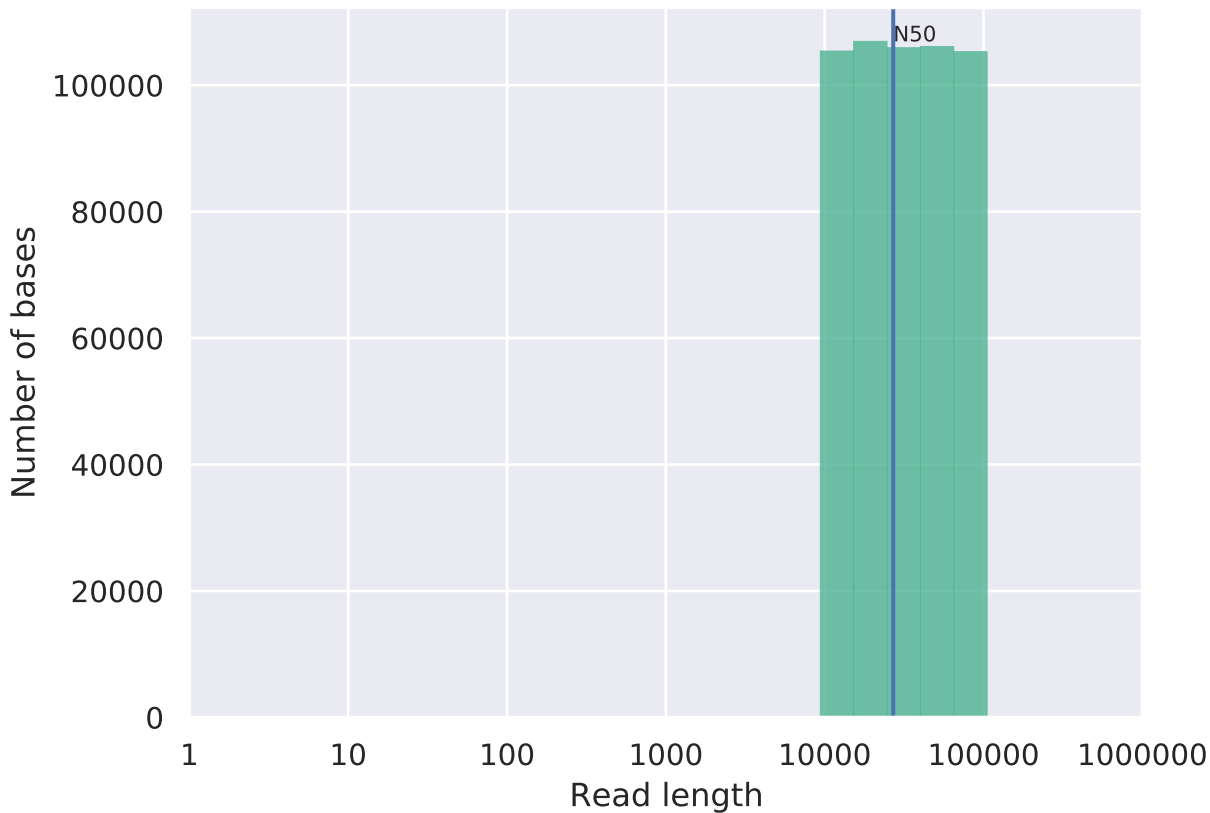

Supplement: Supplementary file 7 [file DataSheet_3.zip › SF1c/ccs999KIR7_18_6.contigs_MN167519_reports/ccs999KIR7_18_6.contigs_MN167519Weighted_LogTransformed_HistogramReadlength.pdf]

Yield by length

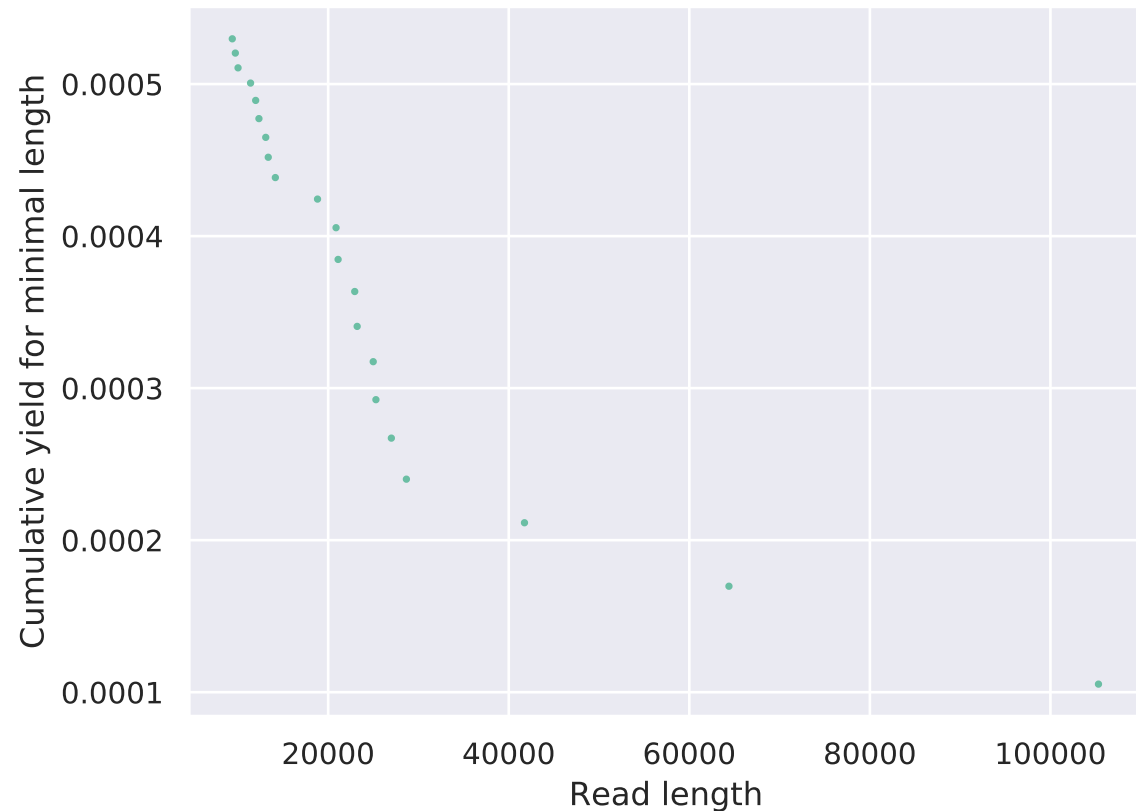

Supplement: Supplementary file 7 [file DataSheet_3.zip › SF1c/ccs999KIR7_18_6.contigs_MN167519_reports/ccs999KIR7_18_6.contigs_MN167519Yield_By_Length.pdf]

# Aligned read length vs Percent identity plot

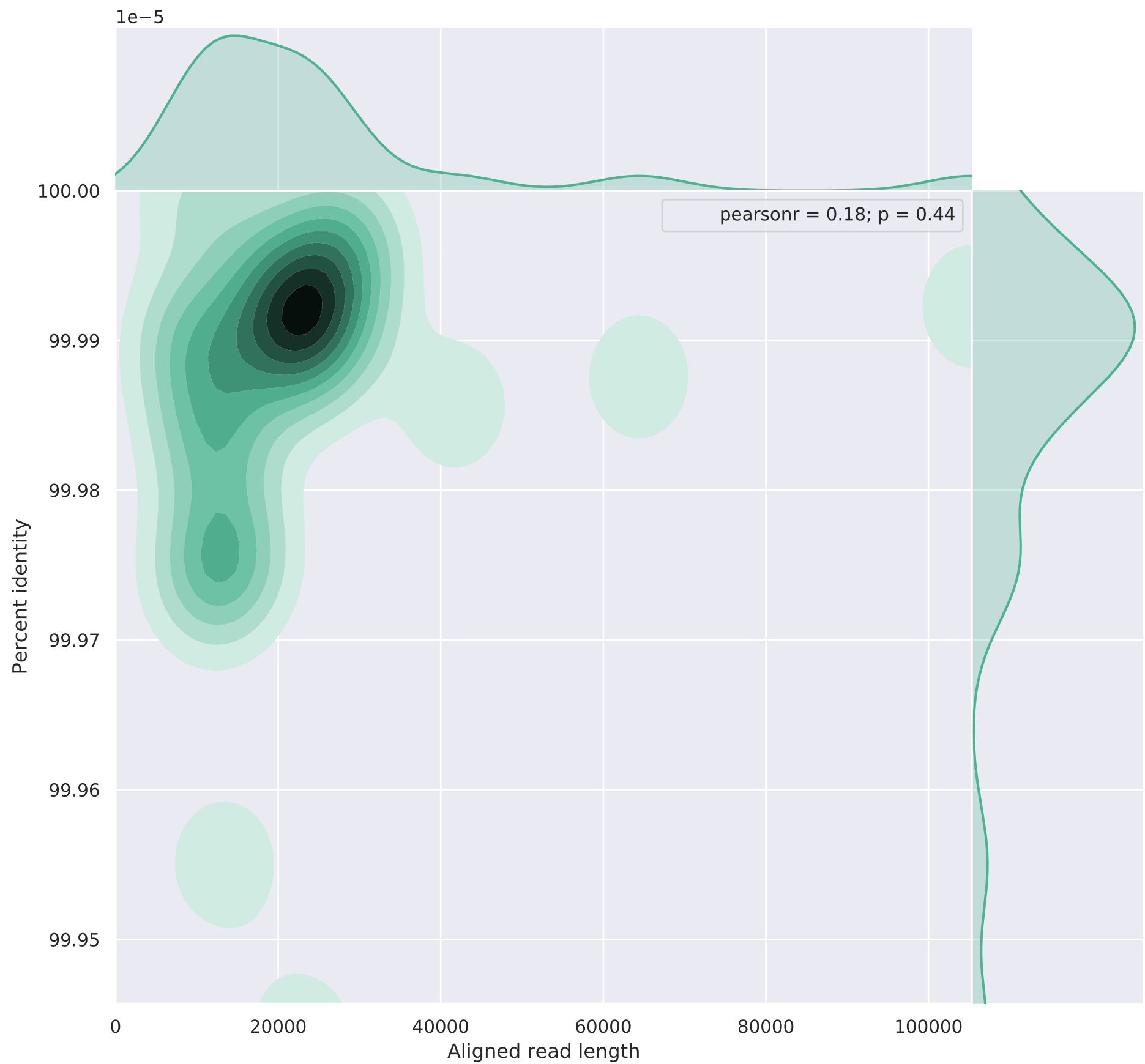

Supplement: Supplementary file 7 [file DataSheet_3.zip › SF1c/ccs999KIR7_18_6.contigs_MN167519_reports/ccs999KIR7_18_6.contigs_MN167519PercentIdentityvsAlignedReadLength_kde.pdf]

# Aligned read lengths vs Sequenced read length plot

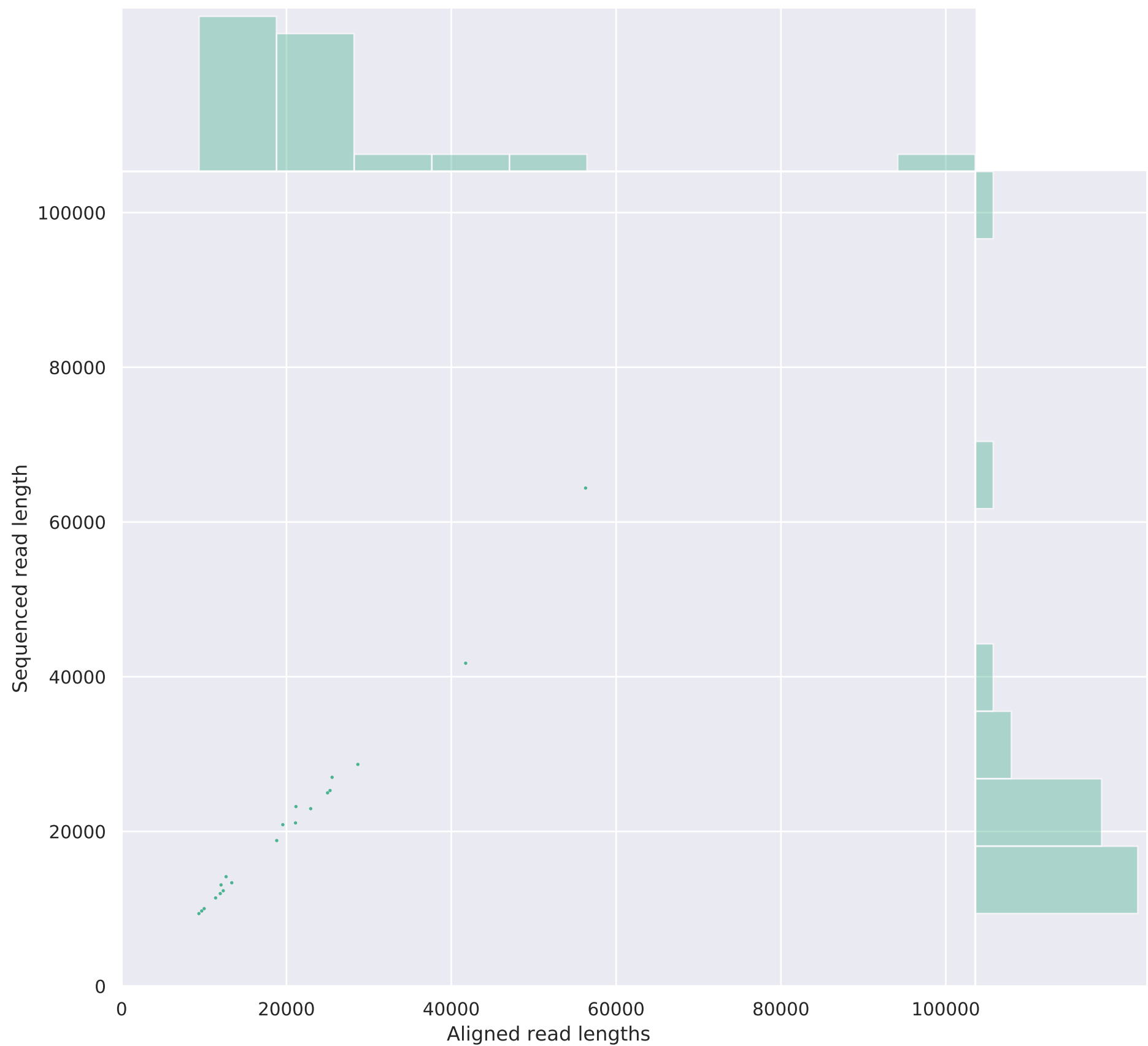

Supplement: Supplementary file 7 [file DataSheet_3.zip › SF1c/ccs999KIR7_18_6.contigs_MN167519_reports/ccs999KIR7_18_6.contigs_MN167519AlignedReadlengthvsSequencedReadLength_dot.pdf]

Weighted Histogram of read lengths

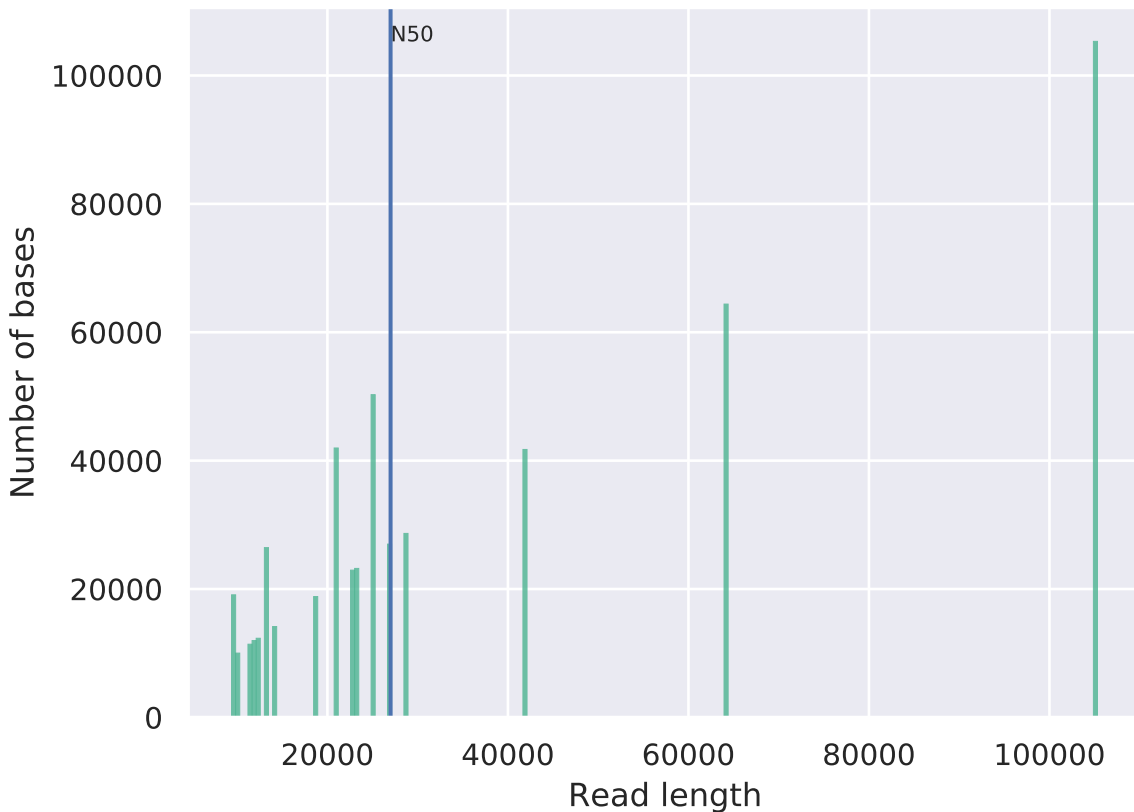

Supplement: Supplementary file 7 [file DataSheet_3.zip › SF1c/ccs999KIR7_18_6.contigs_MN167519_reports/ccs999KIR7_18_6.contigs_MN167519Weighted_HistogramReadlength.pdf]

# Aligned read lengths vs Sequenced read length plot

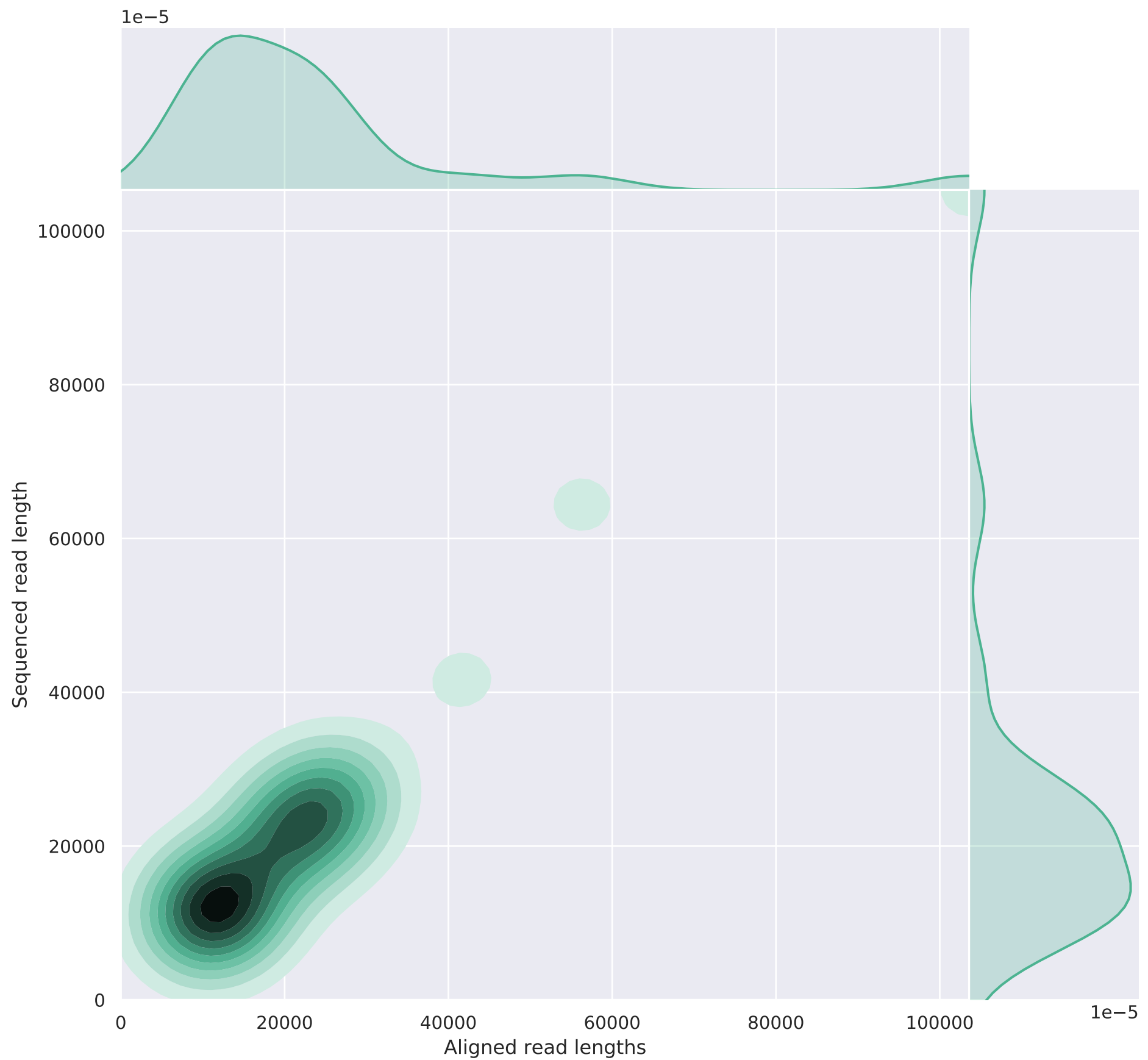

Supplement: Supplementary file 7 [file DataSheet_3.zip › SF1c/ccs999KIR7_18_6.contigs_MN167519_reports/ccs999KIR7_18_6.contigs_MN167519AlignedReadlengthvsSequencedReadLength_kde.pdf]

# Aligned read length vs Percent identity plot

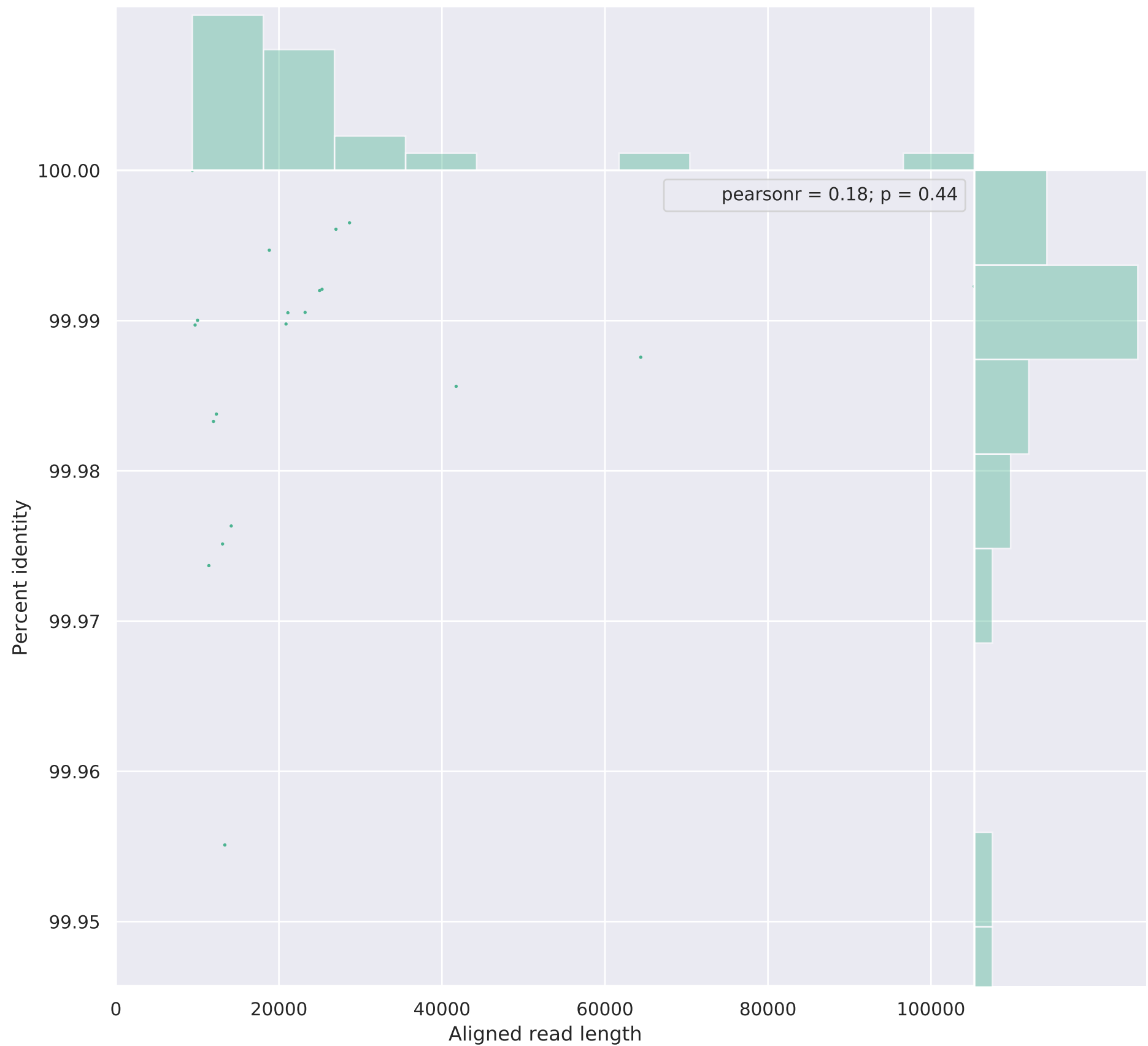

Supplement: Supplementary file 7 [file DataSheet_3.zip › SF1c/ccs999KIR7_18_6.contigs_MN167519_reports/ccs999KIR7_18_6.contigs_MN167519PercentIdentityvsAlignedReadLength_dot.pdf]

Histogram of read lengths after log transformation

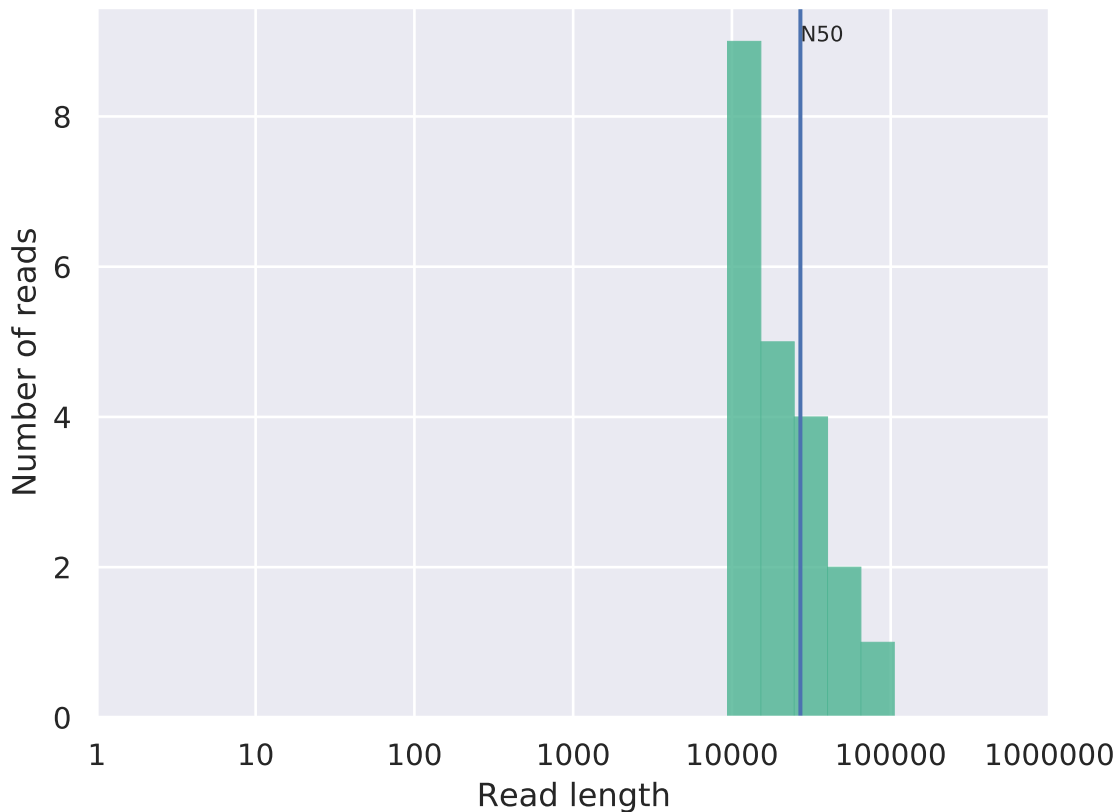

Supplement: Supplementary file 7 [file DataSheet_3.zip › SF1c/ccs999KIR7_18_6.contigs_MN167519_reports/ccs999KIR7_18_6.contigs_MN167519LogTransformed_HistogramReadlength.pdf]

Histogram of read lengths

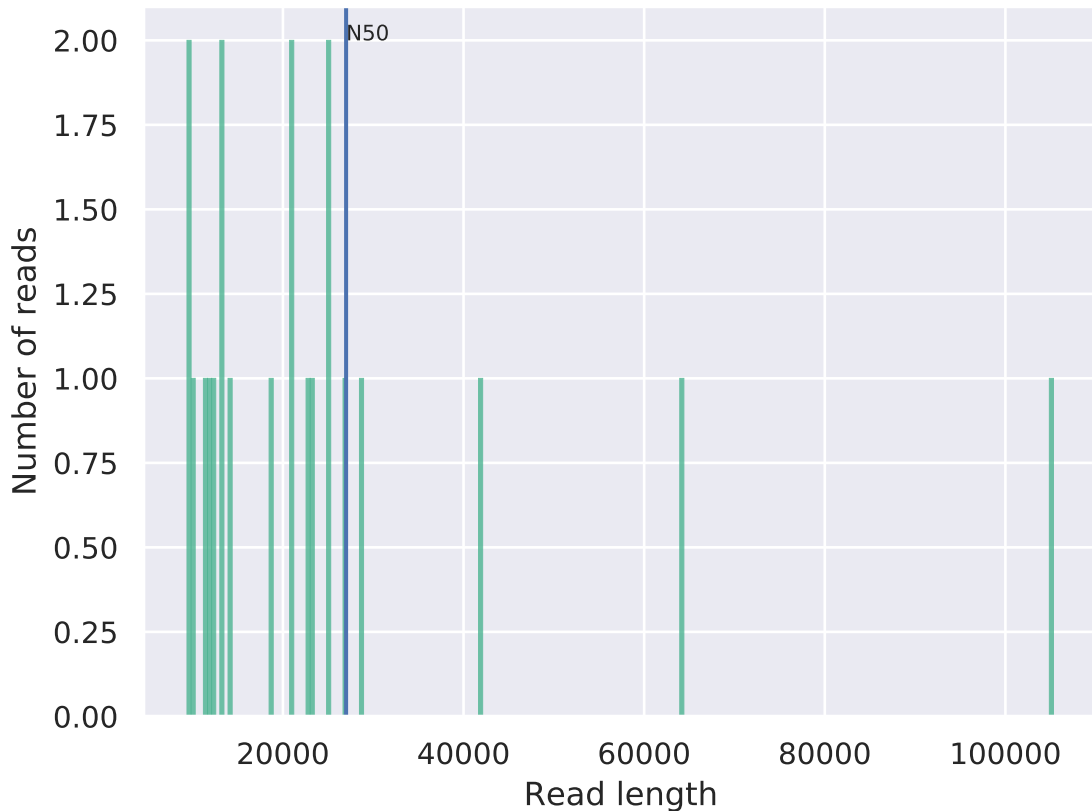

Supplement: Supplementary file 7 [file DataSheet_3.zip › SF1c/ccs999KIR7_18_6.contigs_MN167519_reports/ccs999KIR7_18_6.contigs_MN167519HistogramReadlength.pdf]

Yield by length

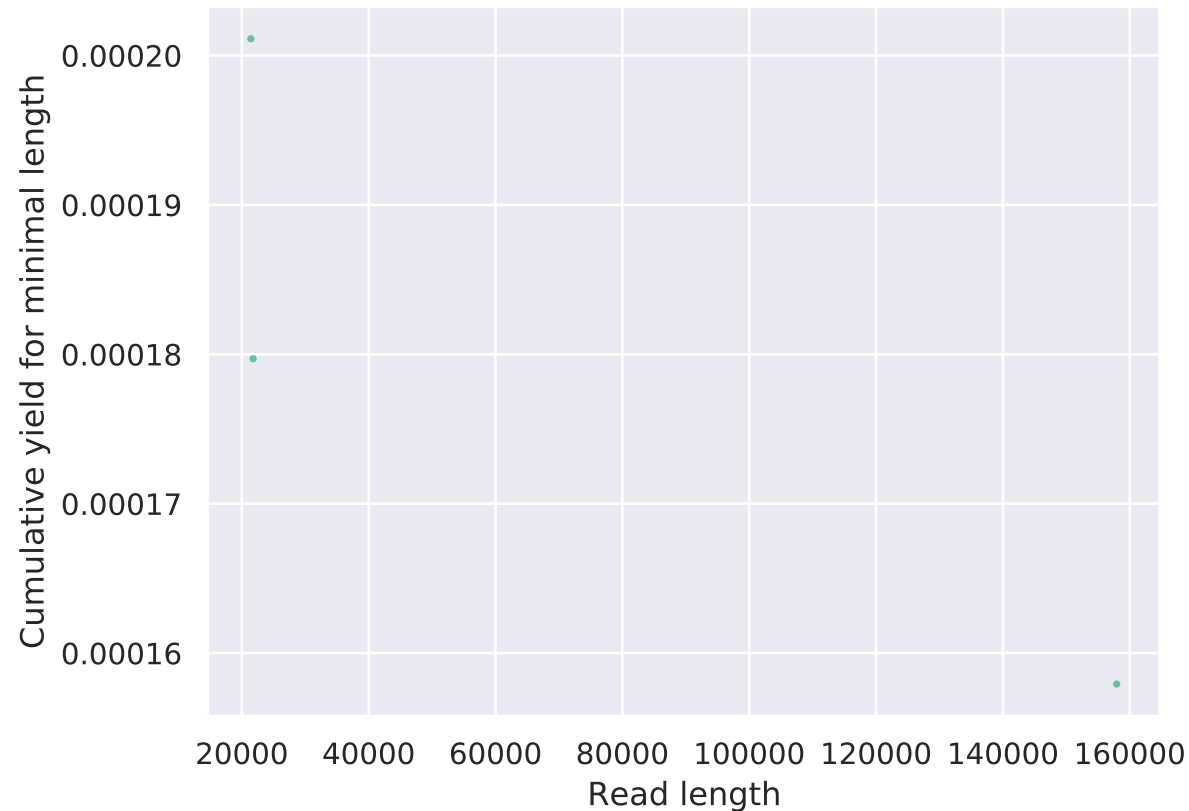

Supplement: Supplementary file 7 [file DataSheet_3.zip › SF1c/ccs999KIR7_18_5.contigs_MN167529_reports/ccs999KIR7_18_5.contigs_MN167529Yield_By_Length.pdf]

# Aligned read lengths vs Sequenced read length plot

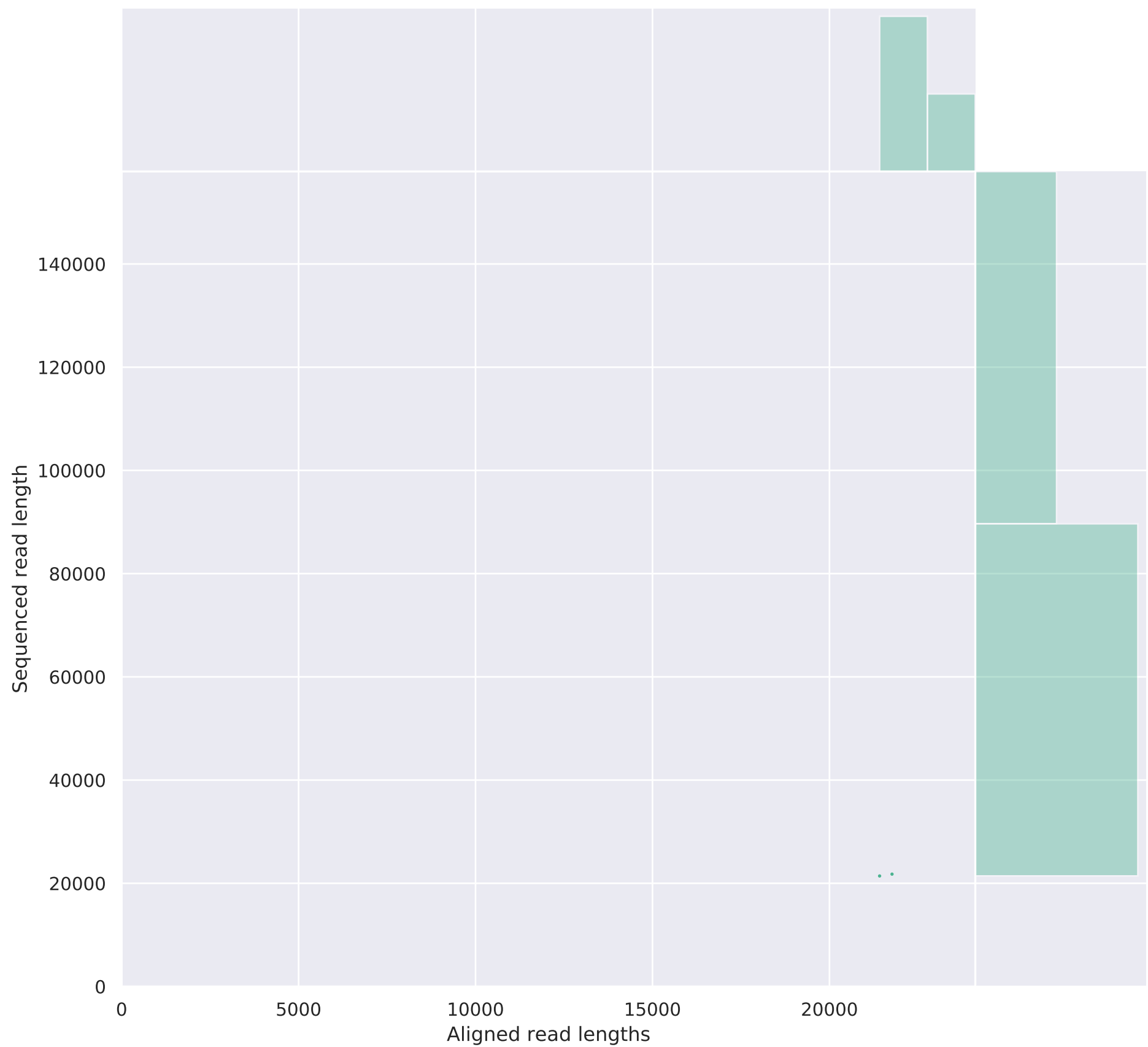

Supplement: Supplementary file 7 [file DataSheet_3.zip › SF1c/ccs999KIR7_18_5.contigs_MN167529_reports/ccs999KIR7_18_5.contigs_MN167529AlignedReadlengthvsSequencedReadLength_dot.pdf]

Histogram of read lengths after log transformation

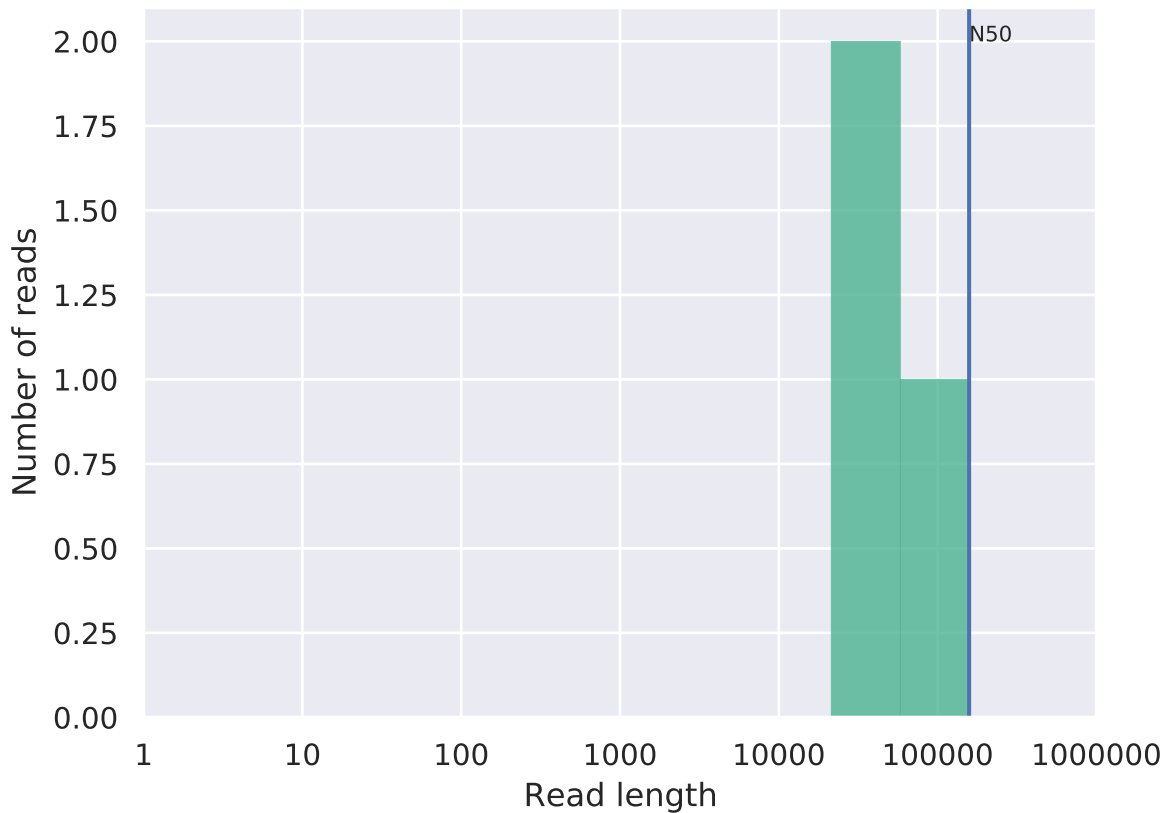

Supplement: Supplementary file 7 [file DataSheet_3.zip › SF1c/ccs999KIR7_18_5.contigs_MN167529_reports/ccs999KIR7_18_5.contigs_MN167529LogTransformed_HistogramReadlength.pdf]

# Aligned read length vs Percent identity plot

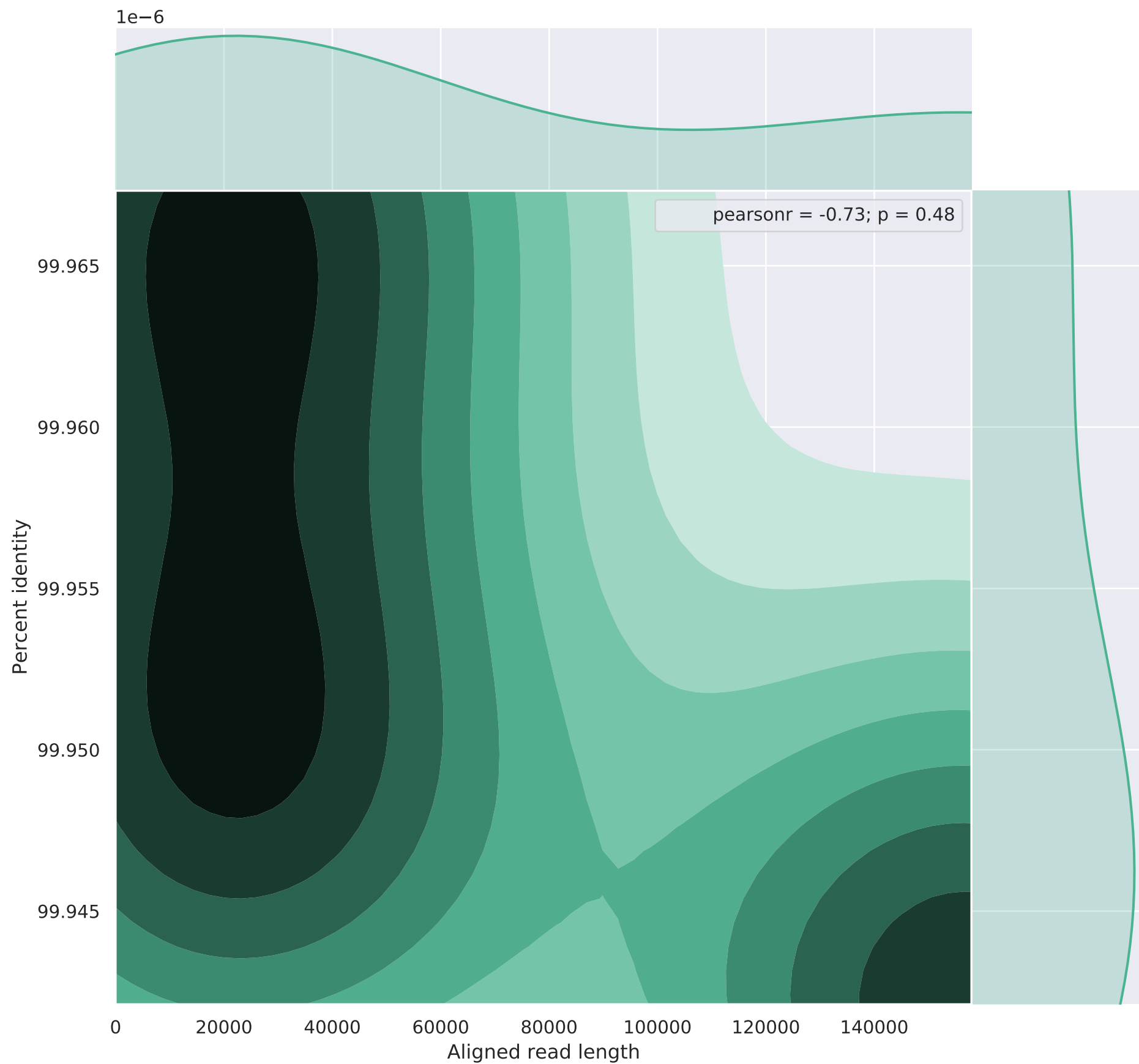

Supplement: Supplementary file 7 [file DataSheet_3.zip › SF1c/ccs999KIR7_18_5.contigs_MN167529_reports/ccs999KIR7_18_5.contigs_MN167529PercentIdentityvsAlignedReadLength_kde.pdf]

Weighted Histogram of read lengths after log transformation

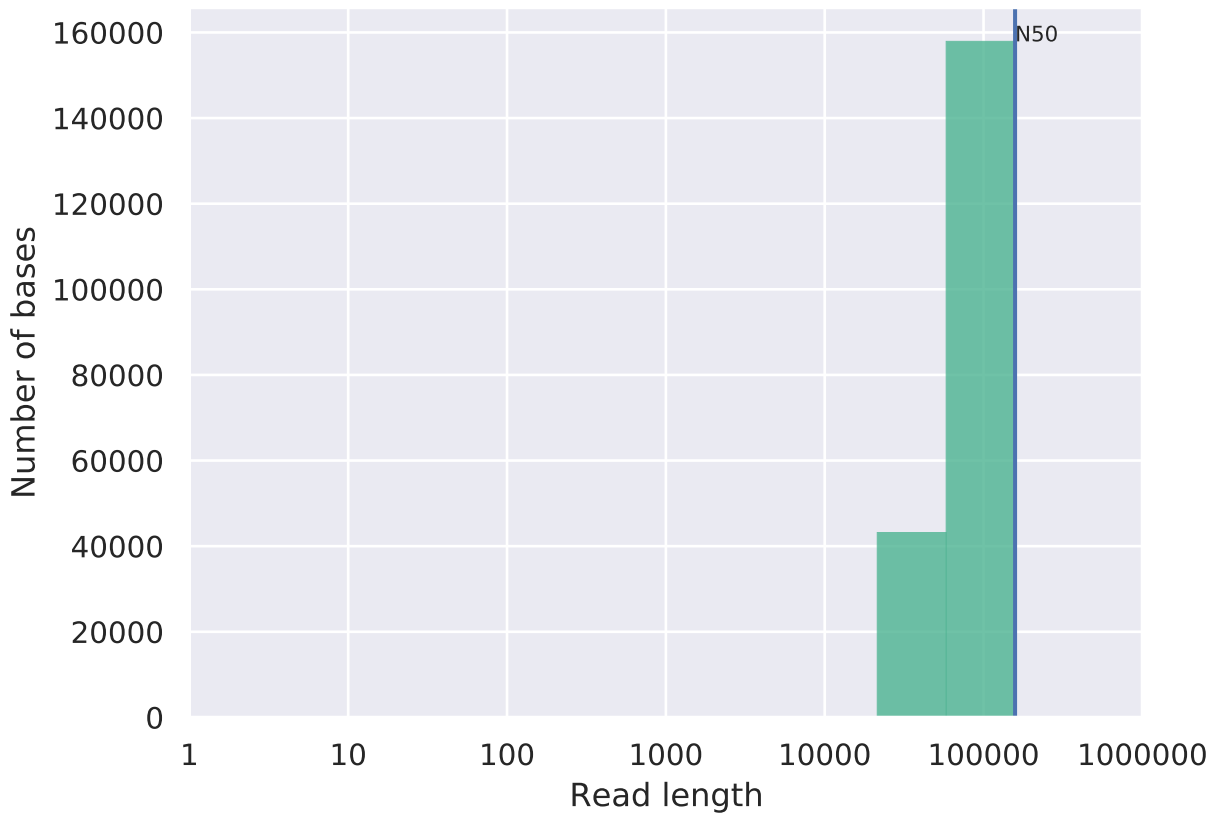

Supplement: Supplementary file 7 [file DataSheet_3.zip › SF1c/ccs999KIR7_18_5.contigs_MN167529_reports/ccs999KIR7_18_5.contigs_MN167529Weighted_LogTransformed_HistogramReadlength.pdf]

Weighted Histogram of read lengths

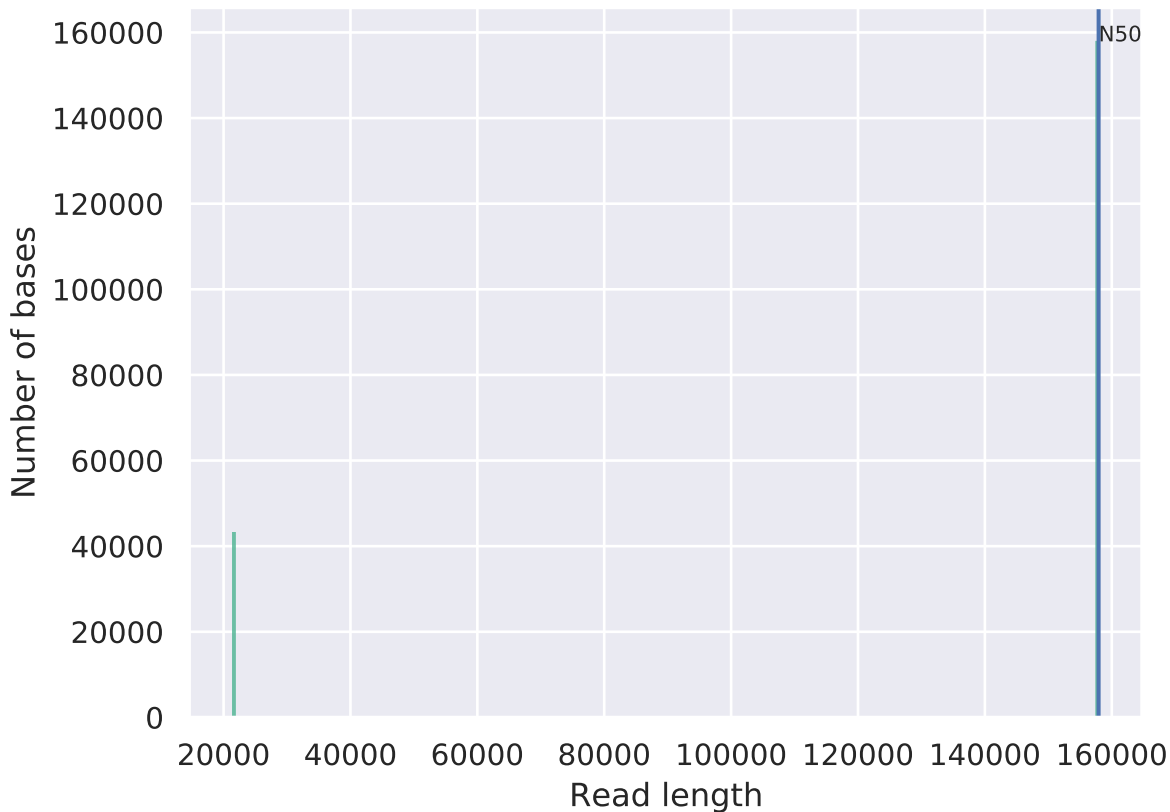

Supplement: Supplementary file 7 [file DataSheet_3.zip › SF1c/ccs999KIR7_18_5.contigs_MN167529_reports/ccs999KIR7_18_5.contigs_MN167529Weighted_HistogramReadlength.pdf]

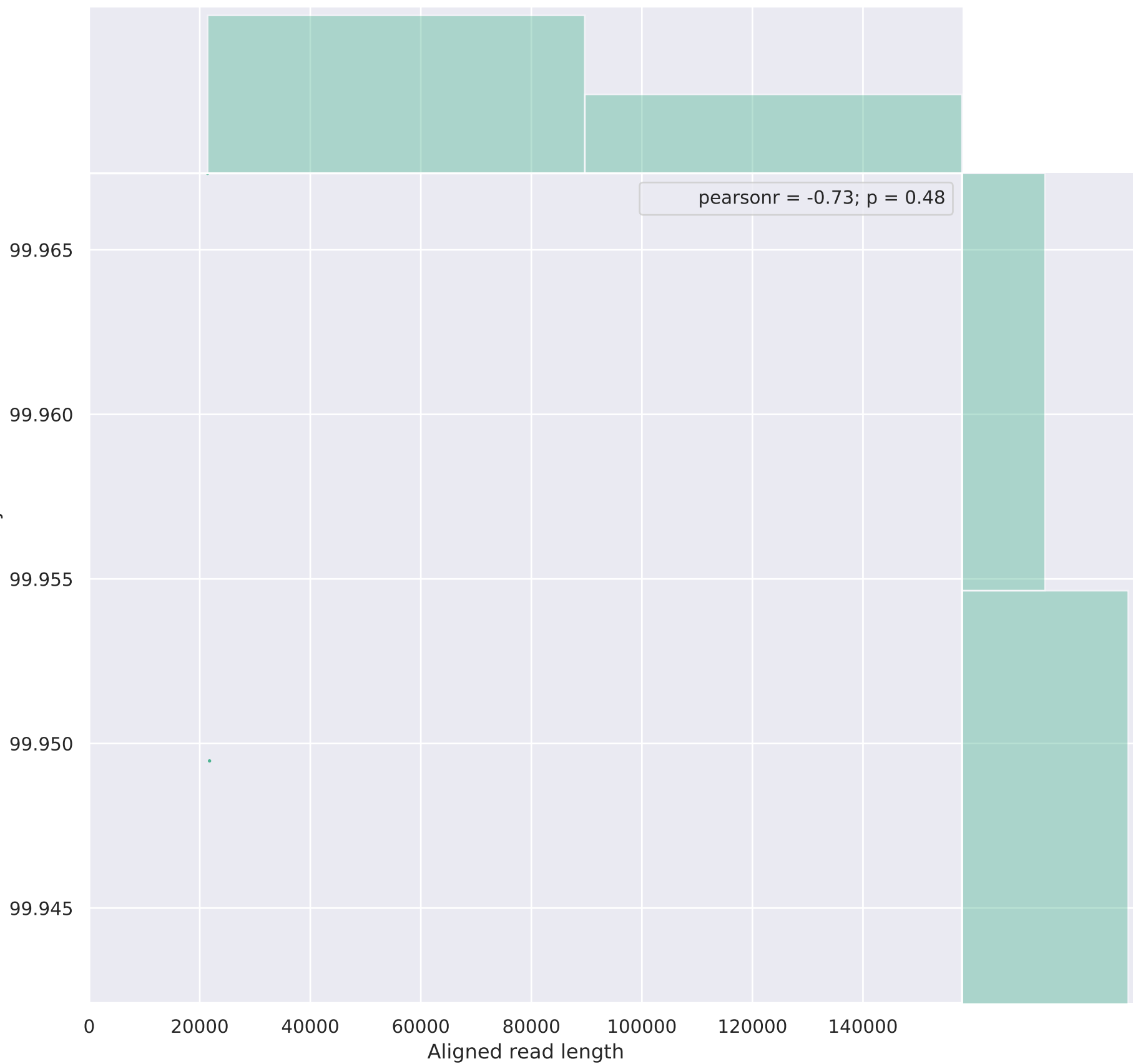

Supplement: Supplementary file 7 [file DataSheet_3.zip › SF1c/ccs999KIR7_18_5.contigs_MN167529_reports/ccs999KIR7_18_5.contigs_MN167529PercentIdentityvsAlignedReadLength_dot.pdf]

Histogram of read lengths

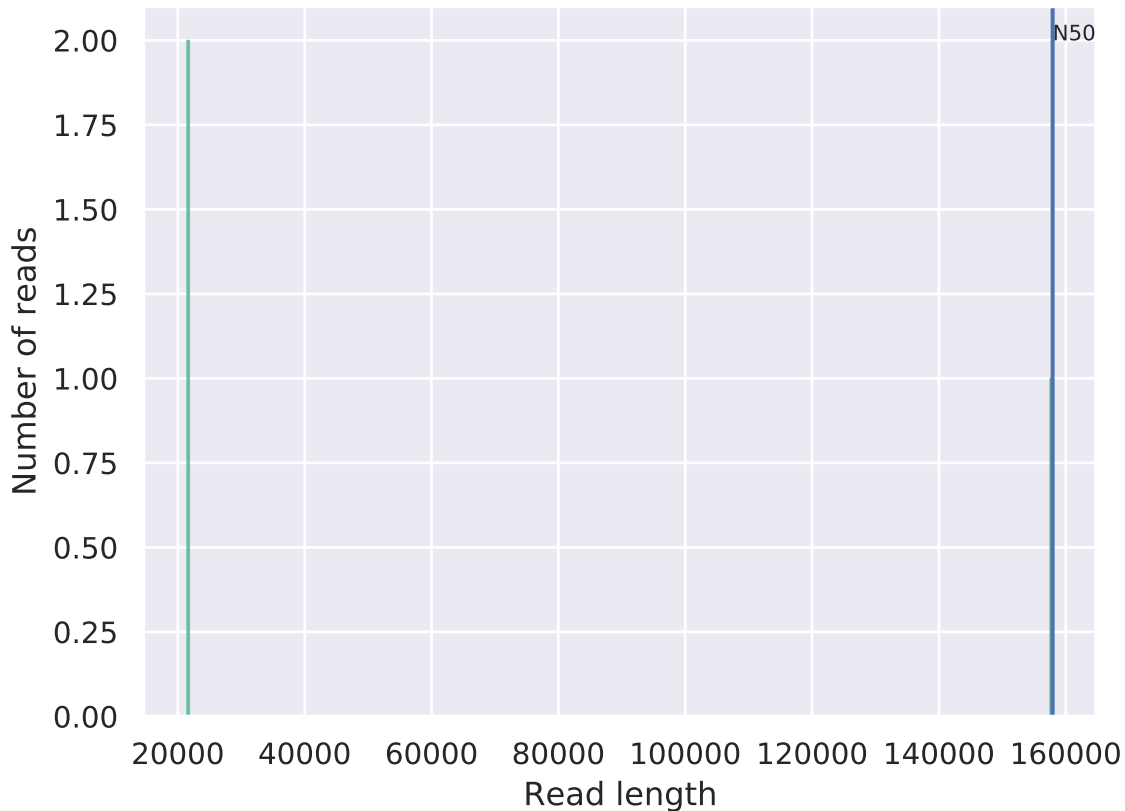

Supplement: Supplementary file 7 [file DataSheet_3.zip › SF1c/ccs999KIR7_18_5.contigs_MN167529_reports/ccs999KIR7_18_5.contigs_MN167529HistogramReadlength.pdf]

# Aligned read lengths vs Sequenced read length plot

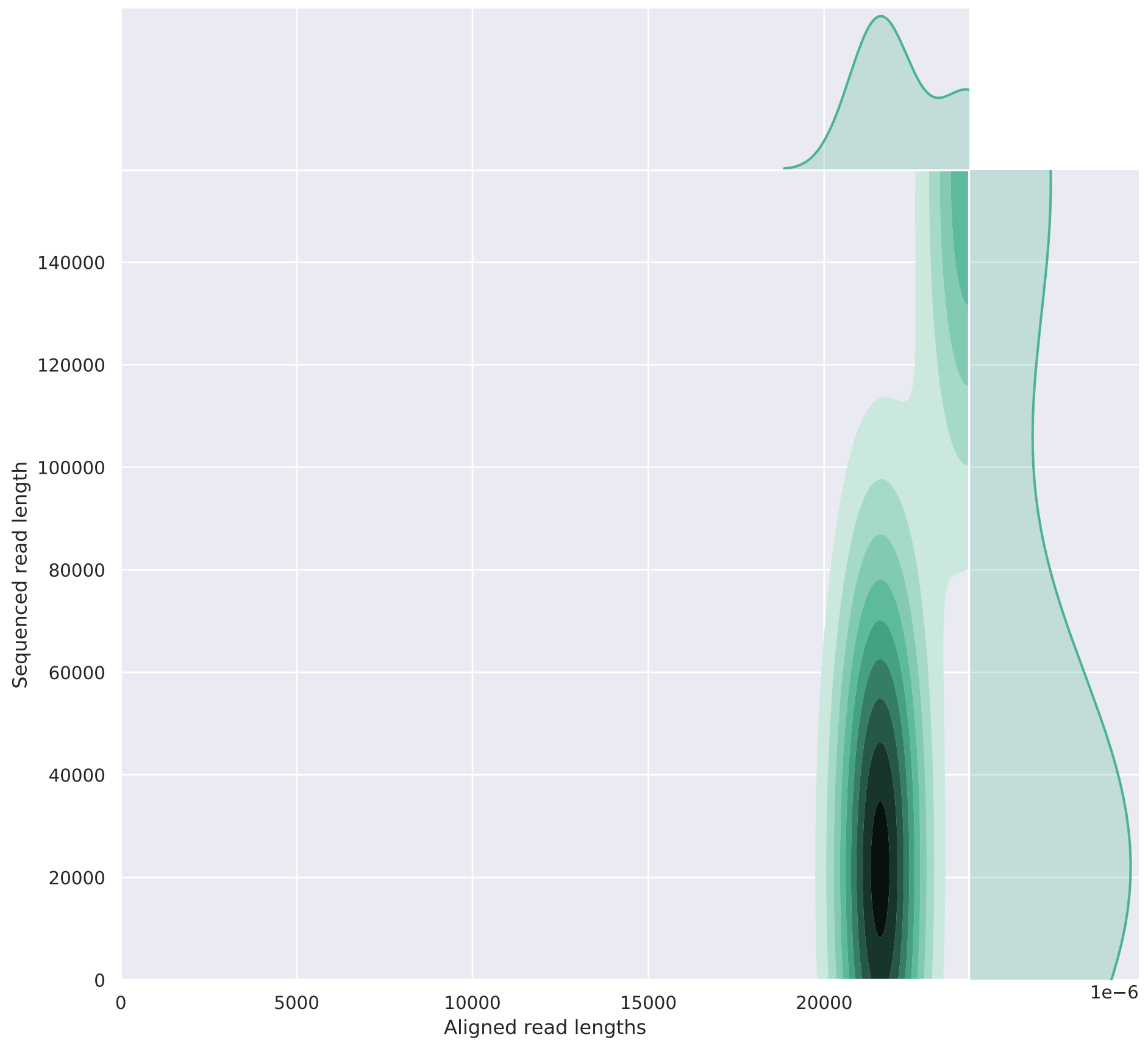

Supplement: Supplementary file 7 [file DataSheet_3.zip › SF1c/ccs999KIR7_18_5.contigs_MN167529_reports/ccs999KIR7_18_5.contigs_MN167529AlignedReadlengthvsSequencedReadLength_kde.pdf]

# Aligned read lengths vs Sequenced read length plot

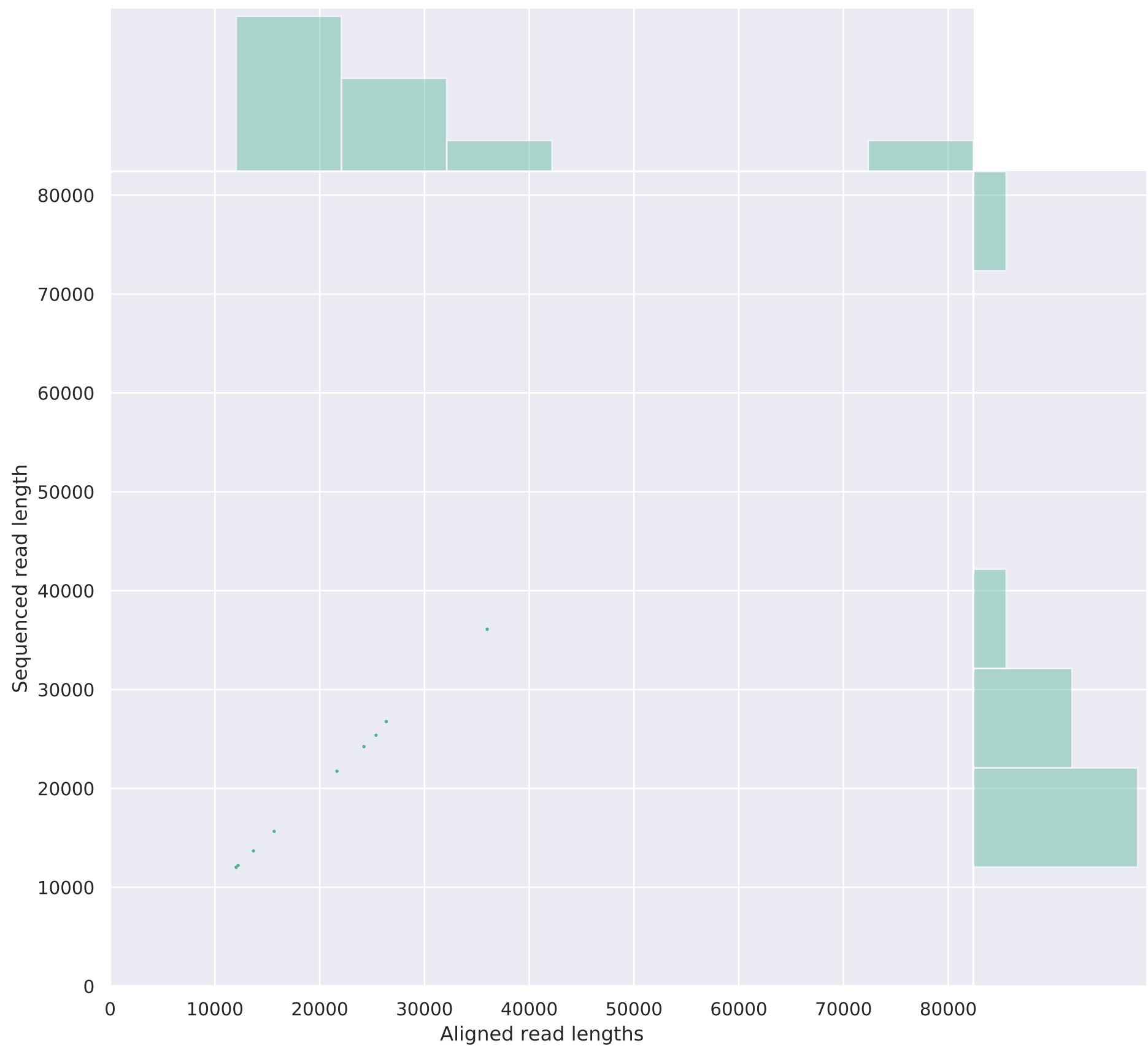

Supplement: Supplementary file 7 [file DataSheet_3.zip › SF1c/ccs999KIR7_18_5.contigs_MN167528_reports/ccs999KIR7_18_5.contigs_MN167528AlignedReadlengthvsSequencedReadLength_dot.pdf]

Histogram of read lengths after log transformation

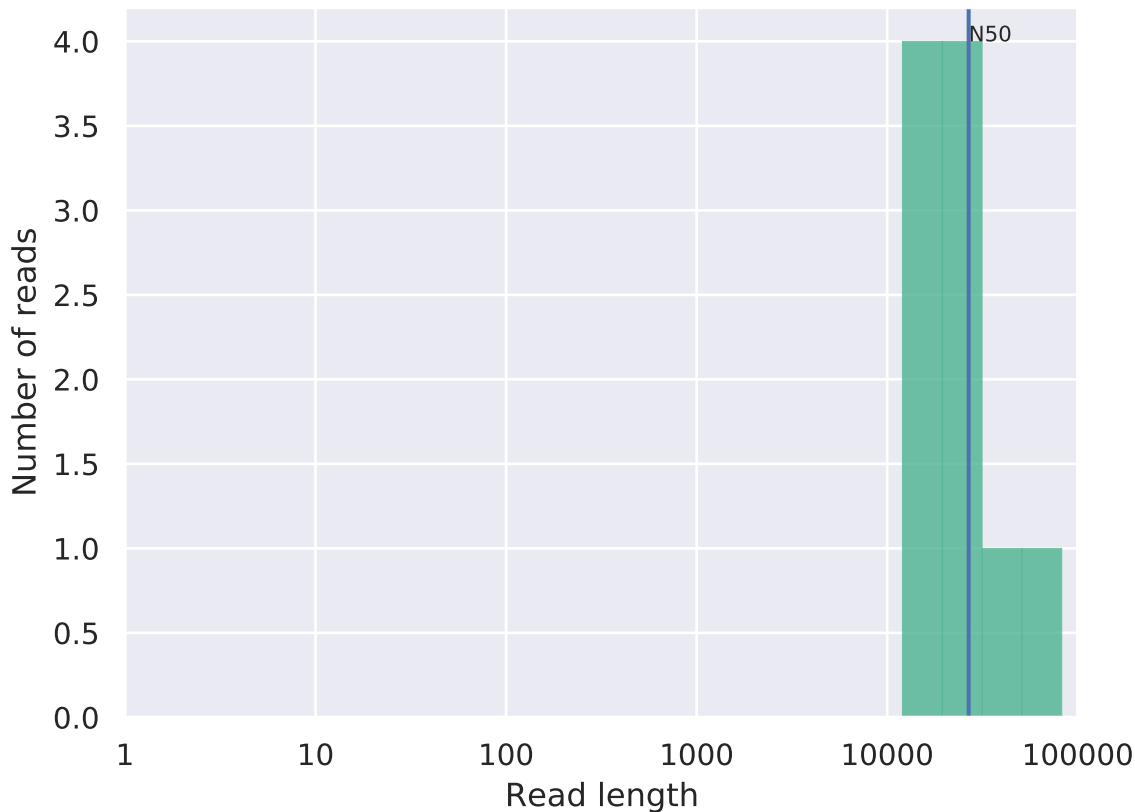

Supplement: Supplementary file 7 [file DataSheet_3.zip › SF1c/ccs999KIR7_18_5.contigs_MN167528_reports/ccs999KIR7_18_5.contigs_MN167528LogTransformed_HistogramReadlength.pdf]

# Aligned read length vs Percent identity plot

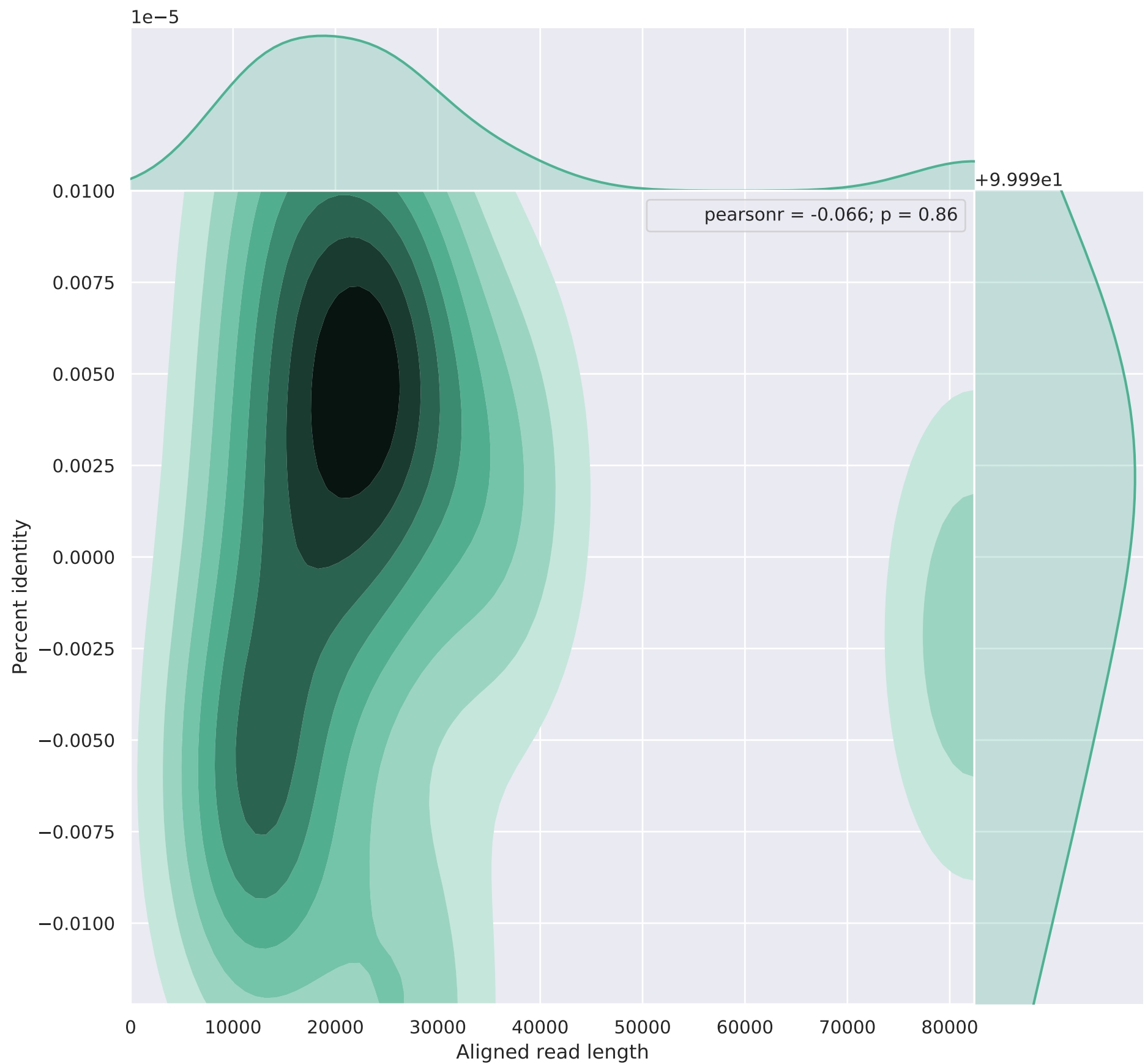

Supplement: Supplementary file 7 [file DataSheet_3.zip › SF1c/ccs999KIR7_18_5.contigs_MN167528_reports/ccs999KIR7_18_5.contigs_MN167528PercentIdentityvsAlignedReadLength_kde.pdf]

Weighted Histogram of read lengths

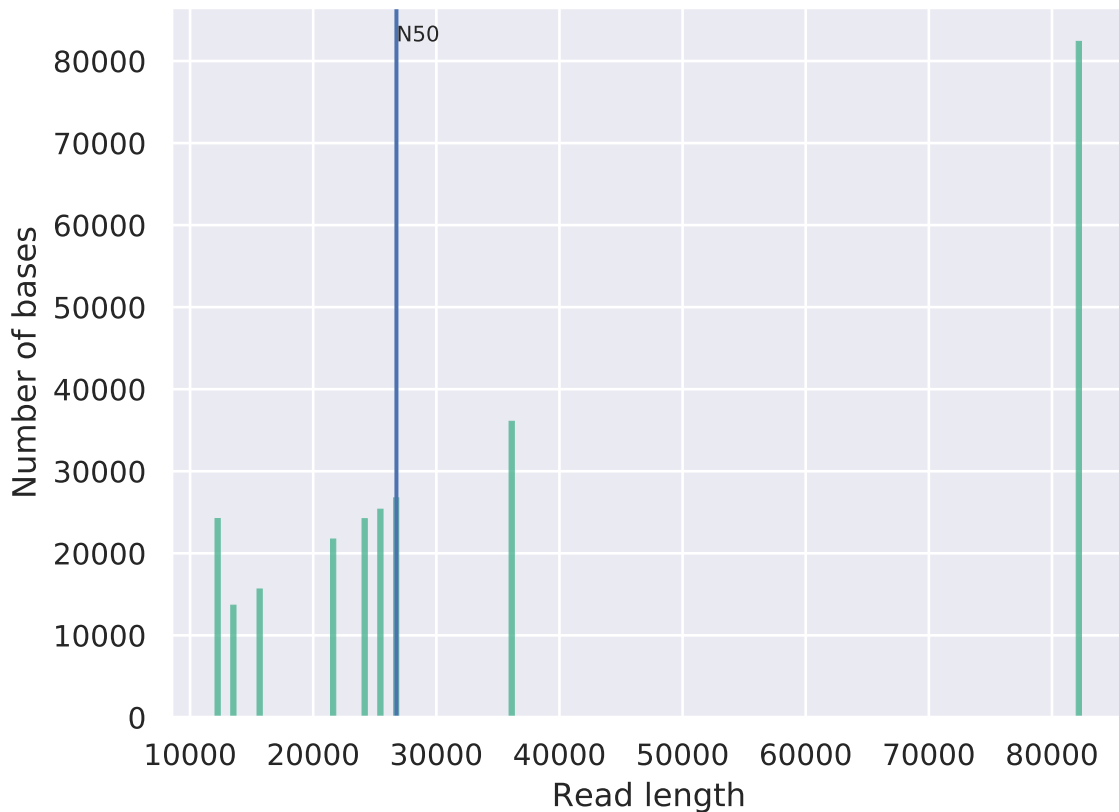

Supplement: Supplementary file 7 [file DataSheet_3.zip › SF1c/ccs999KIR7_18_5.contigs_MN167528_reports/ccs999KIR7_18_5.contigs_MN167528Weighted_HistogramReadlength.pdf]

Weighted Histogram of read lengths after log transformation

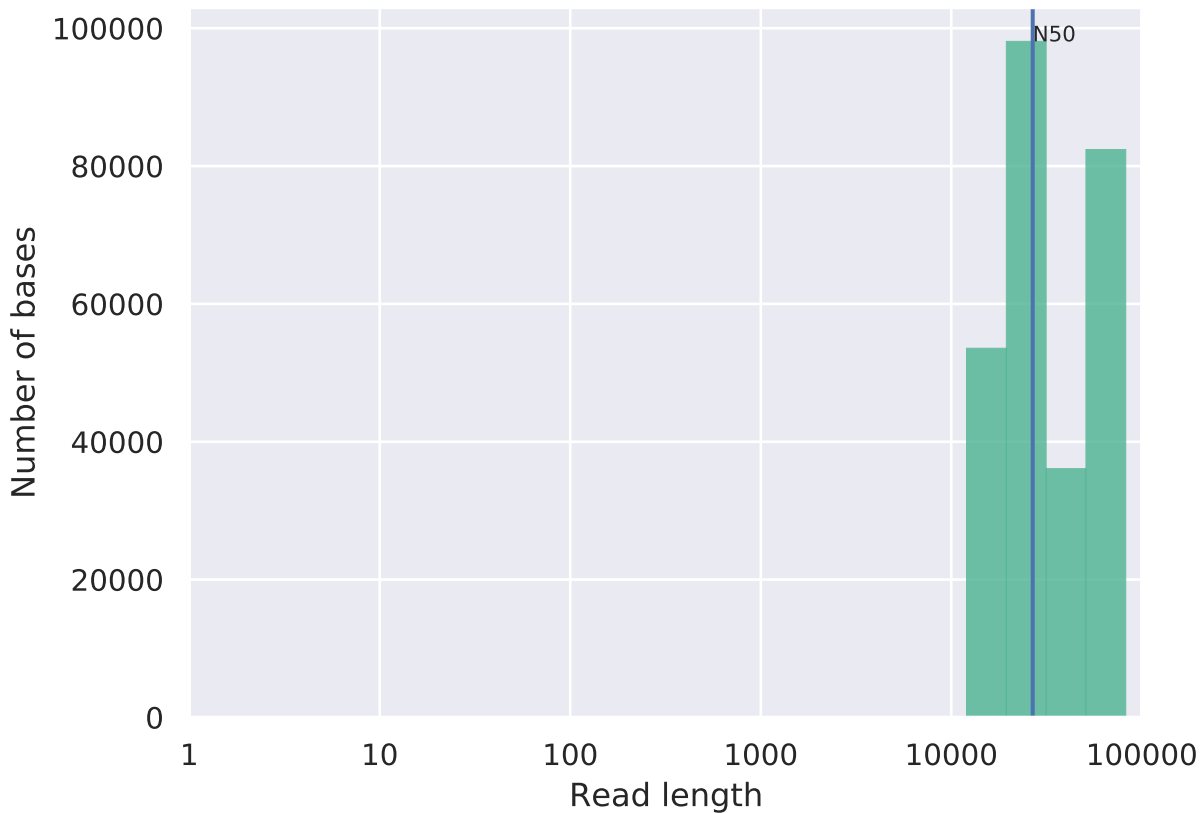

Supplement: Supplementary file 7 [file DataSheet_3.zip › SF1c/ccs999KIR7_18_5.contigs_MN167528_reports/ccs999KIR7_18_5.contigs_MN167528Weighted_LogTransformed_HistogramReadlength.pdf]

# Aligned read length vs Percent identity plot

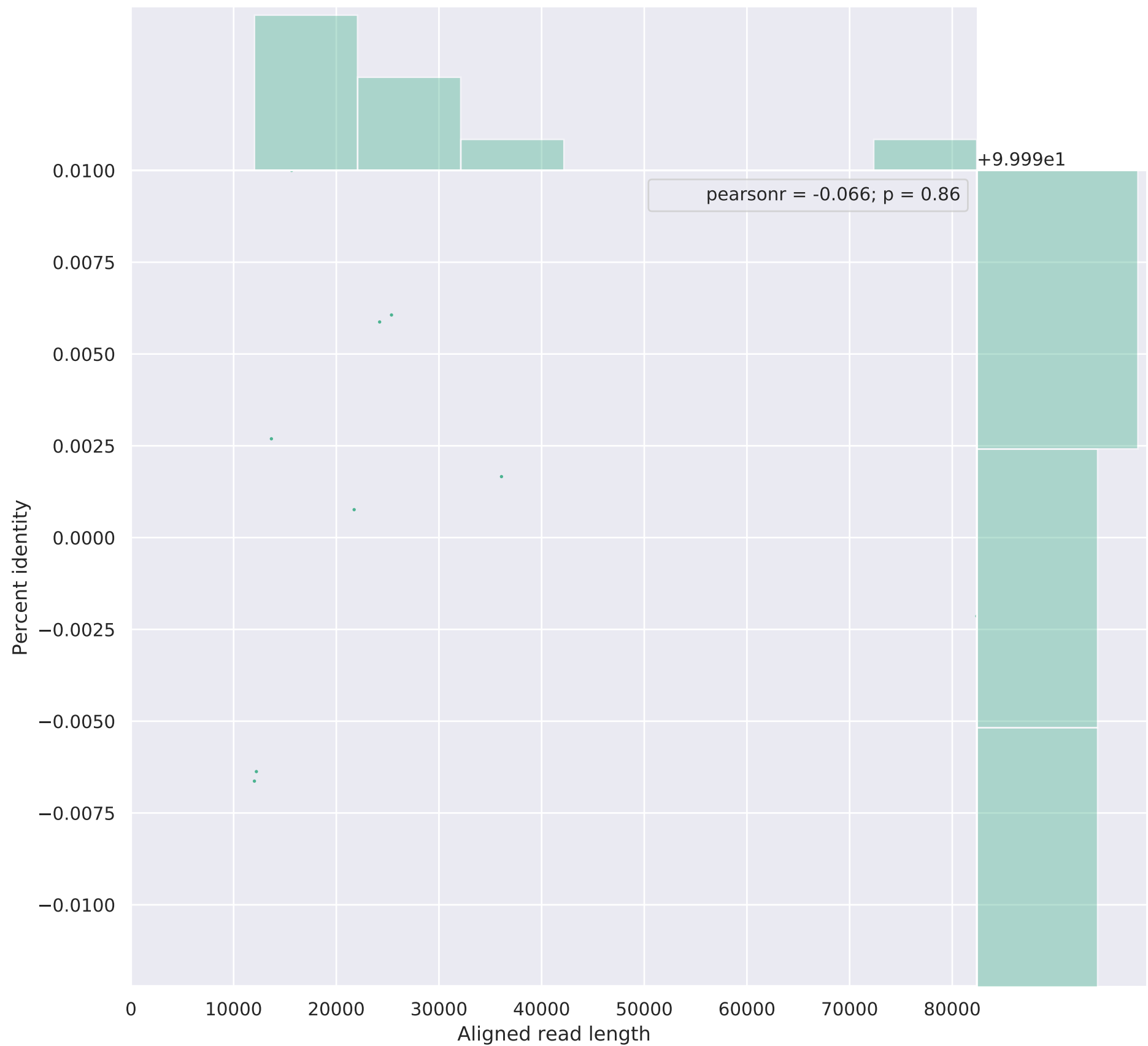

Supplement: Supplementary file 7 [file DataSheet_3.zip › SF1c/ccs999KIR7_18_5.contigs_MN167528_reports/ccs999KIR7_18_5.contigs_MN167528PercentIdentityvsAlignedReadLength_dot.pdf]

Histogram of read lengths

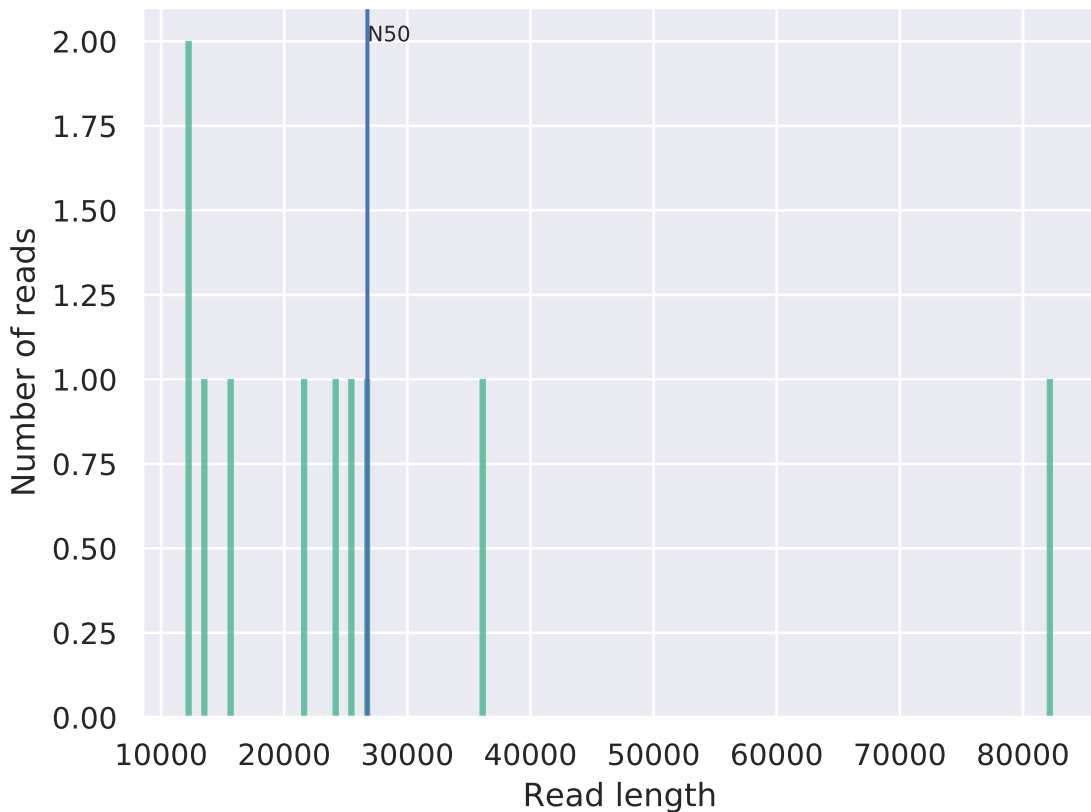

Supplement: Supplementary file 7 [file DataSheet_3.zip › SF1c/ccs999KIR7_18_5.contigs_MN167528_reports/ccs999KIR7_18_5.contigs_MN167528HistogramReadlength.pdf]

Yield by length

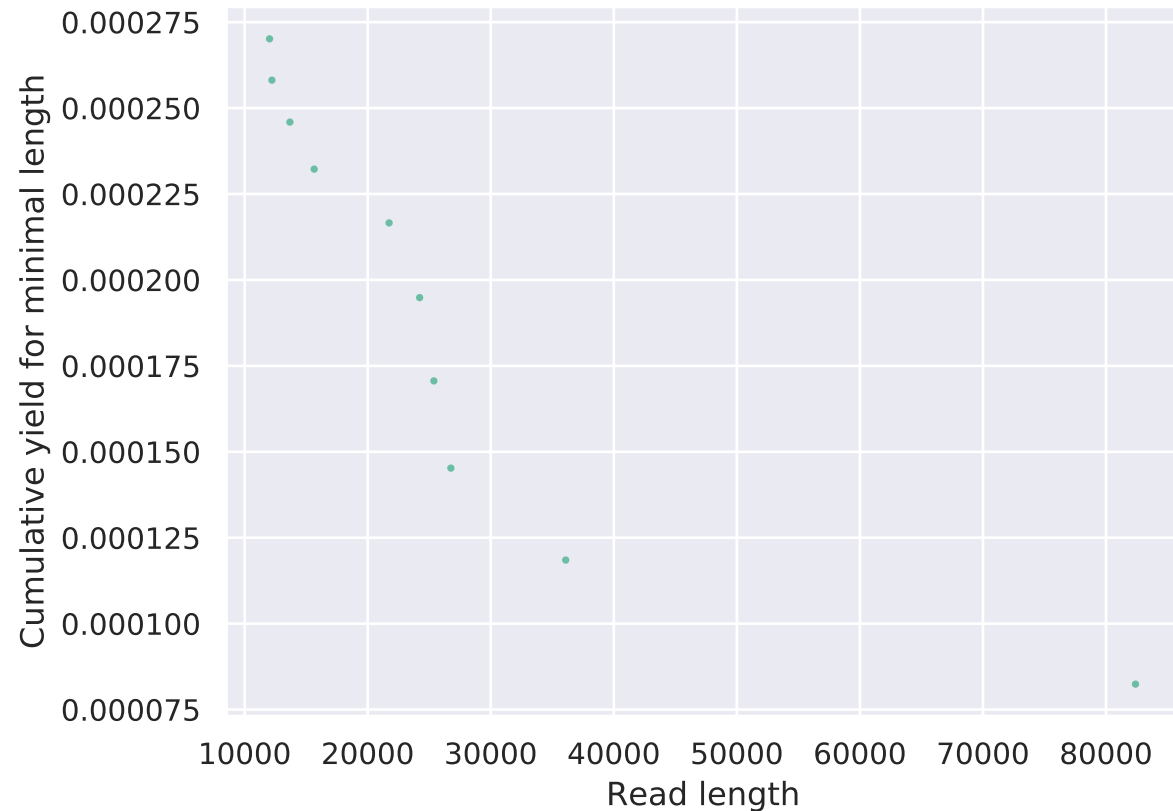

Supplement: Supplementary file 7 [file DataSheet_3.zip › SF1c/ccs999KIR7_18_5.contigs_MN167528_reports/ccs999KIR7_18_5.contigs_MN167528Yield_By_Length.pdf]

# Aligned read lengths vs Sequenced read length plot

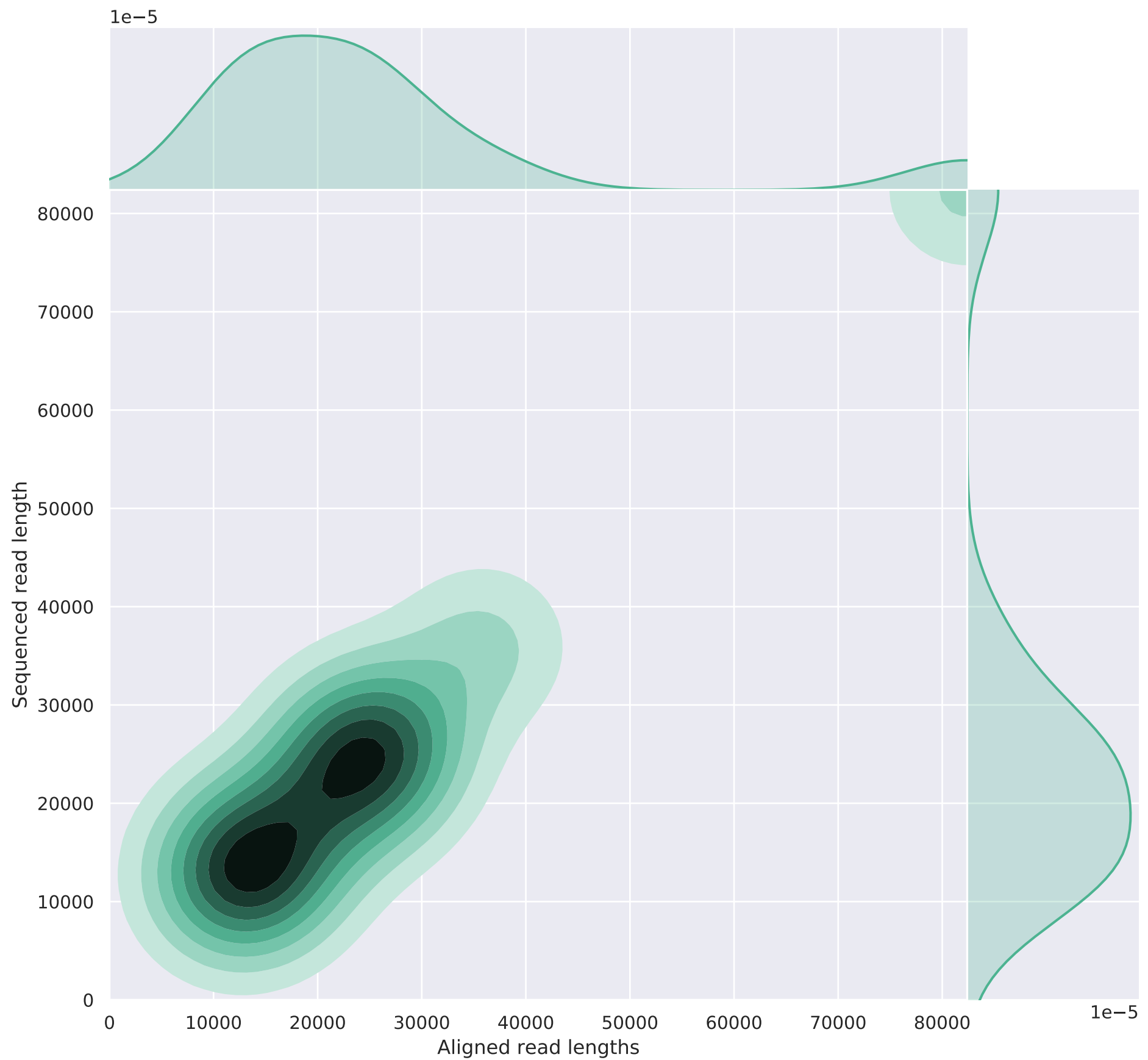

Supplement: Supplementary file 7 [file DataSheet_3.zip › SF1c/ccs999KIR7_18_5.contigs_MN167528_reports/ccs999KIR7_18_5.contigs_MN167528AlignedReadlengthvsSequencedReadLength_kde.pdf]

# Aligned read length vs Percent identity plot

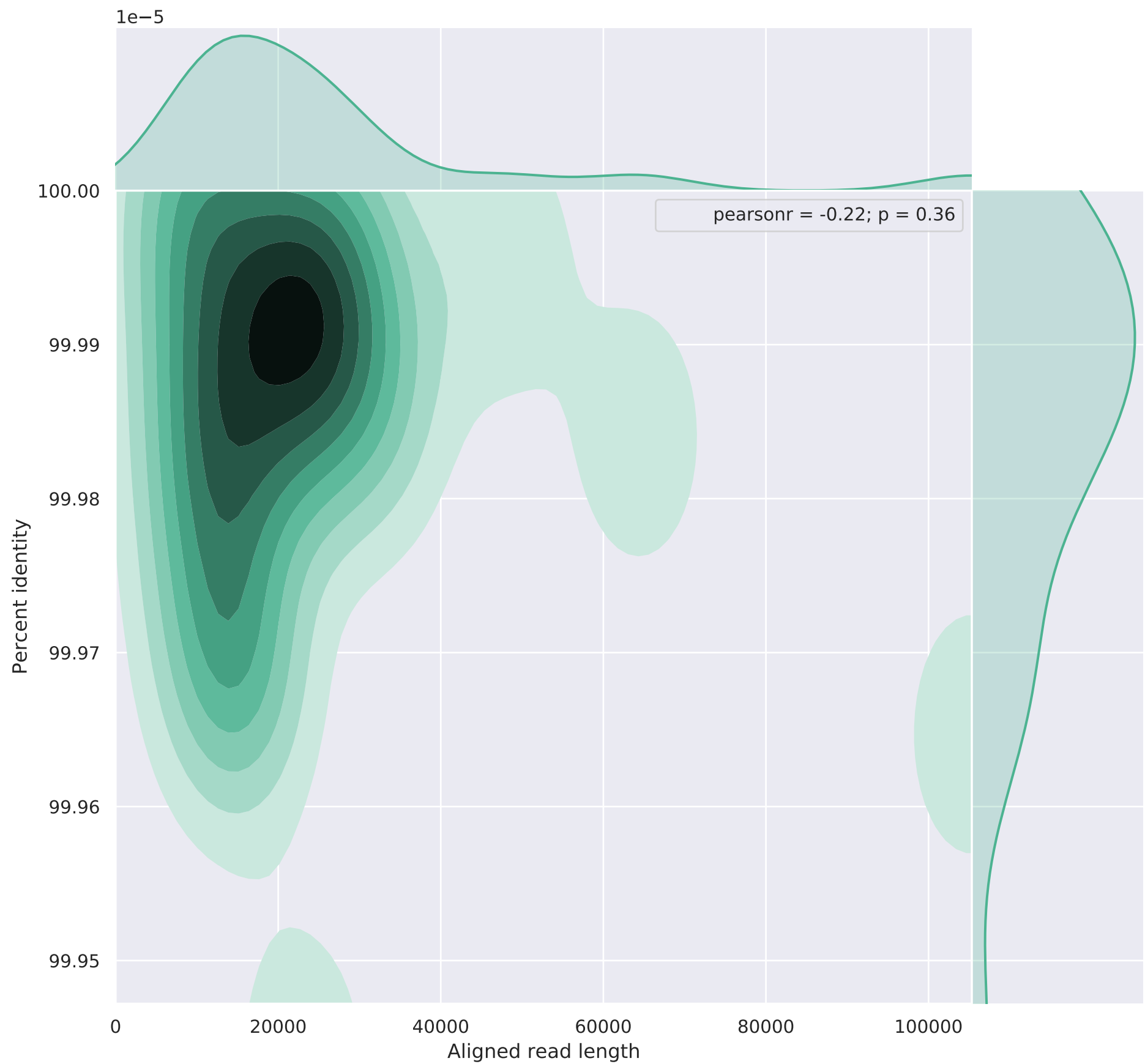

Supplement: Supplementary file 7 [file DataSheet_3.zip › SF1c/ccs999KIR7_18_6.contigs_MN167518_reports/ccs999KIR7_18_6.contigs_MN167518PercentIdentityvsAlignedReadLength_kde.pdf]

# Aligned read lengths vs Sequenced read length plot

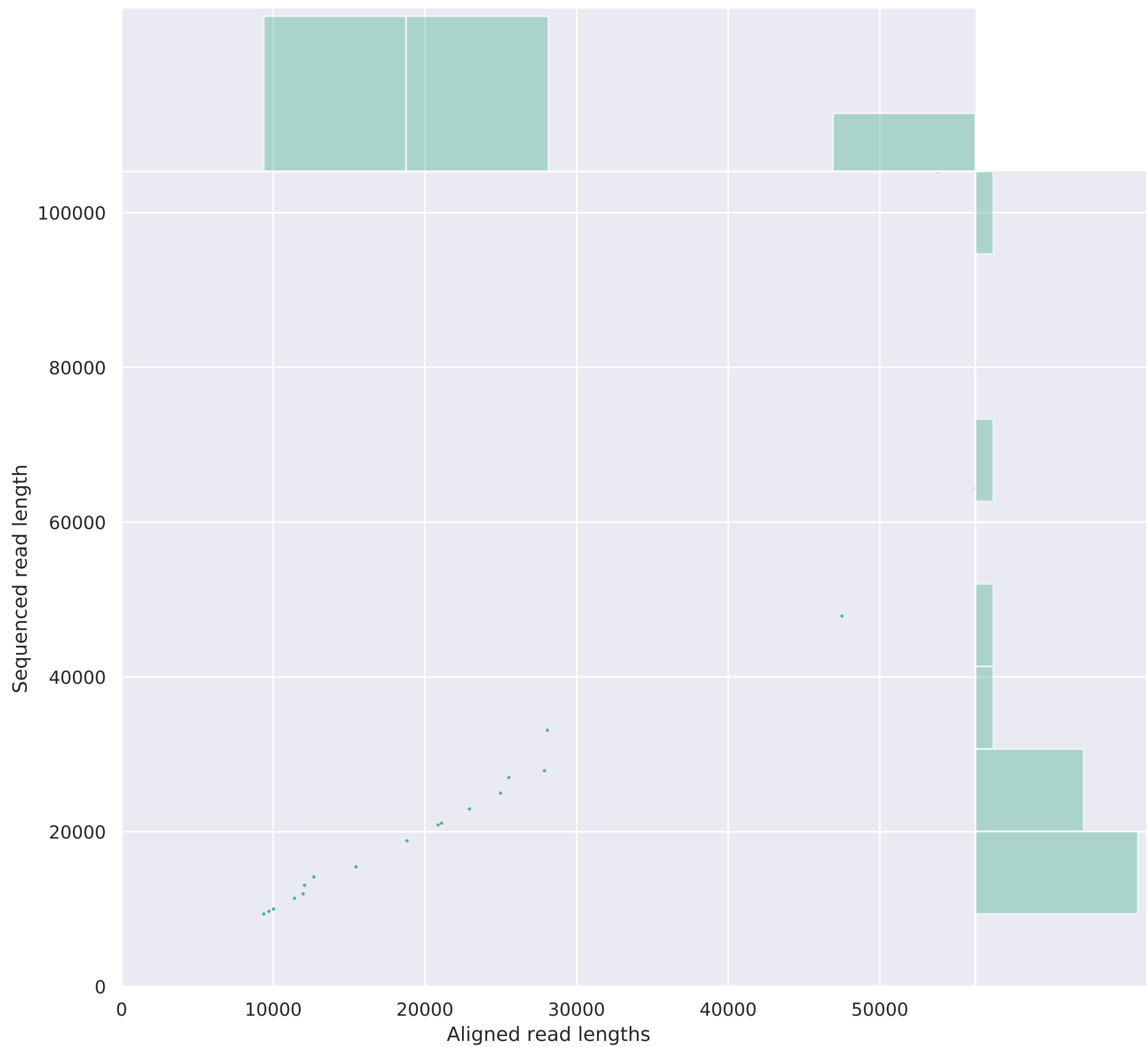

Supplement: Supplementary file 7 [file DataSheet_3.zip › SF1c/ccs999KIR7_18_6.contigs_MN167518_reports/ccs999KIR7_18_6.contigs_MN167518AlignedReadlengthvsSequencedReadLength_dot.pdf]

# Aligned read lengths vs Sequenced read length plot

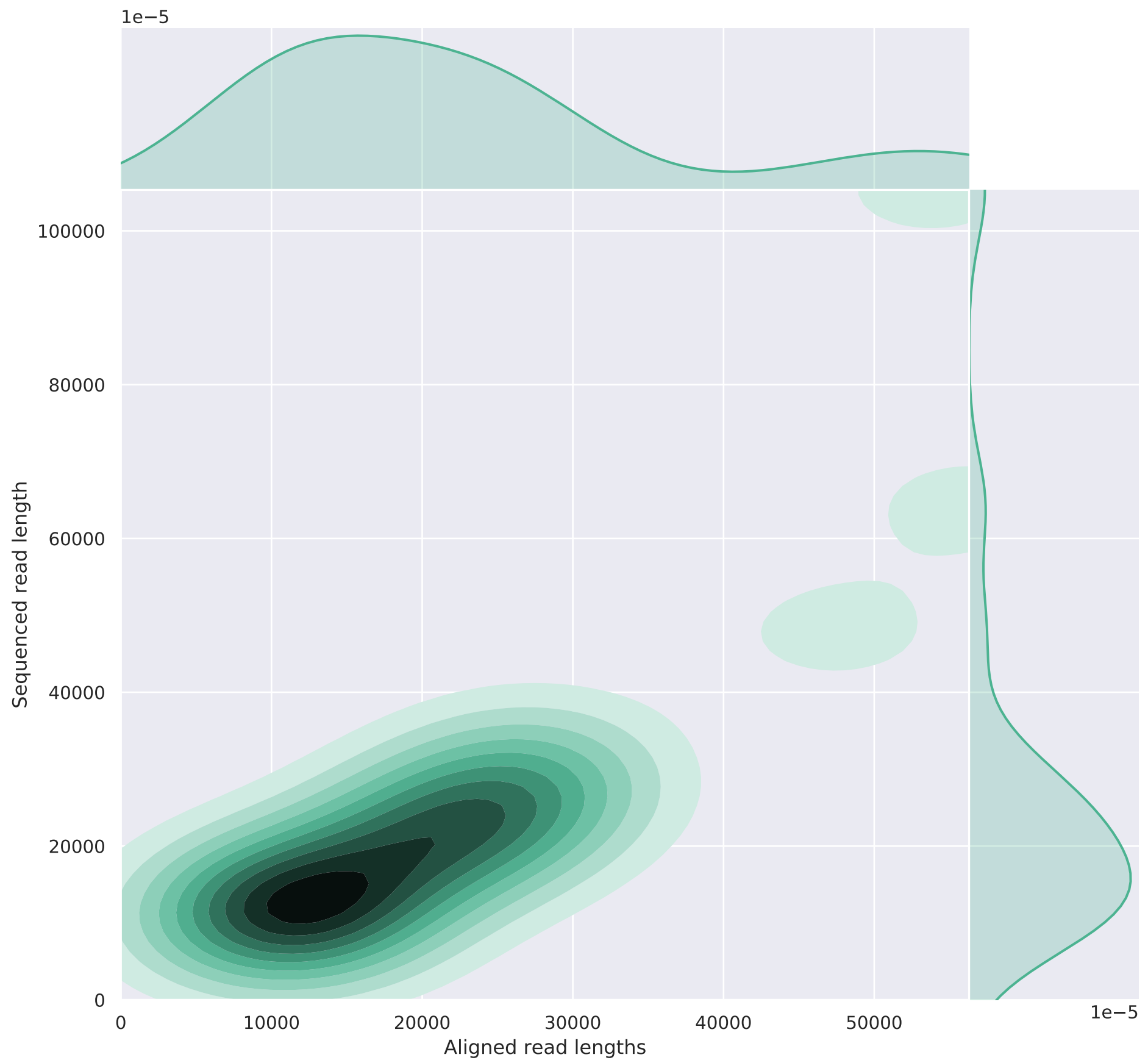

Supplement: Supplementary file 7 [file DataSheet_3.zip › SF1c/ccs999KIR7_18_6.contigs_MN167518_reports/ccs999KIR7_18_6.contigs_MN167518AlignedReadlengthvsSequencedReadLength_kde.pdf]

Histogram of read lengths

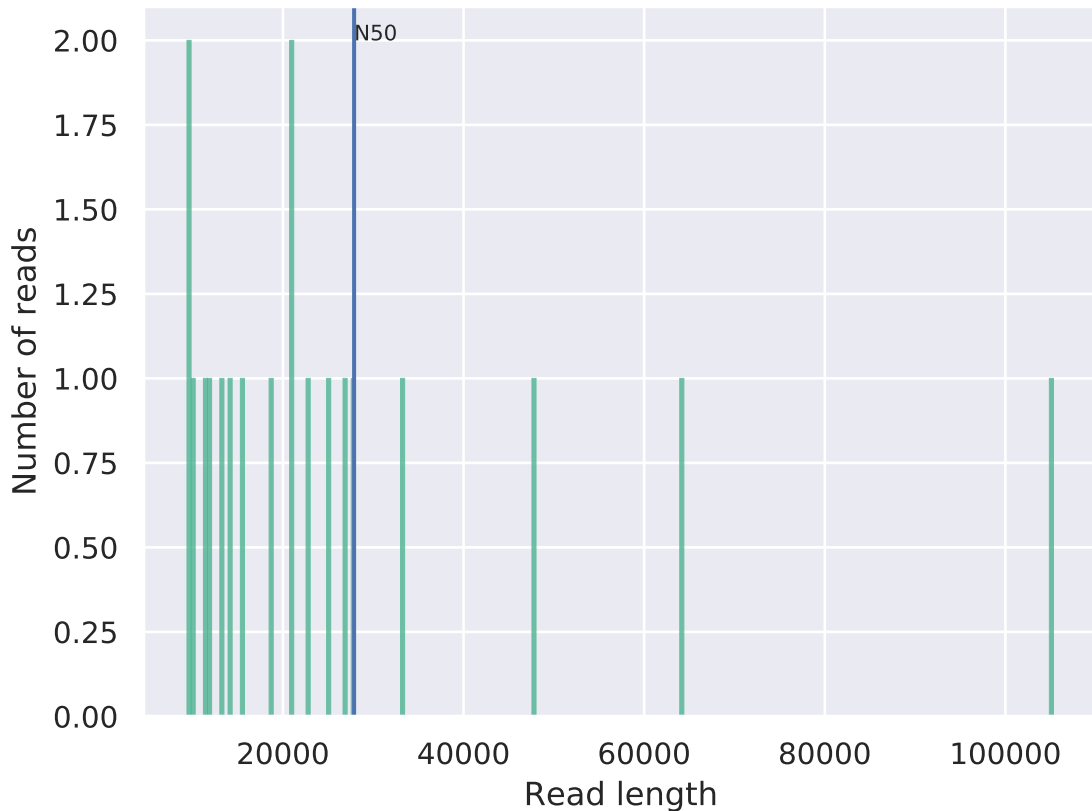

Supplement: Supplementary file 7 [file DataSheet_3.zip › SF1c/ccs999KIR7_18_6.contigs_MN167518_reports/ccs999KIR7_18_6.contigs_MN167518HistogramReadlength.pdf]

Weighted Histogram of read lengths

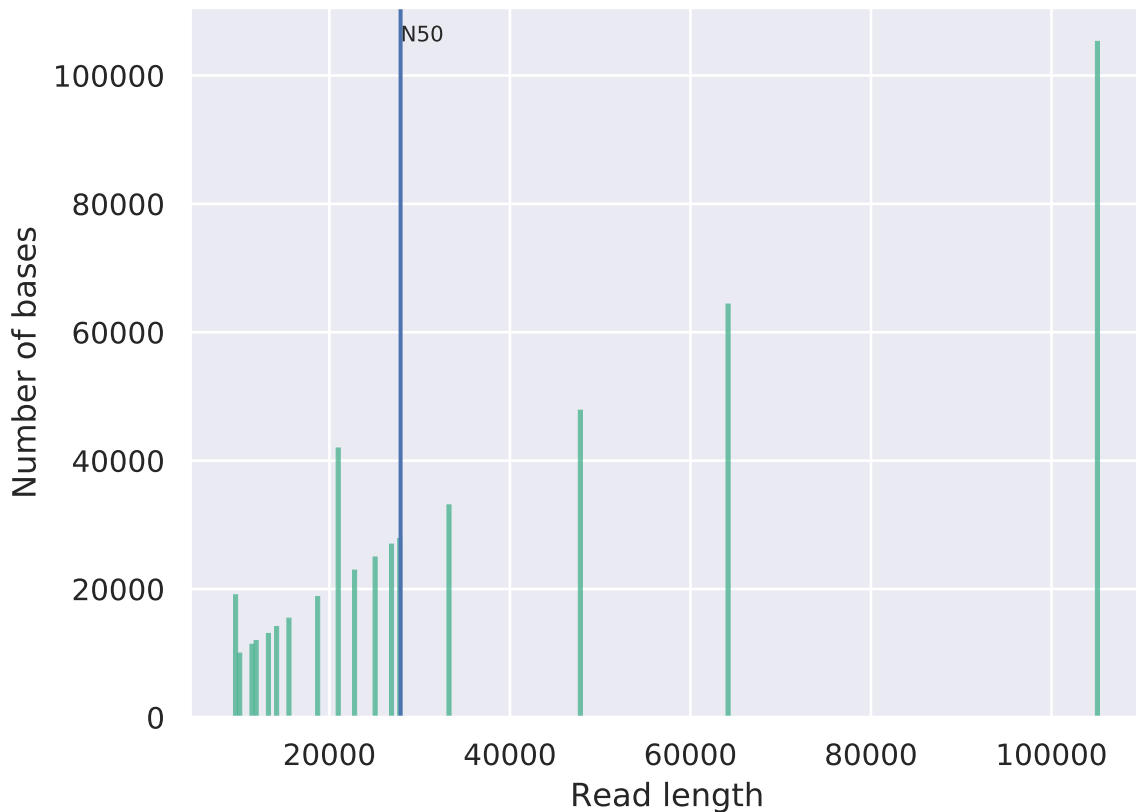

Supplement: Supplementary file 7 [file DataSheet_3.zip › SF1c/ccs999KIR7_18_6.contigs_MN167518_reports/ccs999KIR7_18_6.contigs_MN167518Weighted_HistogramReadlength.pdf]

# Aligned read length vs Percent identity plot

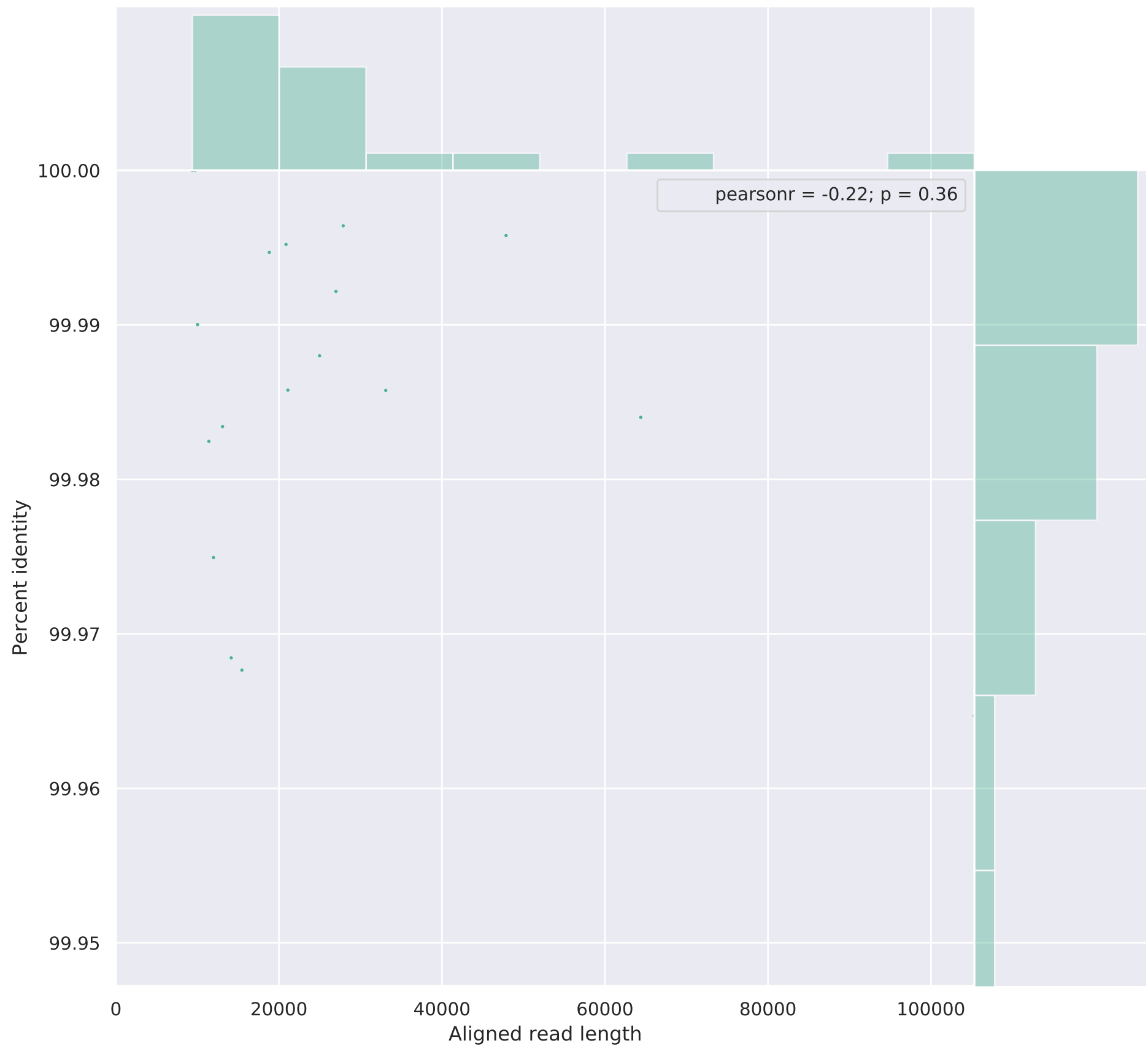

Supplement: Supplementary file 7 [file DataSheet_3.zip › SF1c/ccs999KIR7_18_6.contigs_MN167518_reports/ccs999KIR7_18_6.contigs_MN167518PercentIdentityvsAlignedReadLength_dot.pdf]

Yield by length

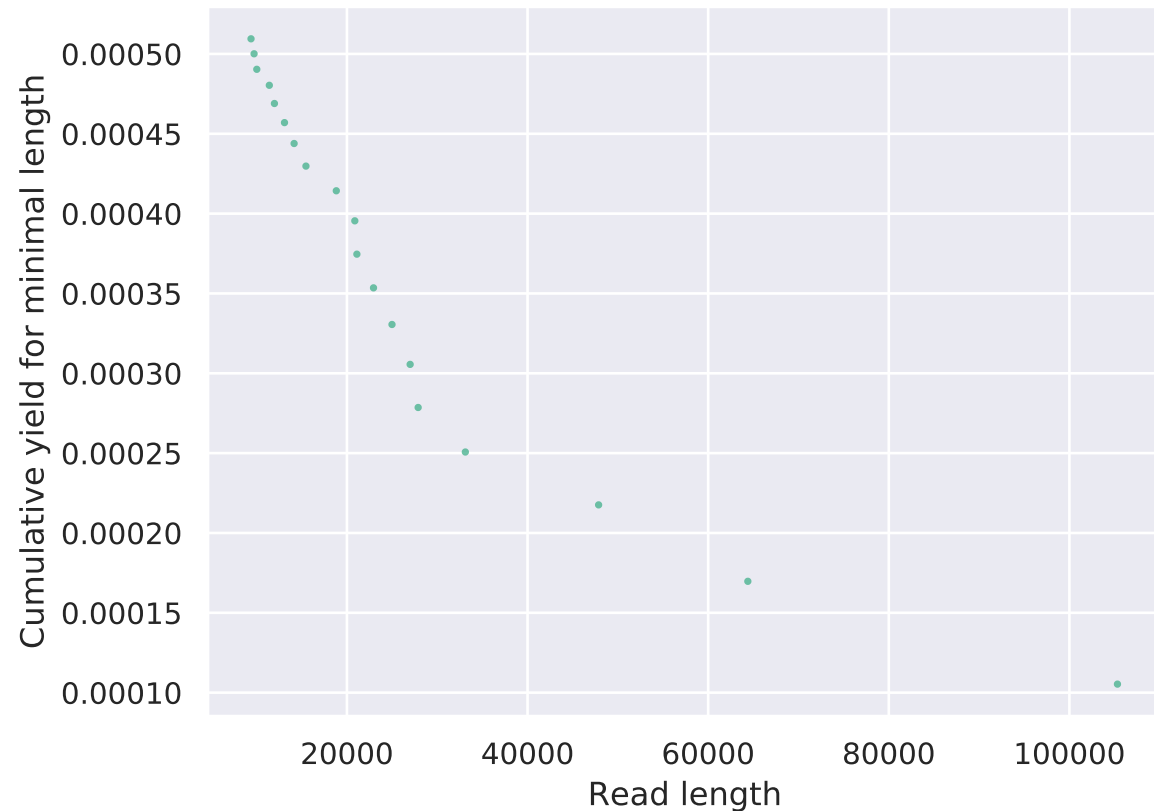

Supplement: Supplementary file 7 [file DataSheet_3.zip › SF1c/ccs999KIR7_18_6.contigs_MN167518_reports/ccs999KIR7_18_6.contigs_MN167518Yield_By_Length.pdf]

Weighted Histogram of read lengths after log transformation

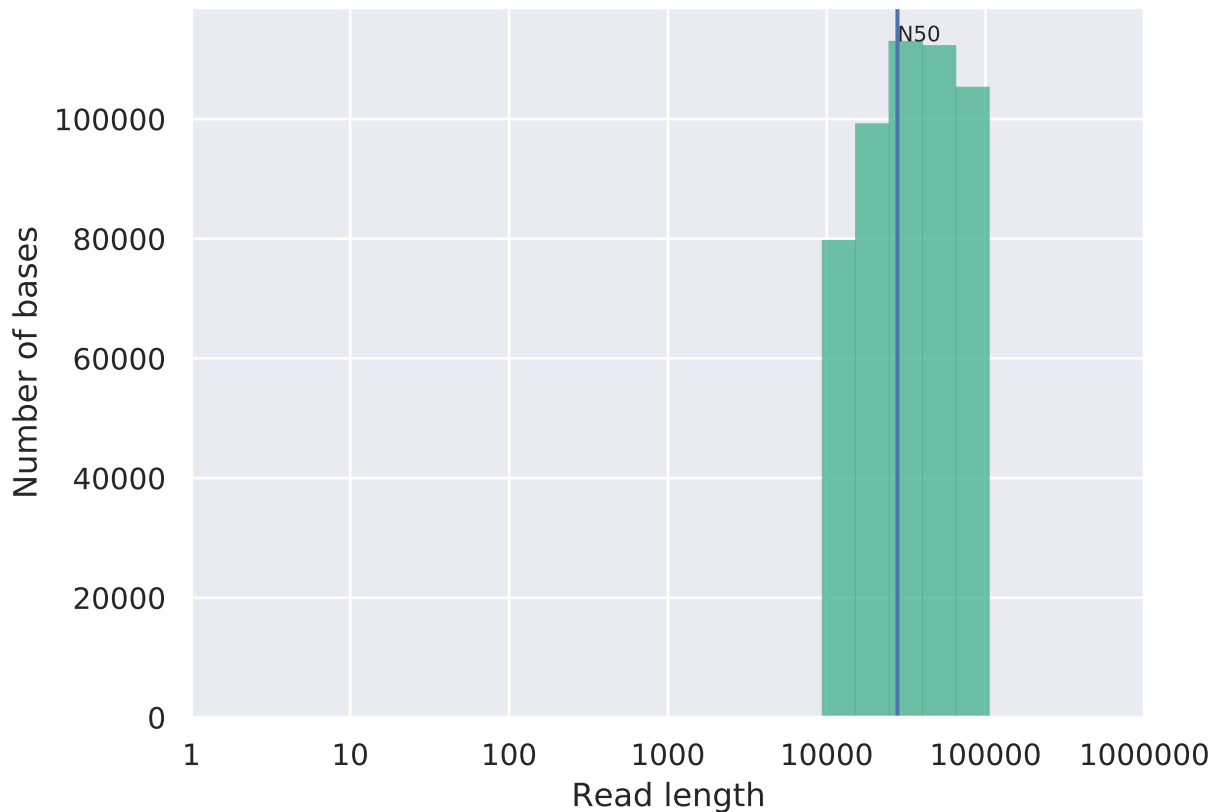

Supplement: Supplementary file 7 [file DataSheet_3.zip › SF1c/ccs999KIR7_18_6.contigs_MN167518_reports/ccs999KIR7_18_6.contigs_MN167518Weighted_LogTransformed_HistogramReadlength.pdf]

Histogram of read lengths after log transformation

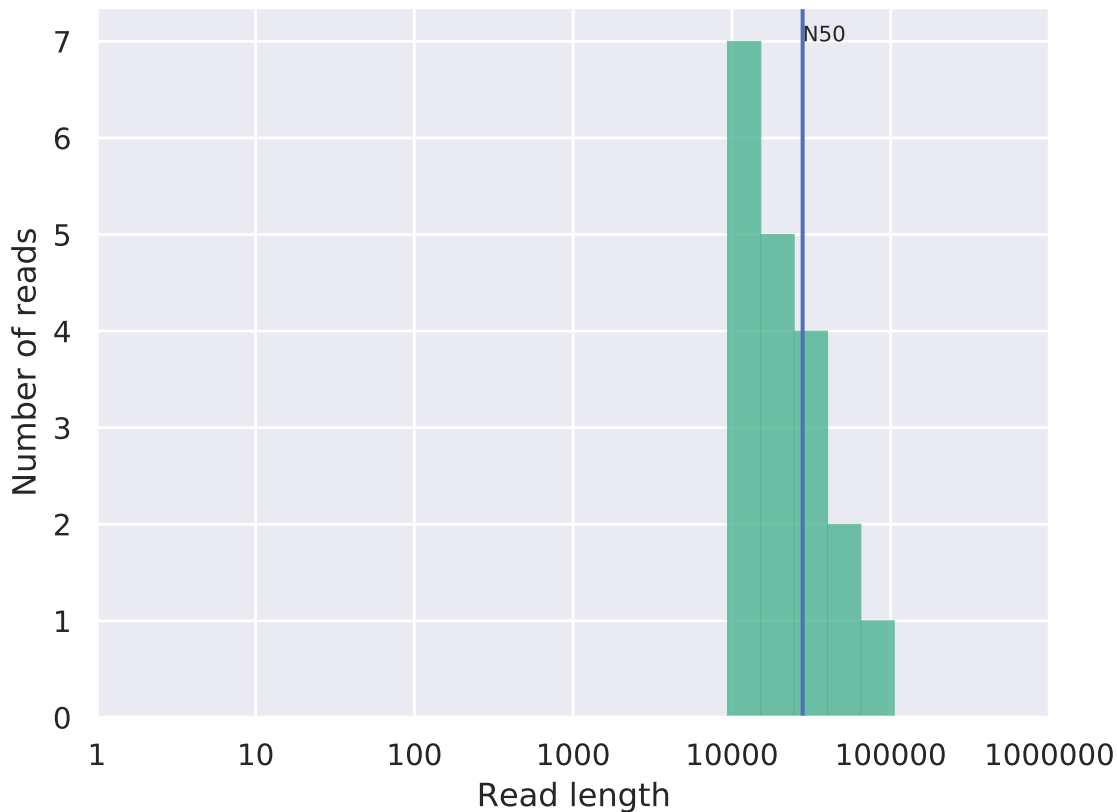

Supplement: Supplementary file 7 [file DataSheet_3.zip › SF1c/ccs999KIR7_18_6.contigs_MN167518_reports/ccs999KIR7_18_6.contigs_MN167518LogTransformed_HistogramReadlength.pdf]

# Aligned read length vs Percent identity plot

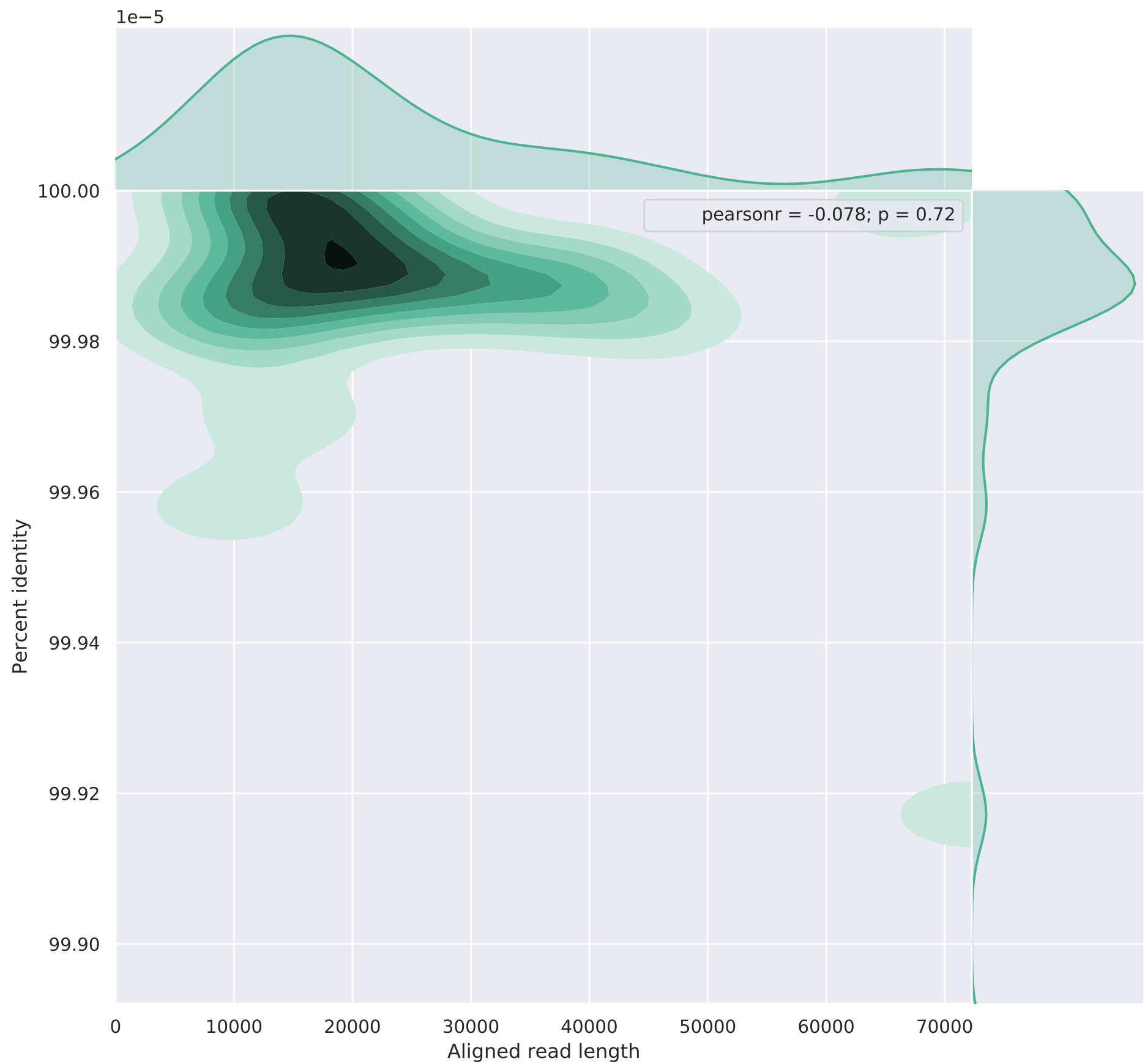

Supplement: Supplementary file 8 [file DataSheet_4.zip › SF1b/ccs999KIR7_18_3.contigs_MN167520_reports/ccs999KIR7_18_3.contigs_MN167520PercentIdentityvsAlignedReadLength_kde.pdf]

Weighted Histogram of read lengths

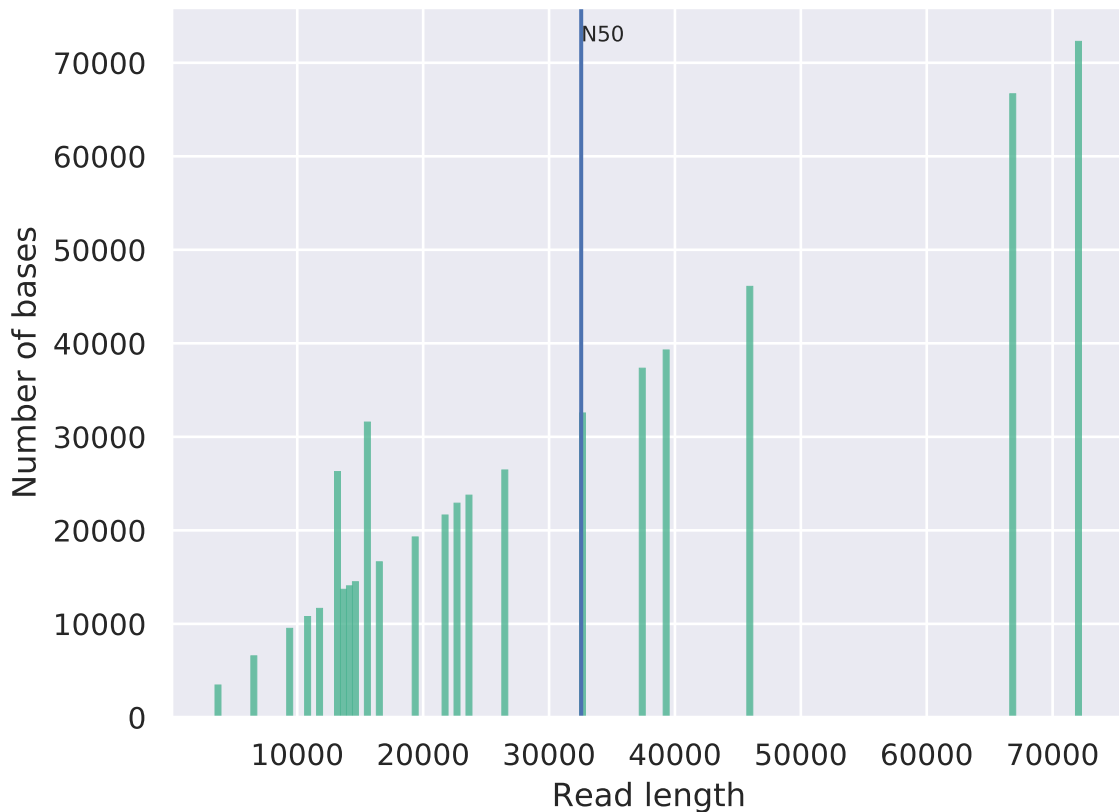

Supplement: Supplementary file 8 [file DataSheet_4.zip › SF1b/ccs999KIR7_18_3.contigs_MN167520_reports/ccs999KIR7_18_3.contigs_MN167520Weighted_HistogramReadlength.pdf]

# Aligned read lengths vs Sequenced read length plot

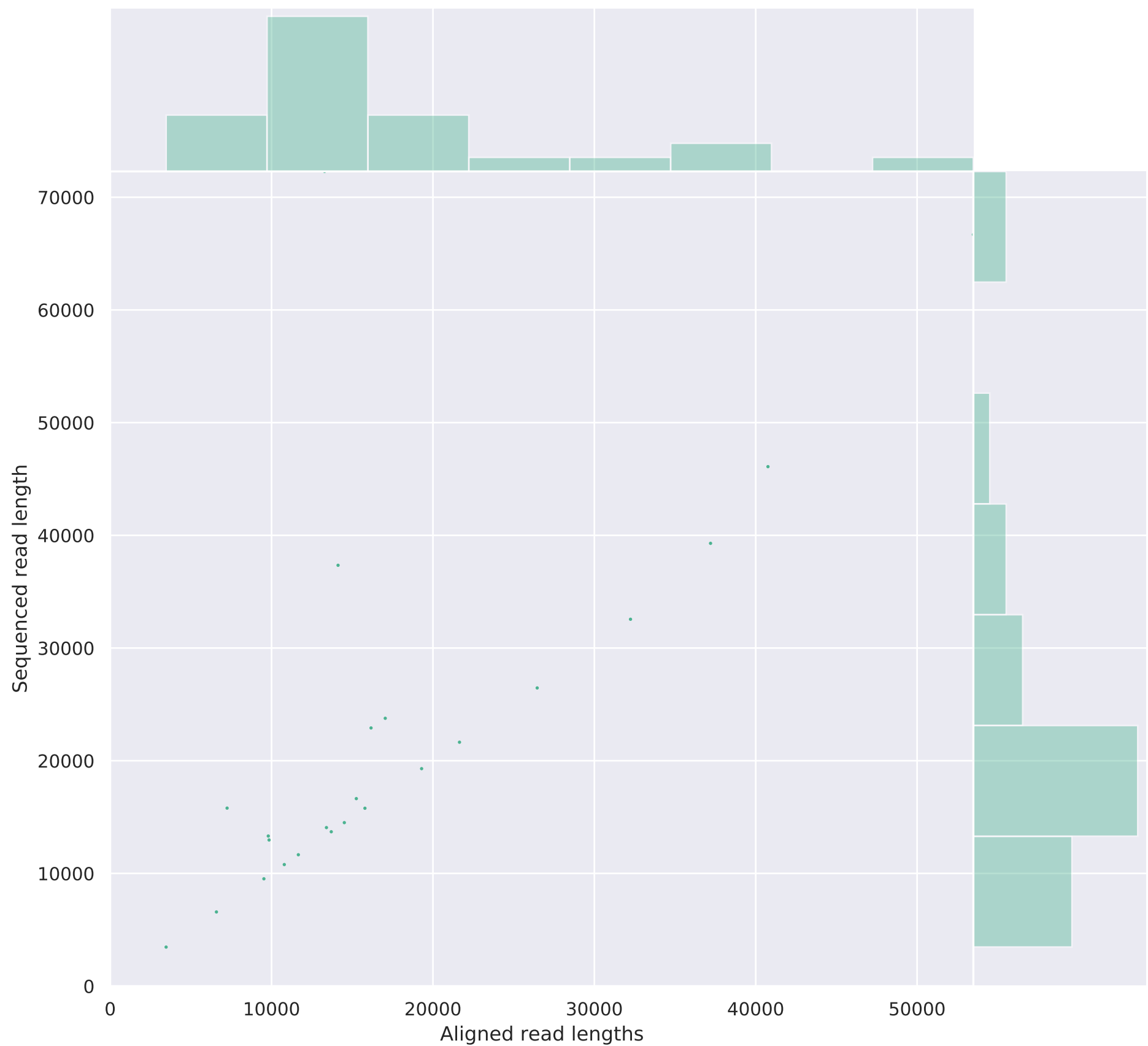

Supplement: Supplementary file 8 [file DataSheet_4.zip › SF1b/ccs999KIR7_18_3.contigs_MN167520_reports/ccs999KIR7_18_3.contigs_MN167520AlignedReadlengthvsSequencedReadLength_dot.pdf]

Histogram of read lengths after log transformation

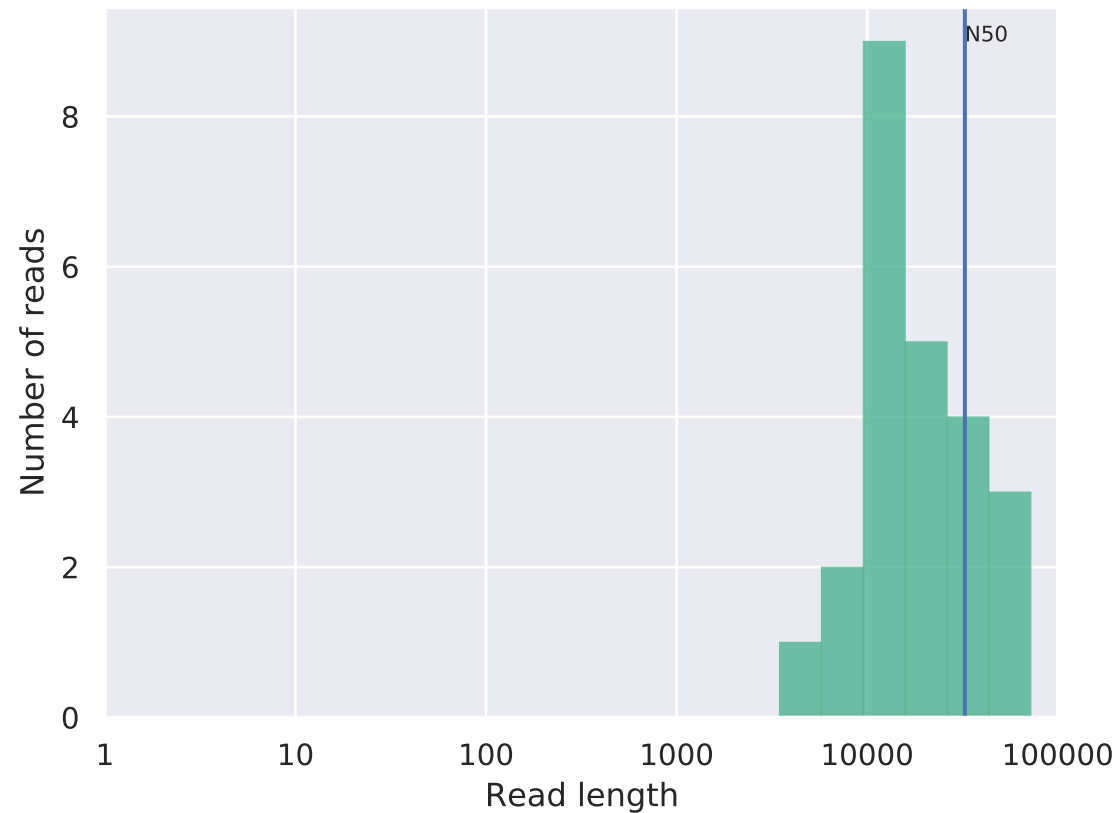

Supplement: Supplementary file 8 [file DataSheet_4.zip › SF1b/ccs999KIR7_18_3.contigs_MN167520_reports/ccs999KIR7_18_3.contigs_MN167520LogTransformed_HistogramReadlength.pdf]

# Aligned read lengths vs Sequenced read length plot

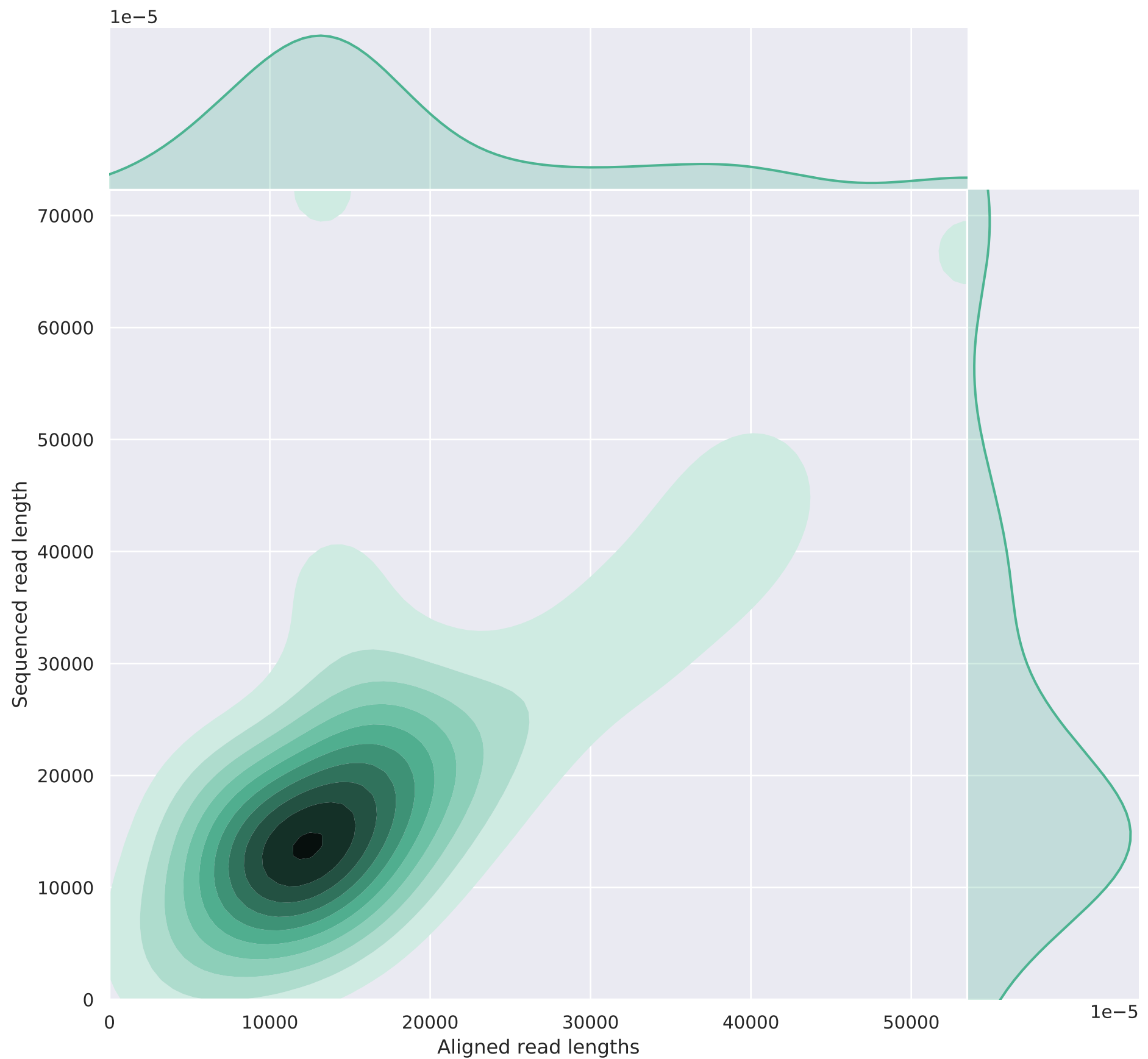

Supplement: Supplementary file 8 [file DataSheet_4.zip › SF1b/ccs999KIR7_18_3.contigs_MN167520_reports/ccs999KIR7_18_3.contigs_MN167520AlignedReadlengthvsSequencedReadLength_kde.pdf]

Weighted Histogram of read lengths after log transformation

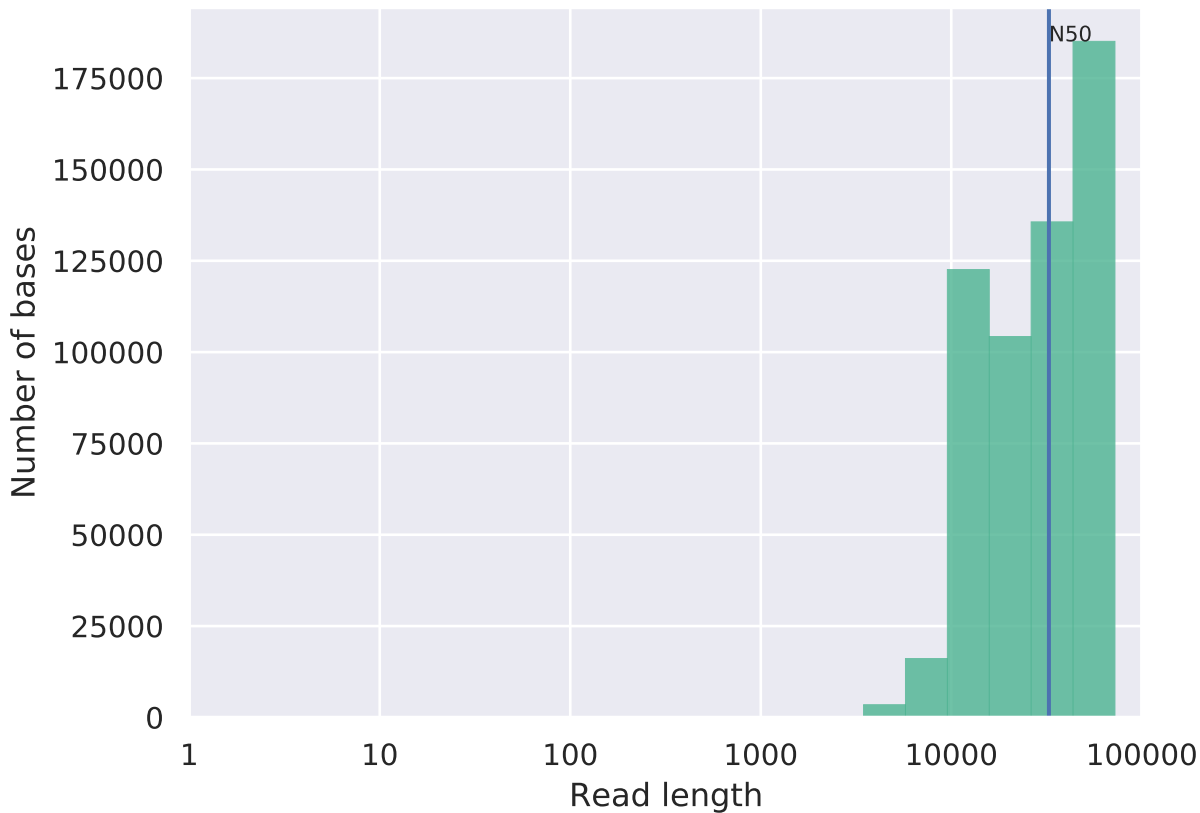

Supplement: Supplementary file 8 [file DataSheet_4.zip › SF1b/ccs999KIR7_18_3.contigs_MN167520_reports/ccs999KIR7_18_3.contigs_MN167520Weighted_LogTransformed_HistogramReadlength.pdf]

Histogram of read lengths

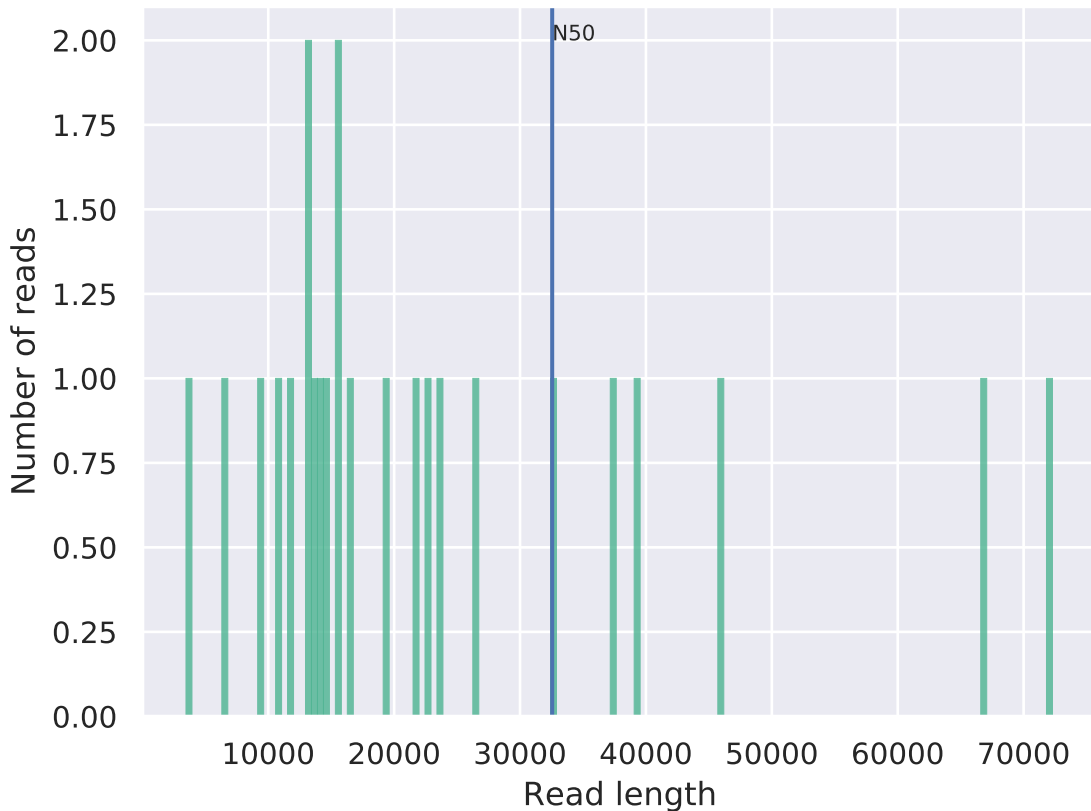

Supplement: Supplementary file 8 [file DataSheet_4.zip › SF1b/ccs999KIR7_18_3.contigs_MN167520_reports/ccs999KIR7_18_3.contigs_MN167520HistogramReadlength.pdf]

Yield by length

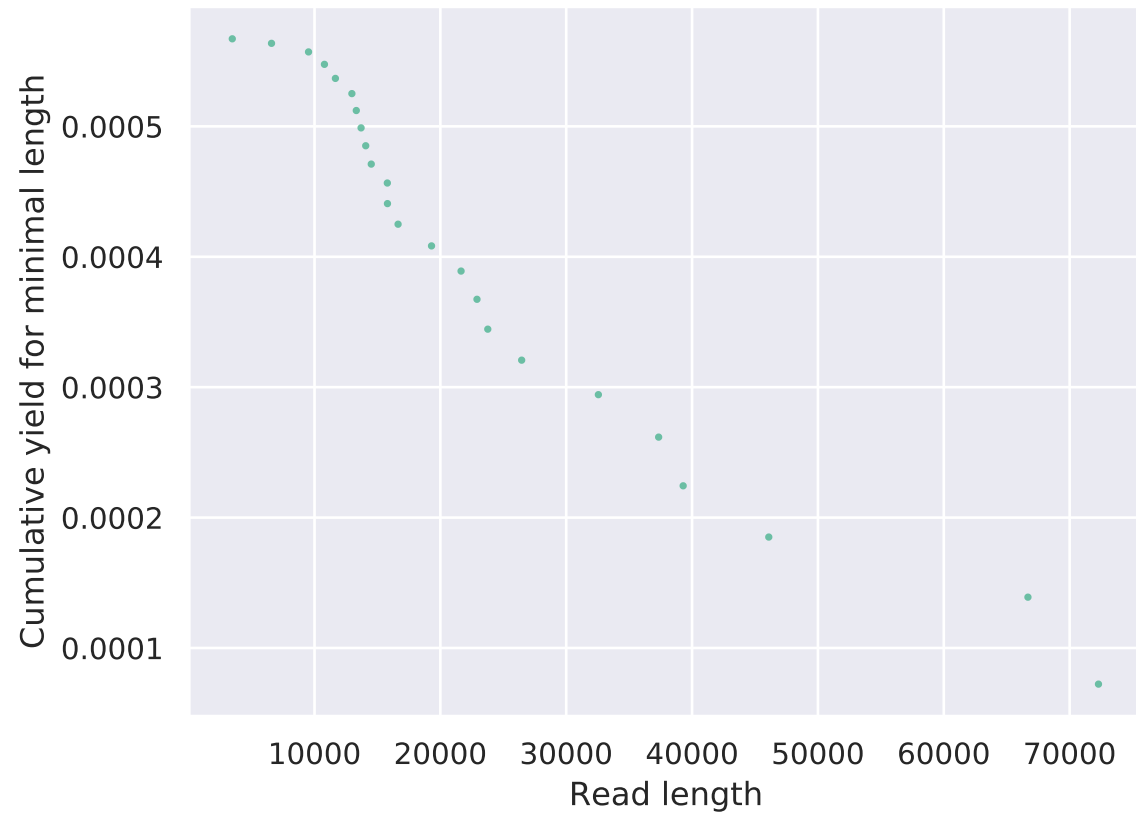

Supplement: Supplementary file 8 [file DataSheet_4.zip › SF1b/ccs999KIR7_18_3.contigs_MN167520_reports/ccs999KIR7_18_3.contigs_MN167520Yield_By_Length.pdf]

# Aligned read length vs Percent identity plot

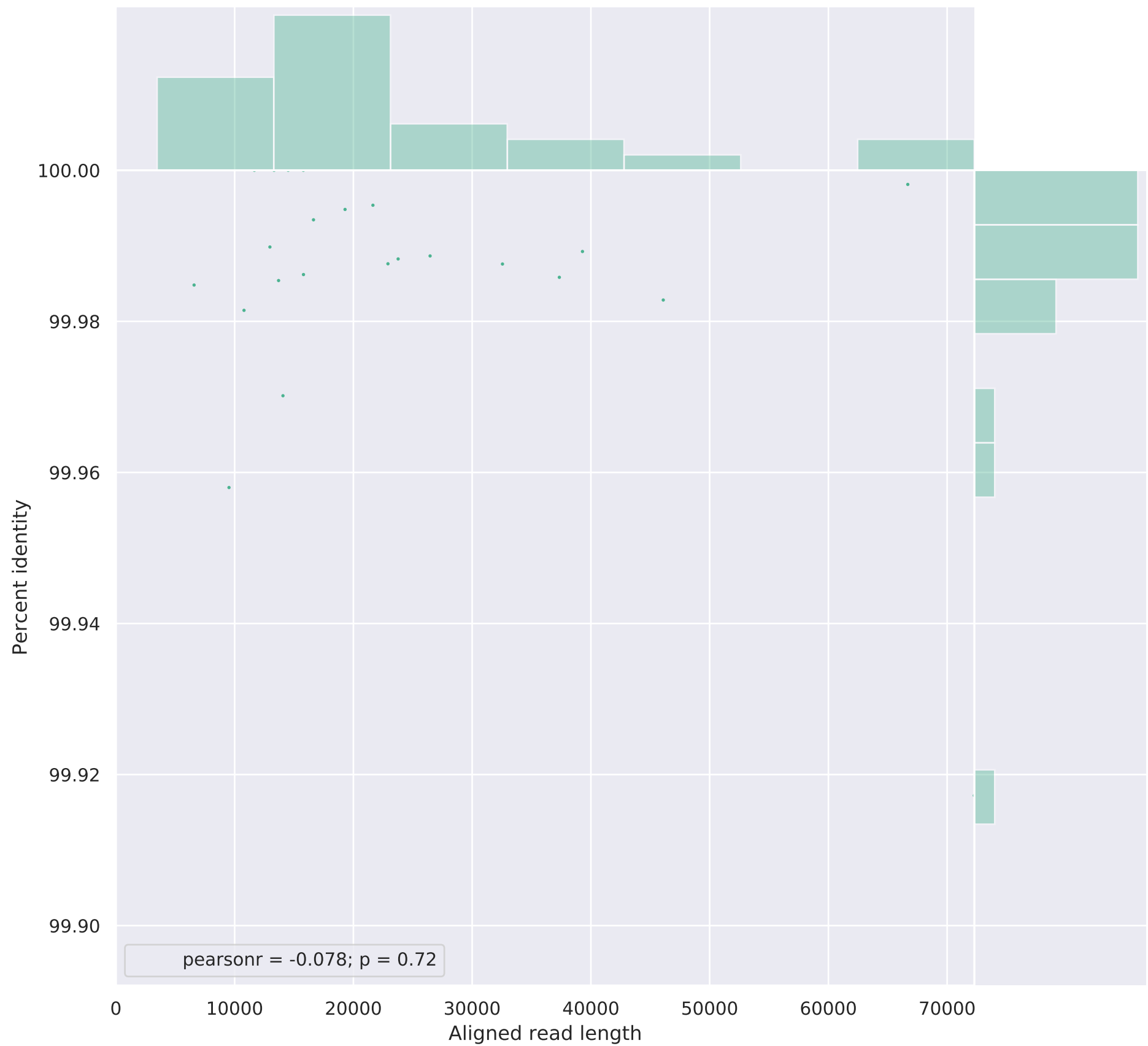

Supplement: Supplementary file 8 [file DataSheet_4.zip › SF1b/ccs999KIR7_18_3.contigs_MN167520_reports/ccs999KIR7_18_3.contigs_MN167520PercentIdentityvsAlignedReadLength_dot.pdf]

Yield by length

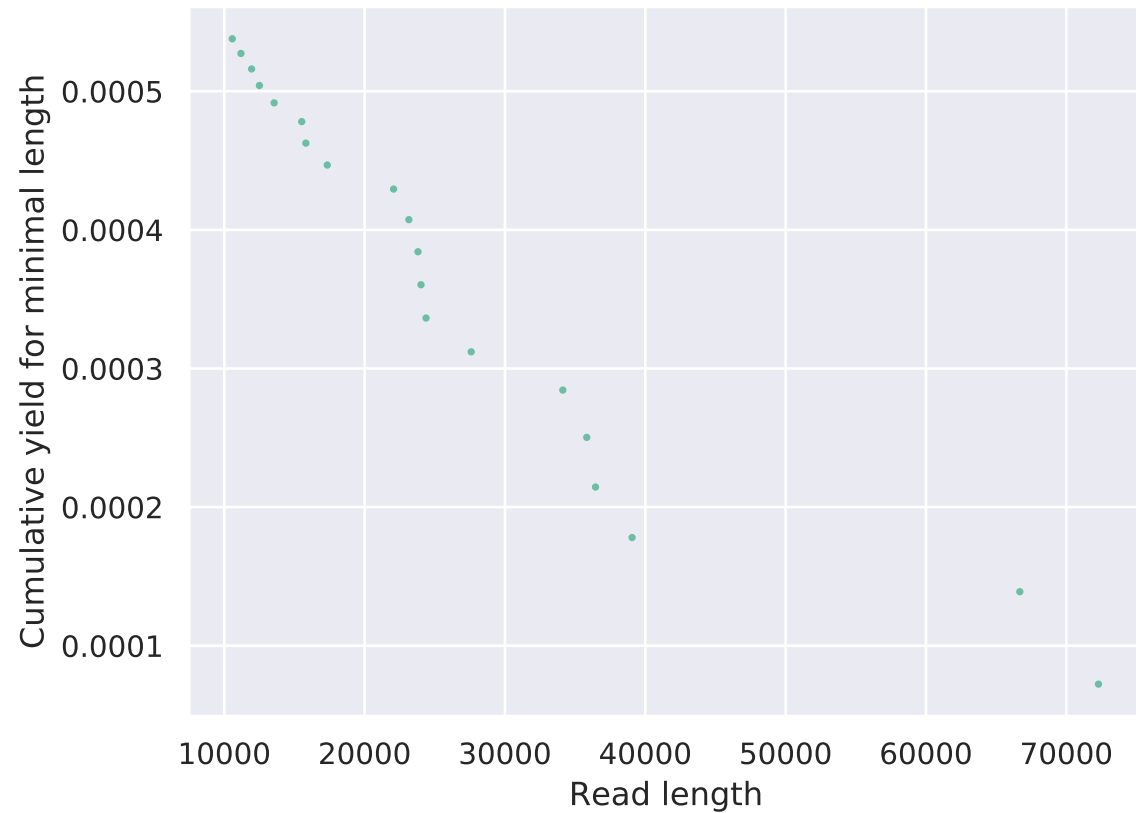

Supplement: Supplementary file 8 [file DataSheet_4.zip › SF1b/ccs999KIR7_18_3.contigs_MN167521_reports/ccs999KIR7_18_3.contigs_MN167521Yield_By_Length.pdf]

# Aligned read length vs Percent identity plot

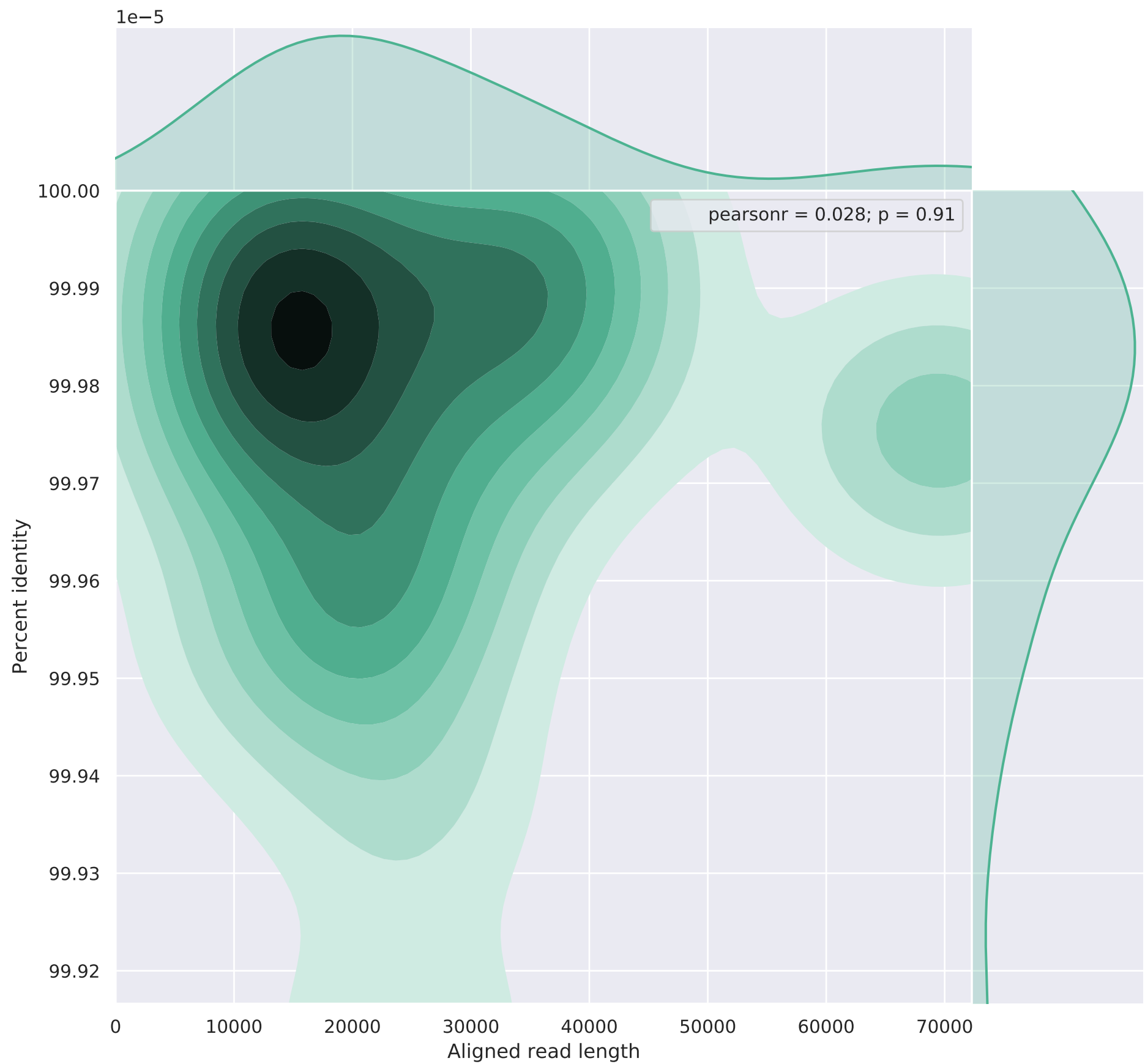

Supplement: Supplementary file 8 [file DataSheet_4.zip › SF1b/ccs999KIR7_18_3.contigs_MN167521_reports/ccs999KIR7_18_3.contigs_MN167521PercentIdentityvsAlignedReadLength_kde.pdf]

Weighted Histogram of read lengths after log transformation

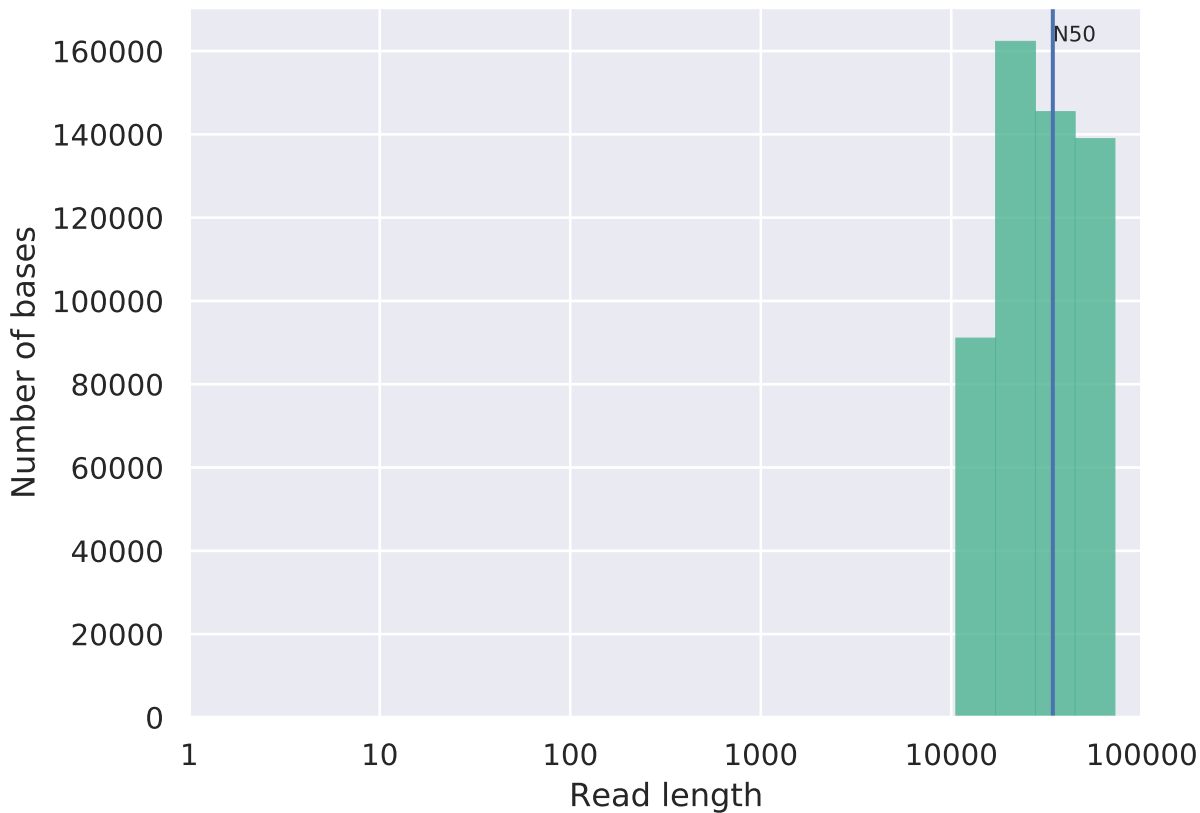

Supplement: Supplementary file 8 [file DataSheet_4.zip › SF1b/ccs999KIR7_18_3.contigs_MN167521_reports/ccs999KIR7_18_3.contigs_MN167521Weighted_LogTransformed_HistogramReadlength.pdf]

# Aligned read lengths vs Sequenced read length plot

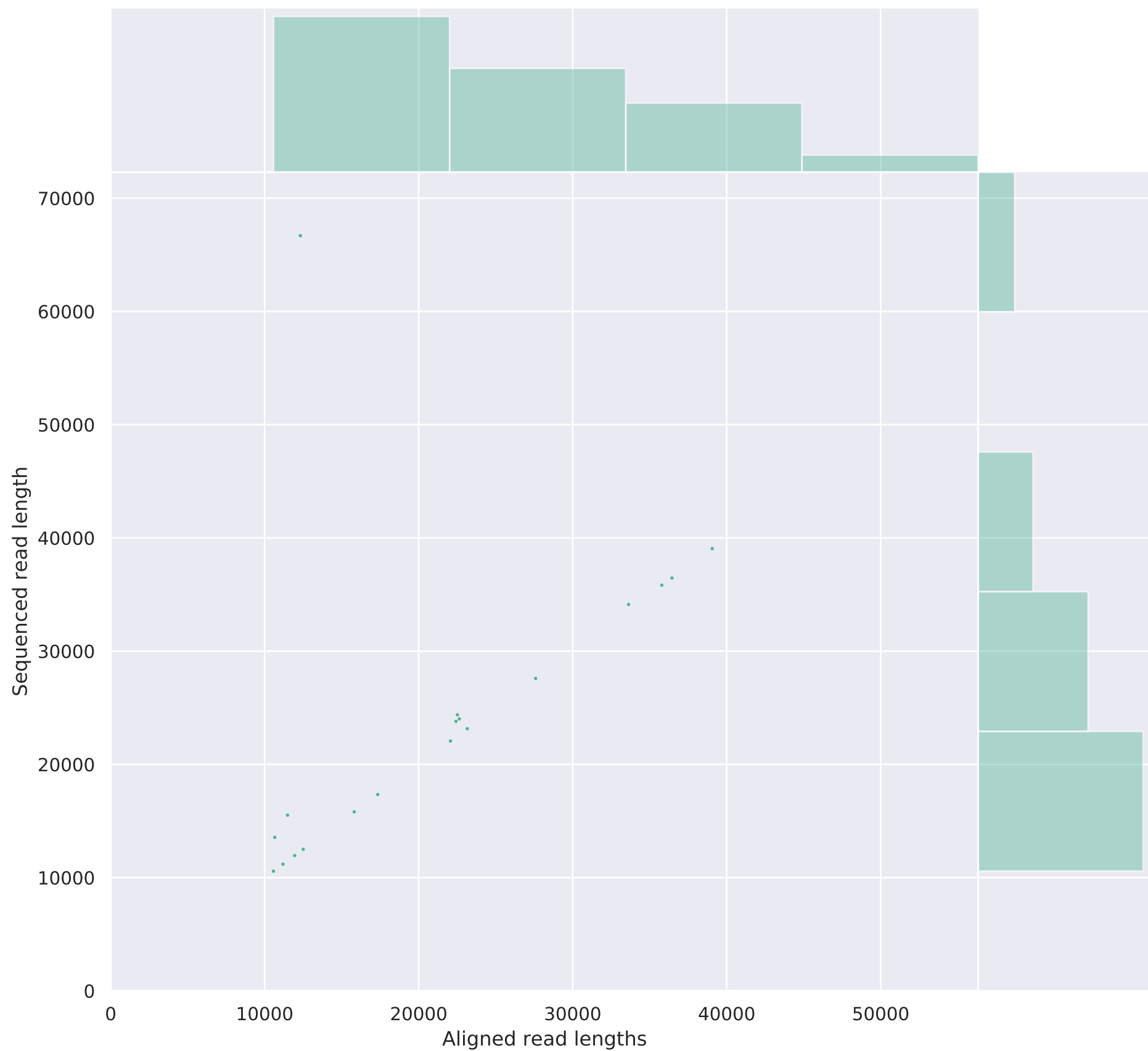

Supplement: Supplementary file 8 [file DataSheet_4.zip › SF1b/ccs999KIR7_18_3.contigs_MN167521_reports/ccs999KIR7_18_3.contigs_MN167521AlignedReadlengthvsSequencedReadLength_dot.pdf]

Histogram of read lengths after log transformation

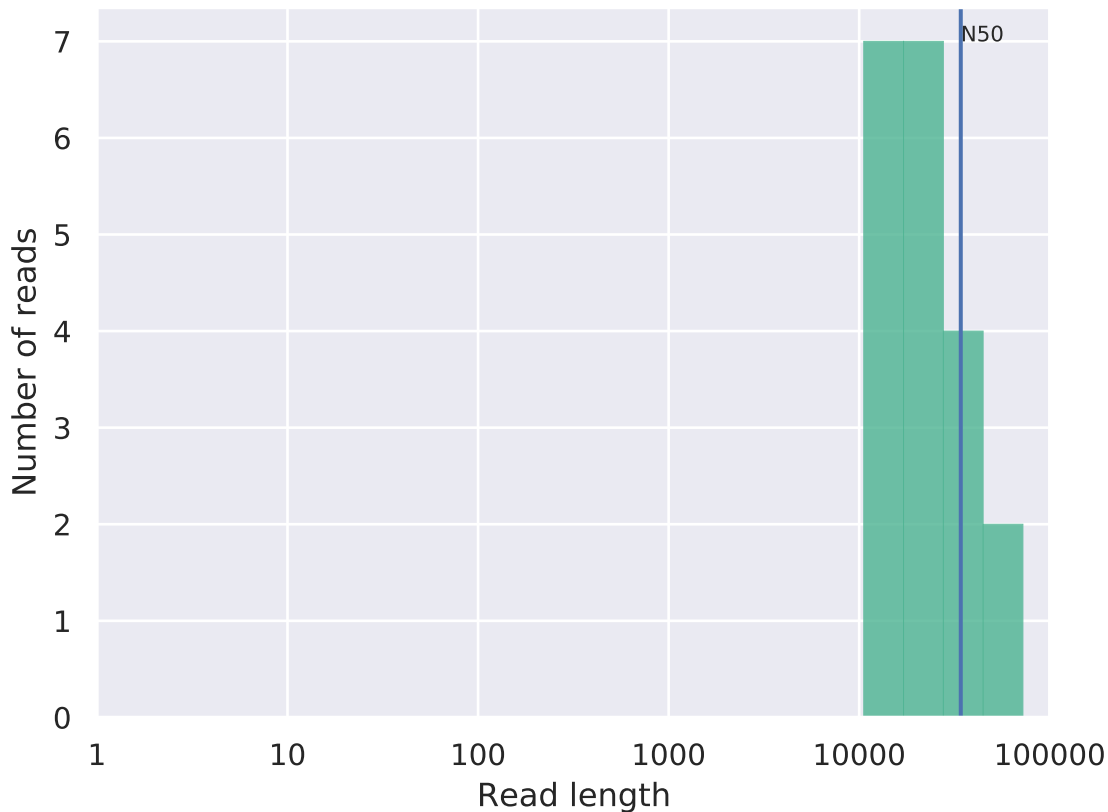

Supplement: Supplementary file 8 [file DataSheet_4.zip › SF1b/ccs999KIR7_18_3.contigs_MN167521_reports/ccs999KIR7_18_3.contigs_MN167521LogTransformed_HistogramReadlength.pdf]

Weighted Histogram of read lengths

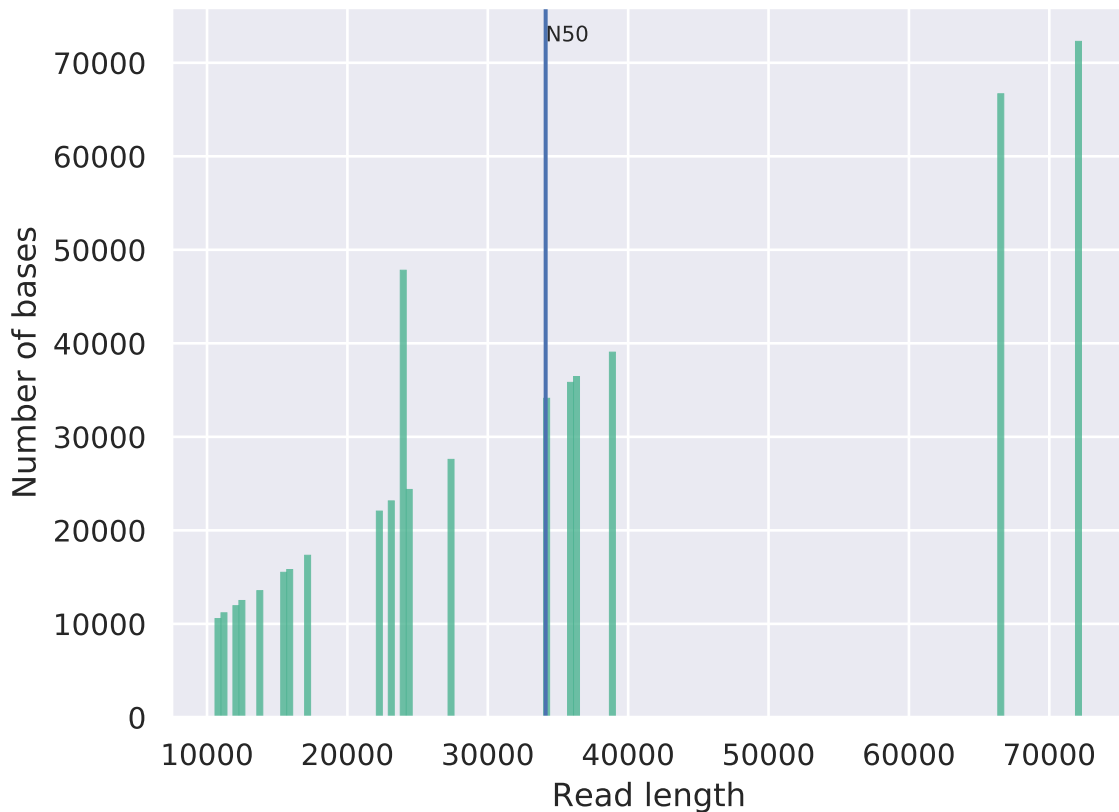

Supplement: Supplementary file 8 [file DataSheet_4.zip › SF1b/ccs999KIR7_18_3.contigs_MN167521_reports/ccs999KIR7_18_3.contigs_MN167521Weighted_HistogramReadlength.pdf]

Histogram of read lengths

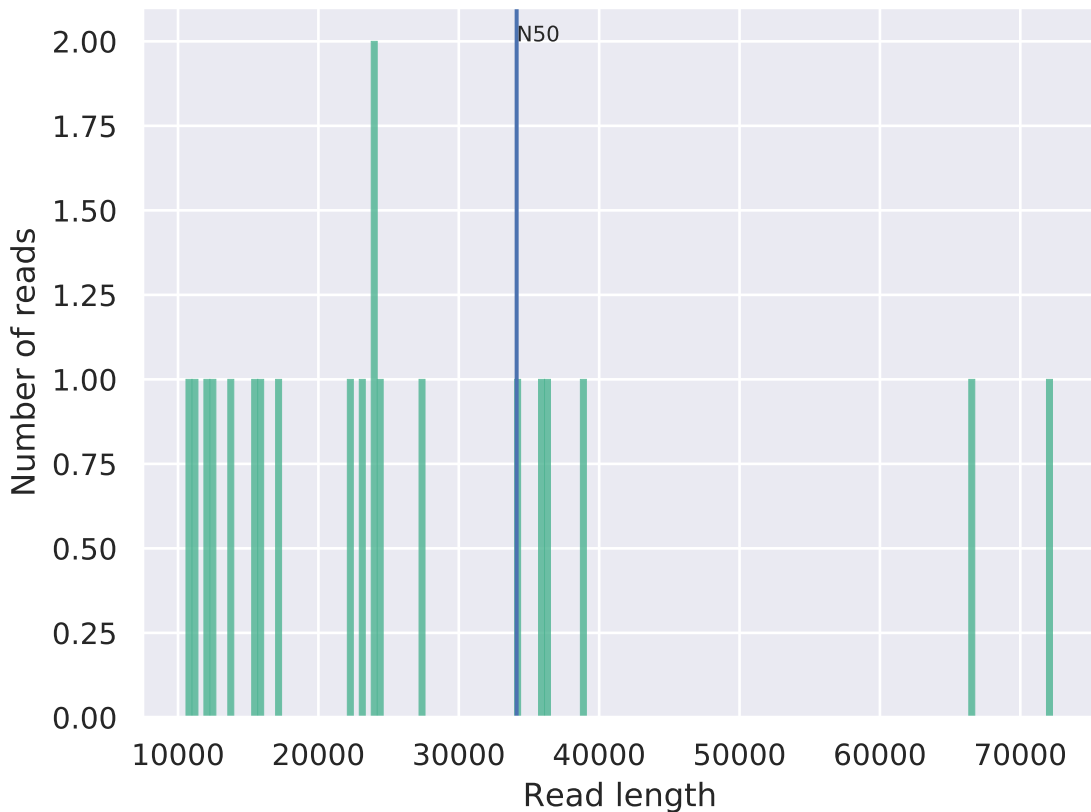

Supplement: Supplementary file 8 [file DataSheet_4.zip › SF1b/ccs999KIR7_18_3.contigs_MN167521_reports/ccs999KIR7_18_3.contigs_MN167521HistogramReadlength.pdf]

# Aligned read lengths vs Sequenced read length plot

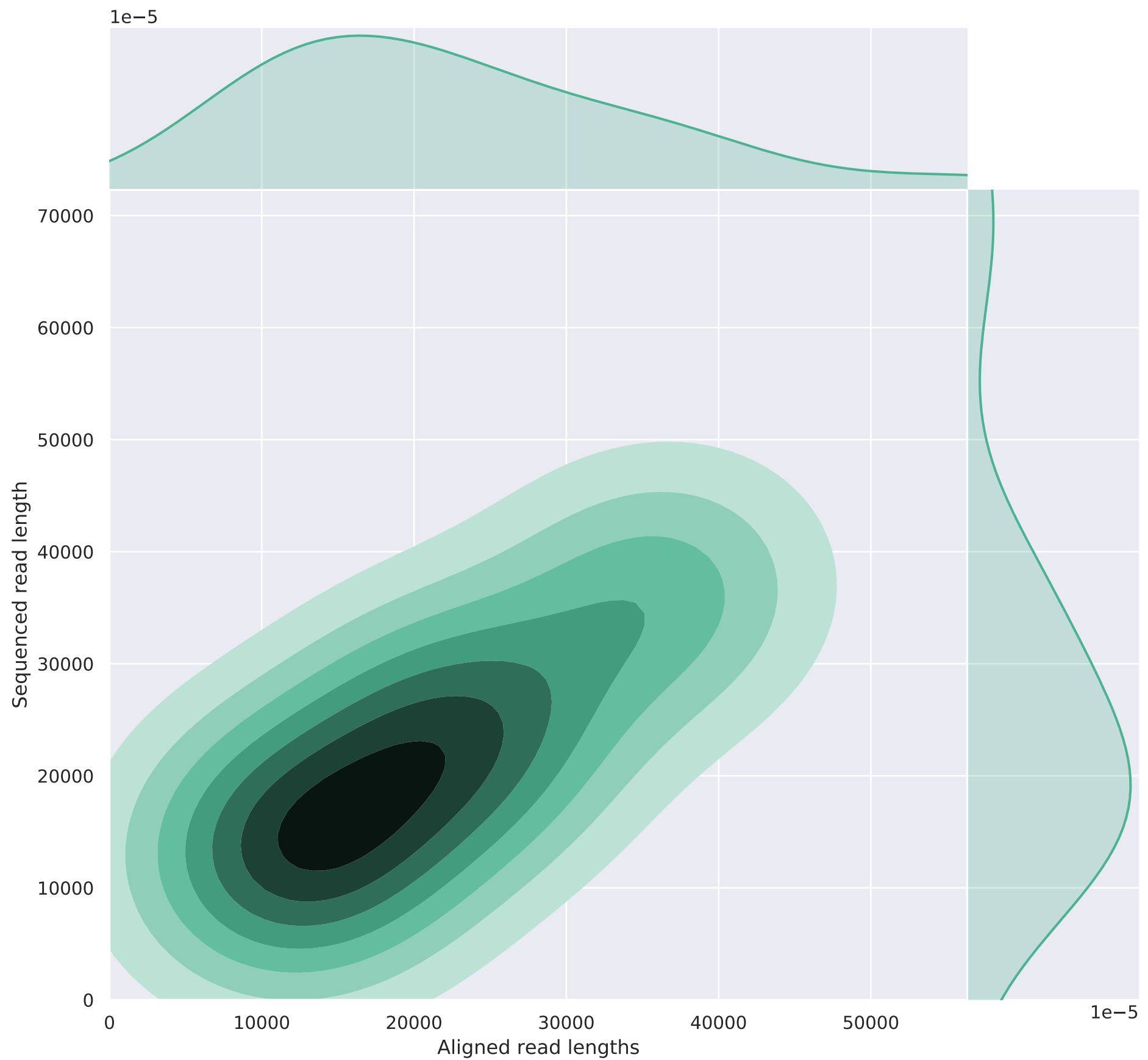

Supplement: Supplementary file 8 [file DataSheet_4.zip › SF1b/ccs999KIR7_18_3.contigs_MN167521_reports/ccs999KIR7_18_3.contigs_MN167521AlignedReadlengthvsSequencedReadLength_kde.pdf]

# Aligned read length vs Percent identity plot

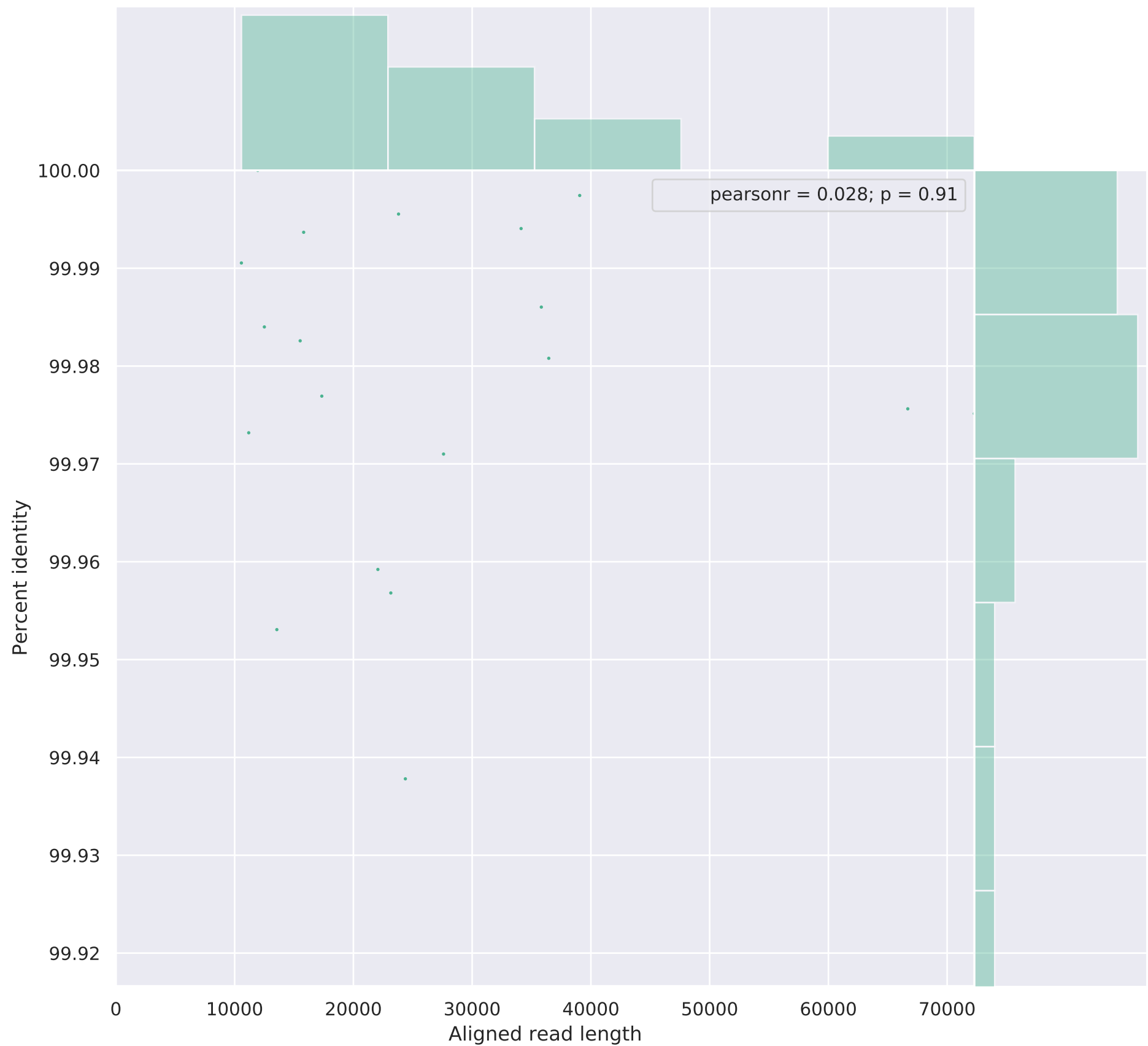

Supplement: Supplementary file 8 [file DataSheet_4.zip › SF1b/ccs999KIR7_18_3.contigs_MN167521_reports/ccs999KIR7_18_3.contigs_MN167521PercentIdentityvsAlignedReadLength_dot.pdf]

# Aligned read lengths vs Sequenced read length plot

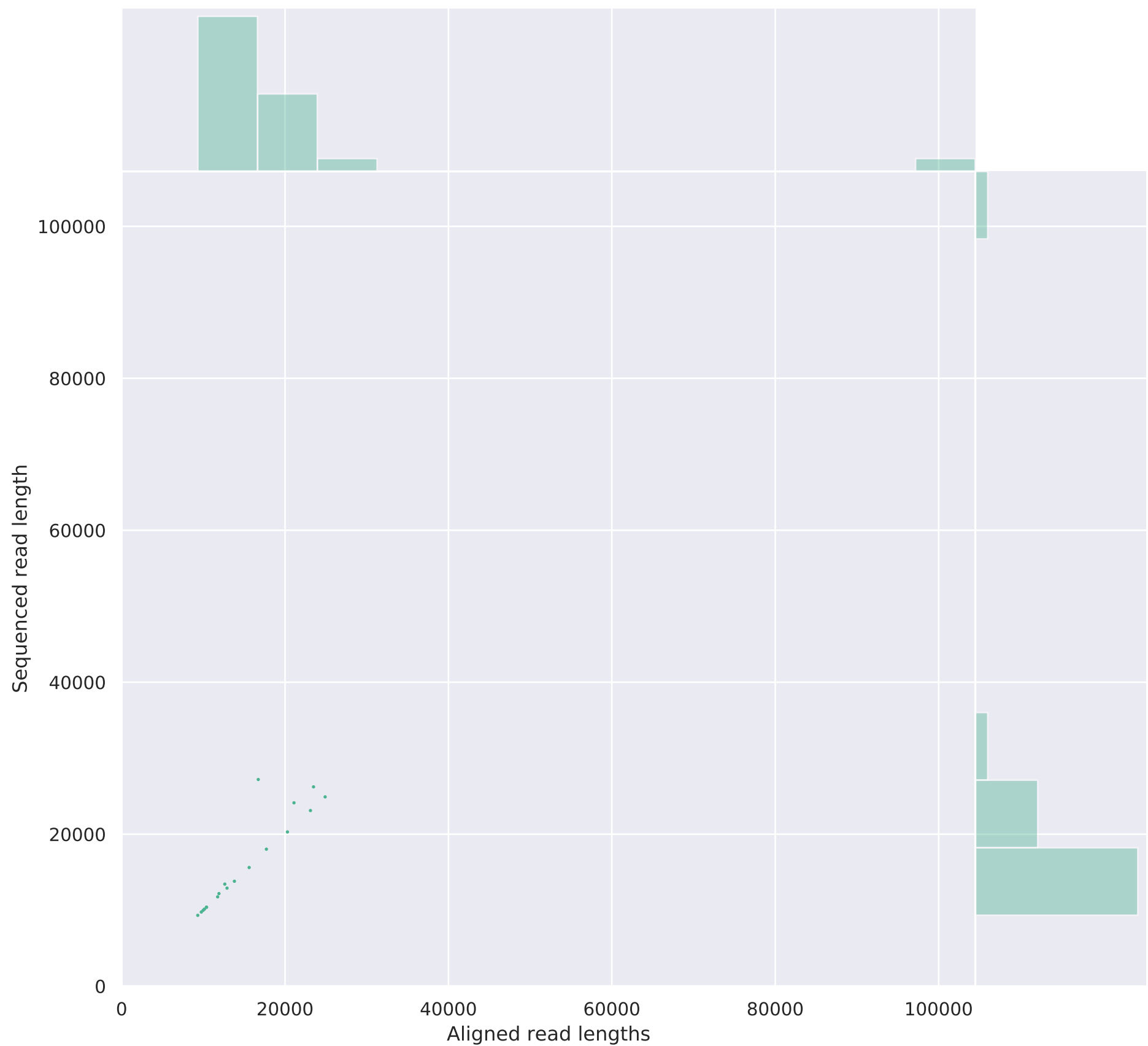

Supplement: Supplementary file 8 [file DataSheet_4.zip › SF1b/ccs999KIR7_18_4.contigs_MN167522_reports/ccs999KIR7_18_4.contigs_MN167522AlignedReadlengthvsSequencedReadLength_dot.pdf]

# Aligned read length vs Percent identity plot

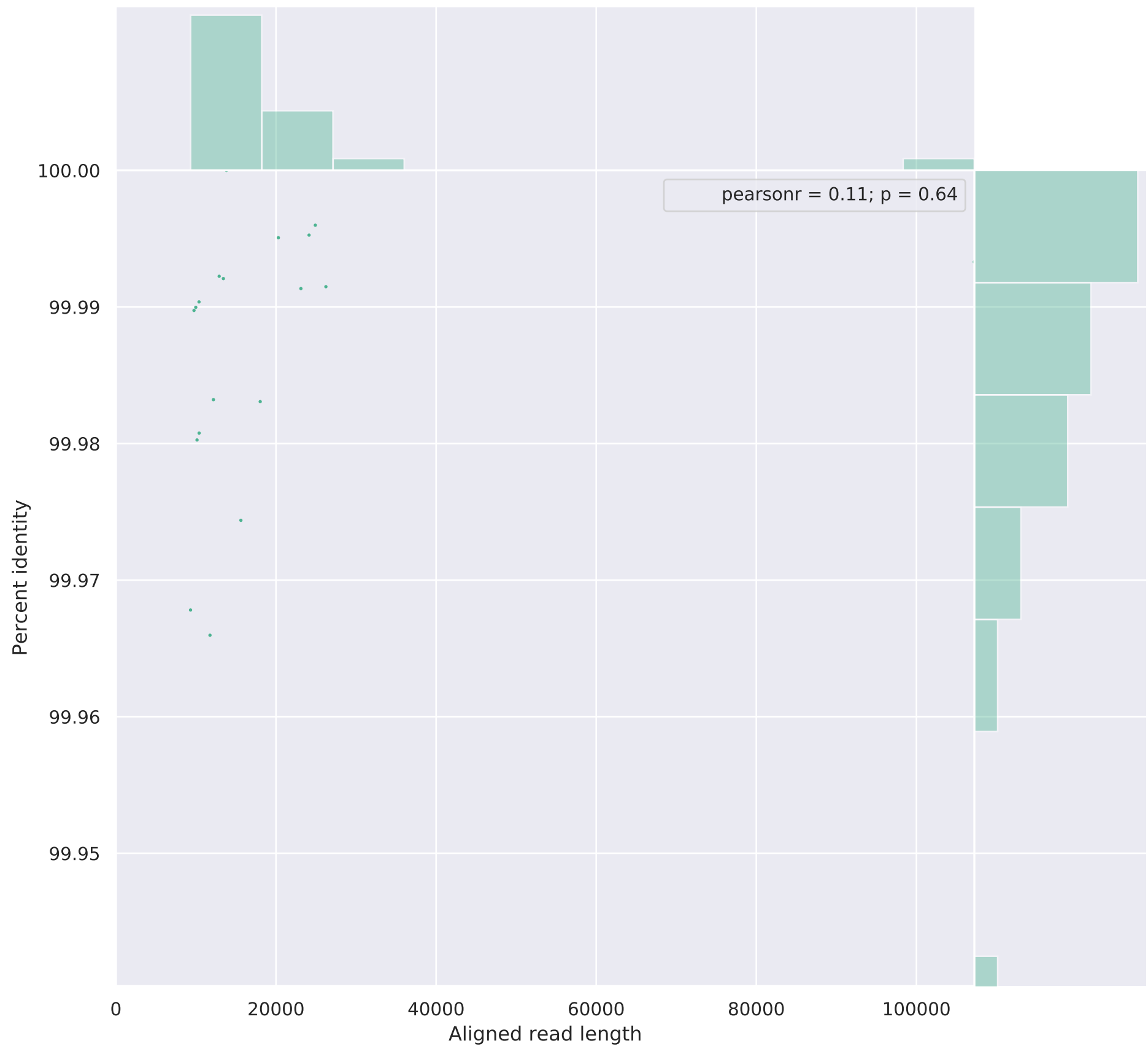

Supplement: Supplementary file 8 [file DataSheet_4.zip › SF1b/ccs999KIR7_18_4.contigs_MN167522_reports/ccs999KIR7_18_4.contigs_MN167522PercentIdentityvsAlignedReadLength_dot.pdf]

Weighted Histogram of read lengths after log transformation

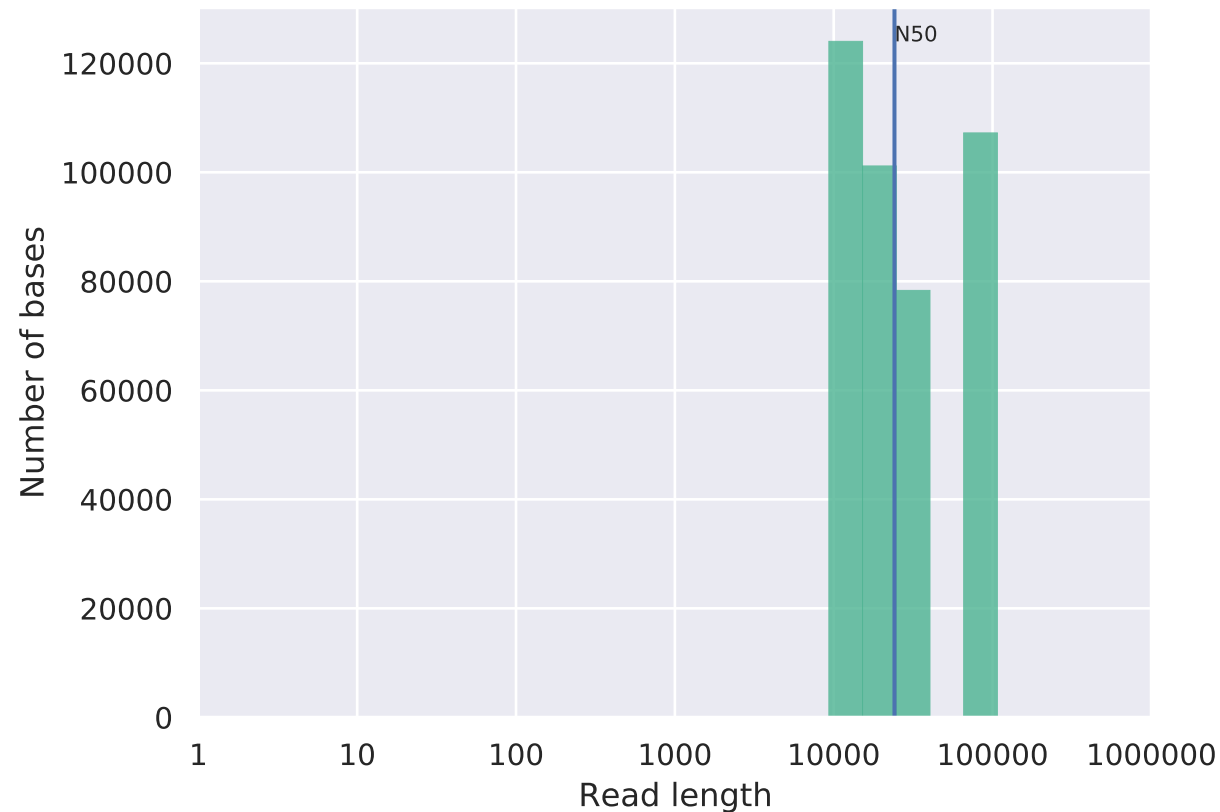

Supplement: Supplementary file 8 [file DataSheet_4.zip › SF1b/ccs999KIR7_18_4.contigs_MN167522_reports/ccs999KIR7_18_4.contigs_MN167522Weighted_LogTransformed_HistogramReadlength.pdf]

Weighted Histogram of read lengths

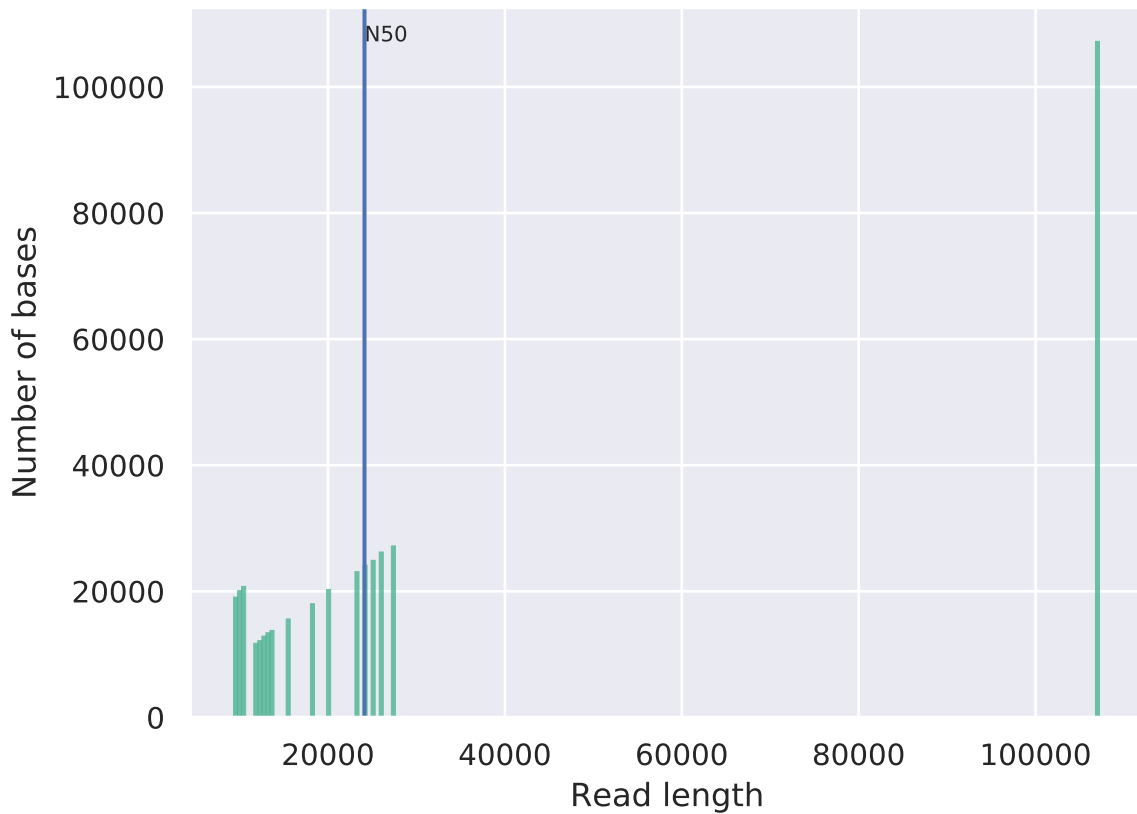

Supplement: Supplementary file 8 [file DataSheet_4.zip › SF1b/ccs999KIR7_18_4.contigs_MN167522_reports/ccs999KIR7_18_4.contigs_MN167522Weighted_HistogramReadlength.pdf]

# Aligned read length vs Percent identity plot

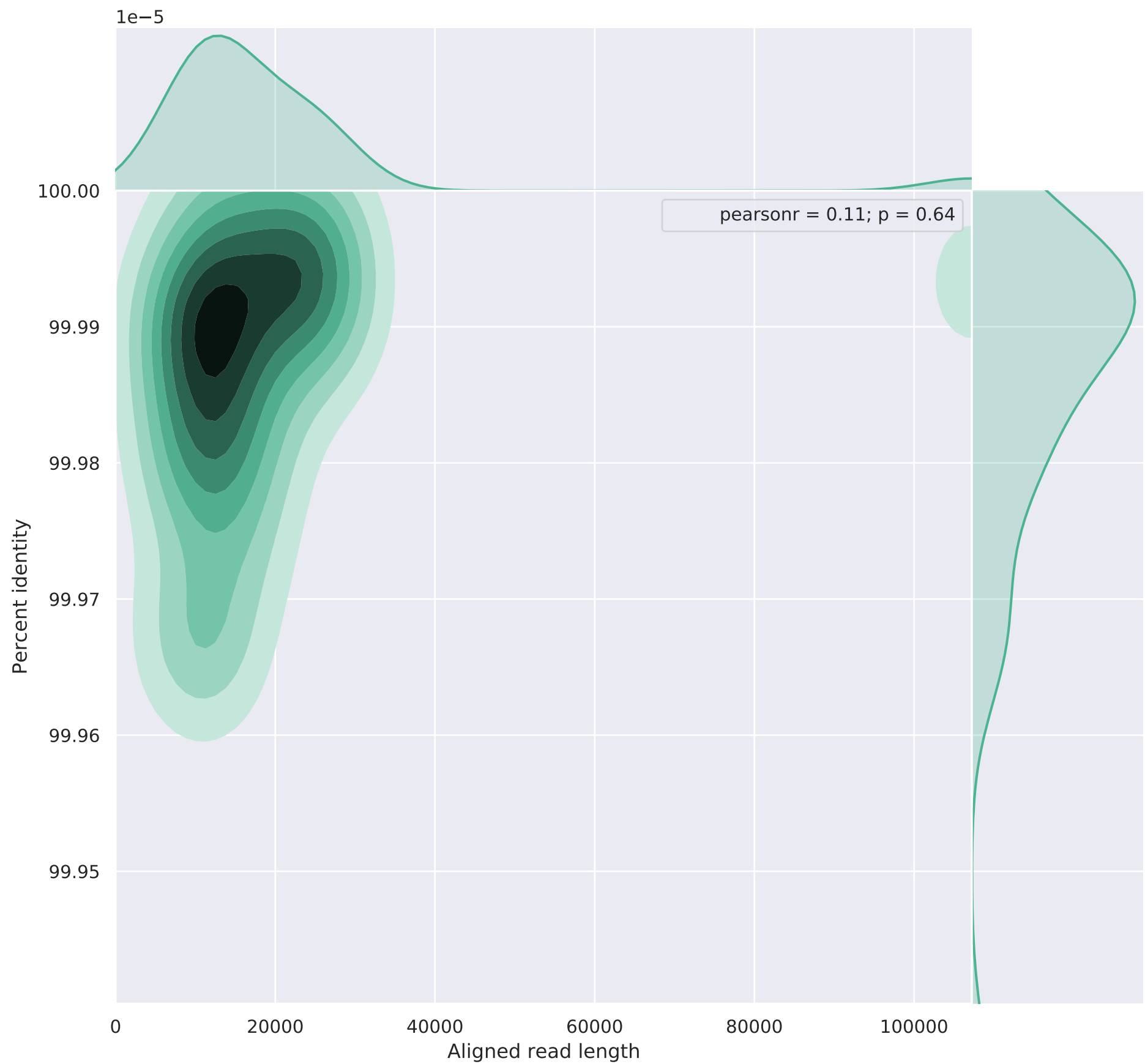

Supplement: Supplementary file 8 [file DataSheet_4.zip › SF1b/ccs999KIR7_18_4.contigs_MN167522_reports/ccs999KIR7_18_4.contigs_MN167522PercentIdentityvsAlignedReadLength_kde.pdf]

Histogram of read lengths

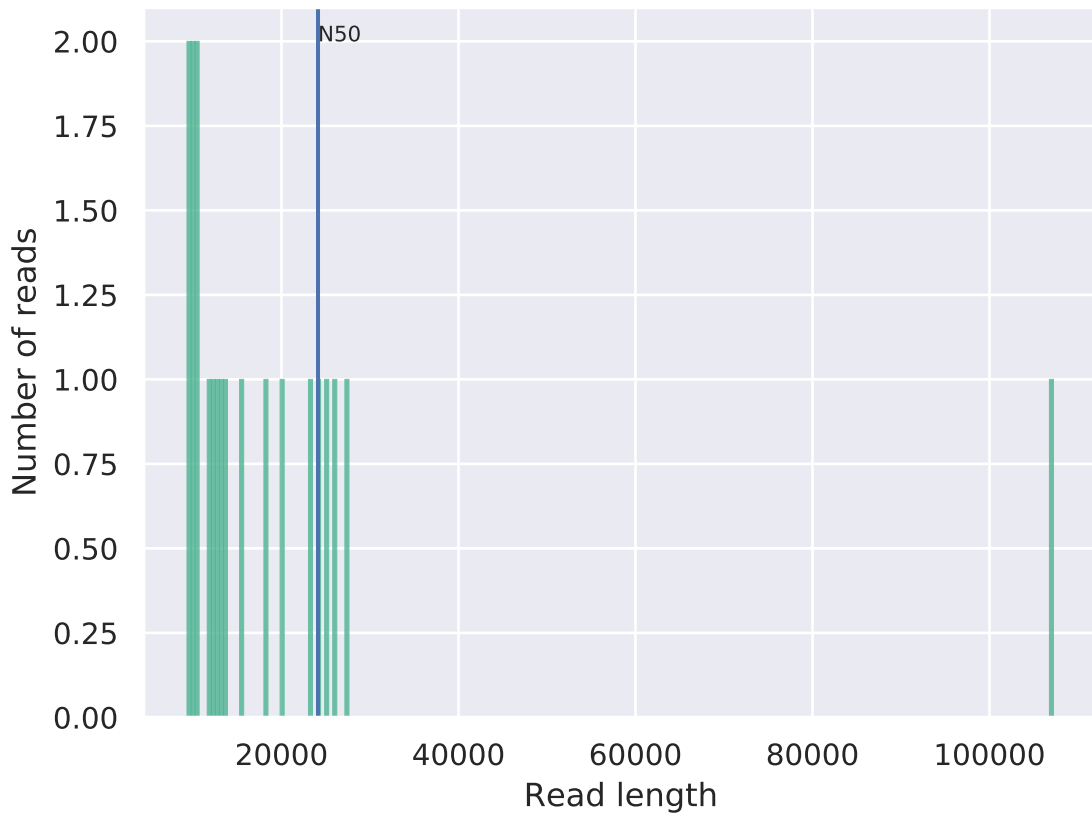

Supplement: Supplementary file 8 [file DataSheet_4.zip › SF1b/ccs999KIR7_18_4.contigs_MN167522_reports/ccs999KIR7_18_4.contigs_MN167522HistogramReadlength.pdf]

Histogram of read lengths after log transformation

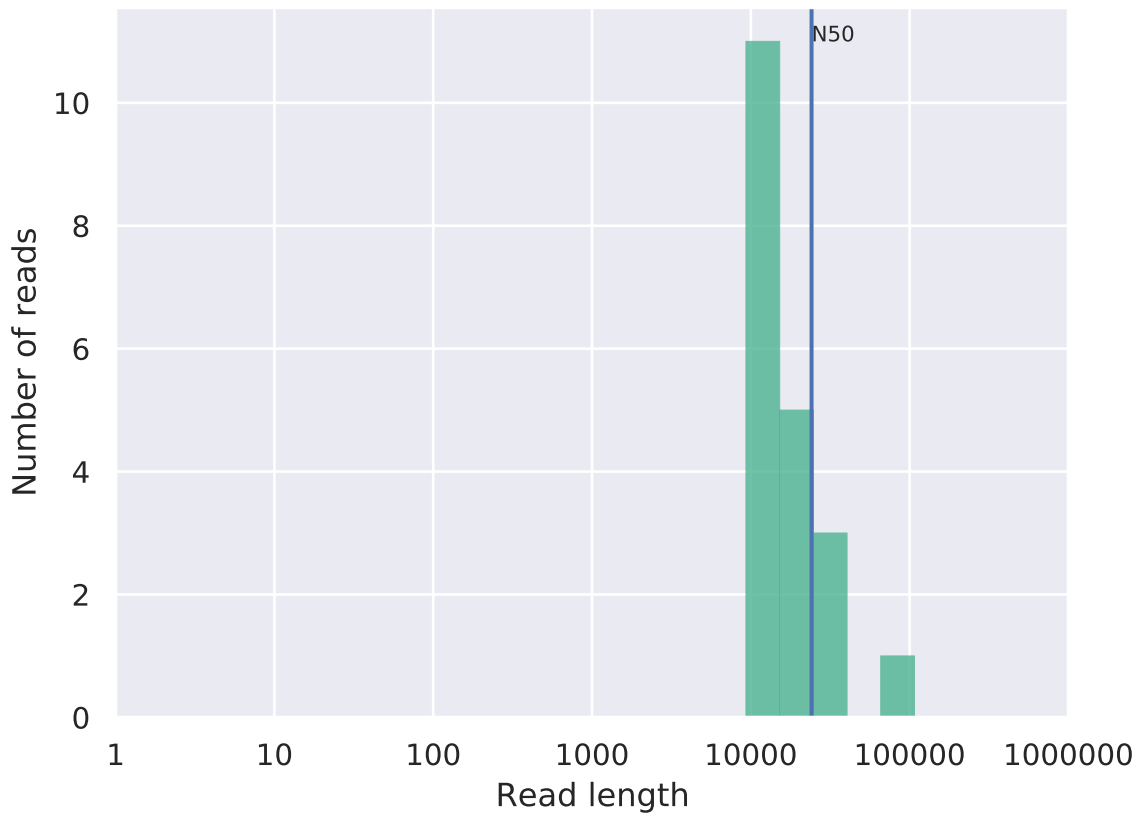

Supplement: Supplementary file 8 [file DataSheet_4.zip › SF1b/ccs999KIR7_18_4.contigs_MN167522_reports/ccs999KIR7_18_4.contigs_MN167522LogTransformed_HistogramReadlength.pdf]

# Aligned read lengths vs Sequenced read length plot

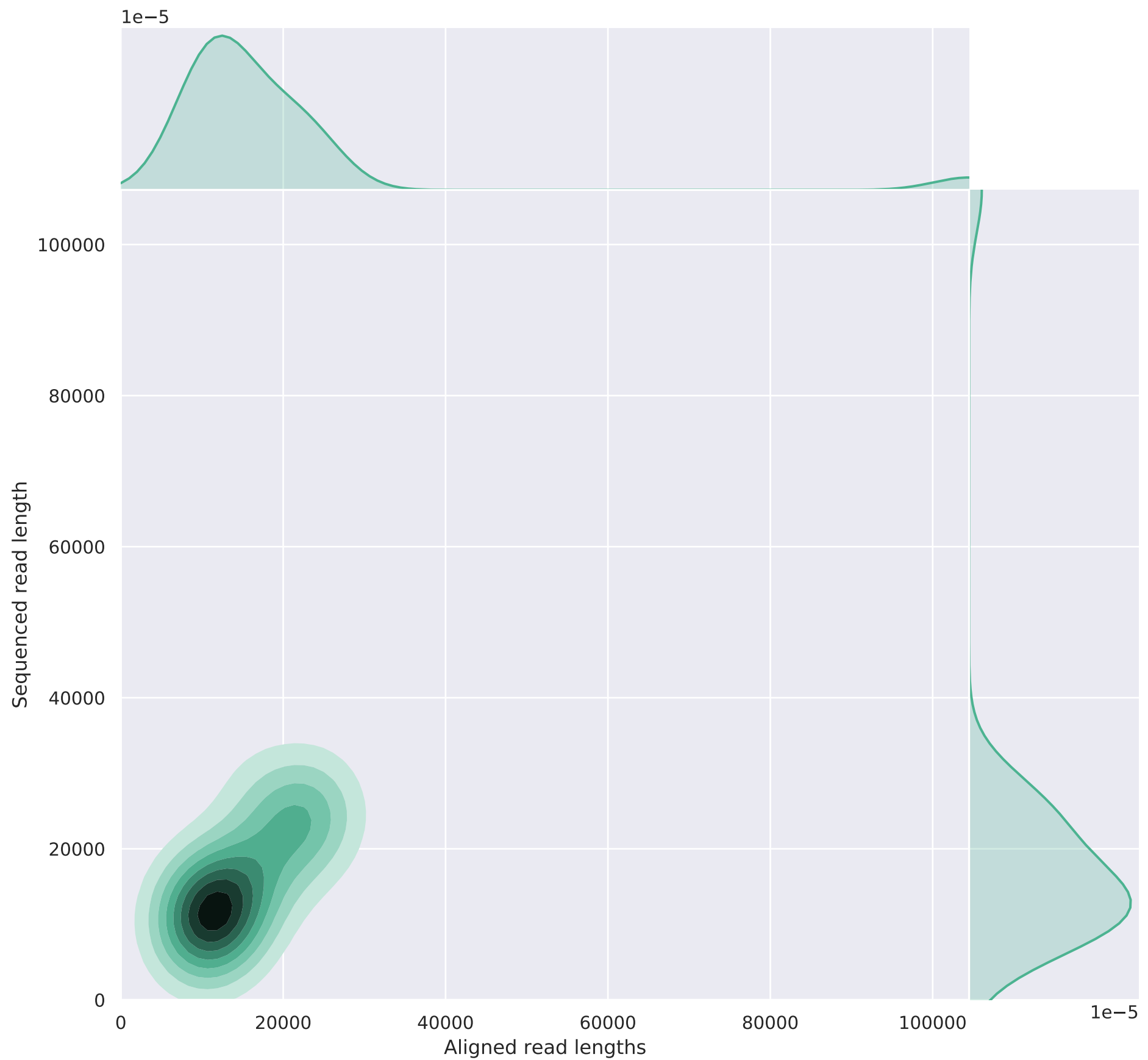

Supplement: Supplementary file 8 [file DataSheet_4.zip › SF1b/ccs999KIR7_18_4.contigs_MN167522_reports/ccs999KIR7_18_4.contigs_MN167522AlignedReadlengthvsSequencedReadLength_kde.pdf]

Yield by length

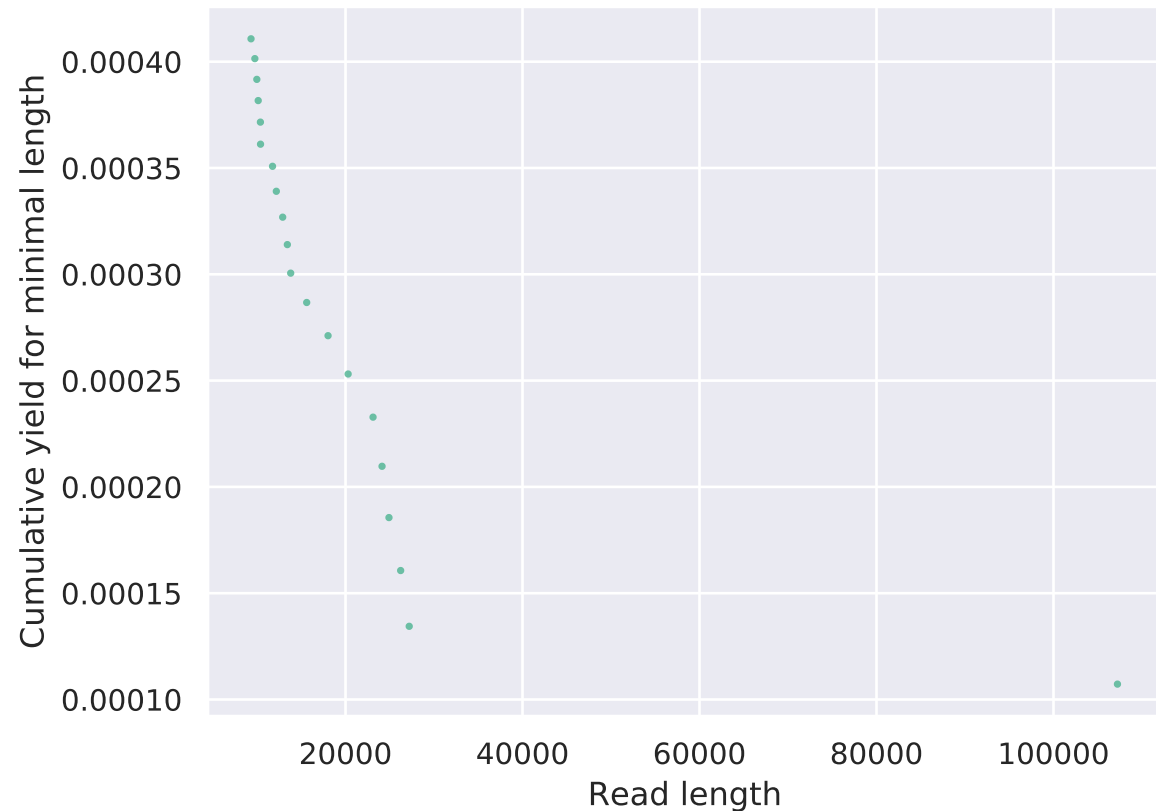

Supplement: Supplementary file 8 [file DataSheet_4.zip › SF1b/ccs999KIR7_18_4.contigs_MN167522_reports/ccs999KIR7_18_4.contigs_MN167522Yield_By_Length.pdf]
