# Supplementary material for: Efficient Sequencing, Assembly, and Annotation of Human KIR Haplotypes
Source: Front Immunol. 2020 Oct 9;11:582927. doi: 10.3389/fimmu.2020.582927 (PMC7581912; doi:10.3389/fimmu.2020.582927)

Qualimap Analysis Results

BAM QC analysis

Generated by Qualimap v.2.2.1

2020/04/21 17:48:32

1. Input data & parameters

1.1. QualiMap command line

qualimap bamqc -bam ccs999KIR7_18_6.contigs_MN167519_sorted.bam -nw 400 -hm 3

1.2. Alignment

Command line:	bwa mem -k1800 -W9000 -r10 -A1 -B100 -O40 -E40 -L50 -t7 MN167519.fasta ccs999KIR7_18_6.contigs.fasta.gz
Draw chromosome limits:	no
Analyze overlapping paired-end reads:	no
Program:	bwa (0.7.17-r1188)
Analysis date:	Tue Apr 21 17:48:31 GMT 2020
Size of a homopolymer:	3
Skip duplicate alignments:	no
Number of windows:	400
BAM file:	ccs999KIR7_18_6.contigs_MN167519_sorted.bam

2. Summary

2.1. Globals

Reference size	236,704
Number of reads	21
Mapped reads	21 / 100%
Unmapped reads	0 / 0%
Mapped paired reads	0 / 0%
Read min/max/mean length	9,378 / 105,322 / 25,226.57
Duplicated reads (estimated)	1 / 4.76%
Duplication rate	5%
Clipped reads	7 / 33.33%

2.2. ACGT Content

Number/percentage of A's	137,980 / 26.92%
Number/percentage of C's	115,744 / 22.58%
Number/percentage of T's	131,987 / 25.75%
Number/percentage of G's	126,880 / 24.75%
Number/percentage of N's	0 / 0%
GC Percentage	47.33%

2.3. Coverage

Mean	2.1657
Standard Deviation	1.4135

2.4. Mapping Quality

Mean Mapping Quality	52.94

2.5. Mismatches and indels

General error rate	0.01%
Mismatches	64
Insertions	4
Mapped reads with at least one insertion	19.05%
Deletions	30
Mapped reads with at least one deletion	71.43%
Homopolymer indels	97.06%

2.6. Chromosome stats

Name	Length	Mapped bases	Mean coverage	Standard deviation
MN167519	236704	512626	2.1657	1.4135

3. Results : Coverage across reference

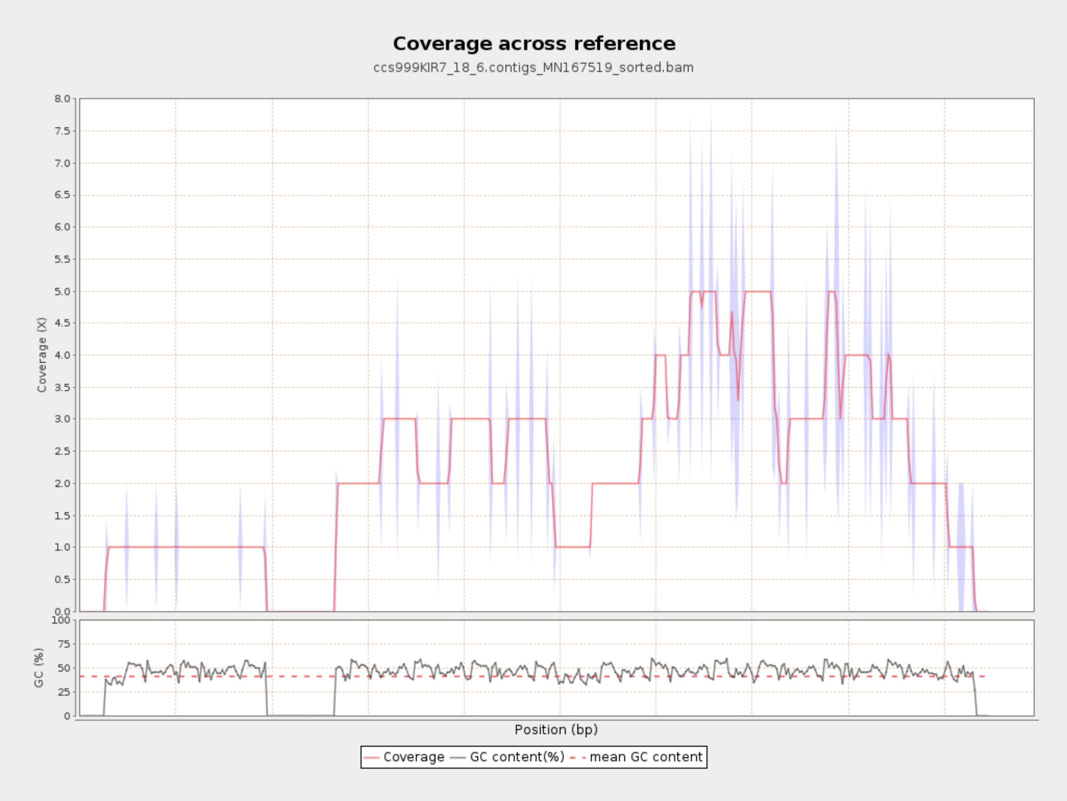

4. Results : Coverage Histogram

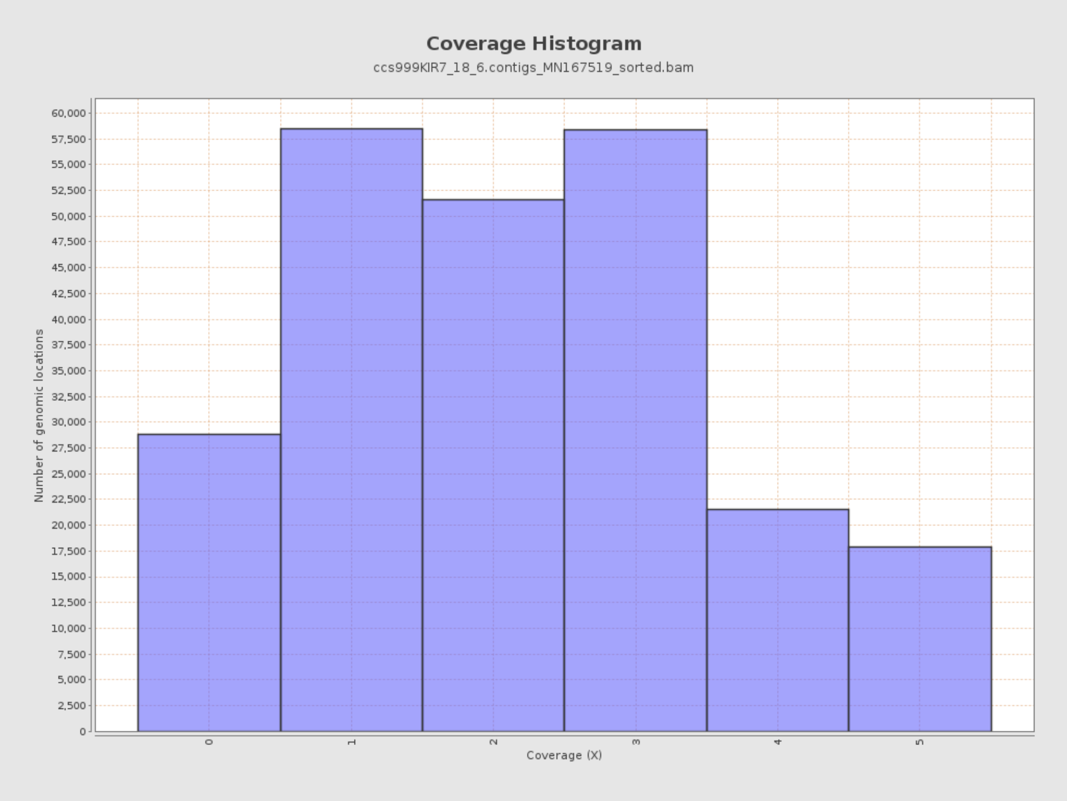

5. Results : Coverage Histogram (0-50X)

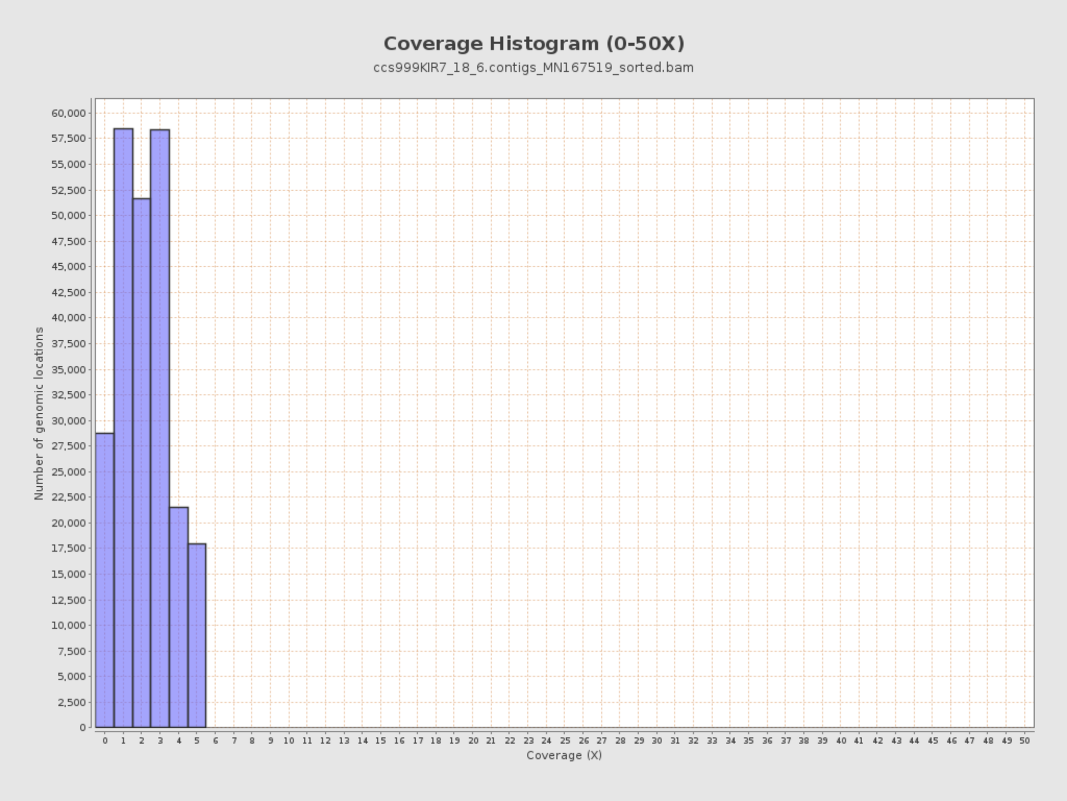

6. Results : Genome Fraction Coverage

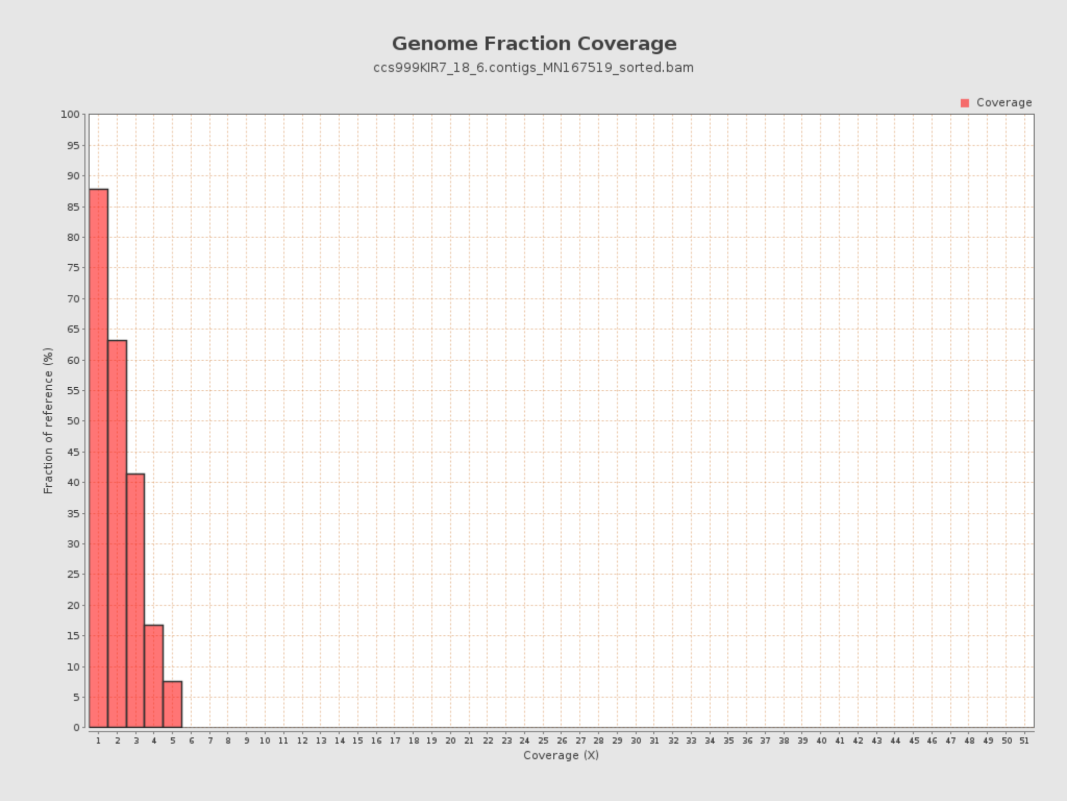

7. Results : Duplication Rate Histogram

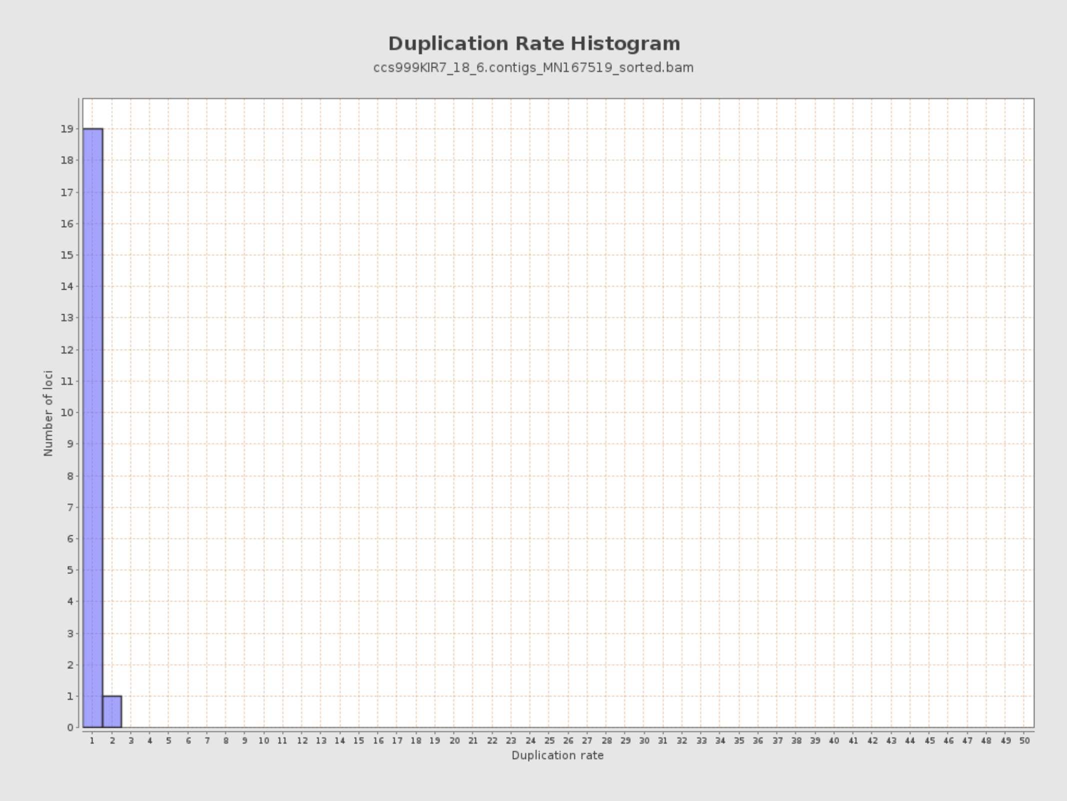

8. Results : Mapped Reads Nucleotide Content

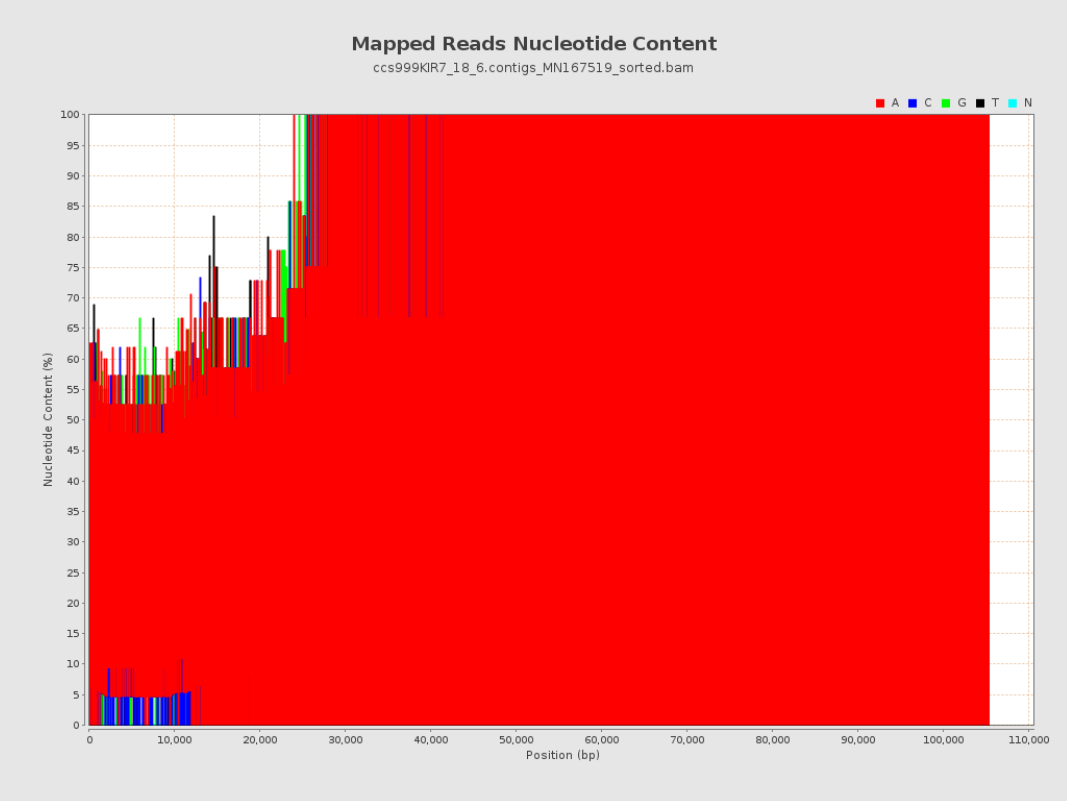

9. Results : Mapped Reads GC-content Distribution

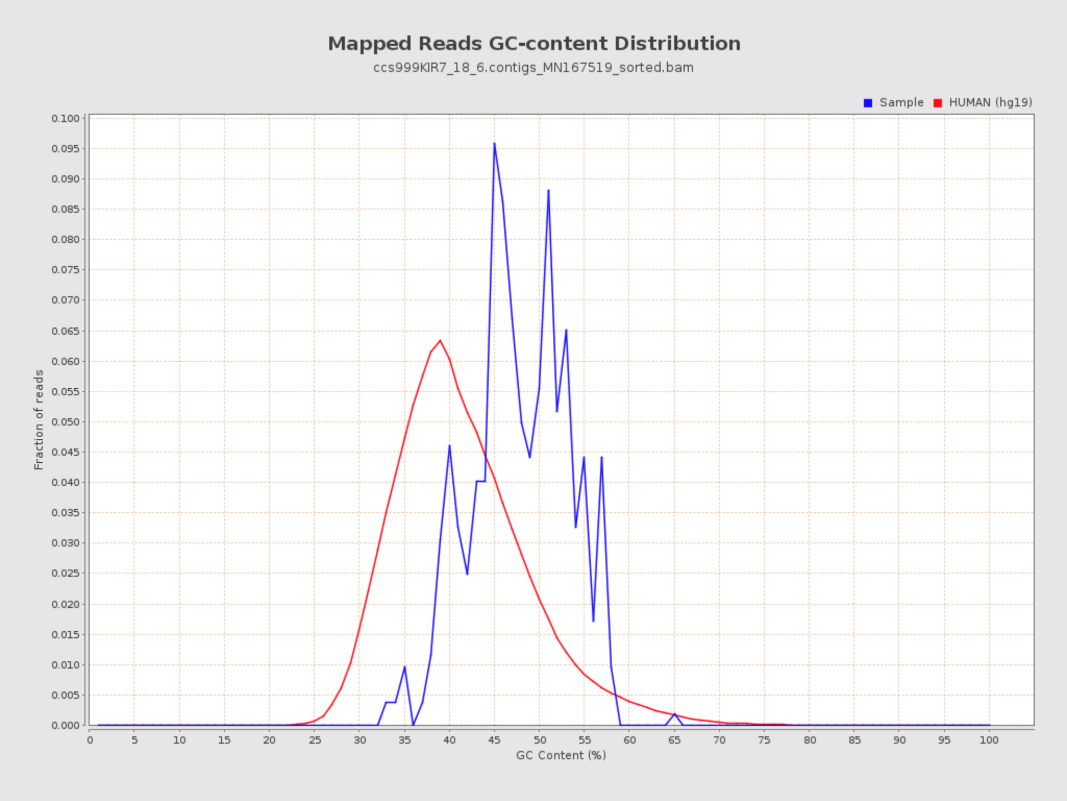

10. Results : Mapped Reads Clipping Profile

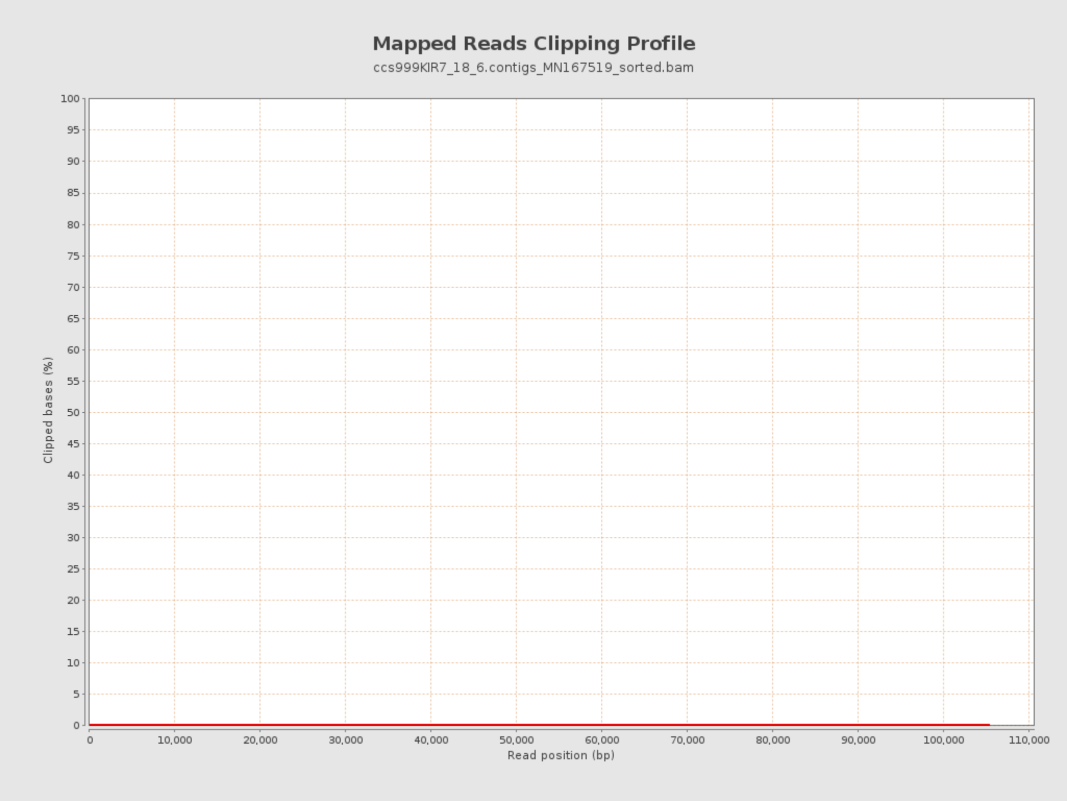

11. Results : Homopolymer Indels

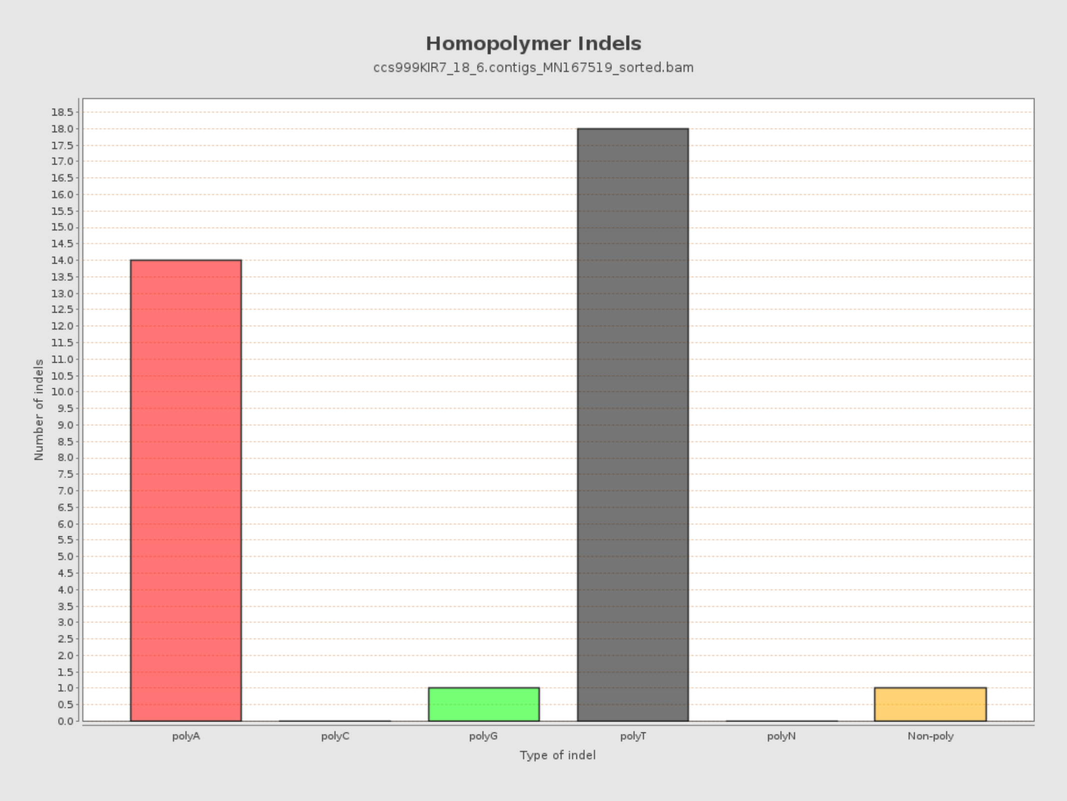

12. Results : Mapping Quality Across Reference

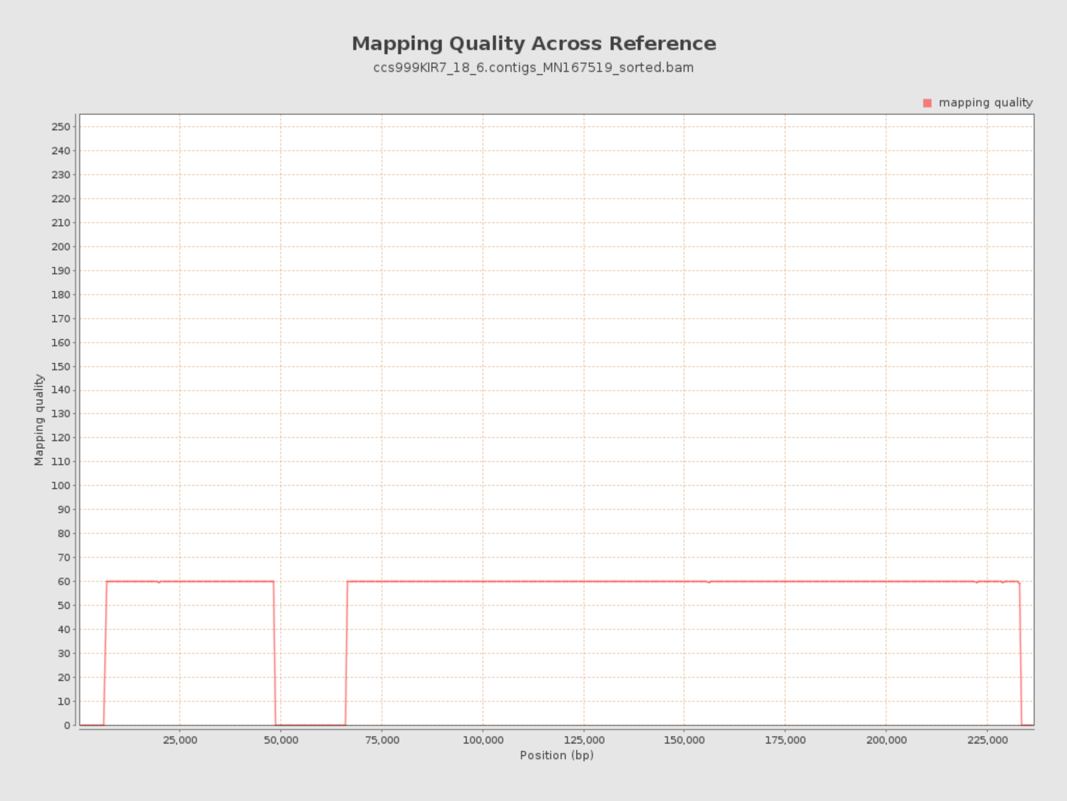

13. Results : Mapping Quality Histogram

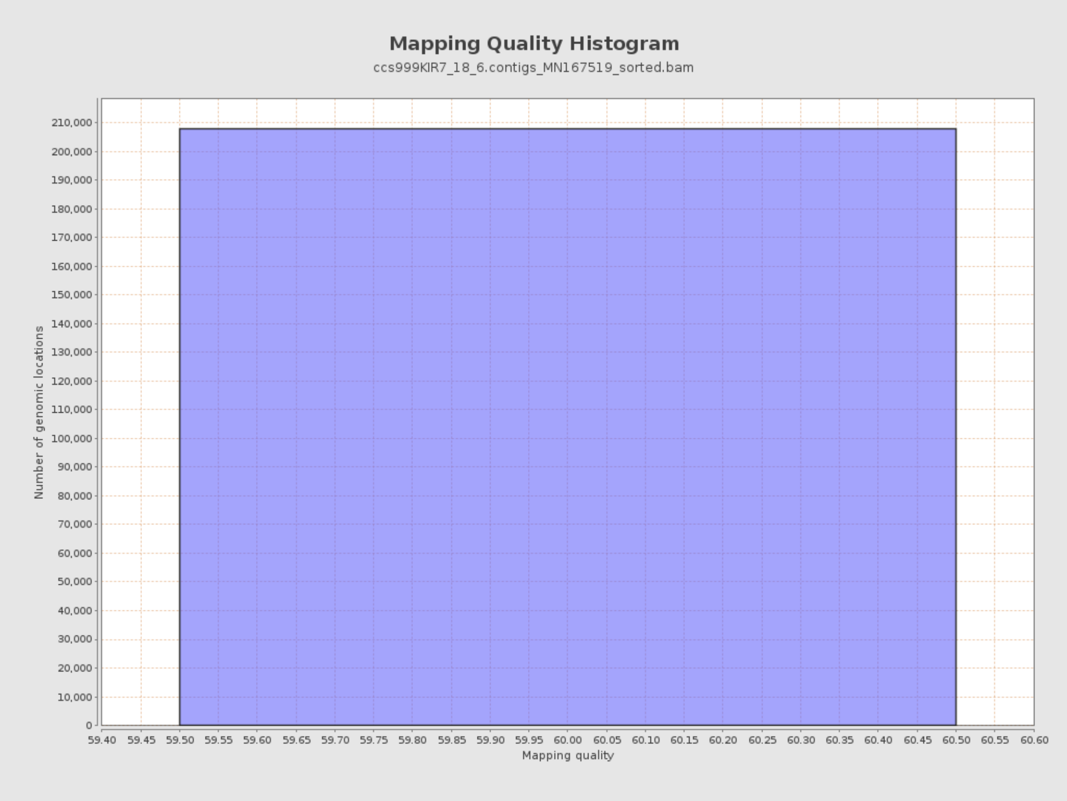

Supplement: Supplementary file 7 [file DataSheet_3.zip › SF1c/ccs999KIR7_18_6.contigs_MN167519_reports/ccs999KIR7_18_6.contigs_MN167519_qualimap.pdf]
