# Supplementary material for: Efficient Sequencing, Assembly, and Annotation of Human KIR Haplotypes
Source: Front Immunol. 2020 Oct 9;11:582927. doi: 10.3389/fimmu.2020.582927 (PMC7581912; doi:10.3389/fimmu.2020.582927)

Qualimap Analysis Results

BAM QC analysis

Generated by Qualimap v.2.2.1

2020/04/21 17:47:33

1. Input data & parameters

1.1. QualiMap command line

qualimap bamqc -bam ccs999KIR7_18_5.contigs_MN167529_sorted.bam -nw 400 -hm 3

1.2. Alignment

Command line:	bwa mem -k1800 -W9000 -r10 -A1 -B100 -O40 -E40 -L50 -t7 MN167529.fasta ccs999KIR7_18_5.contigs.fasta.gz
Draw chromosome limits:	no
Analyze overlapping paired-end reads:	no
Program:	bwa (0.7.17-r1188)
Analysis date:	Tue Apr 21 17:47:32 GMT 2020
Size of a homopolymer:	3
Skip duplicate alignments:	no
Number of windows:	400
BAM file:	ccs999KIR7_18_5.contigs_MN167529_sorted.bam

2. Summary

2.1. Globals

Reference size	201,909
Number of reads	3
Mapped reads	3 / 100%
Unmapped reads	0 / 0%
Mapped paired reads	0 / 0%
Read min/max/mean length	21,417 / 157,925 / 67,038.67
Duplicated reads (estimated)	0 / 0%
Duplication rate	0%
Clipped reads	2 / 66.67%

2.2. ACGT Content

Number/percentage of A's	18,727 / 27.83%
Number/percentage of C's	15,054 / 22.37%
Number/percentage of T's	17,094 / 25.4%
Number/percentage of G's	16,422 / 24.4%
Number/percentage of N's	0 / 0%
GC Percentage	46.77%

2.3. Coverage

Mean	0.3334
Standard Deviation	0.7177

2.4. Mapping Quality

Mean Mapping Quality	12.6

2.5. Mismatches and indels

General error rate	0.05%
Mismatches	23
Insertions	5
Mapped reads with at least one insertion	100%
Deletions	8
Mapped reads with at least one deletion	100%
Homopolymer indels	69.23%

2.6. Chromosome stats

**Name**	**Length**	**Mapped bases**	**Mean coverage**	**Standard deviation**
MN167529	201909	67308	0.3334	0.7177

3. Results : Coverage across reference

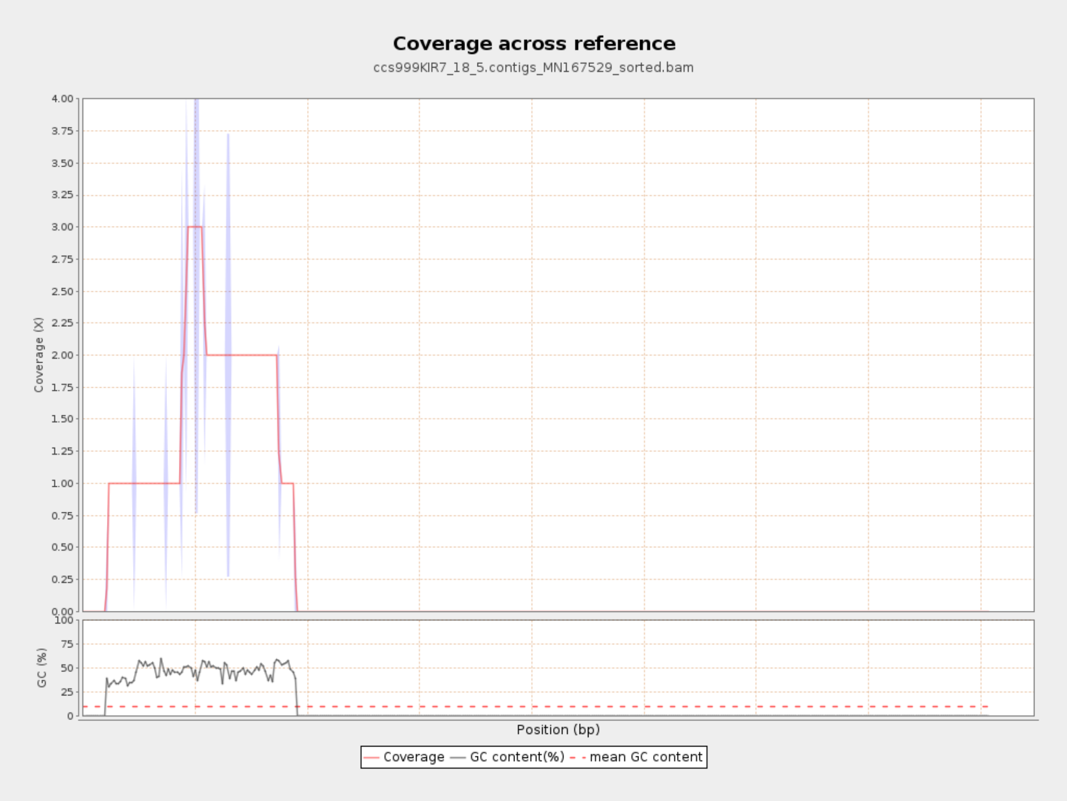

4. Results : Coverage Histogram

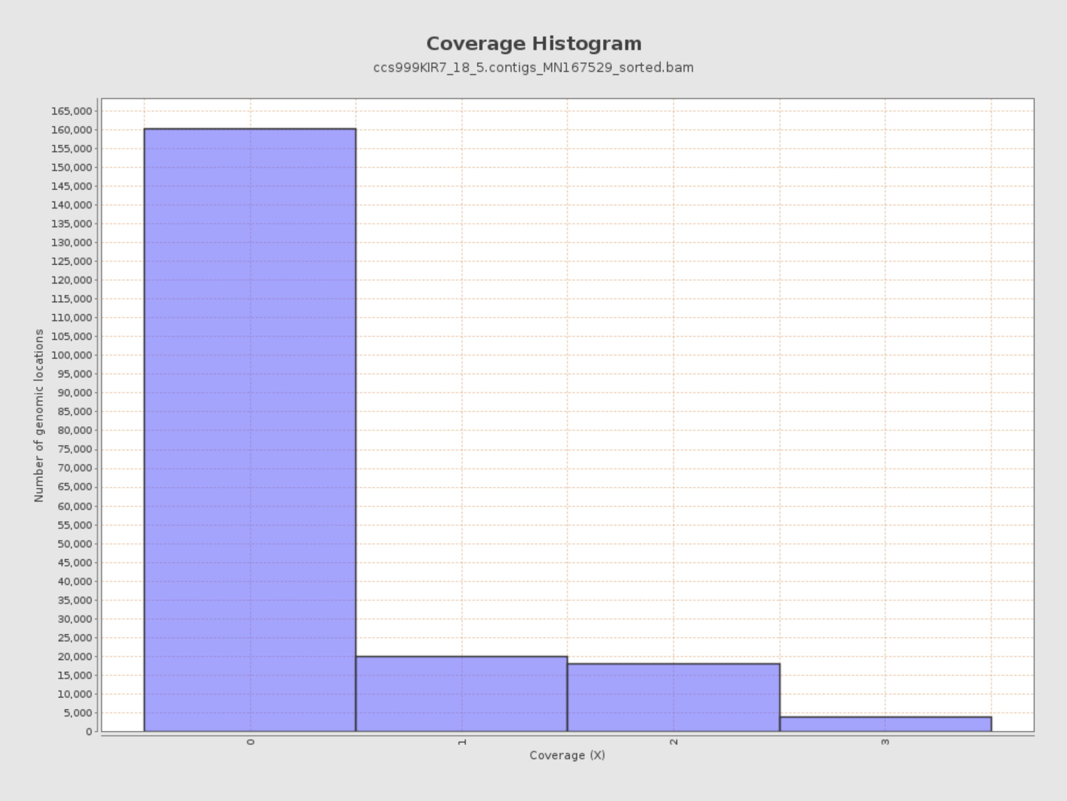

5. Results : Coverage Histogram (0-50X)

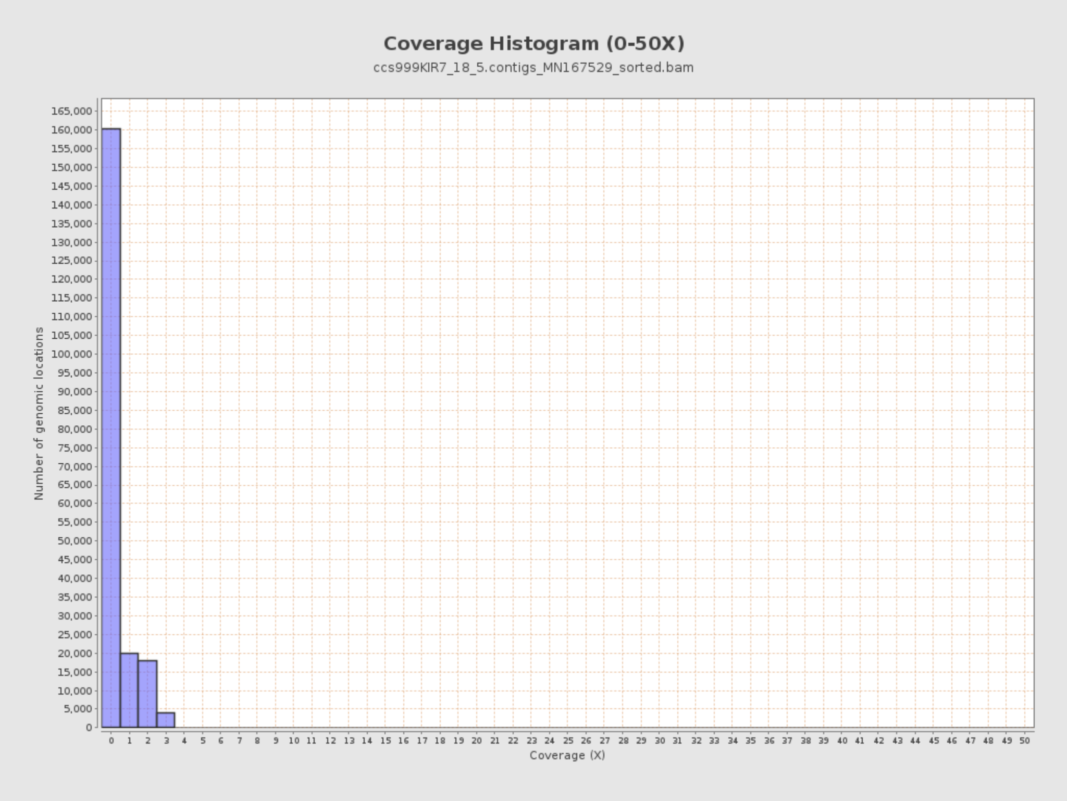

6. Results : Genome Fraction Coverage

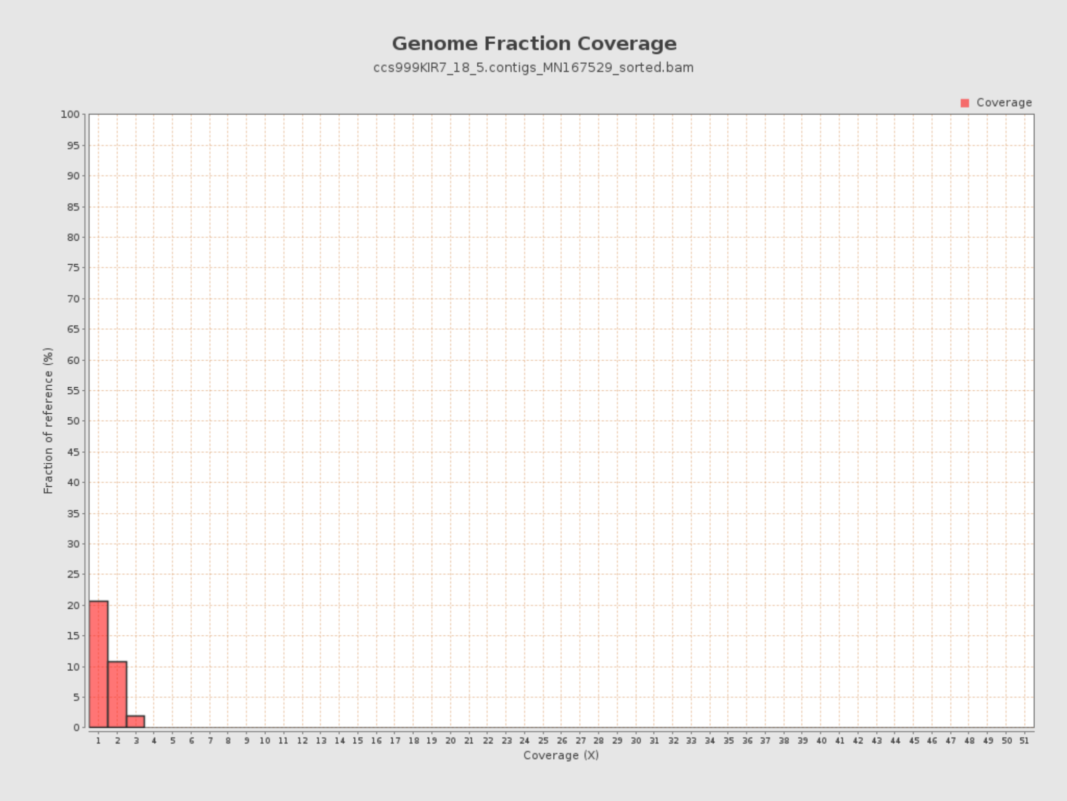

7. Results : Duplication Rate Histogram

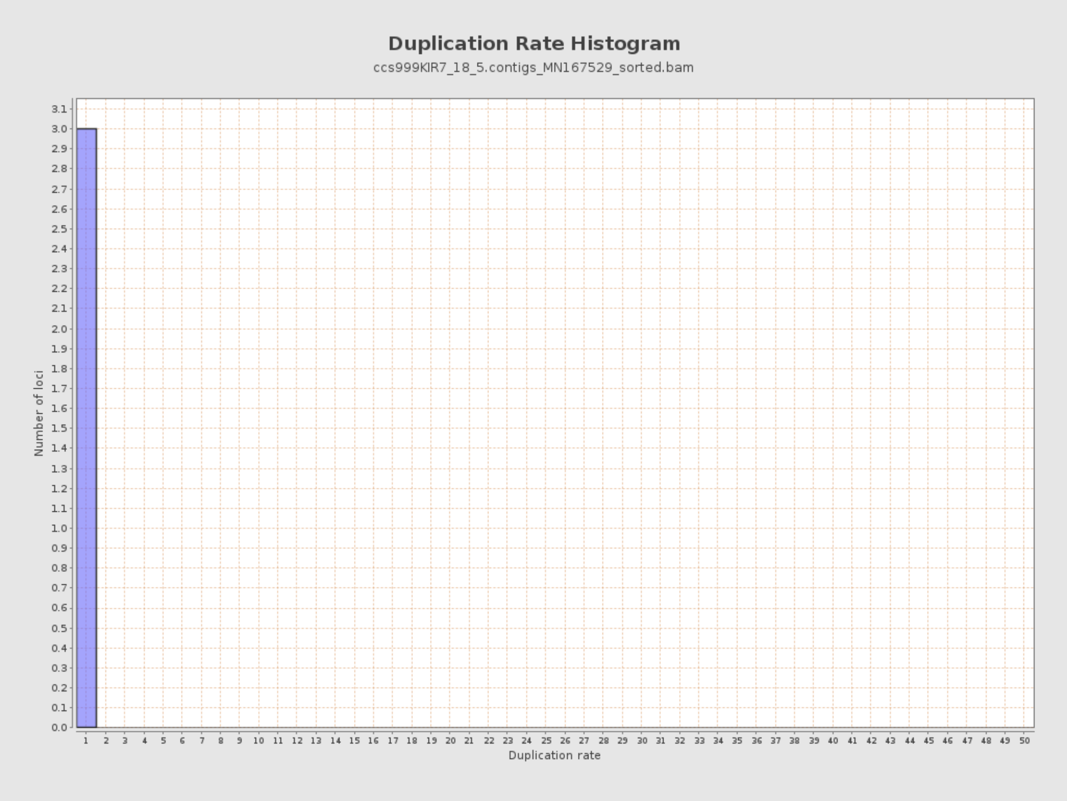

8. Results : Mapped Reads Nucleotide Content

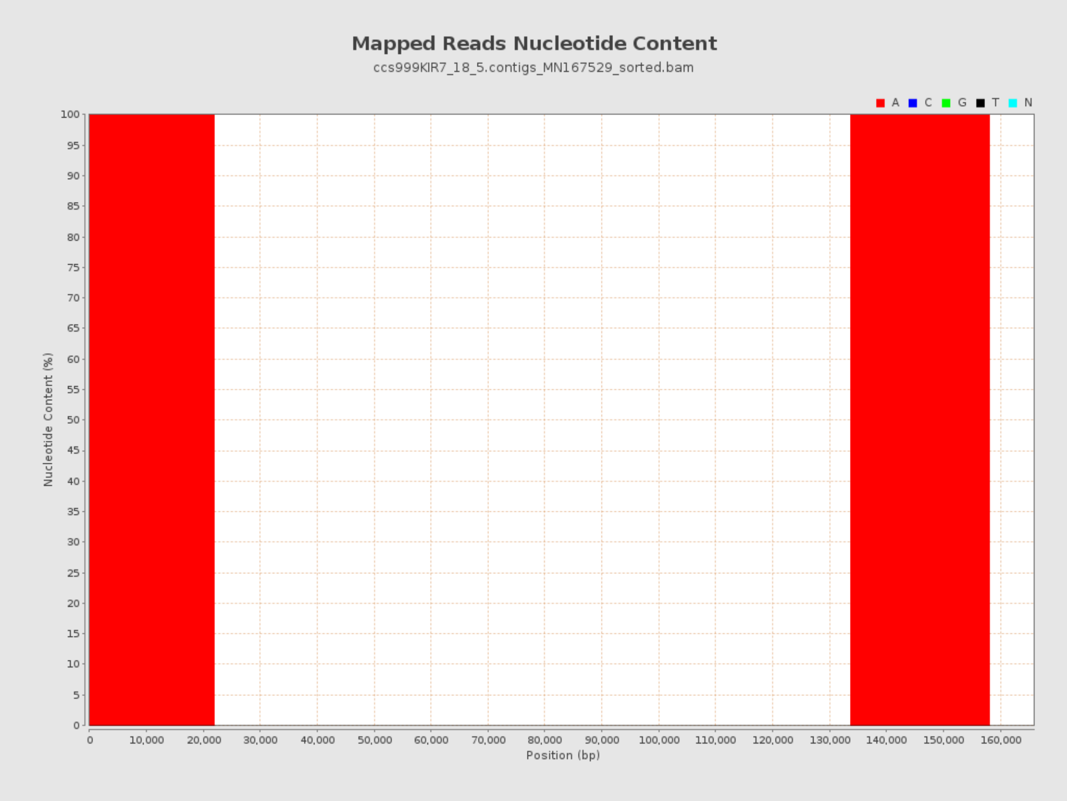

9. Results : Mapped Reads GC-content Distribution

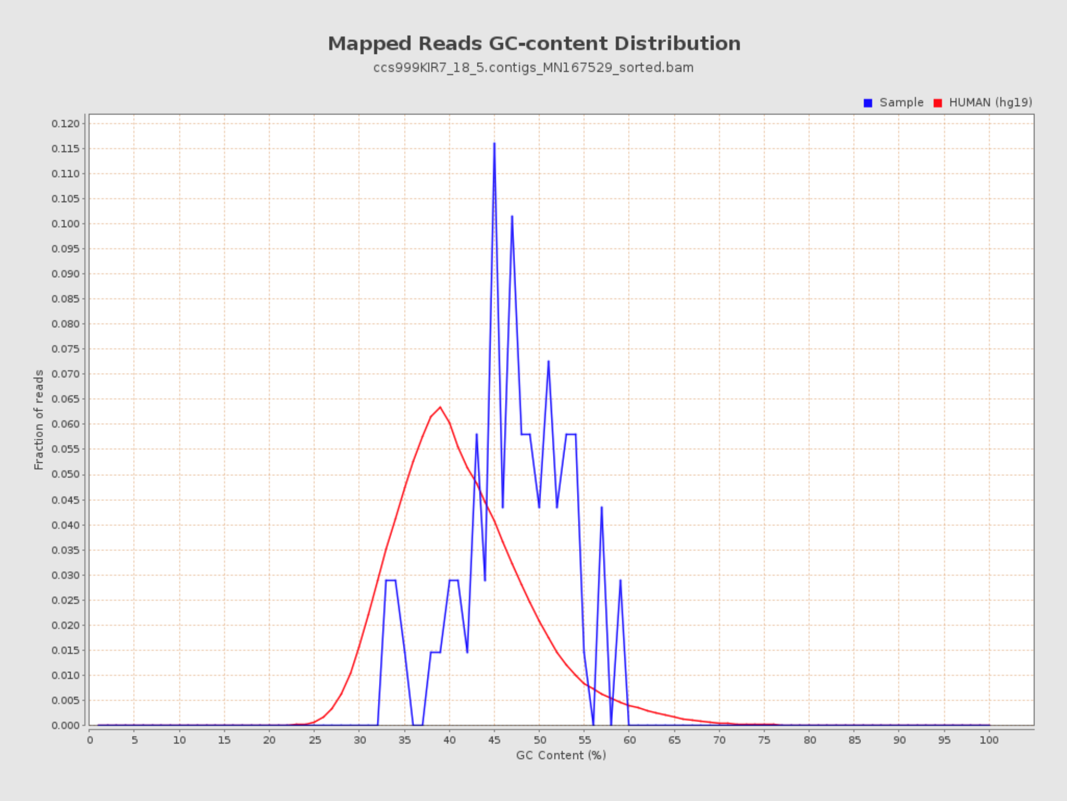

10. Results : Mapped Reads Clipping Profile

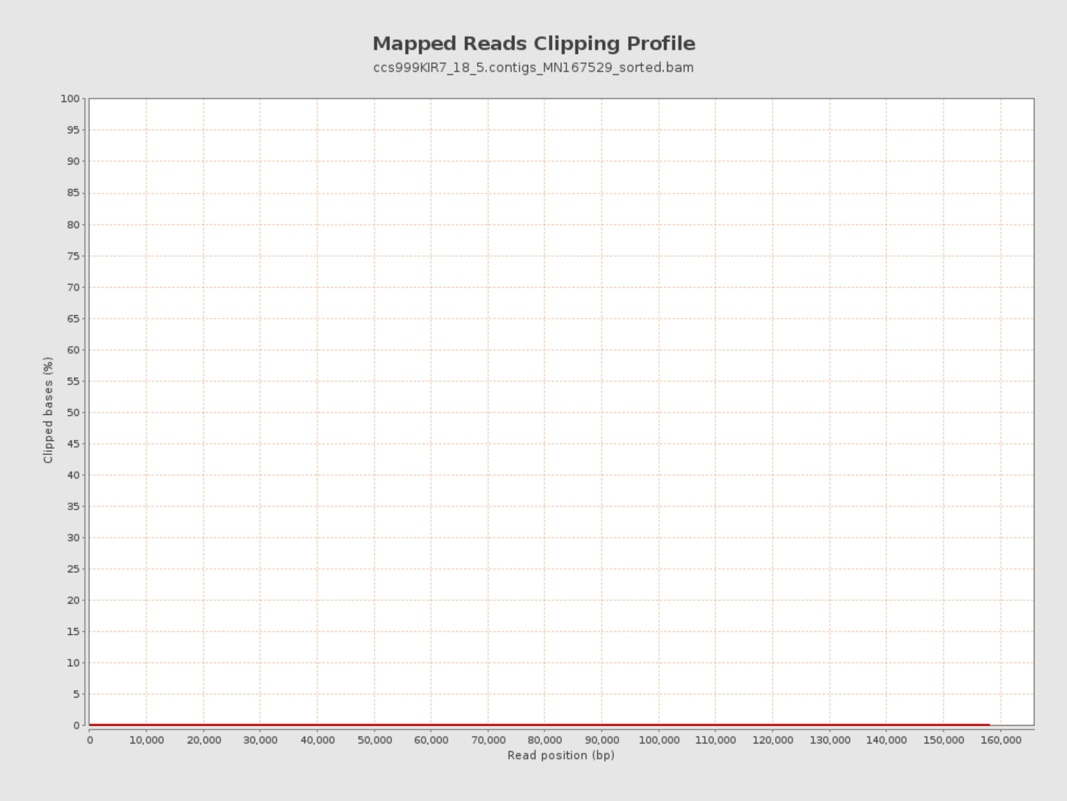

11. Results : Homopolymer Indels

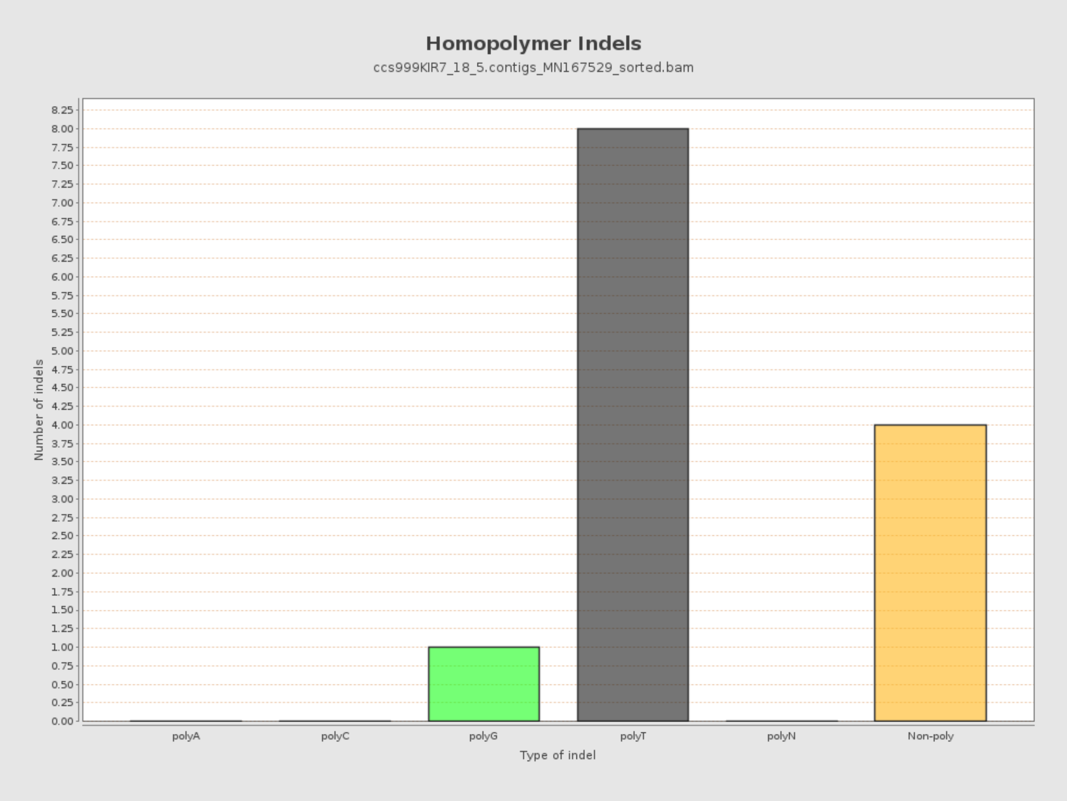

12. Results : Mapping Quality Across Reference

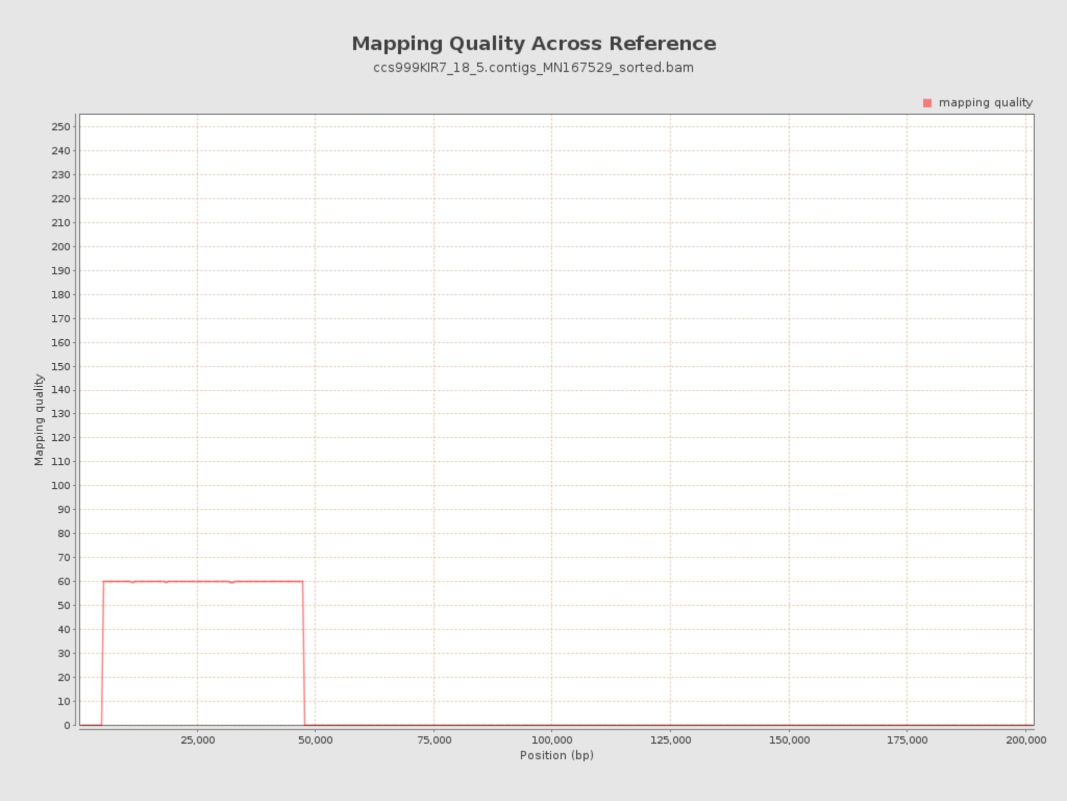

13. Results : Mapping Quality Histogram

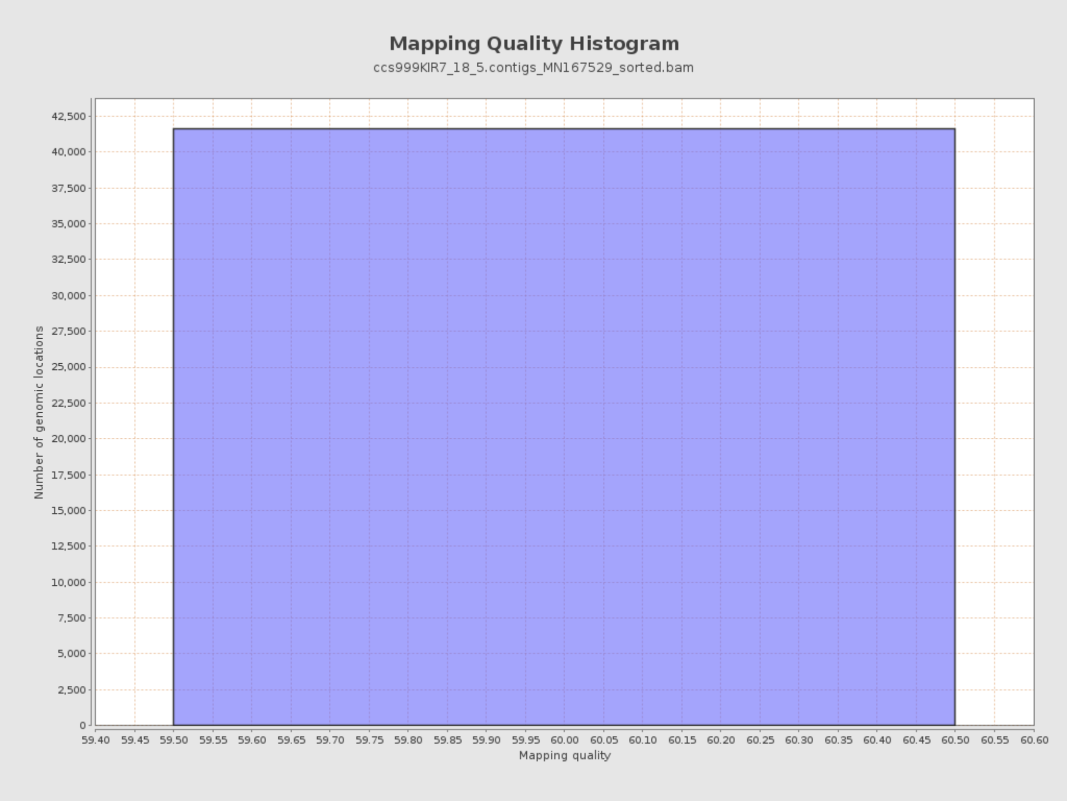

Supplement: Supplementary file 7 [file DataSheet_3.zip › SF1c/ccs999KIR7_18_5.contigs_MN167529_reports/ccs999KIR7_18_5.contigs_MN167529_qualimap.pdf]
