# Supplementary material for: Efficient Sequencing, Assembly, and Annotation of Human KIR Haplotypes
Source: Front Immunol. 2020 Oct 9;11:582927. doi: 10.3389/fimmu.2020.582927 (PMC7581912; doi:10.3389/fimmu.2020.582927)

Qualimap Analysis Results

BAM QC analysis

Generated by Qualimap v.2.2.1

2020/04/21 17:47:04

1. Input data & parameters

1.1. QualiMap command line

qualimap bamqc -bam ccs999KIR7_18_5.contigs_MN167528_sorted.bam -nw 400 -hm 3

1.2. Alignment

Command line:	bwa mem -k1800 -W9000 -r10 -A1 -B100 -O40 -E40 -L50 -t7 MN167528.fasta ccs999KIR7_18_5.contigs.fasta.gz
Draw chromosome limits:	no
Analyze overlapping paired-end reads:	no
Program:	bwa (0.7.17-r1188)
Analysis date:	Tue Apr 21 17:47:03 GMT 2020
Size of a homopolymer:	3
Skip duplicate alignments:	no
Number of windows:	400
BAM file:	ccs999KIR7_18_5.contigs_MN167528_sorted.bam

2. Summary

2.1. Globals

Reference size	127,281
Number of reads	10
Mapped reads	10 / 100%
Unmapped reads	0 / 0%
Mapped paired reads	0 / 0%
Read min/max/mean length	12,023 / 82,396 / 27,014.8
Duplicated reads (estimated)	0 / 0%
Duplication rate	0%
Clipped reads	3 / 30%

2.2. ACGT Content

Number/percentage of A's	73,050 / 27.1%
Number/percentage of C's	60,099 / 22.3%
Number/percentage of T's	69,930 / 25.94%
Number/percentage of G's	66,461 / 24.66%
Number/percentage of N's	0 / 0%
GC Percentage	46.95%

2.3. Coverage

Mean	2.1178
Standard Deviation	0.9297

2.4. Mapping Quality

Mean Mapping Quality	56.39

2.5. Mismatches and indels

General error rate	0.01%
Mismatches	25
Insertions	3
Mapped reads with at least one insertion	30%
Deletions	18
Mapped reads with at least one deletion	70%
Homopolymer indels	100%

2.6. Chromosome stats

Name	Length	Mapped bases	Mean coverage	Standard deviation
MN167528	127281	269558	2.1178	0.9297

3. Results : Coverage across reference

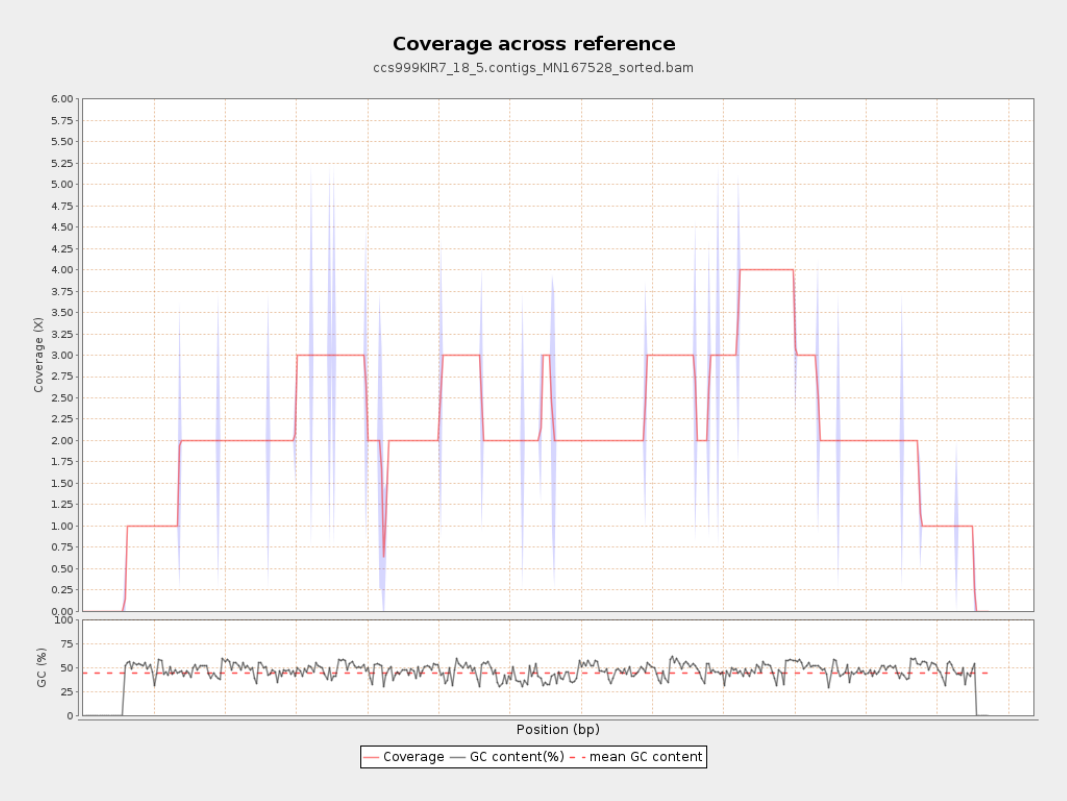

4. Results : Coverage Histogram

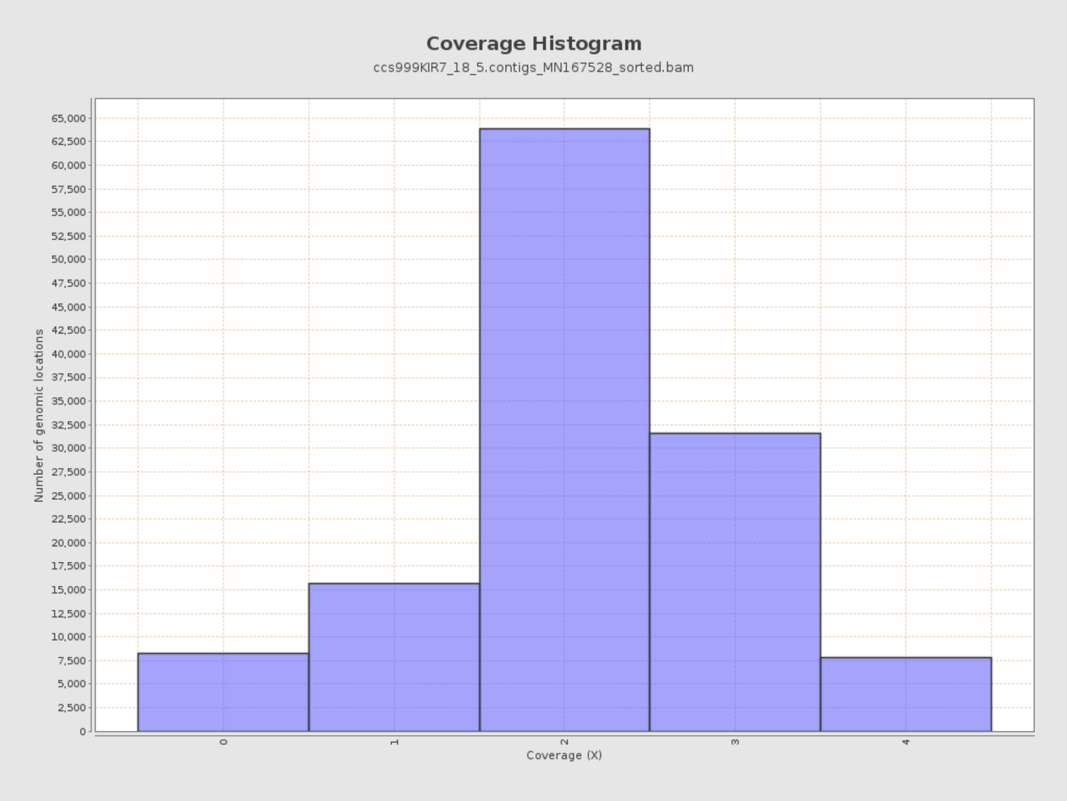

5. Results : Coverage Histogram (0-50X)

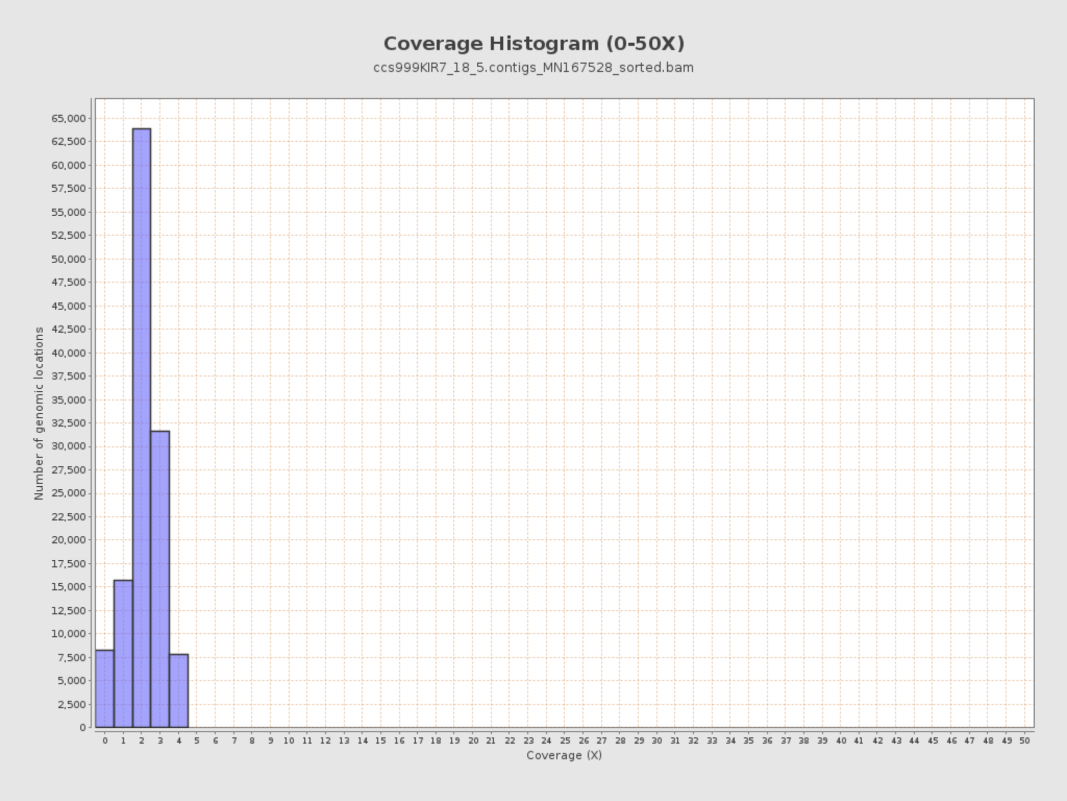

6. Results : Genome Fraction Coverage

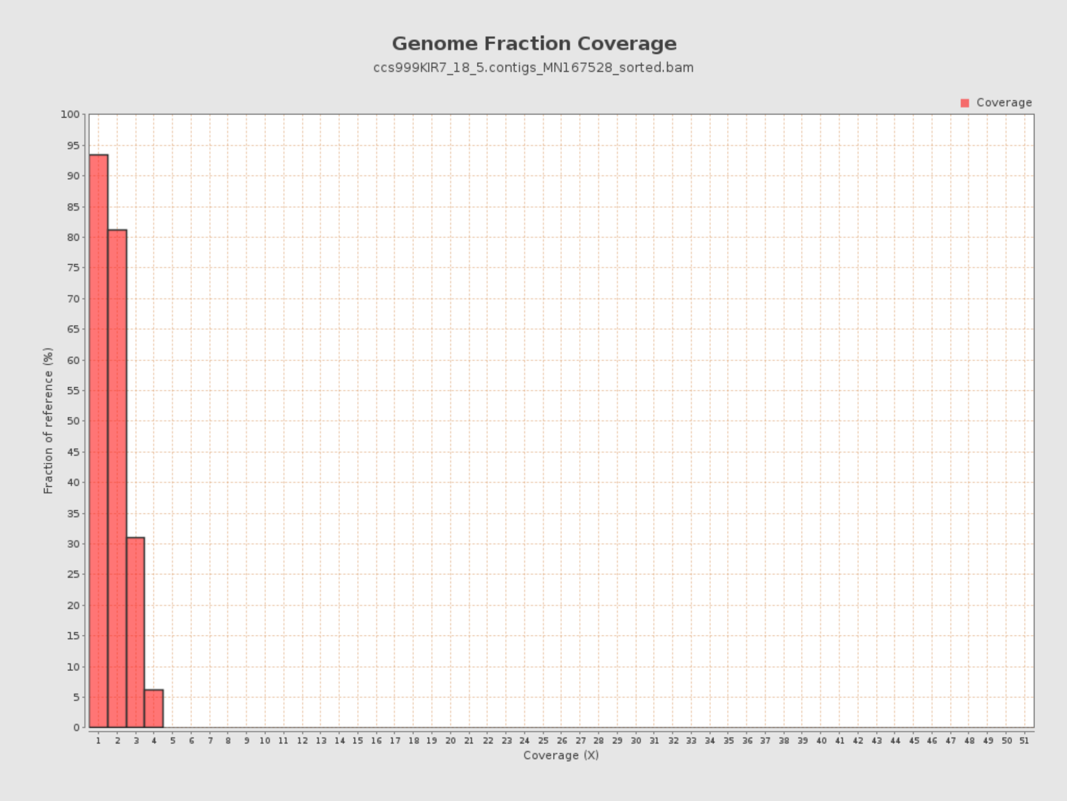

7. Results : Duplication Rate Histogram

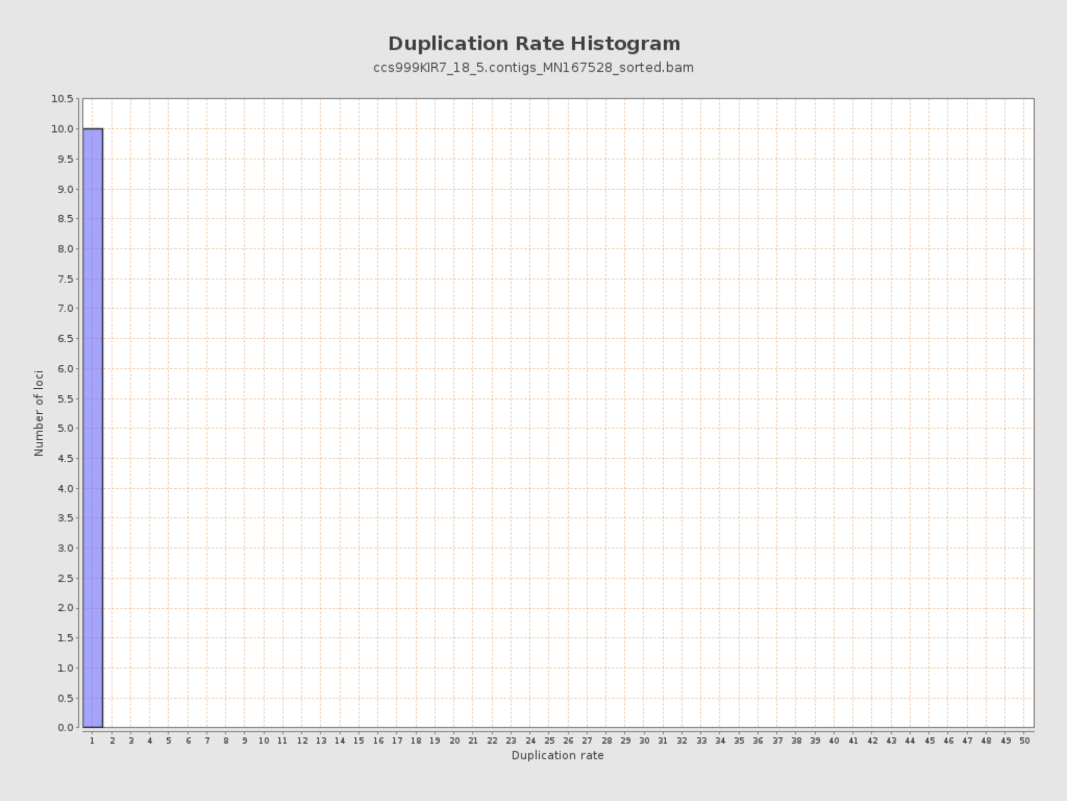

8. Results : Mapped Reads Nucleotide Content

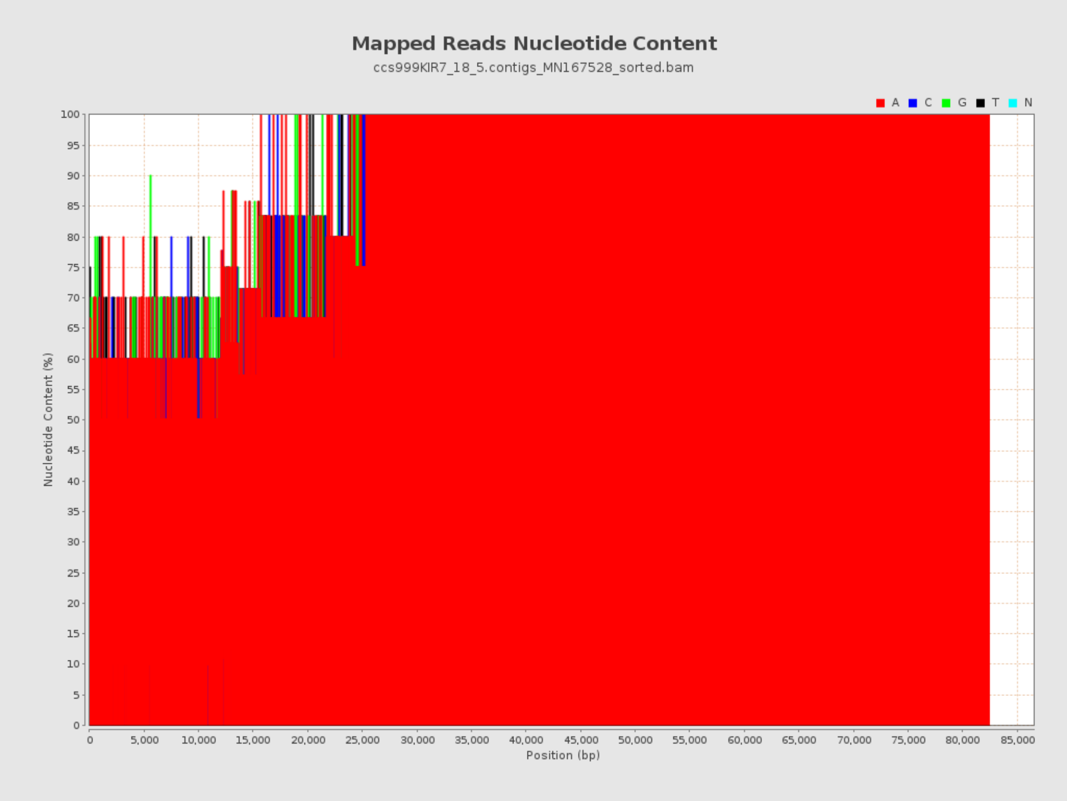

9. Results : Mapped Reads GC-content Distribution

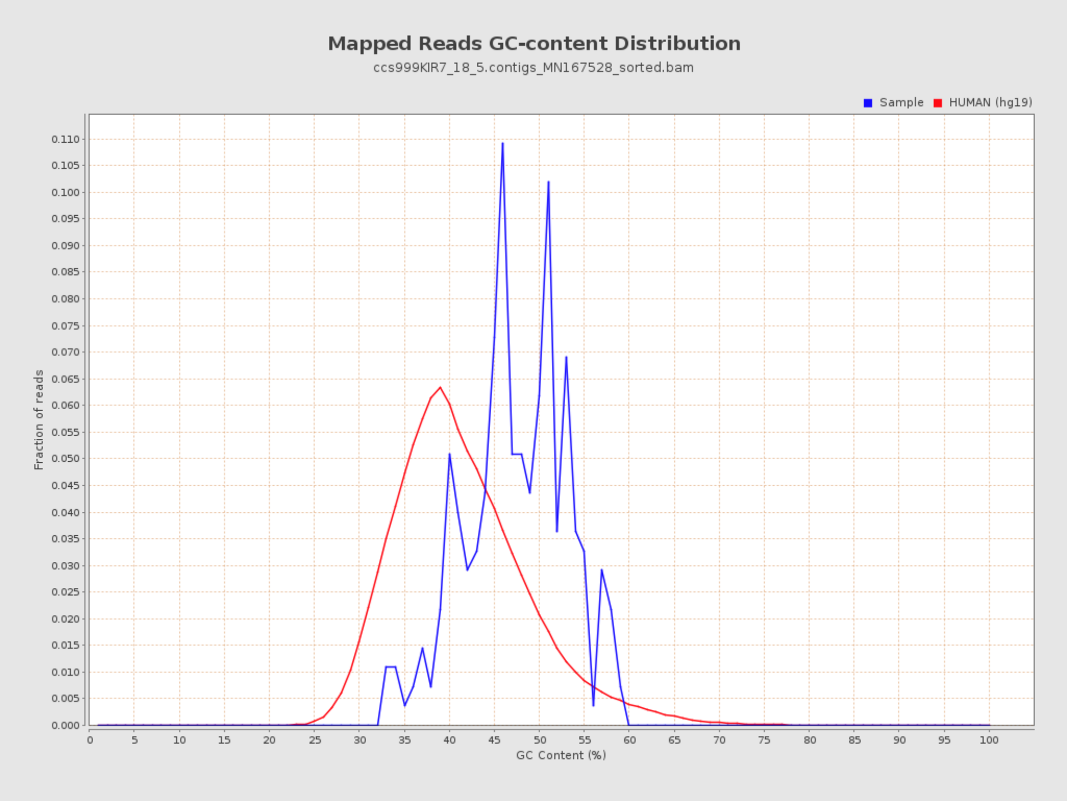

10. Results : Mapped Reads Clipping Profile

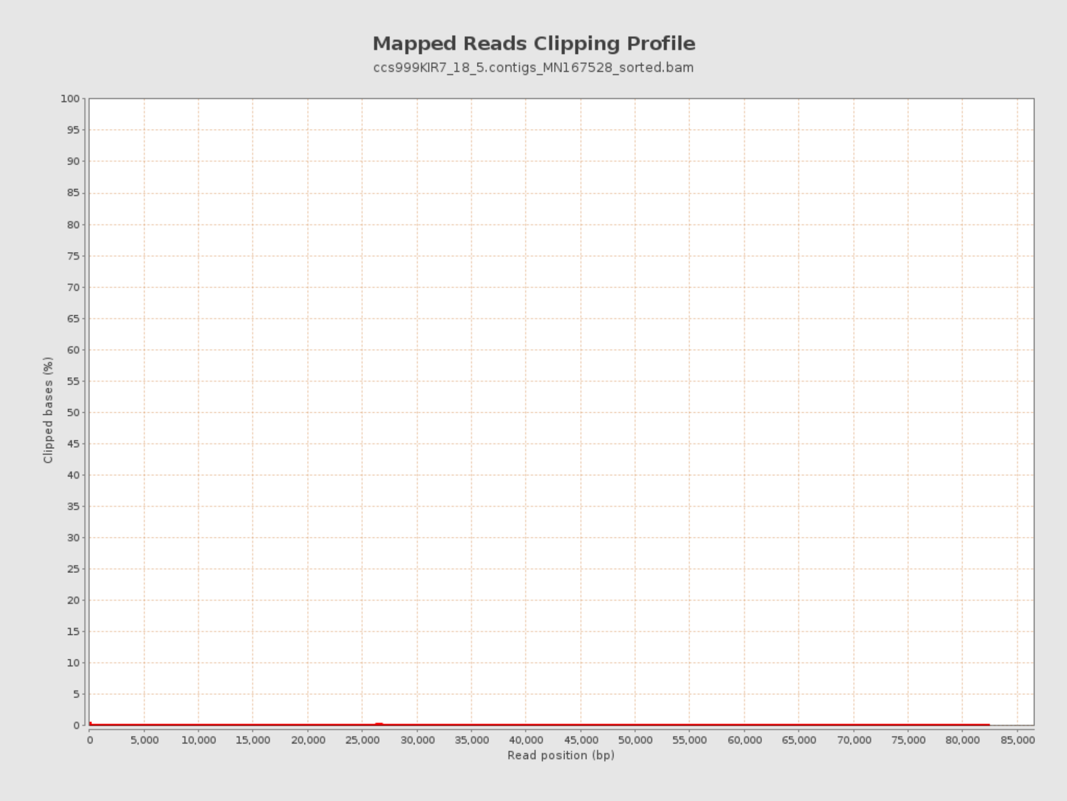

11. Results : Homopolymer Indels

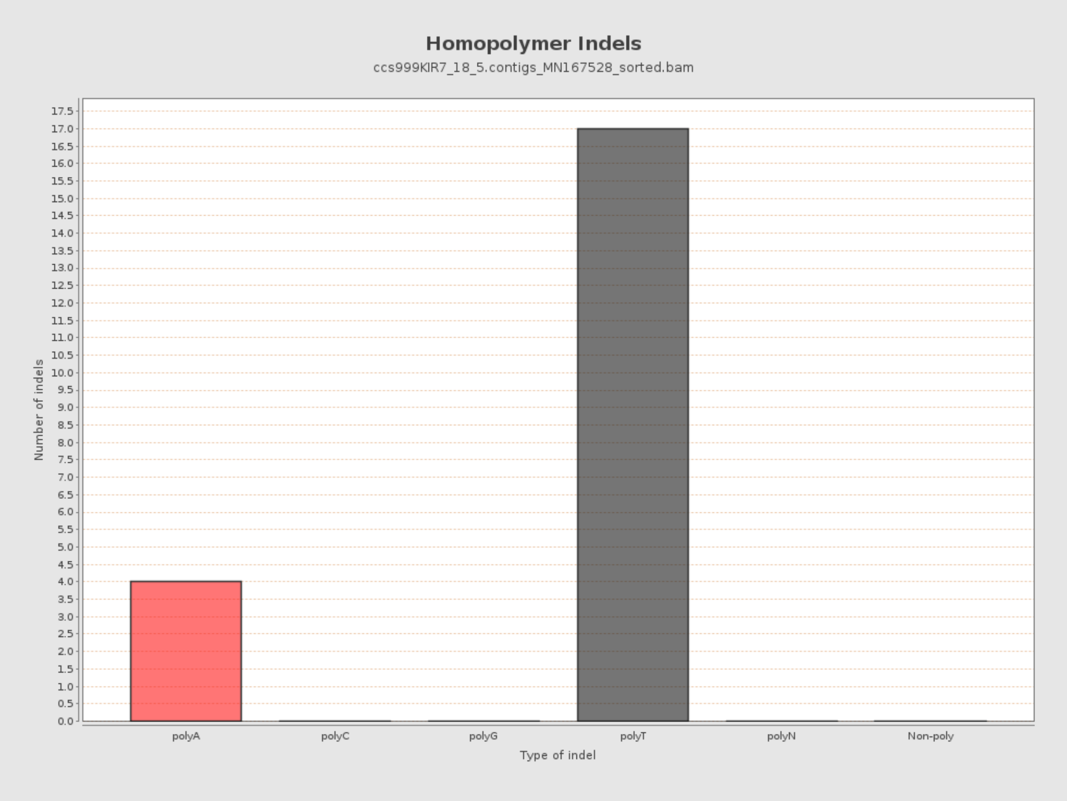

12. Results : Mapping Quality Across Reference

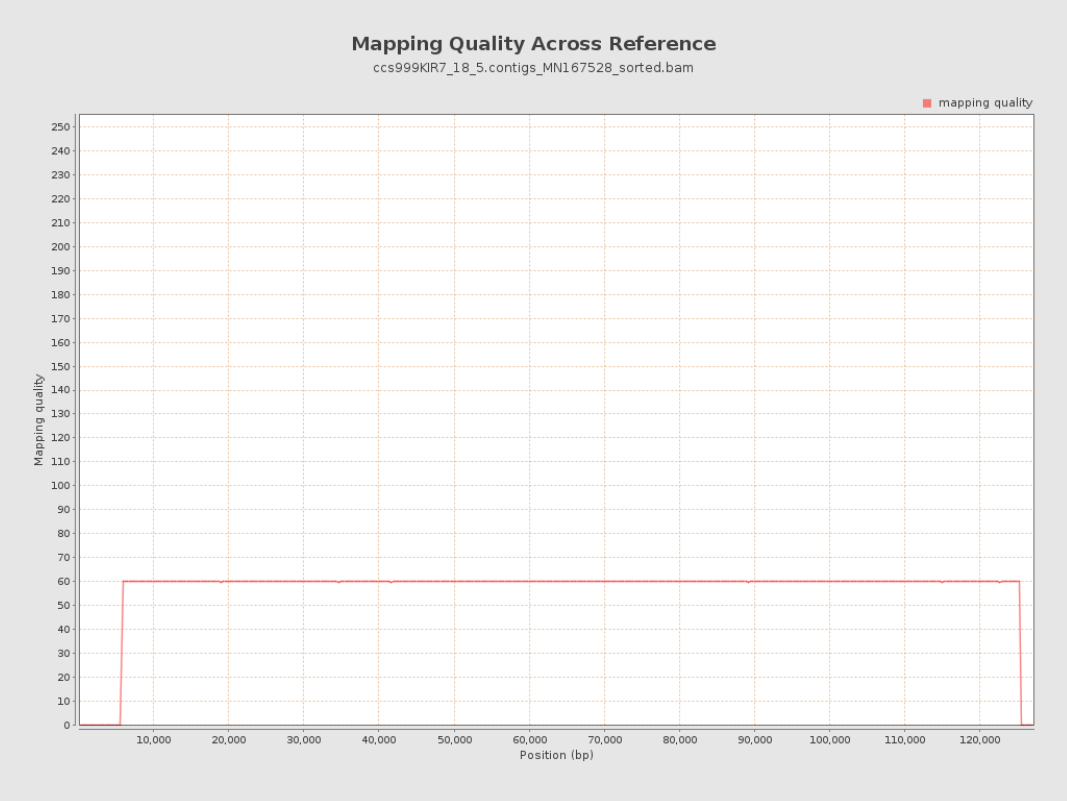

13. Results : Mapping Quality Histogram

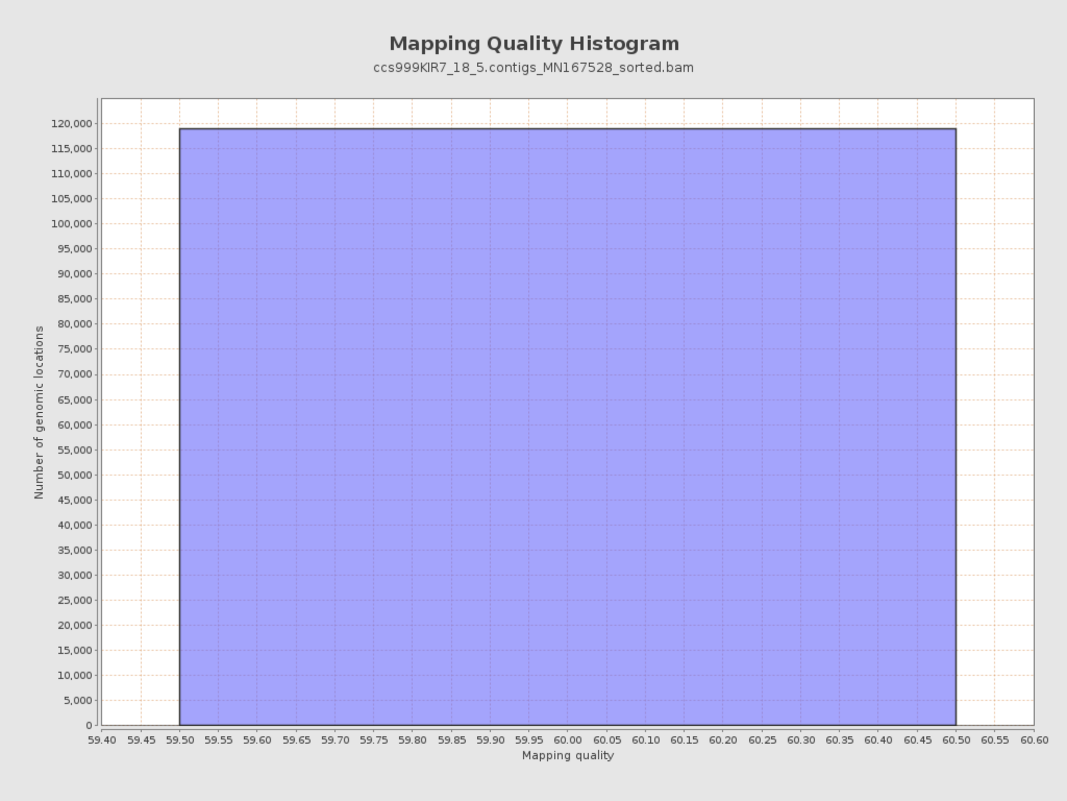

Supplement: Supplementary file 7 [file DataSheet_3.zip › SF1c/ccs999KIR7_18_5.contigs_MN167528_reports/ccs999KIR7_18_5.contigs_MN167528_qualimap.pdf]
