# Supplementary material for: Efficient Sequencing, Assembly, and Annotation of Human KIR Haplotypes
Source: Front Immunol. 2020 Oct 9;11:582927. doi: 10.3389/fimmu.2020.582927 (PMC7581912; doi:10.3389/fimmu.2020.582927)

Qualimap Analysis Results

BAM QC analysis

Generated by Qualimap v.2.2.1

2020/04/21 17:48:03

1. Input data & parameters

1.1. QualiMap command line

qualimap bamqc -bam ccs999KIR7_18_6.contigs_MN167518_sorted.bam -nw 400 -hm 3

1.2. Alignment

Command line:	bwa mem -k1800 -W9000 -r10 -A1 -B100 -O40 -E40 -L50 -t7 MN167518.fasta ccs999KIR7_18_6.contigs.fasta.gz
Draw chromosome limits:	no
Analyze overlapping paired-end reads:	no
Program:	bwa (0.7.17-r1188)
Analysis date:	Tue Apr 21 17:48:02 GMT 2020
Size of a homopolymer:	3
Skip duplicate alignments:	no
Number of windows:	400
BAM file:	ccs999KIR7_18_6.contigs_MN167518_sorted.bam

2. Summary

2.1. Globals

Reference size	178,642
Number of reads	19
Mapped reads	19 / 100%
Unmapped reads	0 / 0%
Mapped paired reads	0 / 0%
Read min/max/mean length	9,378 / 105,322 / 26,814.68
Duplicated reads (estimated)	0 / 0%
Duplication rate	0%
Clipped reads	8 / 42.11%

2.2. ACGT Content

Number/percentage of A's	118,258 / 26.85%
Number/percentage of C's	99,153 / 22.51%
Number/percentage of T's	114,420 / 25.97%
Number/percentage of G's	108,689 / 24.67%
Number/percentage of N's	0 / 0%
GC Percentage	47.18%

2.3. Coverage

Mean	2.4661
Standard Deviation	1.2833

2.4. Mapping Quality

Mean Mapping Quality	56.99

2.5. Mismatches and indels

General error rate	0.02%
Mismatches	64
Insertions	9
Mapped reads with at least one insertion	42.11%
Deletions	29
Mapped reads with at least one deletion	78.95%
Homopolymer indels	97.37%

2.6. Chromosome stats

**Name**	**Length**	**Mapped bases**	**Mean coverage**	**Standard deviation**
MN167518	178642	440556	2.4661	1.2833

3. Results : Coverage across reference

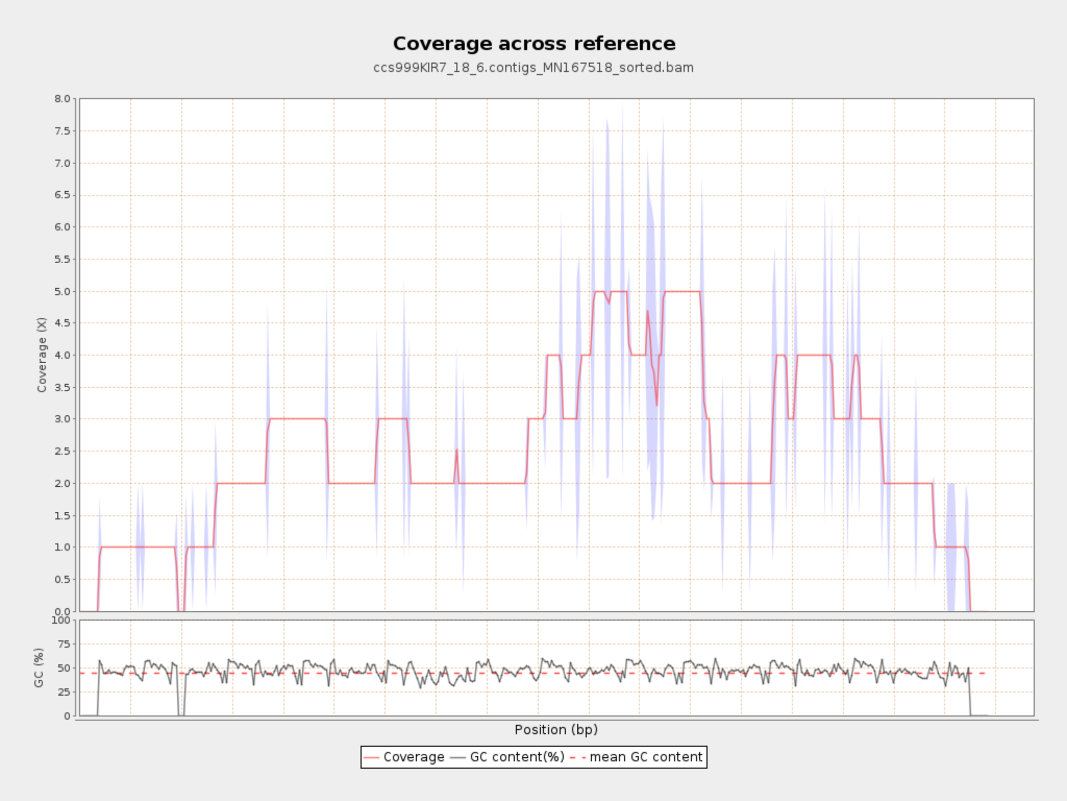

4. Results : Coverage Histogram

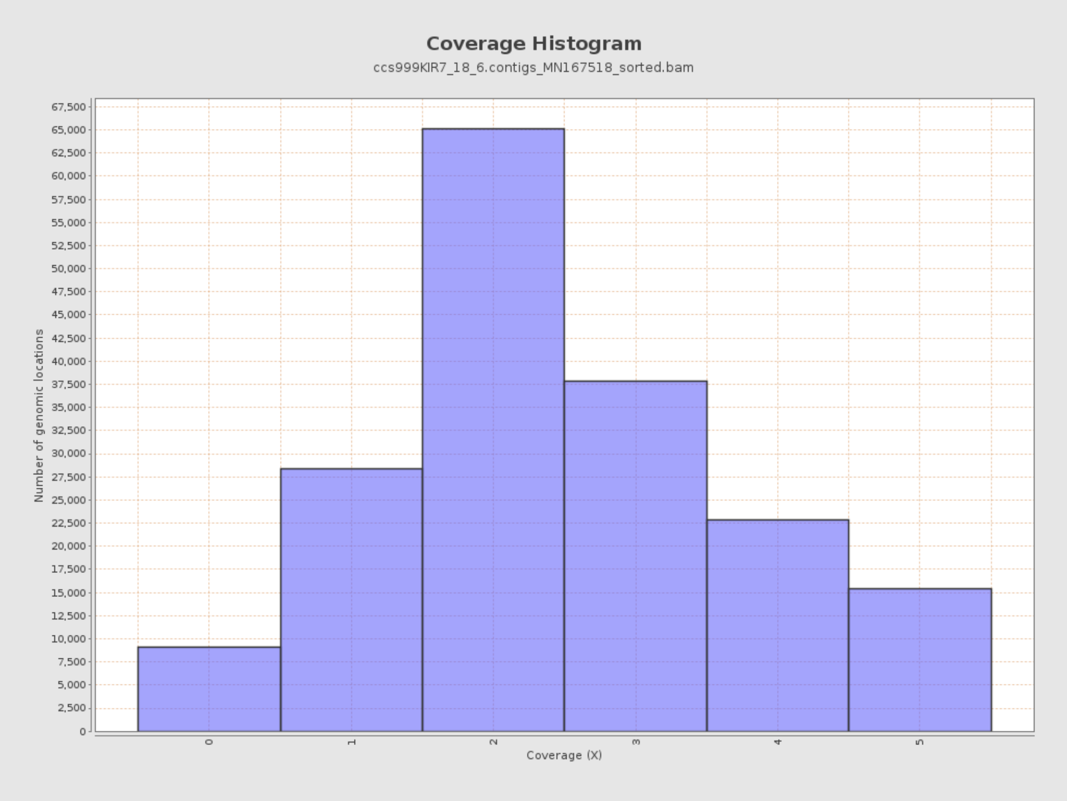

5. Results : Coverage Histogram (0-50X)

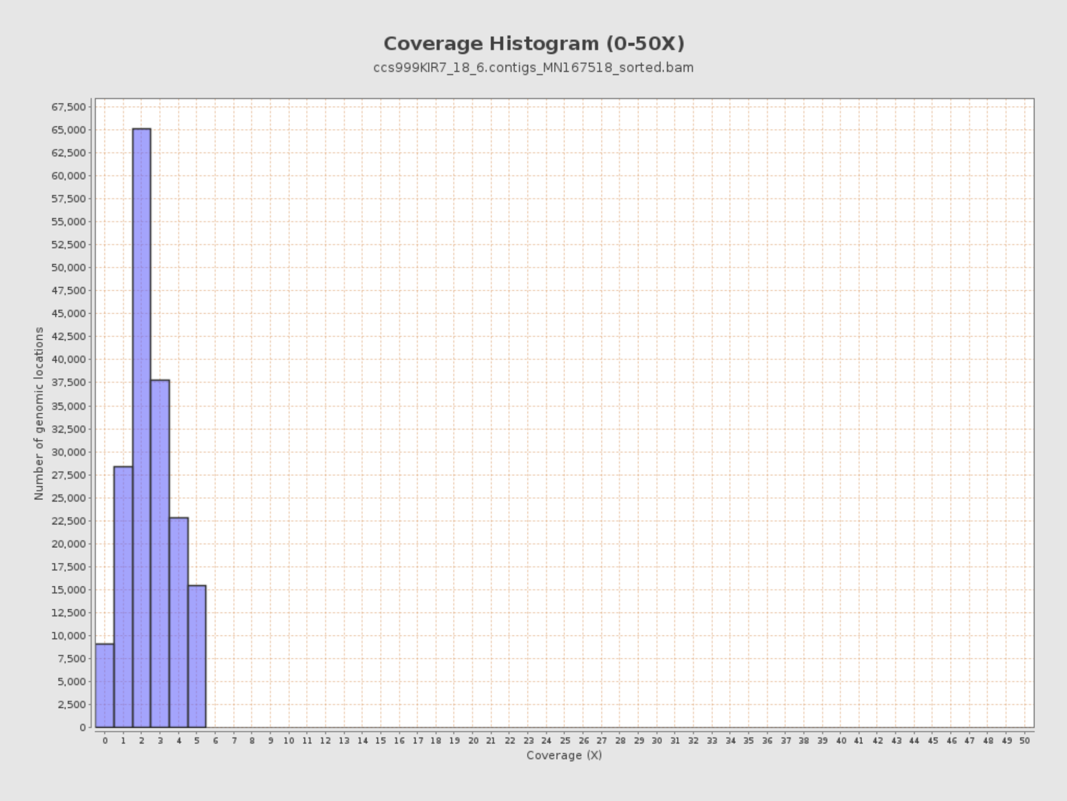

6. Results : Genome Fraction Coverage

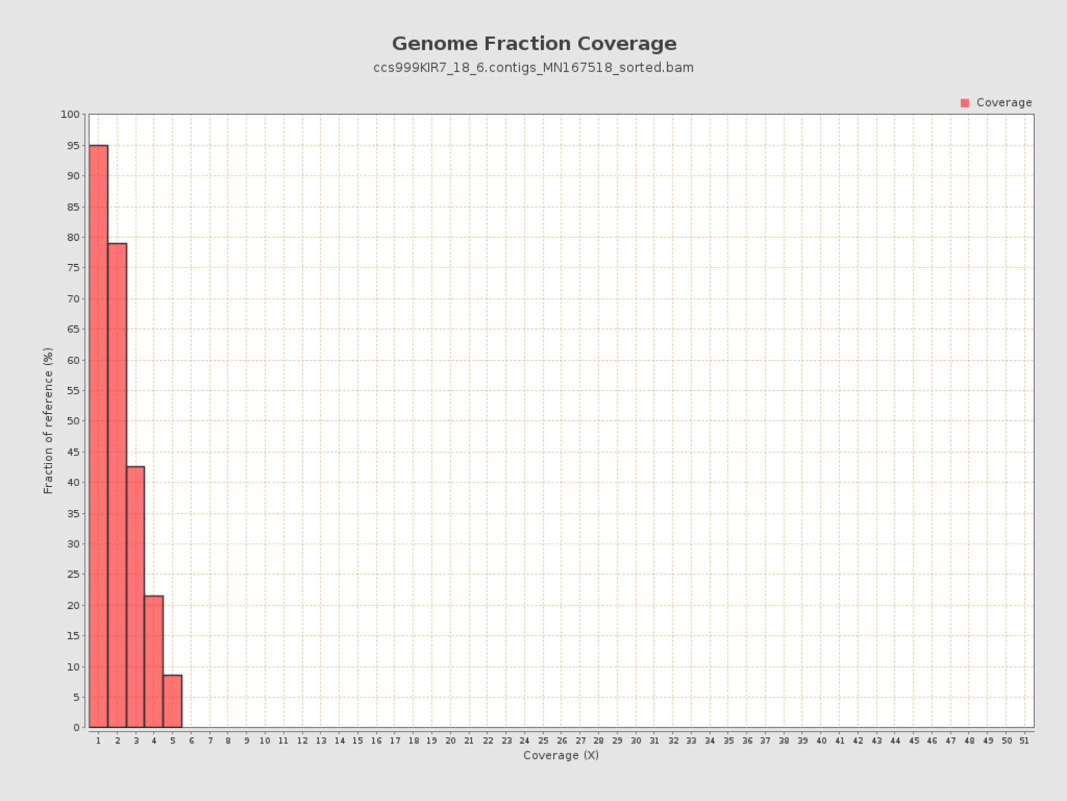

7. Results : Duplication Rate Histogram

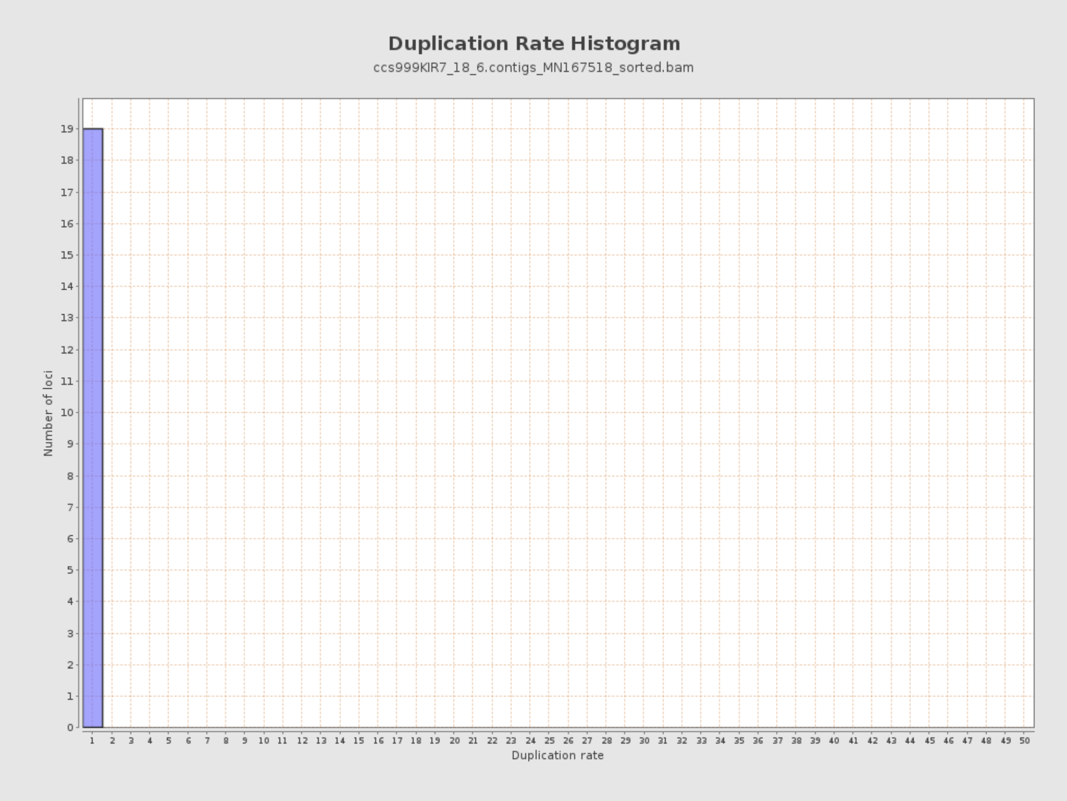

8. Results : Mapped Reads Nucleotide Content

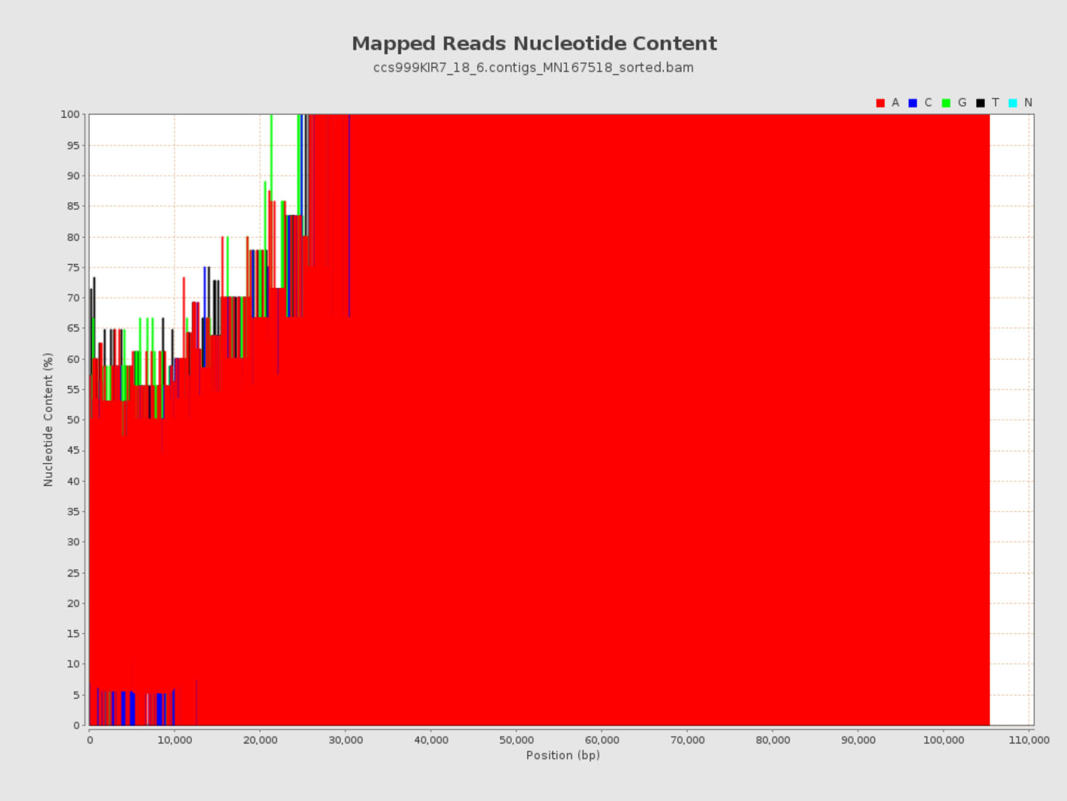

9. Results : Mapped Reads GC-content Distribution

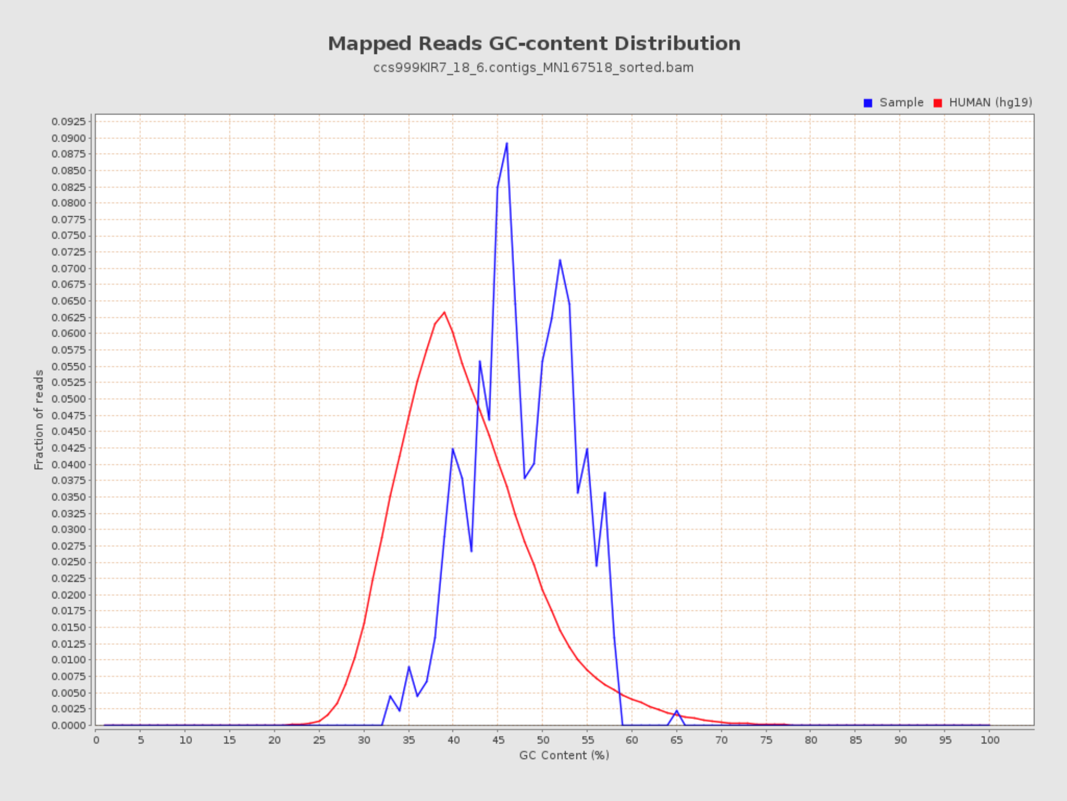

10. Results : Mapped Reads Clipping Profile

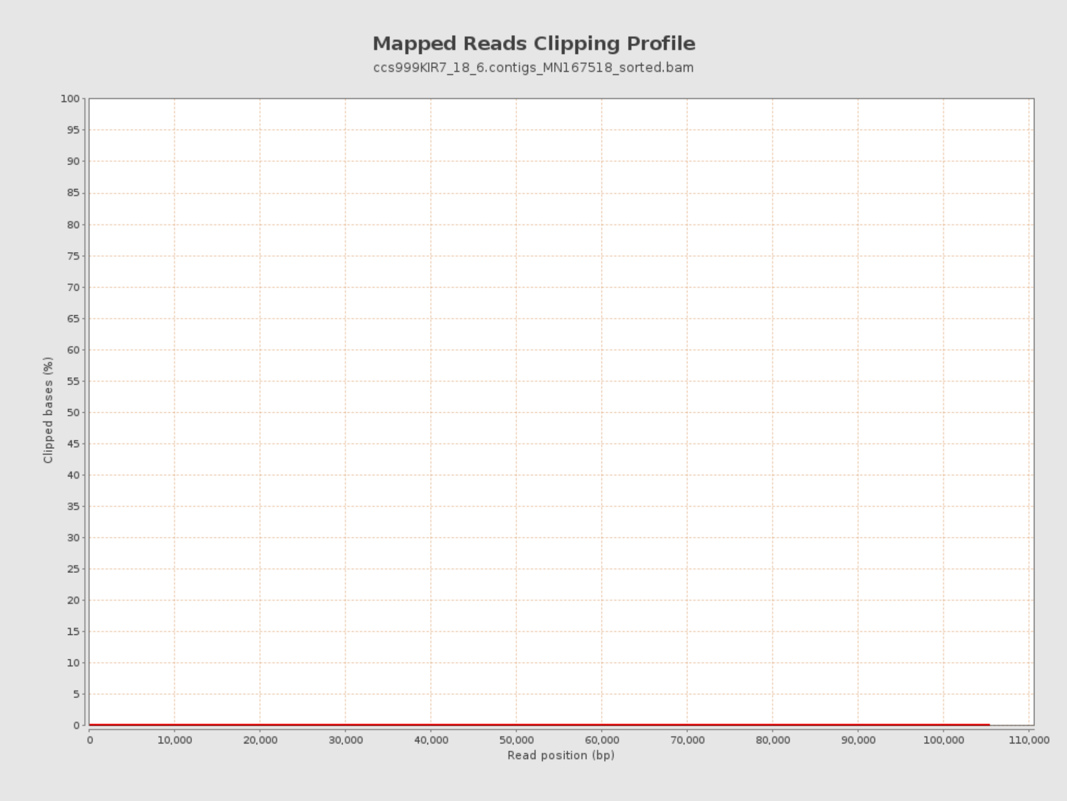

11. Results : Homopolymer Indels

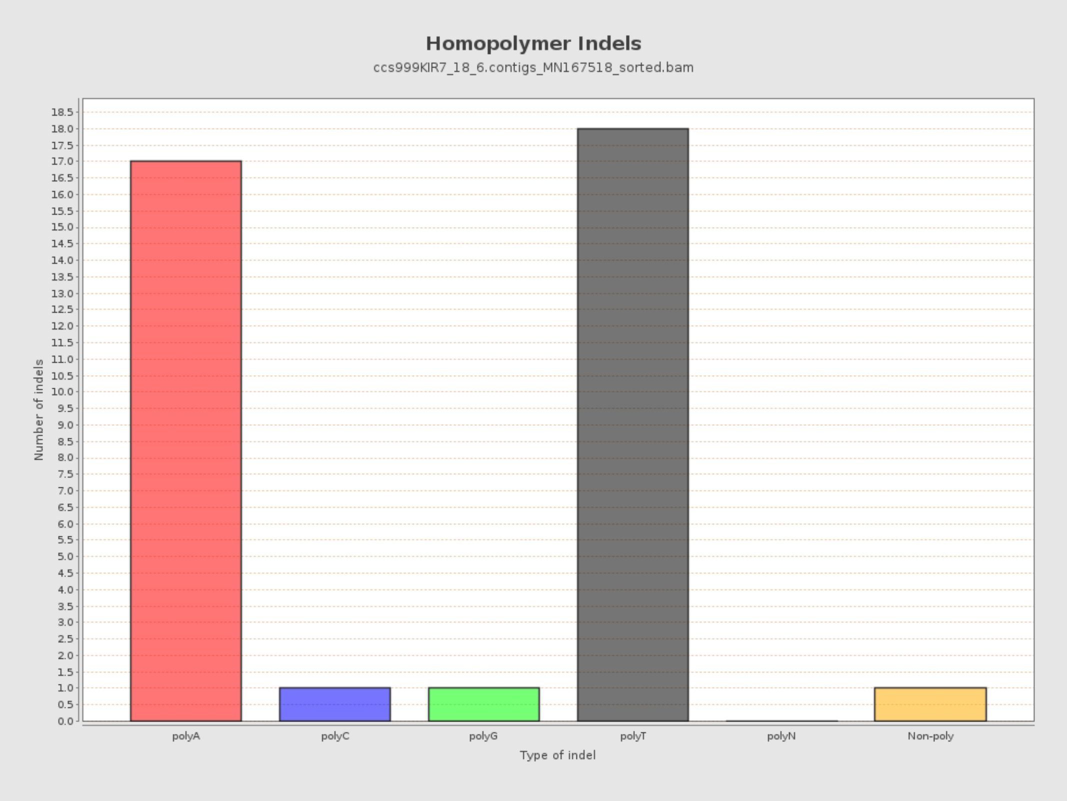

12. Results : Mapping Quality Across Reference

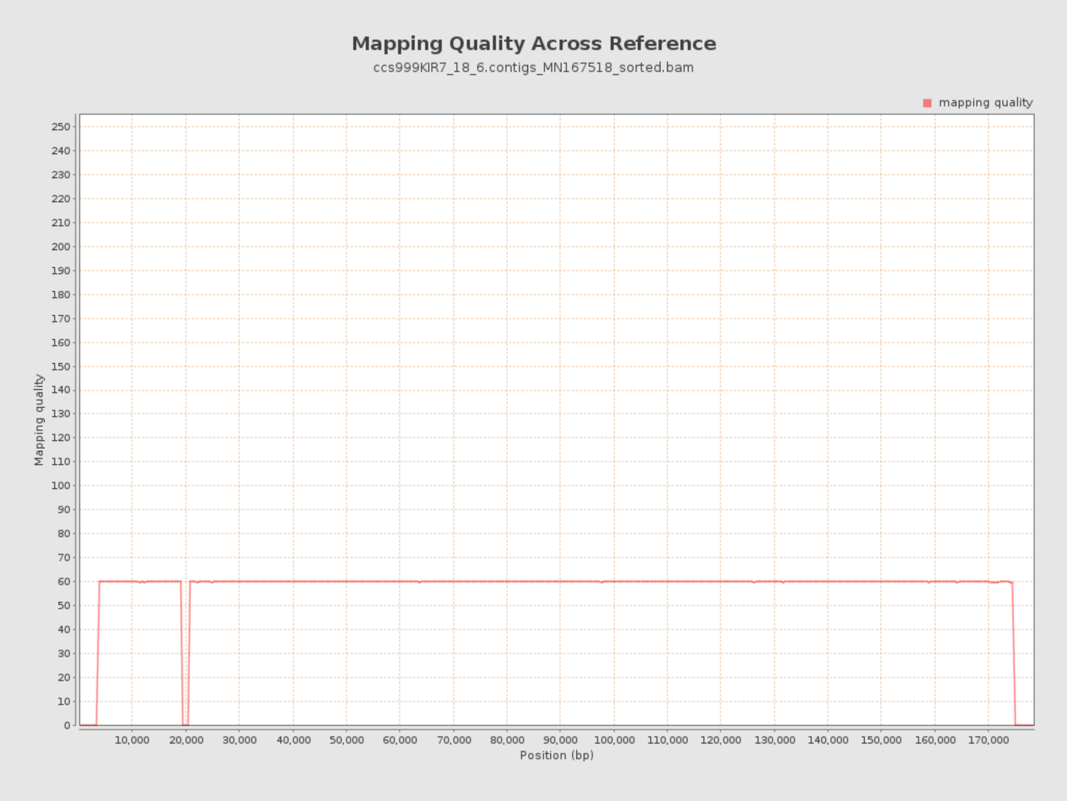

13. Results : Mapping Quality Histogram

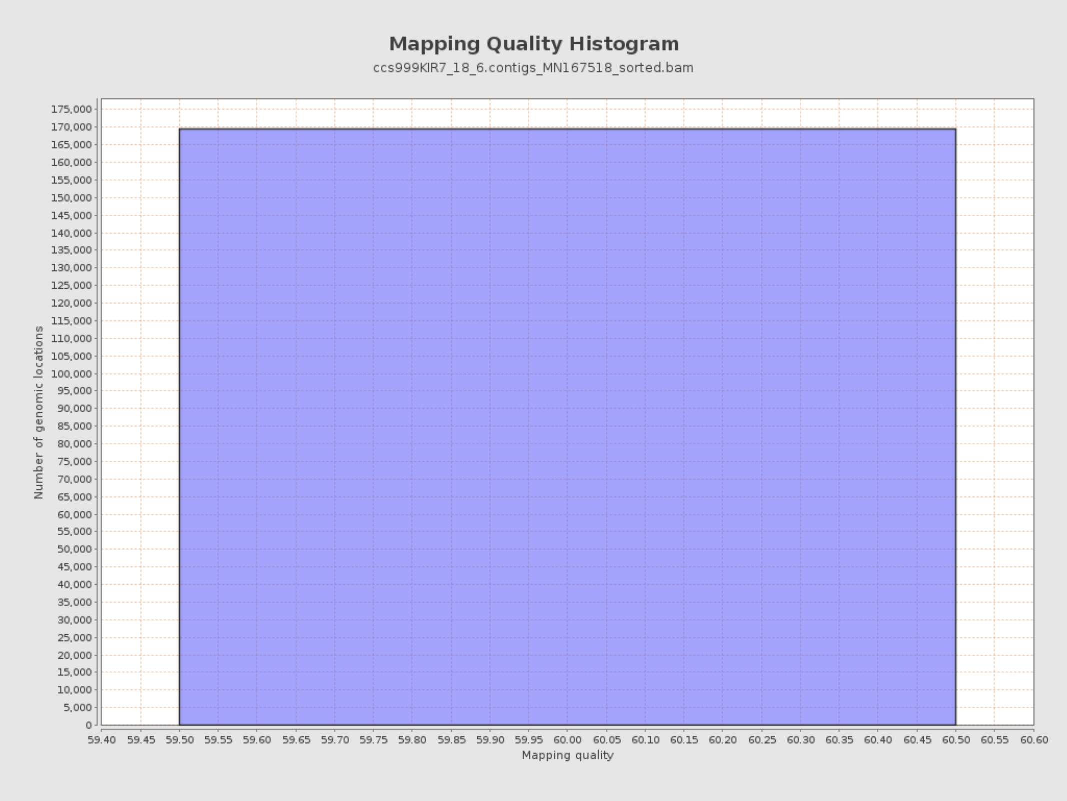

Supplement: Supplementary file 7 [file DataSheet_3.zip › SF1c/ccs999KIR7_18_6.contigs_MN167518_reports/ccs999KIR7_18_6.contigs_MN167518_qualimap.pdf]
