# Supplementary material for: Efficient Sequencing, Assembly, and Annotation of Human KIR Haplotypes
Source: Front Immunol. 2020 Oct 9;11:582927. doi: 10.3389/fimmu.2020.582927 (PMC7581912; doi:10.3389/fimmu.2020.582927)

Qualimap Analysis Results

BAM QC analysis

Generated by Qualimap v.2.2.1

2020/04/21 17:45:07

1. Input data & parameters

1.1. QualiMap command line

qualimap bamqc -bam ccs999KIR7_18_3.contigs_MN167520_sorted.bam -nw 400 -hm 3

1.2. Alignment

Command line:	bwa mem -k1800 -W9000 -r10 -A1 -B100 -O40 -E40 -L50 -t7 MN167520.fasta ccs999KIR7_18_3.contigs.fasta.gz
Draw chromosome limits:	no
Analyze overlapping paired-end reads:	no
Program:	bwa (0.7.17-r1188)
Analysis date:	Tue Apr 21 17:45:07 GMT 2020
Size of a homopolymer:	3
Skip duplicate alignments:	no
Number of windows:	400
BAM file:	ccs999KIR7_18_3.contigs_MN167520_sorted.bam

2. Summary

2.1. Globals

Reference size	193,395
Number of reads	24
Mapped reads	24 / 100%
Supplementary alignments	2 / 8.33%
Unmapped reads	0 / 0%
Mapped paired reads	0 / 0%
Read min/max/mean length	3,465 / 72,291 / 23,630.71
Duplicated reads (estimated)	4 / 16.67%
Duplication rate	10%
Clipped reads	15 / 62.5%

2.2. ACGT Content

Number/percentage of A's	116,521 / 26.9%
Number/percentage of C's	97,167 / 22.43%
Number/percentage of T's	110,860 / 25.59%
Number/percentage of G's	108,692 / 25.09%
Number/percentage of N's	0 / 0%
GC Percentage	47.52%

2.3. Coverage

Mean	2.2403
Standard Deviation	1.1242

2.4. Mapping Quality

Mean Mapping Quality	54.3

2.5. Mismatches and indels

General error rate	0.01%
Mismatches	51
Insertions	7
Mapped reads with at least one insertion	29.17%
Deletions	27
Mapped reads with at least one deletion	50%
Homopolymer indels	94.12%

2.6. Chromosome stats

**Name**	**Length**	**Mapped bases**	**Mean coverage**	**Standard deviation**
MN167520	193395	433272	2.2403	1.1242

3. Results : Coverage across reference

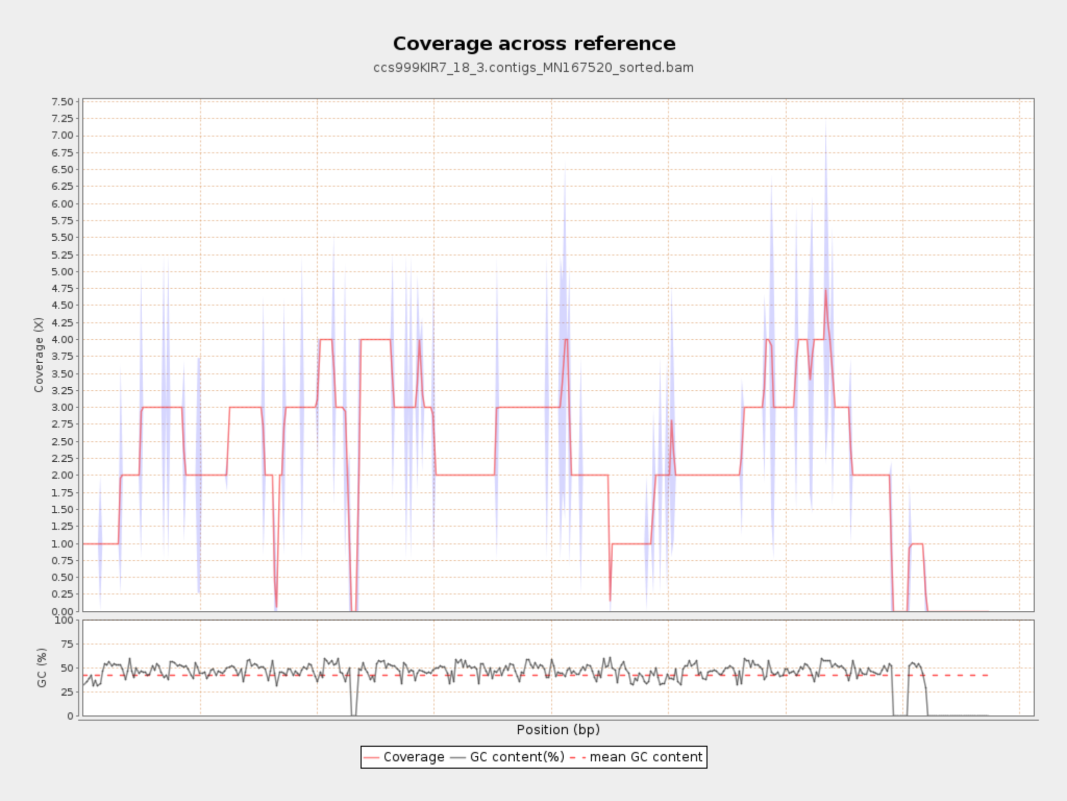

4. Results : Coverage Histogram

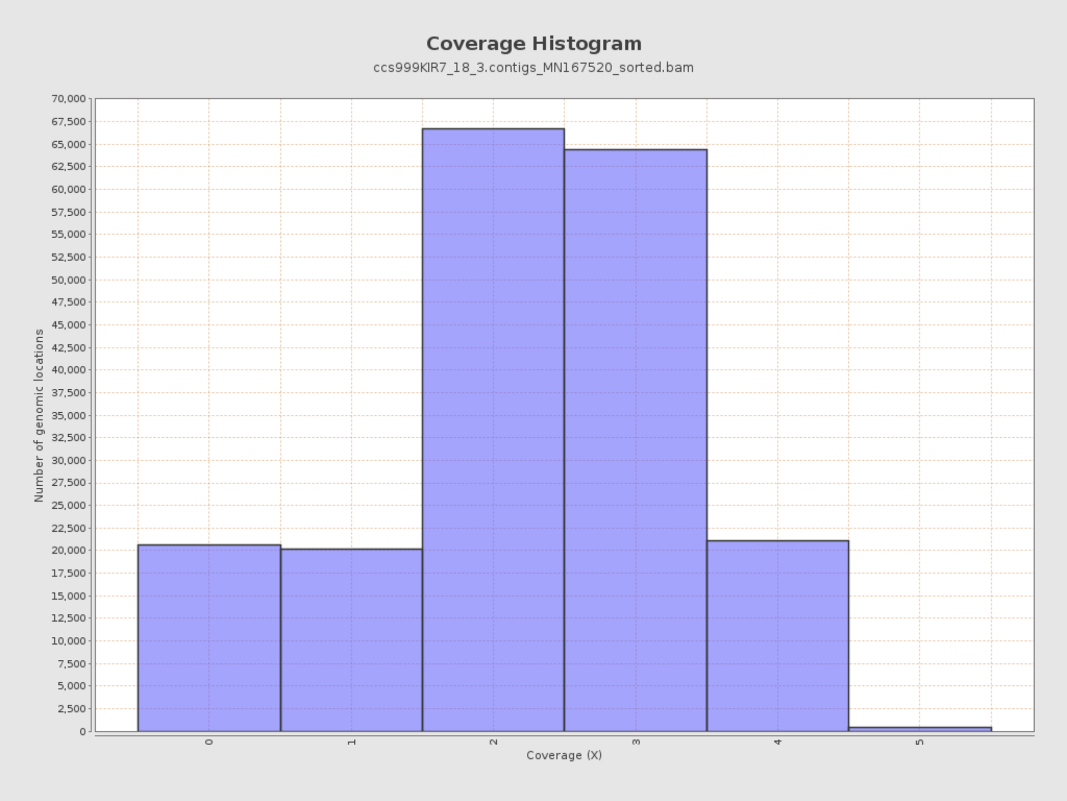

5. Results : Coverage Histogram (0-50X)

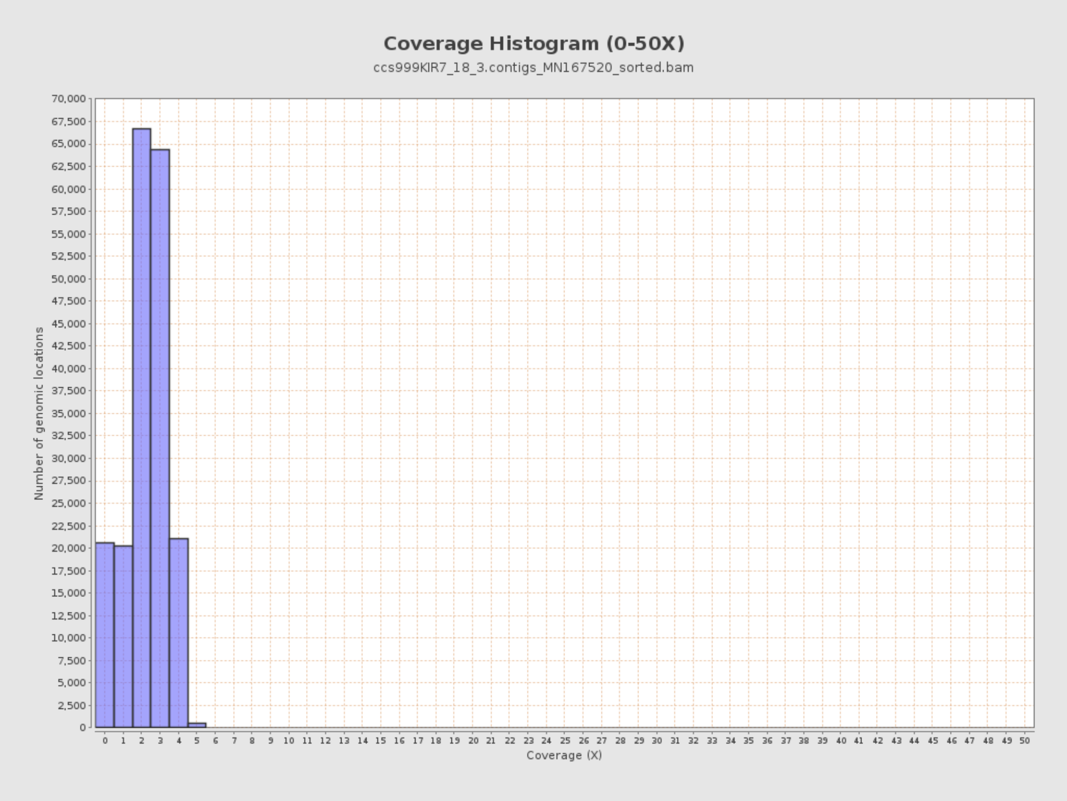

6. Results : Genome Fraction Coverage

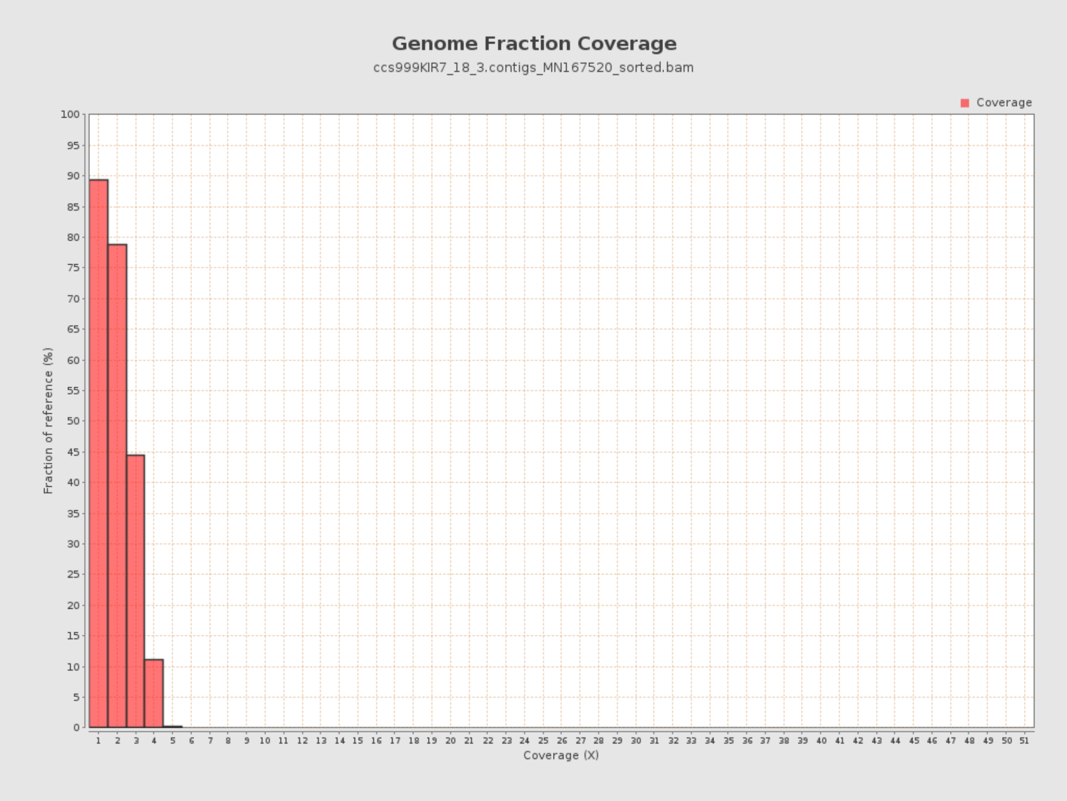

7. Results : Duplication Rate Histogram

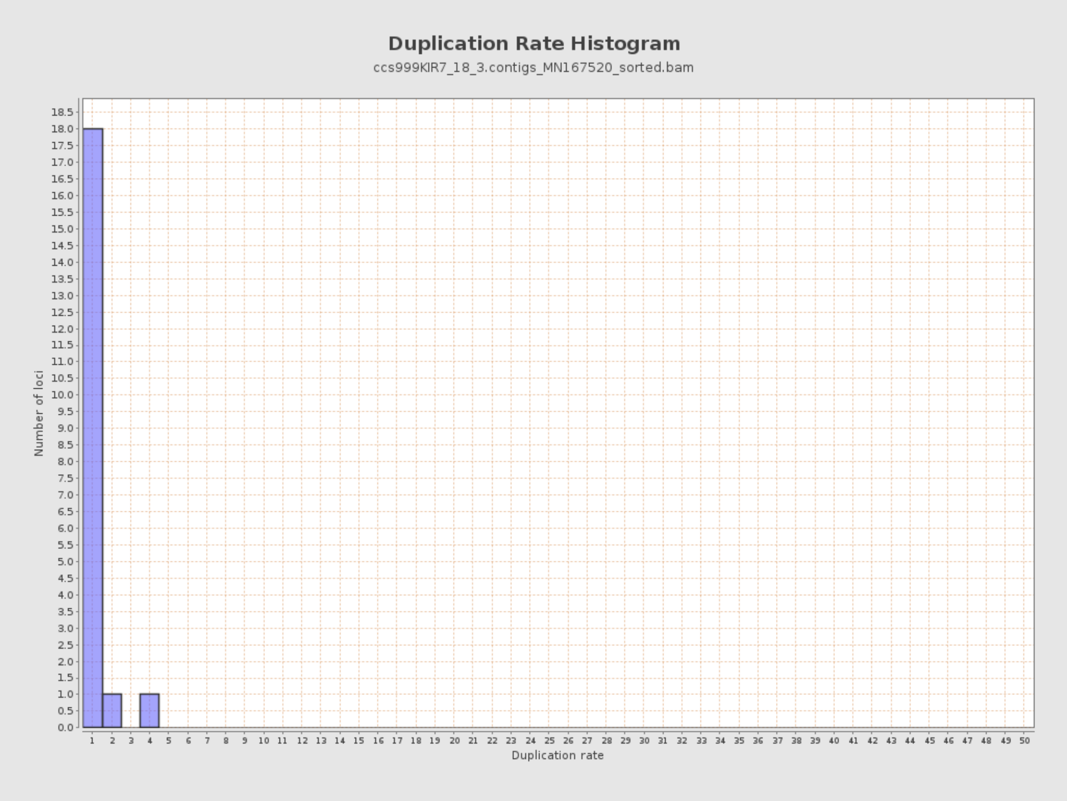

8. Results : Mapped Reads Nucleotide Content

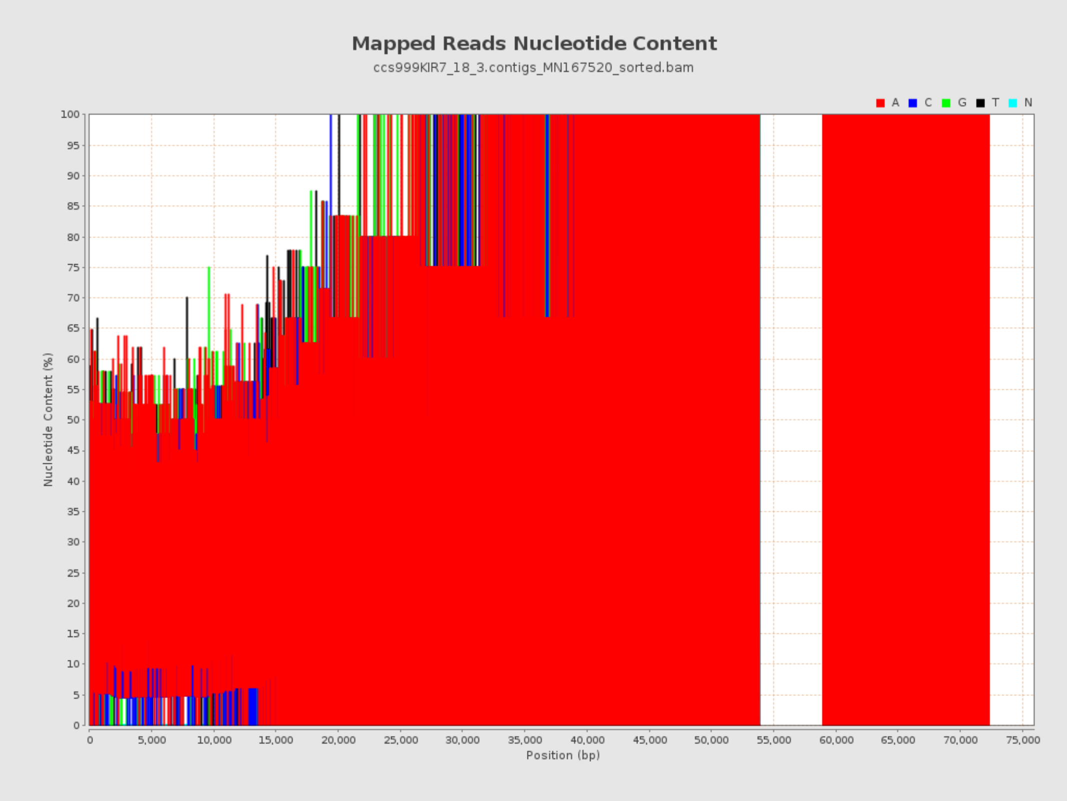

9. Results : Mapped Reads GC-content Distribution

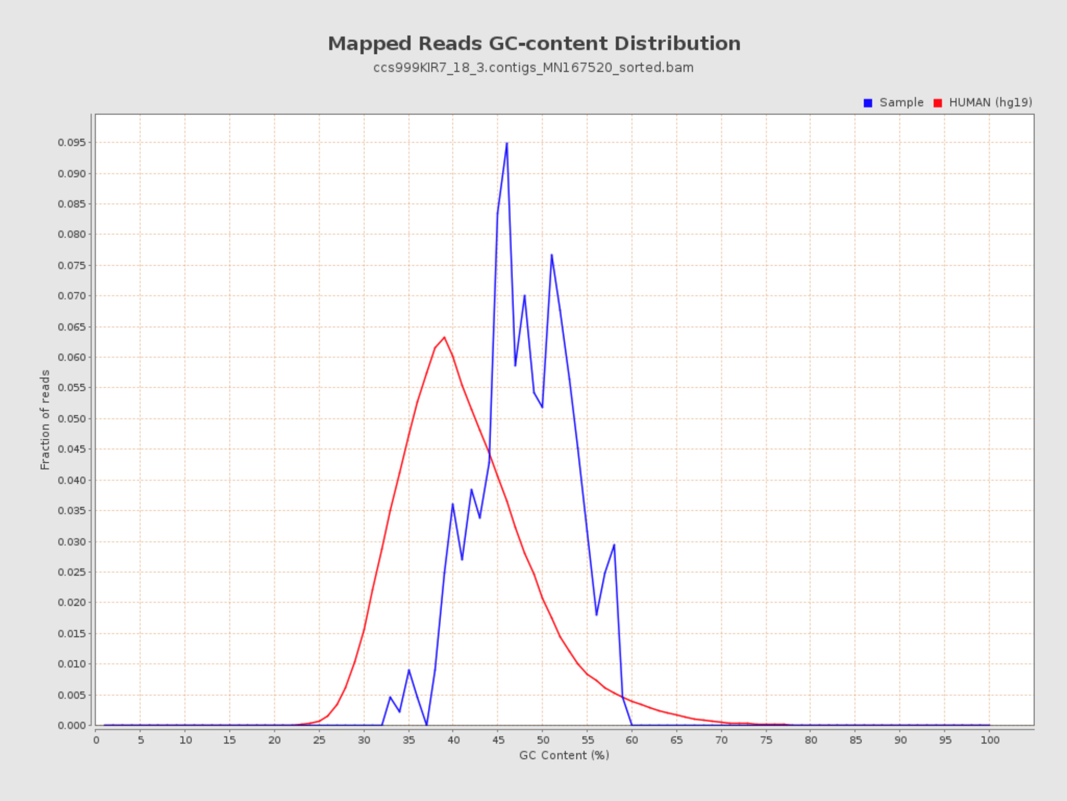

10. Results : Mapped Reads Clipping Profile

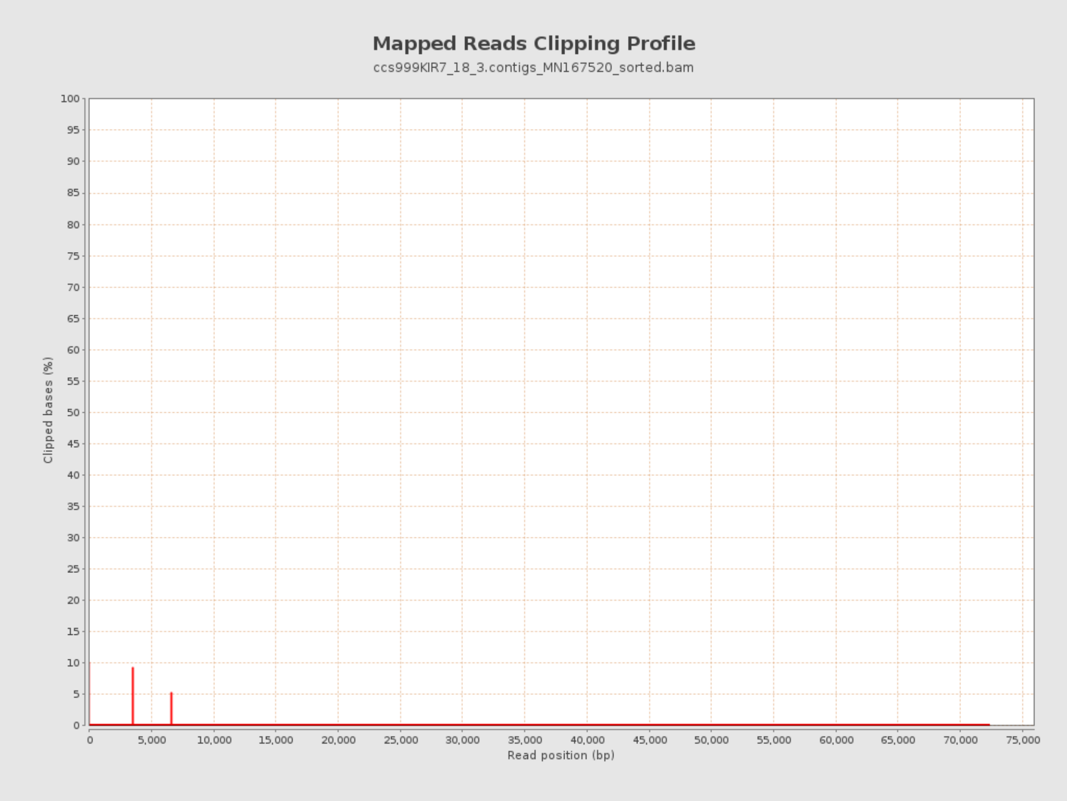

11. Results : Homopolymer Indels

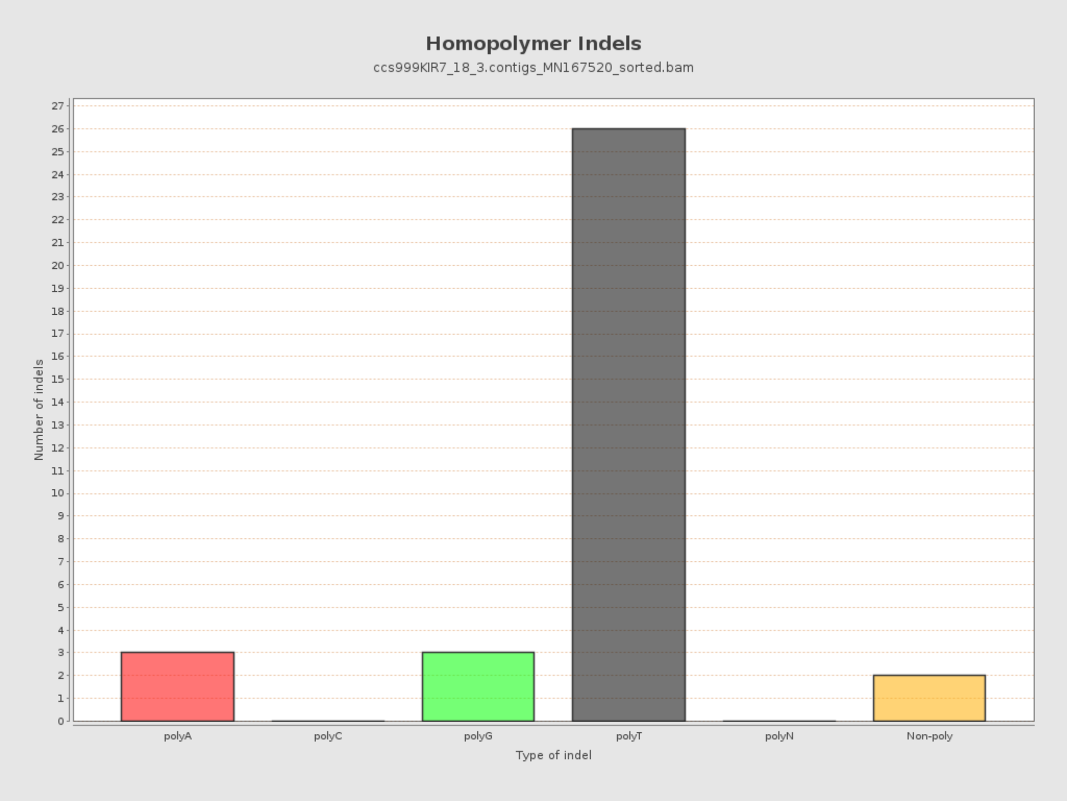

12. Results : Mapping Quality Across Reference

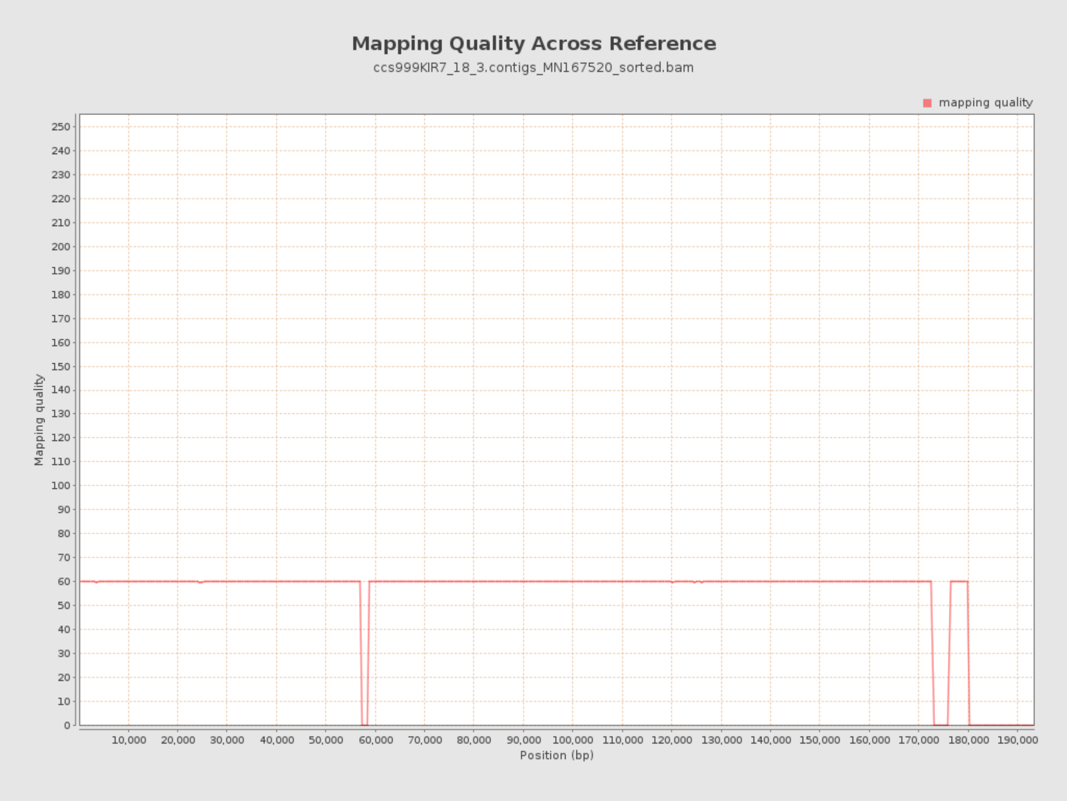

13. Results : Mapping Quality Histogram

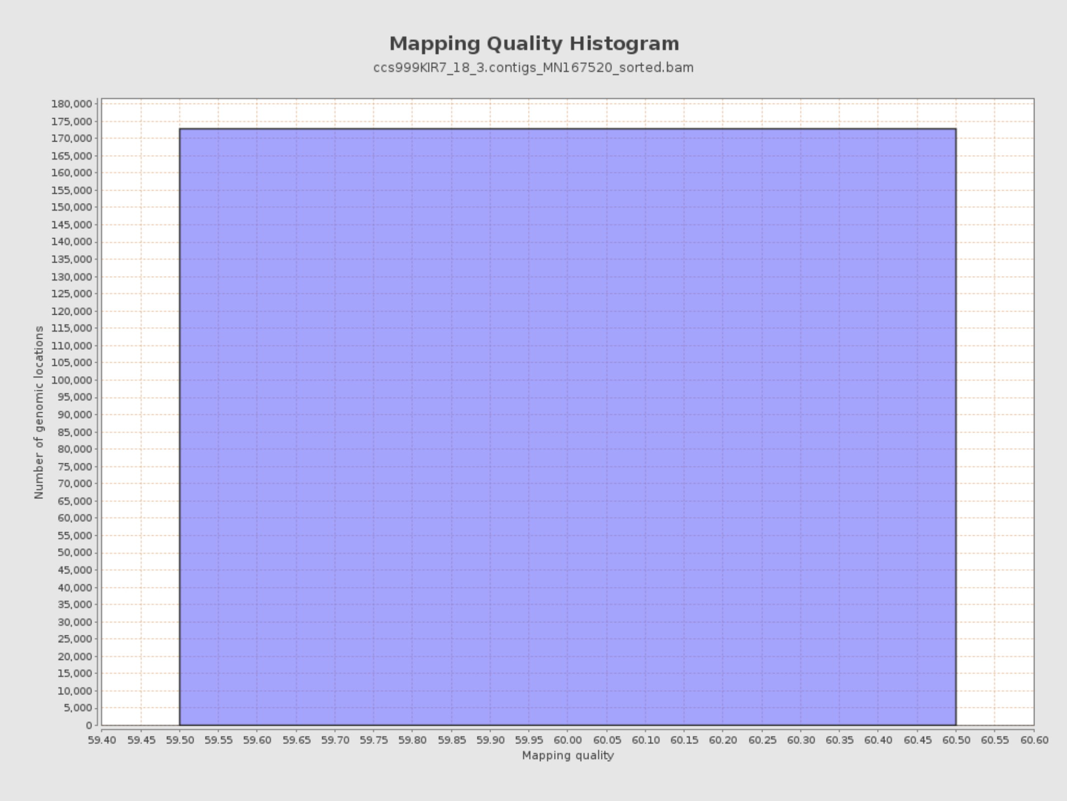

Supplement: Supplementary file 8 [file DataSheet_4.zip › SF1b/ccs999KIR7_18_3.contigs_MN167520_reports/ccs999KIR7_18_3.contigs_MN167520_qualimap.pdf]
