# Supplementary material for: Efficient Sequencing, Assembly, and Annotation of Human KIR Haplotypes
Source: Front Immunol. 2020 Oct 9;11:582927. doi: 10.3389/fimmu.2020.582927 (PMC7581912; doi:10.3389/fimmu.2020.582927)

Qualimap Analysis Results

BAM QC analysis

Generated by Qualimap v.2.2.1

2020/04/21 17:45:36

1. Input data & parameters

1.1. QualiMap command line

qualimap bamqc -bam ccs999KIR7_18_3.contigs_MN167521_sorted.bam -nw 400 -hm 3

1.2. Alignment

Command line:	bwa mem -k1800 -W9000 -r10 -A1 -B100 -O40 -E40 -L50 -t7 MN167521.fasta ccs999KIR7_18_3.contigs.fasta.gz
Draw chromosome limits:	no
Analyze overlapping paired-end reads:	no
Program:	bwa (0.7.17-r1188)
Analysis date:	Tue Apr 21 17:45:35 GMT 2020
Size of a homopolymer:	3
Skip duplicate alignments:	no
Number of windows:	400
BAM file:	ccs999KIR7_18_3.contigs_MN167521_sorted.bam

2. Summary

2.1. Globals

Reference size	201,909
Number of reads	20
Mapped reads	20 / 100%
Unmapped reads	0 / 0%
Mapped paired reads	0 / 0%
Read min/max/mean length	10,568 / 72,291 / 26,892.6
Duplicated reads (estimated)	0 / 0%
Duplication rate	0%
Clipped reads	10 / 50%

2.2. ACGT Content

Number/percentage of A's	121,770 / 26.74%
Number/percentage of C's	102,290 / 22.46%
Number/percentage of T's	117,300 / 25.76%
Number/percentage of G's	113,997 / 25.03%
Number/percentage of N's	0 / 0%
GC Percentage	47.5%

2.3. Coverage

Mean	2.2554
Standard Deviation	1.3592

2.4. Mapping Quality

Mean Mapping Quality	51.45

2.5. Mismatches and indels

General error rate	0.02%
Mismatches	63
Insertions	17
Mapped reads with at least one insertion	45%
Deletions	23
Mapped reads with at least one deletion	55%
Homopolymer indels	77.5%

2.6. Chromosome stats

**Name**	**Length**	**Mapped bases**	**Mean coverage**	**Standard deviation**
MN167521	201909	455382	2.2554	1.3592

3. Results : Coverage across reference

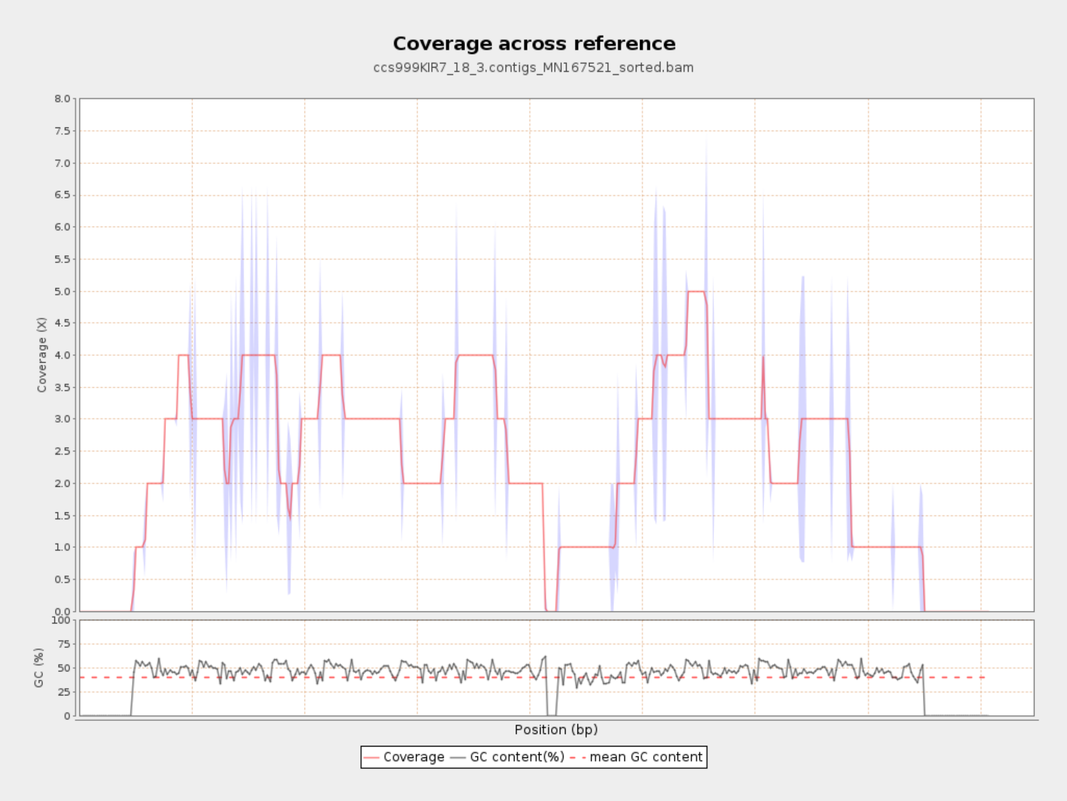

4. Results : Coverage Histogram

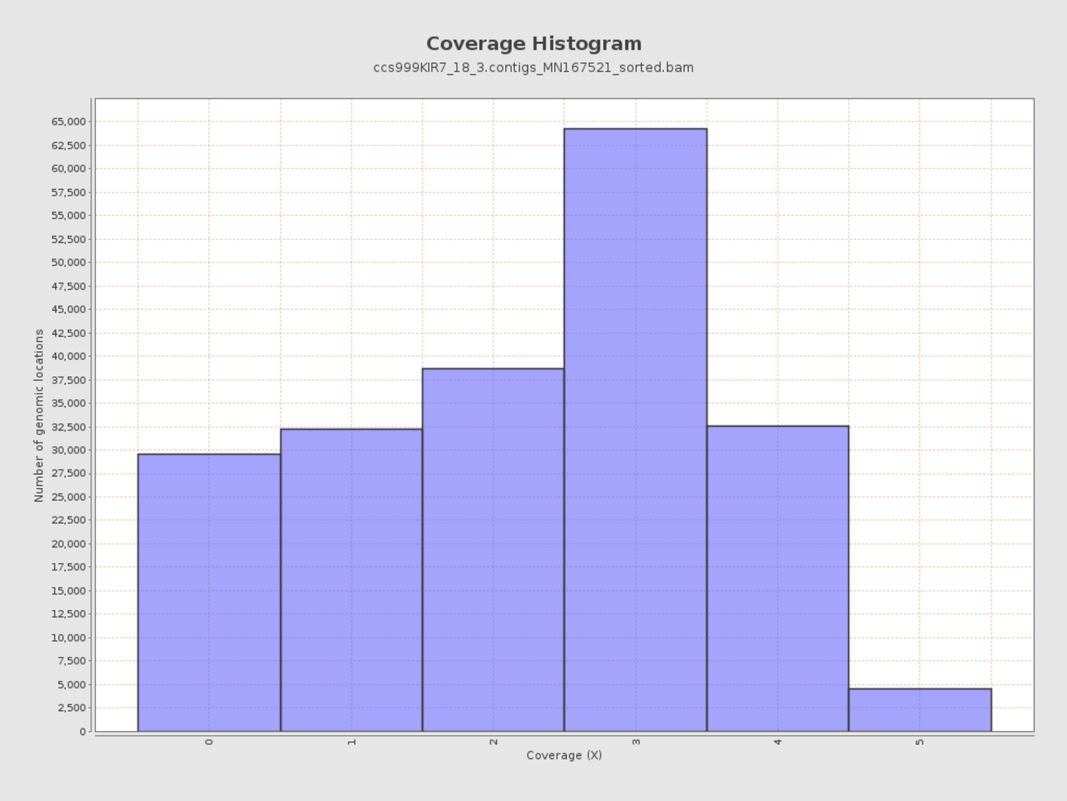

5. Results : Coverage Histogram (0-50X)

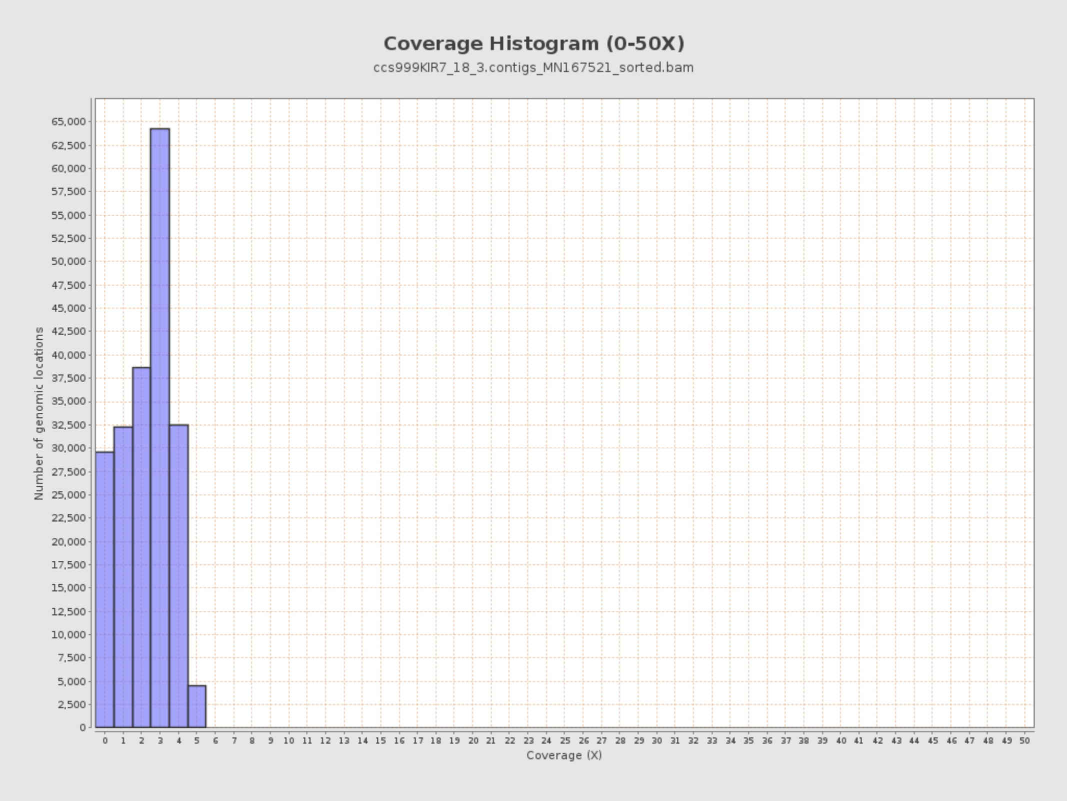

6. Results : Genome Fraction Coverage

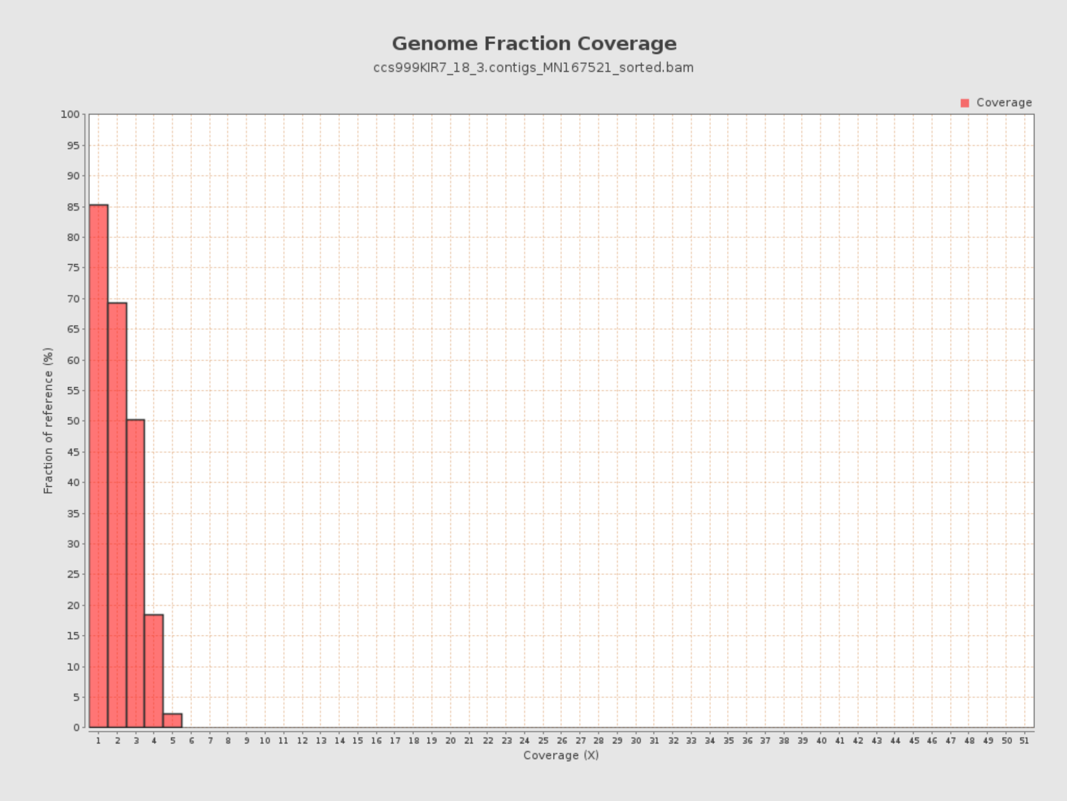

7. Results : Duplication Rate Histogram

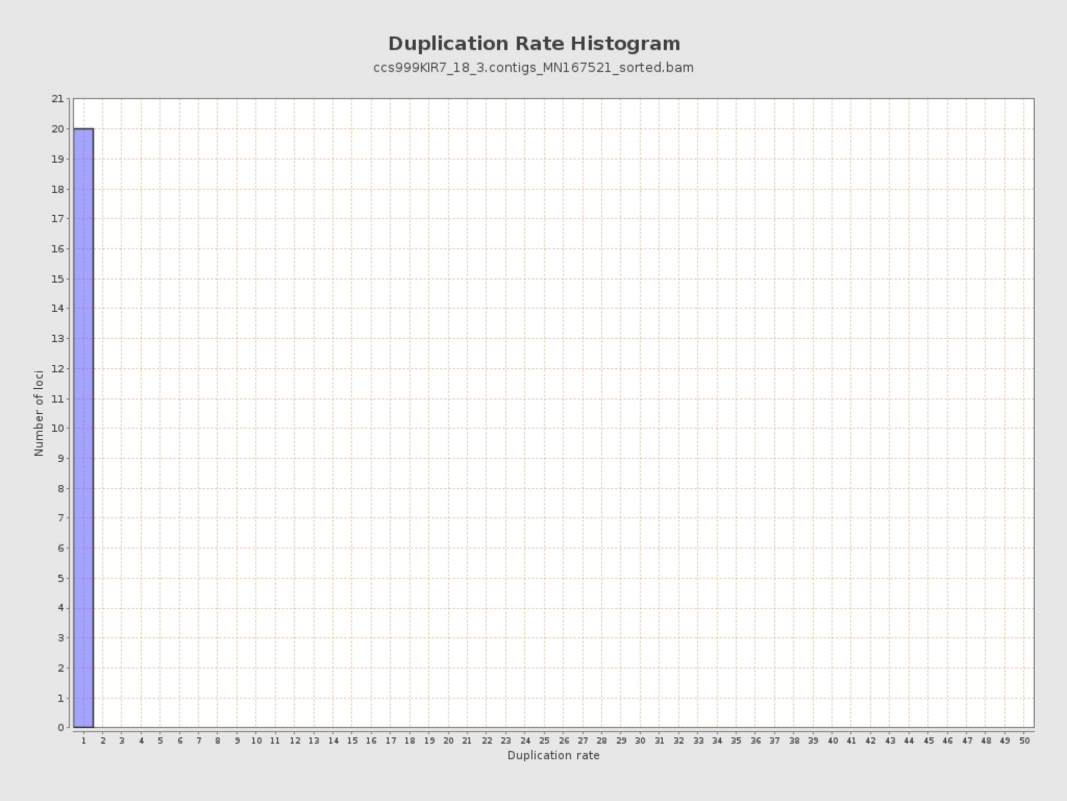

8. Results : Mapped Reads Nucleotide Content

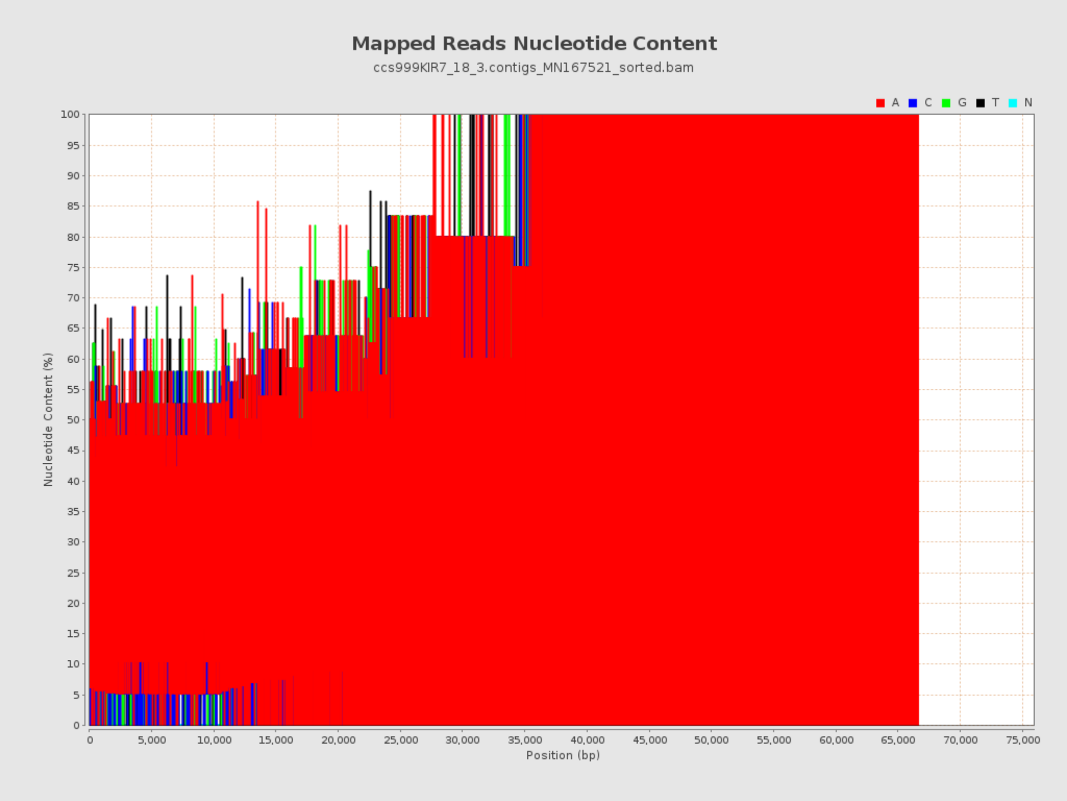

9. Results : Mapped Reads GC-content Distribution

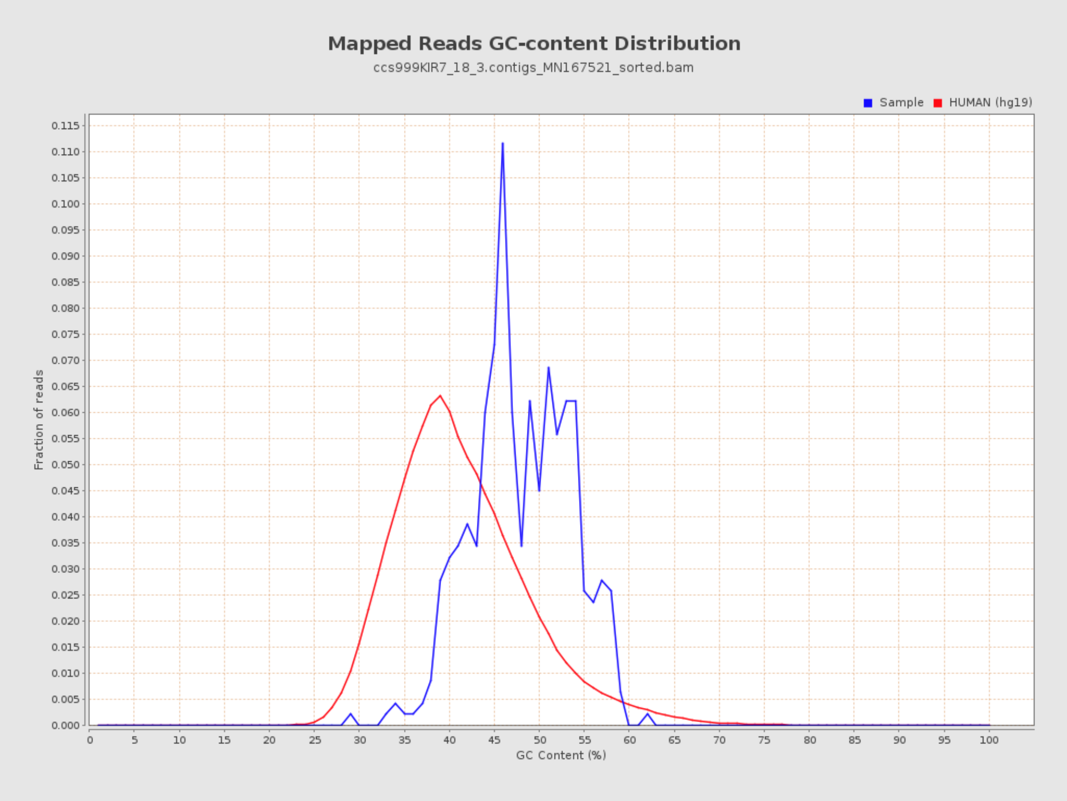

10. Results : Mapped Reads Clipping Profile

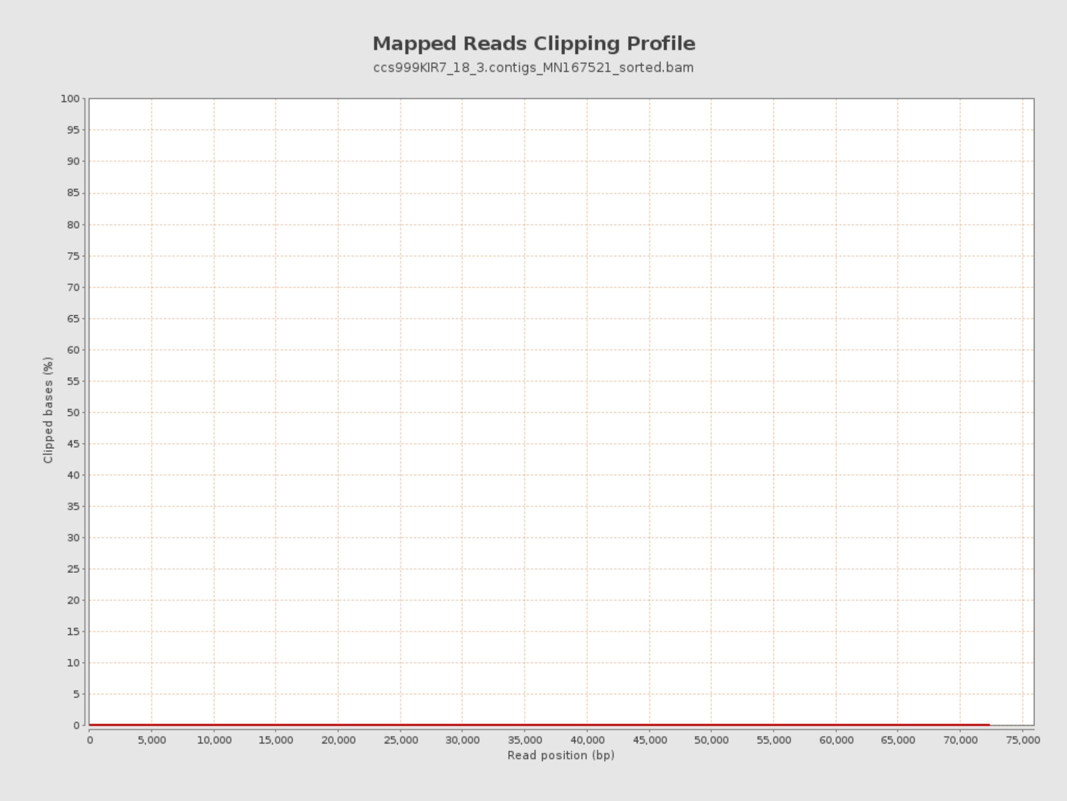

11. Results : Homopolymer Indels

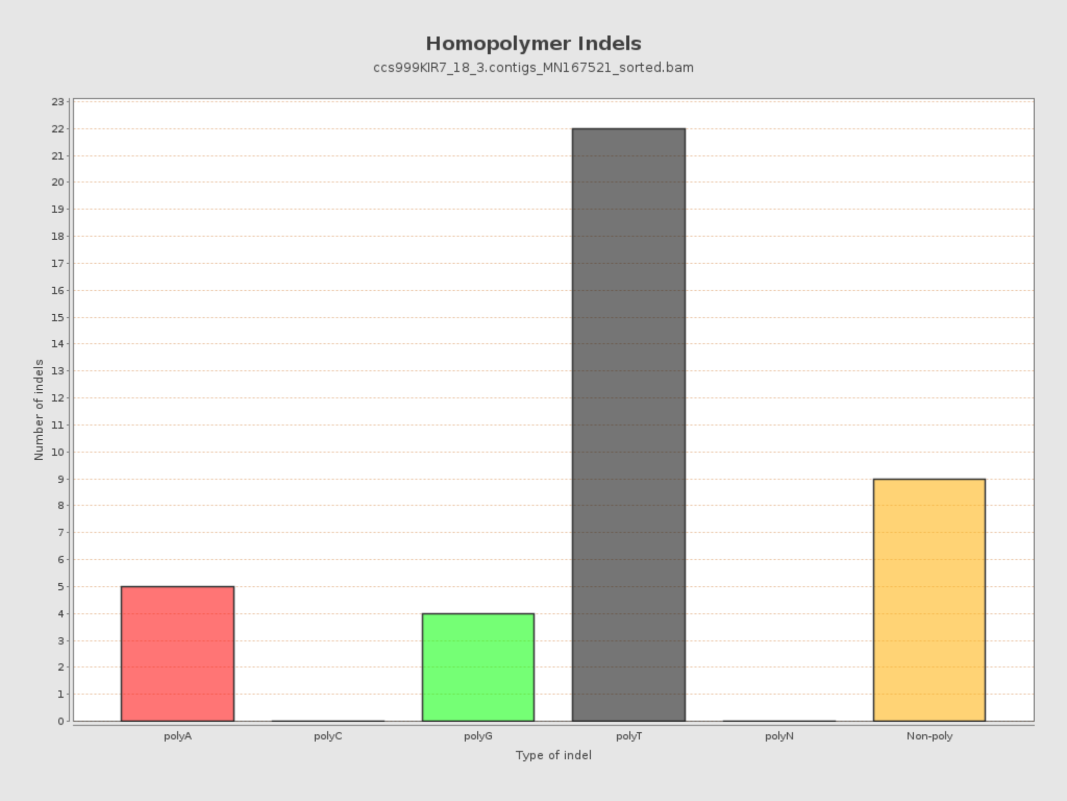

12. Results : Mapping Quality Across Reference

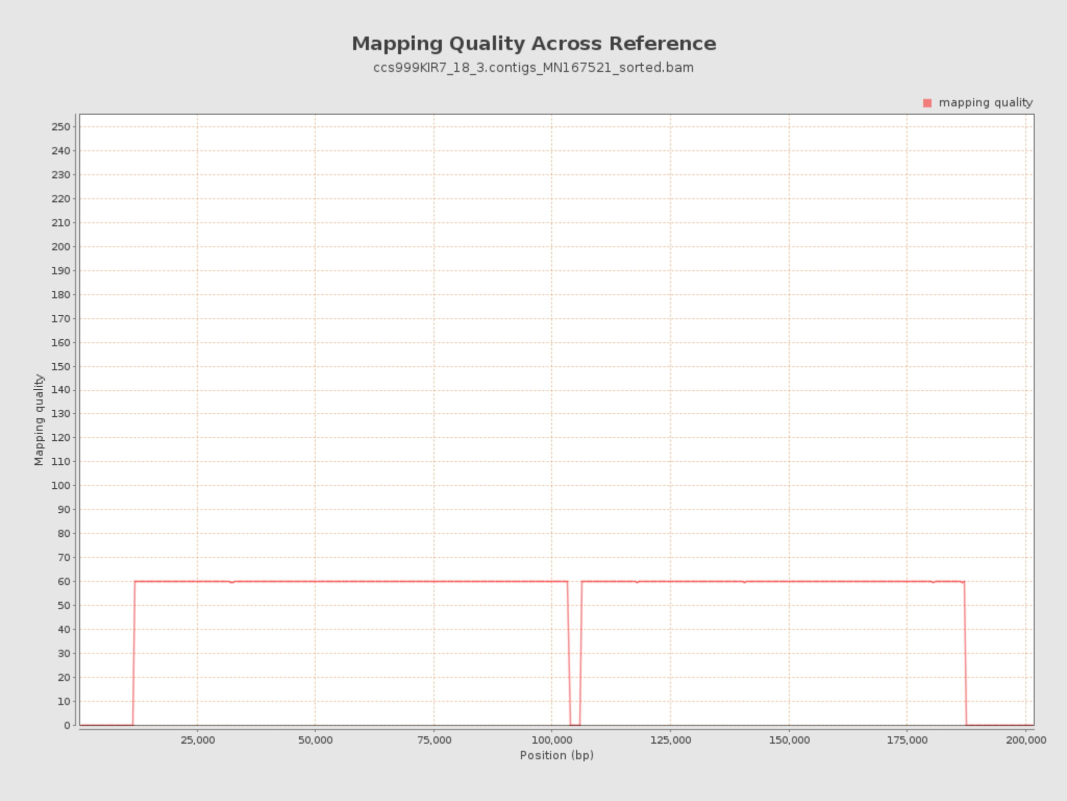

13. Results : Mapping Quality Histogram

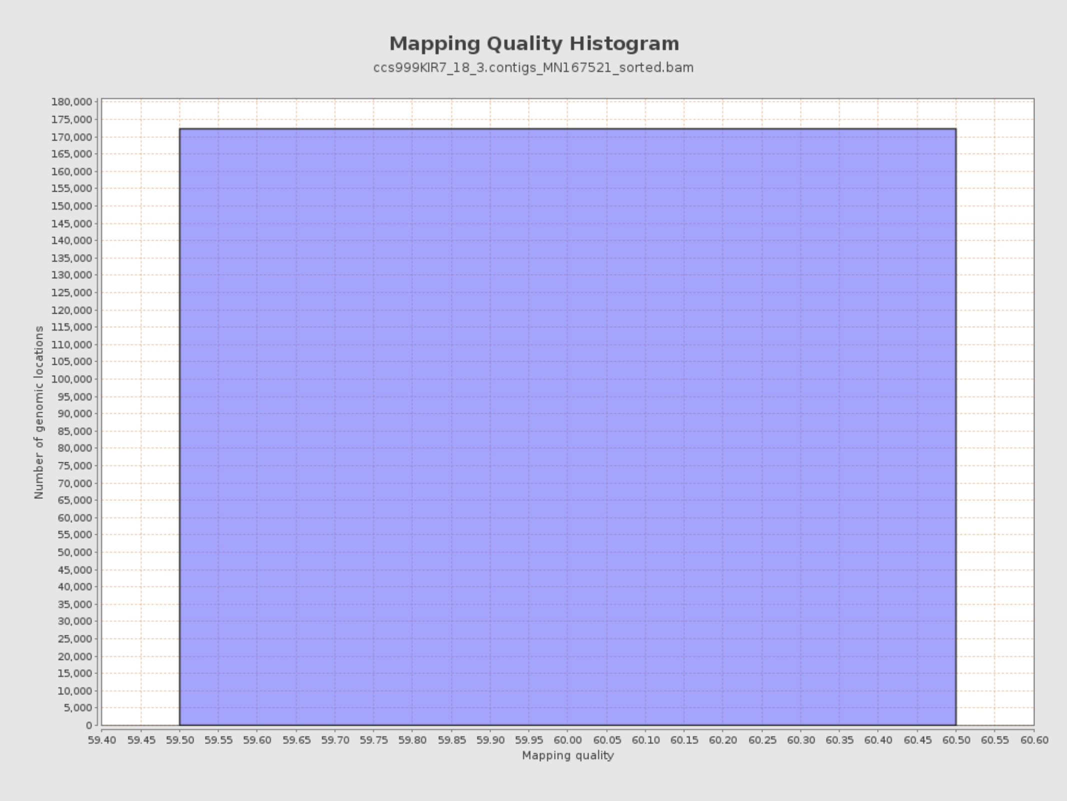

Supplement: Supplementary file 8 [file DataSheet_4.zip › SF1b/ccs999KIR7_18_3.contigs_MN167521_reports/ccs999KIR7_18_3.contigs_MN167521_qualimap.pdf]
