# Supplementary material for: Efficient Sequencing, Assembly, and Annotation of Human KIR Haplotypes
Source: Front Immunol. 2020 Oct 9;11:582927. doi: 10.3389/fimmu.2020.582927 (PMC7581912; doi:10.3389/fimmu.2020.582927)
Supplement: Supplementary file 8 [file DataSheet_4.zip › SF1b/ccs999KIR7_18_4.contigs_MN167522_reports/quast/icarus_viewers/alignment_viewer.html]

|  |  |  |  |  |
| --- | --- | --- | --- | --- |
| Main menu  Icarus **QUAST Contig Browser** | |  |  | | --- | --- | | Move << < > >>  zoom +5x +2x –2x –5x | start   end |   Show misassemblies: relocations (0) translocations (0) inversions (0) local (0) | Search contig or gene: |

**Contig alignment viewer**. Contigs aligned to MN167522

Hide
Show annotation
Hide
Show read coverage and GC distribution
Show physical
Show physical
Hide GC %
Hide GC %

+
-
Reset

+
-
Reset
