# Supplementary material for: Efficient Sequencing, Assembly, and Annotation of Human KIR Haplotypes
Source: Front Immunol. 2020 Oct 9;11:582927. doi: 10.3389/fimmu.2020.582927 (PMC7581912; doi:10.3389/fimmu.2020.582927)

Qualimap Analysis Results

BAM QC analysis

Generated by Qualimap v.2.2.1

2020/04/21 17:46:34

1. Input data & parameters

1.1. QualiMap command line

qualimap bamqc -bam ccs999KIR7_18_4.contigs_MN167523_sorted.bam -nw 400 -hm 3

1.2. Alignment

Command line:	bwa mem -k1800 -W9000 -r10 -A1 -B100 -O40 -E40 -L50 -t7 MN167523.fasta ccs999KIR7_18_4.contigs.fasta.gz
Draw chromosome limits:	no
Analyze overlapping paired-end reads:	no
Program:	bwa (0.7.17-r1188)
Analysis date:	Tue Apr 21 17:46:33 GMT 2020
Size of a homopolymer:	3
Skip duplicate alignments:	no
Number of windows:	400
BAM file:	ccs999KIR7_18_4.contigs_MN167523_sorted.bam

2. Summary

2.1. Globals

Reference size	137,804
Number of reads	25
Mapped reads	25 / 100%
Supplementary alignments	1 / 4%
Unmapped reads	0 / 0%
Mapped paired reads	0 / 0%
Read min/max/mean length	9,079 / 107,229 / 21,837.8
Duplicated reads (estimated)	0 / 0%
Duplication rate	0%
Clipped reads	11 / 44%

2.2. ACGT Content

Number/percentage of A's	122,800 / 27.11%
Number/percentage of C's	102,402 / 22.6%
Number/percentage of T's	116,000 / 25.61%
Number/percentage of G's	111,810 / 24.68%
Number/percentage of N's	0 / 0%
GC Percentage	47.29%

2.3. Coverage

Mean	3.2876
Standard Deviation	1.7927

2.4. Mapping Quality

Mean Mapping Quality	59.99

2.5. Mismatches and indels

General error rate	0.02%
Mismatches	73
Insertions	11
Mapped reads with at least one insertion	20%
Deletions	25
Mapped reads with at least one deletion	68%
Homopolymer indels	80.56%

2.6. Chromosome stats

**Name**	**Length**	**Mapped bases**	**Mean coverage**	**Standard deviation**
MN167523	137804	453046	3.2876	1.7927

3. Results : Coverage across reference

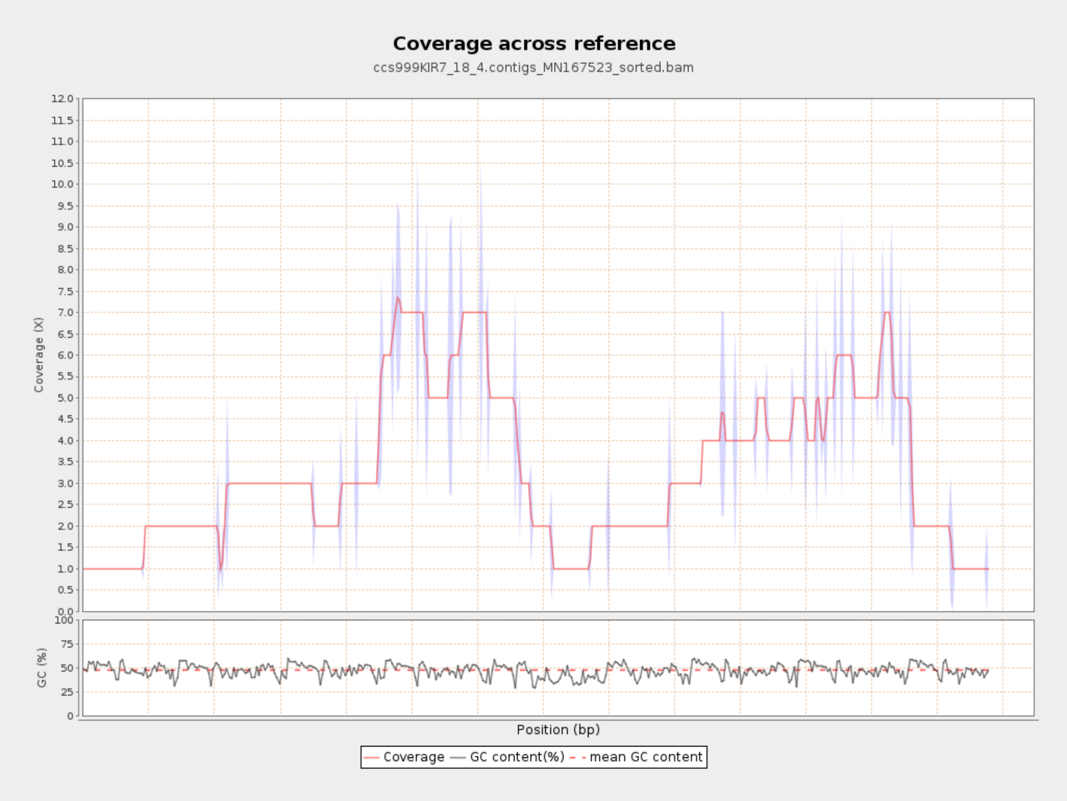

4. Results : Coverage Histogram

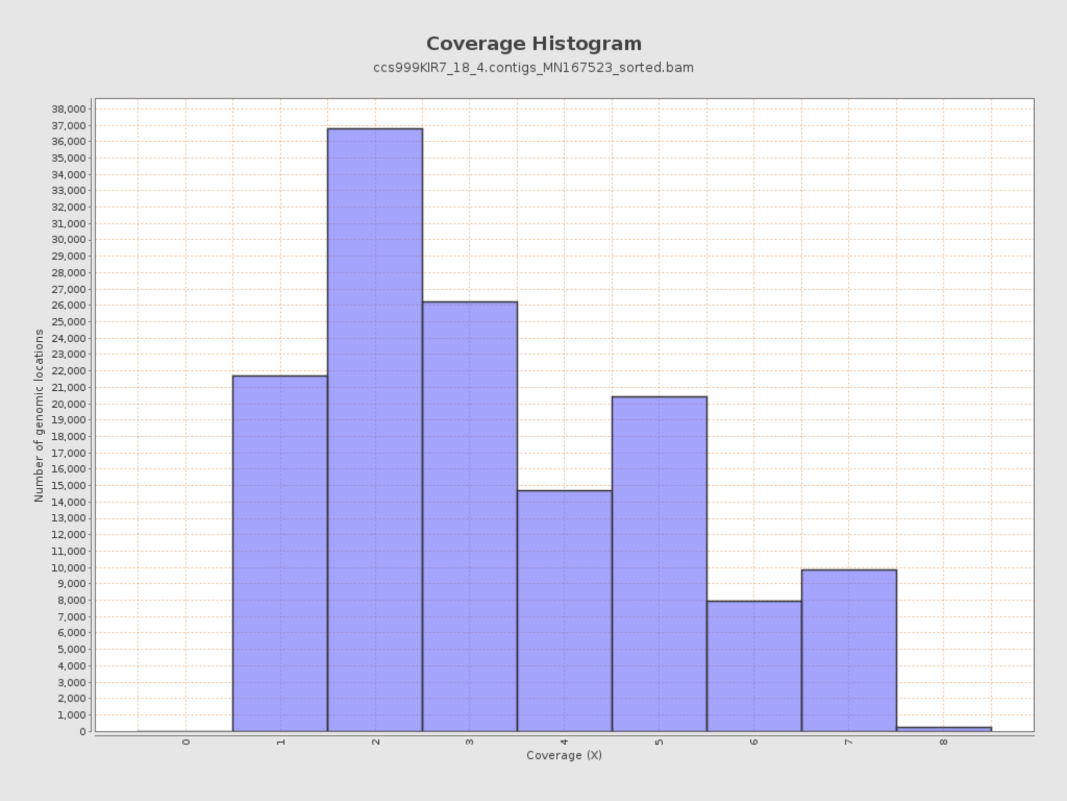

5. Results : Coverage Histogram (0-50X)

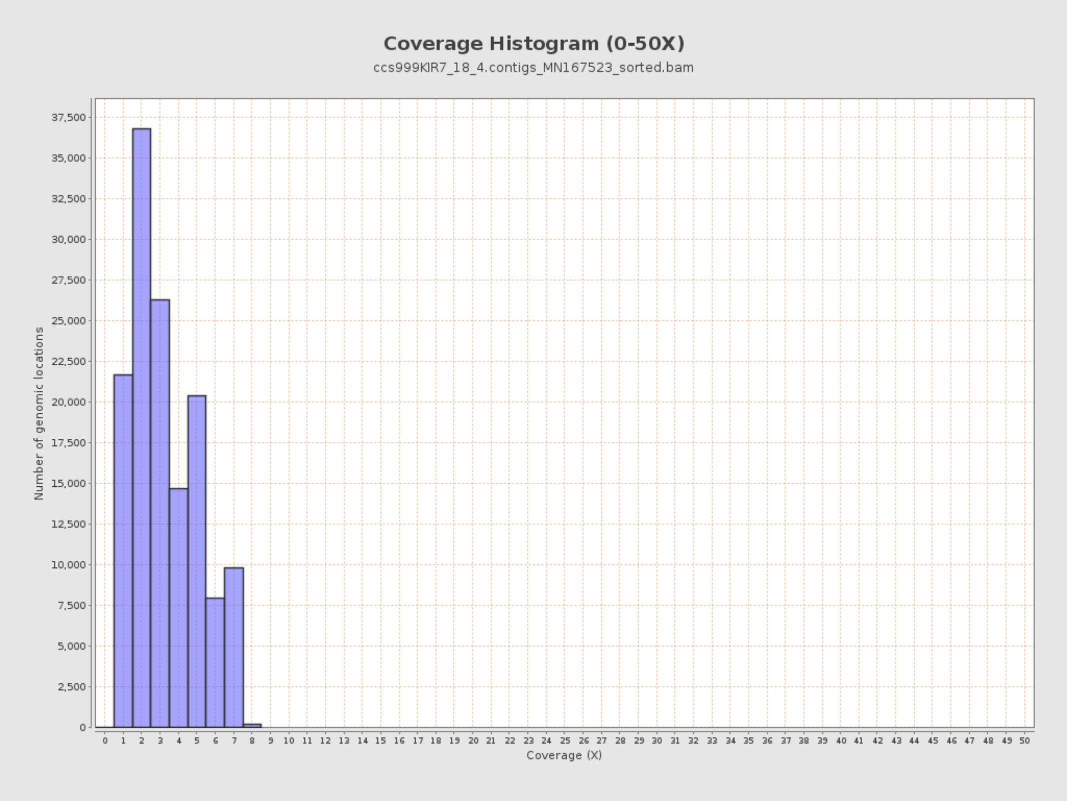

6. Results : Genome Fraction Coverage

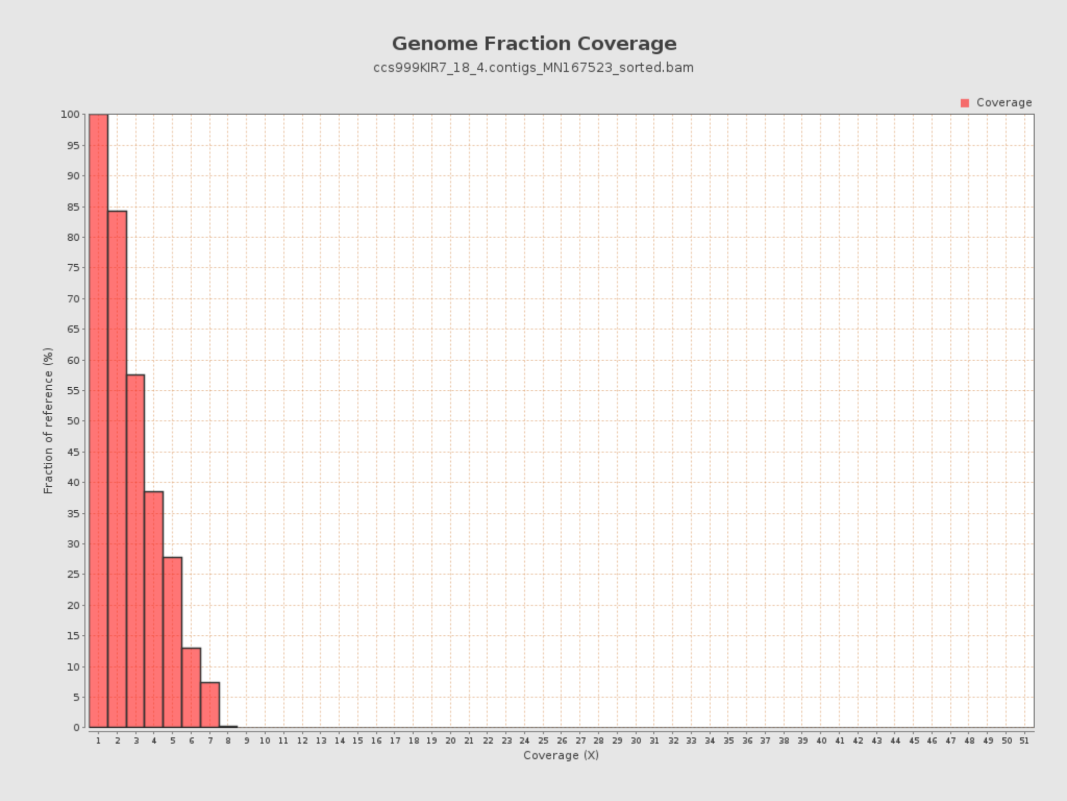

7. Results : Duplication Rate Histogram

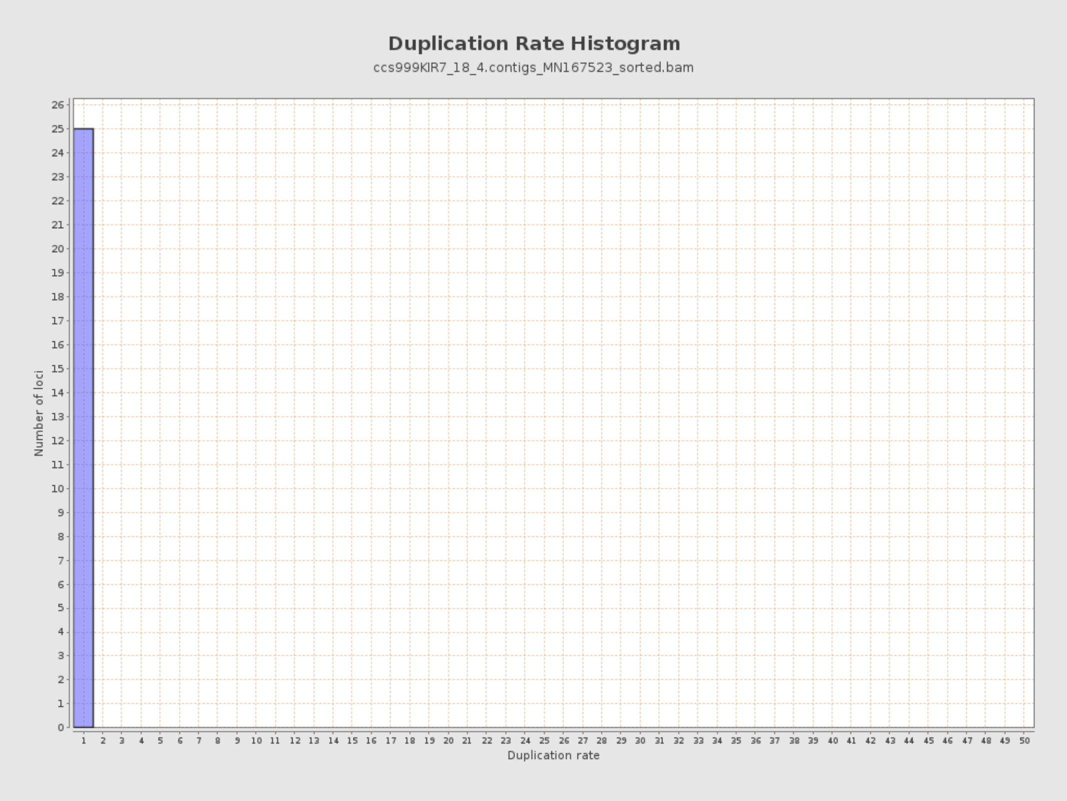

8. Results : Mapped Reads Nucleotide Content

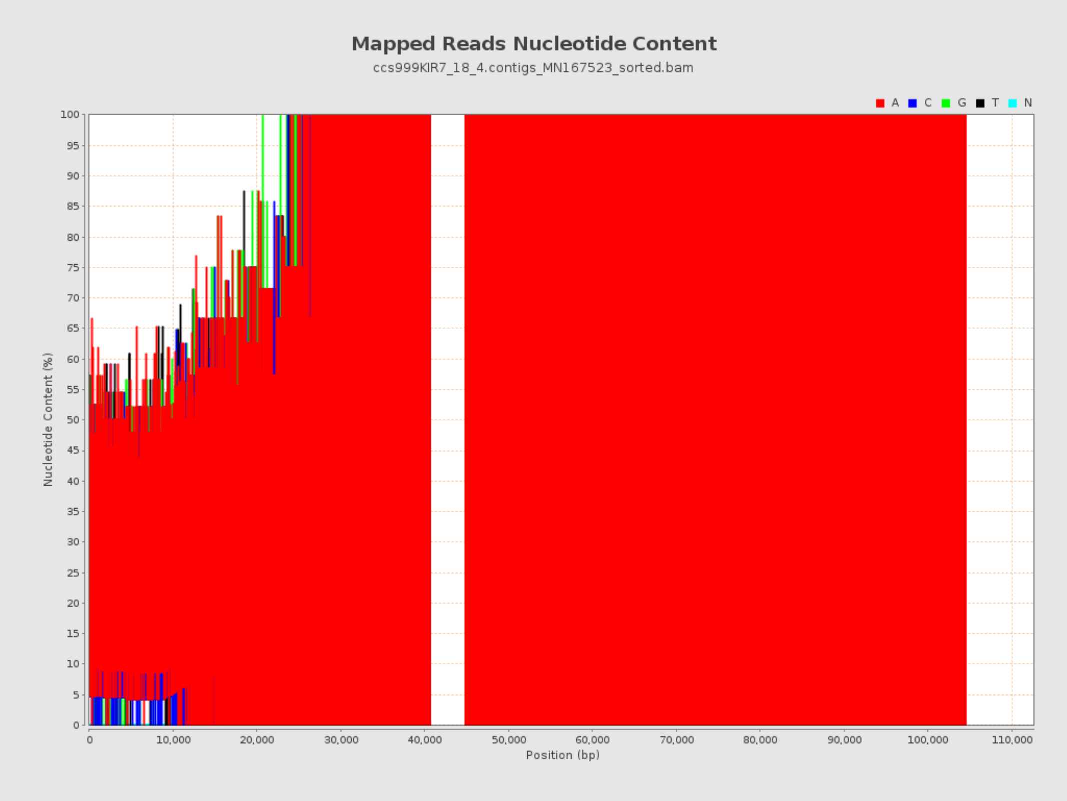

9. Results : Mapped Reads GC-content Distribution

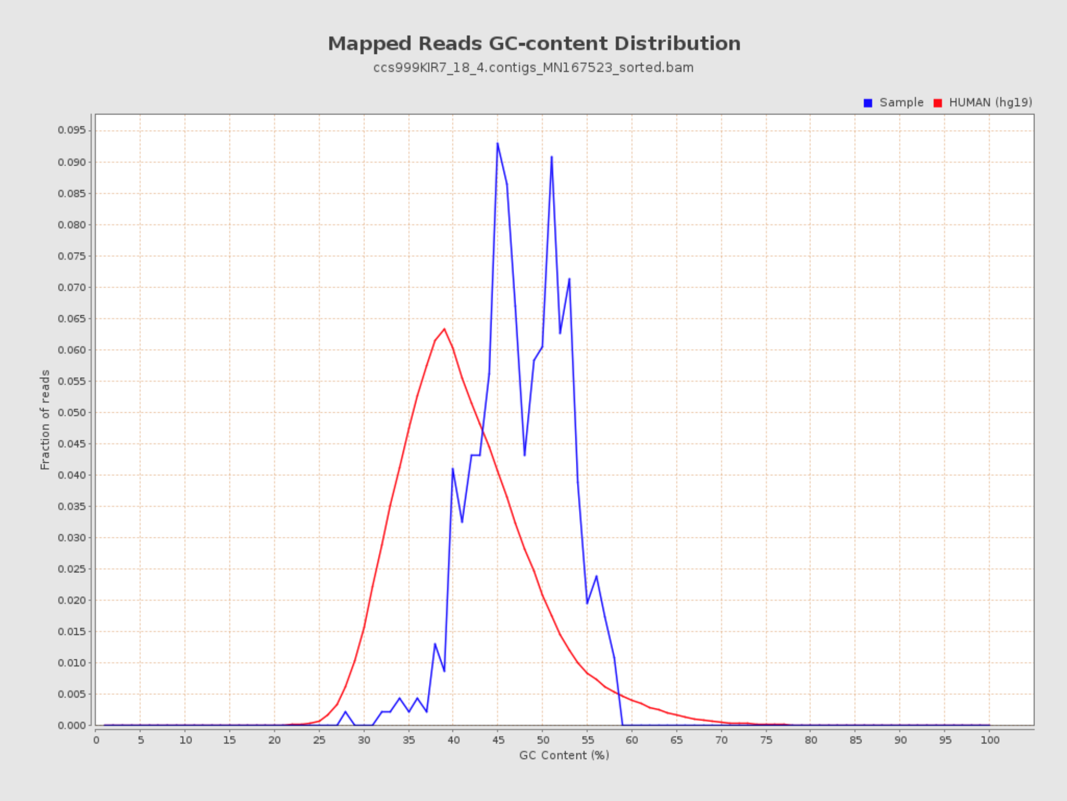

10. Results : Mapped Reads Clipping Profile

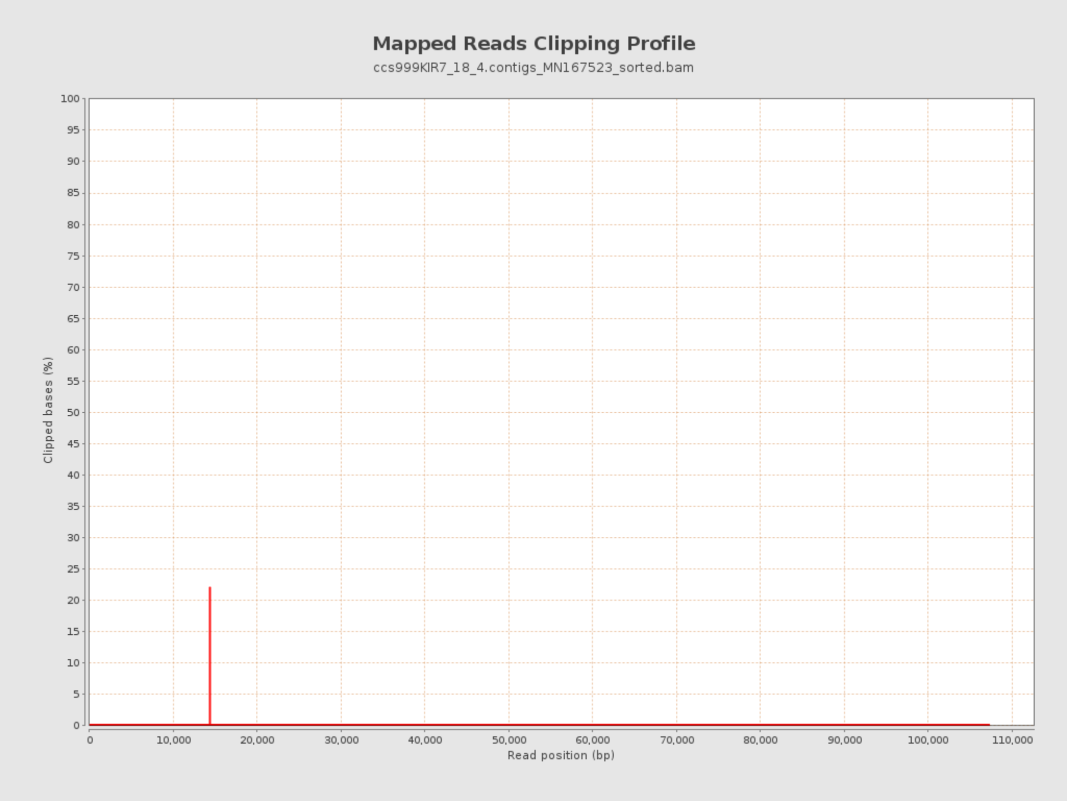

11. Results : Homopolymer Indels

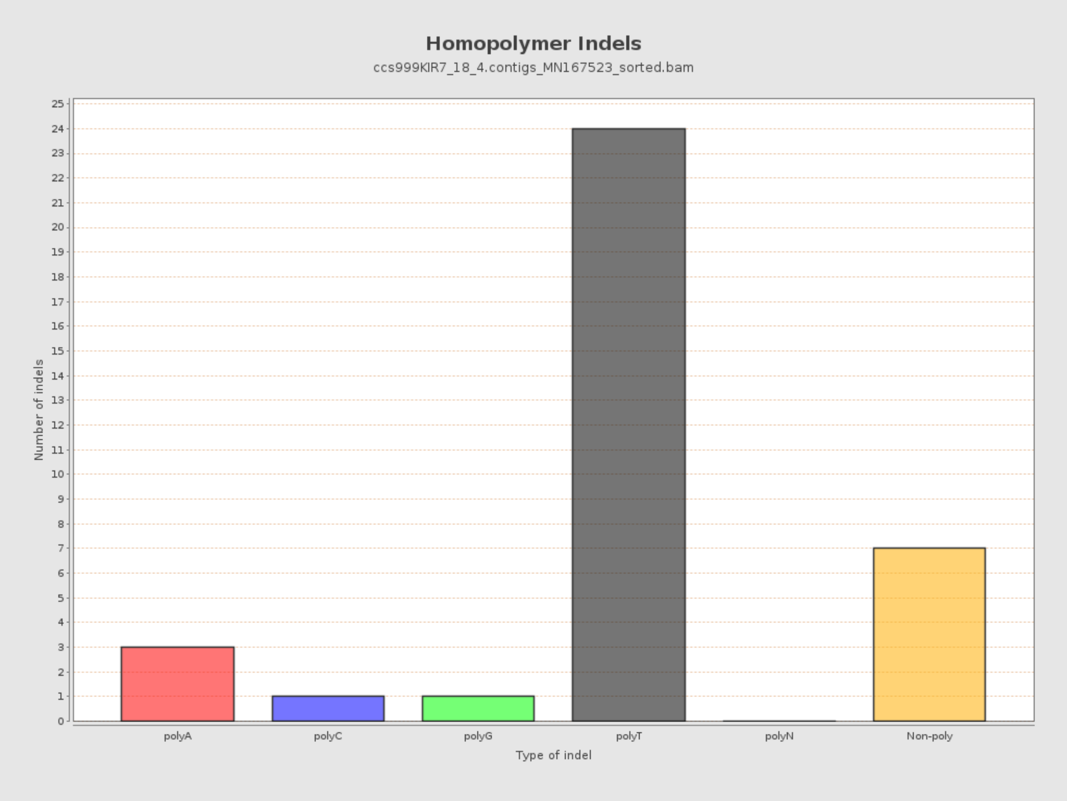

12. Results : Mapping Quality Across Reference

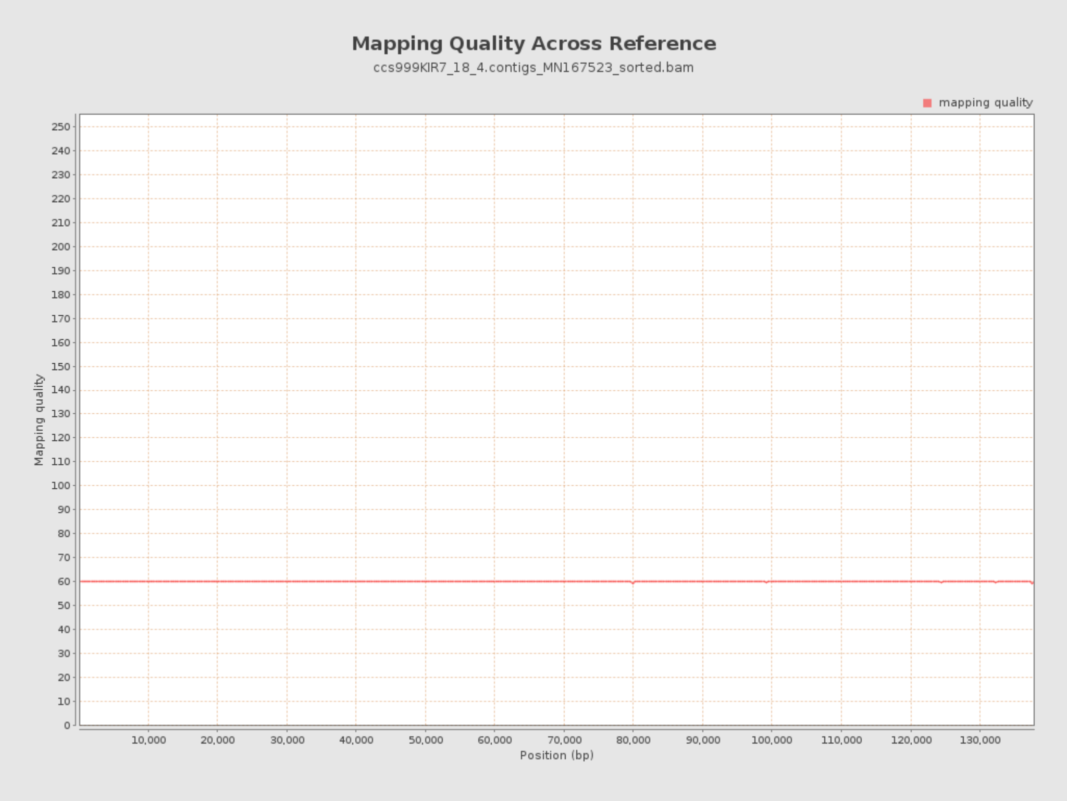

13. Results : Mapping Quality Histogram

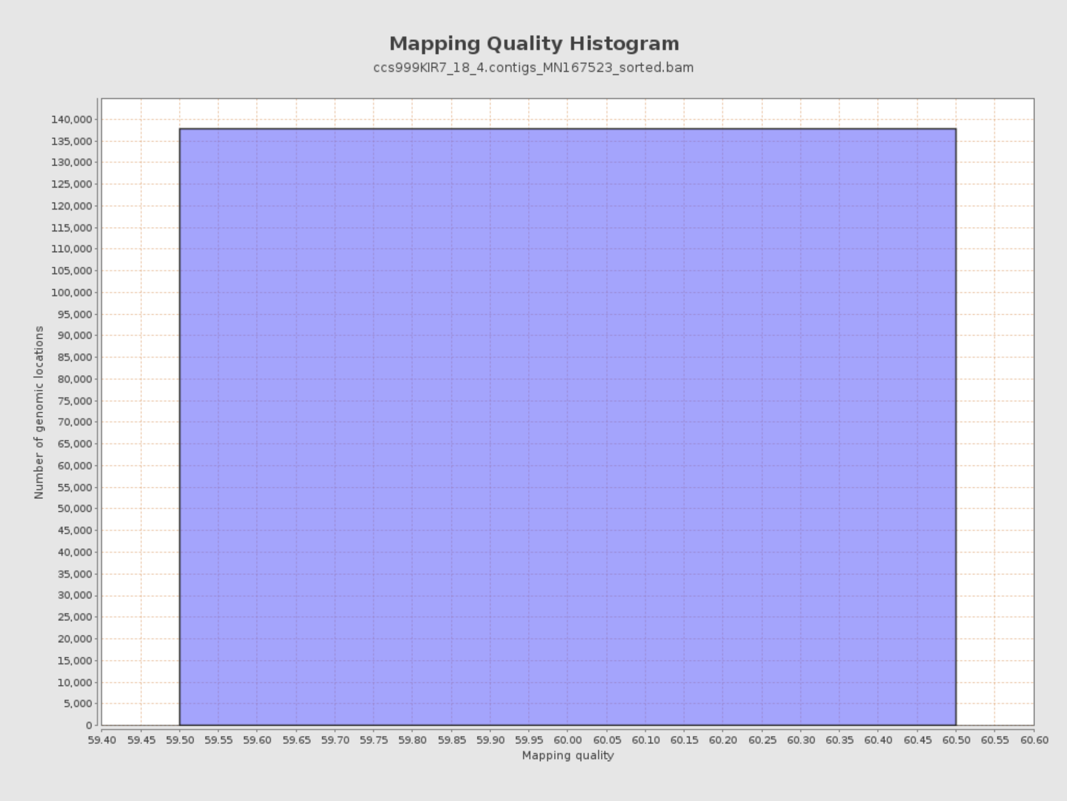

Supplement: Supplementary file 8 [file DataSheet_4.zip › SF1b/ccs999KIR7_18_4.contigs_MN167523_reports/ccs999KIR7_18_4.contigs_MN167523_qualimap.pdf]
