# Supplementary figures and images for: Efficient Sequencing, Assembly, and Annotation of Human KIR Haplotypes (part 2 of 2)
Source: Front Immunol. 2020 Oct 9;11:582927. doi: 10.3389/fimmu.2020.582927 (PMC7581912; doi:10.3389/fimmu.2020.582927)

Yield by length

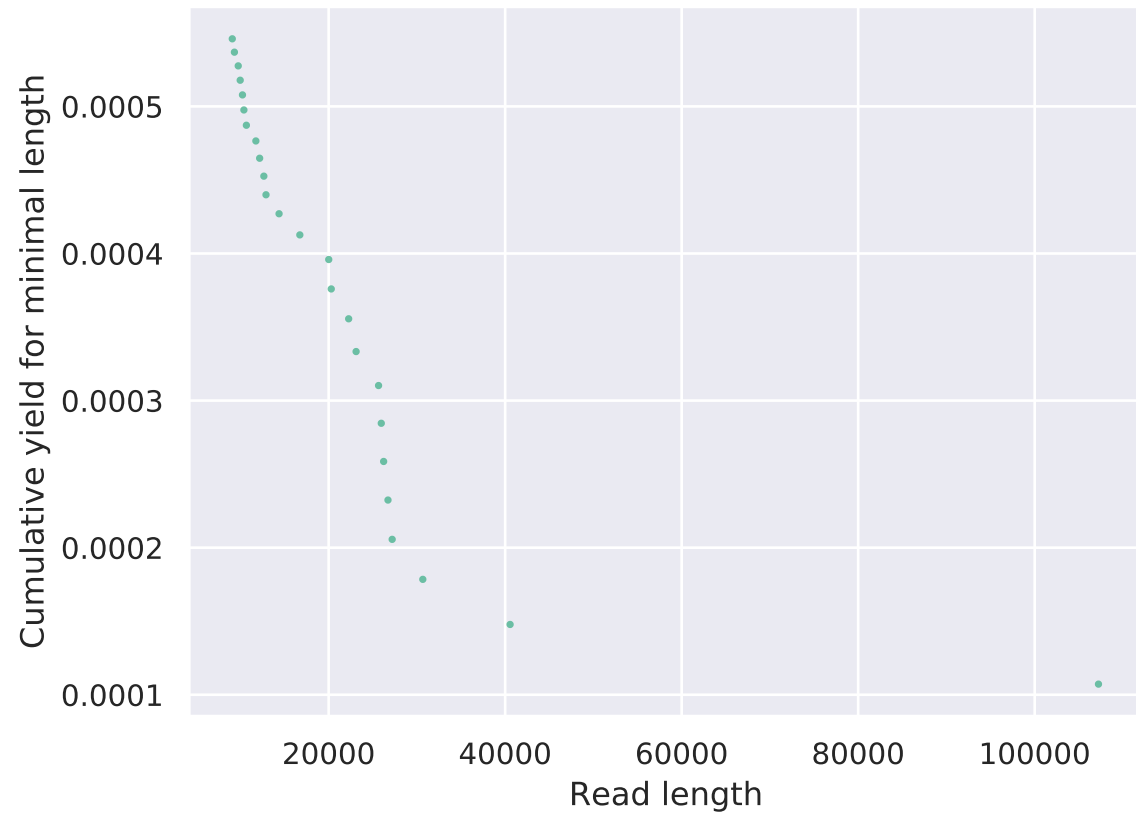

Supplement: Supplementary file 8 [file DataSheet_4.zip › SF1b/ccs999KIR7_18_4.contigs_MN167523_reports/ccs999KIR7_18_4.contigs_MN167523Yield_By_Length.pdf]

# Aligned read lengths vs Sequenced read length plot

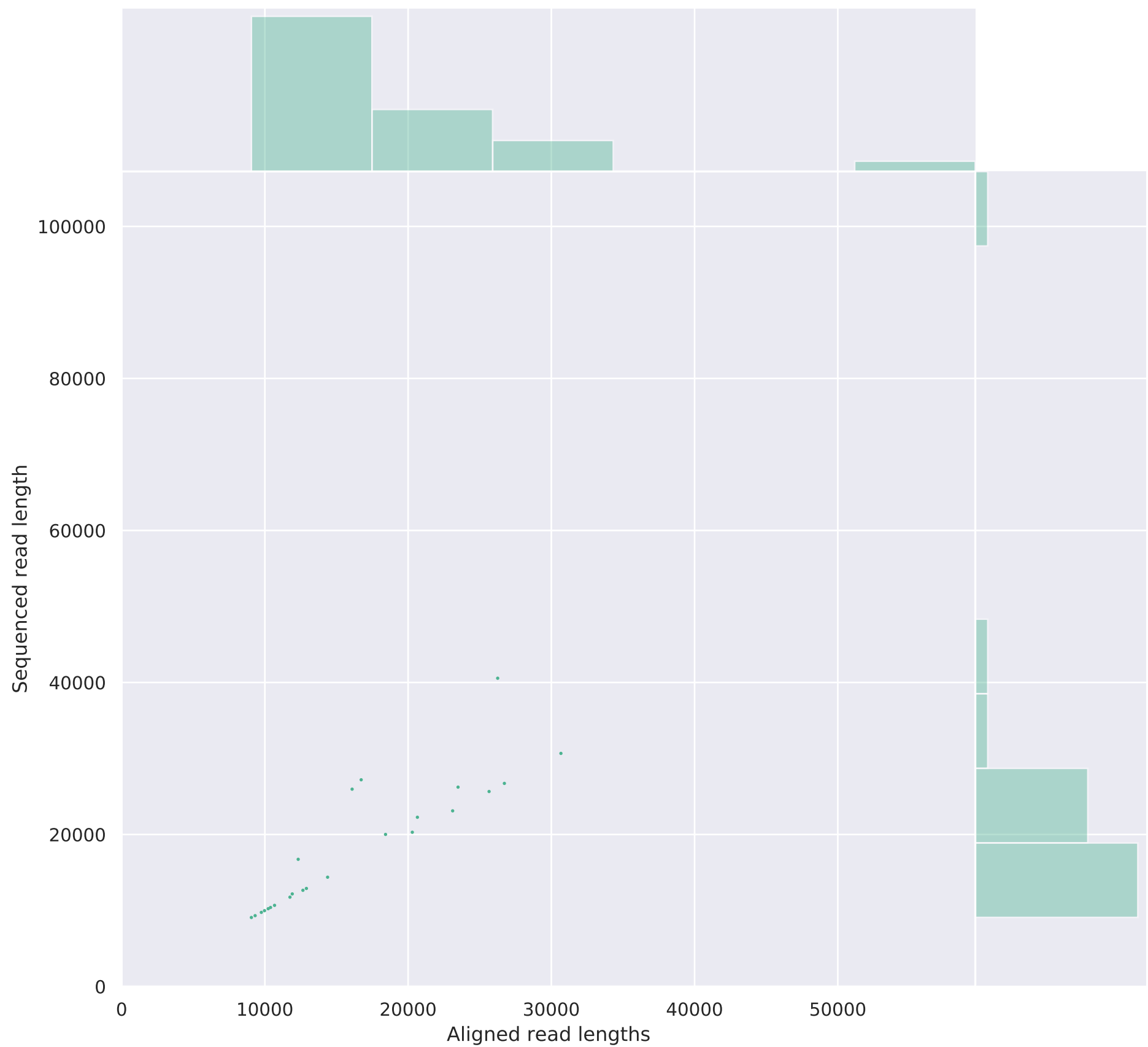

Supplement: Supplementary file 8 [file DataSheet_4.zip › SF1b/ccs999KIR7_18_4.contigs_MN167523_reports/ccs999KIR7_18_4.contigs_MN167523AlignedReadlengthvsSequencedReadLength_dot.pdf]

## Aligned read length vs Percent identity plot

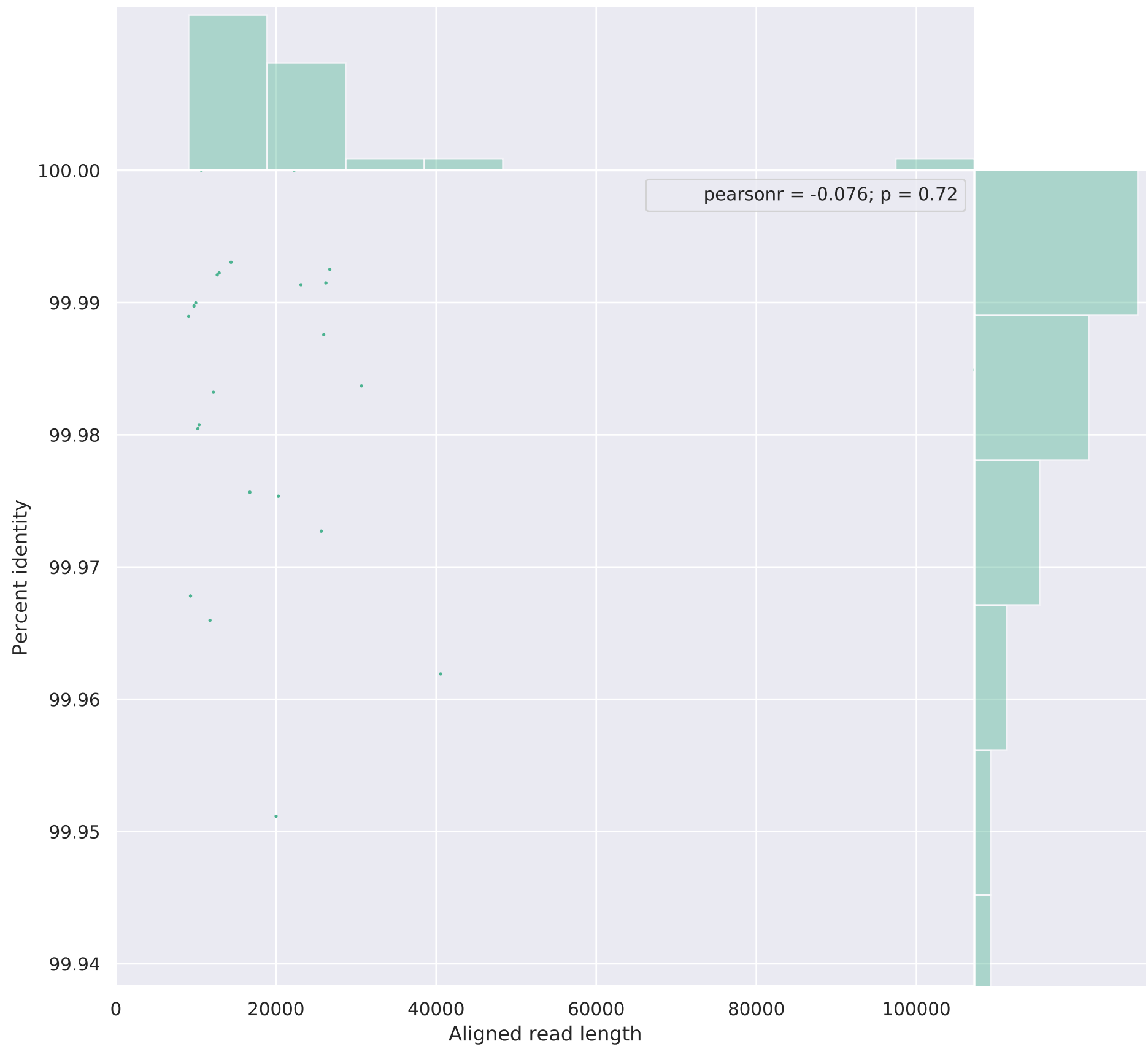

Supplement: Supplementary file 8 [file DataSheet_4.zip › SF1b/ccs999KIR7_18_4.contigs_MN167523_reports/ccs999KIR7_18_4.contigs_MN167523PercentIdentityvsAlignedReadLength_dot.pdf]

Weighted Histogram of read lengths

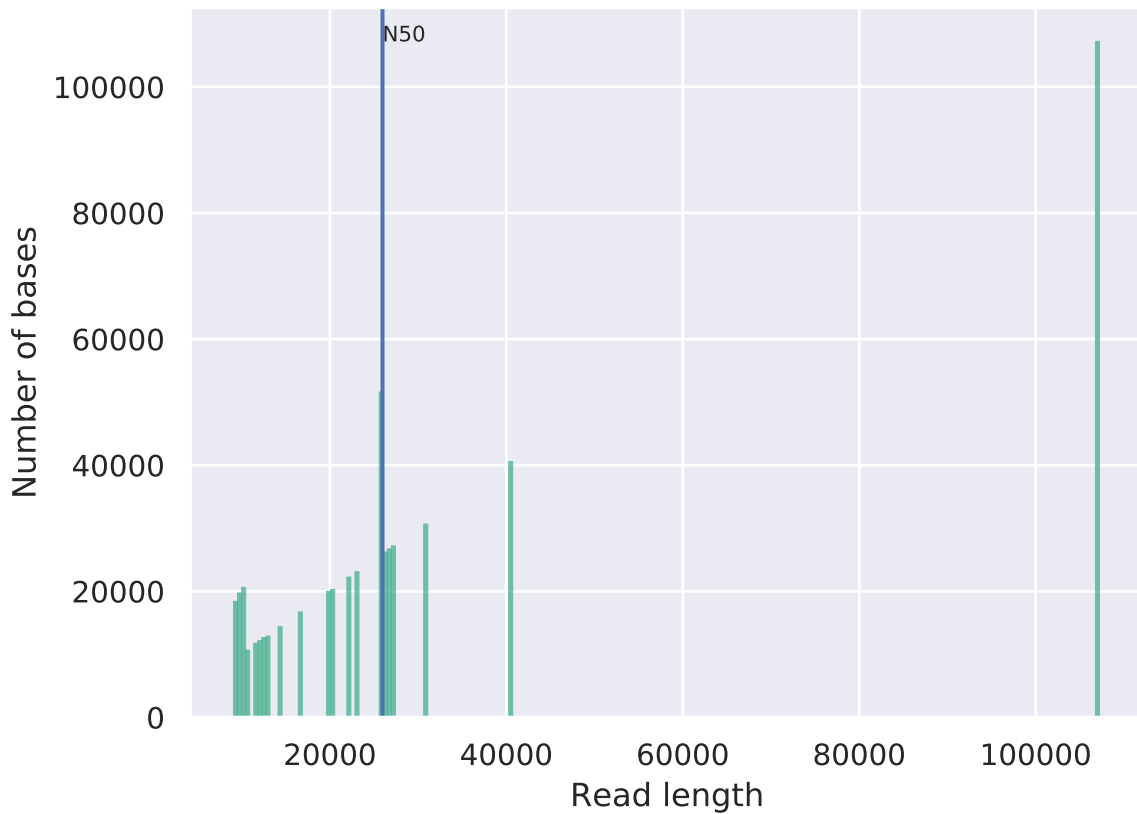

Supplement: Supplementary file 8 [file DataSheet_4.zip › SF1b/ccs999KIR7_18_4.contigs_MN167523_reports/ccs999KIR7_18_4.contigs_MN167523Weighted_HistogramReadlength.pdf]

Histogram of read lengths

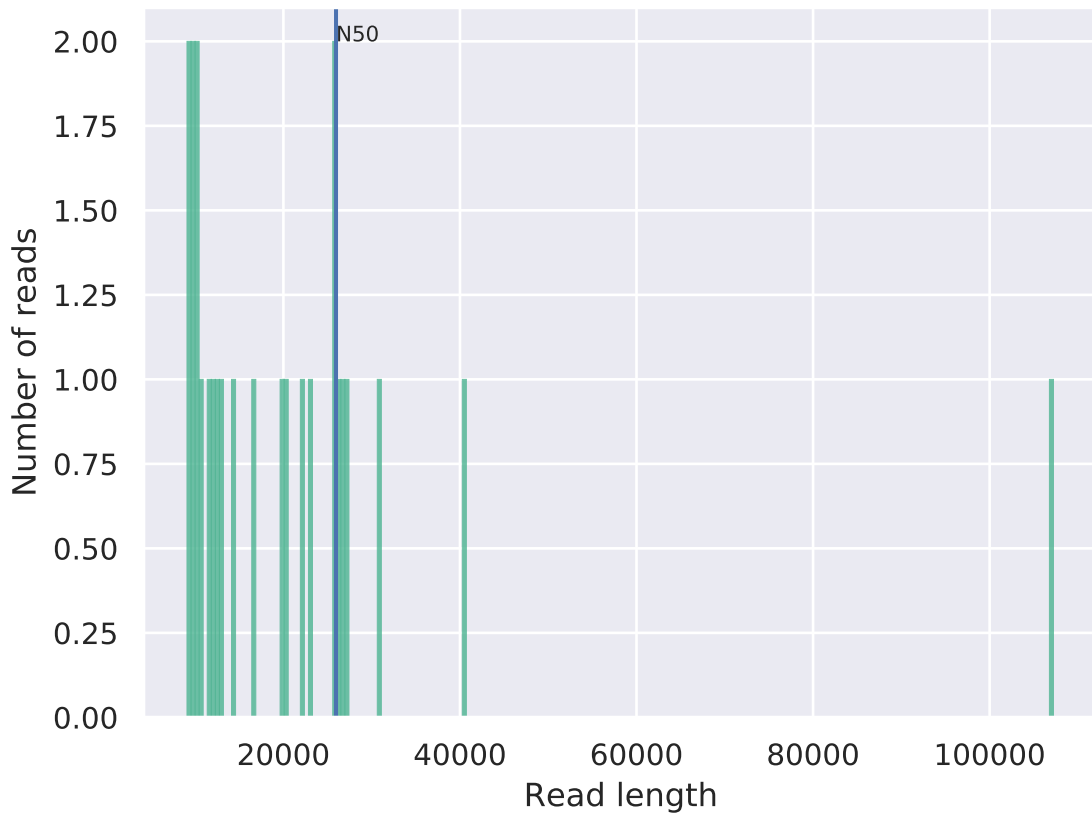

Supplement: Supplementary file 8 [file DataSheet_4.zip › SF1b/ccs999KIR7_18_4.contigs_MN167523_reports/ccs999KIR7_18_4.contigs_MN167523HistogramReadlength.pdf]

Weighted Histogram of read lengths after log transformation

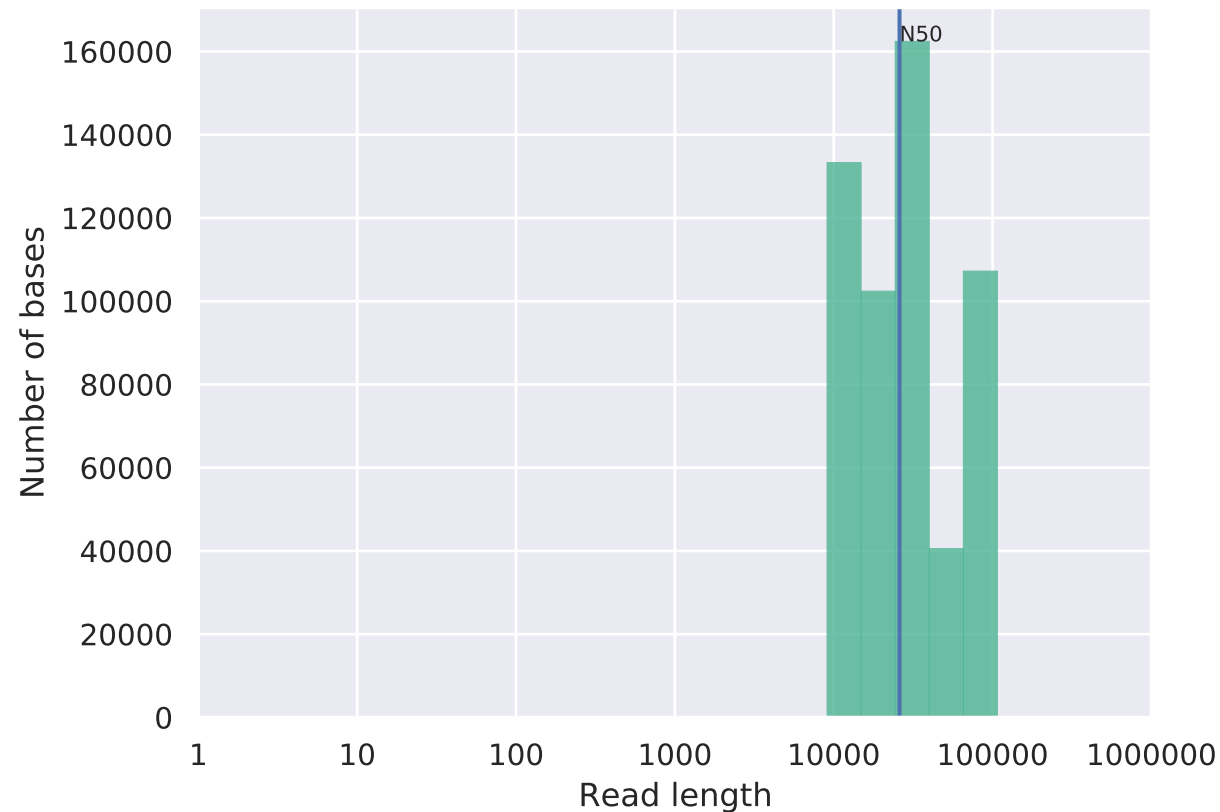

Supplement: Supplementary file 8 [file DataSheet_4.zip › SF1b/ccs999KIR7_18_4.contigs_MN167523_reports/ccs999KIR7_18_4.contigs_MN167523Weighted_LogTransformed_HistogramReadlength.pdf]

# Aligned read lengths vs Sequenced read length plot

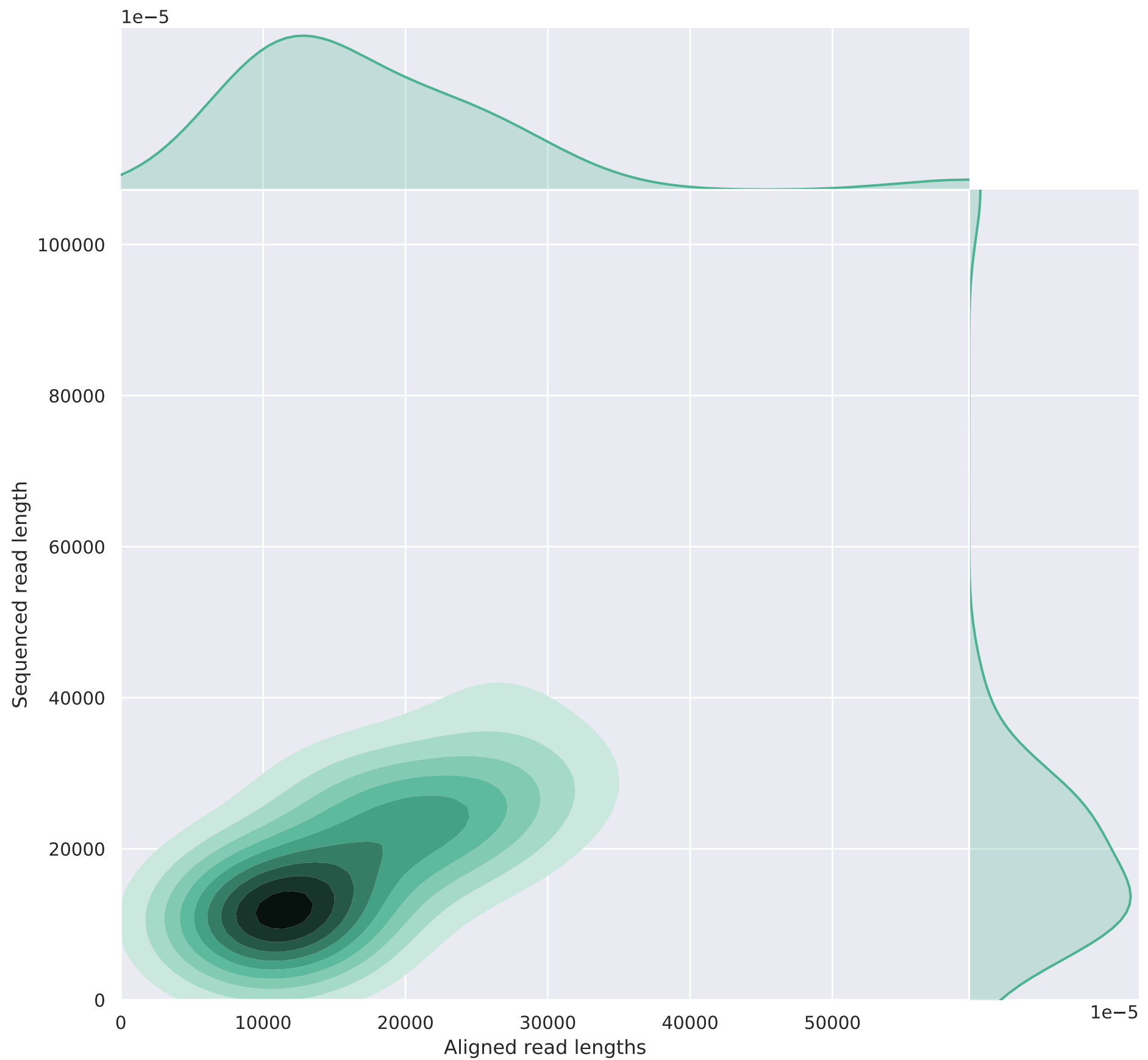

Supplement: Supplementary file 8 [file DataSheet_4.zip › SF1b/ccs999KIR7_18_4.contigs_MN167523_reports/ccs999KIR7_18_4.contigs_MN167523AlignedReadlengthvsSequencedReadLength_kde.pdf]

# Aligned read length vs Percent identity plot

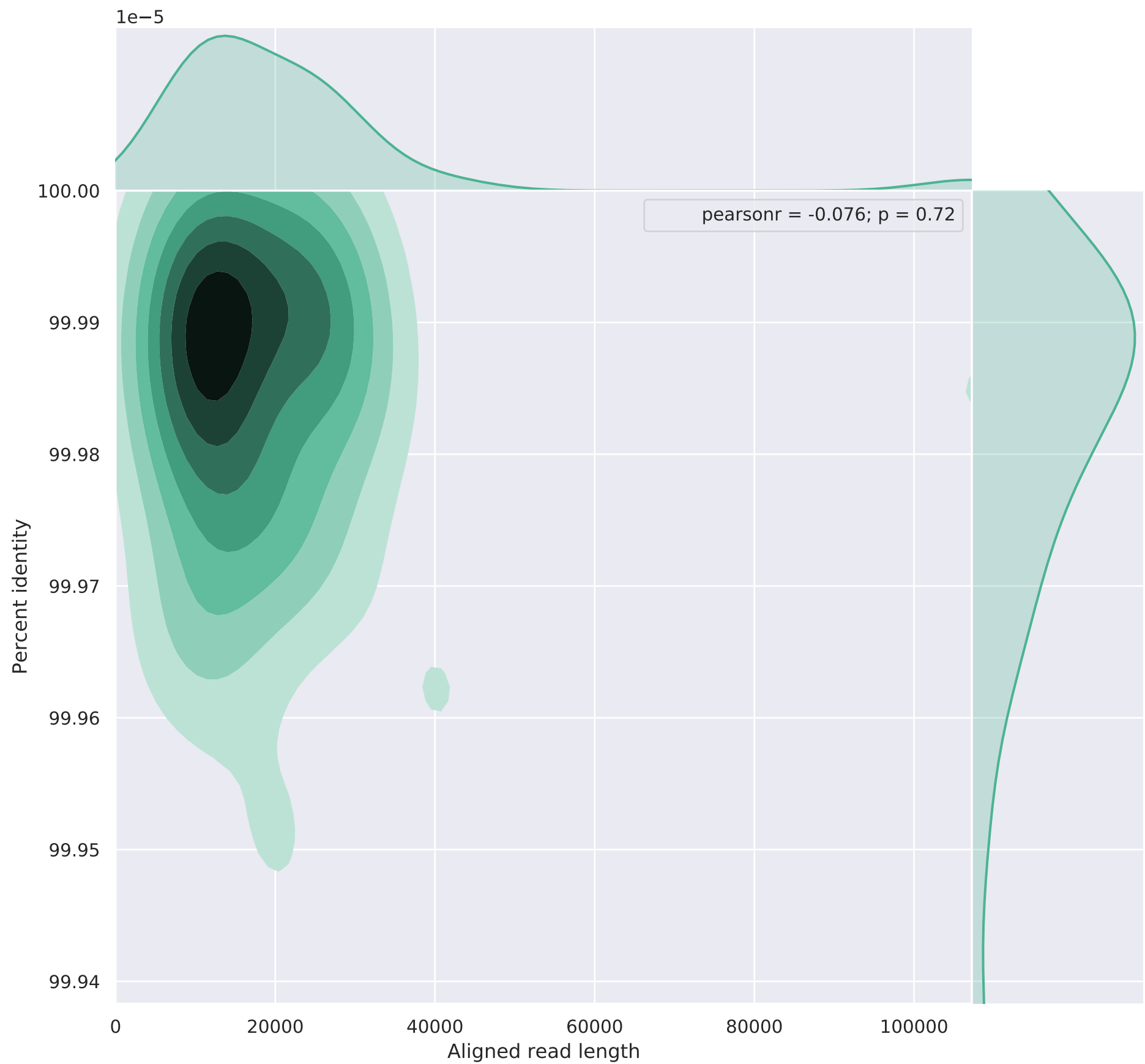

Supplement: Supplementary file 8 [file DataSheet_4.zip › SF1b/ccs999KIR7_18_4.contigs_MN167523_reports/ccs999KIR7_18_4.contigs_MN167523PercentIdentityvsAlignedReadLength_kde.pdf]

Histogram of read lengths after log transformation

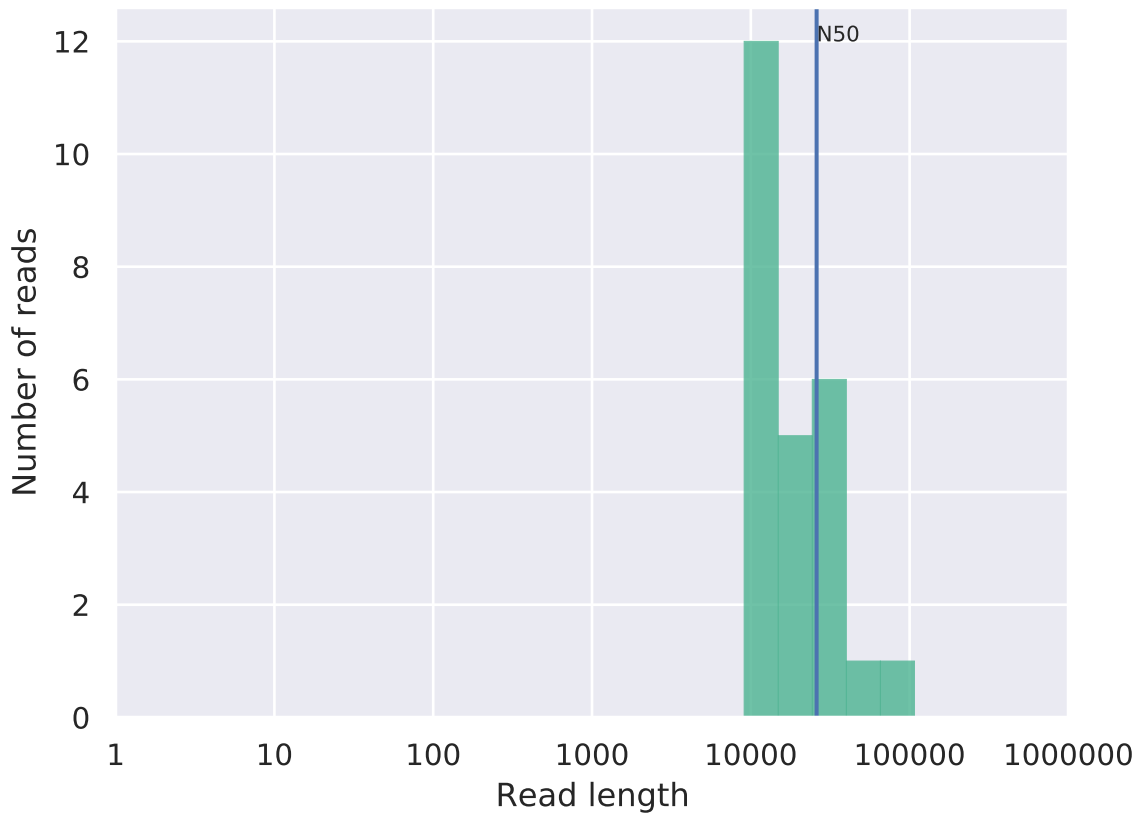

Supplement: Supplementary file 8 [file DataSheet_4.zip › SF1b/ccs999KIR7_18_4.contigs_MN167523_reports/ccs999KIR7_18_4.contigs_MN167523LogTransformed_HistogramReadlength.pdf]

Histogram of read lengths

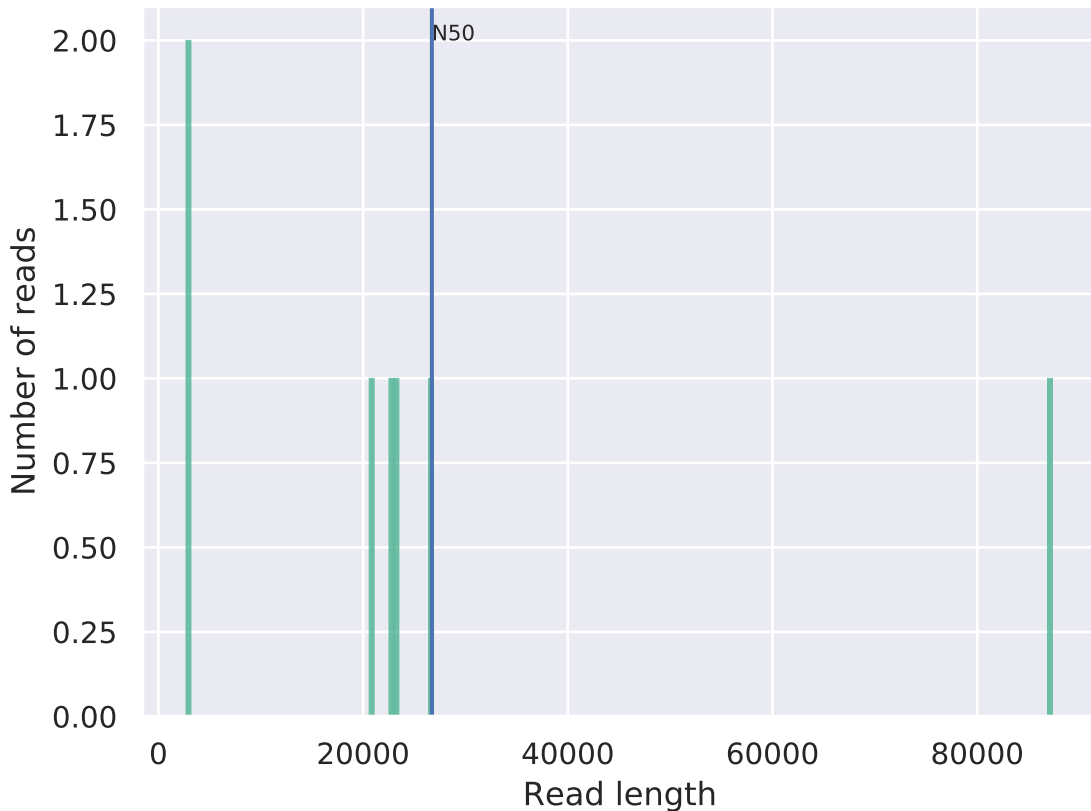

Supplement: Supplementary file 9 [file DataSheet_5.zip › SF1a/ccs999KIR7_18_2.contigs_MN167507_reports/ccs999KIR7_18_2.contigs_MN167507HistogramReadlength.pdf]

# Aligned read lengths vs Sequenced read length plot

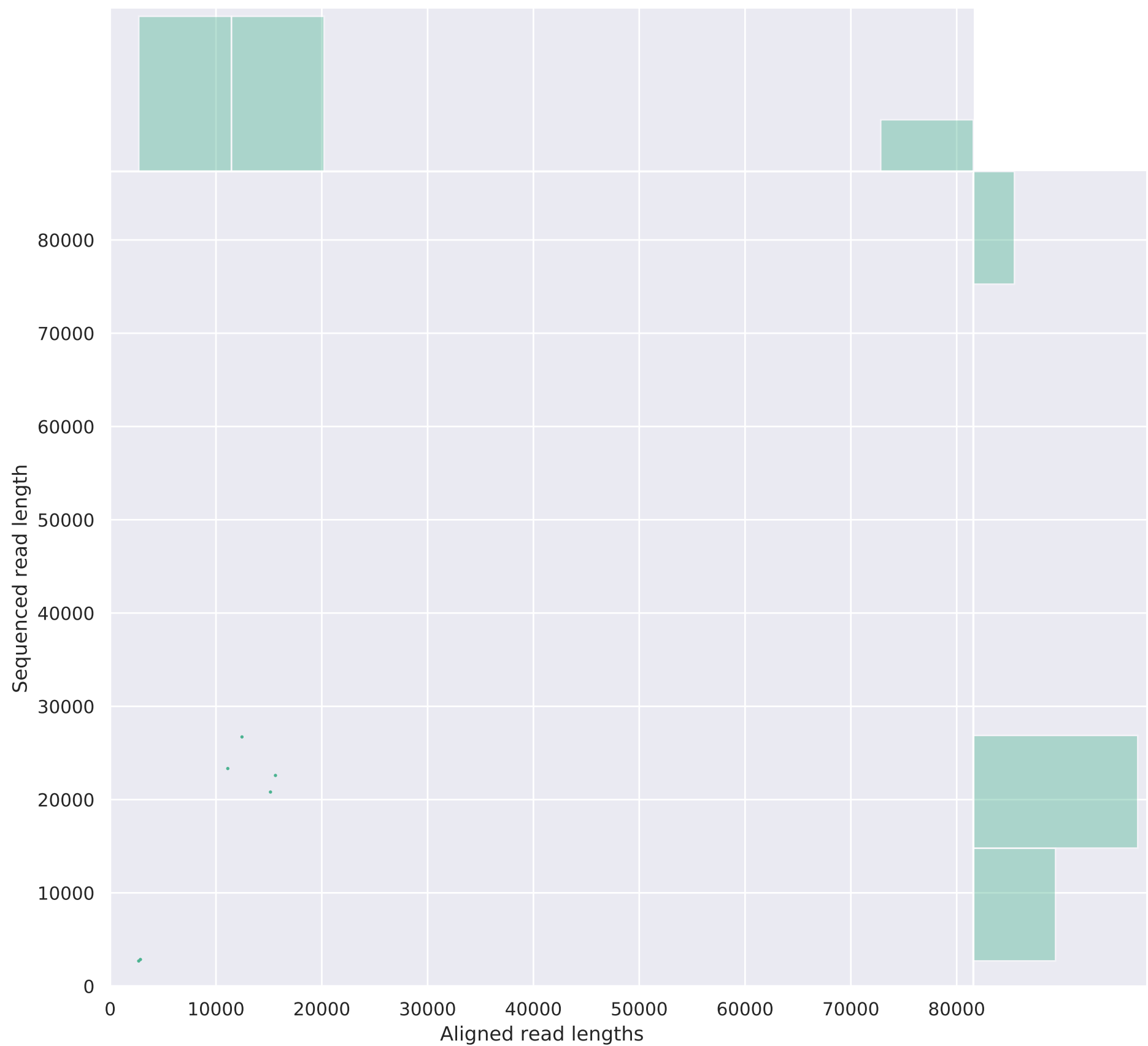

Supplement: Supplementary file 9 [file DataSheet_5.zip › SF1a/ccs999KIR7_18_2.contigs_MN167507_reports/ccs999KIR7_18_2.contigs_MN167507AlignedReadlengthvsSequencedReadLength_dot.pdf]

Weighted Histogram of read lengths after log transformation

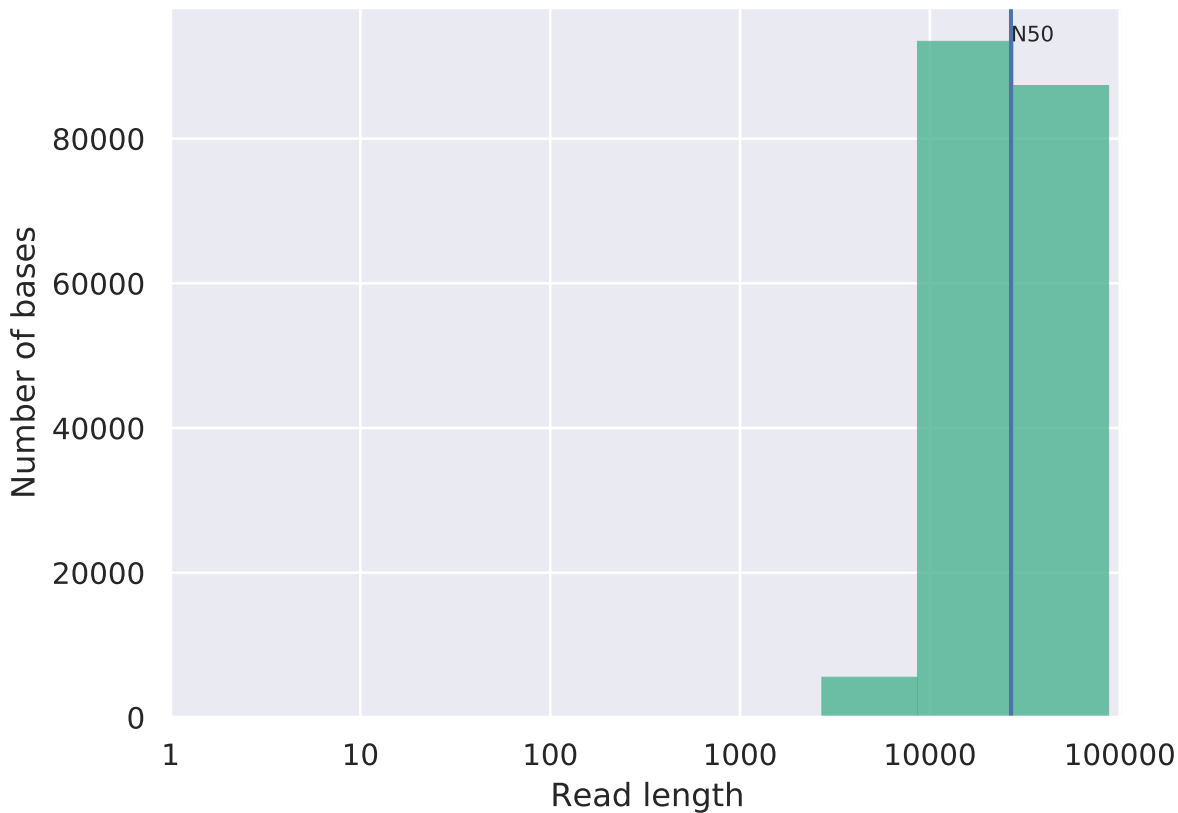

Supplement: Supplementary file 9 [file DataSheet_5.zip › SF1a/ccs999KIR7_18_2.contigs_MN167507_reports/ccs999KIR7_18_2.contigs_MN167507Weighted_LogTransformed_HistogramReadlength.pdf]

Histogram of read lengths after log transformation

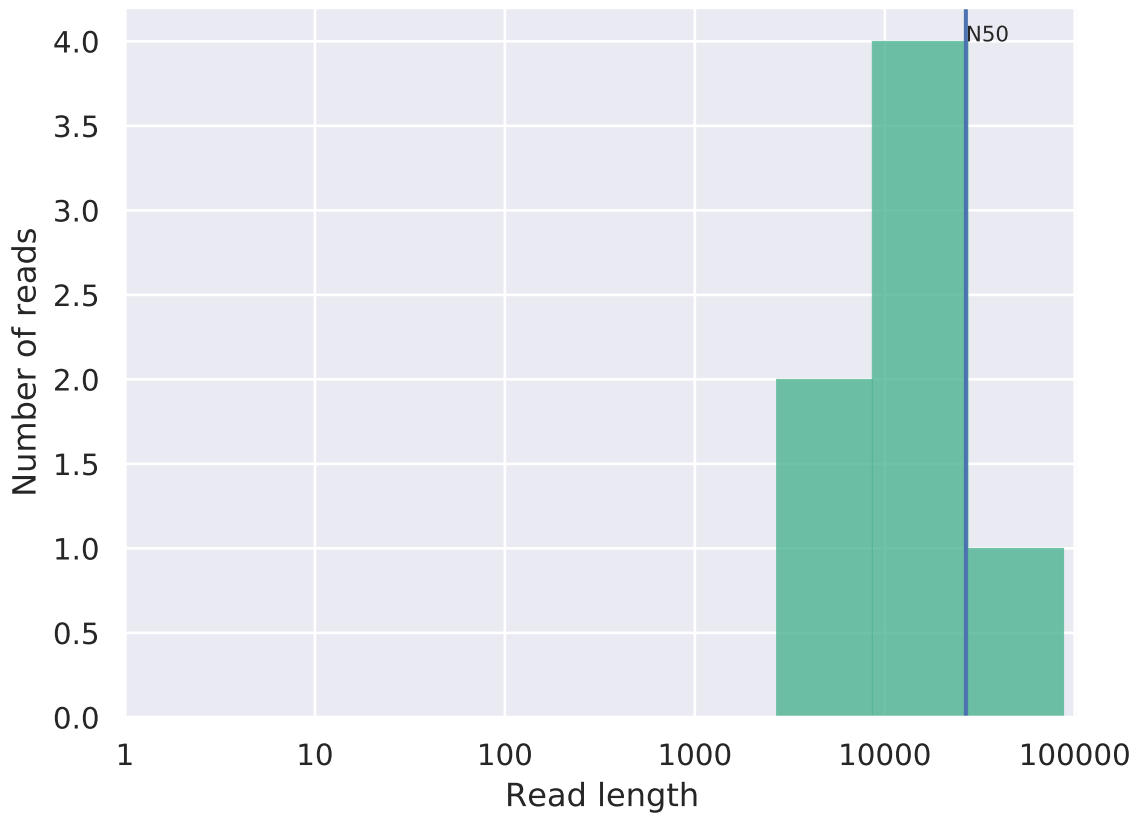

Supplement: Supplementary file 9 [file DataSheet_5.zip › SF1a/ccs999KIR7_18_2.contigs_MN167507_reports/ccs999KIR7_18_2.contigs_MN167507LogTransformed_HistogramReadlength.pdf]

# Aligned read length vs Percent identity plot

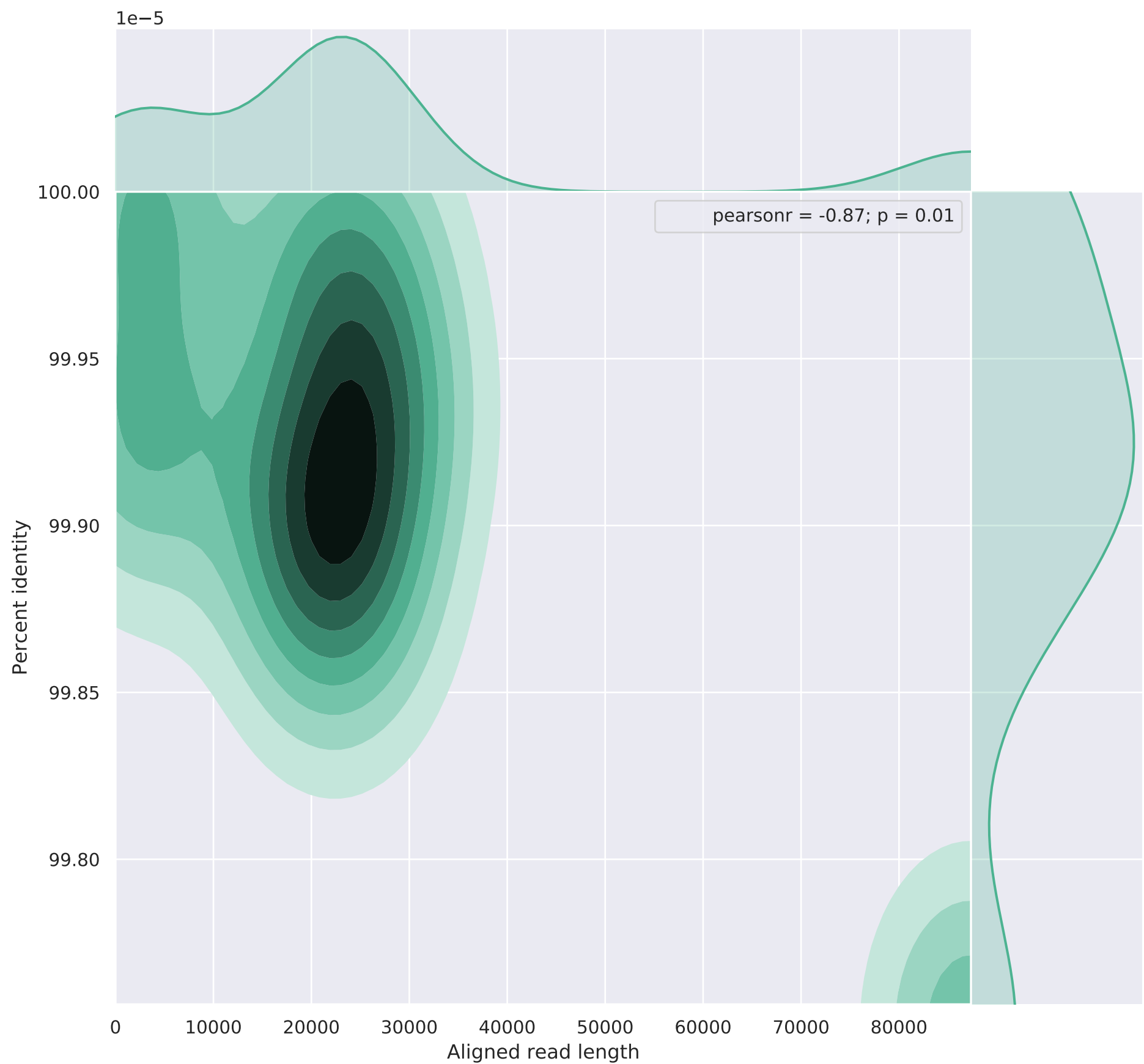

Supplement: Supplementary file 9 [file DataSheet_5.zip › SF1a/ccs999KIR7_18_2.contigs_MN167507_reports/ccs999KIR7_18_2.contigs_MN167507PercentIdentityvsAlignedReadLength_kde.pdf]

# Aligned read length vs Percent identity plot

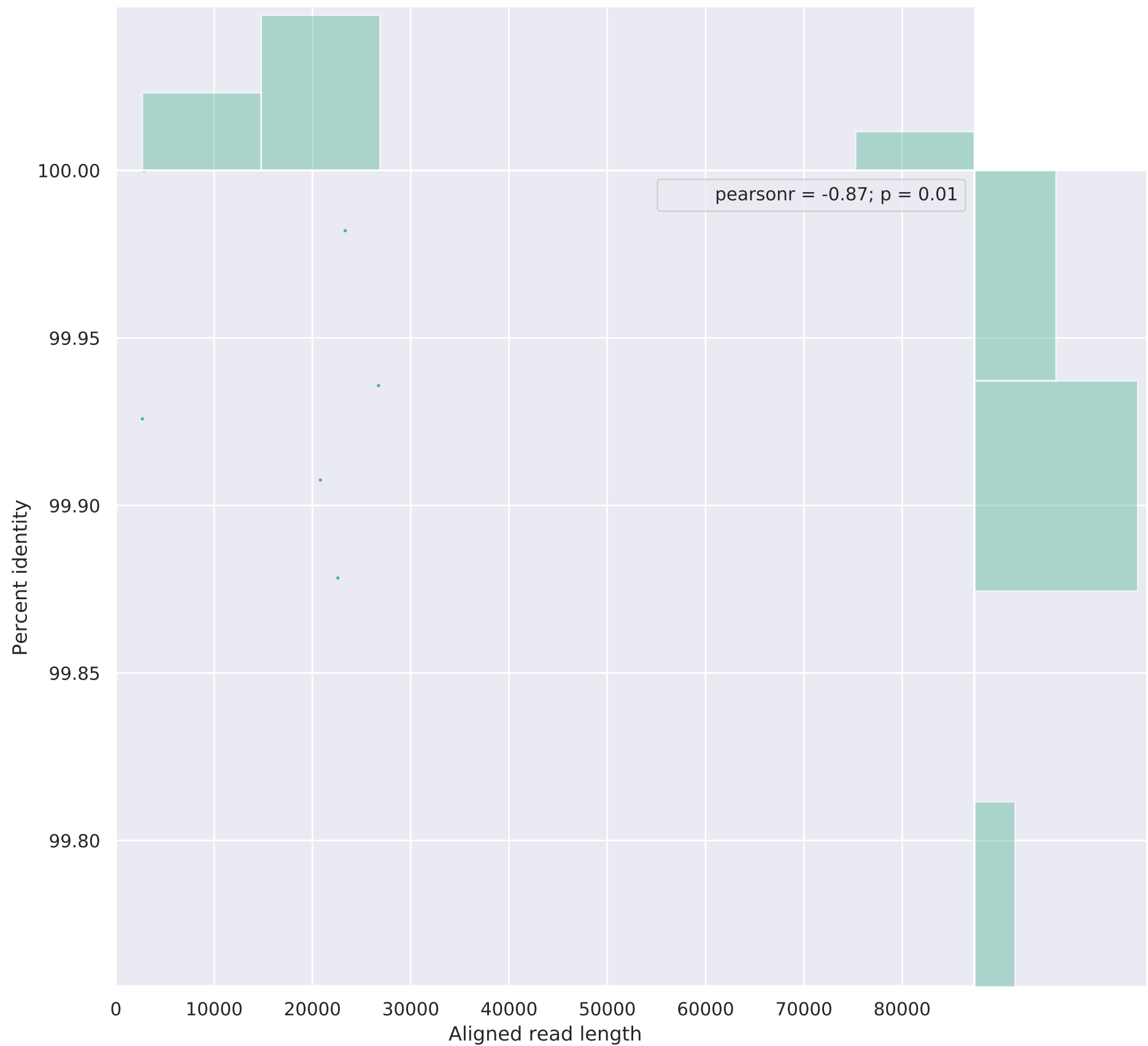

Supplement: Supplementary file 9 [file DataSheet_5.zip › SF1a/ccs999KIR7_18_2.contigs_MN167507_reports/ccs999KIR7_18_2.contigs_MN167507PercentIdentityvsAlignedReadLength_dot.pdf]

Weighted Histogram of read lengths

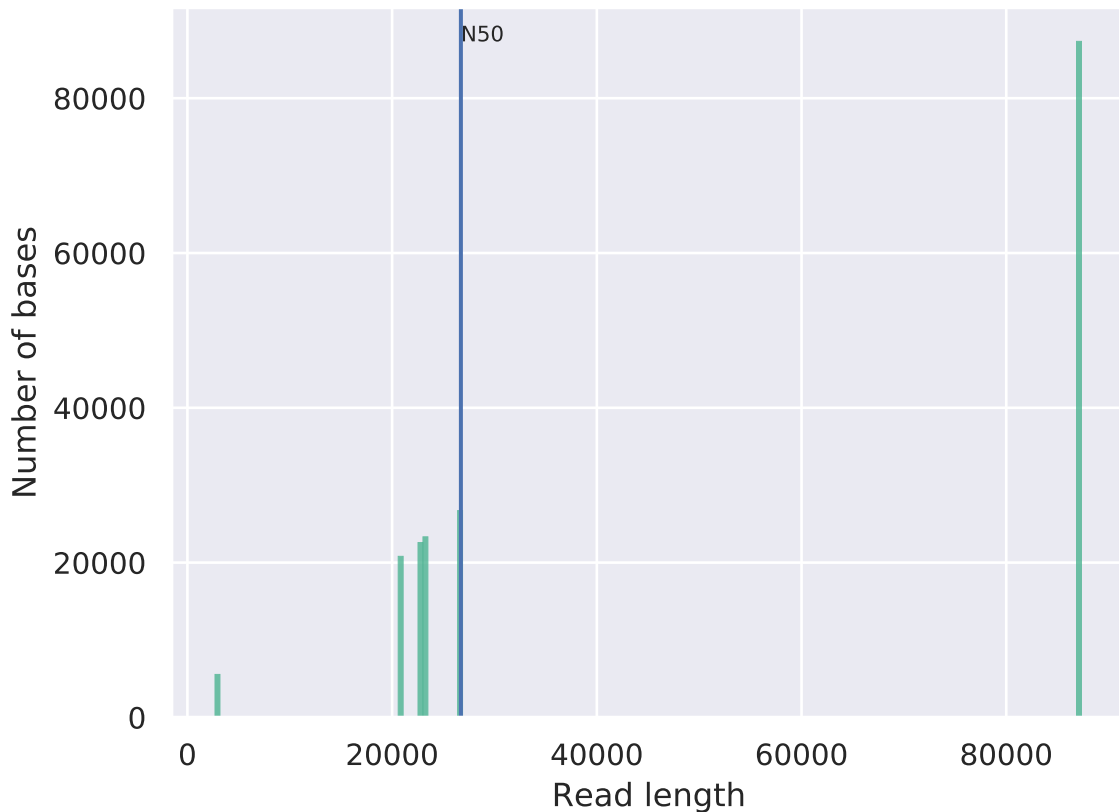

Supplement: Supplementary file 9 [file DataSheet_5.zip › SF1a/ccs999KIR7_18_2.contigs_MN167507_reports/ccs999KIR7_18_2.contigs_MN167507Weighted_HistogramReadlength.pdf]

# Aligned read lengths vs Sequenced read length plot

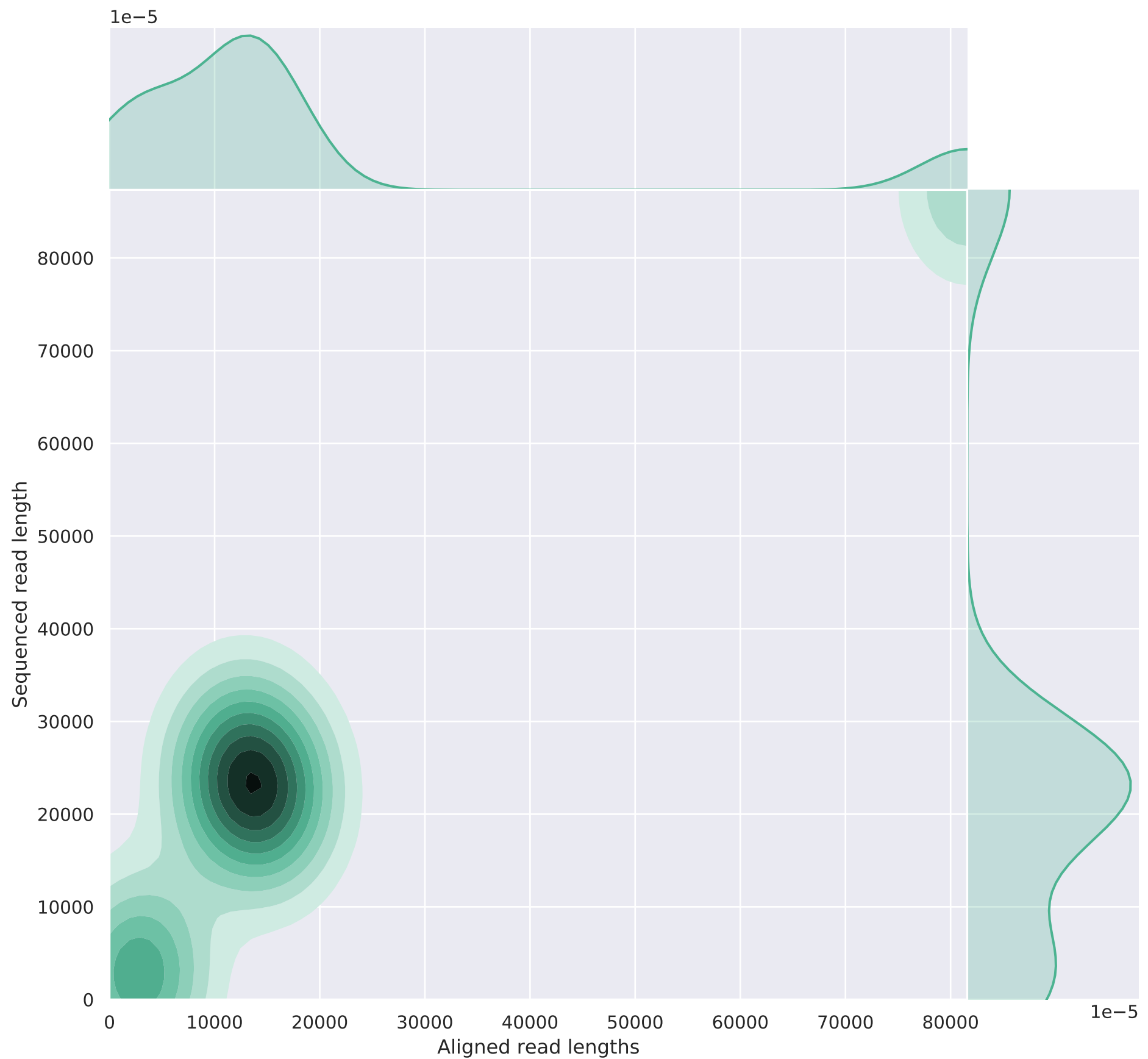

Supplement: Supplementary file 9 [file DataSheet_5.zip › SF1a/ccs999KIR7_18_2.contigs_MN167507_reports/ccs999KIR7_18_2.contigs_MN167507AlignedReadlengthvsSequencedReadLength_kde.pdf]

Yield by length

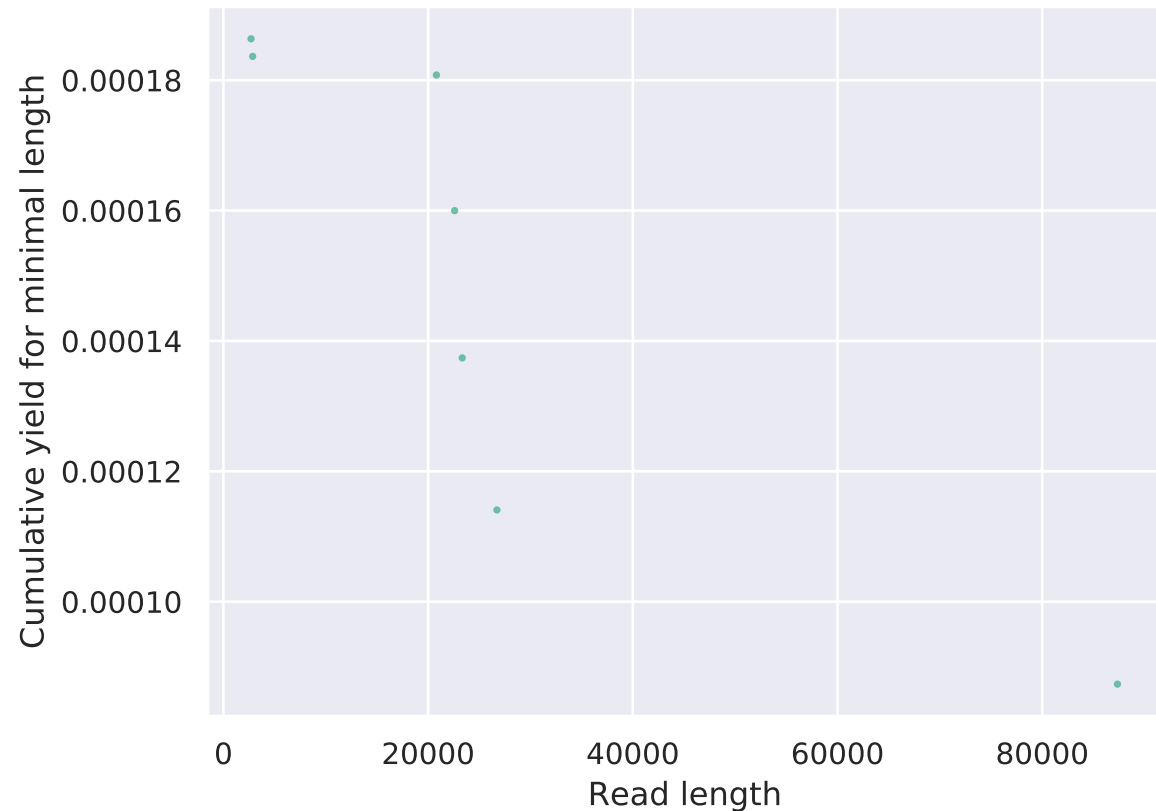

Supplement: Supplementary file 9 [file DataSheet_5.zip › SF1a/ccs999KIR7_18_2.contigs_MN167507_reports/ccs999KIR7_18_2.contigs_MN167507Yield_By_Length.pdf]

Histogram of read lengths after log transformation

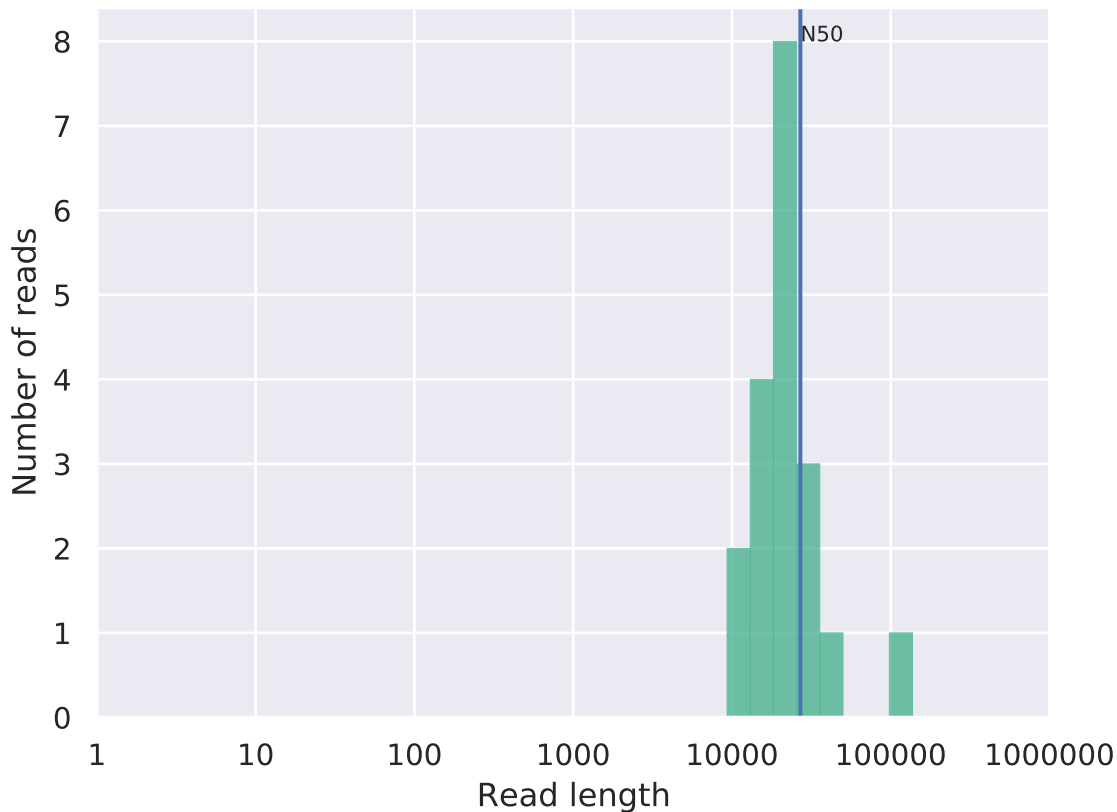

Supplement: Supplementary file 9 [file DataSheet_5.zip › SF1a/ccs999KIR7_18_1.contigs_MN167527_reports/ccs999KIR7_18_1.contigs_MN167527LogTransformed_HistogramReadlength.pdf]

# Aligned read lengths vs Sequenced read length plot

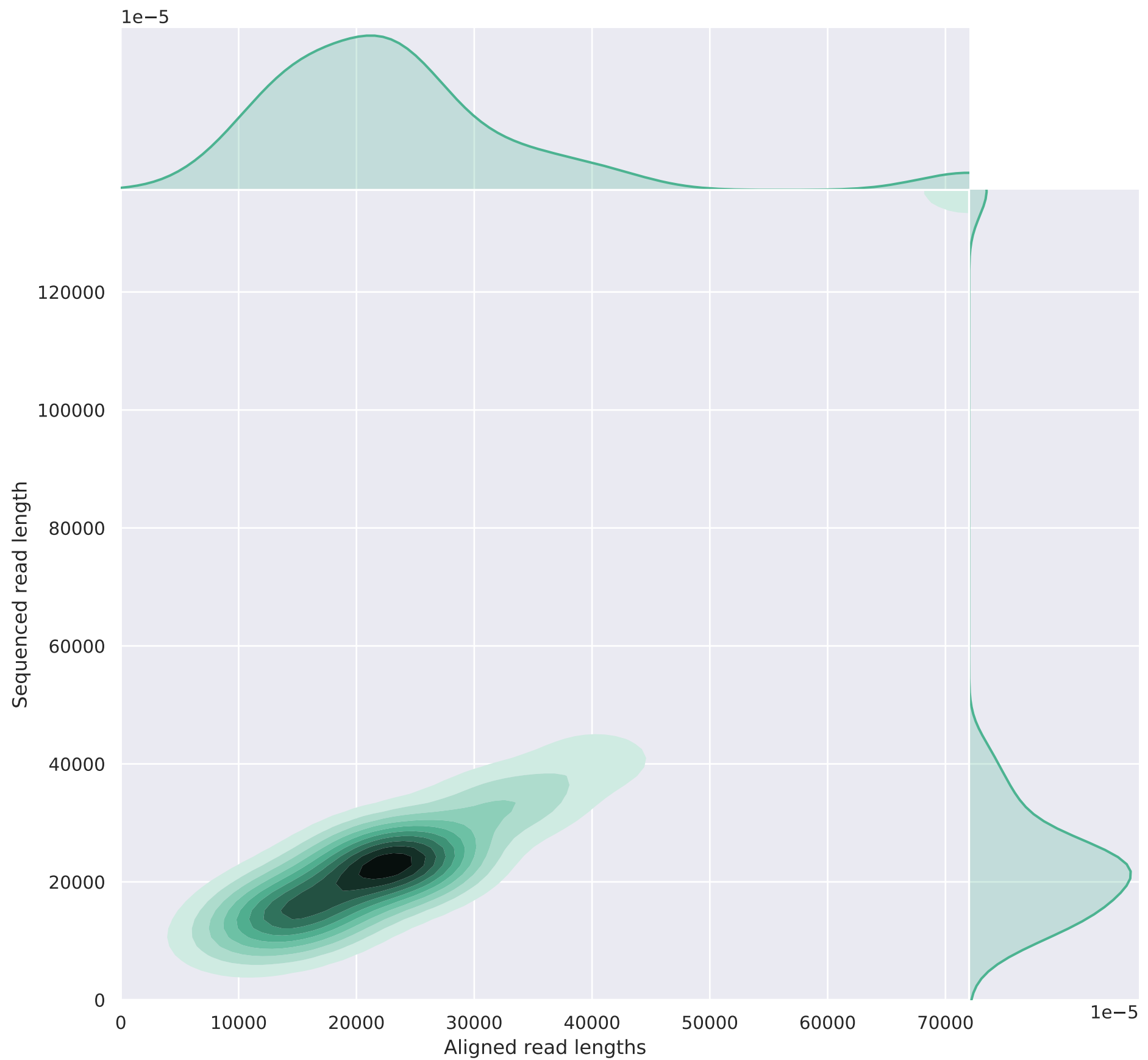

Supplement: Supplementary file 9 [file DataSheet_5.zip › SF1a/ccs999KIR7_18_1.contigs_MN167527_reports/ccs999KIR7_18_1.contigs_MN167527AlignedReadlengthvsSequencedReadLength_kde.pdf]

# Aligned read length vs Percent identity plot

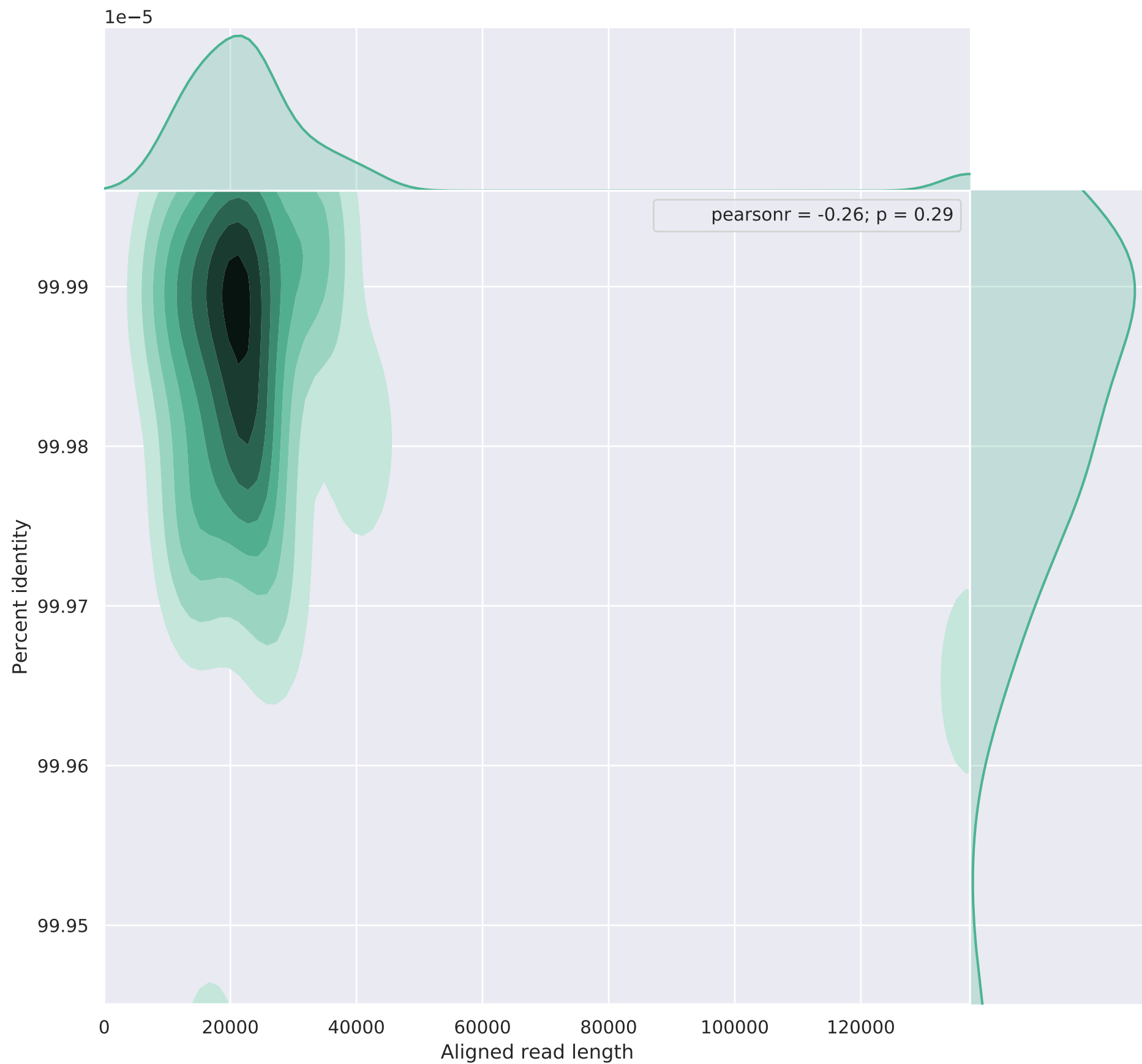

Supplement: Supplementary file 9 [file DataSheet_5.zip › SF1a/ccs999KIR7_18_1.contigs_MN167527_reports/ccs999KIR7_18_1.contigs_MN167527PercentIdentityvsAlignedReadLength_kde.pdf]

Weighted Histogram of read lengths after log transformation

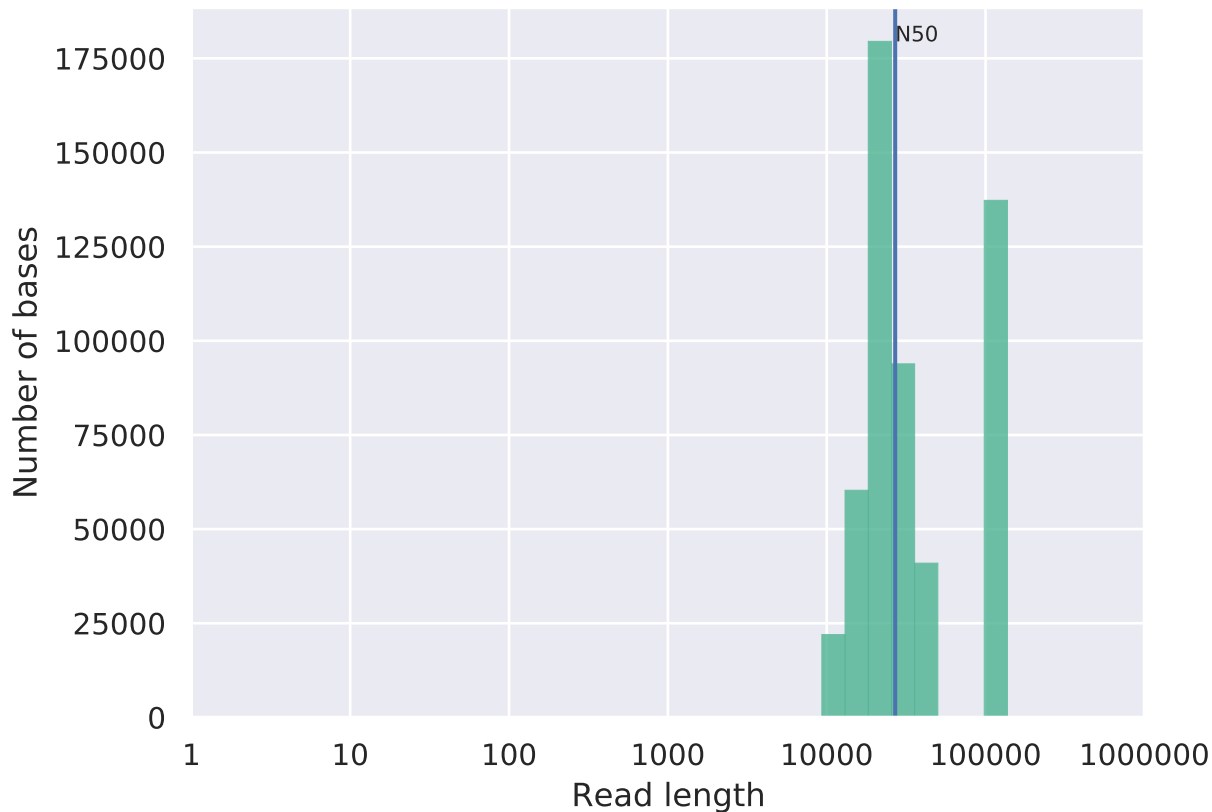

Supplement: Supplementary file 9 [file DataSheet_5.zip › SF1a/ccs999KIR7_18_1.contigs_MN167527_reports/ccs999KIR7_18_1.contigs_MN167527Weighted_LogTransformed_HistogramReadlength.pdf]

# Aligned read lengths vs Sequenced read length plot

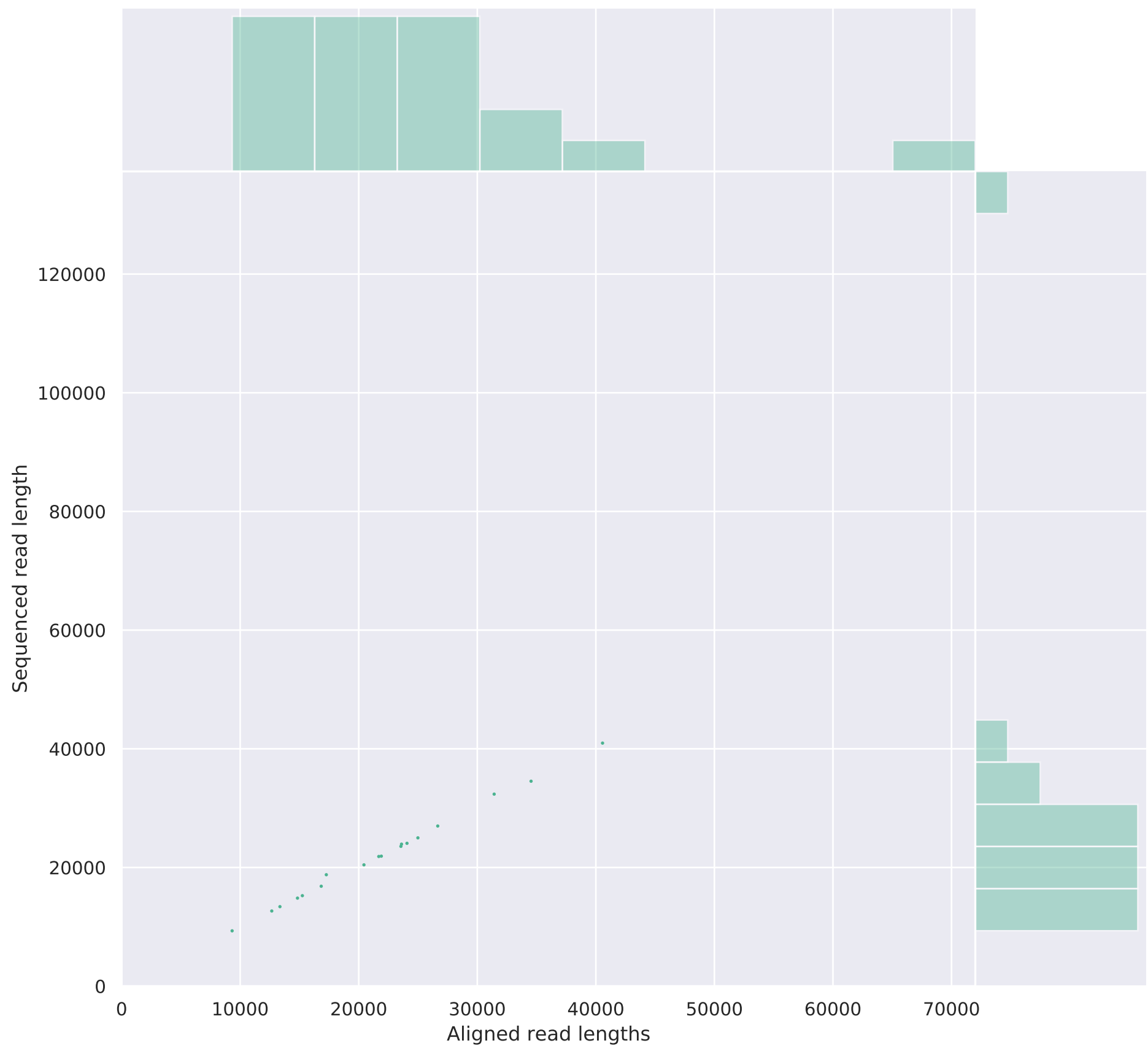

Supplement: Supplementary file 9 [file DataSheet_5.zip › SF1a/ccs999KIR7_18_1.contigs_MN167527_reports/ccs999KIR7_18_1.contigs_MN167527AlignedReadlengthvsSequencedReadLength_dot.pdf]

# Aligned read length vs Percent identity plot

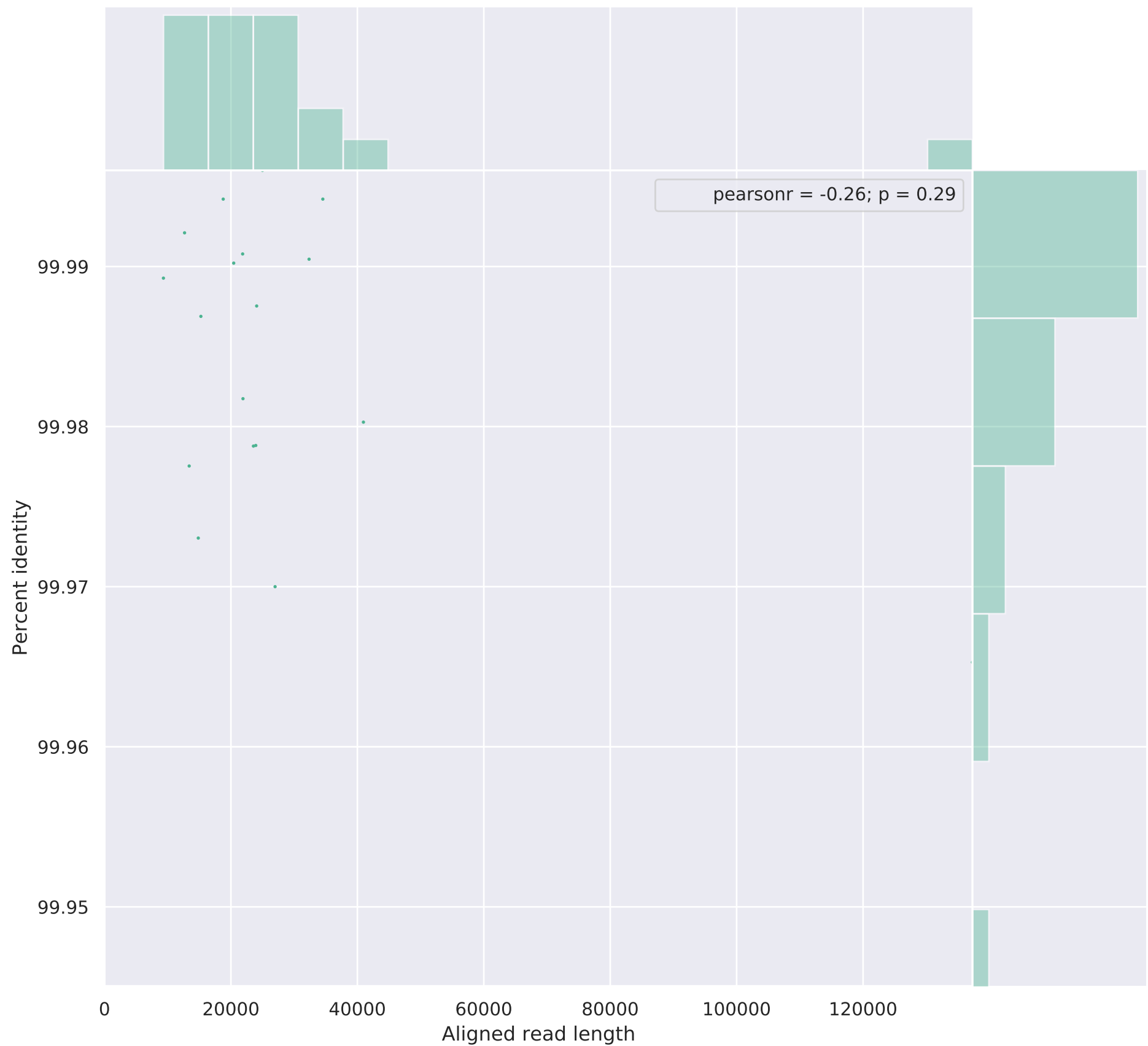

Supplement: Supplementary file 9 [file DataSheet_5.zip › SF1a/ccs999KIR7_18_1.contigs_MN167527_reports/ccs999KIR7_18_1.contigs_MN167527PercentIdentityvsAlignedReadLength_dot.pdf]

Yield by length

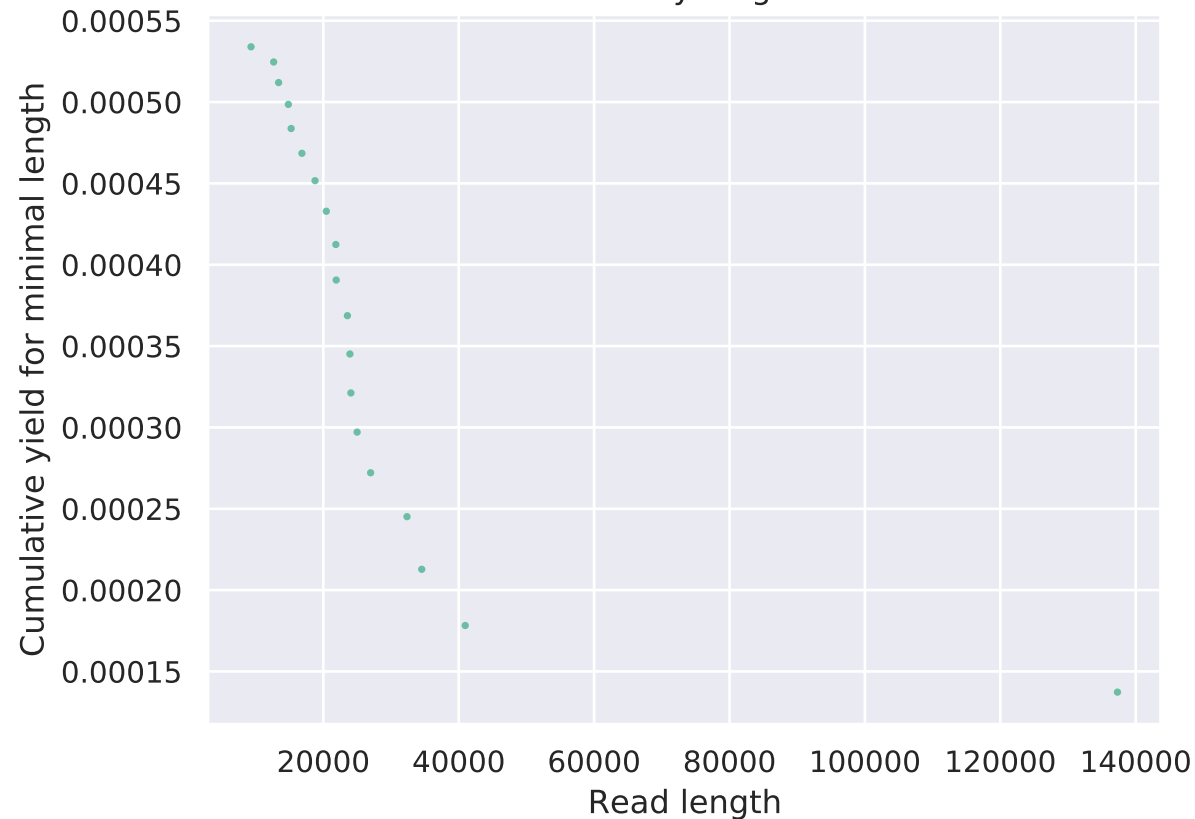

Supplement: Supplementary file 9 [file DataSheet_5.zip › SF1a/ccs999KIR7_18_1.contigs_MN167527_reports/ccs999KIR7_18_1.contigs_MN167527Yield_By_Length.pdf]

Histogram of read lengths

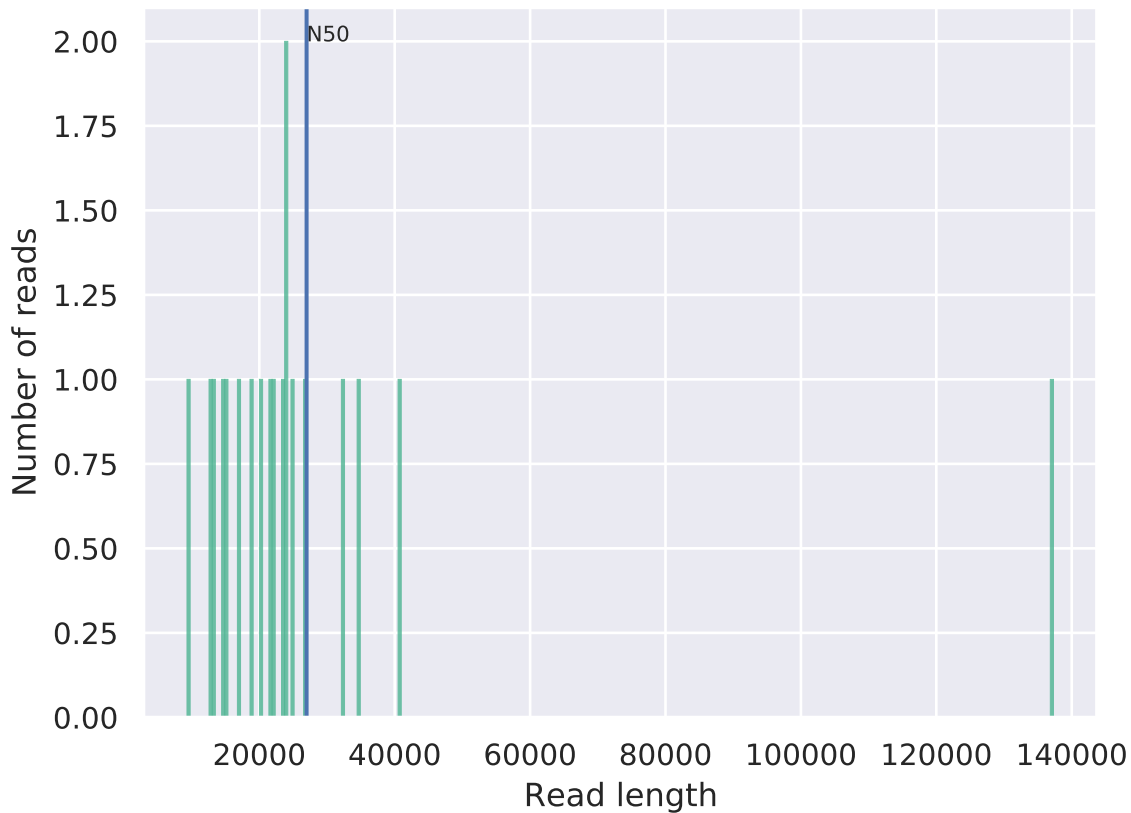

Supplement: Supplementary file 9 [file DataSheet_5.zip › SF1a/ccs999KIR7_18_1.contigs_MN167527_reports/ccs999KIR7_18_1.contigs_MN167527HistogramReadlength.pdf]

Weighted Histogram of read lengths

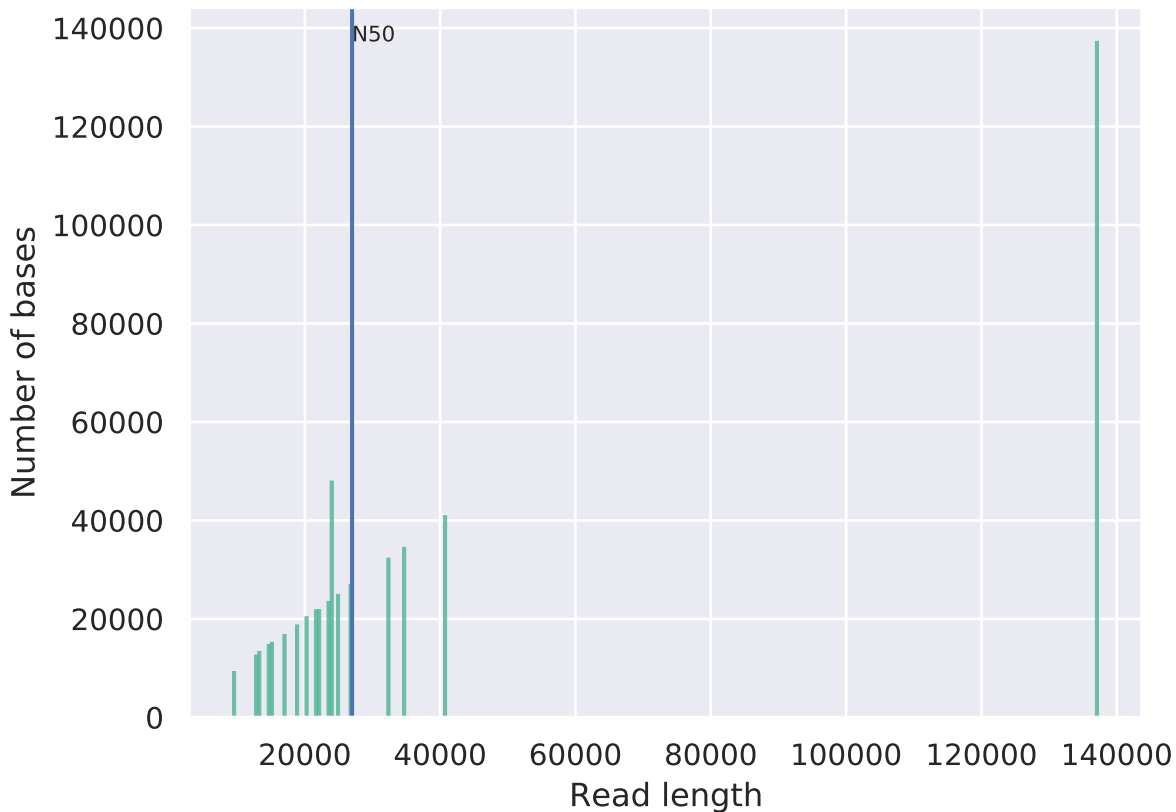

Supplement: Supplementary file 9 [file DataSheet_5.zip › SF1a/ccs999KIR7_18_1.contigs_MN167527_reports/ccs999KIR7_18_1.contigs_MN167527Weighted_HistogramReadlength.pdf]

Histogram of read lengths after log transformation

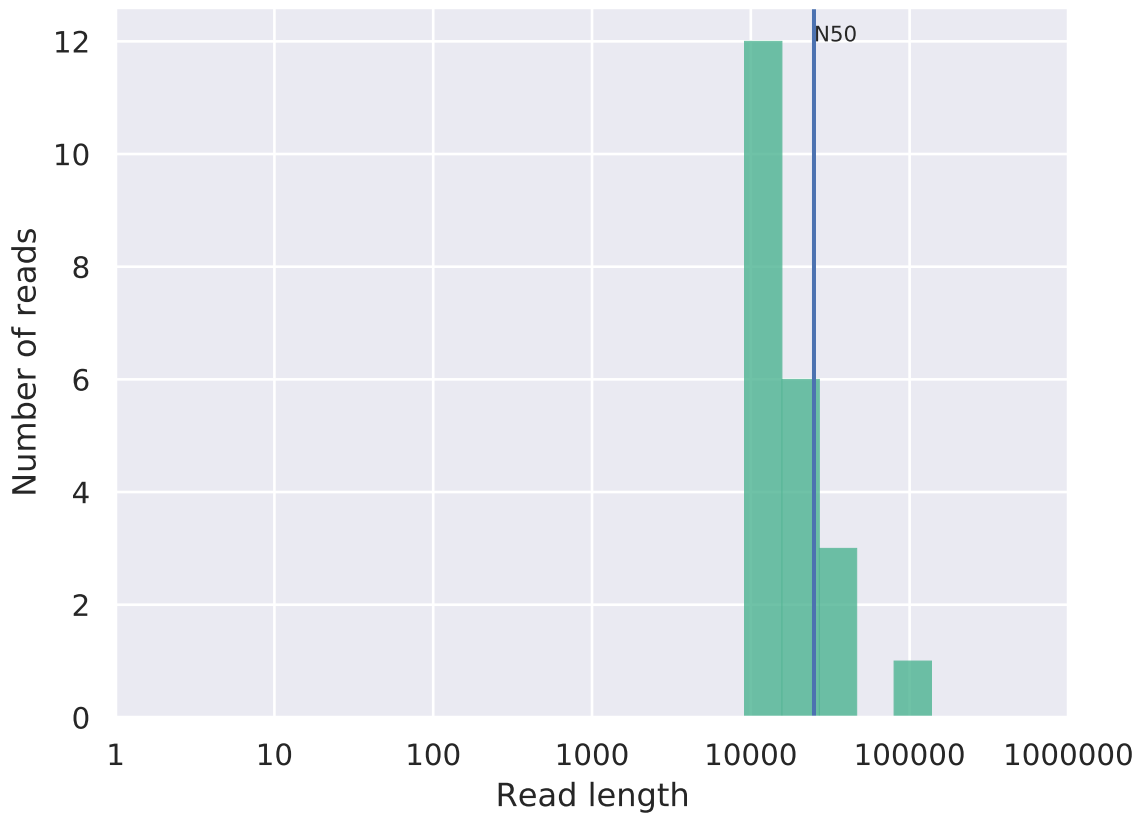

Supplement: Supplementary file 9 [file DataSheet_5.zip › SF1a/ccs999KIR7_18_1.contigs_MN167526_reports/ccs999KIR7_18_1.contigs_MN167526LogTransformed_HistogramReadlength.pdf]

Weighted Histogram of read lengths

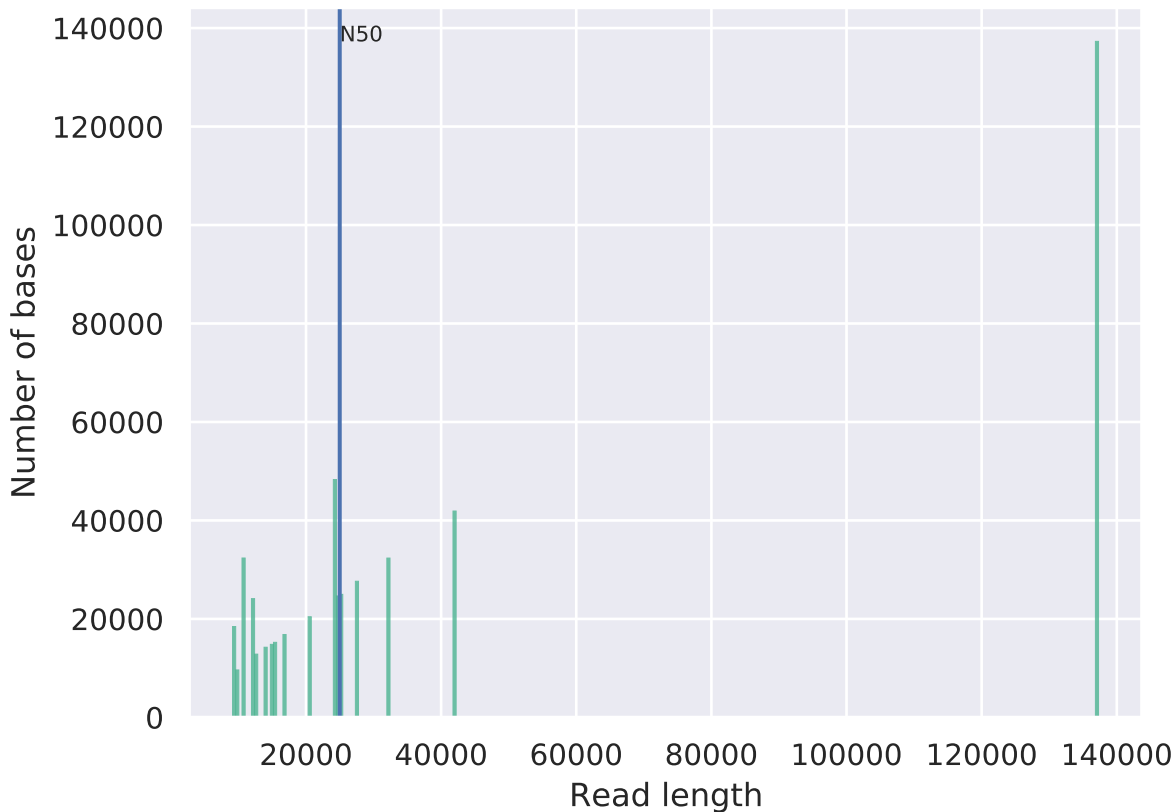

Supplement: Supplementary file 9 [file DataSheet_5.zip › SF1a/ccs999KIR7_18_1.contigs_MN167526_reports/ccs999KIR7_18_1.contigs_MN167526Weighted_HistogramReadlength.pdf]

Yield by length

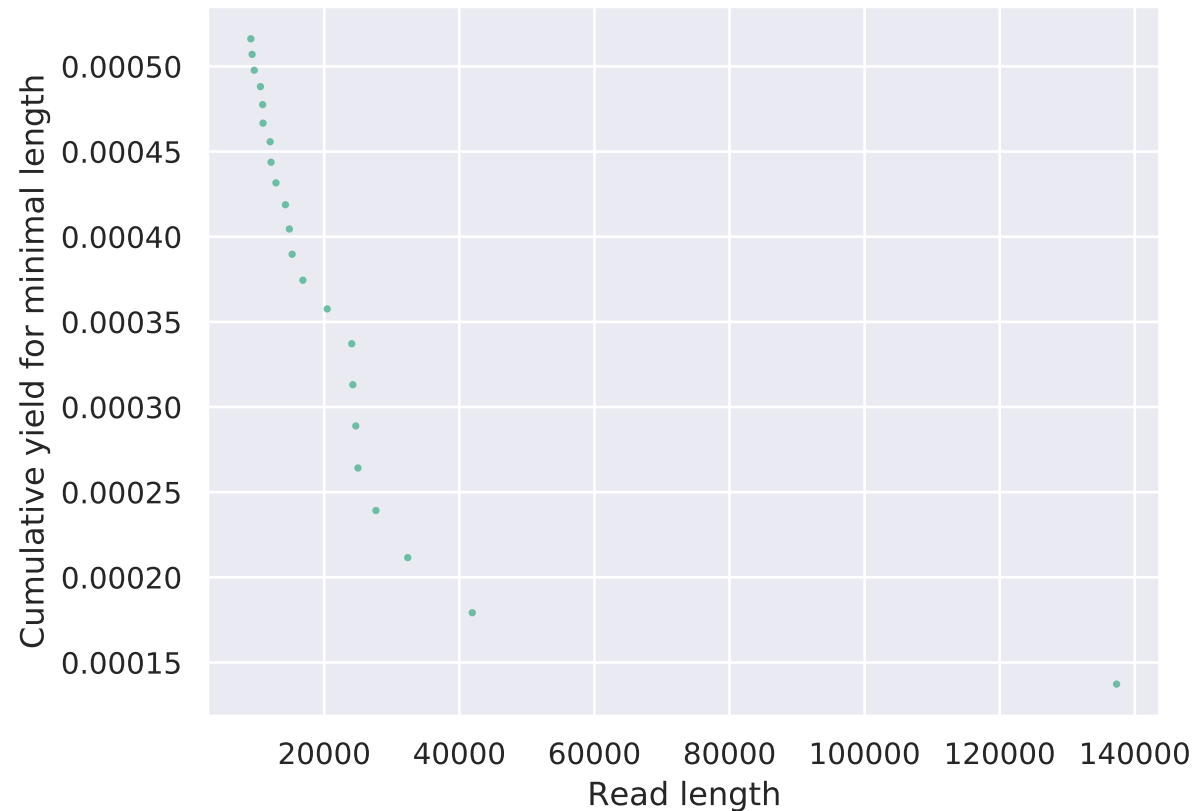

Supplement: Supplementary file 9 [file DataSheet_5.zip › SF1a/ccs999KIR7_18_1.contigs_MN167526_reports/ccs999KIR7_18_1.contigs_MN167526Yield_By_Length.pdf]

# Aligned read length vs Percent identity plot

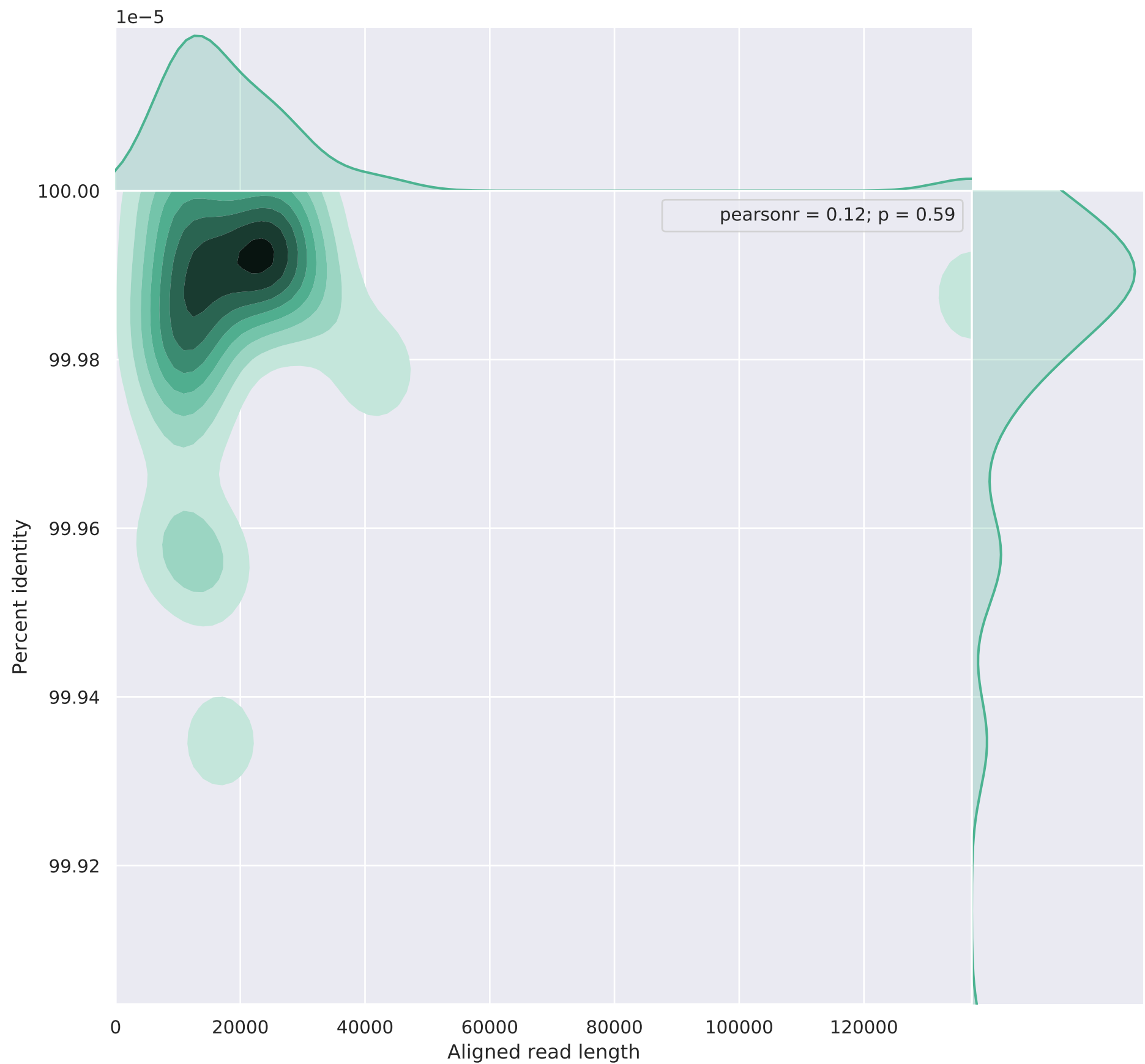

Supplement: Supplementary file 9 [file DataSheet_5.zip › SF1a/ccs999KIR7_18_1.contigs_MN167526_reports/ccs999KIR7_18_1.contigs_MN167526PercentIdentityvsAlignedReadLength_kde.pdf]

# Aligned read lengths vs Sequenced read length plot

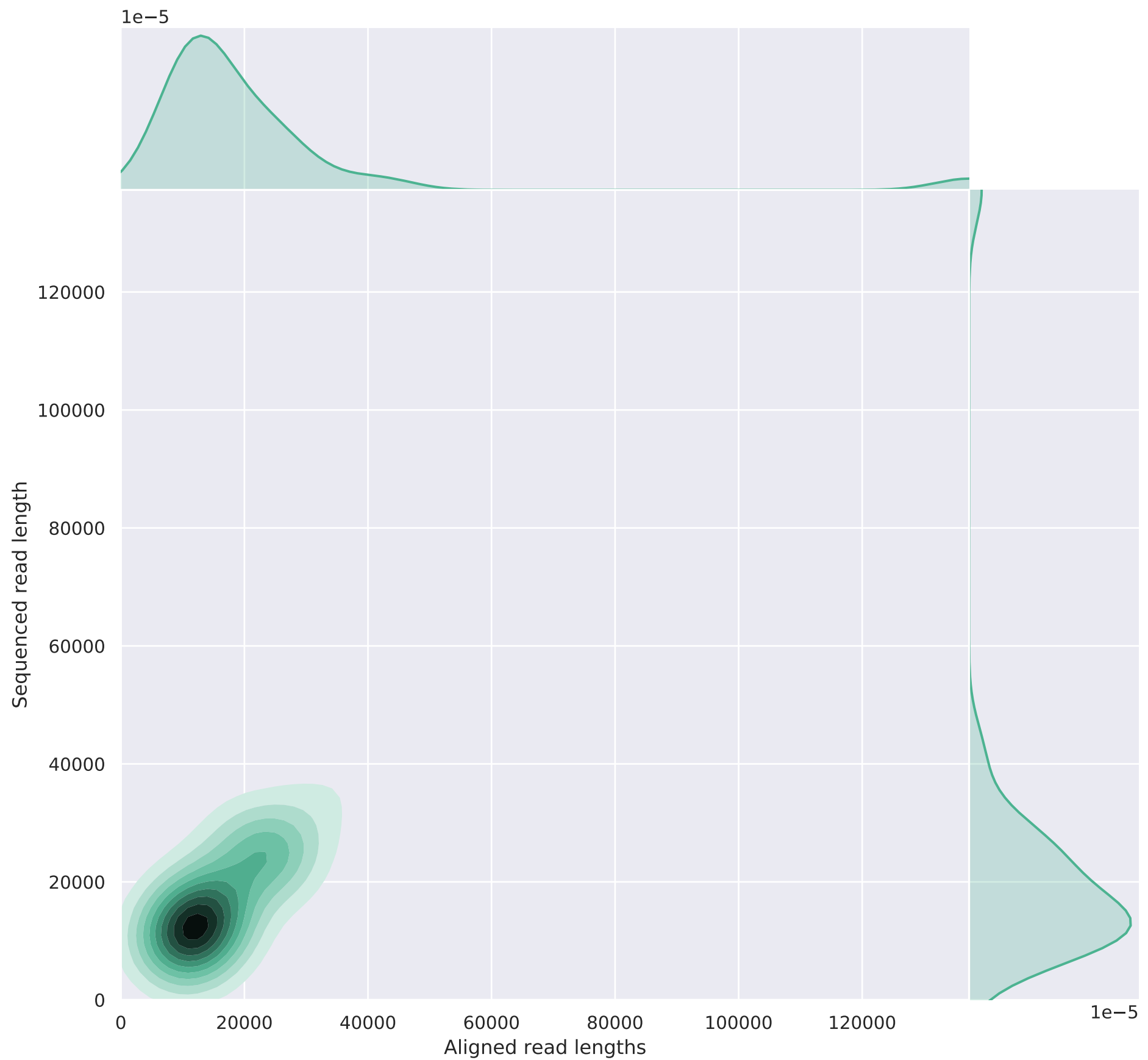

Supplement: Supplementary file 9 [file DataSheet_5.zip › SF1a/ccs999KIR7_18_1.contigs_MN167526_reports/ccs999KIR7_18_1.contigs_MN167526AlignedReadlengthvsSequencedReadLength_kde.pdf]

Weighted Histogram of read lengths after log transformation

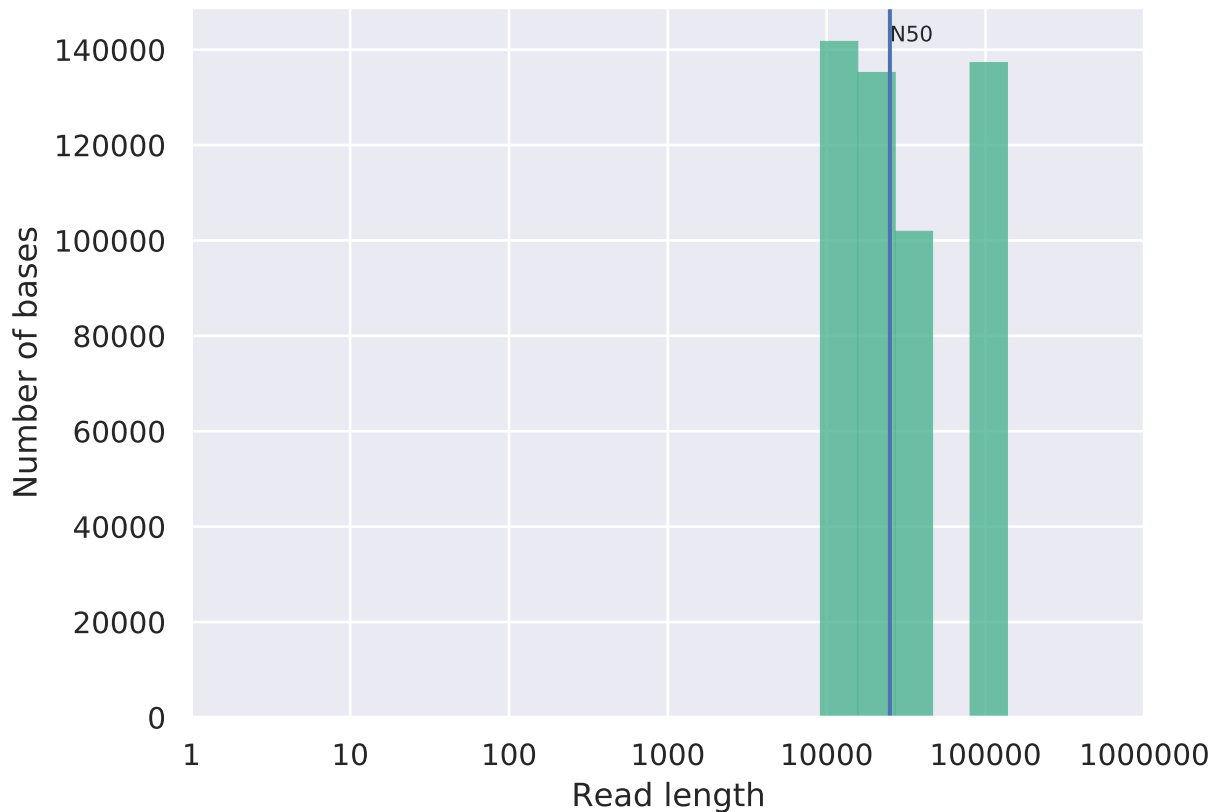

Supplement: Supplementary file 9 [file DataSheet_5.zip › SF1a/ccs999KIR7_18_1.contigs_MN167526_reports/ccs999KIR7_18_1.contigs_MN167526Weighted_LogTransformed_HistogramReadlength.pdf]

# Aligned read lengths vs Sequenced read length plot

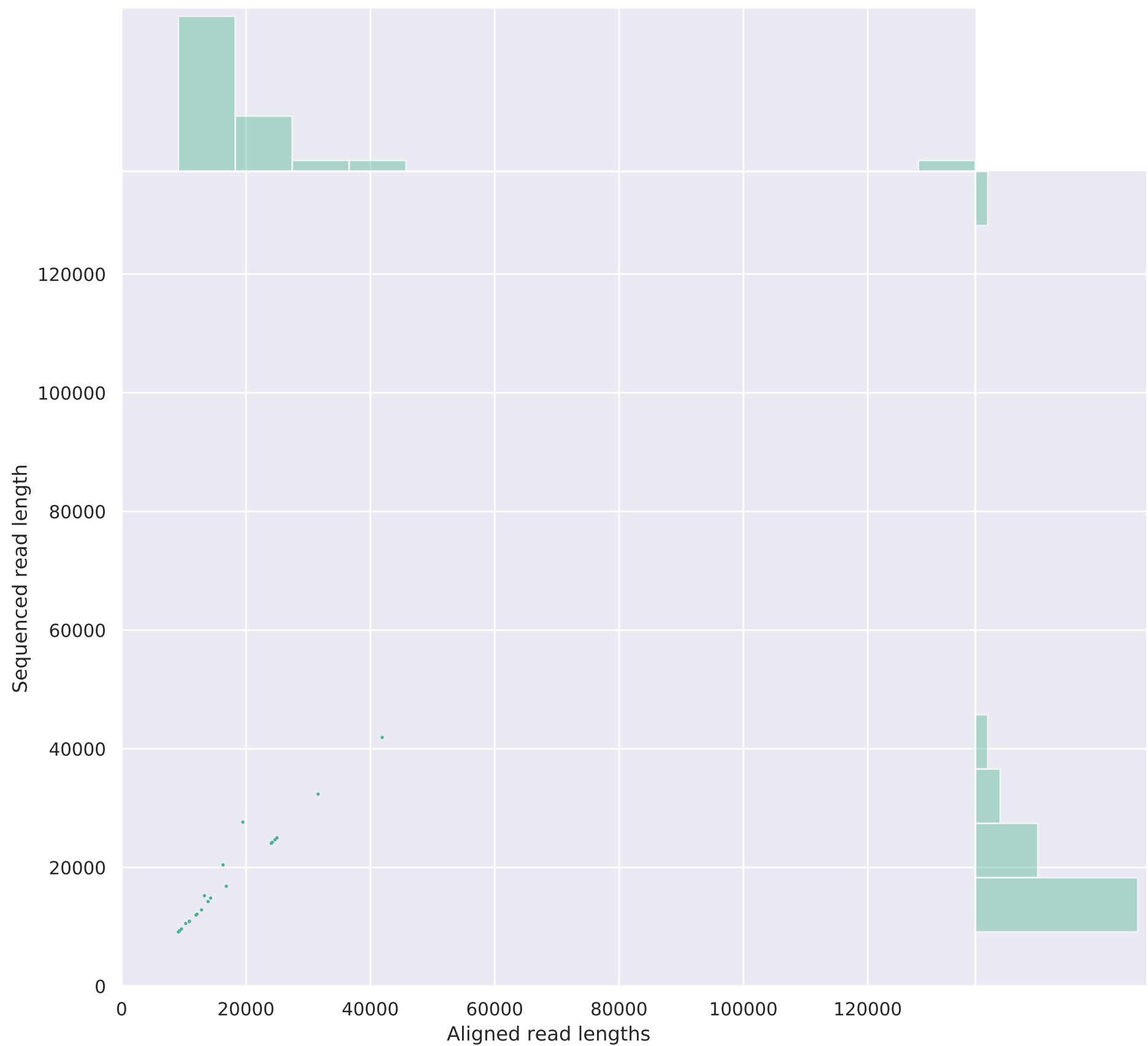

Supplement: Supplementary file 9 [file DataSheet_5.zip › SF1a/ccs999KIR7_18_1.contigs_MN167526_reports/ccs999KIR7_18_1.contigs_MN167526AlignedReadlengthvsSequencedReadLength_dot.pdf]

Histogram of read lengths

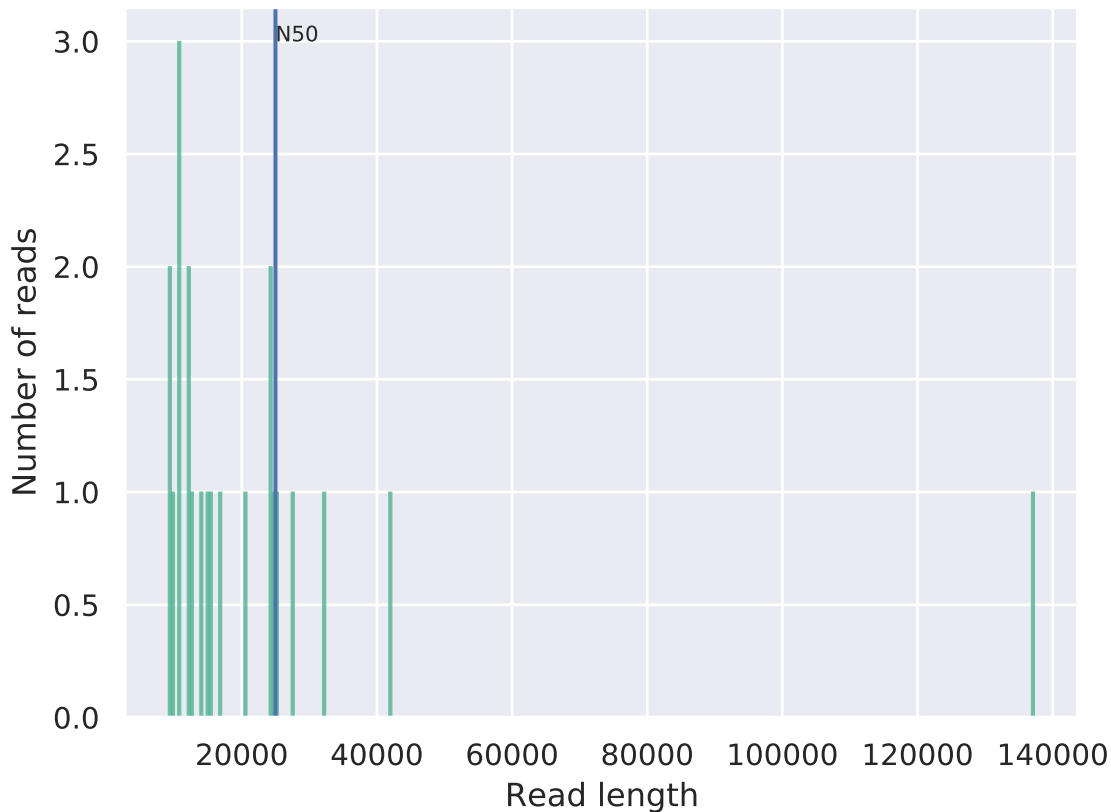

Supplement: Supplementary file 9 [file DataSheet_5.zip › SF1a/ccs999KIR7_18_1.contigs_MN167526_reports/ccs999KIR7_18_1.contigs_MN167526HistogramReadlength.pdf]

# Aligned read length vs Percent identity plot

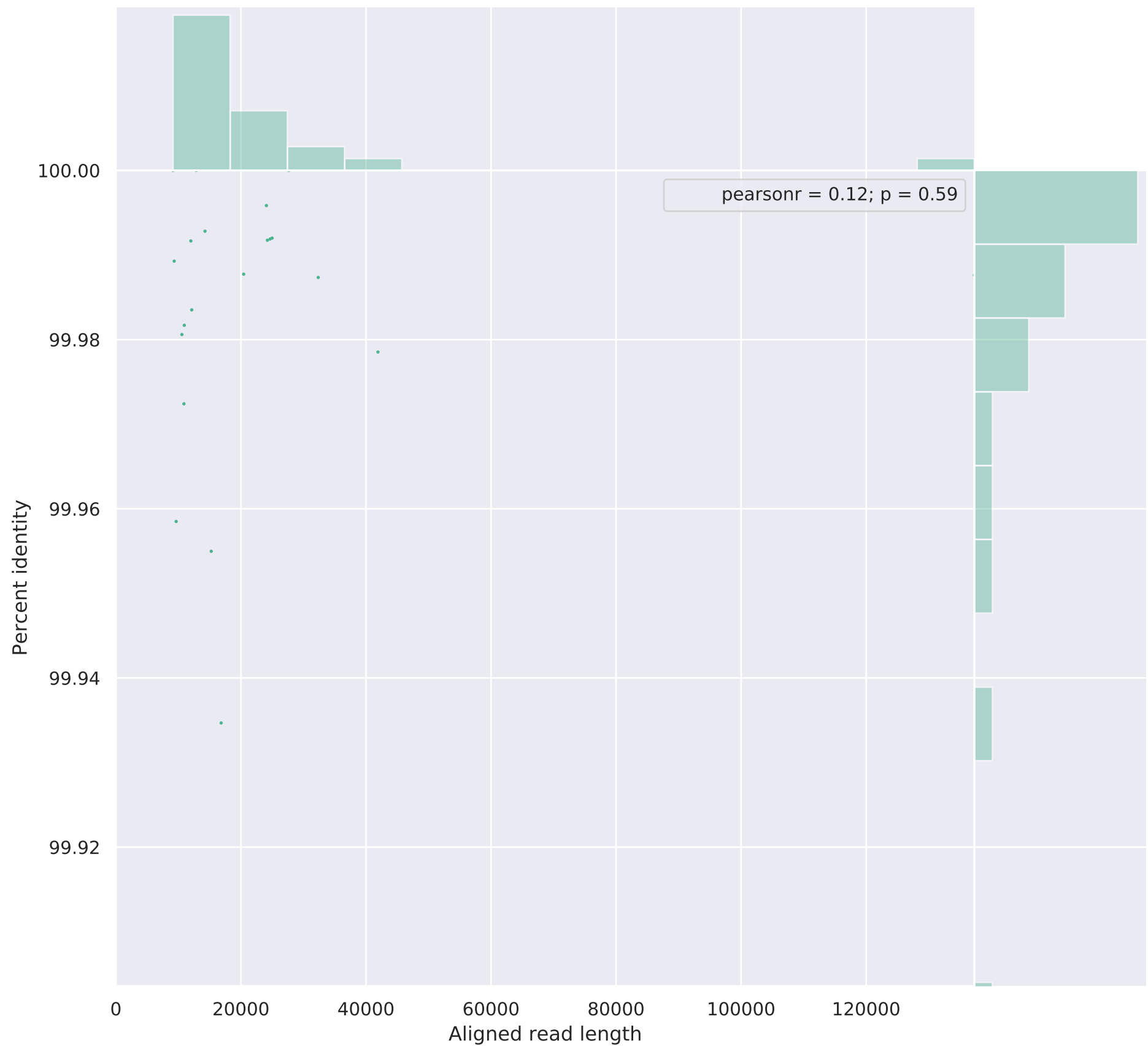

Supplement: Supplementary file 9 [file DataSheet_5.zip › SF1a/ccs999KIR7_18_1.contigs_MN167526_reports/ccs999KIR7_18_1.contigs_MN167526PercentIdentityvsAlignedReadLength_dot.pdf]

# Aligned read length vs Percent identity plot

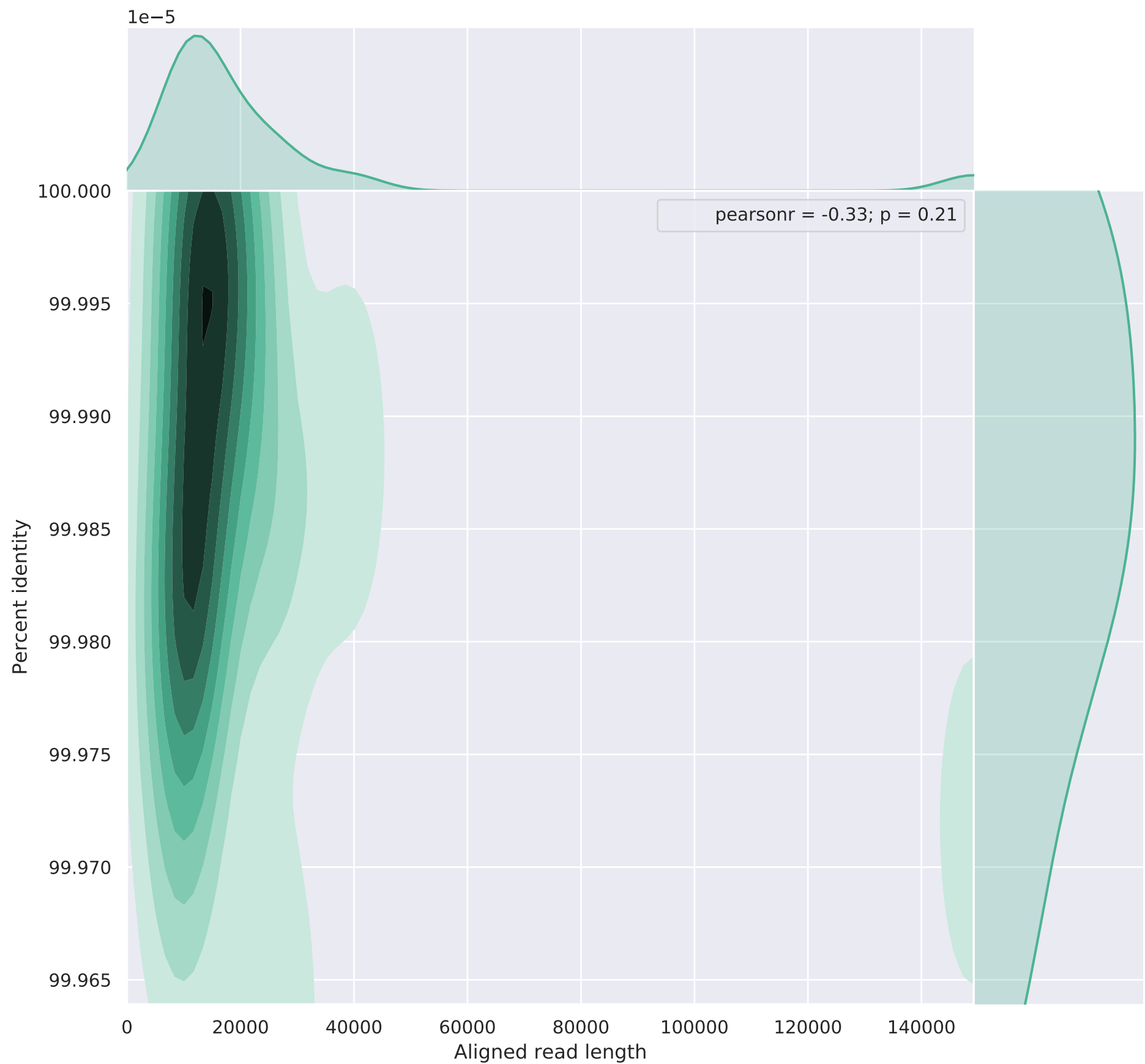

Supplement: Supplementary file 9 [file DataSheet_5.zip › SF1a/ccs999KIR7_18_2.contigs_MN167510_reports/ccs999KIR7_18_2.contigs_MN167510PercentIdentityvsAlignedReadLength_kde.pdf]

# Aligned read lengths vs Sequenced read length plot

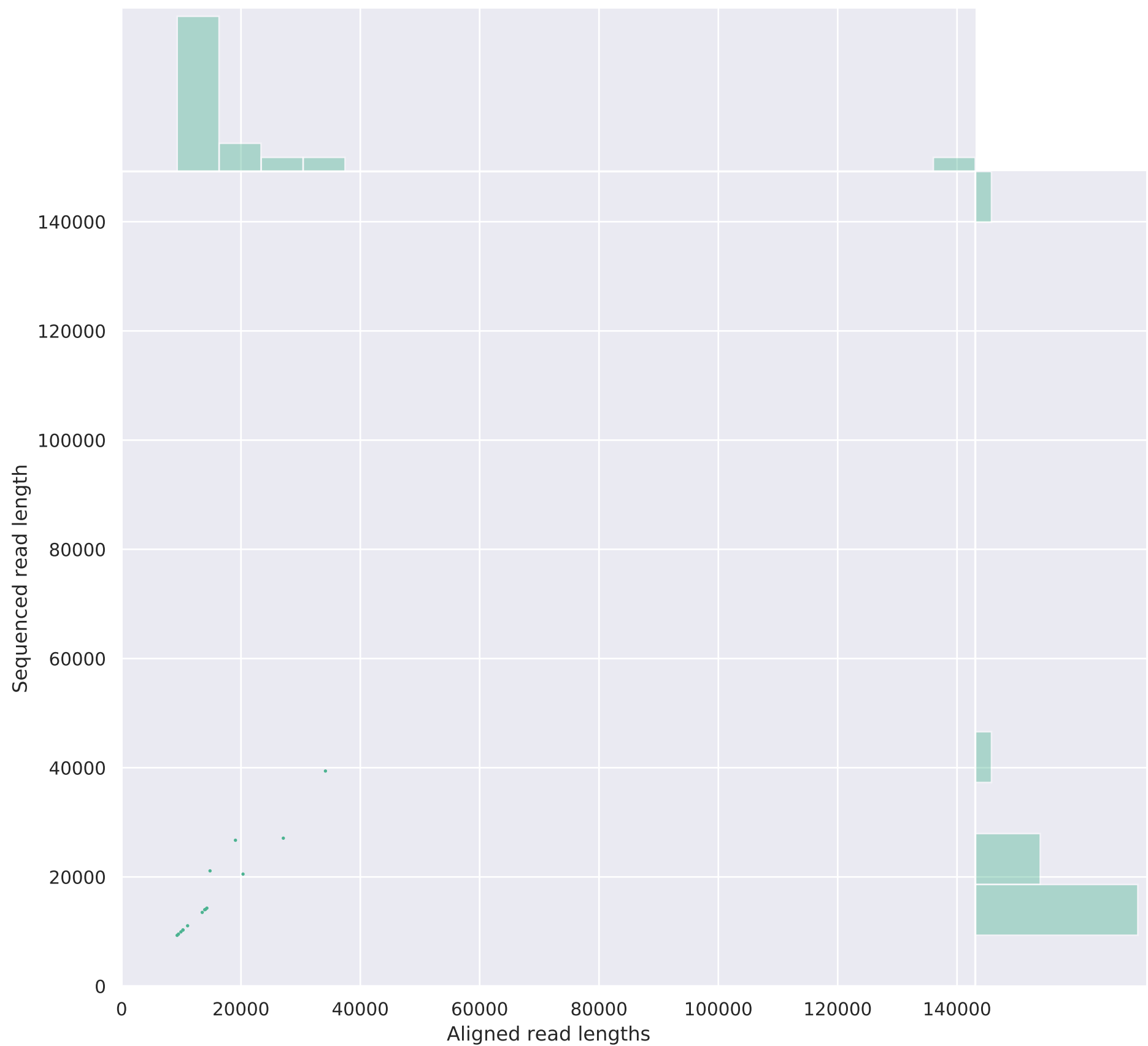

Supplement: Supplementary file 9 [file DataSheet_5.zip › SF1a/ccs999KIR7_18_2.contigs_MN167510_reports/ccs999KIR7_18_2.contigs_MN167510AlignedReadlengthvsSequencedReadLength_dot.pdf]

Weighted Histogram of read lengths after log transformation

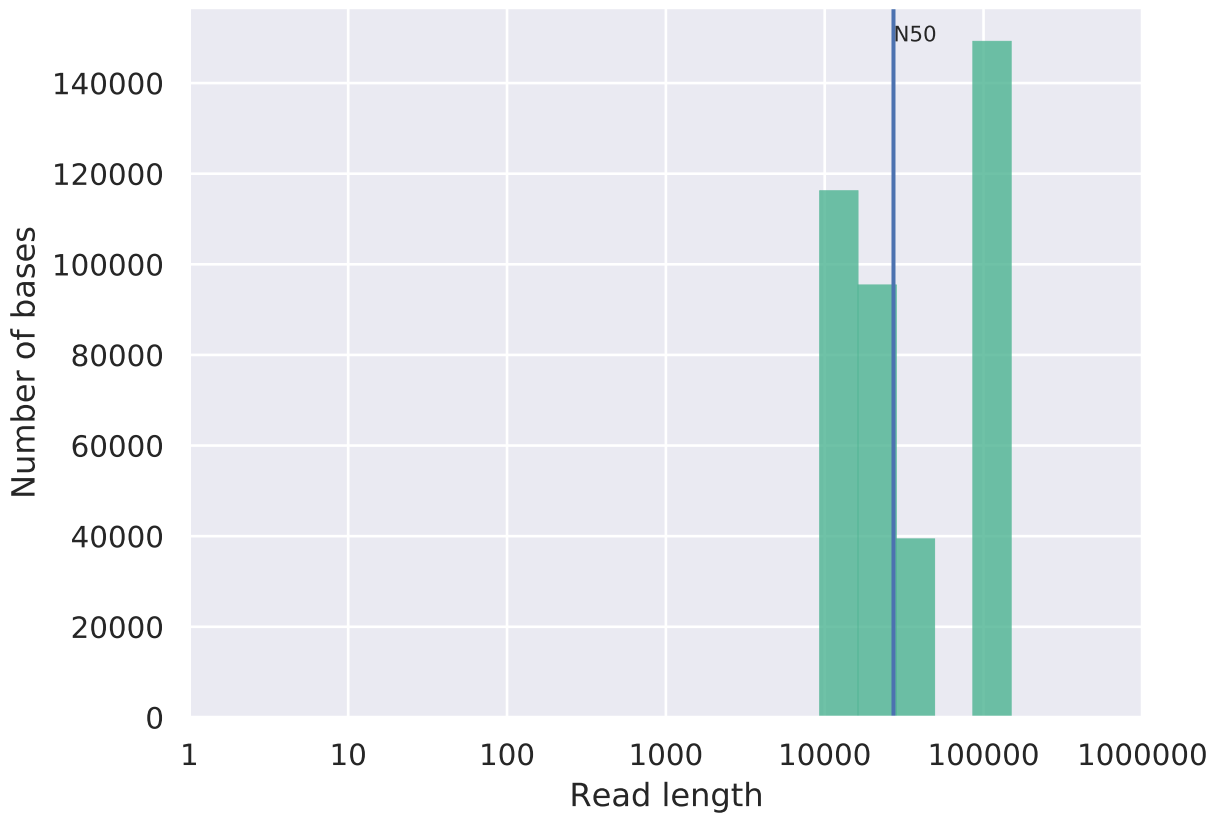

Supplement: Supplementary file 9 [file DataSheet_5.zip › SF1a/ccs999KIR7_18_2.contigs_MN167510_reports/ccs999KIR7_18_2.contigs_MN167510Weighted_LogTransformed_HistogramReadlength.pdf]

Yield by length

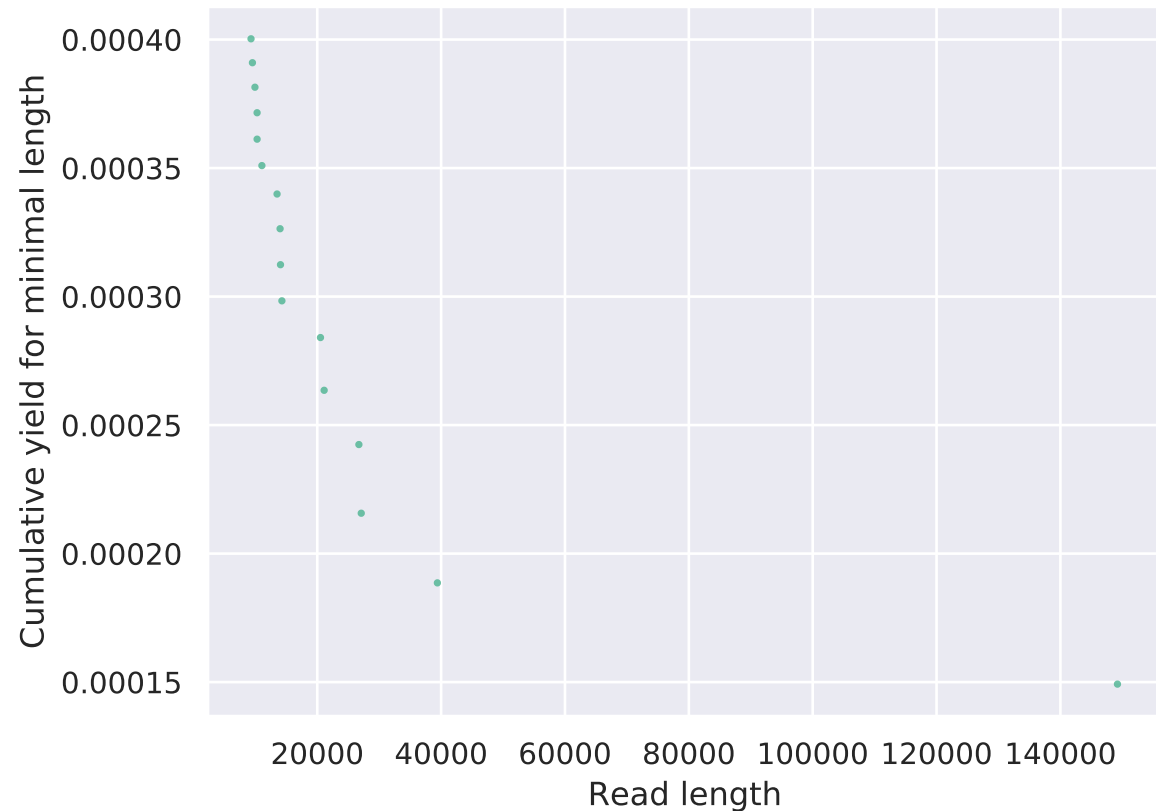

Supplement: Supplementary file 9 [file DataSheet_5.zip › SF1a/ccs999KIR7_18_2.contigs_MN167510_reports/ccs999KIR7_18_2.contigs_MN167510Yield_By_Length.pdf]

Weighted Histogram of read lengths

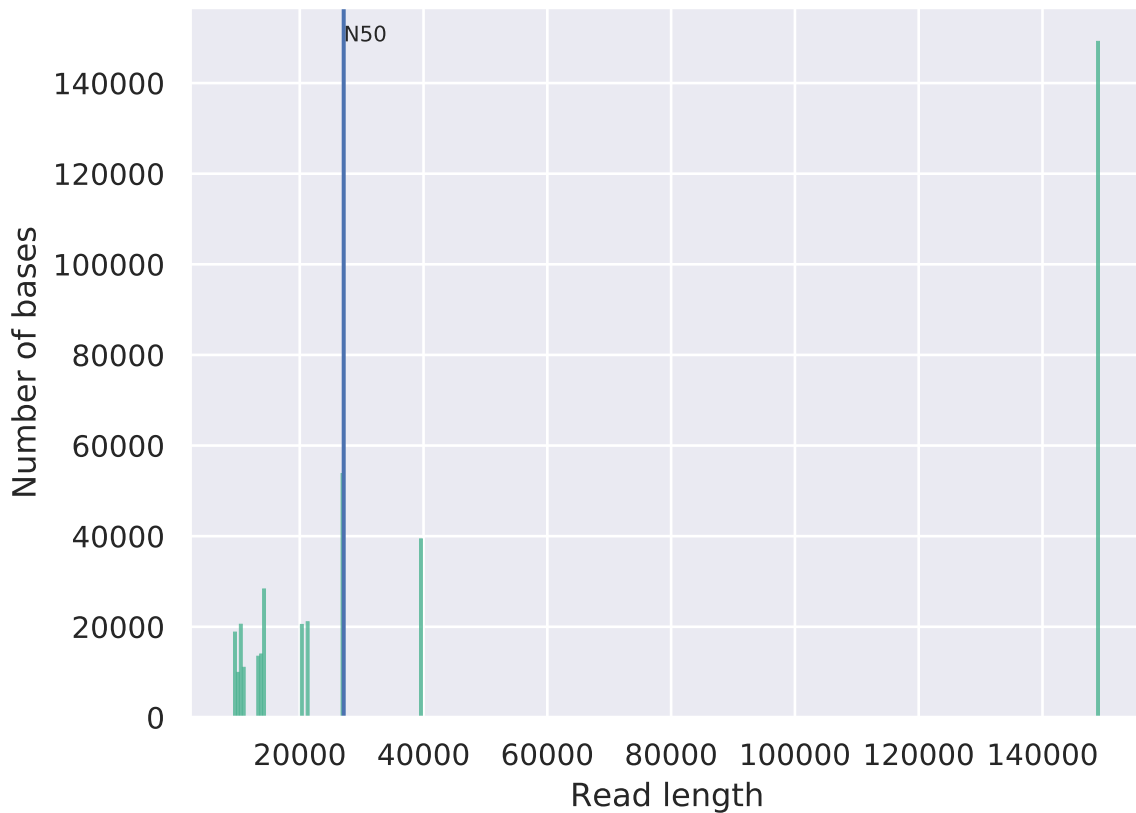

Supplement: Supplementary file 9 [file DataSheet_5.zip › SF1a/ccs999KIR7_18_2.contigs_MN167510_reports/ccs999KIR7_18_2.contigs_MN167510Weighted_HistogramReadlength.pdf]

Histogram of read lengths

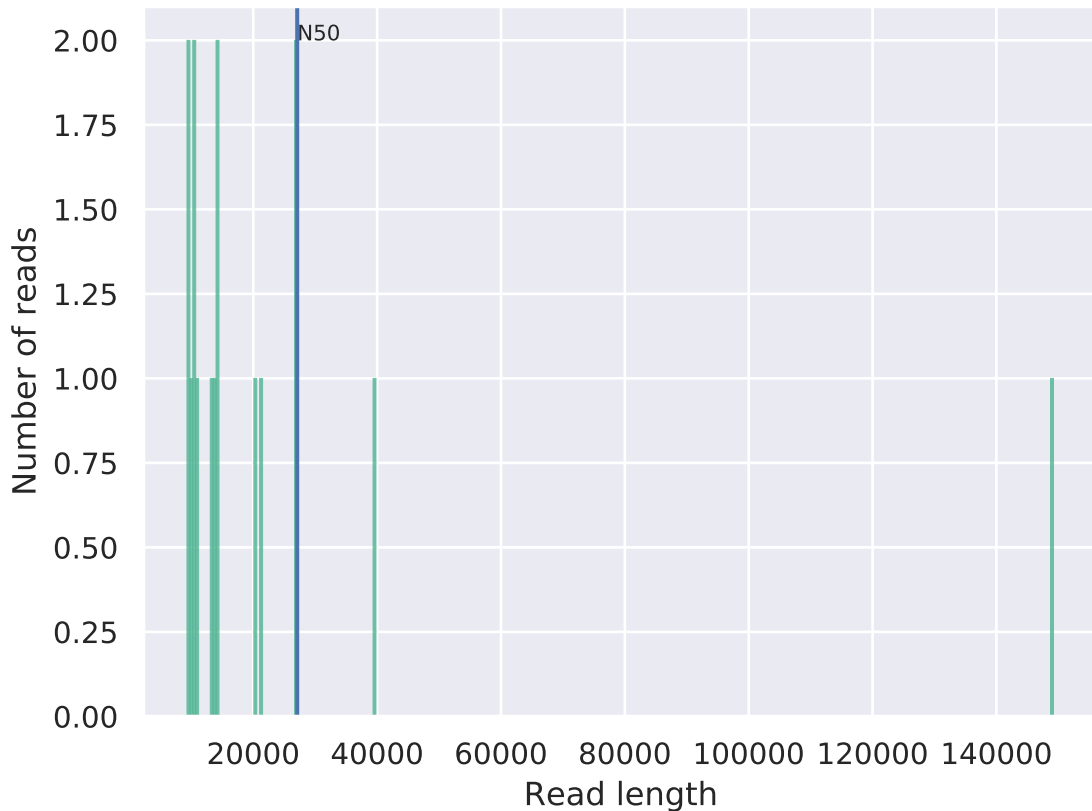

Supplement: Supplementary file 9 [file DataSheet_5.zip › SF1a/ccs999KIR7_18_2.contigs_MN167510_reports/ccs999KIR7_18_2.contigs_MN167510HistogramReadlength.pdf]

Histogram of read lengths after log transformation

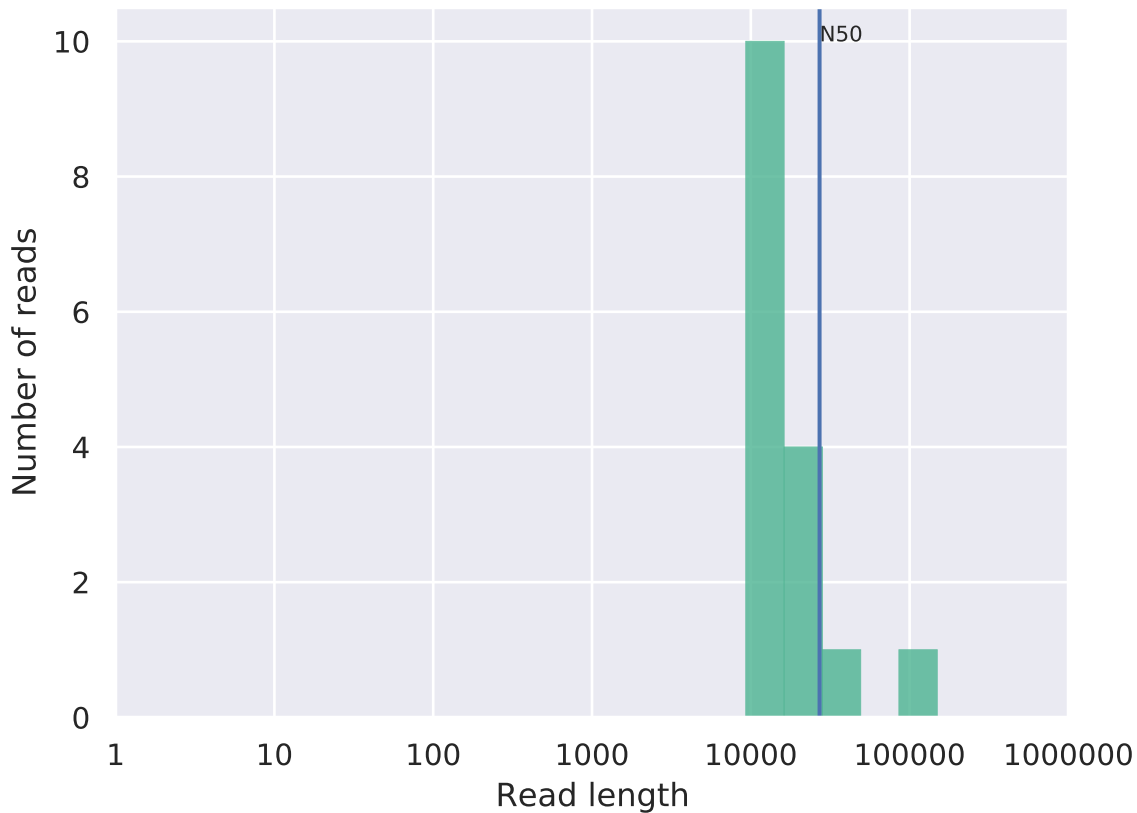

Supplement: Supplementary file 9 [file DataSheet_5.zip › SF1a/ccs999KIR7_18_2.contigs_MN167510_reports/ccs999KIR7_18_2.contigs_MN167510LogTransformed_HistogramReadlength.pdf]

# Aligned read lengths vs Sequenced read length plot

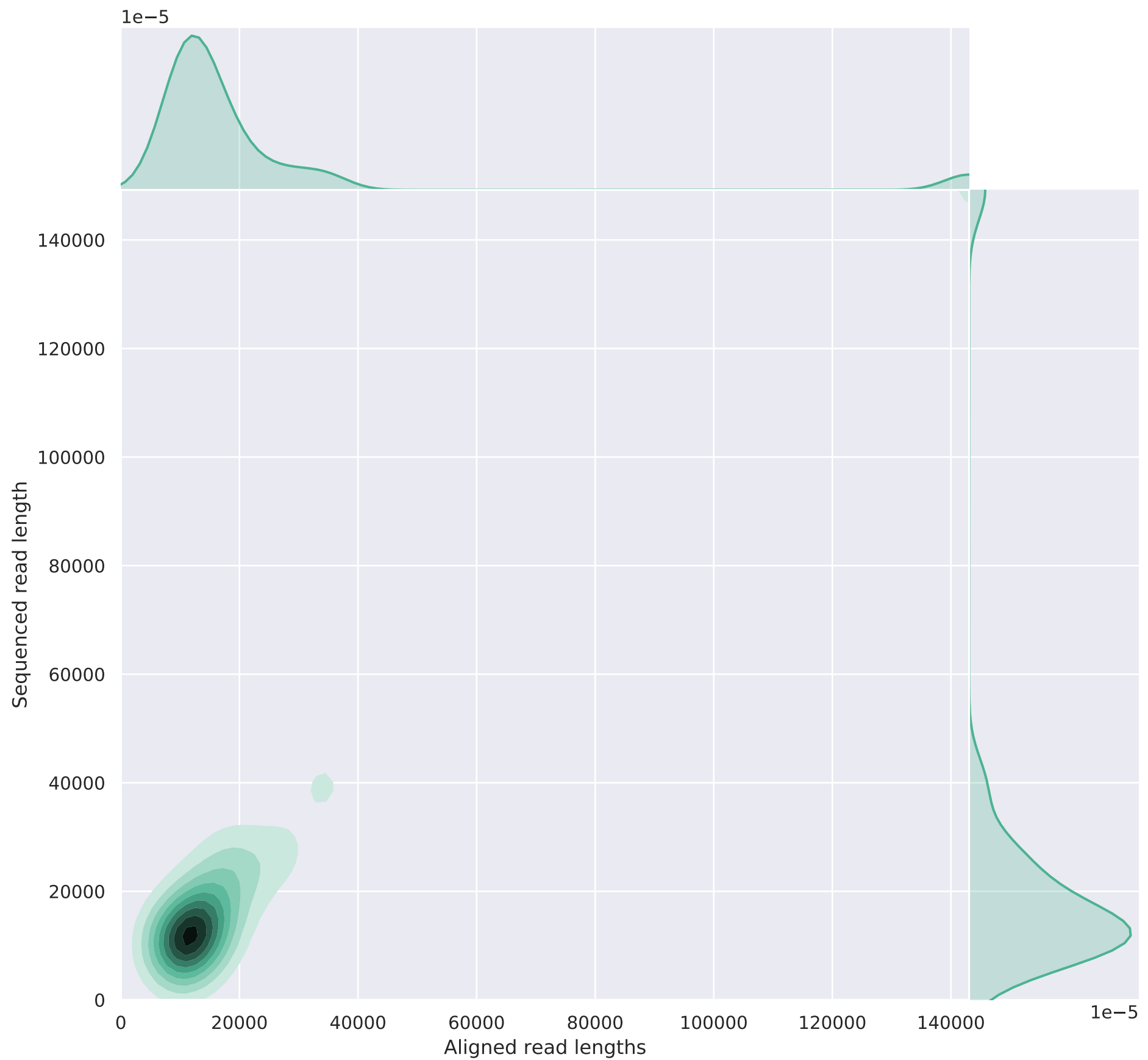

Supplement: Supplementary file 9 [file DataSheet_5.zip › SF1a/ccs999KIR7_18_2.contigs_MN167510_reports/ccs999KIR7_18_2.contigs_MN167510AlignedReadlengthvsSequencedReadLength_kde.pdf]

# Aligned read length vs Percent identity plot

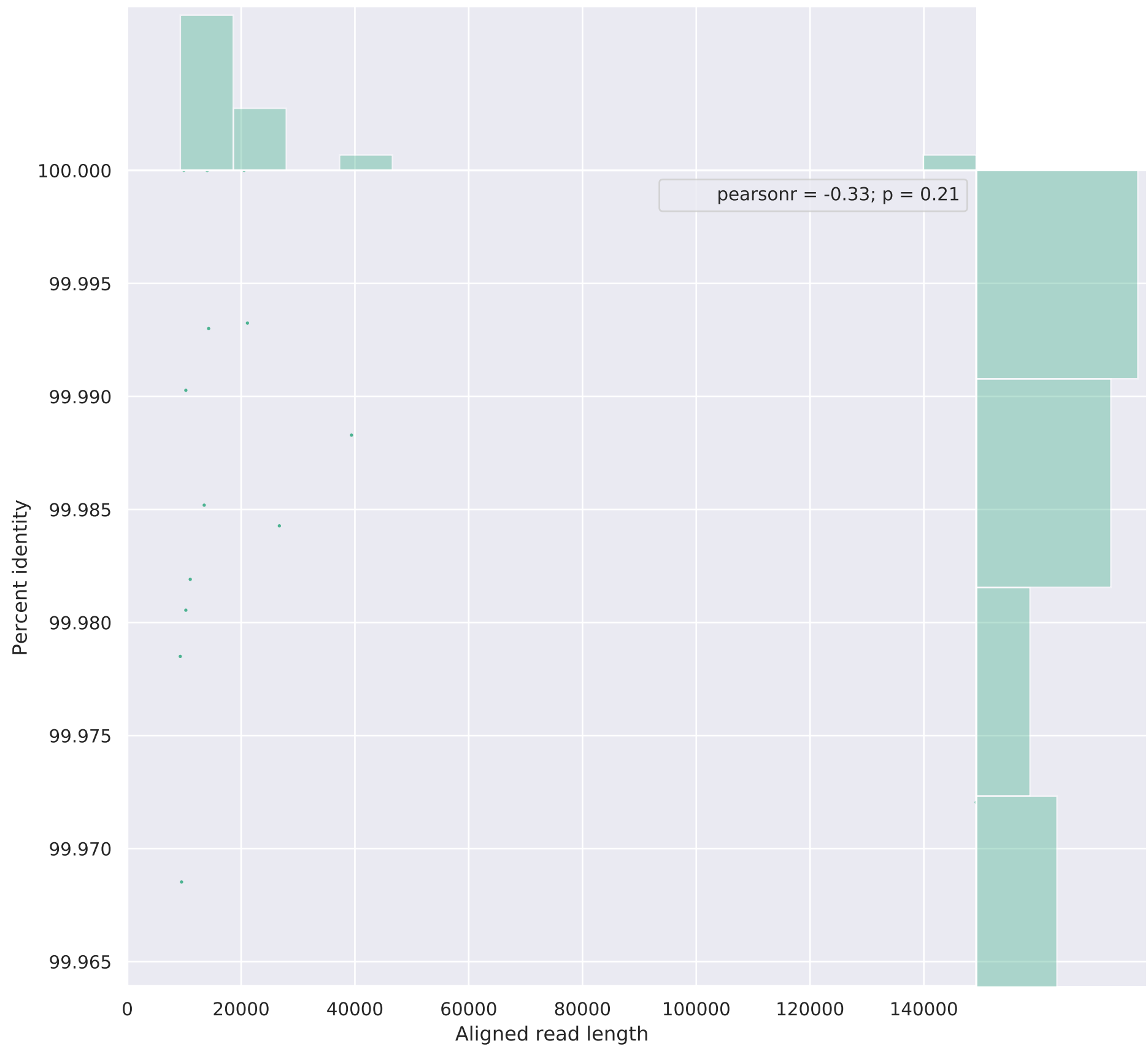

Supplement: Supplementary file 9 [file DataSheet_5.zip › SF1a/ccs999KIR7_18_2.contigs_MN167510_reports/ccs999KIR7_18_2.contigs_MN167510PercentIdentityvsAlignedReadLength_dot.pdf]
