# Supplementary material for: Efficient Sequencing, Assembly, and Annotation of Human KIR Haplotypes
Source: Front Immunol. 2020 Oct 9;11:582927. doi: 10.3389/fimmu.2020.582927 (PMC7581912; doi:10.3389/fimmu.2020.582927)

# Qualimap Analysis Results

*BAM QC analysis*

*Generated by Qualimap v.2.2.1*

*2020/04/21 17:44:06*

# 1. Input data & parameters

## 1.1. QualiMap command line

| |
|---|
| qualimap bamqc -bam ccs999KIR7_18_2.contigs_MN167507_sorted.bam -nw 400 -hm 3 |

## 1.2. Alignment

| | |
|---|---|
| Command line: | bwa mem -k1800 -W9000 -r10 -A1 -B100 -O40 -E40 -L50 -t7 MN167507.fasta ccs999KIR7_18_2.contigs.fasta.gz |
| Draw chromosome limits: | no |
| Analyze overlapping paired-end reads: | no |
| Program: | bwa (0.7.17-r1188) |
| Analysis date: | Tue Apr 21 17:44:05 GMT 2020 |
| Size of a homopolymer: | 3 |
| Skip duplicate alignments: | no |
| Number of windows: | 400 |
| BAM file: | ccs999KIR7_18_2.contigs_MN167507_sorted.bam |

# 2. Summary

## 2.1. Globals

| | |
|---|---|
| Reference size | 110,582 |
| Number of reads | 7 |
| Mapped reads | 7 / 100% |
| Supplementary alignments | 2 / 28.57% |
| Unmapped reads | 0 / 0% |
| Mapped paired reads | 0 / 0% |
| Read min/max/mean length | 2,696 / 87,347 / 26,621.14 |
| Duplicated reads (estimated) | 0 / 0% |
| Duplication rate | 0% |
| Clipped reads | 7 / 100% |

## 2.2. ACGT Content

| | |
|---|---|
| Number/percentage of A's | 38,438 / 27.18% |
| Number/percentage of C's | 31,513 / 22.28% |
| Number/percentage of T's | 36,587 / 25.87% |
| Number/percentage of G's | 34,889 / 24.67% |
| Number/percentage of N's | 0 / 0% |
| GC Percentage | 46.95% |

## 2.3. Coverage

| | |
|---|---|
| Mean | 1.2792 |
| Standard Deviation | 0.7824 |

## 2.4. Mapping Quality

| | |
|---|---|
| Mean Mapping Quality | 52.04 |

## 2.5. Mismatches and indels

| | |
|---|---|
| General error rate | 0.18% |
| Mismatches | 210 |
| Insertions | 6 |
| Mapped reads with at least one insertion | 14.29% |
| Deletions | 18 |
| Mapped reads with at least one deletion | 71.43% |
| Homopolymer indels | 41.67% |

## 2.6. Chromosome stats

| **Name** | **Length** | **Mapped bases** | **Mean coverage** | **Standard deviation** |
|---|---|---|---|---|
| MN167507 | 110582 | 141454 | 1.2792 | 0.7824 |

# 3. Results : Coverage across reference

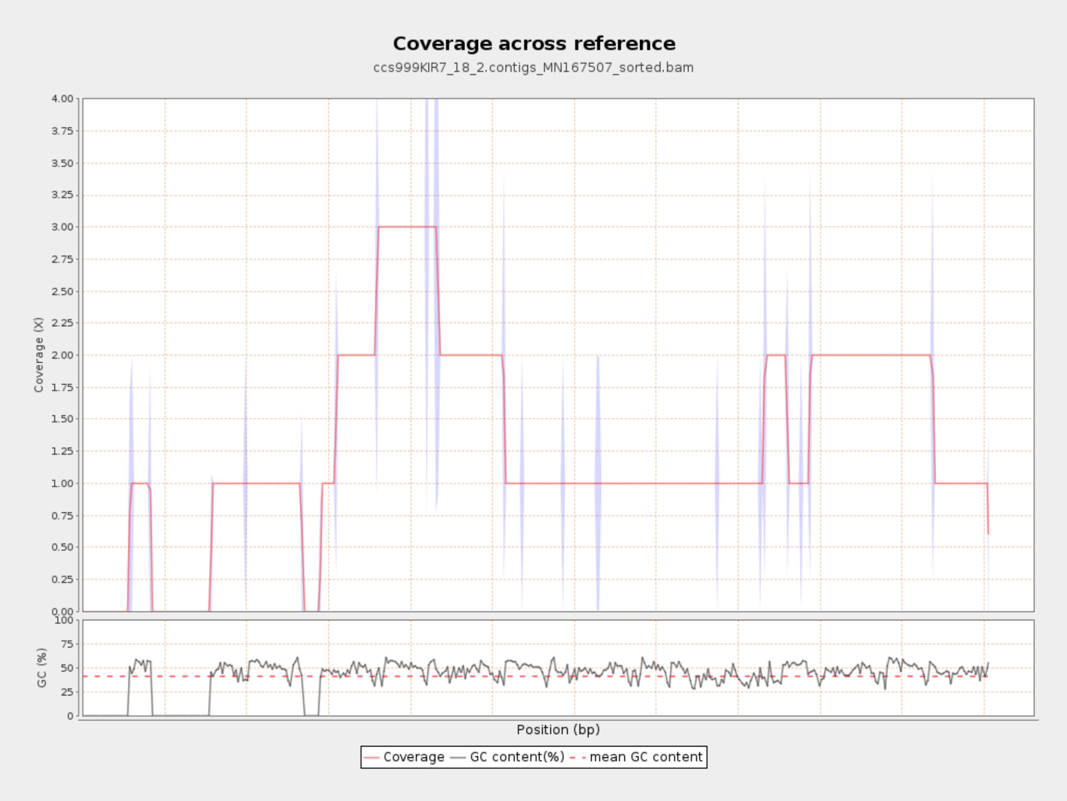

# 4. Results : Coverage Histogram

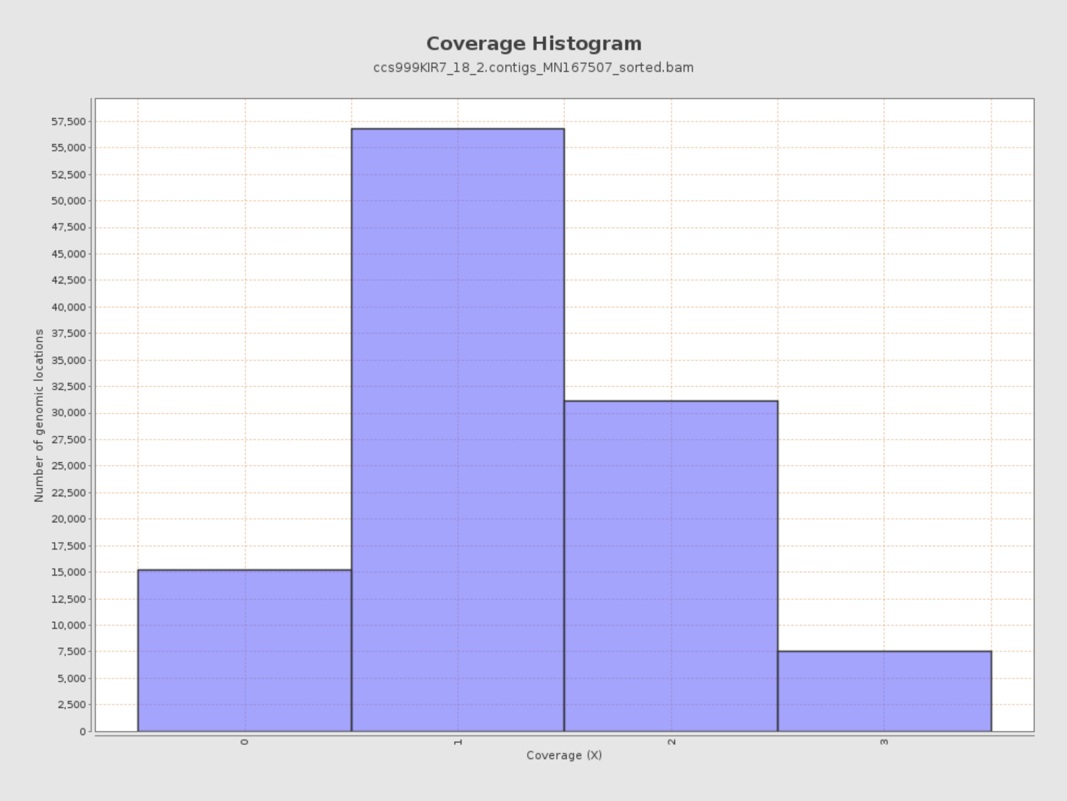

# 5. Results : Coverage Histogram (0-50X)

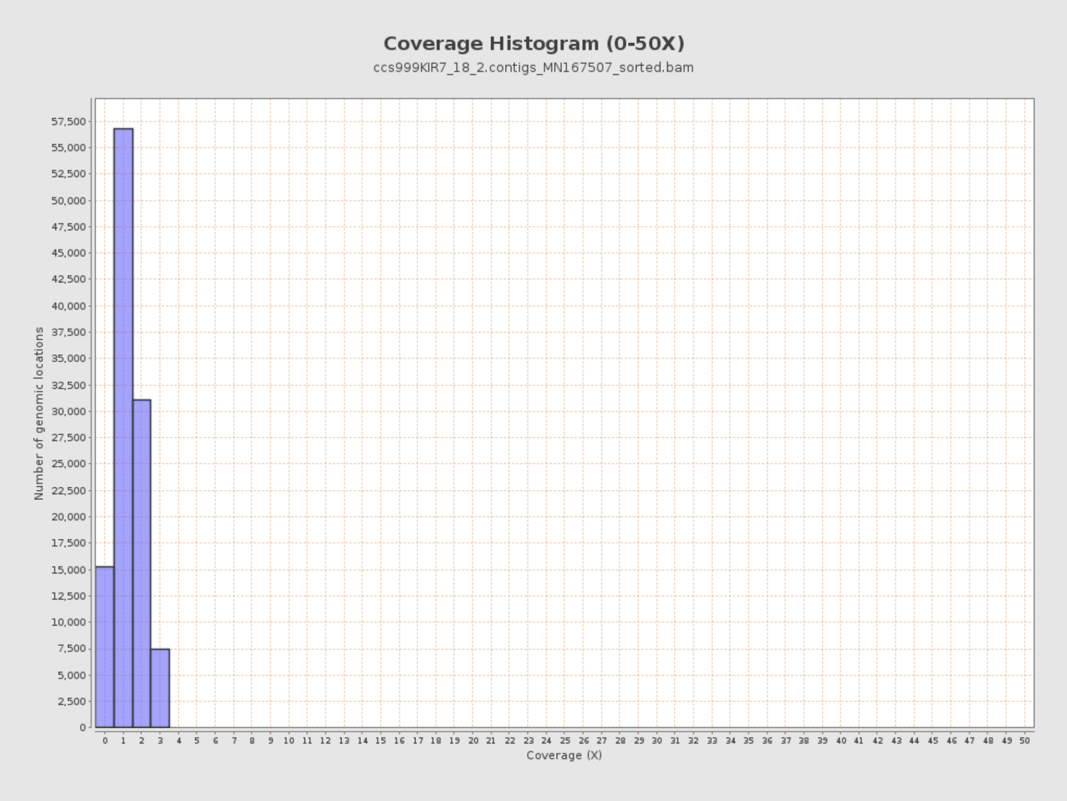

# 6. Results : Genome Fraction Coverage

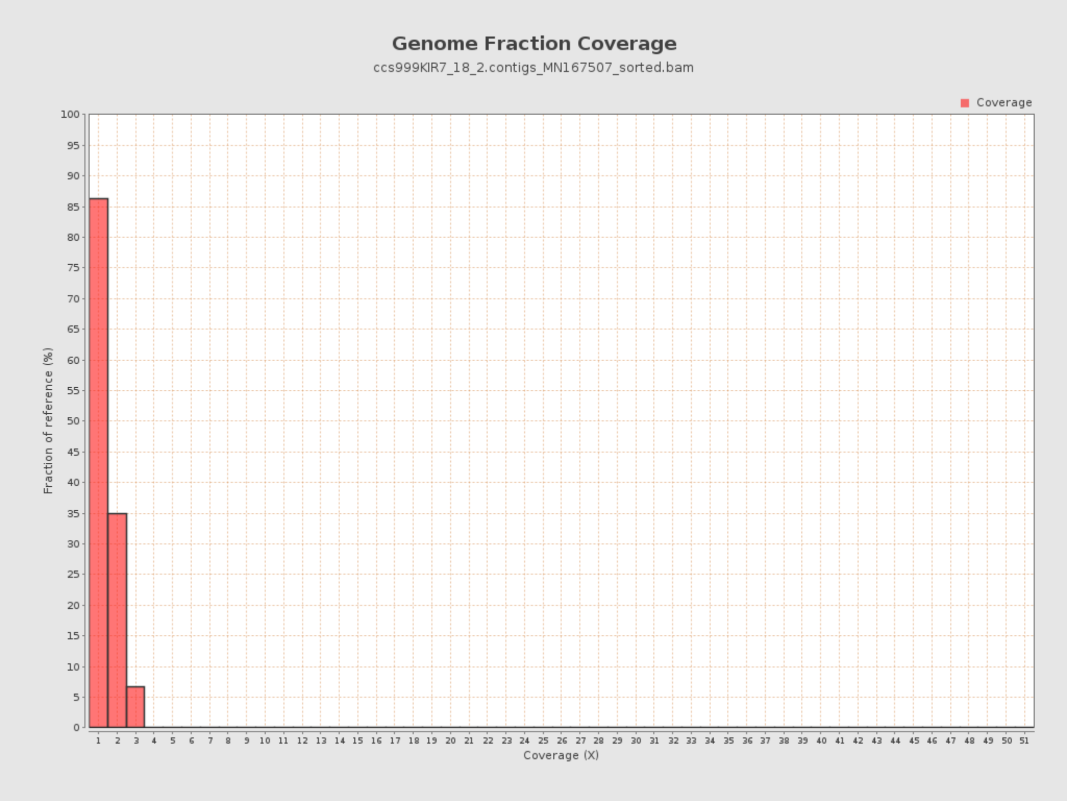

# 7. Results : Duplication Rate Histogram

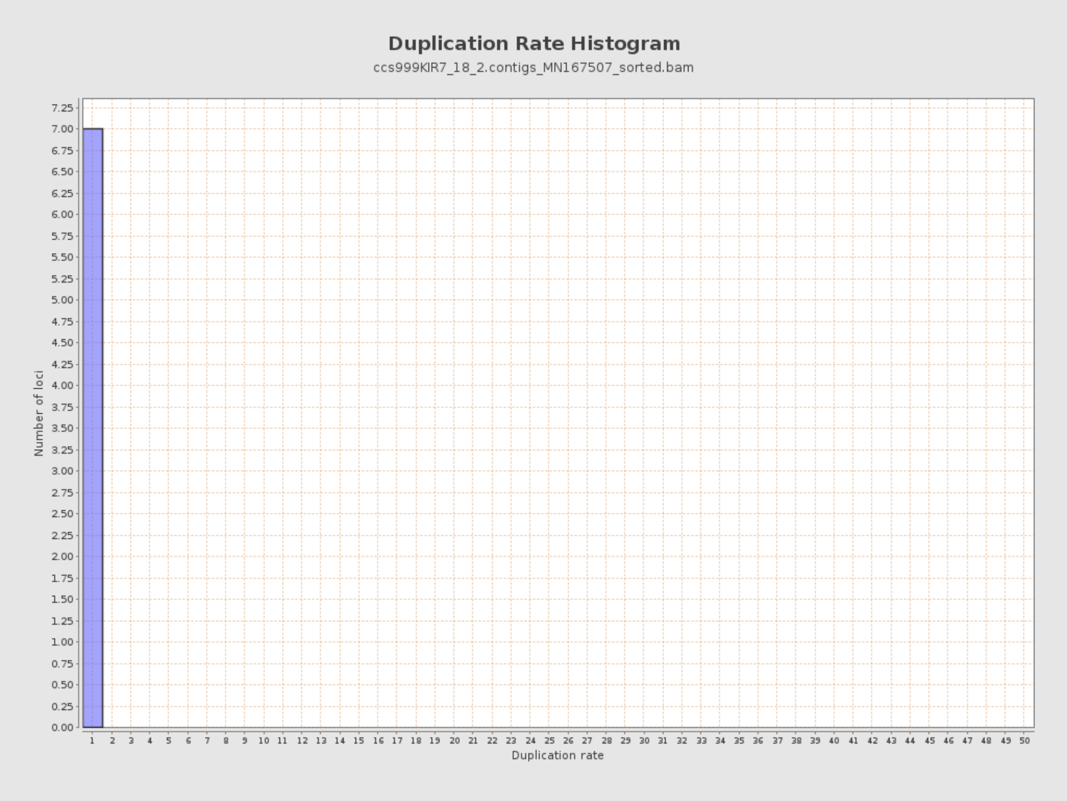

# 8. Results : Mapped Reads Nucleotide Content

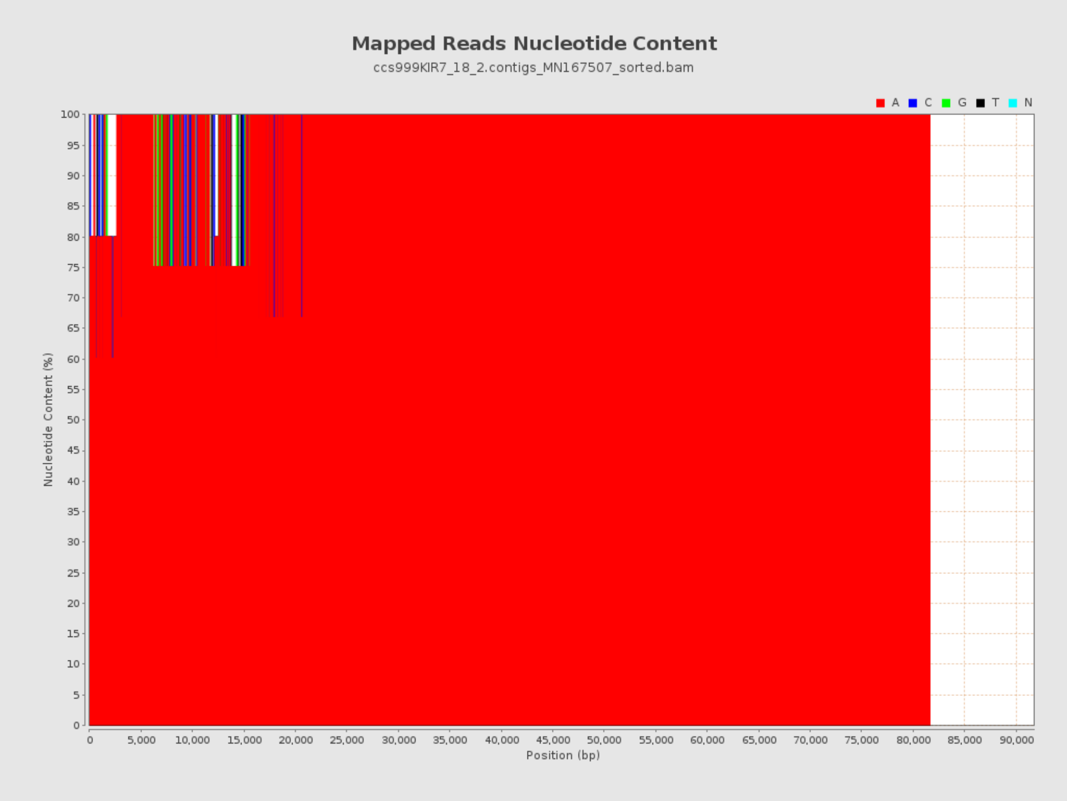

# 9. Results : Mapped Reads GC-content Distribution

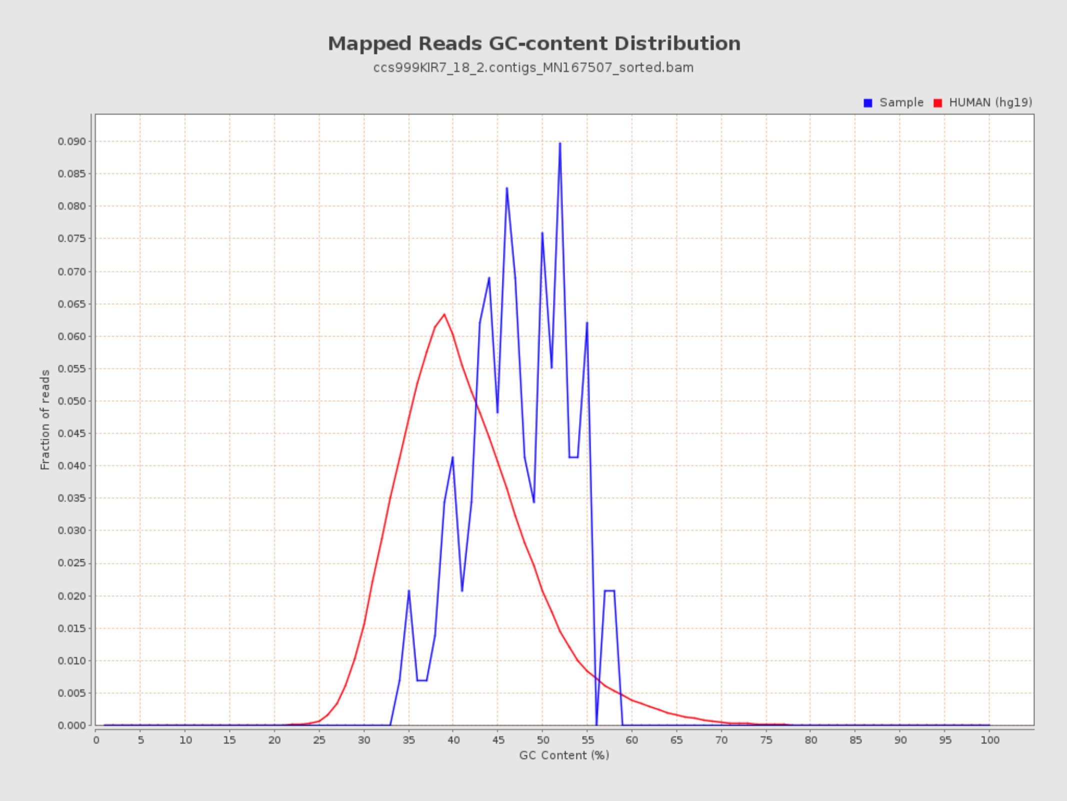

# 10. Results : Mapped Reads Clipping Profile

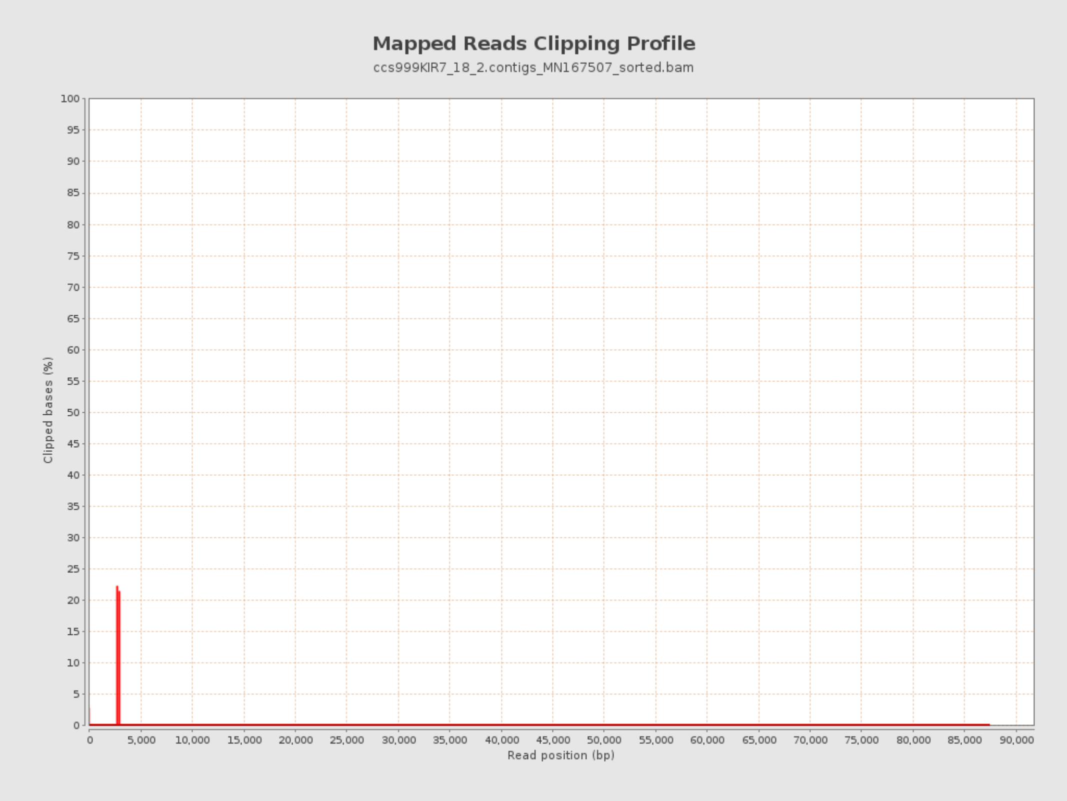

# 11. Results : Homopolymer Indels

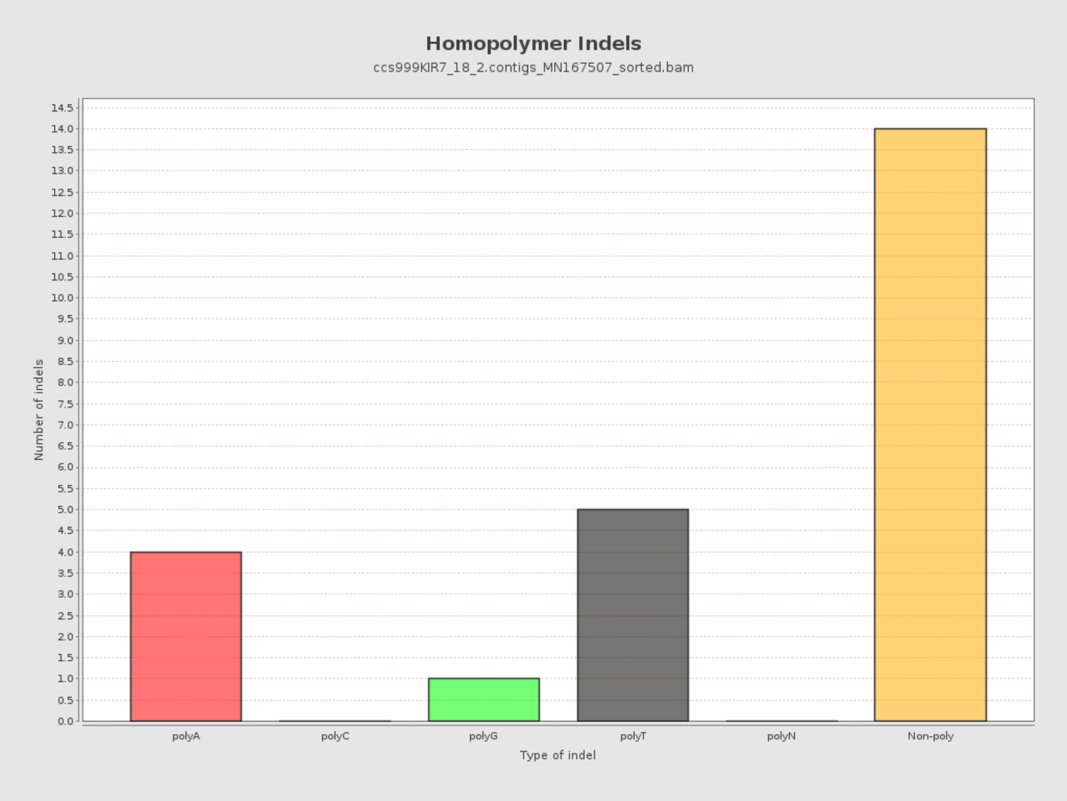

# 12. Results : Mapping Quality Across Reference

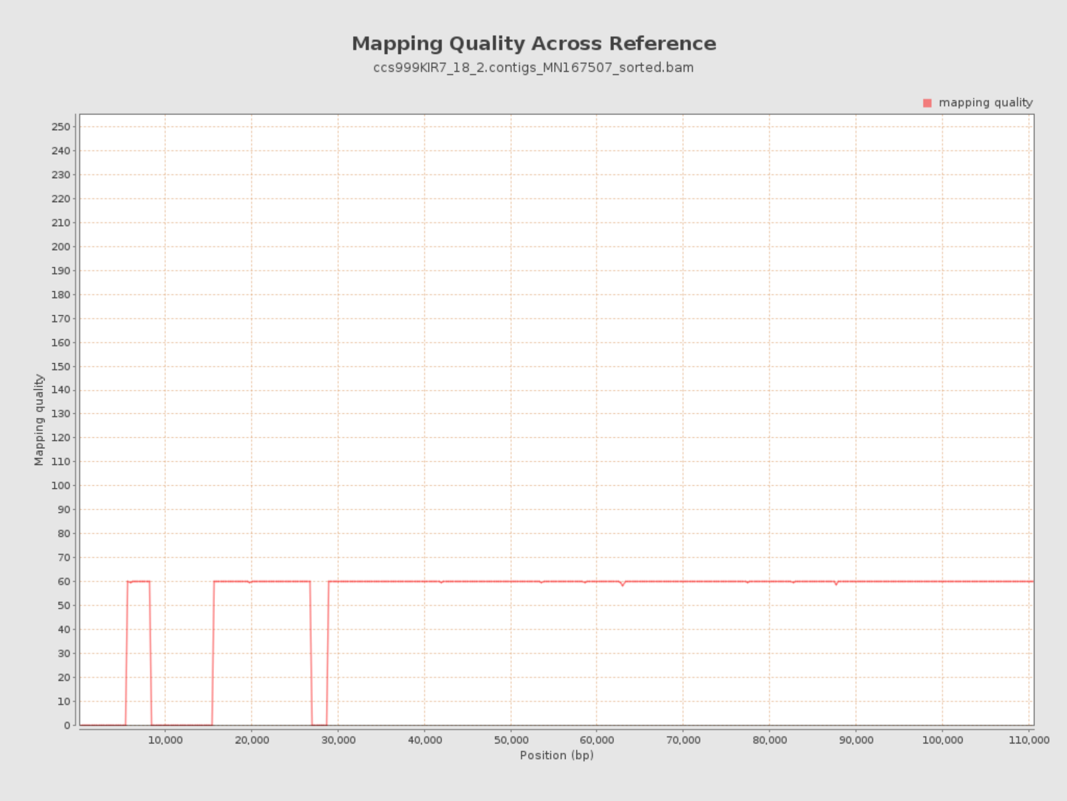

# 13. Results : Mapping Quality Histogram

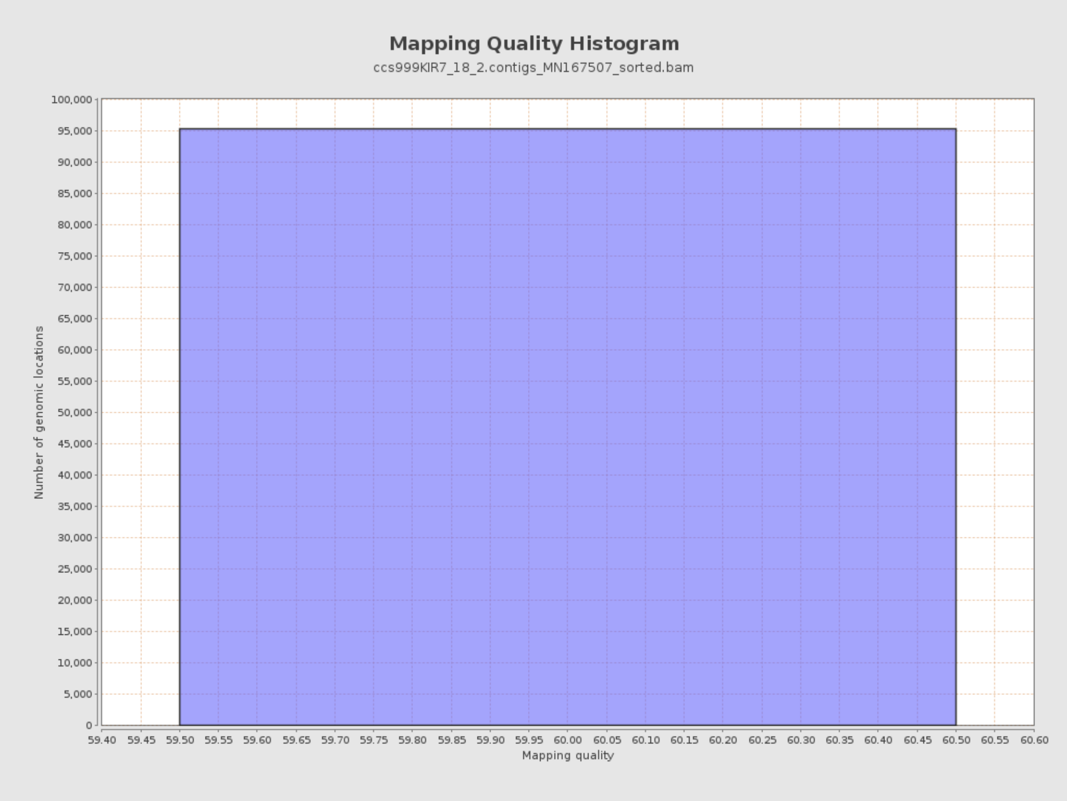

Supplement: Supplementary file 9 [file DataSheet_5.zip › SF1a/ccs999KIR7_18_2.contigs_MN167507_reports/ccs999KIR7_18_2.contigs_MN167507_qualimap.pdf]
