# Supplementary material for: Efficient Sequencing, Assembly, and Annotation of Human KIR Haplotypes
Source: Front Immunol. 2020 Oct 9;11:582927. doi: 10.3389/fimmu.2020.582927 (PMC7581912; doi:10.3389/fimmu.2020.582927)

Qualimap Analysis Results

BAM QC analysis

Generated by Qualimap v.2.2.1

2020/04/21 17:44:35

1. Input data & parameters

1.1. QualiMap command line

qualimap bamqc -bam ccs999KIR7_18_2.contigs_MN167510_sorted.bam -nw 400 -hm 3

1.2. Alignment

Command line:	bwa mem -k1800 -W9000 -r10 -A1 -B100 -O40 -E40 -L50 -t7 MN167510.fasta ccs999KIR7_18_2.contigs.fasta.gz
Draw chromosome limits:	no
Analyze overlapping paired-end reads:	no
Program:	bwa (0.7.17-r1188)
Analysis date:	Tue Apr 21 17:44:34 GMT 2020
Size of a homopolymer:	3
Skip duplicate alignments:	no
Number of windows:	400
BAM file:	ccs999KIR7_18_2.contigs_MN167510_sorted.bam

2. Summary

2.1. Globals

Reference size	199,689
Number of reads	16
Mapped reads	16 / 100%
Unmapped reads	0 / 0%
Mapped paired reads	0 / 0%
Read min/max/mean length	9,301 / 149,211 / 25,017.88
Duplicated reads (estimated)	0 / 0%
Duplication rate	0%
Clipped reads	7 / 43.75%

2.2. ACGT Content

Number/percentage of A's	100,858 / 26.92%
Number/percentage of C's	84,518 / 22.56%
Number/percentage of T's	96,262 / 25.69%
Number/percentage of G's	93,055 / 24.83%
Number/percentage of N's	0 / 0%
GC Percentage	47.39%

2.3. Coverage

Mean	1.8766
Standard Deviation	0.9091

2.4. Mapping Quality

Mean Mapping Quality	57.89

2.5. Mismatches and indels

General error rate	0.02%
Mismatches	52
Insertions	16
Mapped reads with at least one insertion	18.75%
Deletions	29
Mapped reads with at least one deletion	56.25%
Homopolymer indels	84.44%

2.6. Chromosome stats

**Name**	**Length**	**Mapped bases**	**Mean coverage**	**Standard deviation**
MN167510	199689	374727	1.8766	0.9091

3. Results : Coverage across reference

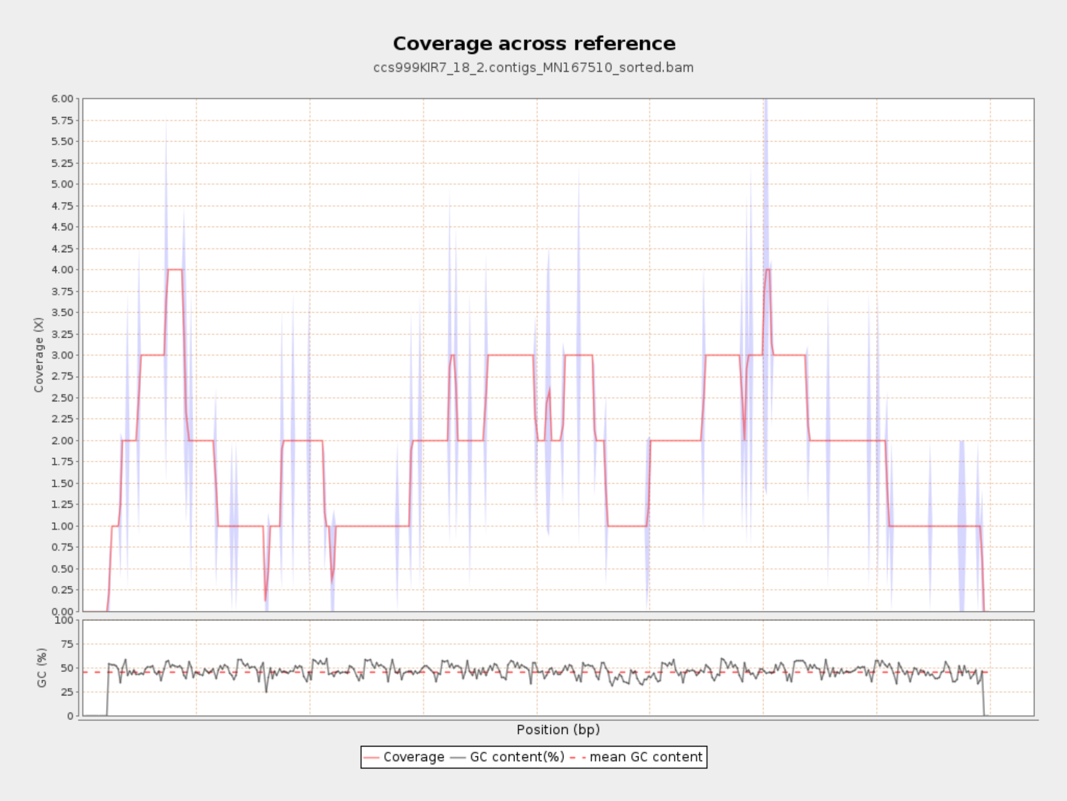

4. Results : Coverage Histogram

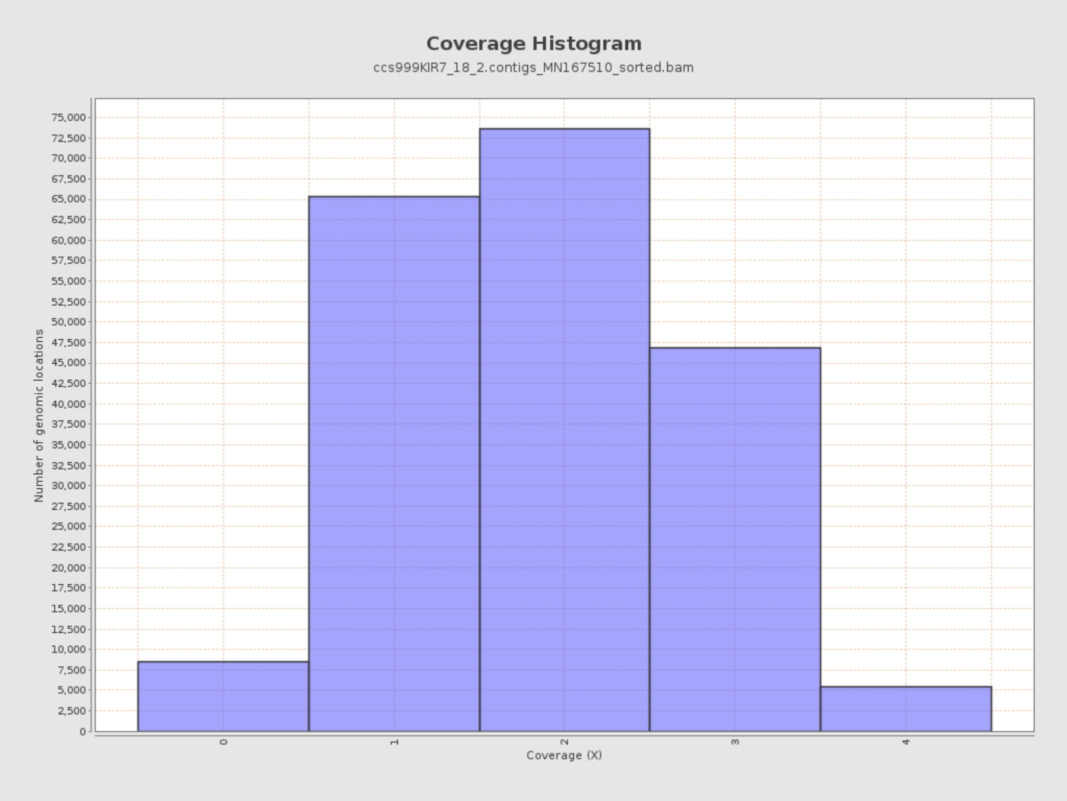

5. Results : Coverage Histogram (0-50X)

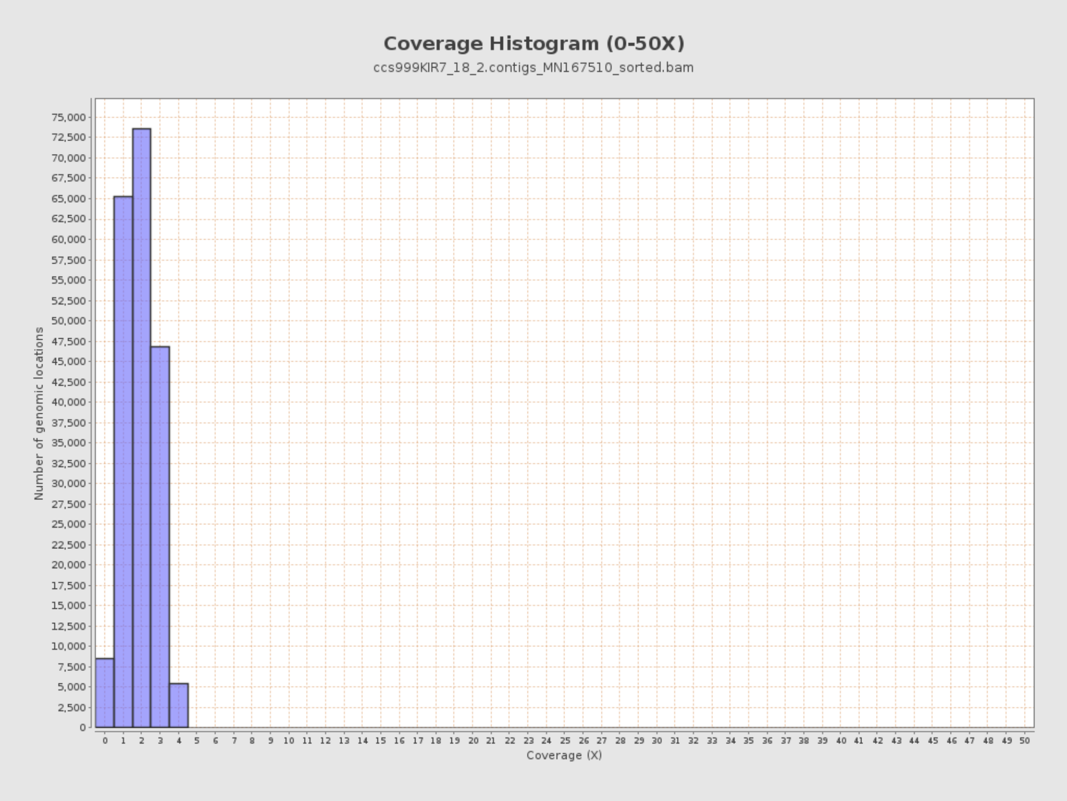

6. Results : Genome Fraction Coverage

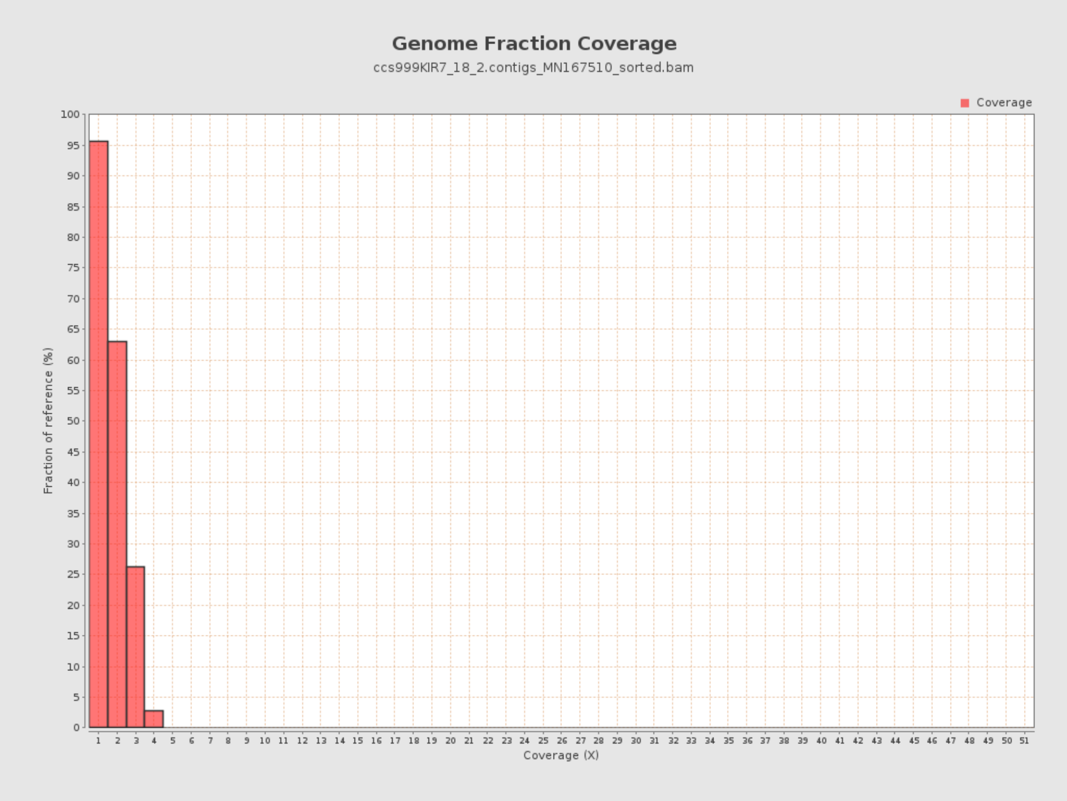

7. Results : Duplication Rate Histogram

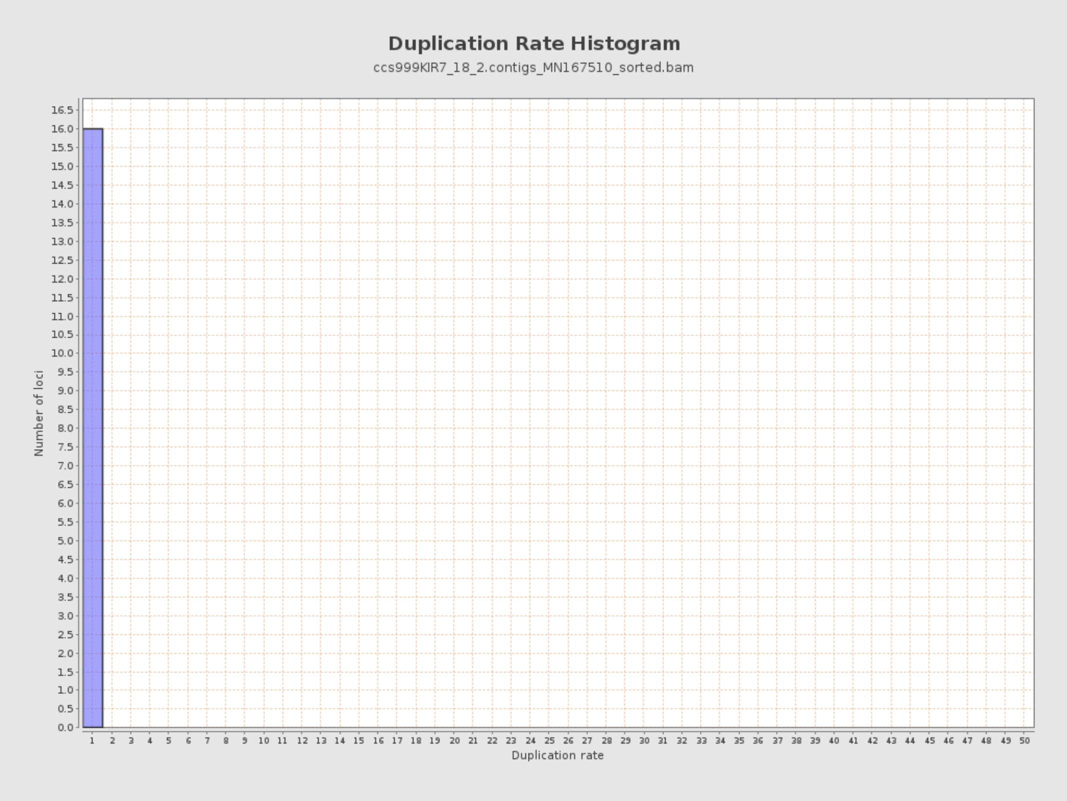

8. Results : Mapped Reads Nucleotide Content

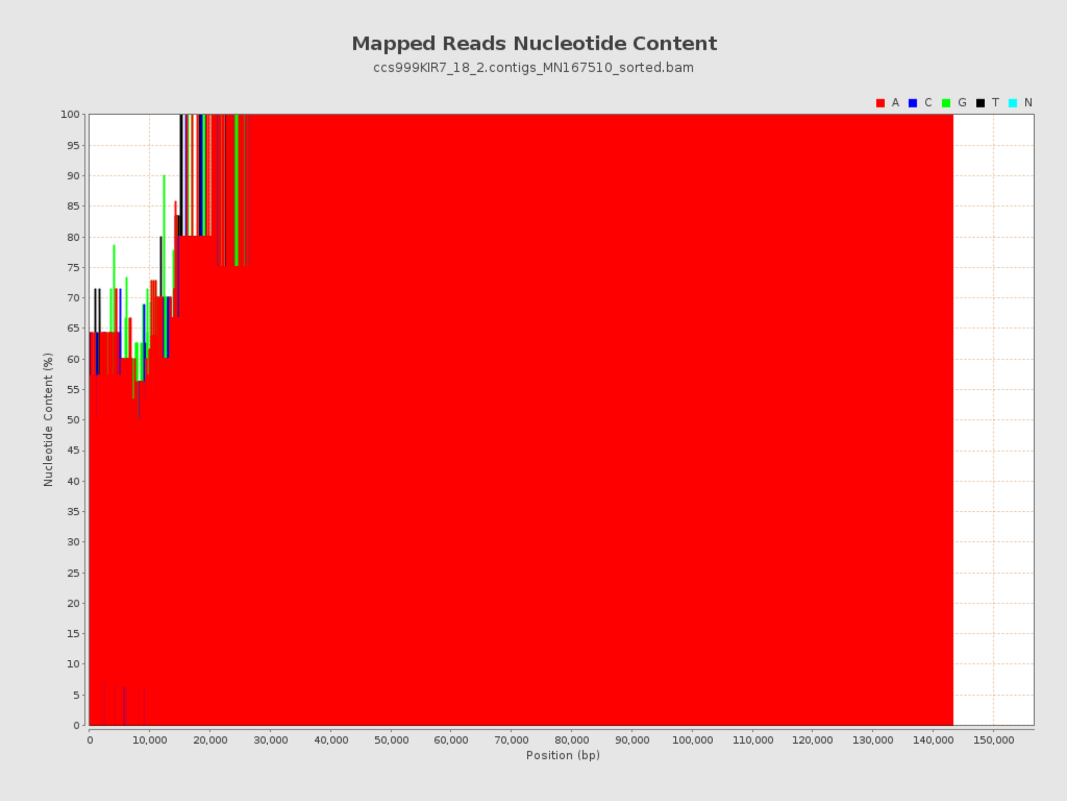

9. Results : Mapped Reads GC-content Distribution

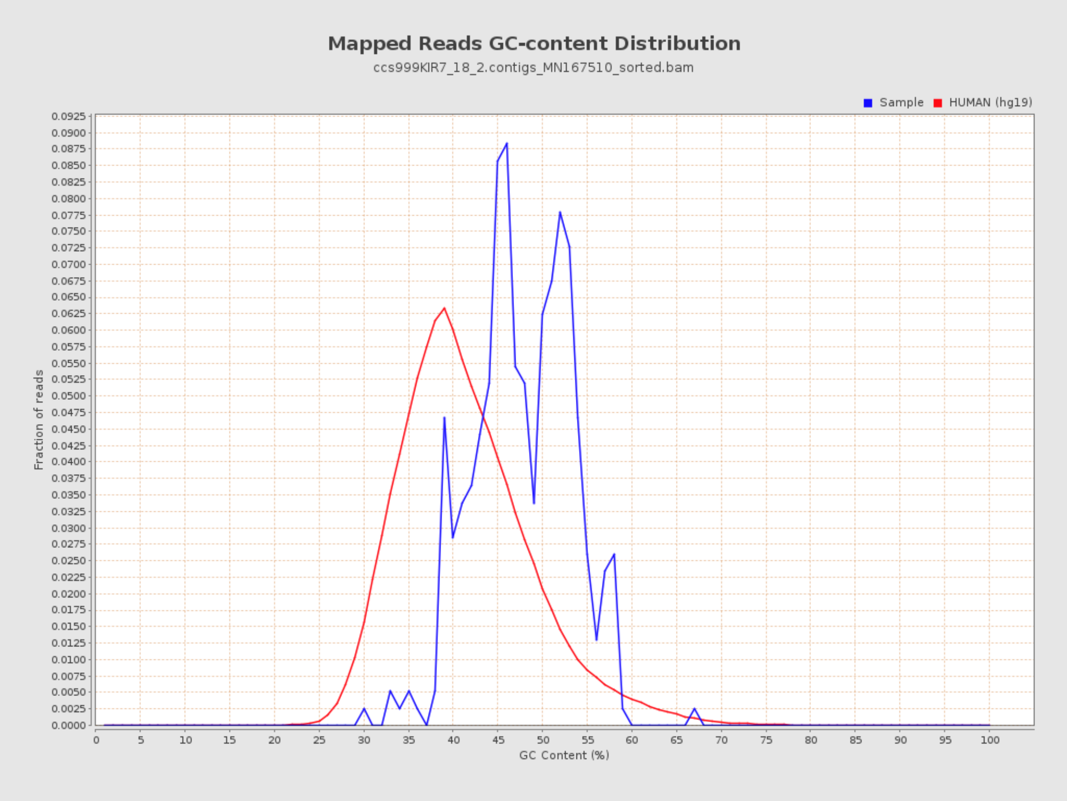

10. Results : Mapped Reads Clipping Profile

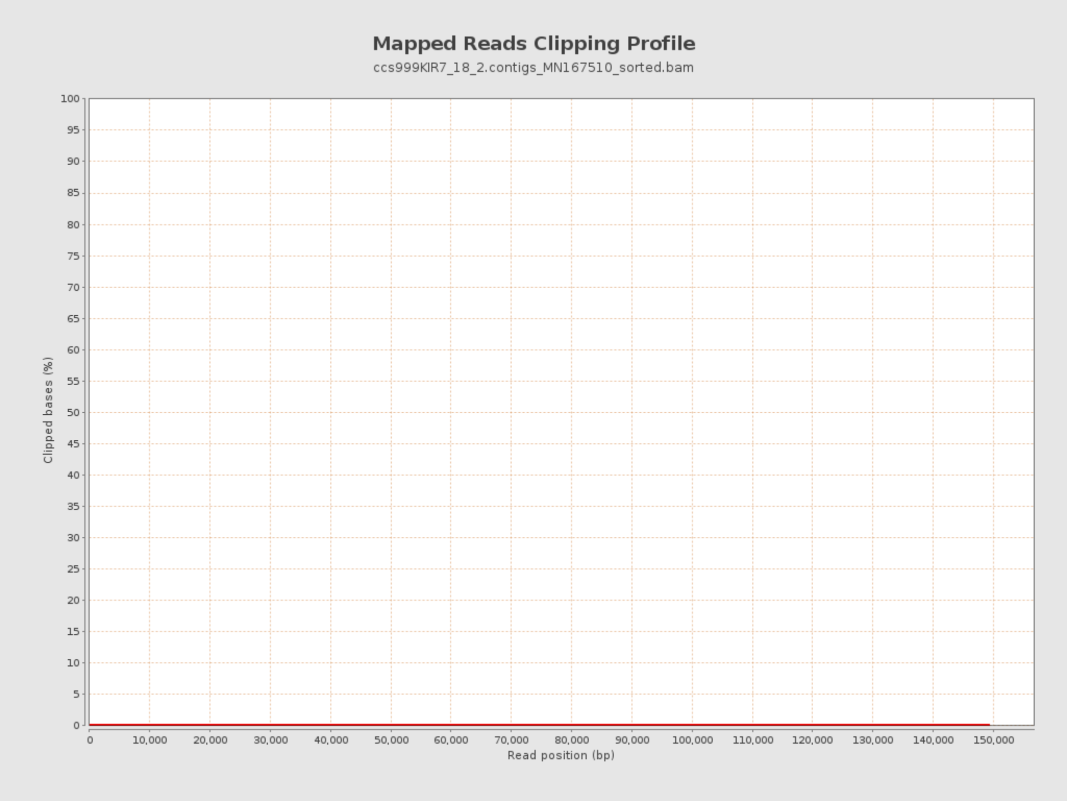

11. Results : Homopolymer Indels

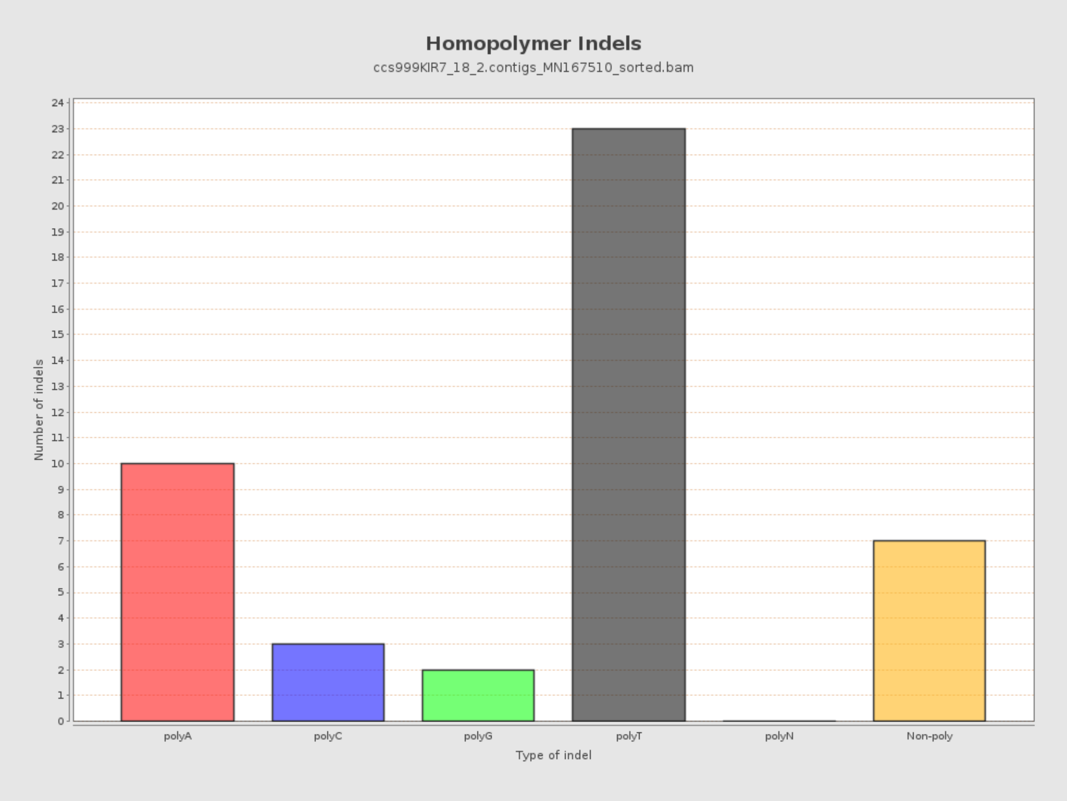

12. Results : Mapping Quality Across Reference

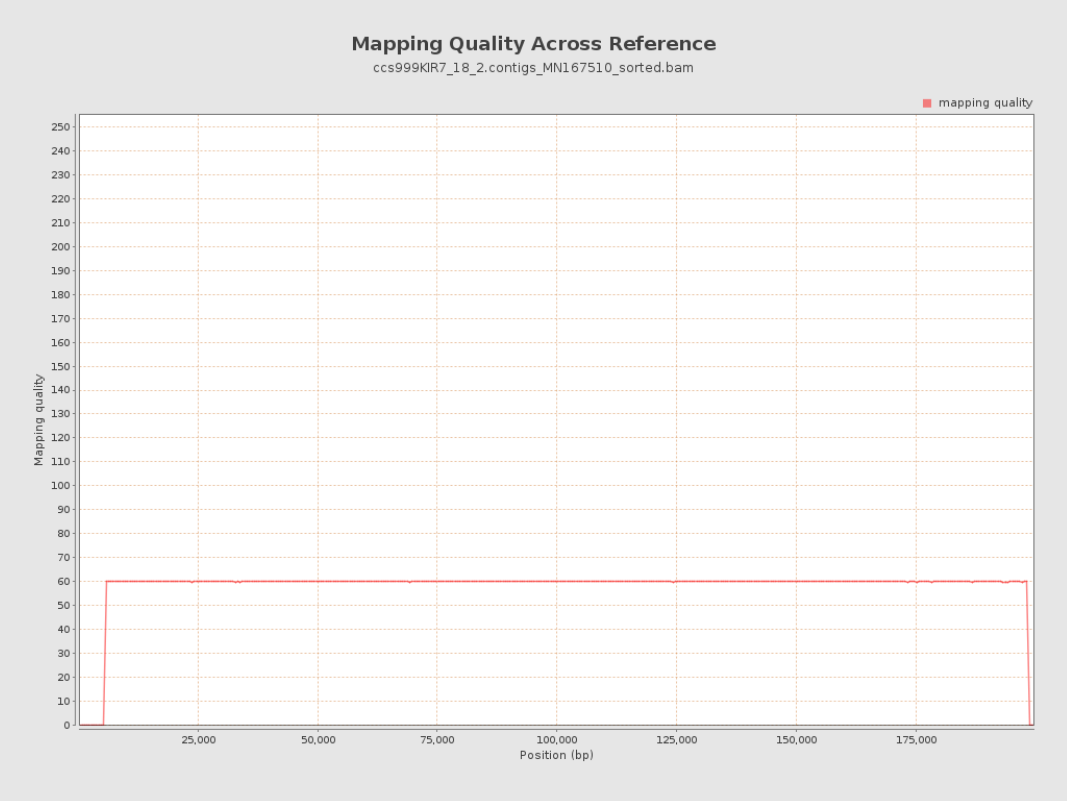

13. Results : Mapping Quality Histogram

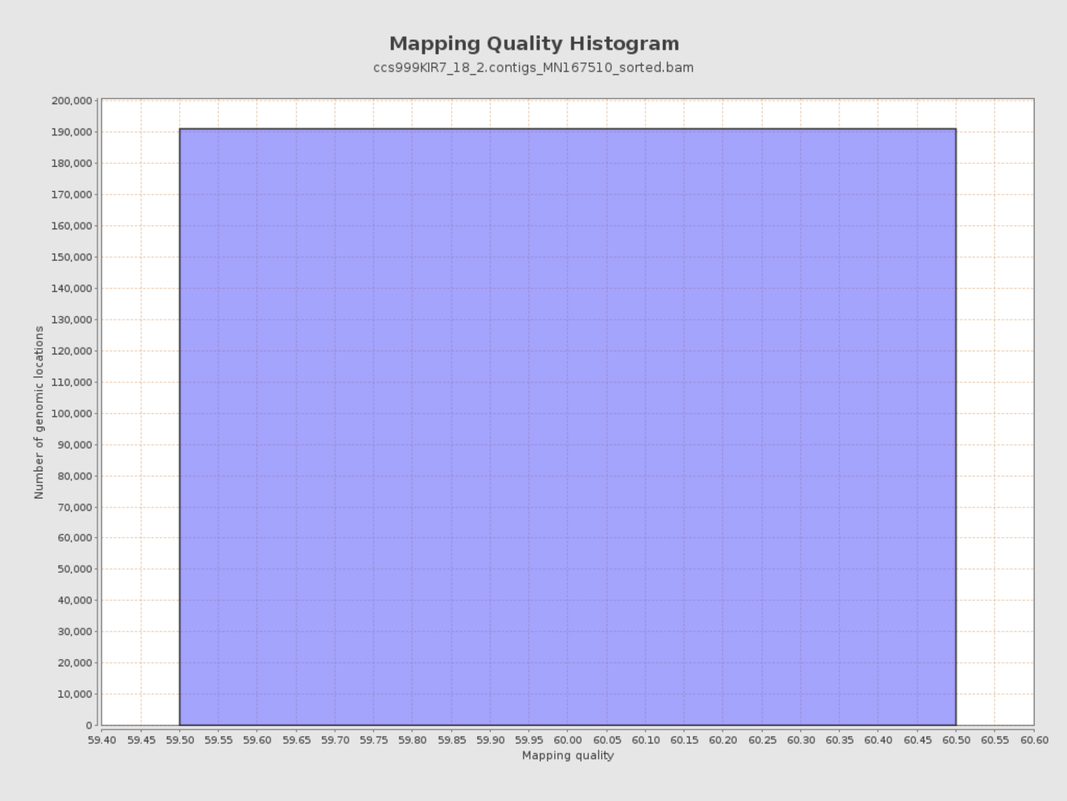

Supplement: Supplementary file 9 [file DataSheet_5.zip › SF1a/ccs999KIR7_18_2.contigs_MN167510_reports/ccs999KIR7_18_2.contigs_MN167510_qualimap.pdf]
