# Supplementary material for: Efficient Sequencing, Assembly, and Annotation of Human KIR Haplotypes
Source: Front Immunol. 2020 Oct 9;11:582927. doi: 10.3389/fimmu.2020.582927 (PMC7581912; doi:10.3389/fimmu.2020.582927)
Supplement: Supplementary file 9 [file DataSheet_5.zip › SF1a/ccs999KIR7_18_2.contigs_MN167510_reports/quast/icarus.html]

|  |
| --- |
| Icarus **QUAST Contig Browser** by CAB |

**Assemblies:** ccs999KIR7\_18\_2.contigs| Contig size viewer |
| QUAST report |

  

Contig alignment viewer

Aligned to sequences from MN167510.fasta

Fragments: 1, length: 199 689 bp, mean genome fraction: 98.395%,
misassembled blocks: 0
